# Supplementary material for: Enantioselective organocatalytic synthesis of axially chiral aldehyde-containing styrenes via SNAr reaction-guided dynamic kinetic resolution
Source: Nat Commun. 2023 Aug 19;14:5050. doi: 10.1038/s41467-023-40840-7 (PMC10439945; doi:10.1038/s41467-023-40840-7)
Supplement: Supplementary file 1 — Supplementary Information [file 41467_2023_40840_MOESM1_ESM.pdf]

Supplementary Information for

Enantioselective Organocatalytic Synthesis of Axially Chiral Aldehyde-containing Styrenes via S_NAr Reaction-Guided Dynamic Kinetic Resolution

Fengyuan Guo,[1,#] Siqiang Fang,[1,#] Jiajia He,[1,#] Zhishan Su,[1]* and Tianli Wang[1,2]*

[1] Key Laboratory of Green Chemistry & Technology of Ministry of Education, College of Chemistry, Sichuan University, Chengdu, P. R. China.
[2] Beijing National Laboratory for Molecular Sciences, Beijing 100190, China.
[#] These authors contributed equally: Fengyuan Guo, Siqiang Fang and Jiajia He.
email: suzhishan@scu.edu.cn; wangtl@scu.edu.cn

1. General information **2**

2. Optimization of reaction conditions **3**

3. Preparation of 1-enal substituted 2-naphthols **6**

4. Preparation of bifunctional phosphonium salts **17**

5. Procedure for the synthesis of axially chiral aldehyde-containing styrenes. **23**

6. Gram-scale preparations and transformations **82**

7. Study on axial stability of products **92**

8. Determination of absolute configuration of substrates and products **95**

9. Mechanism studies **96**

10. DFT studies **101**

11. NMR spectra **147**

12. Supplementary References **234**

1. General information

All the starting materials were obtained from commercial sources and used without further purification unless otherwise stated. ^{1}H and ^{13}C NMR spectra were recorded on a Bruker AVANCE III HD (400 MHz) spectrometer in $CDCl_3$ or $CDCl_3$ (Acetic acid-d_4 in $CDCl_3$). Chemical shifts (δ) are reported in ppm, and the residual solvent peak was used as an internal reference $CDCl_3$ [δ(^{1}H) = 7.26 ppm, δ (^{13}C) = 77.0 ppm], DMSO-d_6 [δ (^{1}H) = 2.50 ppm, δ (^{13}C) = 39.5 ppm], MeOH-d4 [δ (^{1}H) = 3.31 ppm, δ (^{13}C) = 49.0 ppm]. Multiplicity was indicated as follows: s (singlet), d (doublet), t (triplet), q (quartet), m (multiplet), dd (doublet of doublet), br s (broad singlet). Coupling constants (*J*) were reported in Hertz (Hz). All high resolution mass spectra were obtained on a Thermo LTQ mass spectrometer. For thin layer chromatography (TLC), Merck pre-coated TLC plates (Merck 60 F254) were used, and compounds were visualized with a UV light at 254 nm. Further visualization was achieved by staining with iodine, followed by heating on a hot plate. Flash chromatographic separations were performed on Merck 60 (0.040–0.063 mm) mesh silica gel. Enantiomeric excesses were determined by HPLC analysis using chiral column described below in detail. Optical rotations were measured with polarimeter.

All the 1-enal substituted 2-naphthols **1** were synthesized following the previous reported literature. The structure and absolute configurations of axially chiral arylpyrazoles were assigned by X-ray crystallographic analysis of the single crystal of chiral product **3**.

2. Optimization of reaction conditions

Supplementary Table 1. Screening of chiral bifunctional phosphonium salt catalyst.[a]

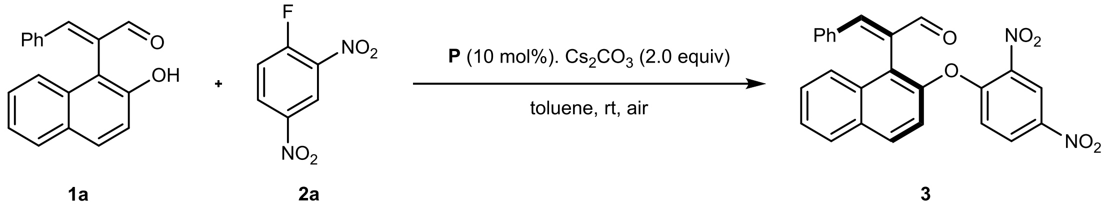

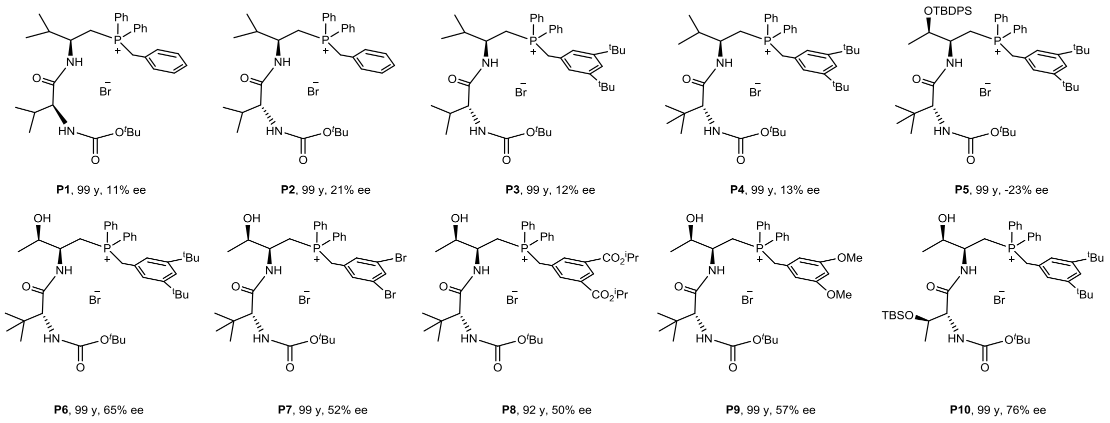

[a] Reaction condition: substrates **1a** (0.10 mmol), **2a** (0.12 mmol), Cs_2CO_3 (0.20 mmol) and **P** (10 mol%) in toluene (1.0 mL) at room temperature for 5 min. [b] Isolated yields based on **1a**. [c] The ee values were determined by chiral HPLC analysis. TBDPS = *tert*-butyldiphenylsilyl, TBS = *tert*-butyldimethylsilyl

Supplementary Table 2. Screening of the Base.[a]

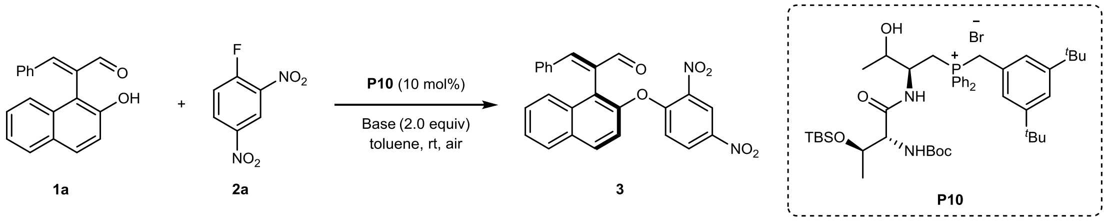

entry	Base	t/h	yield (%)[b]	ee (%)[c]
1	Cs_2CO_3	0.1	99	76
2	PhONa	12	72	81
3	K_2CO_3	4	99	84
4	K_3PO_4	2	99	78
5	K_3PO_4'$3H_2O$	2	99	81

6	K_3PO_4'$7H_2O$	2	99	82
**7**	CsOAc	4	98	75
8	$KHCO_3$	12	84	82
9	Et_3N	6	96	73
10	Na_2CO3	4	99	81
11	K_2HPO4	6	95	80
12	DMAP	0.5	97	10
13	NaOH	0.1	94	74

[a] Reaction condition: substrates **1a** (0.10 mmol), **2a** (0.12 mmol), Base (0.20 mmol) and **P10** (10 mol%) in toluene (1.0 mL) at room temperature. [b] Isolated yields based on **1a**. [c] The ee values were determined by chiral HPLC analysis.

Supplementary Table 3. Screening of the Solvent.[a]

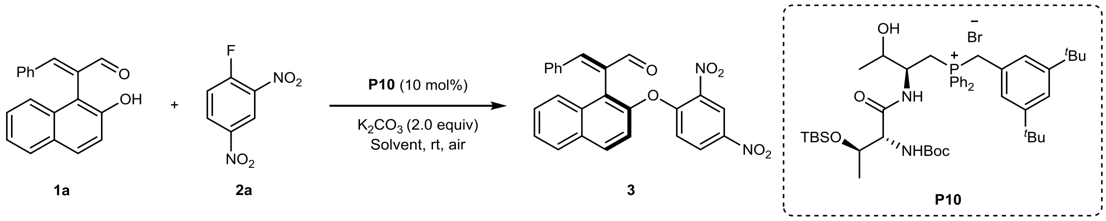

entry	Solvent	t/h	yield (%)[b]	ee (%)[c]
1	Tolune	4	99	82
2	Xylene	4	99	81
3	Mesitylene	4	99	77
4	Ethyl ether	4	99	75
5	Hexane	12	46	47
6	PE_{30-60}	12	35	53
7	PE_{60-90}	12	37	56
8	DCM	2	99	84
9	EA	1	99	79
10	CCl_4	2	96	78
11	*o*-Xylene	4	99	79
12	*m*-Xylene	4	99	80

13	*p*-Xylene	4	99	81
14	PhBr	4	99	81
15	Acetone	4	99	18
16	MeCN	1	99	6
17	$CHCl_3$	2	99	87
18	DMF	1	99	3
19	DMSO	1	99	1
20	THF	2	99	71

[a]Reaction condition: substrates **1a** (0.10 mmol), **2a** (0.12 mmol), K_2CO_3 (x equiv) and **P10** (10 mol%) in solvent (1.0 mL) at room temperature. [b] Isolated yields based on **1a**. [c] The ee values were determined by chiral HPLC analysis.

Supplementary Table 4. Screening of temperature and catalyst loading.[a]

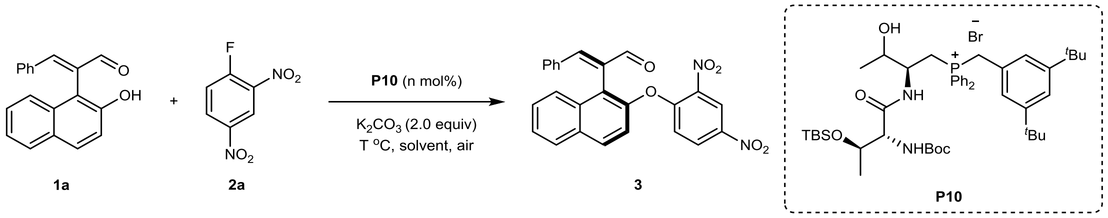

entry	T	**P10** (n mol%)	time (h)	yield (%)[b]	ee (%)[c]
1	rt	10 mol%	2	99	87
2	0 °C	10 mol%	5	99	92
3	-10 °C	10 mol%	9	99	96
4	-20 °C	10 mol%	22	98	94
5	-10 °C	5 mol%	12	99	96
6	-10 °C	2.5 mol%	17	99	96
7	-10 °C	1 mol%	24	99	96
8	-10 °C	0.1 mol%	48	99	24

[a]Reaction condition: substrates **1a** (0.10 mmol), **2a** (0.12 mmol), K_2CO_3 (0.20 mmol) and **P10** (n mol%) in $CHCl_3$ (1.0 mL) for hours. [b] Isolated yields based on **1a**. [c] The ee values were determined by chiral HPLC analysis.

3. Preparation of 1-enal substituted 2-naphthols

3.1. Synthesis of 1a-1ac.[1]

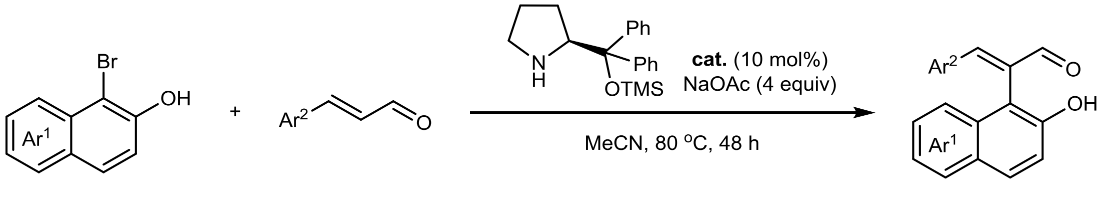

To a solution of **S1** (2.0 mmol), enal **S2** (4.0 mmol) and NaOAc (656.3 mg, 8.0 mmol) in CH_3CN (15 mL) was added diphenylprolinol TMS ether cat. (65.1 mg, 0.20 mmol) under N_2 atmosphere. Then the reaction mixture was stirred at 80 ^{o}C for 48 h. After the reaction was completed, the mixture was filtered and the filtrate was removed by vacuum distillation. The crude product was added with EtOAc (30 mL) and washed with brine (15 mL). The organic layer was dried with anhydrous Na_2SO_4 and concentrated in vacuo. The crude product was then purified by chromatography on silica gel to give **1a-1ac**.

(*E*)-3-([1,1'-biphenyl]-2-yl)-2-(2-hydroxynaphthalen-1-yl)acrylaldehyde (1b)

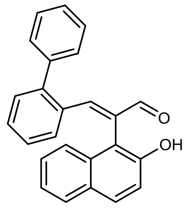

1b

A yellow solid, 64% yield, 448 mg; m.p: 154-156°C; **^{1}H NMR (400 MHz, $CDCl_3$)** δ 9.73 (s, 1H), 7.88 (s, 1H), 7.84 – 7.78 (m, 2H), 7.59 – 7.43 (m, 6H), 7.42 – 7.26 (m, 5H), 7.13 (d, *J* = 8.9 Hz, 1H), 6.98 (d, *J* = 6.9 Hz, 1H), 6.92 – 6.86 (m, 1H), 5.30 (s, 1H); **^{13}C NMR (100 MHz, $CDCl_3$)** δ 193.22, 151.79, 149.66, 142.86, 139.05, 134.57, 131.52, 130.74, 129.57, 129.38, 129.25, 128.90, 128.29, 127.68, 127.52, 127.50, 126.96, 126.39, 126.05, 122.92, 122.74, 117.08, 112.50; **HRMS (ESI, m/z)** calcd for $C_{25}H_{18}O_2$ $[M+Na]^+$ = 373.1205, found = 373.1190.

(*E*)-2-(2-hydroxynaphthalen-1-yl)-3-(2-methoxyphenyl)acrylaldehyde (1c)

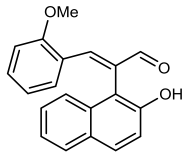

1c

A yellow solid, 76% yield, 462 mg; m.p: 147-148 ^{o}C; **^{1}H NMR (400 MHz, CDCl$_3$)** δ 9.94 (s, 1H), 8.43 (s, 1H), 7.78 (t, *J* = 8.8 Hz, 2H), 7.52 – 7.39 (m, 1H), 7.32 (m, *J* = 11.9, 6.8, 1.6 Hz, 2H), 7.28 – 7.18 (m, 1H), 7.14 (d, *J* = 8.9 Hz, 1H), 6.87 (d, *J* = 8.3 Hz, 1H), 6.82 (dd, *J* = 7.9, 1.5 Hz, 1H), 6.48 (t, *J* = 7.6 Hz, 1H), 5.66 (s, 1H), 3.93 (s, 3H); **^{13}C NMR (100 MHz, CDCl$_3$)** δ 194.72, 158.32, 150.83, 148.18, 134.67, 132.81, 132.30, 130.57, 129.49, 129.33, 128.51, 127.03, 124.04, 123.72, 122.67, 120.81, 118.20, 113.59, 110.86, 55.83; **HRMS (ESI, m/z)** calcd for $C_{20}H_{17}O_3$ $[M+Na]^+$ = 327.0997, found = 327.1001.

(*E*)-3-(2-bromophenyl)-2-(2-hydroxynaphthalen-1-yl)acrylaldehyde (1d)

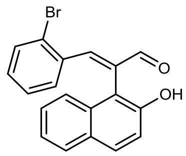

1d

A yellow solid, 66% yield, 466 mg; m.p: 152-154 ^{o}C; **^{1}H NMR (400 MHz, CDCl$_3$)** δ 9.97 (s, 1H), 8.21 (s, 1H), 7.77 – 7.70 (m, 2H), 7.57 (dd, *J* = 8.0, 0.9 Hz, 1H), 7.42 (d, *J* = 8.3 Hz, 1H), 7.37 – 7.27 (m, 2H), 7.10 – 7.00 (m, 2H), 6.89 (dd, *J* = 7.9, 1.6 Hz, 1H), 6.81 (t, *J* = 7.6 Hz, 1H), 5.79 (s, 1H); **^{13}C NMR (100 MHz, CDCl$_3$)** δ 194.39, 151.68, 151.30, 137.54, 133.91, 133.08, 132.20, 131.62, 130.86, 130.07, 129.22, 128.54, 127.49, 127.22, 125.59, 123.80, 123.74, 118.16, 112.58; **HRMS (ESI, m/z)** calcd for $[M+Na]^+$ = 374.9997, found = 374.9994.

(*E*)-2-(2-hydroxynaphthalen-1-yl)-3-(2-nitrophenyl)acrylaldehyde (1f)

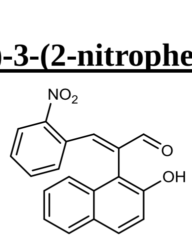

1f

A yellow solid, 58% yield, 370 mg; m.p: 141-143 ^{o}C; **^{1}H NMR (400 MHz, CDCl$_3$)** δ 10.02 (s, 1H), 8.25 (s, 1H), 8.06 (dd, *J* = 8.2, 1.0 Hz, 1H), 7.76 – 7.67 (m, 2H), 7.50 (d, *J* = 8.4 Hz, 1H), 7.41 – 7.27 (m, 3H), 7.23 – 7.17 (m, 1H), 7.03 – 6.97 (m, 2H), 5.87 (s, 1H); **^{13}C NMR (100 MHz, CDCl$_3$)** δ 193.36, 151.76, 149.42, 147.44, 138.10,

133.80, 132.89, 131.07, 130.58, 130.44, 130.33, 129.01, 128.60, 127.44, 124.89, 123.85, 123.73, 118.05, 111.82; **HRMS (ESI, m/z)** calcd For $C_{19}H_{13}NO_4$ $[M+Na]^+$ = 342.0742, found = 342.0741.

(*E*)-3-(3-chlorophenyl)-2-(2-hydroxynaphthalen-1-yl)acrylaldehyde (1g)

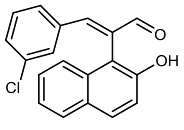

1g

A yellow solid, 69% yield, 426 mg; m.p: 59-61 °C; **^{1}H NMR (400 MHz, $CDCl_3$)** δ 9.87 (s, 1H), 7.86 – 7.82 (m, 2H), 7.76 (s, 1H), 7.43 – 7.33 (m, 5H), 7.16 (d, *J* = 8.9 Hz, 1H), 7.05 – 6.94 (m, 2H), 5.69 (s, 1H); **^{13}C NMR (100 MHz, $CDCl_3$)** δ 194.20, 150.99, 150.86, 136.68, 135.81, 133.77, 132.02, 131.07, 130.36, 129.37, 128.69, 128.45, 127.40, 124.02, 123.65, 122.79, 118.20, 112.90; **HRMS (ESI, m/z)** calcd for $C_{19}H_{13}ClO_2$ $[M+Na]^+$ = 331.0502, found = 331.0509.

(*E*)-3-(2-bromo-4-(trifluoromethyl)phenyl)-2-(2-hydroxynaphthalen-1-yl)acrylaldehyde (1n)

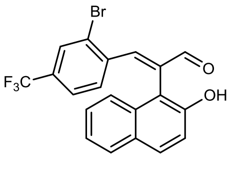

1n

A yellow solid, 52% yield, 438 mg; m.p:149-150 °C; **^{1}H NMR (400 MHz, $CDCl_3$)** δ 9.98 (s, 1H), 8.11 (s, 1H), 7.81 (s, 1H), 7.77 – 7.69 (m, 2H), 7.43 – 7.27 (m, 3H), 7.12 – 6.97 (m, 3H), 5.90 (s, 1H); **^{13}C NMR (100 MHz, $CDCl_3$)** δ 194.10, 151.41, 149.44, 139.41, 137.78, 134.63, 133.97, 132.79 (q, *J* = 33.4 Hz), 132.13, 131.87, 131.16, 130.25, 129.91 (q, *J* = 3.7 Hz), 129.17, 128.68, 127.51, 126.91, 125.97, 125.20, 124.26 (q, *J* = 3.6 Hz), 123.99, 123.89, 123.38, 122.76 (q, *J* = 273.0 Hz), 118.04, 112.86, 112.02; **^{19}F NMR (376 MHz, $CDCl_3$)** δ -63.17; **HRMS (ESI, m/z)** calcd for $C_{20}H_{12}BrF_3O_2$ $[M+Na]^+$ = 442.9871, found = 442.9873.

(*E*)-3-(3,5-di-*tert*-butylphenyl)-2-(2-hydroxynaphthalen-1-yl)acrylaldehyde (1o)

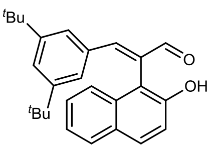

1o

A yellow solid, 79% yield, 587 mg; m.p: 66-67°C; **^{1}H NMR (400 MHz, CDCl$_3$)** δ 9.94 (s, 1H), 7.94 (s, 1H), 7.82 (dt, *J* = 7.6, 2.2 Hz, 2H), 7.49 – 7.44 (m, 1H), 7.32 (ddd, *J* = 5.1, 3.2, 1.8 Hz, 3H), 7.21 (d, *J* = 8.9 Hz, 1H), 7.05 (d, *J* = 1.8 Hz, 2H), 5.33 (s, 1H), 1.02 (s, 18H); **^{13}C NMR (100 MHz, CDCl$_3$)** δ 194.33, 154.95, 151.35, 150.58, 134.35, 132.80, 132.29, 130.68, 129.62, 128.56, 127.25, 125.80, 125.63, 124.02, 123.91, 118.21, 114.02, 34.70, 31.10; **HRMS (ESI, m/z)** calcd for $C_{27}H_{30}O_2$ $[M+Na]^+$ = 409.2143, found = 409.2148.

(*E*)-2-(2-hydroxynaphthalen-1-yl)-3-(thiophen-2-yl)acrylaldehyde (1q)

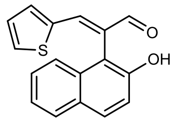

1q

A yellow solid, 64% yield, 359 mg; m.p: 181-182 °C; **^{1}H NMR (400 MHz, CDCl$_3$)** δ 9.85 (s, 1H), 8.07 (s, 1H), 7.89 – 7.80 (m, 2H), 7.47 – 7.38 (m, 1H), 7.38 – 7.29 (m, 3H), 7.27 (d, *J* = 5.1 Hz, 1H), 7.19 (d, *J* = 8.9 Hz, 1H), 6.95 (dd, *J* = 5.0, 3.8 Hz, 1H), 5.66 (s, 1H); **^{13}C NMR (100 MHz, CDCl$_3$)** δ 193.24, 151.63, 145.88, 137.62, 135.33, 134.09, 132.58, 132.12, 131.38, 129.62, 128.57, 127.37, 127.30, 123.96, 123.40, 118.39, 112.41; **HRMS (ESI, m/z)** calcd for $C_{17}H_{12}O_2S$ $[M+H]^+$ = 281.0636, found = 281.0636.

(*E*)-2-(2-hydroxy-7-phenylnaphthalen-1-yl)-3-phenylacrylaldehyde (1r)

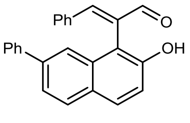

1r

A yellow solid, 73% yield, 518 mg; m.p: 161-162 °C; **^{1}H NMR (400 MHz, CDCl$_3$)** δ 9.89 (s, 1H), 7.89 (t, *J* = 4.4 Hz, 2H), 7.83 (d, *J* = 8.9 Hz, 1H), 7.62 – 7.54 (m, 2H), 7.50 – 7.45 (m, 2H), 7.39 (t, *J* = 7.5 Hz, 2H), 7.32 (dd, *J* = 8.3, 6.2 Hz, 1H), 7.28 – 7.26 (m, 1H), 7.24 – 7.18 (m, 2H), 7.16 (d, *J* = 8.6 Hz, 3H), 5.66 (s, 1H); **^{13}C NMR (100 MHz, CDCl$_3$)** δ 194.54, 153.65, 151.28, 141.41, 139.99, 135.08, 133.74, 132.41, 131.32, 130.77, 130.56, 129.10, 128.97, 128.81, 128.58, 127.65, 127.46, 123.76, 122.10, 118.30, 113.64; **HRMS (ESI, m/z)** calcd for $C_{25}H_{18}O_2$ $[M+Na]^+$ = 373.1205, found = 373.1192.

(*E*)-2-(7-cyclopropyl-2-hydroxynaphthalen-1-yl)-3-phenylacrylaldehyde (1t)

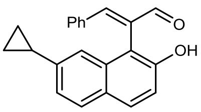

1t

A yellow solid, 81% yield, 509 mg; m.p: 81-82 °C; **^{1}H NMR (400 MHz, CDCl$_3$)** δ 9.83 (s, 1H), 7.83 (s, 1H), 7.71 (dd, *J* = 11.2, 8.7 Hz, 2H), 7.26 (dt, *J* = 13.4, 1.8 Hz, 1H), 7.20 – 7.10 (m, 5H), 7.05 (d, *J* = 8.8 Hz, 1H), 6.99 (dd, *J* = 8.4, 1.7 Hz, 1H), 5.73 (s, 1H), 1.89 (ddd, *J* = 13.5, 8.4, 5.1 Hz, 1H), 0.96 – 0.85 (m, 2H), 0.72 – 0.51 (m, 2H); **^{13}C NMR (100 MHz, CDCl$_3$)** δ 194.70, 153.21, 151.04, 142.92, 135.23, 133.84, 132.26, 131.13, 130.72, 130.44, 128.89, 128.58, 127.76, 122.04, 120.27, 117.12, 112.70, 16.00, 9.64, 9.35; **HRMS (ESI, m/z)** calcd for $C_{22}H_{18}O_2$ $[M+Na]^+$ = 337.1205, found = 337.1195.

<u>(*E*)-2-(7-ethyl-2-hydroxynaphthalen-1-yl)-3-phenylacrylaldehyde (1u)</u>

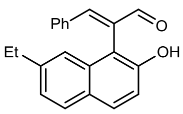

1u

A yellow solid, 67% yield, 405 mg; m.p: 94-96 °C; **^{1}H NMR (400 MHz, CDCl$_3$)** δ 9.83 (s, 1H), 7.82 (s, 1H), 7.77 – 7.69 (m, 2H), 7.27 – 7.09 (m, 8H), 7.06 (d, *J* = 8.9 Hz, 1H), 5.76 (s, 1H), 2.63 (q, *J* = 7.6 Hz, 2H), 1.13 (t, *J* = 7.6 Hz, 3H); **^{13}C NMR (100 MHz, CDCl$_3$)** δ 194.73, 153.20, 150.96, 143.22, 135.33, 133.88, 132.41, 131.09, 130.72, 130.46, 128.87, 128.49, 127.81, 124.96, 121.85, 117.30, 112.96, 29.36, 15.59; **HRMS (ESI, m/z)** calcd for $C_{21}H_{18}O_2$ $[M+H]^+$ = 303.1385, found = 303.1384.

<u>(*E*)-2-(2-hydroxy-7-methylnaphthalen-1-yl)-3-phenylacrylaldehyde (1v)</u>

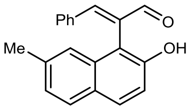

1v

A yellow solid, 78% yield, 450 mg; m.p: 159-160 °C; **^{1}H NMR (400 MHz, CDCl$_3$)** δ 9.87 (s, 1H), 7.86 (s, 1H), 7.77 (d, *J* = 8.8 Hz, 1H), 7.72 (d, *J* = 8.2 Hz, 1H), 7.28 (d, *J* = 6.9 Hz, 1H), 7.22 – 7.13 (m, 6H), 7.08 (d, *J* = 8.8 Hz, 1H), 5.43 (s, 1H), 2.36 (s, 3H); **^{13}C NMR (100 MHz, CDCl$_3$)** δ 194.46, 153.12, 150.75, 137.04, 135.17, 133.79, 132.50, 131.22, 130.74, 130.52, 128.96, 128.41, 127.62, 126.21, 122.99, 117.13, 112.79, 22.13; **HRMS (ESI, m/z)** calcd for $C_{20}H_{16}O_2$ $[M+Na]^+$ = 311.1048, found = 311.1036.

(*E*)-2-(6-cyclopropyl-2-hydroxynaphthalen-1-yl)-3-phenylacrylaldehyde (1x)

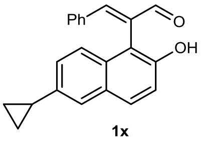

1x

A yellow solid, 71% yield, 446 mg; m.p: 172-173 °C; **^{1}H NMR (400 MHz, CDCl$_3$)** δ 9.85 (s, 1H), 7.84 (s, 1H), 7.73 (d, *J* = 8.9 Hz, 1H), 7.52 (d, *J* = 1.5 Hz, 1H), 7.33 (d, *J* = 8.7 Hz, 1H), 7.29 – 7.23 (m, 1H), 7.20 – 7.16 (m, 3H), 7.15 – 7.09 (m, 2H), 7.06 (dd, *J* = 8.7, 1.8 Hz, 1H), 5.39 (s, 1H), 2.00 (ddd, *J* = 13.5, 8.5, 5.1 Hz, 1H), 1.02 – 0.93 (m, 2H), 0.80 – 0.68 (m, 2H); **^{13}C NMR (100 MHz, CDCl$_3$)** δ 194.38, 153.14, 150.00, 139.36, 135.18, 133.74, 131.22, 130.75, 130.49, 130.12, 129.55, 128.96, 126.04, 124.66, 123.91, 118.28, 113.31, 15.43, 9.10, 8.99; **HRMS (ESI, m/z)** calcd for $C_{22}H_{18}O_2$ $[M+Na]^+$ = 337.1205, found = 337.1197.

(E)-2-(6-ethyl-2-hydroxynaphthalen-1-yl)-3-phenylacrylaldehyde (1y)

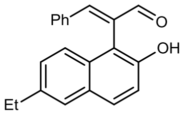

1y

A yellow solid, 73% yield, 441 mg; m.p: 191-193 °C; **^{1}H NMR (400 MHz, DMSO-*d$_6$*)** δ 9.87 (s, 1H), 9.49 (s, 1H), 7.97 (s, 1H), 7.78 (d, *J* = 8.9 Hz, 1H), 7.62 (s, 1H), 7.29 – 7.14 (m, 8H), 2.67 (q, *J* = 7.5 Hz, 2H), 1.21 (t, *J* = 7.6 Hz, 3H); **^{13}C NMR (100 MHz, DMSO-*d$_6$*)** δ 194.61, 151.80, 151.71, 137.97, 137.06, 134.58, 130.62, 130.29, 129.95, 129.07, 128.60, 128.13, 127.63, 125.85, 123.27, 118.27, 113.22, 27.92, 15.45; **HRMS (ESI, m/z)** calcd for $C_{21}H_{18}O_2$ $[M+H]^+$ = 303.1385, found = 303.1383.

(*E*)-2-(2-hydroxy-6-methylnaphthalen-1-yl)-3-phenylacrylaldehyde (1z)

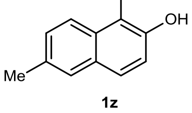

1z

A yellow solid, 77% yield, 444 mg; m.p: 179-180 °C; **^{1}H NMR (400 MHz, CDCl$_3$)** δ 9.86 (s, 1H), 7.85 (s, 1H), 7.73 (d, *J* = 8.9 Hz, 1H), 7.59 (s, 1H), 7.33 (d, *J* = 8.6 Hz, 1H), 7.30 – 7.23 (m, 1H), 7.19 (s, 1H), 7.16 (t, *J* = 2.9 Hz, 3H), 7.13 (dd, *J* = 8.4, 5.8 Hz, 2H), 5.42 (s, 1H), 2.45 (s, 3H); **^{13}C NMR (100 MHz, CDCl$_3$)** δ 194.42, 153.18, 150.08, 135.24, 133.77, 133.38, 131.21, 130.74, 130.31, 130.11, 129.62, 129.46, 128.95, 127.67, 123.77, 118.20, 113.31, 21.44; **HRMS (ESI, m/z)** calcd for $C_{20}H_{16}O_2$ $[M+Na]^+$ = 311.1048, found = 311.1039.

methyl (*E*)-6-hydroxy-5-(3-oxo-1-phenylprop-1-en-2-yl)-2-naphthoate (1ab)

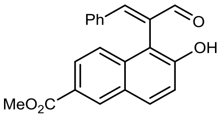

1ab

A yellow solid, 48% yield, 319 mg; m.p: 164-165 °C; **^{1}H NMR (400 MHz, CDCl$_3$)** δ 9.85 (s, 1H), 8.55 (d, *J* = 1.4 Hz, 1H), 7.91 – 7.82 (m, 3H), 7.43 (d, *J* = 8.8 Hz, 1H), 7.24 (dd, *J* = 5.2, 3.5 Hz, 1H), 7.18 (d, *J* = 8.9 Hz, 1H), 7.12 (d, *J* = 3.9 Hz, 4H), 6.41 (s, 1H), 3.93 (s, 3H); ^{13}C NMR (100 MHz, CDCl$_3$) δ 194.53, 167.49, 154.18, 153.33, 134.92, 134.78, 133.62, 132.21, 131.77, 131.35, 130.68, 128.96, 128.26, 126.61, 125.33, 124.05, 119.17, 113.54, 52.31; **HRMS (ESI, m/z)** calcd for $C_{21}H_{16}O_4$ $[M+Na]^+$ = 355.0947, found = 355.0947.

(*E*)-2-(6-hydroxyquinolin-5-yl)-3-phenylacrylaldehyde (1ac)

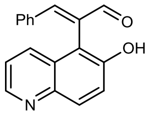

1ac

A yellow solid, 57% yield, 314 mg; m.p: 213-215 °C; **^{1}H NMR (400 MHz, MeOH-*d$_4$*)** δ 9.88 (s, 1H), 8.61 (dd, *J* = 4.2, 1.5 Hz, 1H), 8.07 – 7.94 (m, 2H), 7.86 (d, *J* = 8.4 Hz, 1H), 7.49 (d, *J* = 9.2 Hz, 1H), 7.30 (dd, *J* = 8.6, 4.3 Hz, 1H), 7.28 – 7.23 (m, 1H), 7.22 – 7.12 (m, 4H); **^{13}C NMR (100 MHz, MeOH-*d$_4$*)** δ 196.00, 154.75, 154.59, 148.01, 144.33, 137.00, 135.72, 134.20, 131.73, 131.37, 130.94, 129.67, 129.19, 123.26, 122.73, 114.93; **HRMS (ESI, m/z)** calcd for C18H13NO2 $[M+H]^+$ = 276.1024, found = 276.1015.

3.2. Synthesis of 1ad-1al.[2]

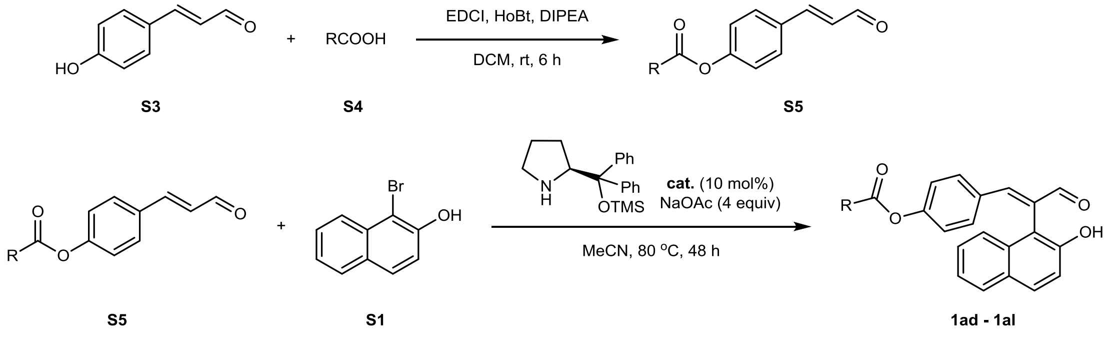

A mixture of **S3** (444.5 mg, 3.0 mmol), **S4** (3.3 mmol), EDCI (862.7 mg, 4.5

mmol), HoBt (608.0 mg, 4.5 mmol), DIPEA (775.5 mg, 6 mmol) in DCM (20 ml) was stirred for 12 h at room tempreture. The reaction mixture was added H_2O (30 mL), and the mixture was extracted with DCM (20 mL×3), dried over $MgSO_4$, and the solvent was removed under reduced pressure, and the residue was purified by flash column to give **S5**.

To a solution of **S1** (2.0 mmol), enal **S5** (4.0 mmol) and NaOAc (656.3 mg, 8.0 mmol) in CH_3CN (15 mL) was added diphenylprolinol TMS ether catalyst (65.1 mg, 0.20 mmol) under N_2 atmosphere. Then the reaction mixture was stirred at 80 ^oC for 48 h. After the reaction was completed, the mixture was filtered and the filtrate was removed by vacuum distillation. The crude product was added with EtOAc (30 mL) and washed with brine (15 mL). The organic layer was dried with anhydrous Na_2SO_4 and concentrated in vacuo. The crude product was then purified by chromatography on silica gel to give **1ad-1al**.

4-((*E*)-2-(2-hydroxynaphthalen-1-yl)-3-oxoprop-1-en-1-yl)phenyl (4*R*)-4-((3*R*,5*R*,8*R*,10*S*,13*R*,14*S*,17*R*)-3-hydroxy-10,13-dimethylhexadecahydro-1H-cyclopenta[a]phenanthren-17-yl)pentanoate (1ad)

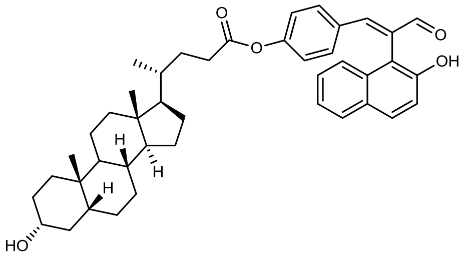

1ad

A yellow solid, 49% yield, 799 mg; m.p: 113-115 ^oC; **^1H NMR (400 MHz, $CDCl_3$)** δ 9.85 (s, 1H), 7.80 (d, *J* = 7.9 Hz, 3H), 7.46 – 7.37 (m, 1H), 7.36 – 7.28 (m, 2H), 7.21 – 7.12 (m, 3H), 6.87 (d, *J* = 8.6 Hz, 2H), 5.96 (s, 1H), 3.61 (m, *J* = 10.6, 4.5 Hz, 1H), 2.52 (m, *J* = 14.9, 9.5, 5.0 Hz, 1H), 2.39 (dt, *J* = 15.6, 7.8 Hz, 1H), 1.94 (d, *J* = 11.7 Hz, 1H), 1.89 – 1.76 (m, 4H), 1.68 (dd, *J* = 27.8, 12.0 Hz, 4H), 1.55 (s, 1H), 1.51 (s, 1H), 1.47 (d, *J* = 5.2 Hz, 1H), 1.39 (d, *J* = 9.0 Hz, 3H), 1.35 (d, *J* = 14.4 Hz, 3H), 1.29 (s, 1H), 1.24 (d, *J* = 14.1 Hz, 3H), 1.14 – 1.06 (m, 2H), 1.06 – 1.01 (m, 2H), 0.97 (dd, *J* = 14.5, 3.3 Hz, 1H), 0.93 (d, *J* = 6.3 Hz, 3H), 0.91 (s, 3H), 0.63 (s, 3H); **^{13}C NMR (100 MHz, $CDCl_3$)** δ 194.28, 172.42, 152.70, 151.86, 151.00, 135.24, 132.22, 132.04, 131.43, 130.77, 129.31, 128.59, 127.29, 123.89, 123.79, 122.11, 118.27, 113.19, 72.06, 56.60, 55.98, 42.87, 42.18, 40.54, 40.27, 36.48, 35.95, 35.44, 34.67, 31.42, 30.95, 30.58, 28.33, 27.28, 26.52, 24.30, 23.49, 20.93, 18.38, 12.17; **HRMS (ESI,**

m/z) calcd for $C_{43}H_{52}O_5$ $[M+Na]^+$ = 671.3713, found = 671.3714.

(*E*)-4-(2-(2-hydroxynaphthalen-1-yl)-3-oxoprop-1-en-1-yl)phenyl 5-(2,5-dimethylphenoxy)-2,2-dimethylpentanoate (1ae)

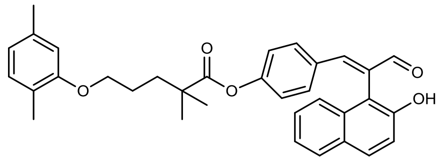

1ae

A yellow solid, 63% yield, 658 mg; m.p: 54-56 °C; **^{1}H NMR (400 MHz, CDCl$_3$)** δ 9.84 (s, 1H), 7.84 – 7.77 (m, 3H), 7.45 – 7.39 (m, 1H), 7.35 (q, *J* = 5.3, 4.3 Hz, 2H), 7.18 – 7.10 (m, 3H), 6.96 (d, *J* = 7.4 Hz, 1H), 6.80 (d, *J* = 8.7 Hz, 2H), 6.61 (d, *J* = 7.6 Hz, 1H), 6.58 (s, 1H), 5.75 (s, 1H), 3.94 (t, *J* = 5.3 Hz, 2H), 2.26 (s, 3H), 2.12 (s, 3H), 1.85 – 1.76 (m, 4H), 1.30 (s, 6H); **^{13}C NMR (100 MHz, CDCl$_3$)** δ 194.39, 176.08, 156.80, 152.97, 152.20, 150.89, 136.59, 135.10, 132.17, 132.05, 131.35, 130.81, 130.48, 129.33, 128.61, 127.32, 123.93, 123.76, 123.68, 122.13, 120.89, 118.24, 113.11, 112.02, 67.66, 42.61, 37.15, 25.24, 25.15, 21.49, 15.89; **HRMS (ESI, m/z)** calcd for $C_{34}H_{34}O_5$ $[M+Na]^+$ = 545.2304, found = 545.2303.

(*E*)-4-(2-(2-hydroxynaphthalen-1-yl)-3-oxoprop-1-en-1-yl)phenyl 4-([1,1'-biphenyl]-4-yl)-4-oxobutanoate (1af)

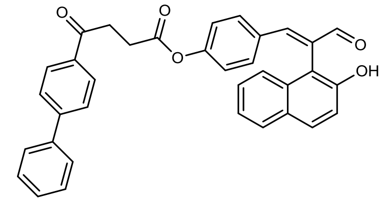

1af

A yellow solid, 75% yield, 789 mg; m.p: 86-88 °C; **^{1}H NMR (400 MHz, CDCl$_3$)** δ 9.84 (s, 1H), 8.02 (d, *J* = 8.5 Hz, 2H), 7.84 – 7.77 (m, 3H), 7.71 – 7.64 (m, 2H), 7.64 – 7.59 (m, 2H), 7.51 – 7.44 (m, 2H), 7.44 – 7.38 (m, 2H), 7.37 – 7.29 (m, 2H), 7.20 – 7.11 (m, 3H), 6.96 – 6.89 (m, 2H), 5.69 (s, 1H), 3.38 (t, *J* = 6.4 Hz, 2H), 2.94 (t, *J* = 6.4 Hz, 2H); **^{13}C NMR (100 MHz, CDCl$_3$)** δ 197.53, 194.26, 171.30, 152.69, 152.04, 150.87, 146.22, 139.86, 135.10, 135.07, 132.17, 132.06, 131.45, 130.83, 129.35, 129.10, 128.77, 128.61, 128.44, 127.42, 127.39, 127.31, 123.94, 123.78, 122.15, 118.22, 113.13, 33.50, 28.58; **HRMS (ESI, m/z)** calcd for $C_{35}H_{26}O_5$ $[M+Na]^+$ = 549.1678, found = 549.1674.

(*E*)-4-(2-(2-hydroxynaphthalen-1-yl)-3-oxoprop-1-en-1-yl)phenyl

2-(3-cyano-4-isobutoxyphenyl)thiazole-5-carboxylate (1ag)

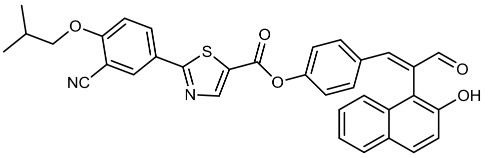

1ag

A yellow solid, 52% yield, 599 mg; m.p: 111-112 °C; **^{1}H NMR (400 MHz, CDCl$_3$)** δ 9.88 (s, 1H), 8.17 (d, *J* = 2.3 Hz, 1H), 8.08 (dd, *J* = 8.9, 2.3 Hz, 1H), 7.89 – 7.76 (m, 3H), 7.48 – 7.39 (m, 1H), 7.39 – 7.29 (m, 2H), 7.23 (d, *J* = 8.8 Hz, 2H), 7.15 (d, *J* = 8.9 Hz, 1H), 7.01 (dd, *J* = 8.9, 3.4 Hz, 3H), 5.85 (s, 1H), 3.89 (d, *J* = 6.5 Hz, 2H), 2.75 (s, 3H), 2.19 (dt, *J* = 13.3, 6.7 Hz, 1H), 1.08 (d, *J* = 6.7 Hz, 7H); **^{13}C NMR (100 MHz, CDCl$_3$)** δ 194.16, 168.63, 163.55, 162.89, 159.93, 152.06, 151.53, 150.94, 135.53, 132.83, 132.38, 132.19, 132.08, 131.89, 130.89, 129.37, 128.64, 127.37, 125.77, 123.98, 123.76, 122.14, 120.22, 118.24, 115.41, 113.17, 112.85, 103.19, 29.83, 28.27, 19.16, 17.81; **HRMS (ESI, m/z)** calcd for $C_{35}H_{28}N_2O_5S$ $[M+Na]^+$ = 611.1617, found = 611.1617.

(*E*)-4-(2-(2-hydroxynaphthalen-1-yl)-3-oxoprop-1-en-1-yl)phenyl 4-(4,5-diphenyloxazol-2-yl)butanoate (1ah)

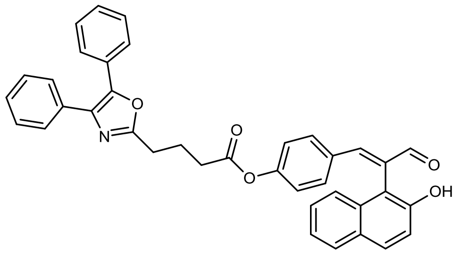

1ah

A yellow solid, 46% yield, 533 mg; m.p: 85-87 °C; **^{1}H NMR (400 MHz, CDCl$_3$)** δ 9.86 (s, 1H), 7.84 – 7.77 (m, 3H), 7.63 – 7.57 (m, 2H), 7.56 – 7.50 (m, 2H), 7.45 – 7.35 (m, 2H), 7.35 – 7.27 (m, 9H), 7.15 (t, *J* = 9.3 Hz, 3H), 6.92 – 6.87 (m, 2H), 5.80 (s, 1H), 3.22 (t, *J* = 7.2 Hz, 2H), 3.07 (t, *J* = 7.2 Hz, 2H); **^{13}C NMR (100 MHz, CDCl$_3$)** δ 194.06, 170.28, 161.45, 152.54, 151.65, 150.88, 145.76, 135.23, 135.20, 132.33, 132.20, 132.03, 131.56, 130.86, 129.36, 128.91, 128.78, 128.71, 128.62, 128.29, 127.99, 127.35, 126.63, 123.98, 123.79, 122.13, 118.21, 113.19, 31.31, 23.51; **HRMS (ESI, m/z)** calcd. for $C_{38}H_{29}NO_5$ $[M+Na]^+$ = 602.1943, found = 602.1947.

4-((*E*)-2-(2-hydroxynaphthalen-1-yl)-3-oxoprop-1-en-1-yl)phenyl (9*Z*,12*Z*)-octadeca-9,12-dienoate (1ai)

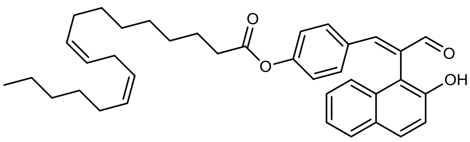

1ai

A yellow oil, 78% yield, 862 mg; **^{1}H NMR (400 MHz, CDCl$_3$)** δ 9.84 (s, 1H), 7.87 – 7.74 (m, 3H), 7.44 – 7.38 (m, 1H), 7.37 – 7.29 (m, 2H), 7.17 (d, *J* = 8.7 Hz, 2H), 7.13 (d, *J* = 8.9 Hz, 1H), 6.87 (d, *J* = 8.7 Hz, 2H), 5.66 (s, 1H), 5.36 (dq, *J* = 11.8, 6.2 Hz, 4H), 2.77 (t, *J* = 6.2 Hz, 2H), 2.48 (t, *J* = 7.5 Hz, 2H), 2.04 (q, *J* = 6.8 Hz, 4H), 1.67 (p, *J* = 7.2 Hz, 3H), 1.32 (dd, *J* = 15.9, 6.2 Hz, 13H), 0.88 (t, *J* = 6.7 Hz, 3H); **^{13}C NMR (100 MHz, CDCl$_3$)** δ 194.35, 171.95, 152.72, 152.13, 150.91, 135.07, 132.17, 132.08, 131.36, 130.83, 130.36, 130.11, 129.34, 128.61, 128.21, 128.01, 127.32, 123.95, 123.78, 122.16, 118.23, 113.10, 34.43, 31.65, 29.68, 29.47, 29.23, 29.18, 29.11, 27.33, 27.28, 25.75, 24.91, 22.70, 14.22; **HRMS (ESI, m/z)** calcd for $C_{37}H_{44}O_4$ $[M+Na]^+$ = 575.3137, found = 575.3135.

(*E*)-4-(2-(2-hydroxynaphthalen-1-yl)-3-oxoprop-1-en-1-yl)phenyl 2-(1-(4-chlorobenzoyl)-5-methoxy-2-methyl-1H-indol-3-yl)acetate (1aj)

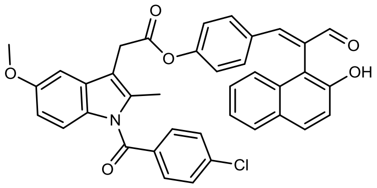

1aj

A yellow solid, 56% yield, 705 mg; m.p: 106-107 ^{o}C; **^{1}H NMR (400 MHz, CDCl$_3$)** δ 9.83 (s, 1H), 7.83 – 7.75 (m, 3H), 7.63 (d, *J* = 8.4 Hz, 2H), 7.44 (d, *J* = 8.4 Hz, 2H), 7.42 – 7.36 (m, 1H), 7.35 – 7.28 (m, 2H), 7.17 – 7.09 (m, 3H), 6.96 (d, *J* = 2.3 Hz, 1H), 6.85 (d, *J* = 8.9 Hz, 3H), 6.66 (dd, *J* = 9.0, 2.4 Hz, 1H), 5.69 (s, 1H), 3.82 (s, 2H), 3.78 (s, 3H), 2.39 (s, 3H); **^{13}C NMR (100 MHz, CDCl$_3$)** δ 194.17, 168.92, 168.43, 156.22, 152.54, 151.67, 150.86, 139.53, 136.42, 135.31, 133.82, 132.14, 132.00, 131.62, 131.31, 130.93, 130.86, 130.46, 129.34, 129.28, 128.62, 127.33, 123.96, 123.73, 121.96, 118.20, 115.15, 113.10, 111.88, 111.70, 101.29, 55.85, 30.63, 13.46; **HRMS (ESI, m/z)** calcd for $C_{38}H_{28}ClNO_6$ $[M+Na]^+$ = 652.1503, found = 652.1500.

(*E*)-4-(2-(2-hydroxynaphthalen-1-yl)-3-oxoprop-1-en-1-yl)phenyl 3-(4-(4-chlorobenzoyl)phenyl)-2,2-dimethylpropanoate (1ak)

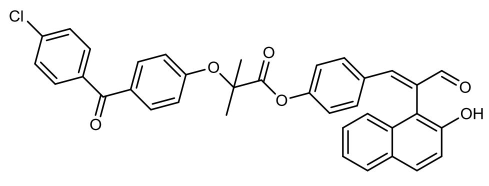

1ak

A yellow oil, 71% yield, 836 mg; **^{1}H NMR (400 MHz, CDCl$_3$)** δ 9.83 (s, 1H), 7.83 – 7.76 (m, 3H), 7.73 (d, *J* = 8.7 Hz, 2H), 7.67 (d, *J* = 8.4 Hz, 2H), 7.43 (d, *J* = 8.4 Hz, 2H), 7.41 – 7.36 (m, 1H), 7.34 – 7.29 (m, 2H), 7.20 – 7.10 (m, 3H), 6.90 (d, *J* = 8.7 Hz, 2H), 6.79 (d, *J* = 8.7 Hz, 2H), 5.92 (s, 1H), 1.75 (s, 6H); **^{13}C NMR (100 MHz, CDCl$_3$)** δ 194.45, 194.27, 172.15, 159.47, 152.12, 151.50, 150.94, 138.67, 136.27, 135.61, 132.30, 132.12, 132.07, 132.03, 131.32, 130.88, 130.79, 129.29, 128.72, 128.64, 127.34, 123.95, 123.67, 121.75, 118.23, 117.30, 112.99, 25.46, 25.44; **HRMS (ESI, m/z)** calcd for $C_{36}H_{27}ClO_6$ $[M+Na]^+$ = 613.1394, found = 613.1392.

(*E*)-4-(2-(2-hydroxynaphthalen-1-yl)-3-oxoprop-1-en-1-yl)phenyl 2-(11-oxo-6,11-dihydrodibenzo[b,e]oxepin-2-yl)acetate (1al)

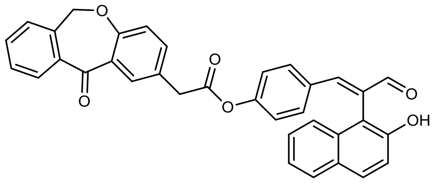

1al

A yellow solid, 61% yield, 659 mg; m.p: 88-90 ^{o}C; **^{1}H NMR (400 MHz, CDCl$_3$)** δ 9.84 (s, 1H), 8.15 (d, *J* = 2.3 Hz, 1H), 7.87 (dd, *J* = 7.7, 1.1 Hz, 1H), 7.81 – 7.77 (m, 3H), 7.55 (td, *J* = 7.5, 1.3 Hz, 1H), 7.47 (dd, *J* = 7.6, 1.2 Hz, 1H), 7.45 – 7.41 (m, 1H), 7.39 (d, *J* = 5.4 Hz, 1H), 7.37 – 7.34 (m, 1H), 7.33 – 7.30 (m, 2H), 7.17 – 7.10 (m, 3H), 7.02 (d, *J* = 8.4 Hz, 1H), 6.89 – 6.85 (m, 2H), 5.69 (s, 1H), 5.17 (s, 2H), 3.80 (s, 2H); **^{13}C NMR (100 MHz, CDCl$_3$)** δ 194.20, 190.95, 169.47, 160.80, 152.53, 151.80, 150.87, 140.44, 136.30, 135.60, 135.24, 133.01, 132.67, 132.14, 132.02, 131.58, 130.84, 129.62, 129.44, 129.33, 128.60, 127.98, 127.31, 126.92, 125.34, 123.94, 123.75, 122.01, 121.43, 118.22, 113.10, 40.35; **HRMS (ESI, m/z)** calcd for $C_{35}H_{24}O_6$ $[M+Na]^+$ = 563.1471, found = 563.1469.

4. Preparation of bifunctional phosphonium salts

All the phosphonium salt catalysts in our studies were listed in Supplementary Figure 2, which were prepared by following our previously reported procedures.[2]

P1-P5 were known compounds. The unknown compounds **P6-P10**, **P10-1**, **P10-2** and **P10-3** were fully characterized.

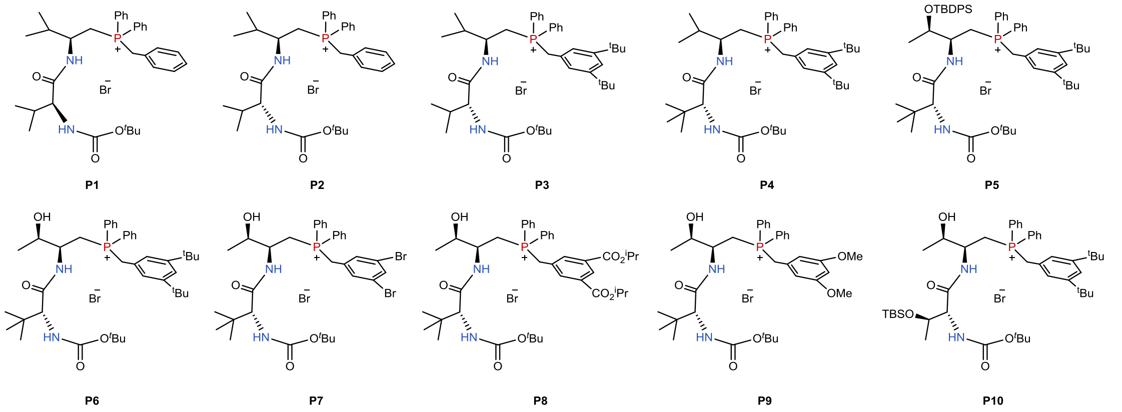

Supplementary Figure 1. Bifunctional phosphonium salt catalysts in this study.

((2*S*,3*R*)-2-((*R*)-2-((*tert*-butoxycarbonyl)amino)-3,3-dimethylbutanamido)-3-hydroxybutyl)(3,5-di-*tert*-butylbenzyl)diphenylphosphonium bromide (P6)

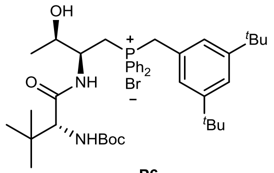

P6

A white solid; **^{1}H NMR (400 MHz, CDCl$_3$)** δ 8.24 (d, *J* = 8.6 Hz, 1H), 7.81 (dd, *J* = 12.4, 7.9 Hz, 2H), 7.72 – 7.48 (m, 6H), 7.40 (dt, *J* = 8.1, 4.0 Hz, 2H), 7.11 (d, *J* = 2.2 Hz, 1H), 6.66 (t, *J* = 2.2 Hz, 2H), 5.13 – 4.60 (m, 4H), 4.40 – 4.26 (m, 1H), 4.14 (t, *J* = 10.8 Hz, 1H), 3.87 (q, *J* = 7.5, 6.8 Hz, 2H), 3.33 (t, *J* = 14.7 Hz, 1H), 1.36 (s, 9H), 1.13 (d, *J* = 6.4 Hz, 3H), 1.00 (s, 18H), 0.97 (s, 9H); **^{13}C NMR (100 MHz, CDCl$_3$)** δ 171.31, 155.50, 151.27, 134.63, 134.25, 134.09, 134.00, 133.89, 133.81, 129.81 (d, *J* = 12.2 Hz), 129.51 (d, *J* = 12.4 Hz), 127.01, 125.11, 125.05, 121.69, 117.70 (d, *J* = 84.3 Hz), 117.57 (d, *J* = 82.4 Hz), 79.16, 69.01 (d, *J* = 12.4 Hz), 62.51, 49.70, 34.59, 34.17, 31.18, 30.37 (d, *J* = 43.5 Hz), 28.40, 27.16, 25.14 (d, *J* = 49.7 Hz), 19.65; **^{31}P NMR (162 MHz, CDCl$_3$)** δ 26.76; **HRMS (ESI, m/z)** calcd for $C_{42}H_{62}BrN_2O_4P$ [M-Br]$^+$ = 689.4442, found = 689.4441.

((2*S*,3*R*)-2-((*R*)-2-((*tert*-butoxycarbonyl)amino)-3,3-dimethylbutanamido)-3-hydroxybutyl)(3,5-dibromobenzyl)diphenylphosphonium bromide (P7)

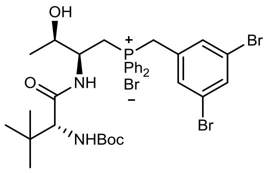

P7

A white solid; **^{1}H NMR (400 MHz, $CDCl_3$)** δ 8.55 (d, *J* = 8.9 Hz, 1H), 7.92 – 7.83 (m, 2H), 7.77 (td, *J* = 7.6, 1.8 Hz, 1H), 7.70 (td, *J* = 7.3, 1.6 Hz, 1H), 7.67 – 7.59 (m, 4H), 7.52 (td, *J* = 7.8, 3.4 Hz, 2H), 7.38 (q, *J* = 1.9 Hz, 1H), 6.94 (dd, *J* = 2.7, 1.8 Hz, 2H), 5.40 (dt, *J* = 20.4, 9.6 Hz, 2H), 5.02 (d, *J* = 7.3 Hz, 1H), 4.71 (q, *J* = 12.1 Hz, 2H), 4.05 – 3.87 (m, 3H), 3.10 (t, *J* = 14.6 Hz, 1H), 1.50 (s, 9H), 1.21 (d, *J* = 6.4 Hz, 3H), 1.06 (s, 9H); **^{13}C NMR (100 MHz, $CDCl_3$)** δ 171.33, 156.28, 135.08 (d, *J* = 37.1 Hz), 134.14, 134.06, 133.97, 133.32, 132.68, 130.39, 130.26, 129.84, 129.72, 122.66, 117.14 (d, *J* = 84.8 Hz), 116.08 (d, *J* = 81.9 Hz), 79.76, 69.56 (d, *J* = 13.5 Hz), 63.09, 49.98, 33.52, 28.88 (d, *J* = 43.1 Hz), 28.57, 27.38, 26.77 (d, *J* = 49.3 Hz), 19.89, 14.26; **^{31}P NMR (162 MHz, $CDCl_3$)** δ 29.64; **HRMS (ESI, m/z)** calcd for $C_{34}H_{44}Br_3N_2O_4P$ $[M-Br]^+$ = 733.1400, found = 733.1404.

(3,5-bis(isopropoxycarbonyl)benzyl)((2*S*,3*R*)-2-((*R*)-2-((*tert*-butoxycarbonyl)amino)-3,3-dimethylbutanamido)-3-hydroxybutyl)diphenylphosphonium bromide (P8)

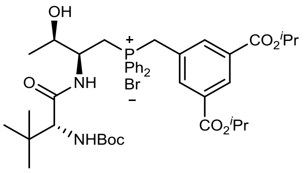

P8

A white solid; **^{1}H NMR (400 MHz, $CDCl_3$)** δ 8.50 (d, *J* = 8.8 Hz, 1H), 8.38 (s, 1H), 7.90 – 7.82 (m, 2H), 7.77 – 7.56 (m, 8H), 7.53 – 7.44 (m, 2H), 5.39 – 5.21 (m, 1H), 5.09 (dq, *J* = 12.5, 6.3 Hz, 4H), 4.65 – 3.99 (m, 3H), 3.97 – 3.83 (m, 2H), 3.39 (t, *J* = 14.7 Hz, 1H), 1.39 (s, 9H), 1.24 (d, *J* = 6.3 Hz, 12H), 1.16 (d, *J* = 6.4 Hz, 3H), 1.00 (s, 9H); **^{13}C NMR (100 MHz, $CDCl_3$)** δ 171.48, 164.37, 155.80, 135.36 (d, *J* = 33.6 Hz), 134.87, 134.02 (d, *J* = 9.4 Hz), 131.68, 130.25, 129.88, 129.75, 116.95 (d, *J* = 84.6 Hz), 115.98 (d, *J* = 82.5 Hz), 79.61, 69.11, 62.66, 49.69, 34.02, 29.61 (d, *J* = 46.4 Hz), 28.44, 27.08, 25.42 (d, *J* = 48.8 Hz), 21.87 (d, *J* = 2.5 Hz), 19.30; **^{31}P NMR (162 MHz, $CDCl_3$)** δ 29.64; **HRMS (ESI, m/z)** calcd for $C_{42}H_{58}BrN_2O_8P$ $[M-Br]^+$ = 749.3925, found = 749.3922.

((2*S*,3*R*)-2-((*R*)-2-((*tert*-butoxycarbonyl)amino)-3,3-dimethylbutanamido)-3-hydroxybutyl)(3,5-dimethoxybenzyl)diphenylphosphonium bromide (P9)

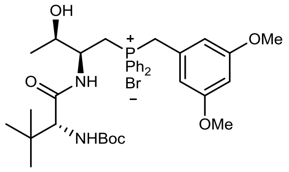

P9

A white solid; **^{1}H NMR (400 MHz, CDCl$_3$)** δ 8.18 (d, *J* = 7.9 Hz, 1H), 7.86 – 7.80 (m, 2H), 7.77 – 7.63 (m, 4H), 7.62 – 7.48 (m, 4H), 6.19 (q, *J* = 2.3 Hz, 1H), 6.08 (t, *J* = 2.4 Hz, 2H), 5.10 (d, *J* = 8.2 Hz, 1H), 4.85 (t, *J* = 14.5 Hz, 1H), 4.69 (t, *J* = 14.8 Hz, 2H), 4.07 (q, *J* = 10.7, 10.1 Hz, 2H), 3.90 – 3.73 (m, 2H), 3.46 (s, 6H), 3.42 (s, 1H), 1.37 (s, 9H), 1.10 (d, *J* = 6.4 Hz, 3H), 0.95 (s, 9H); **^{13}C NMR (100 MHz, CDCl$_3$)** δ 171.38, 160.73, 155.59, 134.78 (d, *J* = 22.6 Hz), 134.12, 134.03, 133.94, 130.05, 129.92, 129.82, 129.70, 117.65 (d, *J* = 84.6 Hz), 117.17 (d, *J* = 82.7 Hz), 108.56, 100.93, 79.54, 68.63 (d, *J* = 11.0 Hz), 62.45, 55.38, 49.58, 34.34, 30.18 (d, *J* = 44.7 Hz), 28.38, 26.98, 24.60 (d, *J* = 48.4 Hz), 19.26; **^{31}P NMR (162 MHz, CDCl$_3$)** δ 26.43; **HRMS (ESI, m/z)** calcd for $C_{36}H_{50}BrN_2O_6P$ $[M-Br]^+$ = 637.3401, found = 637.3406.

(4*S*,7*R*,8*R*)-7-((*tert*-butoxycarbonyl)amino)-1-(3,5-di-*tert*-butylphenyl)-4-((*R*)-1-hydroxyethyl)-8,10,10,11,11-pentamethyl-6-oxo-2,2-diphenyl-9-oxa-5-aza-2-phospha-10-siladodecan-2-ium bromide (P10)

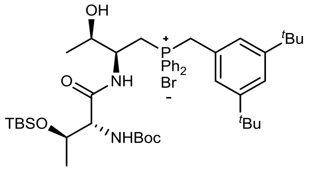

P10

A white solid; **^{1}H NMR (400 MHz, CDCl$_3$)** δ 7.94 (d, *J* = 8.7 Hz, 1H), 7.87 – 7.80 (m, 2H), 7.73 – 7.61 (m, 4H), 7.61 – 7.45 (m, 4H), 7.19 (q, *J* = 2.0 Hz, 1H), 6.70 (t, *J* = 2.1 Hz, 2H), 5.27 (d, *J* = 6.4 Hz, 1H), 4.84 (t, *J* = 14.5 Hz, 1H), 4.75 (d, *J* = 6.3 Hz, 1H), 4.43 (t, *J* = 14.5 Hz, 1H), 4.33 – 4.07 (m, 3H), 3.84 (s, 1H), 3.73 (dd, *J* = 6.5, 3.2 Hz, 1H), 3.35 (t, *J* = 14.3 Hz, 1H), 1.44 (s, 9H), 1.18 (d, *J* = 6.5 Hz, 3H), 1.08 (s, 3H), 1.05 (s, 18H), 0.81 (s, 9H), 0.04 (d, *J* = 3.5 Hz, 3H), 0.00 (s, 3H); **^{13}C NMR (100 MHz, CDCl$_3$)** δ 169.09, 155.20, 151.52, 134.55 (d, *J* = 10.4 Hz), 134.55 (d, *J* = 5.3 Hz), 134.07, 133.97, 133.81, 133.72, 129.89, 129.76, 129.62, 126.50, 124.94 (d, *J* = 5.6 Hz), 122.08, 117.91 (d, *J* = 83.0 Hz), 117.65 (d, *J* = 83.0 Hz), 79.26, 69.27 (d, *J* = 12.3 Hz), 68.84, 59.76, 49.24, 34.66, 31.19, 30.46 (d, *J* = 44.5 Hz), 28.47, 25.74,

25.29 (d, *J* = 51.5 Hz), 20.48, 19.76, 17.94, -4.67, -4.78; **^{31}P NMR (162 MHz, CDCl$_3$)** δ 24.88; **HRMS (ESI, m/z)** calcd for $C_{46}H_{72}BrN_2O_5PSi$ [M-Br]$^+$ = 791.4943, found = 791.4941.

(4*S*,7*R*,8*R*)-7-((*tert*-butoxycarbonyl)amino)-1-(3,5-di-*tert*-butylphenyl)-4-((*R*)-1-(2,4-dinitrophenoxy)ethyl)-8,10,10,11,11-pentamethyl-6-oxo-2,2-diphenyl-9-oxa-5-aza-2-phospha-10-siladodecan-2-ium bromide (P10-1)

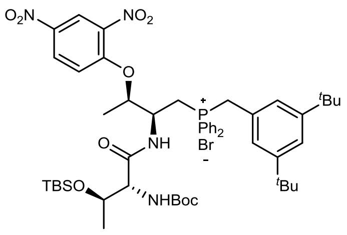

P10-1

A yellow solid; **^{1}H NMR (400 MHz, CDCl$_3$)** δ 9.56 (s, 1H), 8.53 (d, *J* = 2.8 Hz, 1H), 8.28 (dd, *J* = 9.2, 2.8 Hz, 1H), 7.85 – 7.77 (m, 2H), 7.67 (t, *J* = 7.5 Hz, 1H), 7.54 – 7.33 (m, 8H), 7.16 (s, 1H), 6.68 (t, *J* = 2.3 Hz, 2H), 5.35 (d, *J* = 6.0 Hz, 1H), 5.14 – 4.77 (m, 4H), 4.41 (s, 1H), 4.24 (d, *J* = 5.9 Hz, 1H), 4.09 (d, *J* = 5.8 Hz, 1H), 2.91 (t, *J* = 14.9 Hz, 1H), 1.60 (d, *J* = 6.3 Hz, 3H), 1.48 (s, 9H), 1.29 (d, *J* = 6.4 Hz, 3H), 1.02 (s, 18H), 0.87 (s, 9H), 0.13 (s, 3H), 0.10 (s, 3H); **^{13}C NMR (100 MHz, CDCl$_3$)** δ 170.25, 155.98, 154.31, 151.52, 140.33, 139.22, 134.73 (d, *J* = 14.5 Hz), 134.08 (d, *J* = 9.2 Hz), 133.48 (d, *J* = 8.8 Hz), 129.98, 129.85, 129.52 (d, *J* = 40.8 Hz), 125.19 (d, *J* = 5.5 Hz), 122.13, 121.42, 118.06, 117.03, 116.11, 79.56, 75.79 (d, *J* = 16.9 Hz), 68.15, 59.70, 46.00, 34.72, 31.25, 28.55, 25.92, 20.81, 18.11, 14.10, -4.40, -4.54; **^{31}P NMR (162 MHz, CDCl$_3$)** δ 28.34; **HRMS (ESI, m/z)** calcd for $C_{52}H_{74}BrN_4O_9PSi$ [M-Br]$^+$ = 957.4957, found = 957.4958.

(4*S*,7*R*,8*R*)-7-((*tert*-butoxycarbonyl)amino)-1-(3,5-di-*tert*-butylphenyl)-4-((*R*)-1-hydroxyethyl)-5,8,10,10,11,11-hexamethyl-6-oxo-2,2-diphenyl-9-oxa-5-aza-2-phospha-10-siladodecan-2-ium bromide (P10-2)

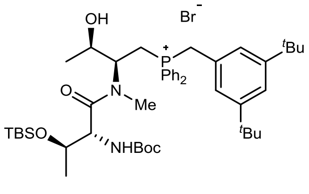

P10-2

A white solid; **^{1}H NMR (400 MHz, CDCl$_3$)** δ 7.87 – 7.64 (m, 6H), 7.62 – 7.51 (m, 4H), 7.23 (d, *J* = 2.0 Hz, 1H), 6.76 (dd, *J* = 2.6, 1.7 Hz, 2H), 5.20 (dd, *J* = 19.6, 7.2 Hz, 2H), 5.05 – 4.90 (m, 1H), 4.83 (t, *J* = 14.5 Hz, 1H), 4.25 (dd, *J* = 8.6, 3.6 Hz, 1H),

4.10 (t, J = 14.5 Hz, 1H), 4.03 – 3.89 (m, 3H), 3.63 (dt, J = 16.3, 10.6 Hz, 1H), 2.79 (s, 3H), 1.45 (s, 9H), 1.08 (s, 18H), 0.97 (dd, J = 13.2, 6.3 Hz, 6H), 0.81 (s, 9H), -0.01 (s, 6H); **^{13}C NMR (100 MHz, CDCl$_3$)** δ 171.71, 155.11, 151.88, 151.85, 134.99, 134.77, 133.70 (d, J = 43.3 Hz), 133.61 (d, J = 42.9 Hz), 130.11, 130.09, 129.99, 129.97, 125.63, 125.55, 124.87, 124.81, 122.55, 118.25 (d, J = 84.8 Hz), 118.11 (d, J = 81.1 Hz), 79.44, 69.26 (d, J = 12.6 Hz), 56.55, 51.88, 34.78, 31.93, 31.25, 30.00 (d, J = 46.5 Hz), 28.54, 25.78, 22.31 (d, J = 51.4 Hz), 19.70, -4.62, -4.66; **^{31}P NMR (162 MHz, CDCl$_3$)** δ 24.94; **HRMS (ESI, m/z)** calcd for $C_{47}H_{74}BrN_2O_5PSi$ [M-Br]$^+$ = 805.5099, found = 805.5101.

(4*S*,7*R*,8*R*)-7-((*tert*-butoxycarbonyl)(methyl)amino)-1-(3,5-di-*tert*-butylphenyl)-4-((*R*)-1-hydroxyethyl)-8,10,10,11,11-pentamethyl-6-oxo-2,2-diphenyl-9-oxa-5-aza-2-phospha-10-siladodecan-2-ium bromide (P10-3)

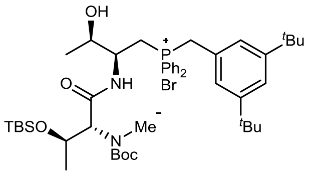

P10-3

A white solid; **^{1}H NMR (400 MHz, CDCl$_3$)** δ 8.00 – 7.86 (m, 2H), 7.86 – 7.68 (m, 4H), 7.65 – 7.34 (m, 5H), 7.22 (s, 1H), 6.80 (t, J = 2.2 Hz, 2H), 5.48 – 4.73 (m, 2H), 4.36 – 3.90 (m, 5H), 3.60 (dt, J = 19.3, 6.2 Hz, 1H), 3.09 (m, J = 57.6, 20.2, 7.6 Hz, 1H), 2.73 (d, J = 38.9 Hz, 3H), 2.24 – 1.95 (m, 1H), 1.43 (d, J = 29.3 Hz, 9H), 1.23 (d, J = 3.2 Hz, 1H), 1.08 (s, 18H), 0.90 (dd, J = 11.7, 6.6 Hz, 3H), 0.83 – 0.77 (m, 9H), 0.05 (s, 3H), -0.02 (s, 3H); **^{13}C NMR (100 MHz, CDCl$_3$)** δ 170.11, 169.79, 156.00, 155.11, 151.65, 134.72 (d, J = 16.4 Hz), 134.09, 133.74, 129.99 (d, J = 12.2 Hz), 125.77, 124.97 (d, J = 5.6 Hz), 122.24, 118.27 (d, J = 93.3 Hz), 81.23, 80.34, 67.81, 66.93, 66.58, 64.86 (d, J = 26.0 Hz), 49.72, 34.69, 32.84, 31.17, 30.95, 28.44, 28.34, 25.88, 20.62 (d, J = 12.3 Hz), 19.76, 17.99, -4.30, -4.45; **^{31}P NMR (162 MHz, CDCl$_3$)** δ 23.83, 23.47; **HRMS (ESI, m/z)** calcd for $C_{47}H_{74}BrN_2O_5PSi$ [M-Br]$^+$ = 805.5099, found = 805.5098.

5. Procedure for the synthesis of axially chiral aldehyde-containing styrenes.

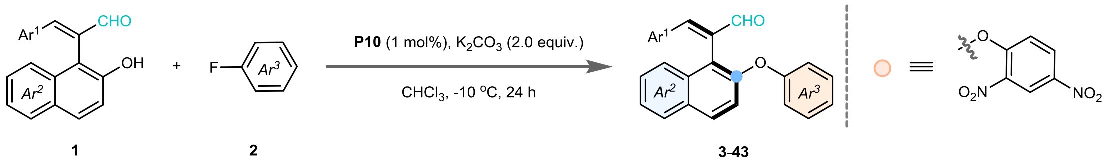

General procedures A: To a round bottle flask with a magnetic stirring bar were added **1** (0.10 mmol) and **2** (22.3 mg, 0.12 mmol), followed by the addition of K_2CO_3 (27.6 mg, 0.20 mmol) and catalyst **P10** (0.9 mg, 0.001 mol), followed by the addition of chloroform (1.0 mL). The reaction mixture was stirred at -10 ^{o}C for 24 h. Then, the solvent was removed under reduced pressure, and the residue was purified by column chromatography on silica gel (CH_2Cl_2/Petroleum ether = 5/1) to afford target product **3-43**.

(*S, E*)-2-(2-(2,4-dinitrophenoxy)naphthalen-1-yl)-3-phenylacrylaldehyde (3)

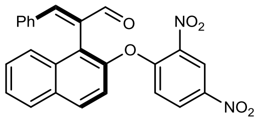

3

A yellow solid, 99% yield, 43.7 mg; m.p: 147-149 ^{o}C; $[\alpha]^{25}_{D}$ = -96.0 (c = 0.25 in $CHCl_3$); **^{1}H NMR (400 MHz, $CDCl_3$)** δ 9.78 (s, 1H), 8.71 (d, *J* = 2.8 Hz, 1H), 8.12 – 8.02 (m, 2H), 8.00 – 7.94 (m, 1H), 7.79 (s, 1H), 7.65 (dd, *J* = 8.3, 1.2 Hz, 1H), 7.59 – 7.47 (m, 2H), 7.28 (d, *J* = 9.0 Hz, 2H), 7.14 (dd, *J* = 8.6, 7.0 Hz, 2H), 7.09 – 7.03 (m, 2H), 6.95 (d, *J* = 9.3 Hz, 1H); **^{13}C NMR (100 MHz, $CDCl_3$)** δ 193.13, 155.41, 154.06, 148.18, 141.38, 139.21, 134.82, 133.65, 132.67, 131.96, 131.63, 131.16, 130.38, 128.89, 128.87, 128.51, 128.18, 126.84, 125.12, 123.43, 121.65, 119.81, 118.87; **HRMS (ESI, m/z)** calcd. for $C_{25}H_{16}N_2O_6$ $[M+Na]^+$ = 463.0906, found = 463.0901; **HPLC analysis**: 96% ee (ADH column, 25 ^{o}C, n-hexane/*i*-PrOH = 70/30, 1.0 mL/min, λ = 254 nm), Rt (major) = 22.2 min, Rt (minor) = 15.8 min.

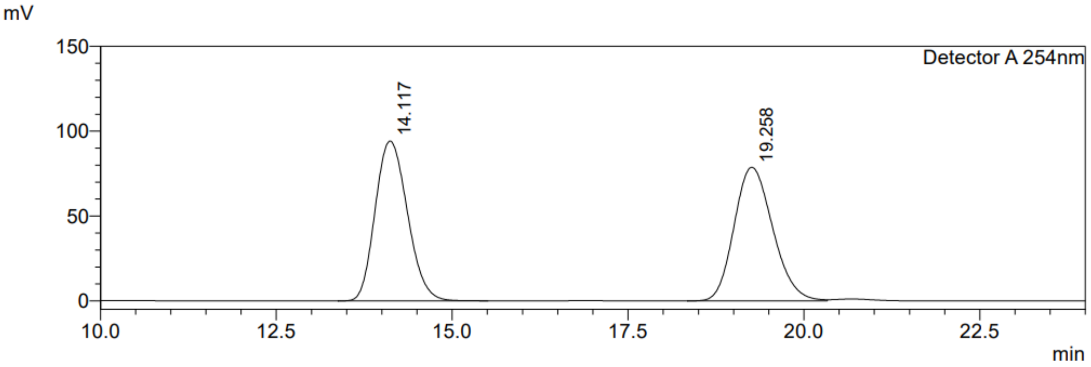

Detector A 254nm

Peak#	Ret. Time	Height	Height%	Area	Area%
1	14.117	94162	54.453	3004554	49.930
2	19.258	78762	45.547	3012966	50.070
Total		172924	100.000	6017520	100.000

Racemic **3**

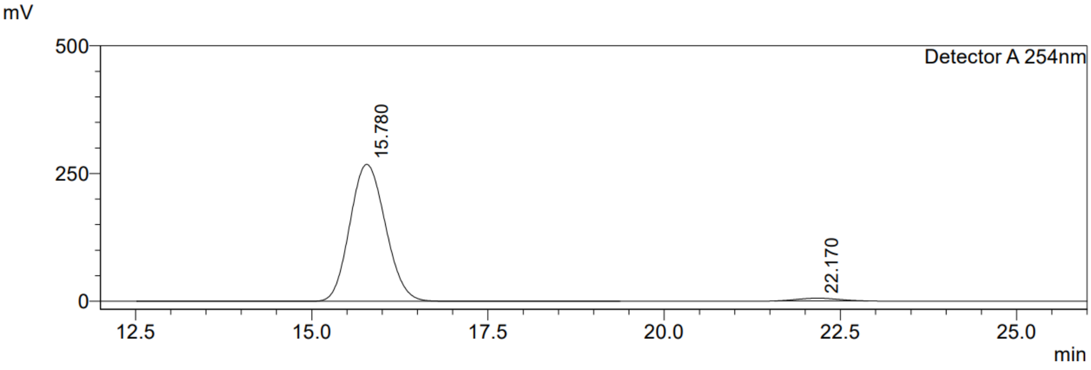

Detector A 254nm

Peak#	Ret. Time	Height	Height%	Area	Area%
1	15.780	267894	98.060	9416442	97.793
2	22.170	5299	1.940	212500	2.207
Total		273193	100.000	9628942	100.000

Enantiomerically enriched **3**

(*S*, *E*)-3-([1,1'-biphenyl]-2-yl)-2-(2-(2,4-dinitrophenoxy)naphthalen-1-yl)acrylaldehyde (4)

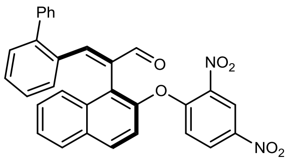

4

A yellow solid, 99% yield, 51.0 mg; m.p: 75-77 ^{o}C; **[α]$^{25}_{D}$** = +15.1 (c = 0.25 in $CHCl_3$).; **^{1}H NMR (400 MHz, $CDCl_3$)** δ 9.65 (s, 1H), 8.73 (d, *J* = 2.8 Hz, 1H), 8.08 (dd, *J* = 9.3, 2.8 Hz, 1H), 8.04 – 7.99 (m, 1H), 7.99 – 7.94 (m, 1H), 7.85 (s, 1H), 7.71 – 7.66 (m, 1H), 7.61 – 7.51 (m, 2H), 7.49 – 7.39 (m, 3H), 7.35 – 7.28 (m, 4H), 7.20 (d, *J* = 8.9 Hz, 1H), 6.97 – 6.93 (m, 2H), 6.82 (d, *J* = 9.3 Hz, 1H); **^{13}C NMR (100 MHz,**

CDCl$_3$) δ 193.32, 155.41, 153.42, 148.60, 143.95, 141.48, 139.68, 139.47, 135.15, 133.02, 131.84, 131.60, 130.67, 130.60, 129.89, 128.94, 128.92, 128.66, 128.49, 128.20, 128.18, 127.46, 126.76, 125.21, 123.28, 121.78, 119.35, 119.20; **HRMS (ESI, m/z)** calcd for $C_{31}H_{20}N_2O_6$ $[M+Na]^+$ = 539.1219, found = 539.1208; **HPLC analysis**: 94% ee (ADH column, 25 °C, n-hexane/*i*-PrOH = 70/30, 1.0 mL/min, λ = 254 nm), Rt (major) = 17.0 min, Rt (minor) = 29.1 min.

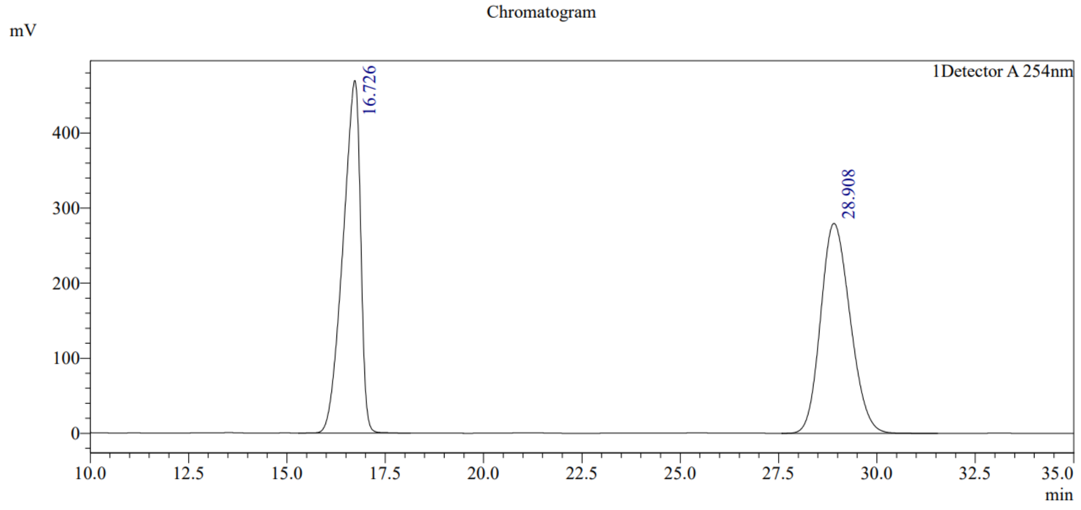

Peak Table

Detector A 254nm

Peak#	Ret. Time	Height	Height%	Area	Area%
1	16.726	469840	62.683	14607745	49.986
2	28.908	279714	37.317	14616049	50.014
Total		749554	100.000	29223794	100.000

Racemic **4**

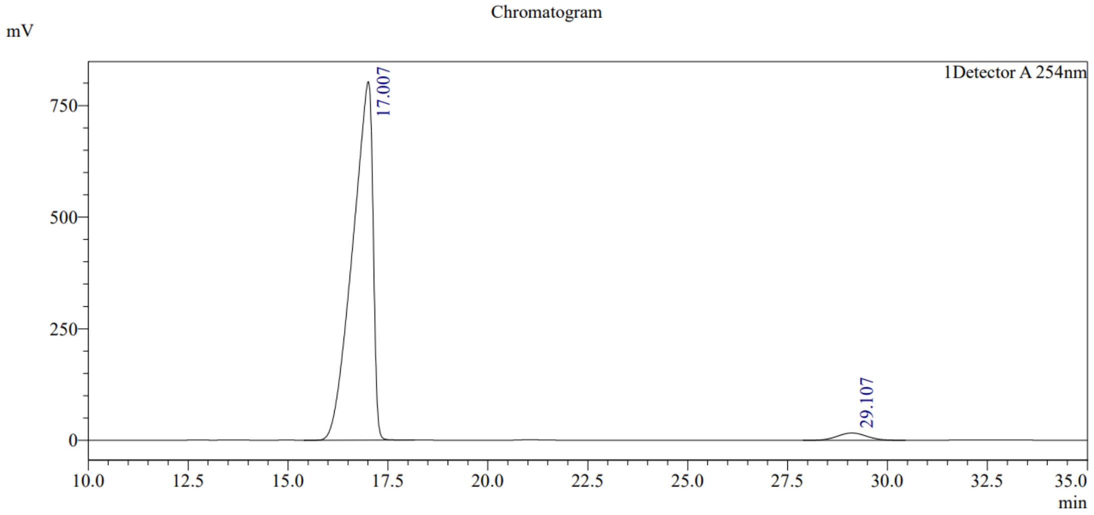

Peak Table

Detector A 254nm

Peak#	Ret. Time	Height	Height%	Area	Area%
1	17.007	803019	98.000	28515558	97.157
2	29.107	16390	2.000	834565	2.843
Total		819409	100.000	29350123	100.000

Enantiomerically enriched **4**

(*S*, *E*)-2-(2-(2,4-dinitrophenoxy)naphthalen-1-yl)-3-(2-methoxyphenyl)acrylaldehyde (5)

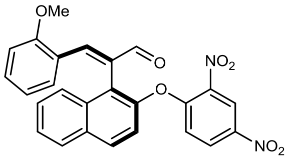

5

A yellow solid, 94% yield, 44.2 mg; m.p:195-196 °C; **[α]$^{25}_{D}$** = -74.5 (c = 0.25 in $CHCl_3$); **^{1}H NMR (400 MHz, $CDCl_3$)** δ 9.78 (s, 1H), 8.75 (d, *J* = 2.7 Hz, 1H), 8.22 (s, 1H), 8.08 – 7.93 (m, 3H), 7.70 – 7.61 (m, 1H), 7.59 – 7.48 (m, 2H), 7.27 – 7.20 (m, 2H), 6.85 (d, *J* = 9.3 Hz, 1H), 6.79 (d, *J* = 8.2 Hz, 1H), 6.64 (dd, *J* = 7.8, 1.6 Hz, 1H), 6.49 (t, *J* = 7.5 Hz, 1H), 3.83 (s, 3H); **^{13}C NMR (100 MHz, $CDCl_3$)** δ 193.38, 158.24, 155.72, 149.25, 147.90, 141.17, 138.81, 134.35, 133.04, 132.68, 131.97, 131.43, 129.34, 128.82, 128.51, 128.06, 126.76, 125.43, 123.95, 122.80, 121.80, 120.48, 119.98, 118.65, 110.82, 55.66; **HRMS (ESI, m/z)** calcd for $C_{26}H_{18}N_2O_7$ $[M+Na]^+$ = 493.1012, found = 493.1009; **HPLC analysis**: 95% ee (ADH column, 25 °C, n-hexane/*i*-PrOH = 80/20, 1.0 mL/min, λ = 254 nm), Rt (major) = 25.7 min, Rt (minor) = 47.6 min.

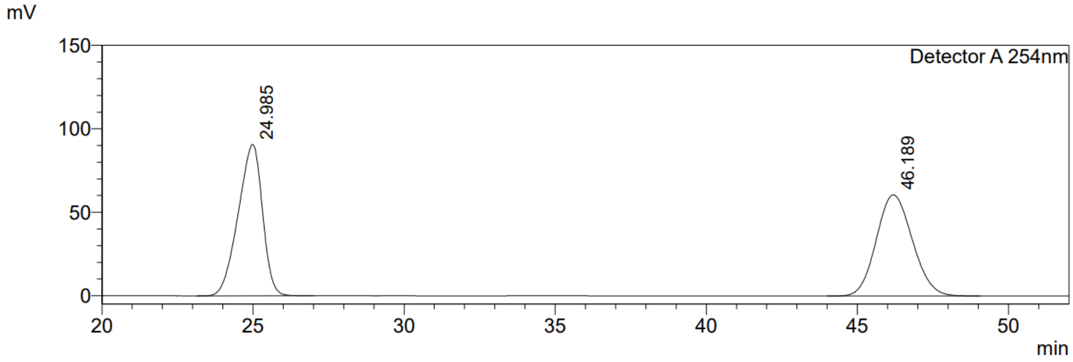

Detector A 254nm

Peak#	Ret. Time	Height	Height%	Area	Area%
1	24.985	90644	59.945	5072514	50.162
2	46.189	60568	40.055	5039768	49.838
Total		151212	100.000	10112281	100.000

Racemic **5**

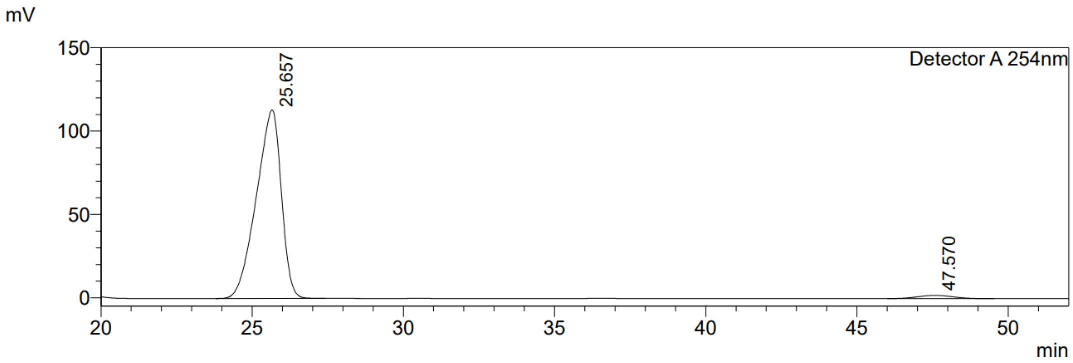

Detector A 254nm

Peak#	Ret. Time	Height	Height%	Area	Area%
1	25.657	113041	98.296	6499394	97.570
2	47.570	1960	1.704	161896	2.430
Total		115001	100.000	6661290	100.000

Enantiomerically enriched **5**

(*S*, *E*)-3-(2-bromophenyl)-2-(2-(2,4-dinitrophenoxy)naphthalen-1-yl)acrylaldehyde (6)

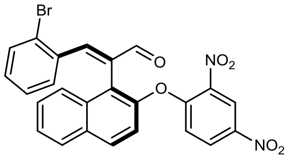

6

A yellow solid, 92% yield, 47.8 mg; m.p:173-175 °C; **$[\alpha]^{25}_D$** = -55.8 (c = 0.25 in $CHCl_3$); **^{1}H NMR (400 MHz, $CDCl_3$)** δ 9.90 (s, 1H), 8.82 (d, *J* = 2.7 Hz, 1H), 8.15 – 8.04 (m, 2H), 7.98 (d, *J* = 8.9 Hz, 1H), 7.93 (dt, *J* = 6.9, 3.9 Hz, 1H), 7.69 – 7.60 (m, 1H), 7.58 – 7.44 (m, 3H), 7.20 (d, *J* = 8.9 Hz, 1H), 7.11 – 7.03 (m, 1H), 6.92 – 6.84 (m, 3H); **^{13}C NMR (100 MHz, $CDCl_3$)** δ 192.87, 155.43, 152.48, 148.49, 141.48, 139.14, 136.91, 133.95, 133.17, 132.75, 131.75, 131.73, 131.60, 129.87, 128.87, 128.60, 128.26, 127.49, 126.76, 125.36, 125.00, 122.37, 122.14, 119.32, 118.75; **HRMS (ESI, m/z)** calcd for $C_{25}H_{15}BrN_2O_6$ $[M+Na]^+$ = 541.0011, found = 541.0011; **HPLC analysis**: 84% ee (ADH column, 25 °C, n-hexane/*i*-PrOH = 80/20, 1.0 mL/min, λ = 254 nm), Rt (major) = 35.8 min, Rt (minor) = 43.8 min.

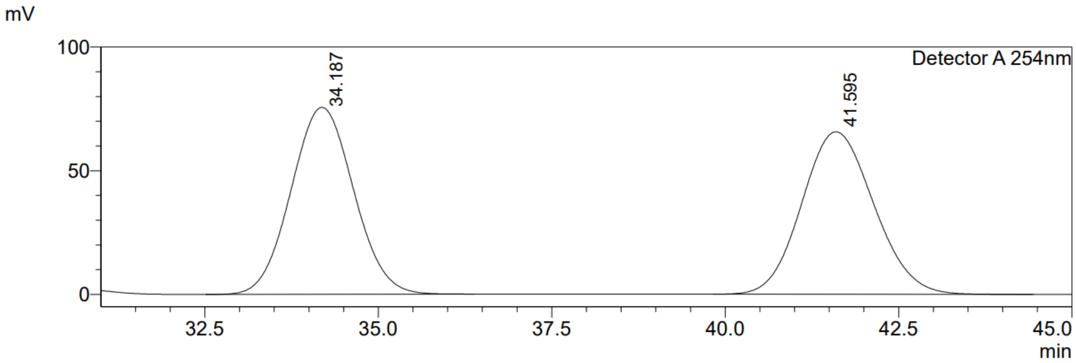

Detector A 254nm

Peak#	Ret. Time	Height	Height%	Area	Area%
1	34.187	75554	53.492	4753096	49.975
2	41.595	65688	46.508	4757914	50.025
Total		141242	100.000	9511011	100.000

Racemic **6**

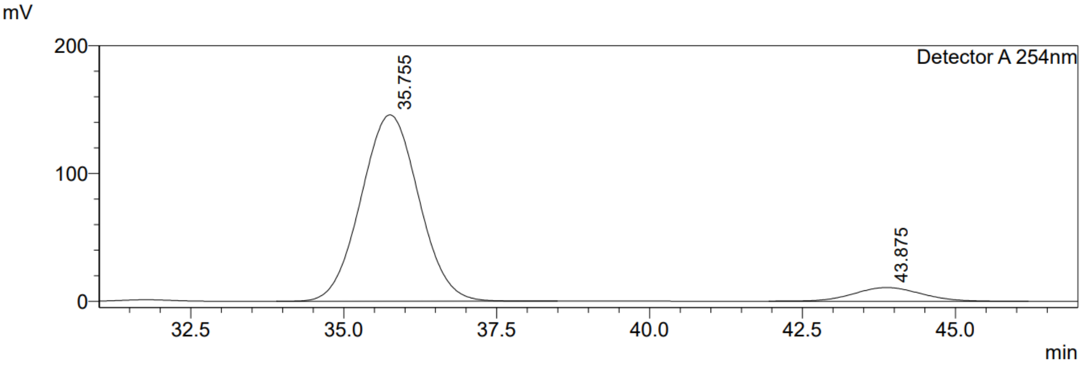

Detector A 254nm

Peak#	Ret. Time	Height	Height%	Area	Area%
1	35.755	145936	93.151	9608483	92.100
2	43.875	10731	6.849	824179	7.900
Total		156667	100.000	10432661	100.000

Enantiomerically enriched **6**

(*S*, *E*)-2-(2-(2,4-dinitrophenoxy)naphthalen-1-yl)-3-(2-fluorophenyl)acrylaldehyde (7)

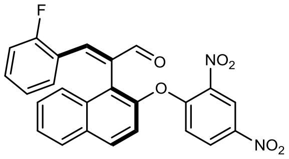

7

A yellow solid, 95% yield, 43.5 mg; m.p: 124-125 ^{o}C; **[α]^{25}$_{D}$** = -200.6 (c = 0.25 in CHCl$_3$); **^{1}H NMR (400 MHz, CDCl$_3$)** δ 9.82 (s, 1H), 8.77 (d, *J* = 2.8 Hz, 1H), 8.15 (dd, *J* = 9.3, 2.8 Hz, 1H), 8.09 (d, *J* = 0.8 Hz, 1H), 8.07 – 8.01 (m, 1H), 7.99 – 7.93

(m, 1H), 7.62 (m, J = 8.1, 1.6, 0.8 Hz, 1H), 7.59 – 7.48 (m, 2H), 7.30 – 7.26 (m, 1H), 7.25 (d, J = 3.6 Hz, 1H), 7.08 – 7.03 (m, 1H), 7.00 (d, J = 9.3 Hz, 1H), 6.76 – 6.69 (m, 2H); **^{13}C NMR (100 MHz, $CDCl_3$)** δ 193.04, 161.24 (d, J = 254.3 Hz), 155.39, 148.30, 145.38 (d, J = 6.5 Hz), 141.55, 139.25, 136.12, 133.04 (d, J = 9.1 Hz), 132.49, 131.93, 131.85, 129.32 (d, J = 1.6 Hz), 128.90, 128.68, 128.27, 126.88, 125.02, 124.41 (d, J = 3.7 Hz), 122.97, 121.99, 121.89, 119.60, 118.94, 115.98 (d, J = 21.9 Hz); **^{19}F NMR (376 MHz, $CDCl_3$)** δ -113.39; HRMS (ESI, m/z) calcd for $C_{25}H_{15}FN_2O_6$ $[M+Na]^+$ = 481.0812, found = 481.0808; **HPLC analysis**: 90% ee (ADH column, 25 ^{o}C, n-hexane/*i*-PrOH = 70/30, 1.0 mL/min, λ = 254 nm), Rt (major) = 20.3 min, Rt (minor) = 23.7 min.

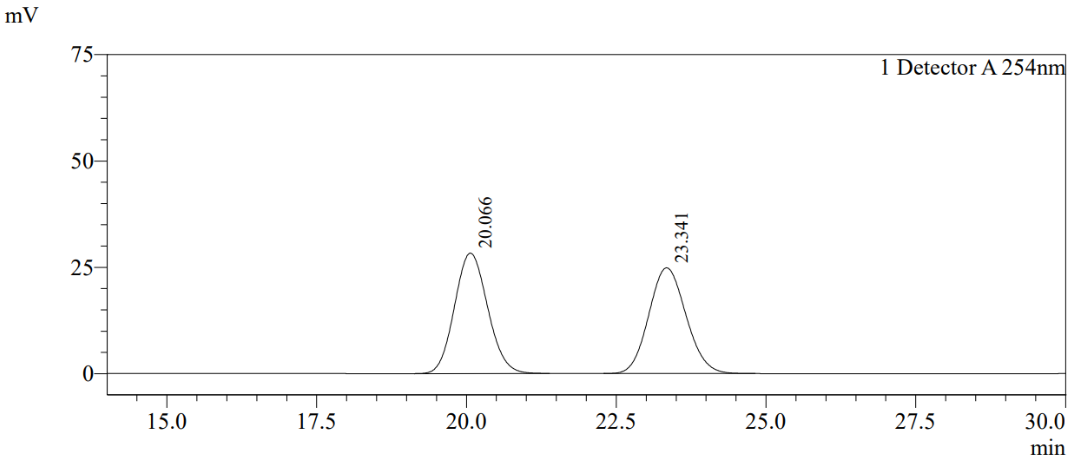

Peak Table

Detector A 254nm

Peak#	Ret. Time	Height	Height%	Area	Area%
1	20.066	28307	53.243	1091032	50.326
2	23.341	24859	46.757	1076880	49.674
Total		53166	100.000	2167912	100.000

Racemic **7**

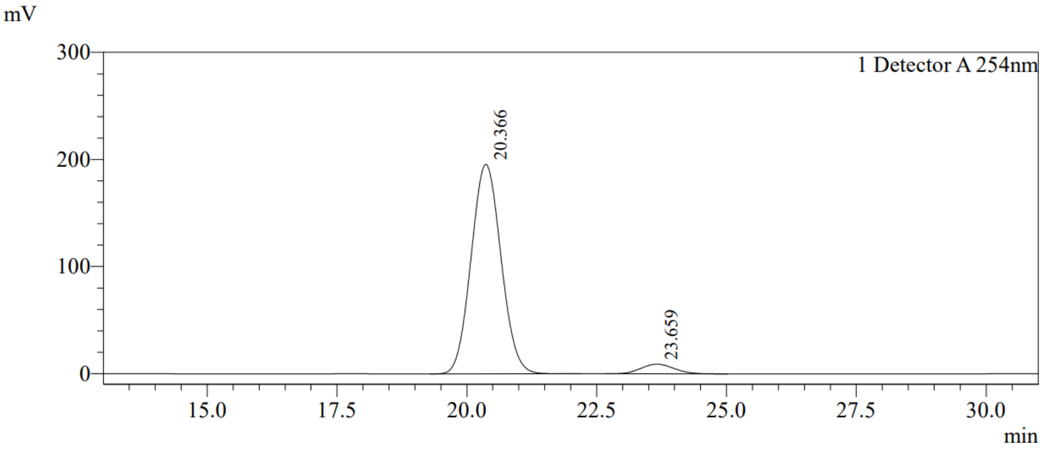

Peak Table

Detector A 254nm

Peak#	Ret. Time	Height	Height%	Area	Area%
1	20.366	195634	95.601	7844755	95.135
2	23.659	9002	4.399	401173	4.865
Total		204637	100.000	8245928	100.000

Enantiomerically enriched **7**

(*S*, *E*)-2-(2-(2,4-dinitrophenoxy)naphthalen-1-yl)-3-(2-nitrophenyl)acrylaldehyde (8)

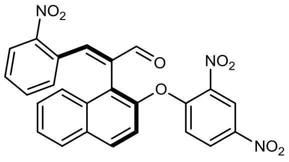

8

A yellow solid, 93% yield, 45.1mg; m.p: 96-97 °C; $[\alpha]^{25}_{D}$ = -269.4 (c = 0.25 in $CHCl_3$); **^{1}H NMR (400 MHz, $CDCl_3$)** δ 9.94 (s, 1H), 8.88 (d, *J* = 2.8 Hz, 1H), 8.33 (s, 1H), 8.29 (dd, *J* = 9.3, 2.8 Hz, 1H), 8.11 (dd, *J* = 8.0, 1.5 Hz, 1H), 7.95 – 7.90 (m, 1H), 7.88 – 7.81 (m, 1H), 7.72 – 7.66 (m, 1H), 7.52 – 7.44 (m, 2H), 7.41 – 7.30 (m, 2H), 7.25 – 7.21 (m, 1H), 7.20 – 7.10 (m, 2H); **^{13}C NMR (100 MHz, $CDCl_3$)** δ 192.73, 155.51, 151.42, 149.23, 146.90, 141.88, 139.23, 137.19, 134.01, 132.66, 131.91, 131.60, 130.60, 130.36, 130.18, 129.16, 128.74, 128.32, 126.69, 125.14, 124.67, 122.27, 121.59, 119.69, 118.65; **HRMS (ESI, m/z)** calcd for $C_{25}H_{15}N_3O_8$ $[M+Na]^+$ = 508.0757, found = 508.0754; HPLC analysis: 86% ee (ADH column, 25 °C, n-hexane/*i*-PrOH = 70/30, 1.0 mL/min, λ = 254 nm), Rt (major) = 42.6 min, Rt (minor) = 38.3 min.

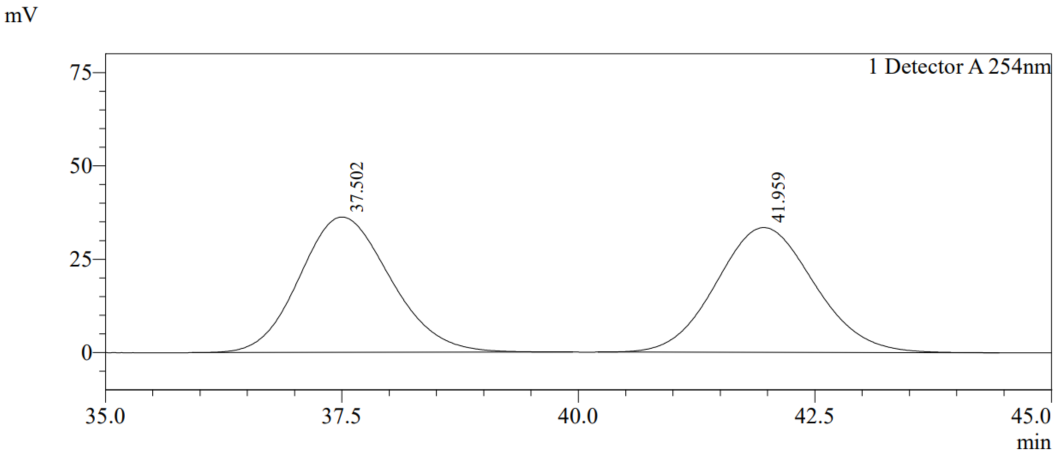

Peak Table

Detector A 254nm

Peak#	Ret. Time	Height	Height%	Area	Area%
1	37.502	36273	52.037	2433239	50.118
2	41.959	33434	47.963	2421805	49.882
Total		69707	100.000	4855044	100.000

Racemic **8**

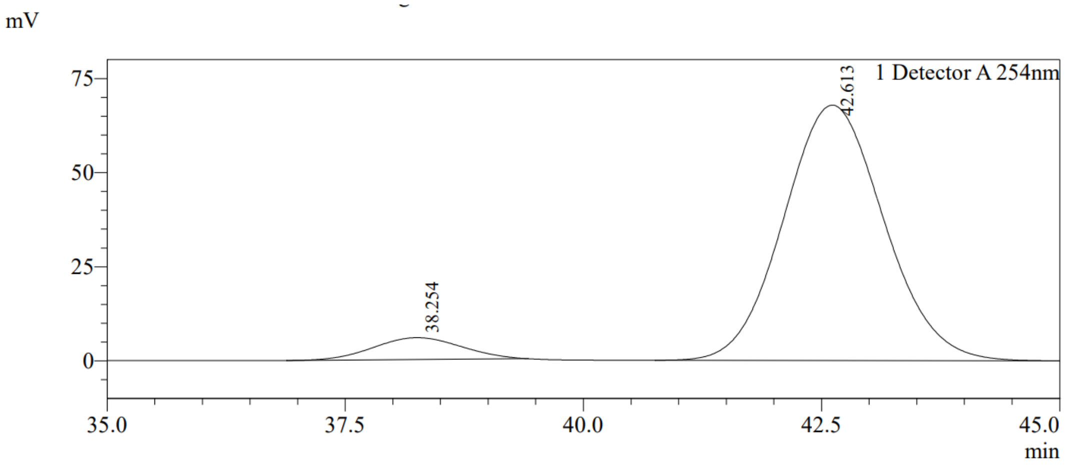

Peak Table

Detector A 254nm

Peak#	Ret. Time	Height	Height%	Area	Area%
1	38.254	5807	7.884	373897	6.971
2	42.613	67851	92.116	4989812	93.029
Total		73658	100.000	5363709	100.000

Enantiomerically enriched **8**

(*S*, *E*)-3-(3-chlorophenyl)-2-(2-(2,4-dinitrophenoxy)naphthalen-1-yl)acrylaldehyde (9)

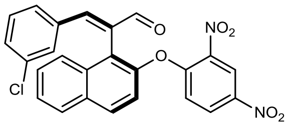

9

A yellow solid, 94% yield, 44.6 mg; m.p: 207-208 ^{o}C; **$[\alpha]^{25}_{D}$** = -208.6 (c = 0.25 in $CHCl_3$); **^{1}H NMR (400 MHz, $CDCl_3$)** δ 9.79 (s, 1H), 8.73 (d, *J* = 2.7 Hz, 1H), 8.14 (dd, *J* = 9.3, 2.7 Hz, 1H), 8.06 (d, *J* = 8.9 Hz, 1H), 7.98 (d, *J* = 8.0 Hz, 1H), 7.73 (s, 1H), 7.63 – 7.45 (m, 3H), 7.28 (d, *J* = 8.9 Hz, 1H), 7.23 (d, *J* = 7.1 Hz, 1H), 7.10 – 6.87 (m, 4H); **^{13}C NMR (100 MHz, $CDCl_3$)** δ 192.83, 155.26, 152.04, 148.18, 141.54, 139.31, 136.14, 135.37, 134.84, 132.46, 131.94, 131.90, 130.90, 130.25, 130.13, 128.98, 128.57, 128.36, 127.97, 126.96, 124.91, 122.76, 121.73, 119.67, 118.76; **HRMS (ESI, m/z)** calcd for $C_{25}H_{15}ClN_2O_6$ $[M+Na]^+$ = 497.0517, found = 497.0510; **HPLC analysis**: 90% ee (ADH column, 25 ^{o}C, n-hexane/*i*-PrOH = 80/20, 1.0 mL/min, λ = 254 nm), Rt (major) = 24.3 min, Rt (minor) = 33.1 min.

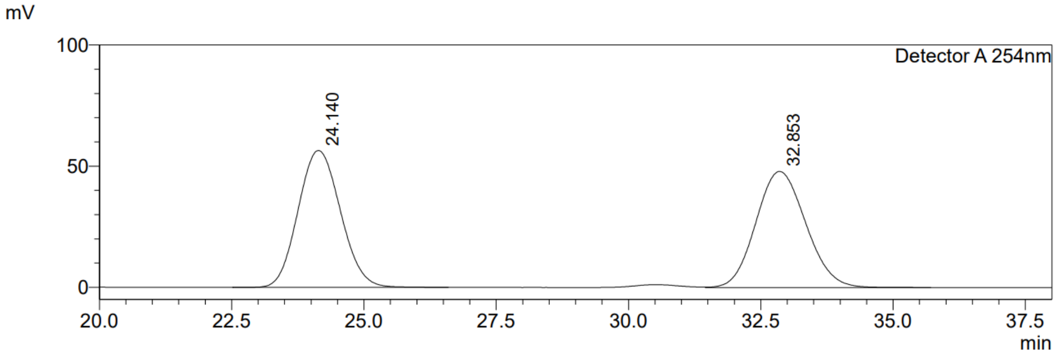

Detector A 254nm

Peak#	Ret. Time	Height	Height%	Area	Area%
1	24.140	56501	54.125	3153723	50.067
2	32.853	47890	45.875	3145278	49.933
Total		104391	100.000	6299002	100.000

Racemic **9**

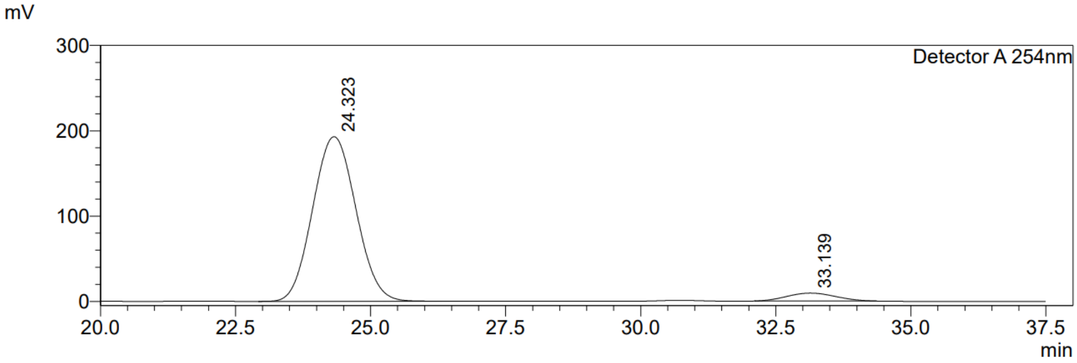

Detector A 254nm

Peak#	Ret. Time	Height	Height%	Area	Area%
1	24.323	193054	95.376	10761217	94.824
2	33.139	9359	4.624	587442	5.176
Total		202413	100.000	11348660	100.000

Enantiomerically enriched **9**

(*S*, *E*)-2-(2-(2,4-dinitrophenoxy)naphthalen-1-yl)-3-(4-methoxyphenyl)acrylaldehyde (10)

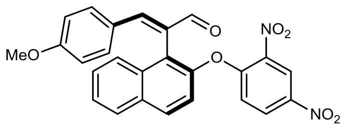

10

A yellow solid, 97% yield, 45.6 mg; m.p: 192-193 °C; **[α]$^{25}_{D}$** = -290.7 (c = 0.25 in $CHCl_3$); **^{1}H NMR (400 MHz, $CDCl_3$)** δ 9.70 (s, 1H), 8.72 (d, *J* = 2.7 Hz, 1H), 8.12 (dd, *J* = 9.3, 2.7 Hz, 1H), 8.05 (d, *J* = 8.9 Hz, 1H), 7.97 (d, *J* = 8.1 Hz, 1H), 7.70 (s, 1H), 7.64 (d, *J* = 8.3 Hz, 1H), 7.60 – 7.52 (m, 1H), 7.52 – 7.45 (m, 1H), 7.30 (d, *J* = 8.9 Hz, 1H), 7.01 (dd, *J* = 9.1, 2.0 Hz, 3H), 6.70 – 6.61 (m, 2H), 3.74 (s, 3H); **^{13}C NMR (100 MHz, $CDCl_3$)** δ 193.06, 162.14, 155.59, 153.97, 148.34, 141.39, 139.22, 132.70, 132.65, 132.28, 132.07, 131.53, 128.85, 128.55, 128.14, 126.85, 126.41, 125.25, 123.91, 121.66, 119.95, 119.08, 114.48, 55.46; **HRMS (ESI, m/z)** calcd for $C_{26}H_{18}N_2O_7$ $[M+Na]^+$ = 493.1012, found = 493.1012; **HPLC analysis**: 96% ee (ADH column, 25 °C, n-hexane/*i*-PrOH = 80/20, 1.0 mL/min, λ = 254 nm), Rt (major) = 25.1 min, Rt (minor) = 35.4 min.

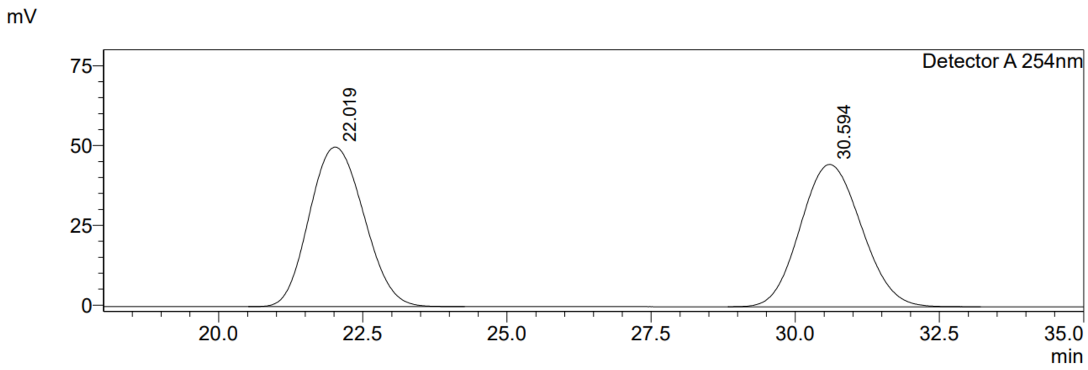

Detector A 254nm

Peak#	Ret. Time	Height	Height%	Area	Area%
1	22.019	49995	52.854	3297678	50.008
2	30.594	44596	47.146	3296648	49.992
Total		94591	100.000	6594326	100.000

Racemic **10**

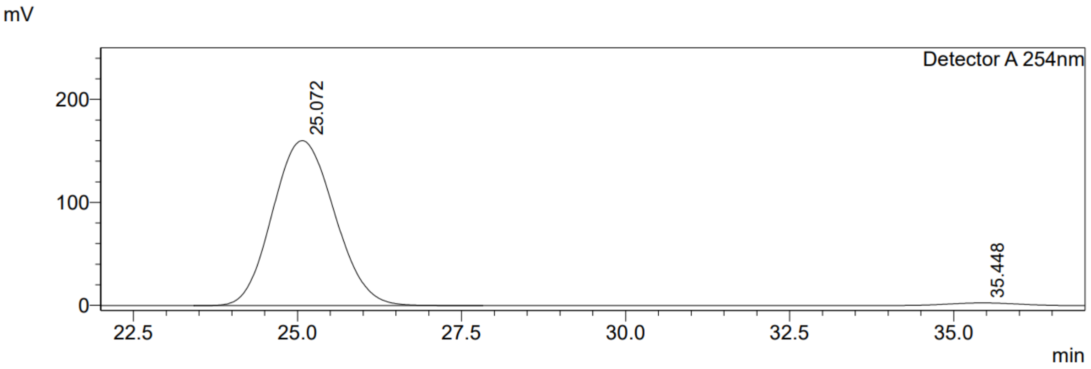

Detector A 254nm

Peak#	Ret. Time	Height	Height%	Area	Area%
1	25.072	160155	98.344	10523243	98.031
2	35.448	2697	1.656	211355	1.969
Total		162852	100.000	10734598	100.000

Enantiomerically enriched **10**

(*S*, *E*)-2-(2-(2,4-dinitrophenoxy)naphthalen-1-yl)-3-(*p*-tolyl)acrylaldehyde (11)

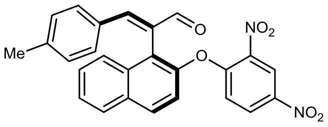

11

A yellow solid, 95% yield, 43.1 mg; m.p: 181-182 ^{o}C; **[α]$^{25}_{D}$** = -240.2 (c = 0.25 in $CHCl_3$); **^{1}H NMR (400 MHz, $CDCl_3$)** δ 9.75 (s, 1H), 8.71 (d, *J* = 2.7 Hz, 1H), 8.14 – 8.00 (m, 2H), 7.97 (d, *J* = 8.1 Hz, 1H), 7.75 (s, 1H), 7.67 – 7.60 (m, 1H), 7.58 – 7.45 (m, 2H), 7.29 (d, *J* = 8.9 Hz, 1H), 7.01 – 6.89 (m, 5H), 2.26 (s, 3H); **^{13}C NMR (100 MHz, $CDCl_3$)** δ 193.16, 155.51, 154.25, 148.20, 142.09, 141.36, 139.21, 133.81, 132.72, 132.01, 131.54, 130.96, 130.56, 129.70, 128.85, 128.50, 128.15, 126.83, 125.20, 123.76, 121.64, 119.90, 118.97, 21.62; **HRMS (ESI, m/z)** calcd for $C_{26}H_{18}N_2O_6$ $[M+Na]^+$ = 477.1063, found = 477.1060; **HPLC analysis**: 95% ee (ADH column, 25 ^{o}C, n-hexane/*i*-PrOH = 80/20, 1.0 mL/min, λ = 254 nm), Rt (major) = 20.5 min, Rt (minor) = 23.2 min.

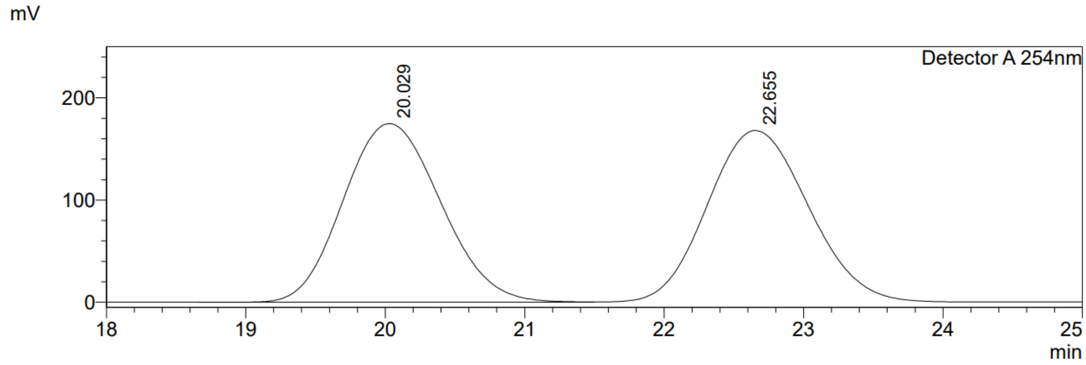

Detector A 254nm

Peak#	Ret. Time	Height	Height%	Area	Area%
1	20.029	174774	51.045	8496289	50.260
2	22.655	167620	48.955	8408217	49.740
Total		342394	100.000	16904506	100.000

Racemic **11**

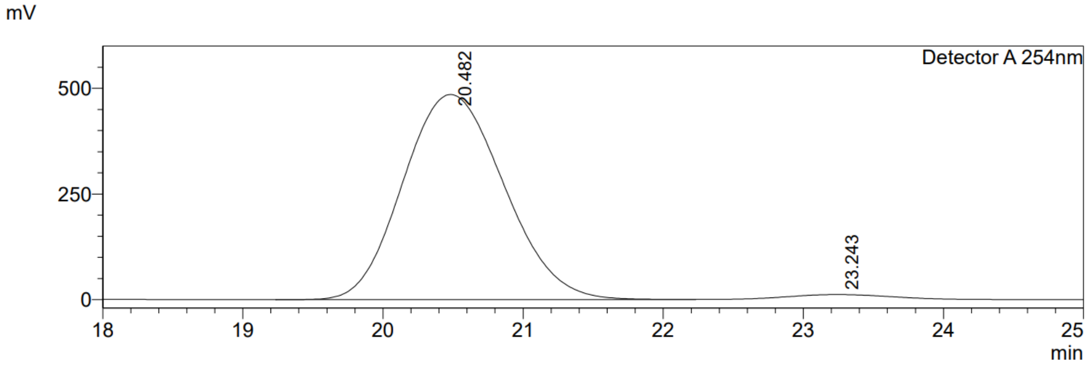

Detector A 254nm

Peak#	Ret. Time	Height	Height%	Area	Area%
1	20.482	485492	97.609	24345957	97.497
2	23.243	11894	2.391	625071	2.503
Total		497386	100.000	24971028	100.000

Enantiomerically enriched **11**

(*S*, *E*)-3-(4-bromophenyl)-2-(2-(2,4-dinitrophenoxy)naphthalen-1-yl)acrylaldehyde (12)

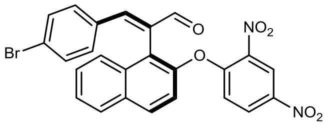

12

A yellow solid, 93% yield, 48.3 mg; m.p: 195-196 °C; **$[\alpha]^{25}_{D}$** = -262.5 (c = 0.25 in $CHCl_3$); **^{1}H NMR (400 MHz, $CDCl_3$)** δ 9.76 (s, 1H), 8.73 (d, *J* = 2.7 Hz, 1H), 8.18 (dd, *J* = 9.3, 2.7 Hz, 1H), 8.05 (d, *J* = 8.9 Hz, 1H), 7.97 (d, *J* = 8.1 Hz, 1H), 7.74 (s, 1H), 7.62 – 7.46 (m, 3H), 7.30 – 7.27 (m, 2H), 7.01 (d, *J* = 9.3 Hz, 1H), 6.93 (d, *J* =

8.6 Hz, 2H); **^{13}C NMR (100 MHz, $CDCl_3$)** δ 192.92, 155.30, 152.60, 148.21, 141.56, 139.31, 135.33, 132.53, 132.40, 132.26, 131.96, 131.86, 131.63, 128.96, 128.69, 128.35, 126.96, 125.89, 124.92, 122.93, 121.74, 119.69, 118.95; **HRMS (ESI, m/z)** calcd for $C_{25}H_{15}BrN_2O_6$ $[M+Na]^+$ = 541.0011, found = 541.0009; **HPLC analysis**: 94% ee (IE column, 25 ^{o}C, n-hexane/*i*-PrOH = 80/20, 1.0 mL/min, λ = 254 nm), Rt (major) = 28.0 min, Rt (minor) = 21.9 min.

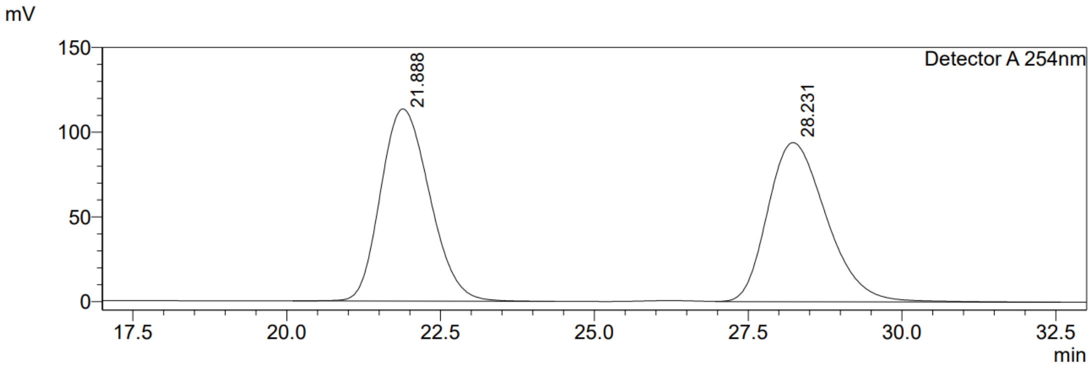

Detector A 254nm

Peak#	Ret. Time	Height	Height%	Area	Area%
1	21.888	113436	54.699	6291779	50.127
2	28.231	93945	45.301	6259864	49.873
Total		207380	100.000	12551643	100.000

Racemic **12**

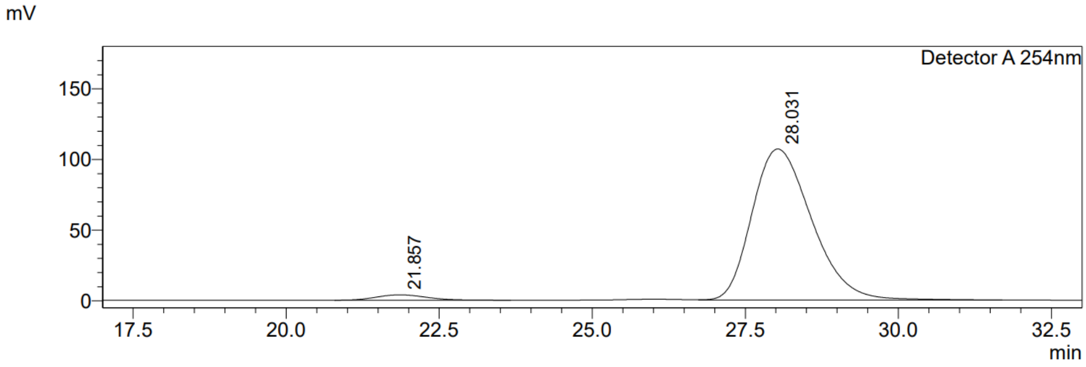

Detector A 254nm

Peak#	Ret. Time	Height	Height%	Area	Area%
1	21.857	3787	3.421	215003	2.874
2	28.031	106916	96.579	7266494	97.126
Total		110703	100.000	7481497	100.000

Enantiomerically enriched **12**

(*S*, *E*)-3-(4-chlorophenyl)-2-(2-(2,4-dinitrophenoxy)naphthalen-1-yl)acrylaldehyde (13)

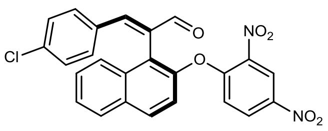

13

A yellow solid, 99% yield, 46.9 mg; m.p: 207-208 ^{o}C; **[α]$^{25}_{D}$** = -322.5 (c = 0.25 in $CHCl_3$); **^{1}H NMR (400 MHz, $CDCl_3$)** δ 9.76 (s, 1H), 8.73 (d, *J* = 2.7 Hz, 1H), 8.18 (dd, *J* = 9.3, 2.7 Hz, 1H), 8.05 (d, *J* = 8.9 Hz, 1H), 7.97 (d, *J* = 8.1 Hz, 1H), 7.75 (s, 1H), 7.63 – 7.45 (m, 3H), 7.28 (d, *J* = 9.0 Hz, 1H), 7.13 (d, *J* = 8.5 Hz, 2H), 7.02 (dd, *J* = 8.9, 4.8 Hz, 3H); **^{13}C NMR (100 MHz, $CDCl_3$)** δ 192.93, 155.31, 152.55, 148.25, 141.56, 139.30, 137.36, 135.18, 132.41, 132.11, 131.97, 131.85, 131.51, 129.28, 128.95, 128.68, 128.32, 126.95, 124.93, 122.94, 121.74, 119.69, 118.97; **HRMS (ESI, m/z)** calcd for $C_{25}H_{15}ClN_2O_6$ $[M+Na]^+$ = 497.0517, found = 497.0511; HPLC analysis: 95% ee (IE column, 25 ^{o}C, n-hexane/*i*-PrOH = 80/20, 1.0 mL/min, λ = 254 nm), Rt (major) = 25.3 min, Rt (minor) = 20.2 min.

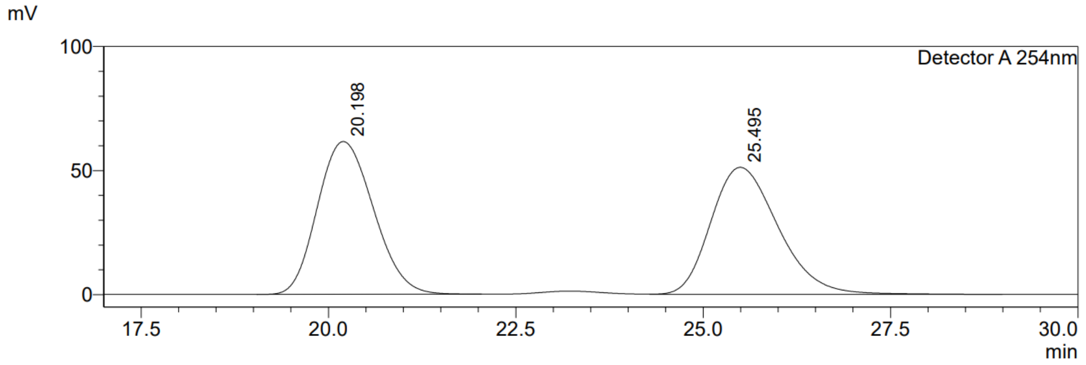

Detector A 254nm

Peak#	Ret. Time	Height	Height%	Area	Area%
1	20.198	61596	54.621	3209335	50.062
2	25.495	51174	45.379	3201447	49.938
Total		112770	100.000	6410782	100.000

Racemic **13**

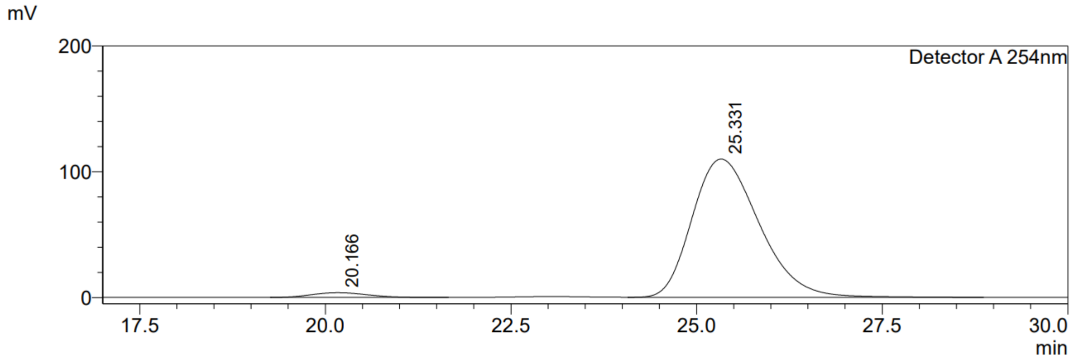

Detector A 254nm

Peak#	Ret. Time	Height	Height%	Area	Area%
1	20.166	3741	3.289	194769	2.735
2	25.331	110000	96.711	6926320	97.265
Total		113741	100.000	7121090	100.000

Enantiomerically enriched **13**

(*S*, *E*)-2-(2-(2,4-dinitrophenoxy)naphthalen-1-yl)-3-(4-fluorophenyl)acrylaldehyde (14)

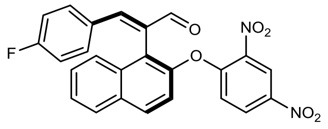

14

A yellow solid, 91% yield, 41.7 mg; m.p: 169-171 ^{o}C; **$[\alpha]^{25}_{D}$** = -262 (c = 0.25 in $CHCl_3$); **^{1}H NMR (400 MHz, $CDCl_3$)** δ 9.75 (s, 1H), 8.74 (d, *J* = 2.7 Hz, 1H), 8.18 (dd, *J* = 9.3, 2.7 Hz, 1H), 8.06 (d, *J* = 8.9 Hz, 1H), 7.98 (d, *J* = 8.1 Hz, 1H), 7.76 (s, 1H), 7.64 – 7.46 (m, 3H), 7.28 (d, *J* = 8.9 Hz, 1H), 7.14 – 6.99 (m, 3H), 6.85 (t, *J* = 8.6 Hz, 2H); **^{13}C NMR (100 MHz, $CDCl_3$)** δ 193.03, 164.20 (d, *J* = 254.3 Hz), 155.38, 152.80, 148.35, 141.57, 139.31, 132.63 (d, *J* = 8.8 Hz), 132.45, 132.02, 131.83, 129.96 (d, *J* = 3.5 Hz), 128.95, 128.69, 128.28, 126.94, 124.99, 123.08, 121.74, 119.73, 119.04, 116.27 (d, *J* = 21.9 Hz); ^{19}F NMR (376 MHz, $CDCl_3$) δ -106.99; **HRMS (ESI, m/z)** calcd for $C_{25}H_{15}FN_2O_6$ $[M+Na]^+$ = 481.0812, found = 481.0811; **HPLC analysis**: 95% ee (ADH column, 25 ^{o}C, n-hexane/*i*-PrOH = 80/20, 1.0 mL/min, λ = 254 nm), Rt (major) = 19.3 min, Rt (minor) = 26.1 min.

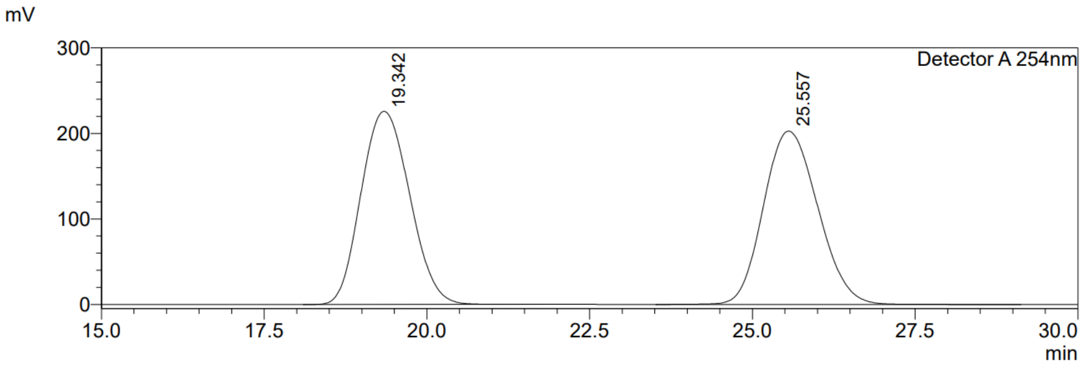

Detector A 254nm

Peak#	Ret. Time	Height	Height%	Area	Area%
1	19.342	225792	52.679	11740425	49.864
2	25.557	202831	47.321	11804593	50.136
Total		428623	100.000	23545018	100.000

Racemic **14**

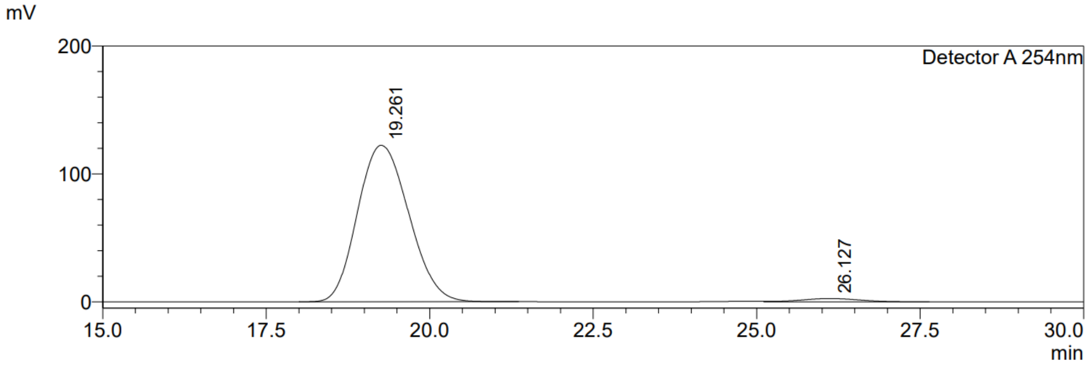

Detector A 254nm

Peak#	Ret. Time	Height	Height%	Area	Area%
1	19.261	122399	97.951	6670817	97.689
2	26.127	2561	2.049	157802	2.311
Total		124960	100.000	6828619	100.000

Enantiomerically enriched **14**

(*S*, *E*)-2-(2-(2,4-dinitrophenoxy)naphthalen-1-yl)-3-(naphthalen-2-yl)acrylaldehyde (15)

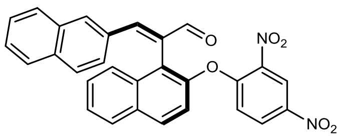

15

A yellow solid, 99% yield, 48.5 mg; m.p: 177-178 °C; **[α]$^{25}_{D}$** = -170.1 (c = 0.25 in $CHCl_3$); **^{1}H NMR (400 MHz, $CDCl_3$)** δ 9.85 (s, 1H), 8.67 (d, *J* = 2.7 Hz, 1H), 8.10 – 8.04 (m, 1H), 8.01 – 7.96 (m, 2H), 7.88 (dd, *J* = 9.3, 2.7 Hz, 1H), 7.73 – 7.66 (m, 3H), 7.62 – 7.54 (m, 2H), 7.53 – 7.38 (m, 4H), 7.29 (d, *J* = 8.9 Hz, 1H), 6.94 (dd, *J* = 8.7,

1.8 Hz, 1H), 6.88 (d, J = 9.3 Hz, 1H); **^{13}C NMR (100 MHz, $CDCl_3$)** δ 193.05, 155.27, 154.08, 148.23, 141.25, 139.20, 135.07, 134.22, 132.96, 132.91, 132.23, 131.98, 131.61, 131.37, 128.90, 128.85, 128.59, 128.32, 128.27, 128.23, 127.71, 126.98, 126.91, 125.57, 125.28, 123.63, 121.56, 119.87, 118.53; **HRMS (ESI, m/z)** calcd for $C_{29}H_{18}N_2O_6$ $[M+Na]^+$ = 513.1063, found = 513.1057; **HPLC analysis**: 97% ee (ADH column, 25 °C, n-hexane/*i*-PrOH = 70/30, 1.0 mL/min, λ = 254 nm), Rt (major) = 20.2 min, Rt (minor) = 15.7 min.

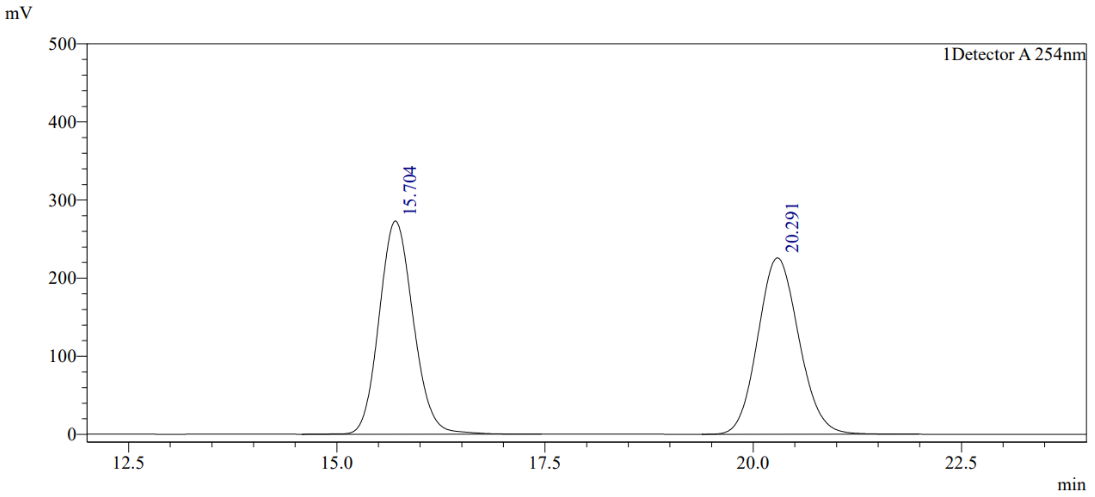

Peak Table

Detector A 254nm

Peak#	Ret. Time	Height	Height%	Area	Area%
1	15.704	272973	54.715	7796838	50.103
2	20.291	225926	45.285	7764693	49.897
Total		498899	100.000	15561530	100.000

Racemic **15**

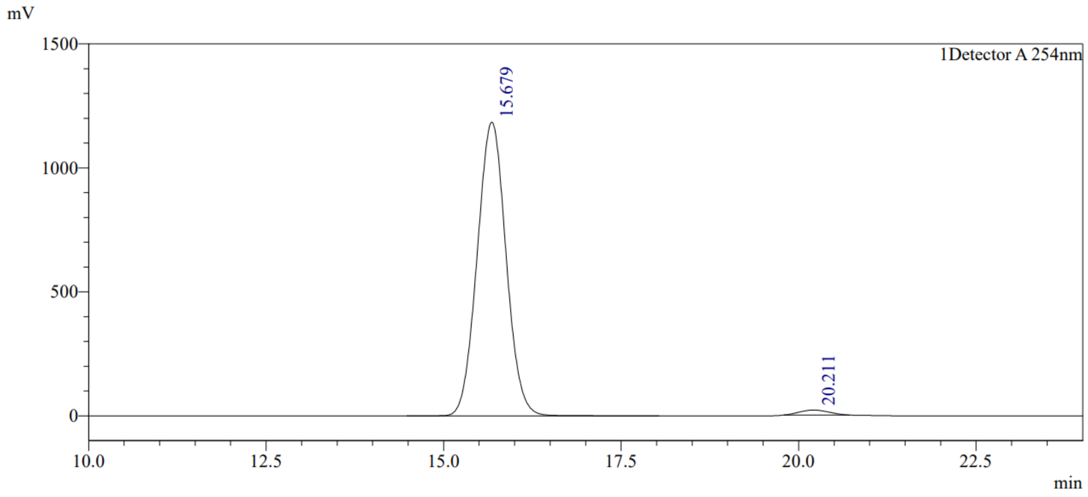

Peak Table

Detector A 254nm

Peak#	Ret. Time	Height	Height%	Area	Area%
1	15.679	1185043	98.327	33550472	98.306
2	20.211	20158	1.673	578068	1.694
Total		1205201	100.000	34128540	100.000

Enantiomerically enriched **15**

***(S, E)*-3-(2-bromo-4-(trifluoromethyl)phenyl)-2-(2-(2,4-dinitrophenoxy)naphthalen-1-yl)acrylaldehyde (16)**

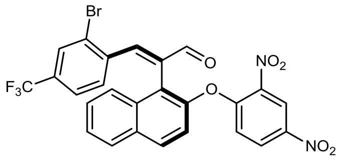

16

A yellow solid, 99% yield, 57.9 mg; m.p: 90-92 °C; **[α]$^{25}_{D}$** = -134.5 (c = 0.25 in CHCl3); **^{1}H NMR (400 MHz, $CDCl_3$)** δ 9.93 (s, 1H), 8.84 (d, *J* = 2.7 Hz, 1H), 8.20 (dd, *J* = 9.3, 2.8 Hz, 1H), 8.09 (d, *J* = 0.8 Hz, 1H), 8.03 – 7.97 (m, 1H), 7.95 – 7.89 (m, 1H), 7.80 – 7.76 (m, 1H), 7.65 – 7.58 (m, 1H), 7.57 – 7.50 (m, 2H), 7.22 – 7.16 (m, 2H), 7.05 (d, *J* = 8.2 Hz, 1H), 7.01 (d, *J* = 9.3 Hz, 1H); **^{13}C NMR (100 MHz, $CDCl_3$)** δ 192.52, 155.24, 150.72, 148.78, 141.81, 139.33, 138.53, 137.63, 133.06 (q, *J* = 33.5 Hz), 132.43, 132.11, 131.71, 130.09, 130.02 (q, *J* = 4.0 Hz), 129.00, 128.84, 128.48, 126.90, 125.02, 124.60, 124.42 (q, *J* = 3.7 Hz), 122.71 (q, *J* = 273.1 Hz), 122.20, 121.43, 119.10, 118.99; **^{19}F NMR (376 MHz, $CDCl_3$)** δ -63.14; **HRMS (ESI, m/z)** calcd for $C_{26}H_{14}BrF_3N_2O_6$ $[M+Na]^+$ = 610.9865, found = 610.9859; HPLC analysis: 82% ee (IE column, 25 °C, n-hexane/*i*-PrOH = 80/20, 1.0 mL/min, λ = 254 nm), Rt (major) = 20.2 min, Rt (minor) = 15.7 min.

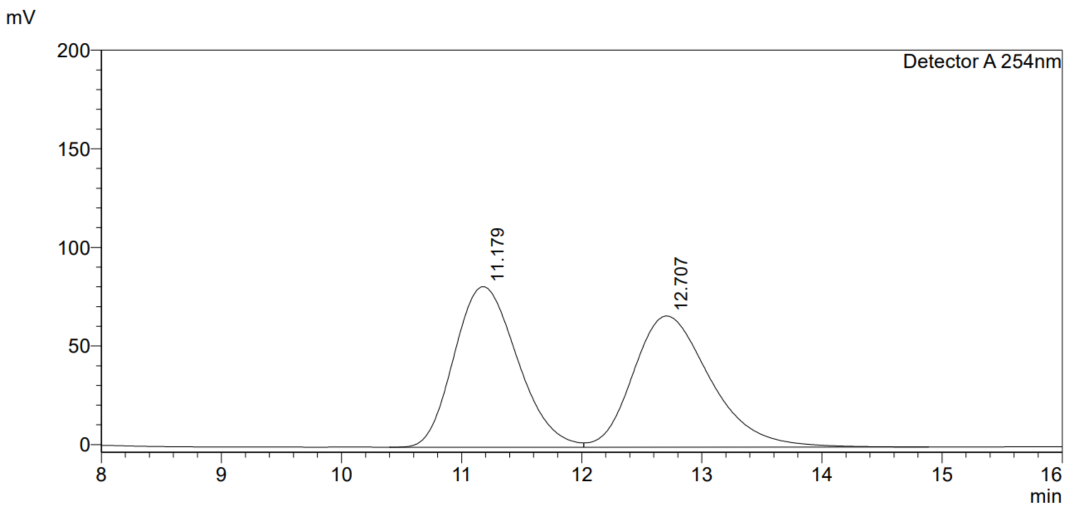

Detector A 254nm

Peak#	Ret. Time	Area	Height	Height%	Area%
1	11.179	2986107	81420	55.044	50.070
2	12.707	2977794	66498	44.956	49.930
Total		5963901	147917	100.000	100.000

Racemic **16**

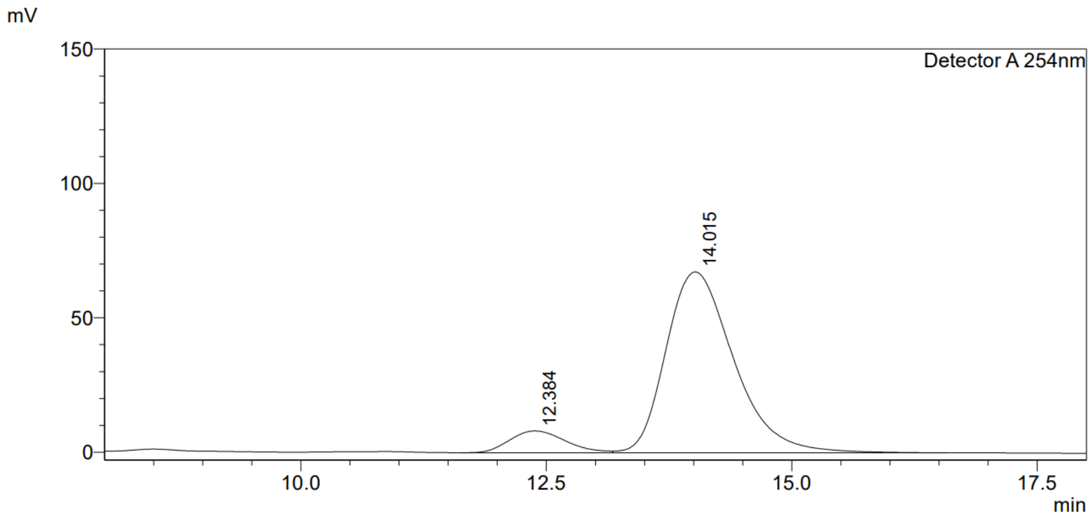

Detector A 254nm

Peak#	Ret. Time	Area	Height	Height%	Area%
1	12.384	322673	8129	10.777	9.070
2	14.015	3234748	67298	89.223	90.930
Total		3557421	75426	100.000	100.000

Enantiomerically enriched **16**

(*S*, *E*)-3-(3,5-di-*tert*-butylphenyl)-2-(2-(2,4-dinitrophenoxy)naphthalen-1-yl)acrylaldehyde (17)

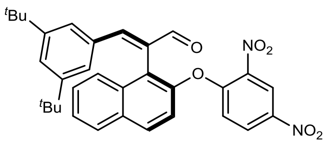

17

A yellow solid, 99% yield, 54.5 mg; m.p: 83-84 ^{o}C; **$[\alpha]^{25}_{D}$** = -63.2 (c = 0.25 in $CHCl_3$); **^{1}H NMR (400 MHz, $CDCl_3$)** δ 9.81 (s, 1H), 8.71 (d, *J* = 2.7 Hz, 1H), 8.05 – 8.02 (m, 1H), 8.01 – 7.98 (m, 1H), 7.98 – 7.95 (m, 1H), 7.80 (s, 1H), 7.68 (m, *J* = 8.1, 1.5, 0.8 Hz, 1H), 7.52 (m, *J* = 22.6, 8.3, 6.9, 1.4 Hz, 2H), 7.31 (t, *J* = 1.8 Hz, 1H), 7.28 (d, *J* = 8.9 Hz, 1H), 6.92 – 6.87 (m, 3H), 1.00 (s, 18H); **^{13}C NMR (100 MHz, $CDCl_3$)** δ 193.20, 155.56, 154.99, 151.31, 148.22, 141.34, 139.39, 134.28, 132.89, 132.80, 132.04, 131.35, 128.80, 128.23, 126.80, 125.49, 125.31, 125.23, 124.17, 121.59, 119.84, 119.01, 34.68, 31.08; **HRMS (ESI, m/z)** calcd. for $C_{33}H_{32}N_2O_6$ $[M+Na]^+$ = 575.2158, found = 575.2164; HPLC analysis: 96% ee (IE column, 25 ^{o}C, n-hexane / *i*-PrOH = 90/10, 1.0 mL/min, λ = 254 nm), Rt (major) = 29.1 min, Rt (minor) = 17.8 min.

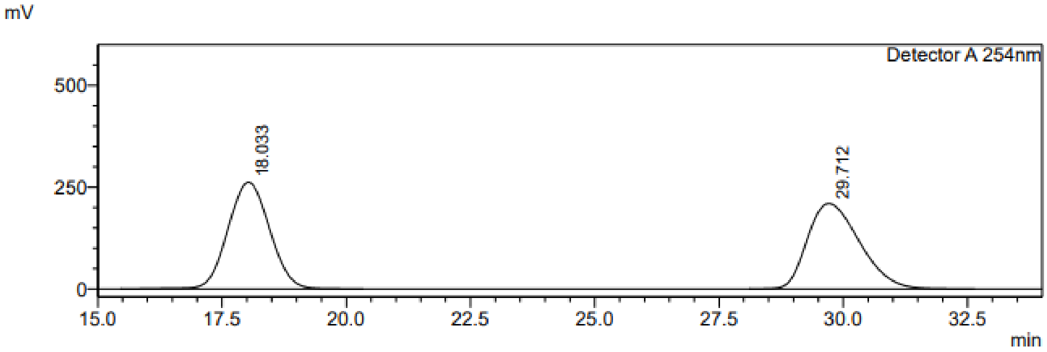

Detector A 254nm

Peak#	Ret. Time	Height	Height%	Area	Area%
1	18.033	262144	55.567	15263434	50.003
2	29.712	209618	44.433	15261793	49.997
Total		471762	100.000	30525226	100.000

Racemic **17**

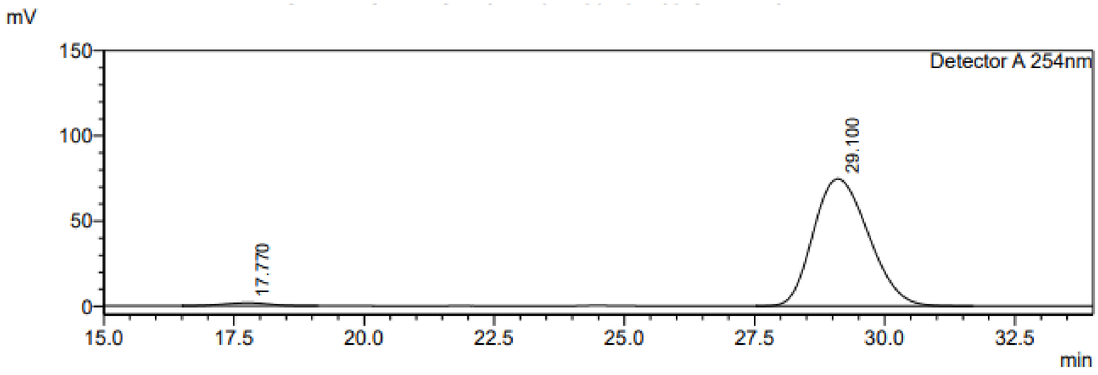

Detector A 254nm

Peak#	Ret. Time	Height	Height%	Area	Area%
1	17.770	1795	2.349	106889	1.906
2	29.100	74635	97.651	5499739	98.094
Total		76430	100.000	5606629	100.000

Enantiomerically enriched **17**

(*S*, *E*)-2-(2-(2,4-dinitrophenoxy)naphthalen-1-yl)-3-(furan-2-yl)acrylaldehyde (18)

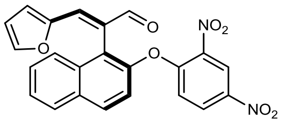

18

A yellow solid, 99% yield, 42.5 mg; m.p: 114-115 °C; **[α]$^{25}_{D}$** = -321.4 (c = 0.25 in $CHCl_3$); **^{1}H NMR (400 MHz, $CDCl_3$)** δ 9.68 (s, 1H), 8.74 (d, *J* = 2.7 Hz, 1H), 8.18 (dd, *J* = 9.3, 2.8 Hz, 1H), 8.05 (d, *J* = 8.9 Hz, 1H), 7.97 (d, *J* = 8.1 Hz, 1H), 7.65 – 7.44 (m, 4H), 7.38 (d, *J* = 1.4 Hz, 1H), 7.31 (d, *J* = 8.9 Hz, 1H), 7.05 (d, *J* = 9.3 Hz, 1H), 6.33 (dd, *J* = 3.5, 1.7 Hz, 1H), 6.14 (d, *J* = 3.6 Hz, 1H); **^{13}C NMR (100 MHz, $CDCl_3$)** δ 191.64, 155.56, 149.98, 148.28, 146.70, 141.29, 139.08, 138.97, 132.44, 131.88, 131.49, 130.66, 128.62, 128.49, 127.80, 126.60, 124.99, 123.31, 121.64, 119.62, 118.78, 118.46, 113.17; **HRMS (ESI, m/z)** calcd for $C_{23}H_{14}N_2O_7$ $[M+Na]^+$ = 453.0699, found = 453.0695; **HPLC analysis**: 92% ee (ADH column, 25 °C, n-hexane/*i*-PrOH = 70/30, 1.0 mL/min, λ = 254 nm), Rt (major) = 19.5 min, Rt (minor) = 25.0 min.

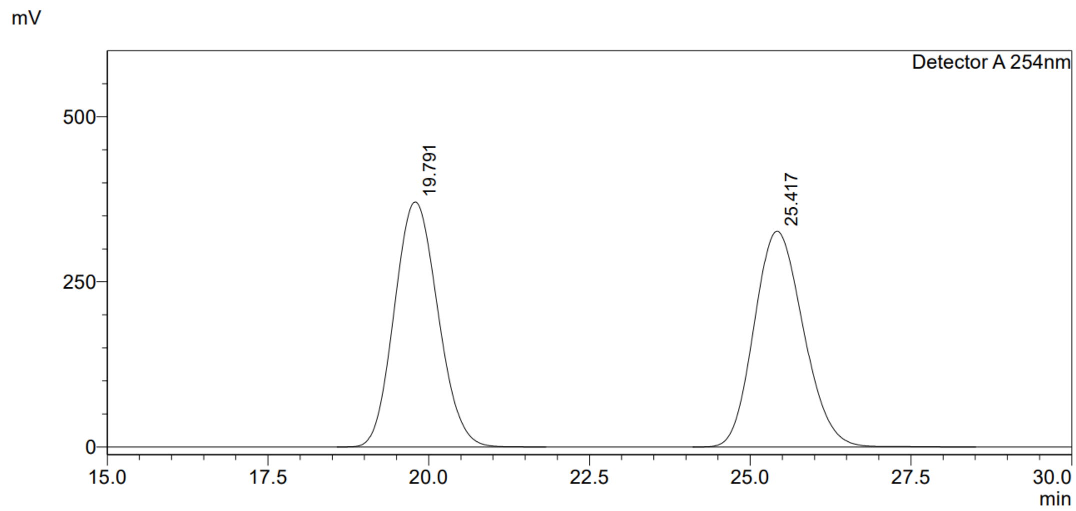

Detector A 254nm

Peak#	Ret. Time	Area	Height	Height%	Area%
1	19.791	17464105	371076	53.177	50.066
2	25.417	17417894	326733	46.823	49.934
Total		34882000	697809	100.000	100.000

Racemic **18**

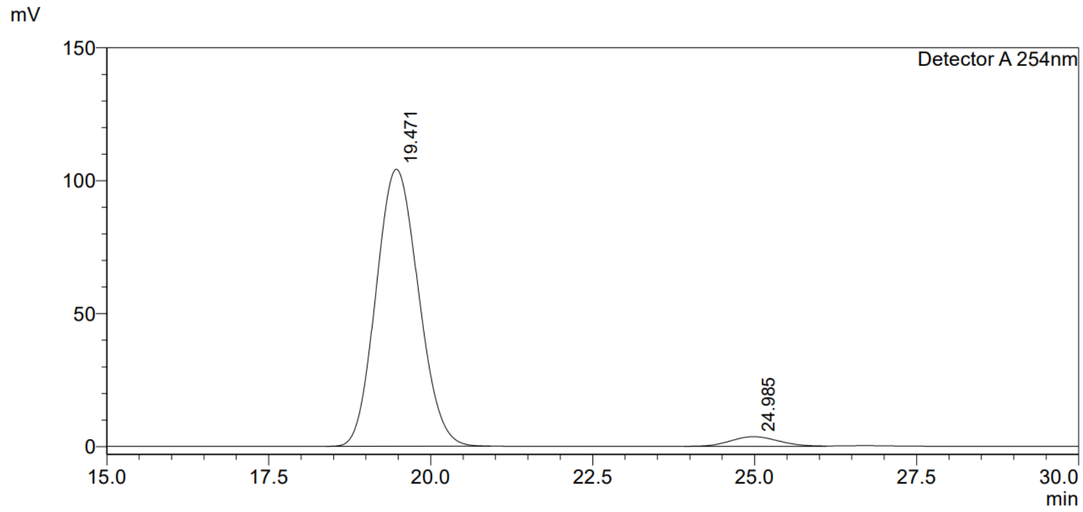

Detector A 254nm

Peak#	Ret. Time	Area	Height	Height%	Area%
1	19.471	4744163	104174	96.634	96.209
2	24.985	186947	3629	3.366	3.791
Total		4931110	107803	100.000	100.000

Enantiomerically enriched **18**

(*S*, *E*)-2-(2-(2,4-dinitrophenoxy)naphthalen-1-yl)-3-(thiophen-2-yl)acrylaldehyde (19)

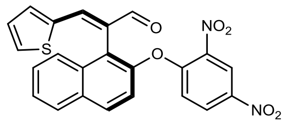

19

A yellow solid, 99% yield, 44.1 mg; m.p: 93-94 ^{o}C; $[\alpha]^{25}_{D}$ = -403.5 (c = 0.25 in

$CHCl_3$); **^{1}H NMR (400 MHz, $CDCl_3$)** δ 9.72 (s, 1H), 8.74 (d, *J* = 2.7 Hz, 1H), 8.19 (dd, *J* = 9.3, 2.8 Hz, 1H), 8.10 (d, *J* = 8.9 Hz, 1H), 7.98 (d, *J* = 6.9 Hz, 2H), 7.62 (d, *J* = 8.3 Hz, 1H), 7.59 – 7.53 (m, 1H), 7.48 (m, *J* = 8.2, 7.0, 1.2 Hz, 1H), 7.37 – 7.29 (m, 3H), 7.11 (d, *J* = 9.3 Hz, 1H), 6.99 (dd, *J* = 5.0, 3.8 Hz, 1H); **^{13}C NMR (100 MHz, $CDCl_3$)** δ 191.86, 155.44, 148.97, 145.81, 141.40, 139.18, 137.28, 135.33, 133.70, 132.69, 132.13, 132.11, 131.50, 128.70, 128.56, 128.06, 127.51, 126.78, 124.64, 122.57, 121.59, 119.88, 119.10; **HRMS (ESI, m/z)** calcd for $C_{23}H_{14}N_2O_6S$ $[M+Na]^+$ = 469.0471, found = 469.0470; HPLC analysis: 90% ee (ADH column, 25 ^{o}C, n-hexane/*i*-PrOH = 70/30, 1.0 mL/min, λ = 254 nm), Rt (major) = 23.6 min, Rt (minor) = 32.0 min.

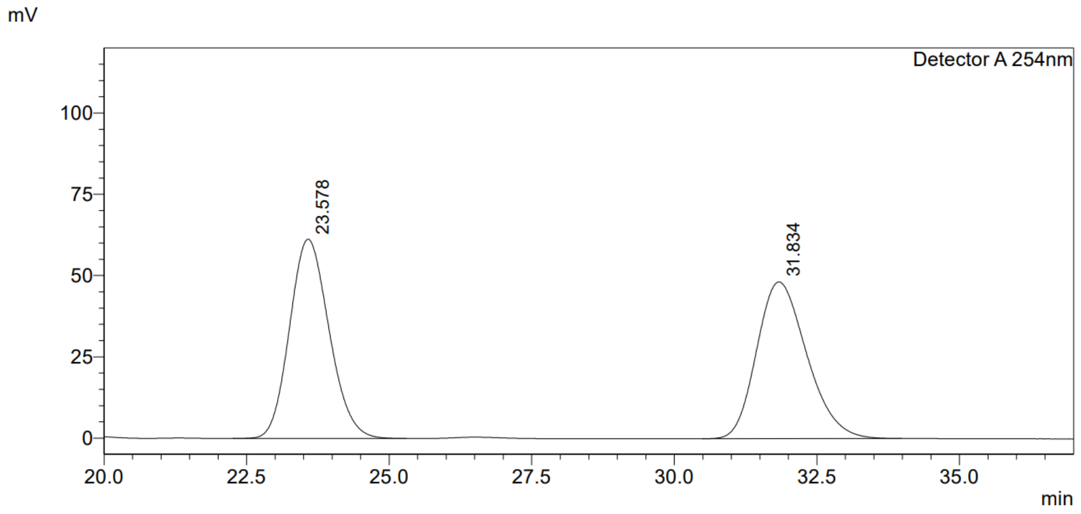

Detector A 254nm

Peak#	Ret. Time	Area	Height	Height%	Area%
1	23.578	2961067	61302	55.984	50.101
2	31.834	2949089	48197	44.016	49.899
Total		5910157	109498	100.000	100.000

Racemic **19**

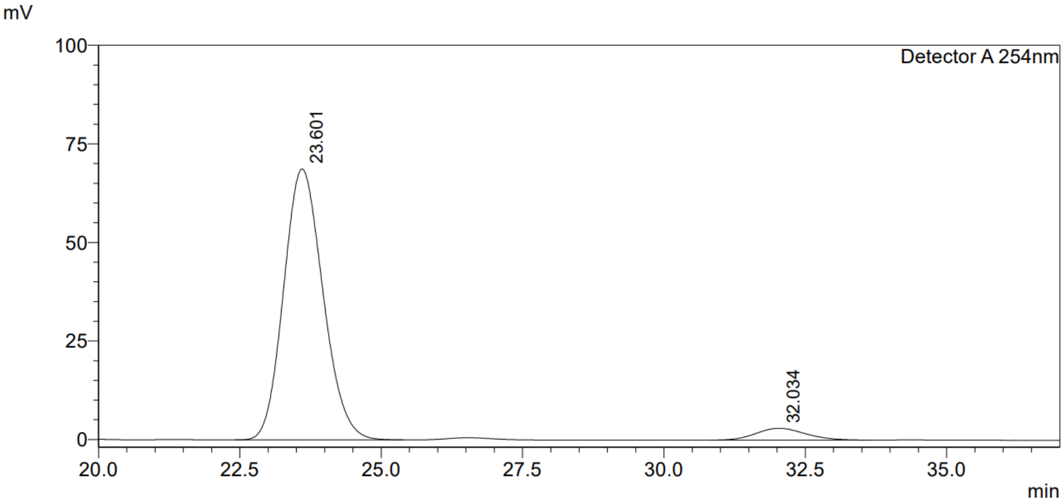

Detector A 254nm

Peak#	Ret. Time	Area	Height	Height%	Area%
1	23.601	3328899	68747	95.841	94.840
2	32.034	181124	2983	4.159	5.160
Total		3510023	71730	100.000	100.000

Enantiomerically enriched **19**

(*S*, *E*)-2-(2-(2,4-dinitrophenoxy)-7-phenylnaphthalen-1-yl)-3-phenylacrylaldehyde (20)

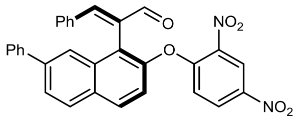

20

A yellow solid, 99% yield, 51.1 mg; m.p: 91-92 ^{o}C; **$[\alpha]^{25}_{D}$** = +74.3 (c = 0.25 in $CHCl_3$); **^{1}H NMR (400 MHz, $CDCl_3$)** δ 9.81 (s, 1H), 8.73 (d, *J* = 2.8 Hz, 1H), 8.10 (dd, *J* = 9.3, 2.8 Hz, 1H), 8.08 – 8.02 (m, 2H), 7.84 – 7.76 (m, 3H), 7.54 – 7.49 (m, 2H), 7.45 – 7.39 (m, 2H), 7.38 – 7.33 (m, 1H), 7.31 – 7.26 (m, 2H), 7.19 – 7.09 (m, 4H), 6.98 (d, *J* = 9.3 Hz, 1H); **^{13}C NMR (100 MHz, $CDCl_3$)** δ 193.17, 155.39, 154.29, 148.65, 141.43, 141.07, 140.67, 139.26, 134.78, 133.71, 132.98, 131.38, 131.25, 131.11, 130.42, 129.39, 129.01, 128.94, 128.55, 127.97, 127.70, 126.74, 123.60, 123.12, 121.68, 119.75, 118.91; **HRMS (ESI, m/z)** calcd. for $C_{31}H_{20}N_2O_6$ $[M+Na]^+$ = 539.1219, found = 539.1209; **HPLC analysis**: 92% ee (IE column, 25 ^{o}C, n-hexane/*i*-PrOH = 80/20, 1.0 mL/min, λ = 254 nm), Rt (major) = 31.0 min, Rt (minor) = 21.6 min.

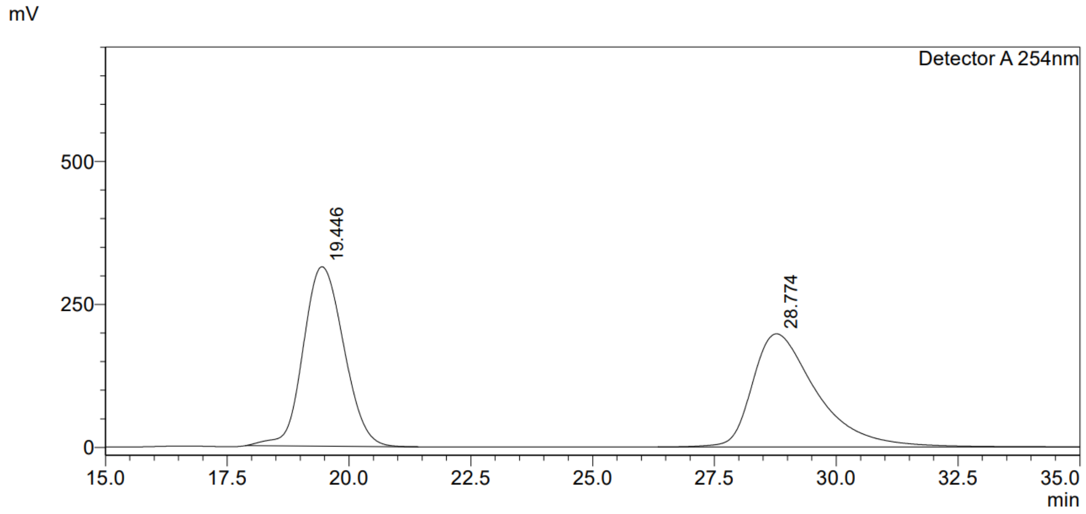

Detector A 254nm

Peak#	Ret. Time	Area	Height	Height%	Area%
1	19.446	18246092	313927	61.336	50.001
2	28.774	18245424	197891	38.664	49.999
Total		36491516	511819	100.000	100.000

Racemic **20**

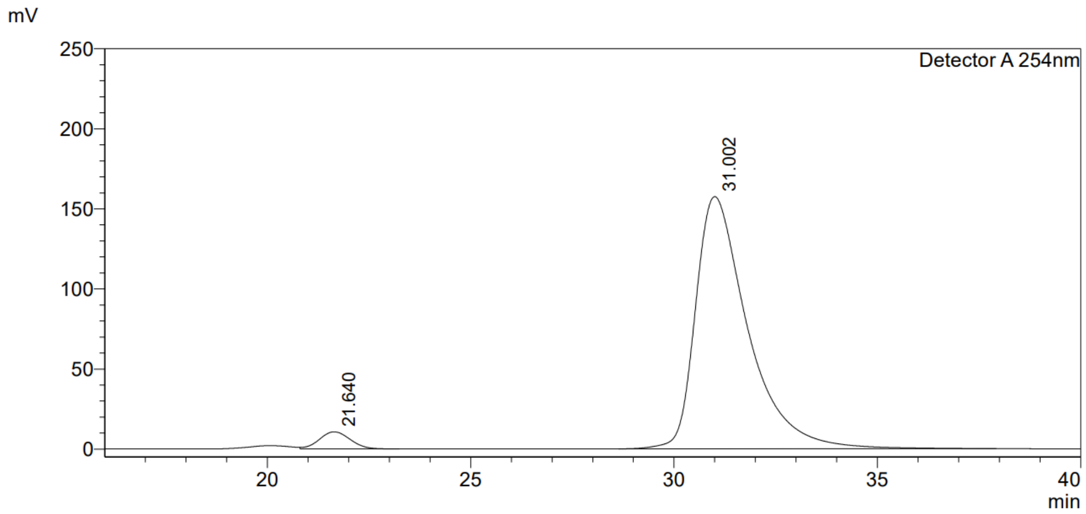

Detector A 254nm

Peak#	Ret. Time	Area	Height	Height%	Area%
1	21.640	565373	10675	6.348	3.854
2	31.002	14105496	157496	93.652	96.146
Total		14670869	168171	100.000	100.000

Enantiomerically enriched **20**

<u>(*S*, *E*)-2-(2-(2,4-dinitrophenoxy)-7-methoxynaphthalen-1-yl)-3-phenylacrylaldehyde(21)</u>

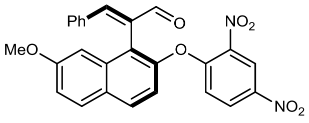

21

A yellow solid, 92% yield, 43.2 mg; m.p: 79-81 °C; **[α]$^{25}_{D}$** = -59.9 (c = 0.25 in $CHCl_3$); **^{1}H NMR (400 MHz, $CDCl_3$)** δ 9.79 (s, 1H), 8.71 (d, *J* = 2.8 Hz, 1H), 8.08 (dd, *J* = 9.3, 2.8 Hz, 1H), 7.95 (d, *J* = 8.8 Hz, 1H), 7.85 (d, *J* = 9.0 Hz, 1H), 7.78 (s, 1H), 7.29 (d, *J* = 7.4 Hz, 1H), 7.20 (dd, *J* = 9.0, 2.5 Hz, 1H), 7.17 – 7.06 (m, 5H), 6.96 (d, *J* = 9.3 Hz, 1H), 6.85 (d, *J* = 2.5 Hz, 1H), 3.74 (s, 3H); **^{13}C NMR (100 MHz, $CDCl_3$)** δ 193.12, 159.46, 155.46, 153.89, 148.94, 141.34, 139.23, 135.05, 134.23, 133.74, 131.23, 131.19, 130.44, 130.33, 128.92, 128.48, 127.35, 122.01, 121.63, 119.39, 118.93, 117.17, 103.61, 55.43; **HRMS (ESI, m/z)** calcd. for $C_{26}H_{18}N_2O_7$ $[M+Na]^+$ = 493.1012, found = 493.1007; **HPLC analysis**: 95% ee (ADH column, 25 °C, n-hexane/*i*-PrOH = 70/30, 1.0 mL/min, λ = 254 nm), Rt (major) = 15.1 min, Rt (minor) = 22.8 min.

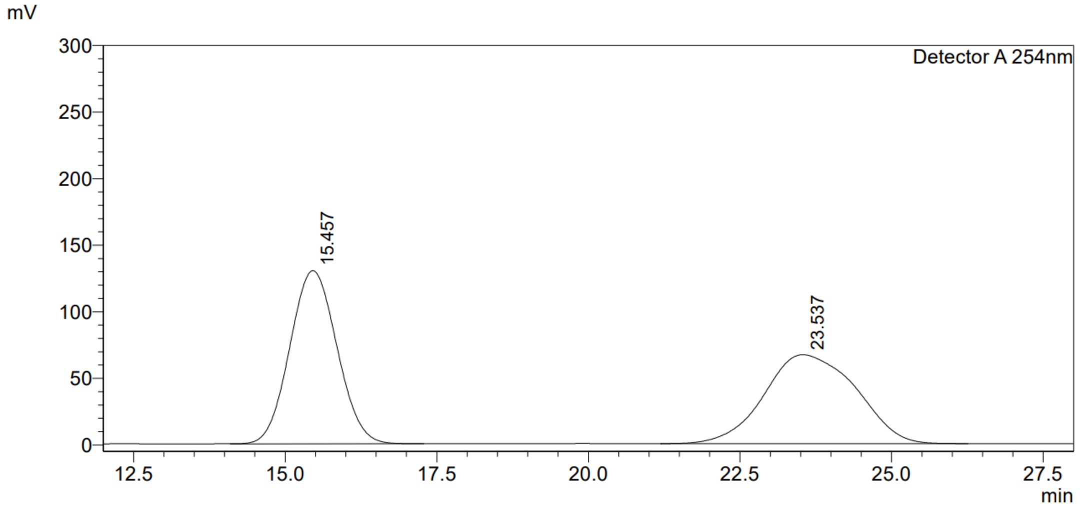

Detector A 254nm

Peak#	Ret. Time	Area	Height	Height%	Area%
1	15.457	7038587	130062	66.075	49.973
2	23.537	7046176	66777	33.925	50.027
Total		14084763	196838	100.000	100.000

Racemic **21**

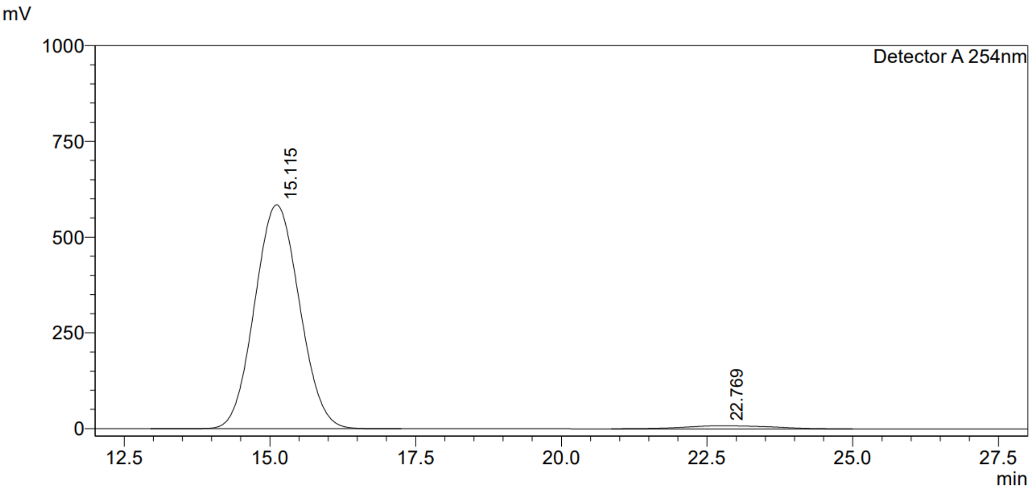

Detector A 254nm

Peak#	Ret. Time	Area	Height	Height%	Area%
1	15.115	31272102	584829	98.640	97.471
2	22.769	811401	8064	1.360	2.529
Total		32083503	592893	100.000	100.000

Enantiomerically enriched **21**

(*S*, *E*)-2-(7-cyclopropyl-2-(2,4-dinitrophenoxy)naphthalen-1-yl)-3-phenylacrylaldehyde (22)

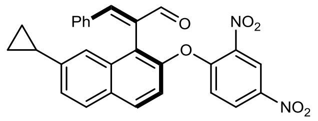

22

A yellow solid, 99% yield, 47.5 mg; m.p: 119-121 ^{o}C; **[α]$^{25}_{D}$** = -17.3 (c = 0.25 in $CHCl_3$); **^{1}H NMR (400 MHz, $CDCl_3$)** δ 9.78 (s, 1H), 8.70 (d, *J* = 2.7 Hz, 1H), 8.07 (dd, *J* = 9.3, 2.8 Hz, 1H), 8.00 – 7.94 (m, 1H), 7.85 (d, *J* = 8.5 Hz, 1H), 7.78 (s, 1H), 7.33 – 7.26 (m, 2H), 7.21 (dd, *J* = 8.6, 1.8 Hz, 1H), 7.19 – 7.11 (m, 3H), 7.09 – 7.05 (m, 2H), 6.92 (d, *J* = 9.3 Hz, 1H), 1.96 (tt, *J* = 8.4, 5.1 Hz, 1H), 0.98 (m, *J* = 8.4, 3.3, 1.6 Hz, 2H), 0.78 – 0.56 (m, 2H); **^{13}C NMR (100 MHz, $CDCl_3$)** δ 193.20, 155.51, 153.98, 148.32, 144.40, 141.30, 139.16, 134.97, 133.78, 132.82, 131.28, 131.12, 130.38, 128.89, 128.85, 128.49, 125.12, 122.59, 121.64, 121.31, 118.81, 118.66, 16.07, 10.00, 9.75; **HRMS (ESI, m/z)** calcd for $C_{28}H_{20}N_2O_6$ $[M+Na]^+$ = 503.1219, found = 503.1211; HPLC analysis: 97% ee (ADH column, 25 ^{o}C, n-hexane/*i*-PrOH = 70/30, 1.0 mL/min, λ = 254 nm), Rt (major) = 13.6 min, Rt (minor) = 16.3 min.

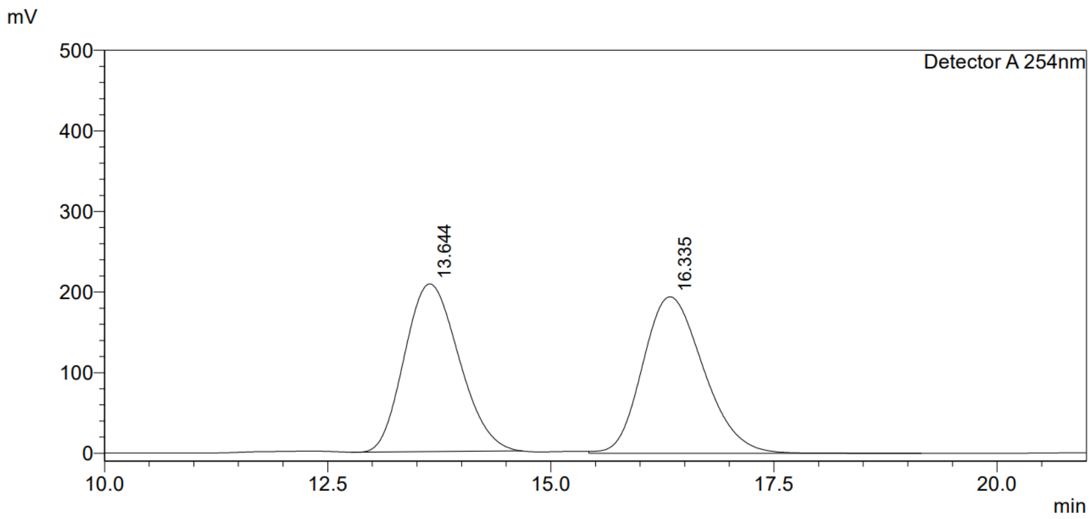

Detector A 254nm

Peak#	Ret. Time	Area	Height	Height%	Area%
1	13.644	8852890	208192	51.734	49.013
2	16.335	9209475	194237	48.266	50.987
Total		18062365	402429	100.000	100.000

Racemic **22**

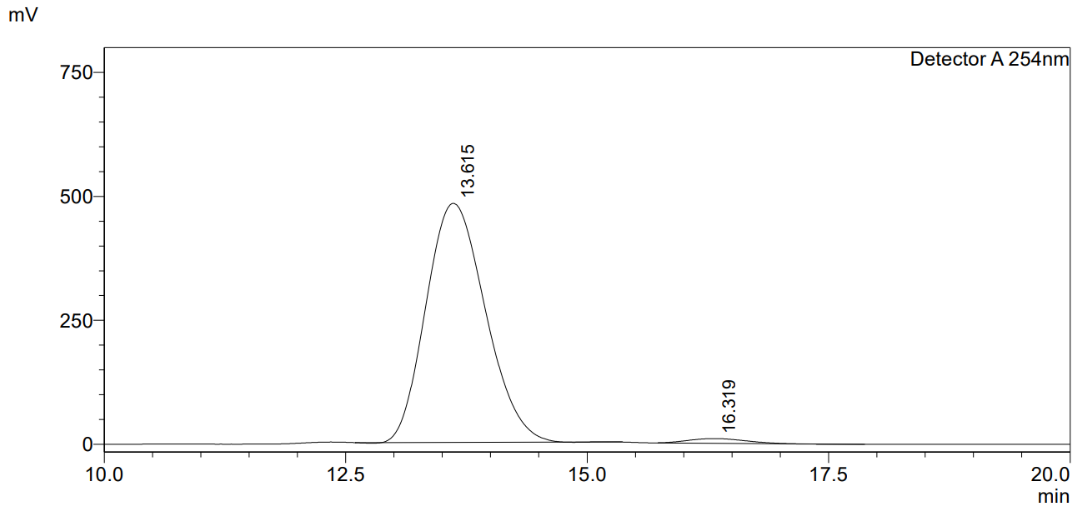

Detector A 254nm

Peak#	Ret. Time	Area	Height	Height%	Area%
1	13.615	20660150	482353	98.090	98.266
2	16.319	364519	9393	1.910	1.734
Total		21024669	491747	100.000	100.000

Enantiomerically enriched **22**

(*S, E*)-2-(2-(2,4-dinitrophenoxy)-7-ethylnaphthalen-1-yl)-3-phenylacrylaldehyde (23)

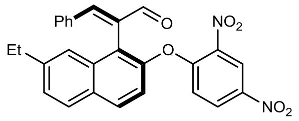

23

A yellow solid, 96% yield, 44.9 mg; m.p: 116-117 ^{o}C; $[\alpha]^{25}_{D}$ = -105.2 (c = 0.25 in

$CHCl_3$); **^{1}H NMR (400 MHz, $CDCl_3$)** δ 9.78 (s, 1H), 8.71 (d, *J* = 2.7 Hz, 1H), 8.07 (dd, *J* = 9.3, 2.8 Hz, 1H), 8.02 – 7.97 (m, 1H), 7.89 (d, *J* = 8.4 Hz, 1H), 7.78 (s, 1H), 7.43 (dd, *J* = 8.4, 1.7 Hz, 1H), 7.38 (dt, *J* = 1.7, 0.8 Hz, 1H), 7.30 – 7.26 (m, 1H), 7.21 (d, *J* = 8.8 Hz, 1H), 7.14 (dd, *J* = 8.6, 7.1 Hz, 2H), 7.09 – 7.04 (m, 2H), 6.93 (d, *J* = 9.3 Hz, 1H), 2.72 (q, *J* = 7.6 Hz, 2H), 1.19 (t, *J* = 7.6 Hz, 3H); **^{13}C NMR (100 MHz, $CDCl_3$)** δ 193.22, 155.54, 154.00, 148.22, 144.50, 141.31, 139.17, 135.02, 133.80, 132.97, 131.31, 131.10, 130.48, 130.37, 128.87, 128.80, 128.49, 128.06, 122.96, 122.88, 121.65, 118.90, 118.81, 29.38, 15.52; **HRMS (ESI, m/z)** calcd for $C_{27}H_{20}N_2O_6$ $[M+Na]^+$ = 491.1219, found = 491.1214; HPLC analysis: 95% ee (ADH column, 25 ^{o}C, n-hexane/*i*-PrOH = 70/30, 1.0 mL / min, λ = 254 nm), Rt (major) = 10.8 min, Rt (minor) = 18.6 min.

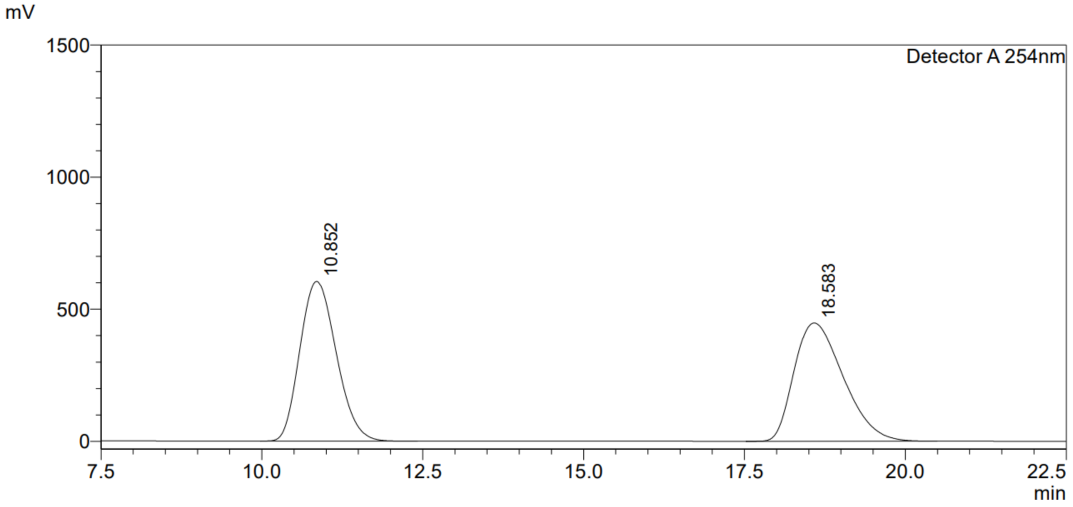

Detector A 254nm

Peak#	Ret. Time	Area	Height	Height%	Area%
1	10.852	23785127	604174	57.438	49.947
2	18.583	23836021	447691	42.562	50.053
Total		47621148	1051865	100.000	100.000

Racemic **23**

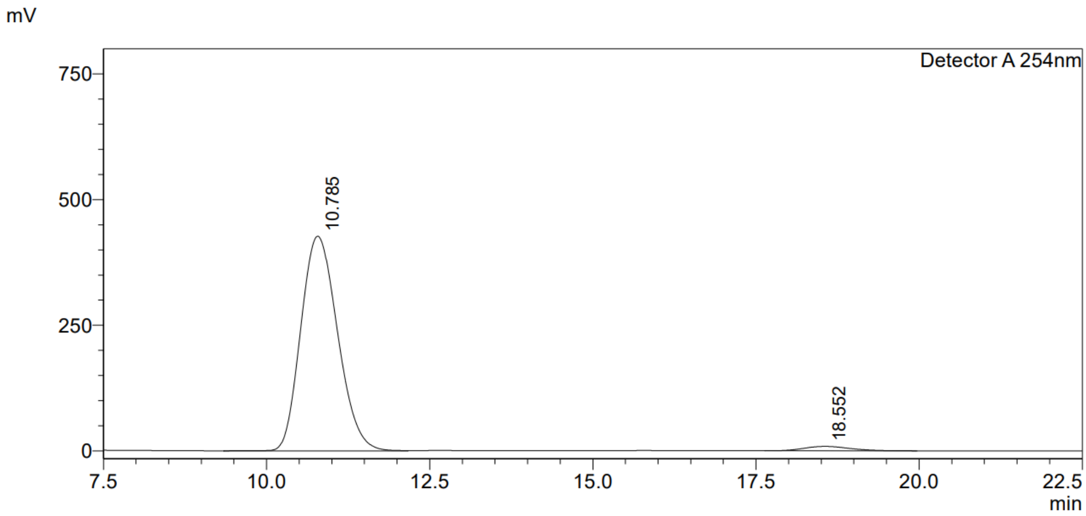

Detector A 254nm

Peak#	Ret. Time	Area	Height	Height%	Area%
1	10.785	16768883	427146	98.001	97.520
2	18.552	426381	8714	1.999	2.480
Total		17195264	435860	100.000	100.000

Enantiomerically enriched **23**

(*S*, *E*)-2-(2-(2,4-dinitrophenoxy)-7-methylnaphthalen-1-yl)-3-phenylacrylaldehyde (24)

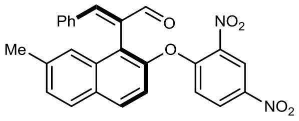

24

A yellow solid, 99% yield, 44.9 mg; m.p: 84-86 °C; $[\alpha]^{25}_{D}$ = -116.2 (c = 0.25 in $CHCl_3$); **^{1}H NMR (400 MHz, $CDCl_3$)** δ 9.70 (s, 1H), 8.62 (d, *J* = 2.7 Hz, 1H), 7.98 (dd, *J* = 9.3, 2.8 Hz, 1H), 7.91 (d, *J* = 8.7 Hz, 1H), 7.81 – 7.77 (m, 1H), 7.70 (s, 1H), 7.32 (d, *J* = 7.6 Hz, 2H), 7.21 (d, *J* = 7.4 Hz, 1H), 7.12 (d, *J* = 8.9 Hz, 1H), 7.07 (t, *J* = 7.8 Hz, 2H), 7.01 – 6.96 (m, 2H), 6.82 (d, *J* = 9.3 Hz, 1H), 2.36 (s, 3H); **^{13}C NMR (100 MHz, $CDCl_3$)** δ 193.25, 155.53, 154.01, 148.14, 141.29, 139.14, 138.33, 135.00, 133.77, 133.06, 131.32, 131.14, 130.40, 130.25, 129.20, 128.89, 128.68, 128.48, 124.10, 122.81, 121.64, 118.90, 118.77, 22.16; **HRMS (ESI, m/z)** calcd for $C_{26}H_{18}N_2O_6$ $[M+Na]^+$ = 477.1063, found = 477.1058; **HPLC analysis**: 96% ee (ADH column, 25 °C, n-hexane/*i*-PrOH = 70/30, 1.0 mL / min, λ = 254 nm), Rt (major) = 12.3 min, Rt (minor) = 17.4 min.

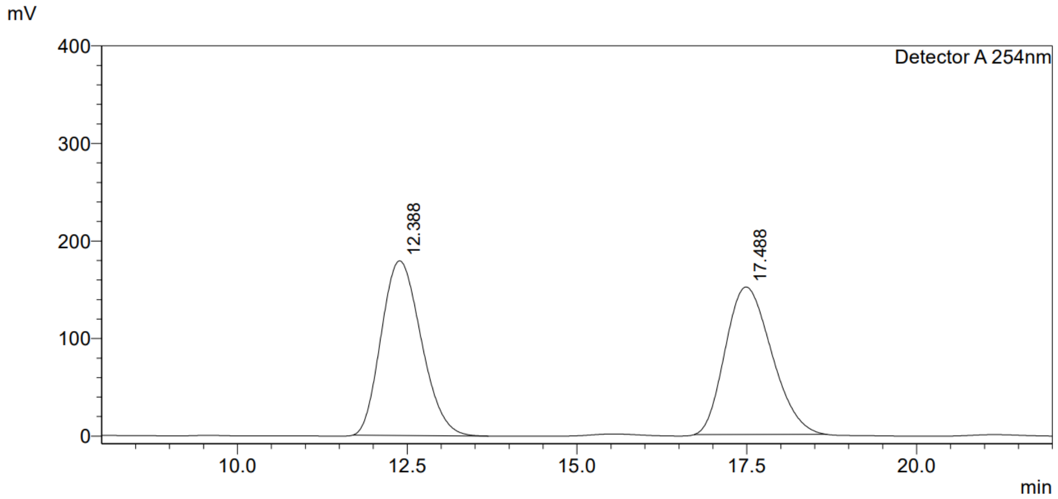

Detector A 254nm

Peak#	Ret. Time	Area	Height	Height%	Area%
1	12.388	7339760	179001	54.212	50.500
2	17.488	7194465	151184	45.788	49.500
Total		14534225	330184	100.000	100.000

Racemic **24**

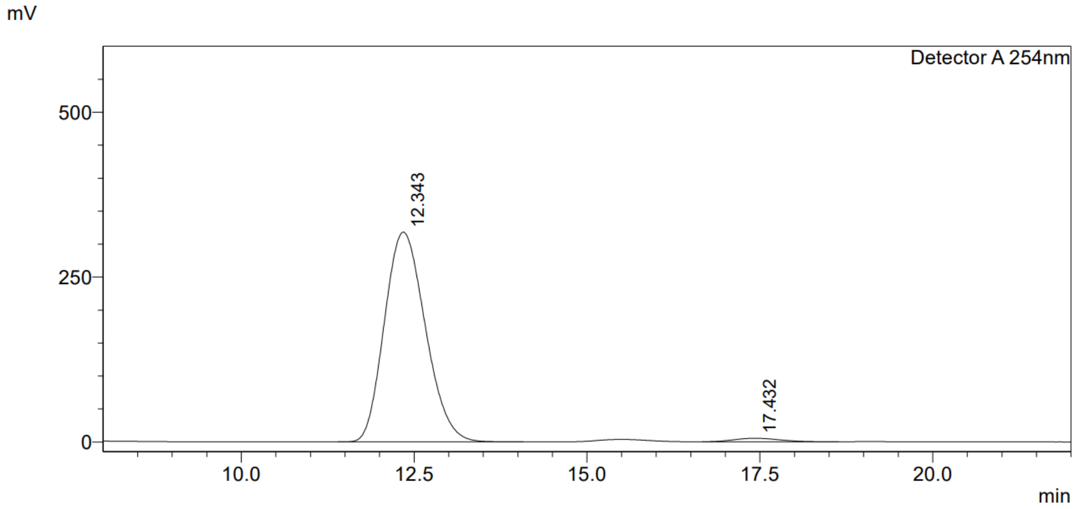

Detector A 254nm

Peak#	Ret. Time	Area	Height	Height%	Area%
1	12.343	13104286	318033	98.329	98.158
2	17.432	245870	5405	1.671	1.842
Total		13350156	323438	100.000	100.000

Enantiomerically enriched **24**

(*S*, *E*)-2-(7-bromo-2-(2,4-dinitrophenoxy)naphthalen-1-yl)-3-phenylacrylaldehyde (25)

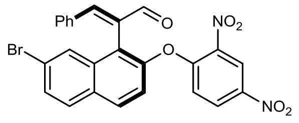

25

A yellow solid, 96% yield, 49.8 mg; m.p: 155-157 ^{o}C; **[α]$^{25}_{D}$** = -40.1 (c = 0.25 in $CHCl_3$); **^{1}H NMR (400 MHz, $CDCl_3$)** δ 9.78 (s, 1H), 8.72 (d, *J* = 2.7 Hz, 1H), 8.11 (dd, *J* = 9.3, 2.7 Hz, 1H), 8.01 (dd, *J* = 9.1, 0.9 Hz, 1H), 7.85 – 7.75 (m, 3H), 7.63 (dd, *J* = 8.7, 1.9 Hz, 1H), 7.33 – 7.27 (m, 2H), 7.17 (dd, *J* = 8.6, 7.1 Hz, 2H), 7.09 – 7.04 (m, 2H), 6.93 (d, *J* = 9.3 Hz, 1H); **^{13}C NMR (100 MHz, $CDCl_3$)** δ 192.98, 155.09, 154.59, 149.16, 141.64, 139.38, 134.15, 133.95, 133.45, 131.57, 131.41, 130.42, 130.39, 130.38, 129.01, 128.60, 127.30, 122.91, 122.81, 121.71, 120.19, 119.04; **HRMS (ESI, m/z)** calcd for $C_{25}H_{15}BrN_2O_6$ $[M+Na]^+$ = 541.0011, found = 541.0007; **HPLC analysis**: 91% ee (IE column, 25 ^{o}C, n-hexane/*i*-PrOH = 70/30, 1.0 mL / min, λ = 254 nm), Rt (major) = 17.9 min, Rt (minor) = 13.6 min.

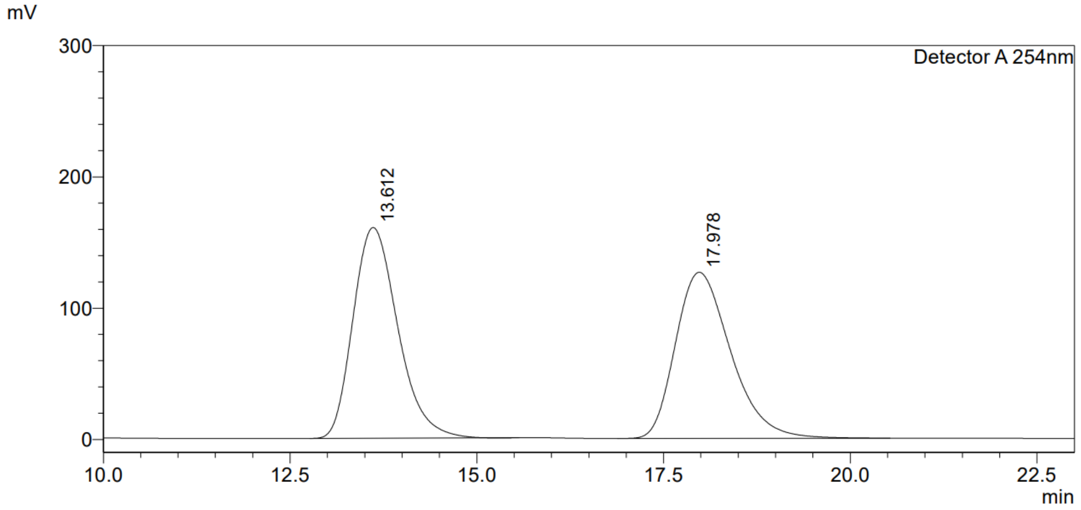

Detector A 254nm

Peak#	Ret. Time	Area	Height	Height%	Area%
1	13.612	6693470	160481	55.921	50.704
2	17.978	6507700	126498	44.079	49.296
Total		13201171	286979	100.000	100.000

Racemic **25**

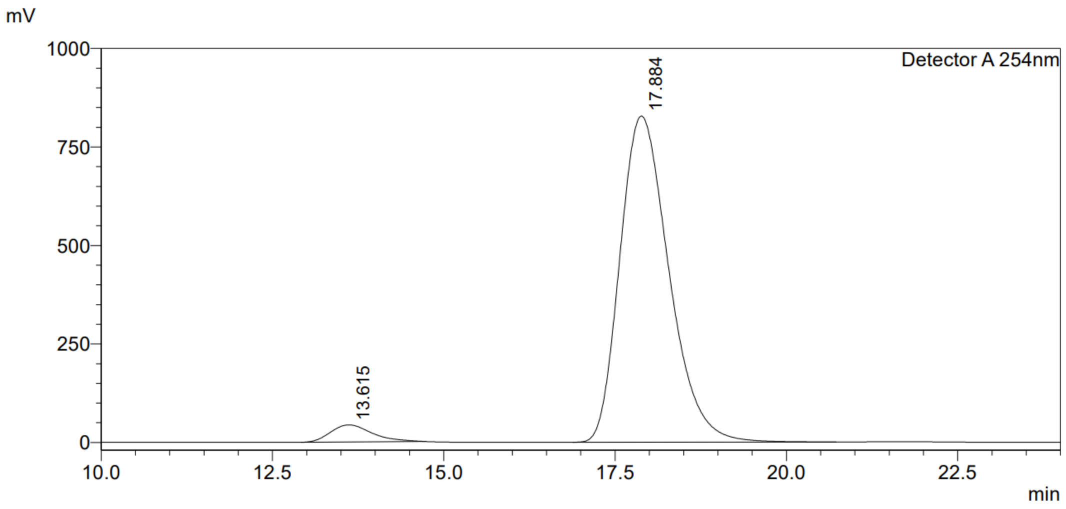

Detector A 254nm

Peak#	Ret. Time	Area	Height	Height%	Area%
1	13.615	1840580	43378	4.979	4.284
2	17.884	41127733	827892	95.021	95.716
Total		42968313	871270	100.000	100.000

Enantiomerically enriched **25**

(*S*, *E*)-2-(6-cyclopropyl-2-(2,4-dinitrophenoxy)naphthalen-1-yl)-3-phenylacrylaldehyde (26)

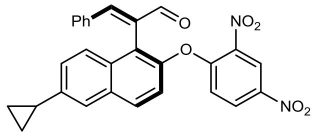

26

A yellow solid, 99% yield, 47.2 mg; m.p: 170-171 ^{o}C; **[α]^{25}$_{D}$** = -92.7 (c = 0.25 in $CHCl_3$); **^{1}H NMR (400 MHz, $CDCl_3$)** δ 9.76 (s, 1H), 8.70 (d, *J* = 2.7 Hz, 1H), 8.07 (dd, *J* = 9.3, 2.8 Hz, 1H), 7.97 – 7.90 (m, 1H), 7.76 (s, 1H), 7.65 (d, *J* = 1.8 Hz, 1H), 7.52 (d, *J* = 8.7 Hz, 1H), 7.30 – 7.26 (m, 1H), 7.23 (d, *J* = 8.9 Hz, 1H), 7.20 (dd, *J* = 8.7, 1.9 Hz, 1H), 7.14 (dd, *J* = 8.6, 7.1 Hz, 2H), 7.08 – 7.03 (m, 2H), 6.91 (d, *J* = 9.3 Hz, 1H), 2.07 (tt, *J* = 8.4, 5.0 Hz, 1H), 1.06 (m, *J* = 8.3, 2.7, 1.0 Hz, 2H), 0.86 – 0.78 (m, 2H); **^{13}C NMR (100 MHz, $CDCl_3$)** δ 193.15, 155.59, 153.98, 147.31, 142.89, 141.27, 139.12, 134.93, 133.71, 132.19, 131.15, 130.97, 130.87, 130.41, 128.90, 128.50, 126.95, 125.11, 124.74, 123.29, 121.64, 119.97, 118.75, 15.66, 9.64, 9.54; **HRMS (ESI, m/z)** calcd for $C_{28}H_{20}N_2O_6$ $[M+Na]^+$ = 503.1219, found: 503.1214; HPLC analysis: 94% ee (ADH column, 25 ^{o}C, n-hexane/*i*-PrOH = 70/30, 1.0 mL/min, λ = 254 nm), Rt (major) = 18.9 min, Rt (minor) = 40.0 min.

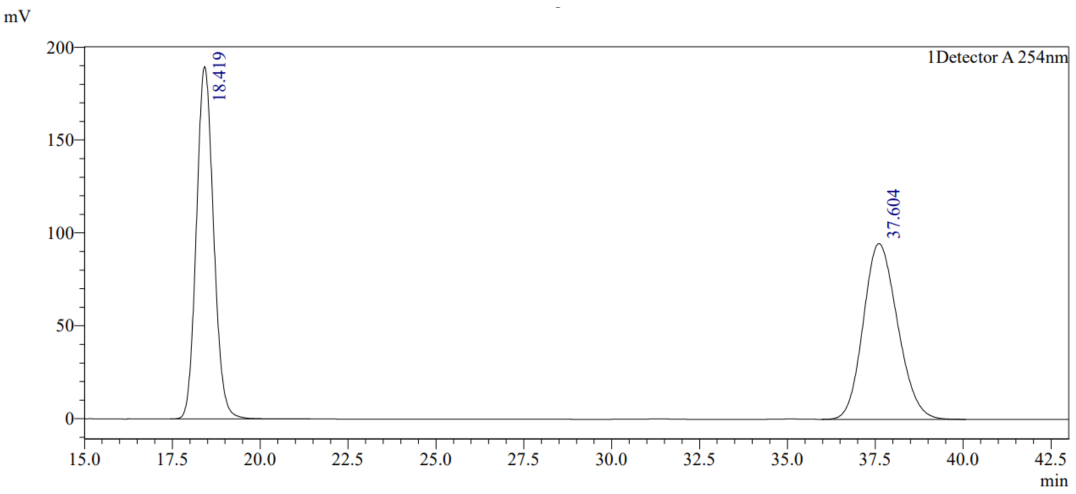

Peak Table

Detector A 254nm

Peak#	Ret. Time	Height	Height%	Area	Area%
1	18.419	189721	66.726	6459017	50.115
2	37.604	94606	33.274	6429409	49.885
Total		284327	100.000	12888427	100.000

Racemic **26**

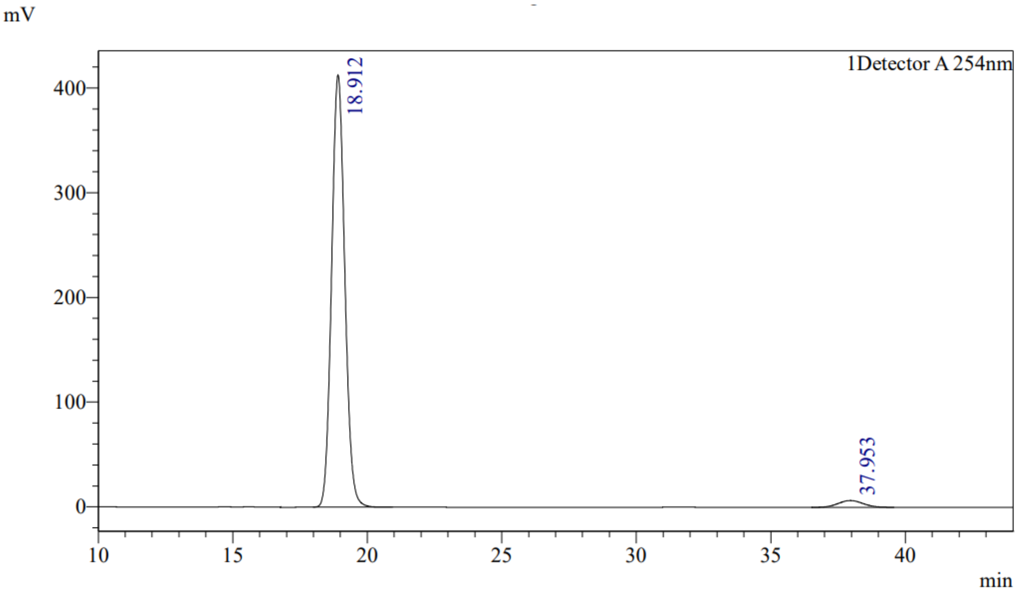

Peak Table

Detector A 254nm

Peak#	Ret. Time	Area	Height	Height%	Area%
1	18.912	14048680	412719	98.448	97.080
2	37.953	422605	6505	1.552	2.920
Total		14471285	419225	100.000	100.000

Enantiomerically enriched **26**

(*S*, *E*)-2-(2-(2,4-dinitrophenoxy)-6-ethylnaphthalen-1-yl)-3-phenylacrylaldehyde (27)

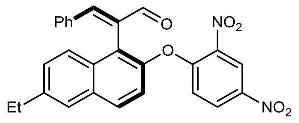

27

A yellow solid, 99% yield, 46.3 mg; m.p: 145-156 °C; **[α]^{25}$_{D}$** = -97.9 (c = 0.25 in $CHCl_3$); **^{1}H NMR (400 MHz, $CDCl_3$)** δ 9.77 (s, 1H), 8.70 (d, *J* = 2.7 Hz, 1H), 8.07 (dd, *J* = 9.3, 2.8 Hz, 1H), 8.01 – 7.94 (m, 1H), 7.79 – 7.73 (m, 2H), 7.56 (d, *J* = 8.6 Hz, 1H), 7.37 (dd, *J* = 8.6, 1.8 Hz, 1H), 7.30 – 7.26 (m, 1H), 7.24 (s, 1H), 7.14 (dd, *J* = 8.6, 7.1 Hz, 2H), 7.07 (dd, *J* = 8.4, 1.3 Hz, 2H), 6.92 (d, *J* = 9.3 Hz, 1H), 2.83 (q, *J* = 7.6 Hz, 2H), 1.34 (t, *J* = 7.6 Hz, 3H); **^{13}C NMR (100 MHz, $CDCl_3$)** δ 193.18, 155.60, 154.01, 147.48, 142.97, 141.28, 139.13, 134.96, 133.73, 132.32, 131.15, 131.11, 130.42, 129.50, 128.90, 128.50, 126.59, 125.08, 123.28, 121.65, 119.83, 118.76, 28.95, 15.37; **HRMS (ESI, m/z)** calcd for $C_{27}H_{20}N_2O_6$ $[M+Na]^+$ = 491.1219, found = 491.1210; HPLC analysis: 94% ee (ADH column, 25 °C, n-hexane/*i*-PrOH = 70/30, 1.0 mL/min, λ = 254 nm), Rt (major) = 12.7 min, Rt (minor) = 27.1 min.

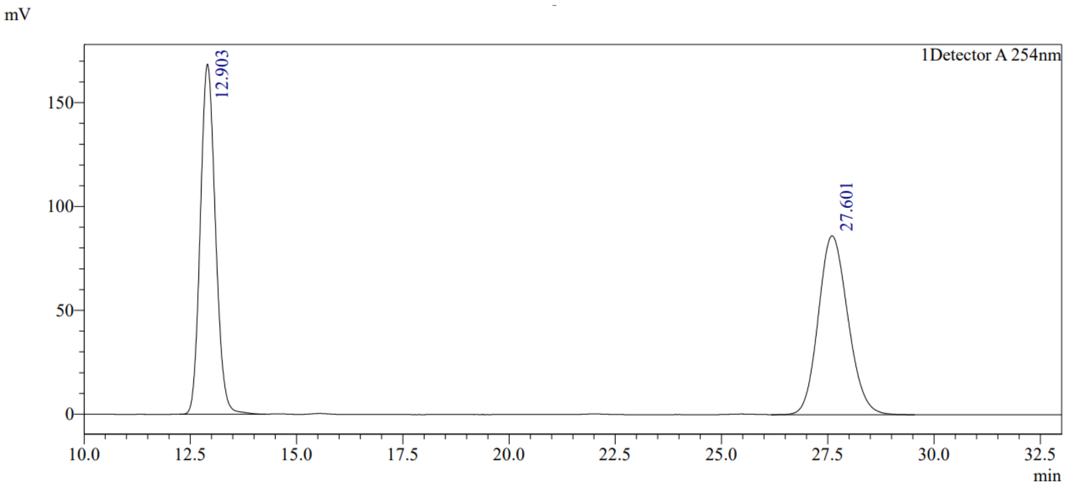

Peak Table

Detector A 254nm

Peak#	Ret. Time	Height	Height%	Area	Area%
1	12.903	168577	66.179	4170329	49.940
2	27.601	86152	33.821	4180290	50.060
Total		254729	100.000	8350620	100.000

Racemic **27**

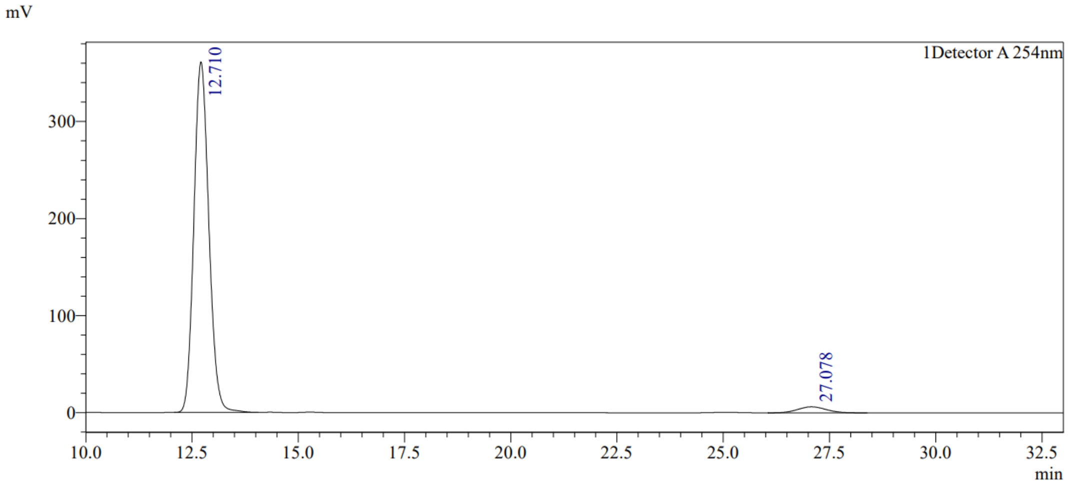

Peak Table

Detector A 254nm

Peak#	Ret. Time	Height	Height%	Area	Area%
1	12.710	361336	98.315	8841156	96.820
2	27.078	6193	1.685	290406	3.180
Total		367530	100.000	9131562	100.000

Enantiomerically enriched **27**

(*S*, *E*)-2-(2-(2,4-dinitrophenoxy)-6-methylnaphthalen-1-yl)-3-phenylacrylaldehyde (28)

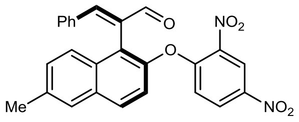

28

A yellow solid, 99% yield, 44.8 mg; m.p: 222-223 ^{o}C; **[α]^{25}$_{D}$** = -126.2 (c = 0.25 in $CHCl_3$).; **^{1}H NMR (600 MHz, DMSO-*d*$_6$)** δ 9.78 (s, 1H), 8.77 (d, *J* = 2.8 Hz, 1H), 8.16 (dd, *J* = 9.3, 2.8 Hz, 1H), 8.11 (d, *J* = 9.0 Hz, 1H), 8.06 (s, 1H), 7.88 (s, 1H), 7.53 (t, *J* = 8.3 Hz, 2H), 7.37 (d, *J* = 8.6 Hz, 1H), 7.28 (t, *J* = 7.4 Hz, 1H), 7.16 (t, *J* = 7.8 Hz, 2H), 7.04 (d, *J* = 7.6 Hz, 2H), 6.90 (d, *J* = 9.3 Hz, 1H), 2.48 (s, 3H); **^{13}C NMR (151 MHz, DMSO-*d*$_6$)** δ 193.79, 154.37, 153.40, 147.29, 140.95, 138.87, 136.03, 134.57, 133.64, 131.64, 130.82, 130.59, 130.24, 130.08, 129.93, 128.86, 128.75, 127.62, 124.63, 122.85, 121.42, 120.39, 118.49, 21.08; **HRMS (ESI, m/z)** calcd. for $C_{26}H_{18}N_2O_6$ $[M+Na]^+$ = 477.1063, found = 477.1059; **HPLC analysis**: 96% ee (ADH column, 25 ^{o}C, n-hexane/*i*-PrOH = 70/30, 1.0 mL/min, λ = 254 nm), Rt (major) = 14.3 min, Rt (minor) = 24.4 min.

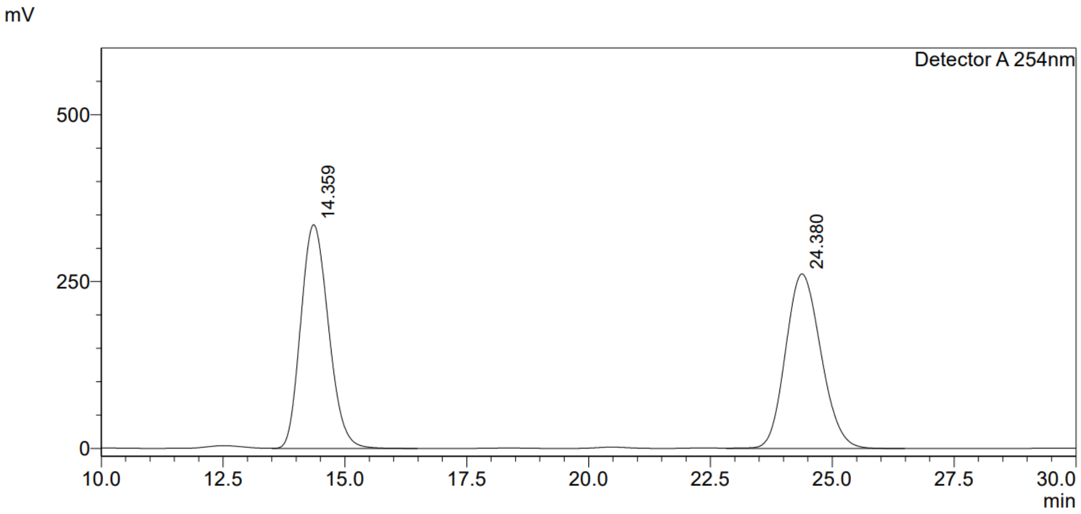

Detector A 254nm

Peak#	Ret. Time	Area	Height	Height%	Area%
1	14.359	13274456	335275	56.179	50.086
2	24.380	13229061	261526	43.821	49.914
Total		26503517	596800	100.000	100.000

Racemic **28**

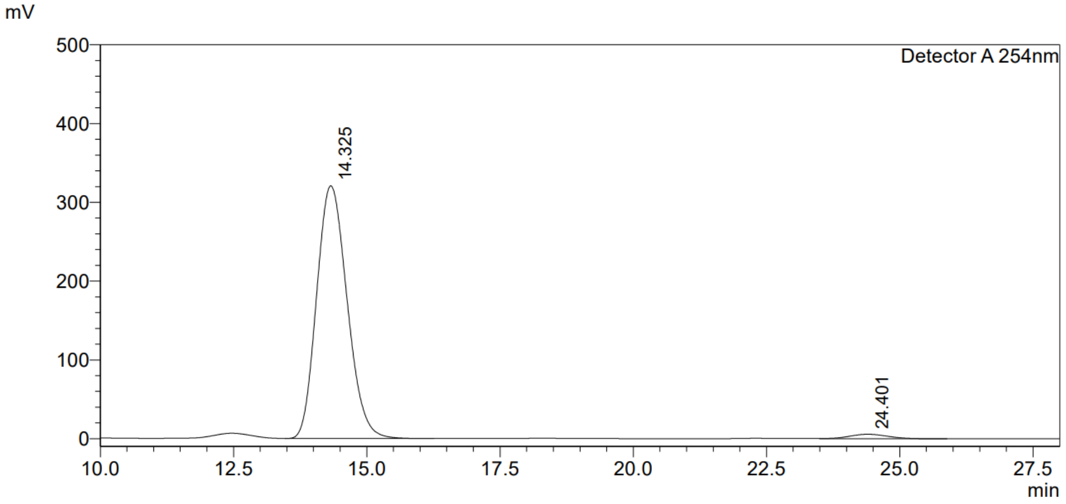

Detector A 254nm

Peak#	Ret. Time	Area	Height	Height%	Area%
1	14.325	12769200	320875	98.259	97.788
2	24.401	288810	5685	1.741	2.212
Total		13058010	326560	100.000	100.000

Enantiomerically enriched **28**

(*S*, *E*)-2-(6-bromo-2-(2,4-dinitrophenoxy)naphthalen-1-yl)-3-phenylacrylaldehyde (29)

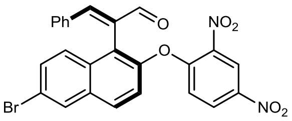

29

A yellow solid, 98% yield, 50.8 mg; m.p: 221-222 °C; **[α]$^{25}_{D}$** = -85.8 (c = 0.25 in $CHCl_3$); **^{1}H NMR (400 MHz, DMSO-*d*$_6$)** δ 9.77 (s, 1H), 8.76 (d, *J* = 2.8 Hz, 1H), 8.40 (d, *J* = 1.9 Hz, 1H), 8.23 – 8.14 (m, 2H), 8.08 (s, 1H), 7.63 (dd, *J* = 8.9, 2.3 Hz, 2H), 7.55 (d, *J* = 9.0 Hz, 1H), 7.28 (t, *J* = 7.4 Hz, 1H), 7.16 (t, *J* = 7.7 Hz, 2H), 7.02 (d, *J* = 7.4 Hz, 2H), 6.91 (d, *J* = 9.3 Hz, 1H); **^{13}C NMR (100 MHz, DMSO-*d*$_6$)** δ 193.66, 153.98, 153.72, 148.45, 141.18, 138.99, 133.88, 133.45, 132.62, 130.94, 130.81, 130.69, 130.61, 129.93, 128.94, 128.82, 127.05, 123.22, 121.61, 121.47, 119.79, 118.70; **HRMS (ESI, m/z)** calcd. for $C_{25}H_{15}BrN_2O_6$ $[M+Na]^+$ = 541.0011, found = 541.0013; HPLC analysis: 89% ee (ADH column, 25 °C, n-hexane/*i*-PrOH = 70/30, 1.0 mL/min, λ = 254 nm), Rt (major) = 22.5 min, Rt (minor) = 35.9 min.

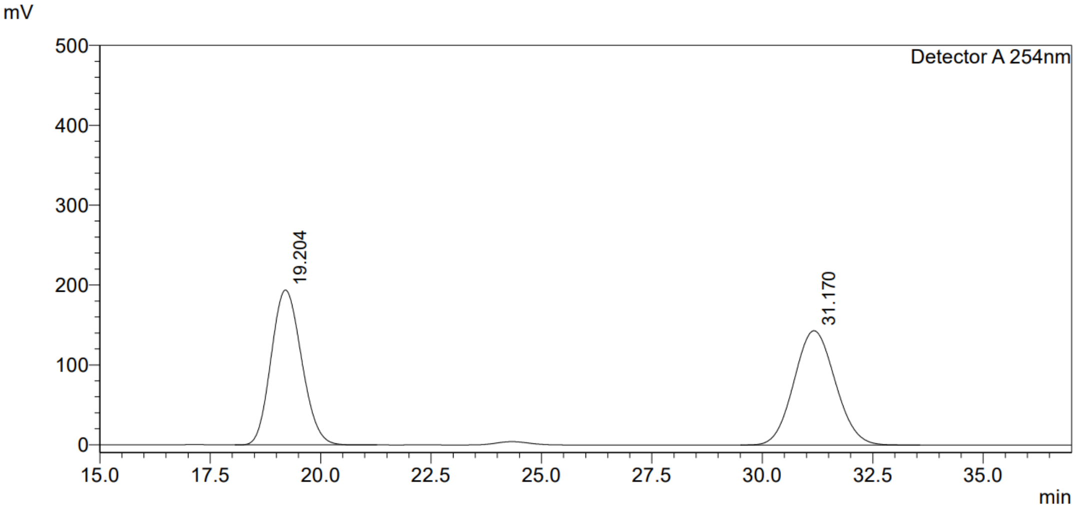

Detector A 254nm

Peak#	Ret. Time	Area	Height	Height%	Area%
1	19.204	9320570	193956	57.538	50.017
2	31.170	9314278	143133	42.462	49.983
Total		18634848	337089	100.000	100.000

Racemic **29**

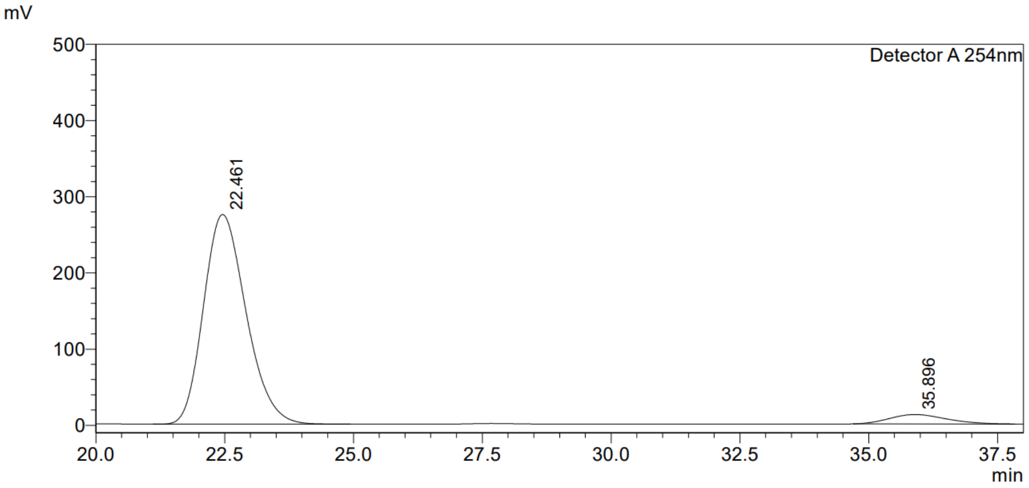

Detector A 254nm

Peak#	Ret. Time	Area	Height	Height%	Area%
1	22.461	15806814	275184	95.709	94.318
2	35.896	952172	12339	4.291	5.682
Total		16758986	287523	100.000	100.000

Enantiomerically enriched **29**

Methyl (*S*, *E*)-6-(2,4-dinitrophenoxy)-5-(3-oxo-1-phenylprop-1-en-2-yl)-2-naphthoate (30)

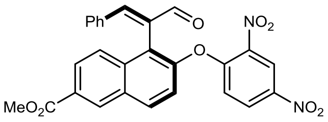

30

A yellow solid, 99% yield, 49.2 mg; m.p: 84-86 °C; **[α]^{25}$_D$** = -67.1 (c = 0.25 in $CHCl_3$); **^{1}H NMR (400 MHz, $CDCl_3$)** δ 9.80 (s, 1H), 8.76 – 8.70 (m, 2H), 8.20 – 8.11 (m, 2H), 8.05 (dd, *J* = 8.8, 1.7 Hz, 1H), 7.83 (s, 1H), 7.67 (dt, *J* = 8.9, 0.8 Hz, 1H), 7.33 (d, *J* = 8.9 Hz, 1H), 7.29 (d, *J* = 7.4 Hz, 1H), 7.14 (dd, *J* = 8.6, 7.1 Hz, 2H), 7.07 – 7.03 (m, 2H), 7.00 (d, *J* = 9.3 Hz, 1H), 3.99 (s, 3H); **^{13}C NMR (100 MHz, $CDCl_3$)** δ 192.98, 166.76, 154.97, 154.39, 150.34, 141.81, 139.57, 134.94, 134.38, 133.48, 132.96, 131.74, 131.38, 131.08, 130.38, 129.01, 128.66, 128.43, 127.56, 125.47, 123.48, 121.76, 120.50, 119.32, 52.59; **HRMS (ESI, m/z)** calcd for $C_{27}H_{18}N_2O_8$ $[M+Na]^+$ = 521.0961, found = 521.0963; **HPLC analysis**: 83% ee (IE column, 25 °C, n-hexane/*i*-PrOH = 70/30, 1.0 mL/min, λ = 254 nm), Rt (major) = 38.6 min, Rt (minor) = 28.2 min.

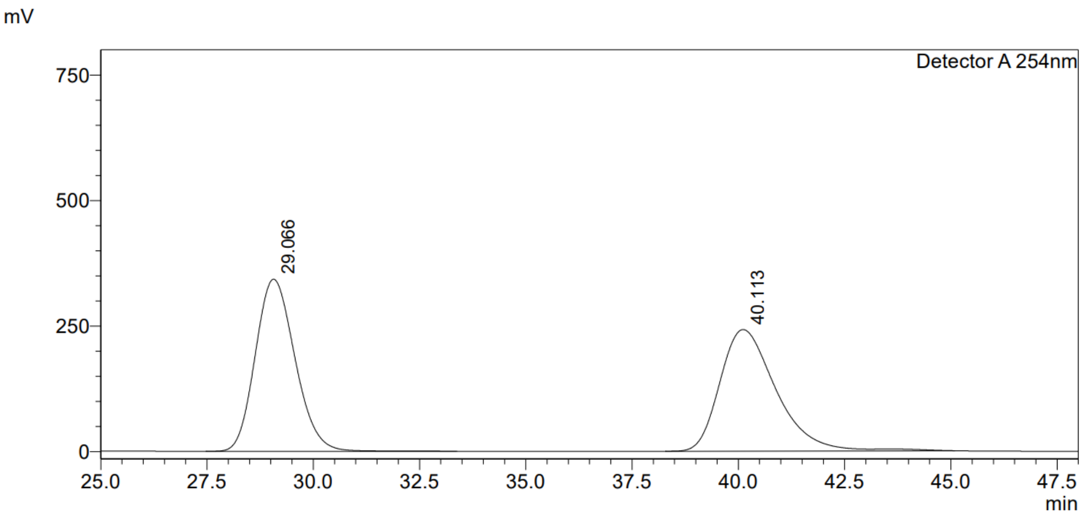

Detector A 254nm

Peak#	Ret. Time	Area	Height	Height%	Area%
1	29.066	22258031	342862	58.610	49.936
2	40.113	22314996	242129	41.390	50.064
Total		44573027	584991	100.000	100.000

Racemic **30**

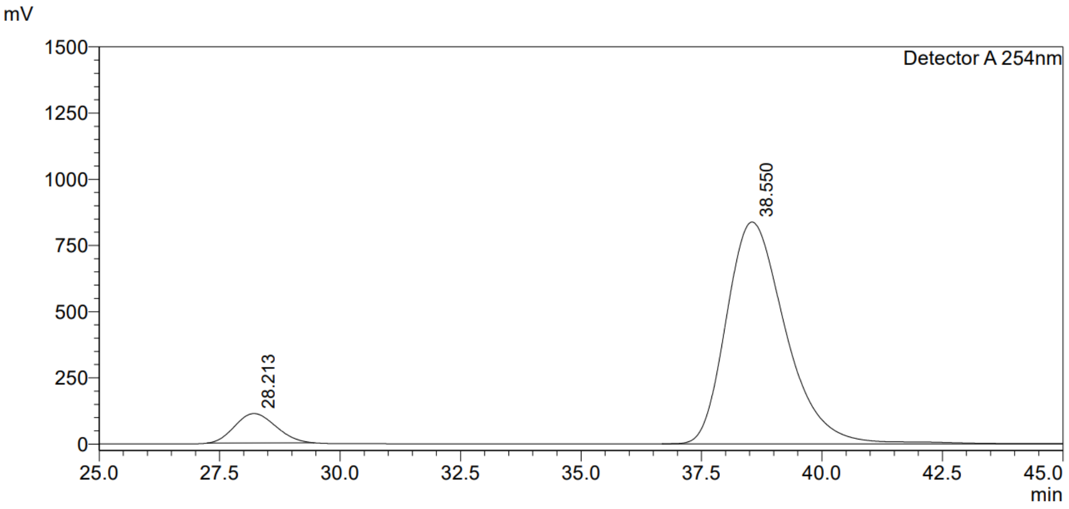

Detector A 254nm

Peak#	Ret. Time	Area	Height	Height%	Area%
1	28.213	6576337	111104	11.711	8.393
2	38.550	71782630	837583	88.289	91.607
Total		78358968	948688	100.000	100.000

Enantiomerically enriched **30**

<u>(*S*, *E*)-2-(6-(2,4-dinitrophenoxy)quinolin-5-yl)-3-phenylacrylaldehyde (31)</u>

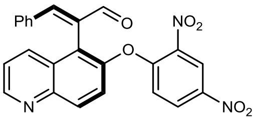

31

A yellow solid, 92% yield, 40.6 mg; m.p: 150-152 °C; **[α]^{25}$_D$** = -242.3 (c = 0.25 in $CHCl_3$); **^{1}H NMR (400 MHz, $CDCl_3$)** δ 9.78 (s, 1H), 8.97 (dd, *J* = 4.2, 1.7 Hz, 1H),

8.74 (d, *J* = 2.7 Hz, 1H), 8.31 (dd, *J* = 9.2, 0.8 Hz, 1H), 8.17 (dd, *J* = 9.3, 2.8 Hz, 1H), 7.93 (ddd, *J* = 8.5, 1.7, 0.9 Hz, 1H), 7.83 (s, 1H), 7.52 (d, *J* = 9.2 Hz, 1H), 7.40 (dd, *J* = 8.5, 4.2 Hz, 1H), 7.33 – 7.27 (m, 1H), 7.16 (dd, *J* = 8.5, 7.1 Hz, 2H), 7.08 – 6.99 (m, 3H); **^{13}C NMR (100 MHz, $CDCl_3$)** δ 192.87, 154.98, 154.72, 151.13, 148.70, 146.71, 141.80, 139.51, 133.70, 133.43, 133.30, 133.18, 131.54, 130.43, 129.10, 128.71, 127.89, 123.53, 123.28, 122.69, 121.78, 119.11; **HRMS (ESI, m/z)** calcd. for $C_{24}H_{15}N_3O_6$ $[M+H]^+$ = 442.1039, found = 442.1042; HPLC analysis: 92% ee (ADH column, 25 ^{o}C, n-hexane/*i*-PrOH = 70/30, 1.0 mL/min, λ = 254 nm), Rt (major) = 19.3 min, Rt (minor) = 33.4 min.

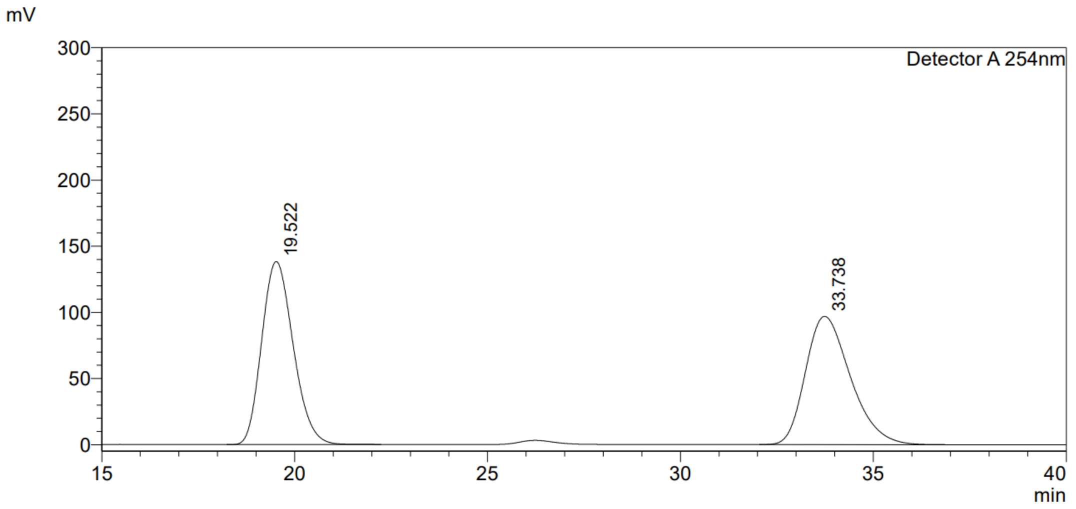

Detector A 254nm

Peak#	Ret. Time	Area	Height	Height%	Area%
1	19.522	7858836	138288	58.801	50.015
2	33.738	7854177	96893	41.199	49.985
Total		15713013	235181	100.000	100.000

Racemic **31**

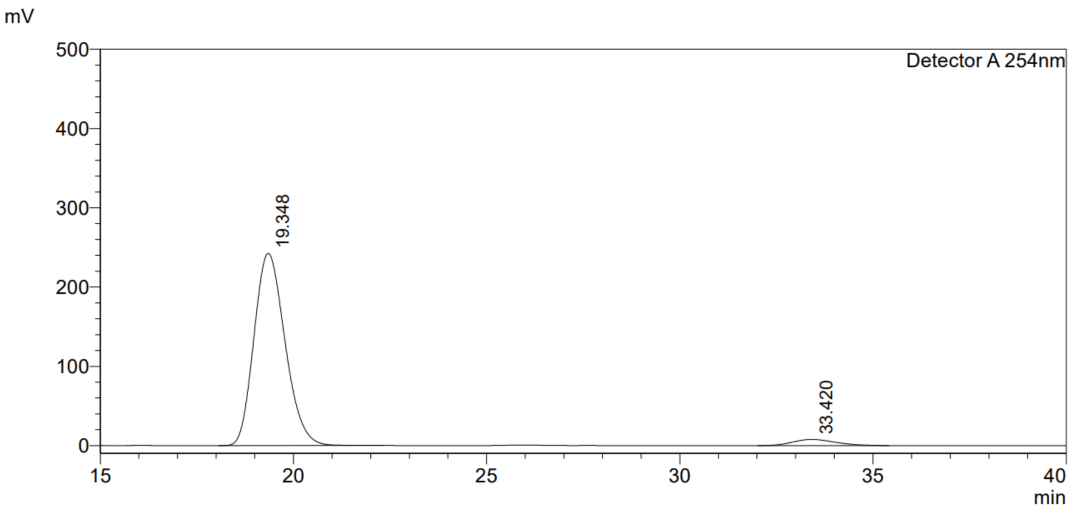

Detector A 254nm

Peak#	Ret. Time	Area	Height	Height%	Area%
1	19.348	13548633	242435	96.895	95.760
2	33.420	599826	7768	3.105	4.240
Total		14148459	250202	100.000	100.000

Enantiomerically enriched **31**

***(S, E)*-2-(2-(5-methyl-2,4-dinitrophenoxy)naphthalen-1-yl)-3-phenylacrylaldehyde (32)**

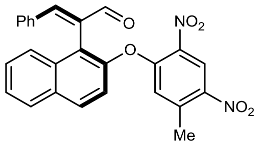

32

A yellow solid, 99% yield, 44.9 mg; m.p: 83-85 ^{o}C; **[α]$^{25}_{D}$** = -134.0 (c = 0.25 in $CHCl_3$); **^{1}H NMR (400 MHz, $CDCl_3$)** δ 9.78 (s, 1H), 8.64 (s, 1H), 8.05 (d, *J* = 8.9 Hz, 1H), 8.01 – 7.95 (m, 1H), 7.77 (s, 1H), 7.64 (dd, *J* = 8.3, 1.3 Hz, 1H), 7.60 – 7.47 (m, 2H), 7.32 – 7.26 (m, 2H), 7.19 – 7.11 (m, 2H), 7.10 – 7.03 (m, 2H), 6.71 (s, 1H), 2.41 (s, 3H); **^{13}C NMR (100 MHz, $CDCl_3$)** δ 193.16, 153.99, 153.70, 148.32, 142.09, 141.59, 137.18, 134.93, 133.68, 132.72, 131.93, 131.62, 131.18, 130.41, 128.88, 128.16, 126.78, 125.12, 123.44, 123.35, 121.95, 120.04, 21.36; **HRMS (ESI, m/z)** calcd for $C_{26}H_{18}N_2O_6$ $[M+Na]^+$ = 477.1063, found = 477.1060; HPLC analysis: 93% ee (IE column, 25 ^{o}C, n-hexane/*i*-PrOH = 70/30, 1.0 mL/min, λ = 254 nm), Rt (major) = 20.5 min, Rt (minor) = 14.1 min.

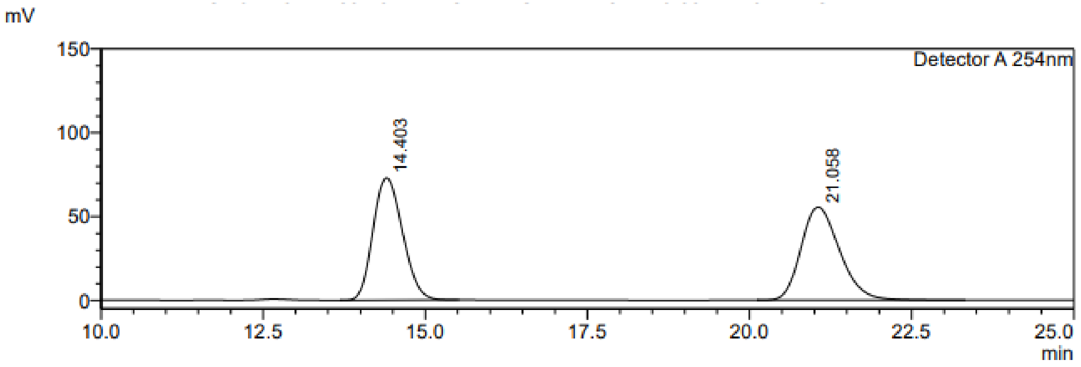

Detector A 254nm

Peak#	Ret. Time	Height	Height%	Area	Area%
1	14.403	72986	56.774	2318568	50.013
2	21.058	55569	43.226	2317400	49.987
Total		128554	100.000	4635968	100.000

Racemic **32**

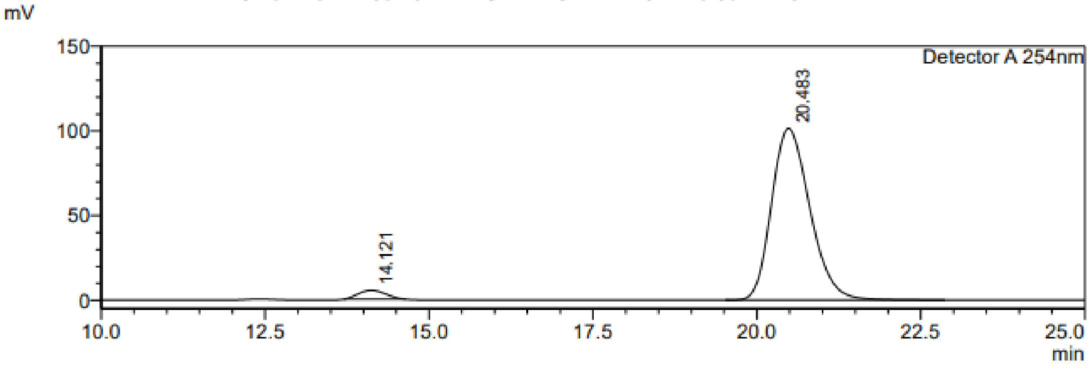

Detector A 254nm

Peak#	Ret. Time	Height	Height%	Area	Area%
1	14.121	5282	4.952	152565	3.562
2	20.483	101377	95.048	4129989	96.438
Total		106659	100.000	4282554	100.000

Enantiomerically enriched **32**

(*S*, *E*)-2-(2-((2,4-dinitronaphthalen-1-yl)oxy)naphthalen-1-yl)-3-phenylacrylaldehyde (33)

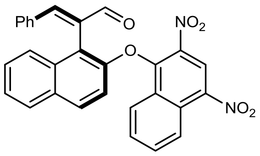

33

A yellow solid, 99% yield, 48 mg; m.p:104-106 ^{o}C; **$[\alpha]^{25}_{D}$** = -61.6 (c = 0.25 in $CHCl_3$); **^{1}H NMR (400 MHz, $CDCl_3$)** δ 10.03 (s, 1H), 8.84 (s, 1H), 8.66 (dd, *J* = 8.8, 1.0 Hz, 1H), 7.95 (s, 1H), 7.92 – 7.80 (m, 2H), 7.76 (d, *J* = 9.0 Hz, 1H), 7.69 – 7.53 (m, 2H), 7.43 (dd, *J* = 6.3, 3.1 Hz, 2H), 7.37 – 7.24 (m, 6H), 6.61 (d, *J* = 9.1 Hz, 1H); **^{13}C**

NMR (100 MHz, CDCl$_3$) δ 193.90, 149.39, 143.31, 135.38, 133.94, 133.23, 131.17, 131.06, 130.97, 130.38, 129.89, 129.78, 128.97, 128.75, 128.14, 128.09, 126.06, 125.46, 124.47, 123.88, 119.98, 113.52; **HRMS (ESI, m/z)** calcd for $C_{29}H_{18}N_2O_6$ $[M+Na]^+$ = 513.1063, found: 513.1061; **HPLC analysis**: 92% ee (ADH column, 25 ^{o}C, n-hexane / *i*-PrOH = 70/30, 1.0 mL/min, λ = 254 nm), Rt (major) = 28.1 min, Rt (minor) = 20.5 min.

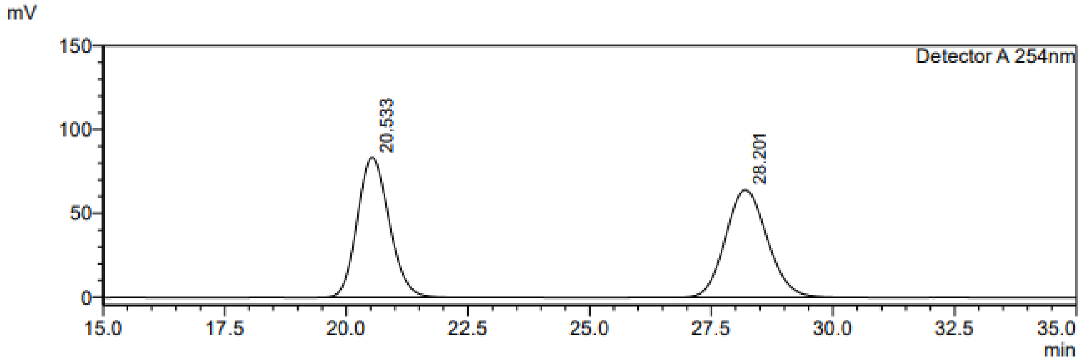

Detector A 254nm

Peak#	Ret. Time	Height	Height%	Area	Area%
1	20.533	83509	56.554	3824013	49.972
2	28.201	64153	43.446	3828335	50.028
Total		147661	100.000	7652348	100.000

Racemic **33**

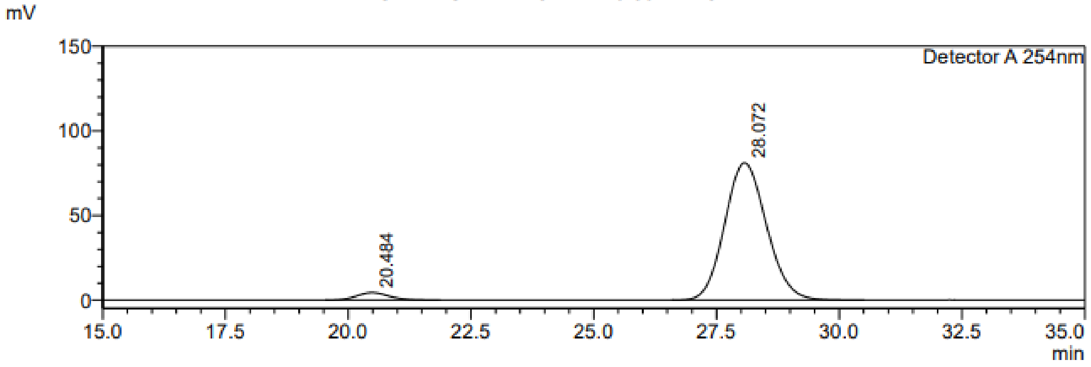

Detector A 254nm

Peak#	Ret. Time	Height	Height%	Area	Area%
1	20.484	4430	5.177	202710	4.029
2	28.072	81139	94.823	4829120	95.971
Total		85569	100.000	5031830	100.000

Enantiomerically enriched **33**

(2*S*, 2’*S*, 2*E*, 2’*E*)-2,2'-(((4,6-dinitro-1,3-phenylene)bis(oxy))bis(naphthalene-2,1-diyl))bis(3-phenylacrylaldehyde) (34)

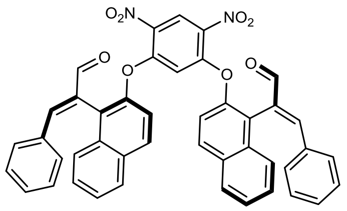

34

A yellow solid, 87% yield, 62.0 mg; m.p: 177-178 °C; **[α]$^{25}_{D}$** = -323.9 (c = 0.25 in $CHCl_3$); **^{1}H NMR (400 MHz, $CDCl_3$)** δ 9.75 (s, 2H), 8.65 (s, 1H), 7.77 (s, 2H), 7.74 (d, *J* = 7.9 Hz, 2H), 7.66 (d, *J* = 9.0 Hz, 2H), 7.54 (d, *J* = 8.3 Hz, 2H), 7.47 – 7.42 (m, 2H), 7.42 – 7.37 (m, 2H), 7.24 (d, *J* = 9.0 Hz, 2H), 7.22 – 7.16 (m, 2H), 7.11 – 7.04 (m, 8H), 6.57 (s, 1H); **^{13}C NMR (100 MHz, $CDCl_3$)** δ 192.86, 155.45, 153.90, 148.36, 134.84, 133.71, 133.01, 132.14, 131.71, 131.01, 130.98, 130.47, 128.80, 128.75, 127.62, 126.22, 124.97, 124.79, 122.49, 119.85, 108.08; **HRMS (ESI, m/z)** calcd for $C_{44}H_{28}N_2O_8$ $[M+Na]^+$ = 735.1744, found = 735.1748; HPLC analysis: >99% ee (ADH column, 25 °C, n-hexane/*i*-PrOH = 70/30, 1.0 mL/min, λ = 254 nm), Rt (major) = 29.6 min, Rt (minor) = 58.4 min.

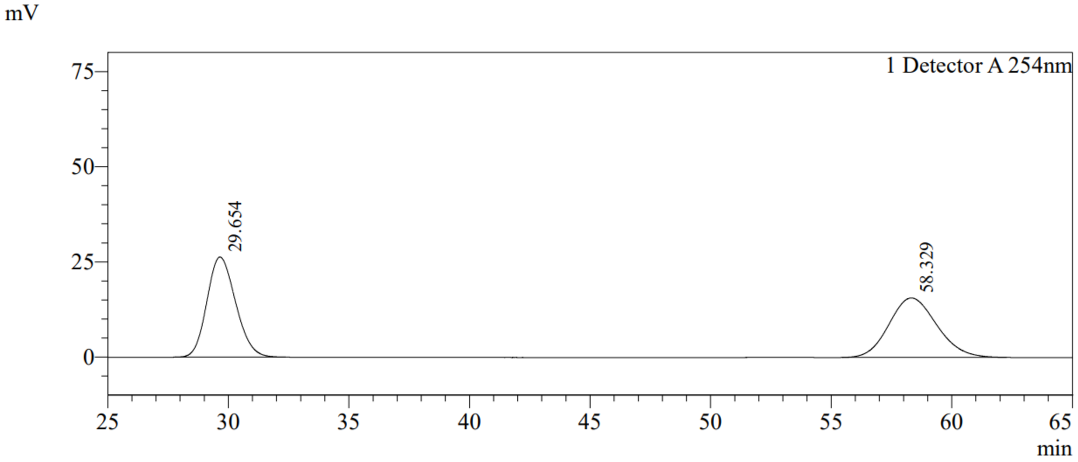

Peak Table

Detector A 254nm

Peak#	Ret. Time	Height	Height%	Area	Area%
1	29.654	26294	62.747	2177391	50.294
2	58.329	15611	37.253	2151896	49.706
Total		41906	100.000	4329287	100.000

Racemic **34**

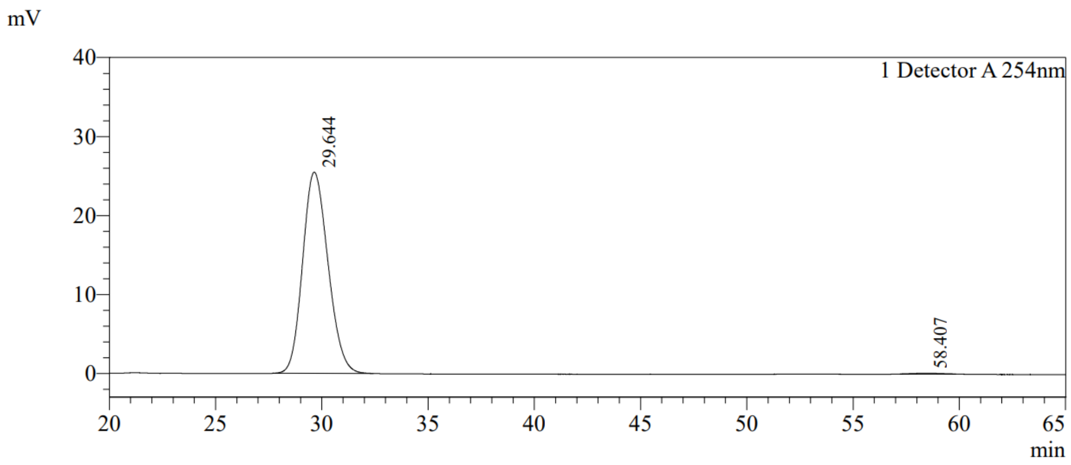

Peak Table

Detector A 254nm

Peak#	Ret. Time	Height	Height%	Area	Area%
1	29.644	25484	99.616	2123367	99.575
2	58.407	98	0.384	9058	0.425
Total		25582	100.000	2132425	100.000

Enantiomerically enriched **34**

4-((*S*, *E*)-2-(2-(2,4-dinitrophenoxy)naphthalen-1-yl)-3-oxoprop-1-en-1-yl)phenyl (4*R*)-4-((3*R*,5*R*,8*R*,10*S*,13*R*,14*S*,17*R*)-3-hydroxy-10,13-dimethylhexadecahydro-1H-cyclopenta[a]phenanthren-17-yl)pentanoate (35)

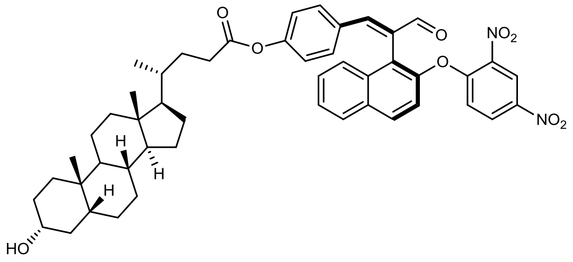

35

A yellow solid, 95% yield, 77.3 mg; m.p: 113-115 ^{o}C; **[α]$^{25}_{D}$** = -87.7 (c = 0.25 in $CHCl_3$); **^{1}H NMR (400 MHz, $CDCl_3$)** δ 9.78 (s, 1H), 8.70 (d, *J* = 2.7 Hz, 1H), 8.13 (dd, *J* = 9.3, 2.8 Hz, 1H), 8.04 (d, *J* = 8.9 Hz, 1H), 7.99 – 7.94 (m, 1H), 7.76 (s, 1H), 7.63 (dt, *J* = 7.5, 1.1 Hz, 1H), 7.57 (ddd, *J* = 8.1, 6.9, 1.4 Hz, 1H), 7.51 (m, *J* = 8.3, 6.9, 1.5 Hz, 1H), 7.28 (s, 1H), 7.09 – 7.02 (m, 2H), 6.93 – 6.83 (m, 3H), 3.62 (dt, *J* = 11.1, 6.1 Hz, 1H), 2.54 (m, *J* = 15.1, 9.7, 5.0 Hz, 1H), 2.41 (ddd, *J* = 15.8, 9.1, 6.7 Hz, 1H), 2.01 – 1.92 (m, 1H), 1.90 – 1.78 (m, 4H), 1.79 – 1.62 (m, 3H), 1.59 – 1.46 (m, 4H), 1.46 – 1.38 (m, 6H), 1.31 – 1.16 (m, 6H), 1.15 – 0.98 (m, 6H), 0.94 (d, *J* = 6.3 Hz, 3H), 0.91 (s, 3H), 0.64 (s, 3H); **^{13}C NMR (100 MHz, $CDCl_3$)** δ 192.90, 172.29, 155.29, 152.80, 152.64, 148.21, 141.50, 139.30, 134.89, 132.77, 131.94, 131.68, 131.57, 128.92, 128.70, 128.35, 126.92, 125.07, 123.21, 122.16, 121.61, 119.82,

118.86, 56.64, 56.07, 42.91, 42.23, 40.58, 40.32, 36.59, 36.00, 35.47, 34.72, 31.46, 30.98, 30.68, 28.35, 27.33, 26.55, 24.33, 23.51, 20.97, 18.42, 12.20; **HRMS (ESI, m/z)** calcd for $C_{49}H_{54}N_2O_9$ $[M+Na]^+$ = 837.3727, found = 837.3727; **HPLC analysis**: 94% ee (ADH column, 25 ^oC, n-hexane/*i*-PrOH = 70/30, 1.0 mL/min, λ = 254 nm), Rt (major) = 68.8 min, Rt (minor) = 55.8 min.

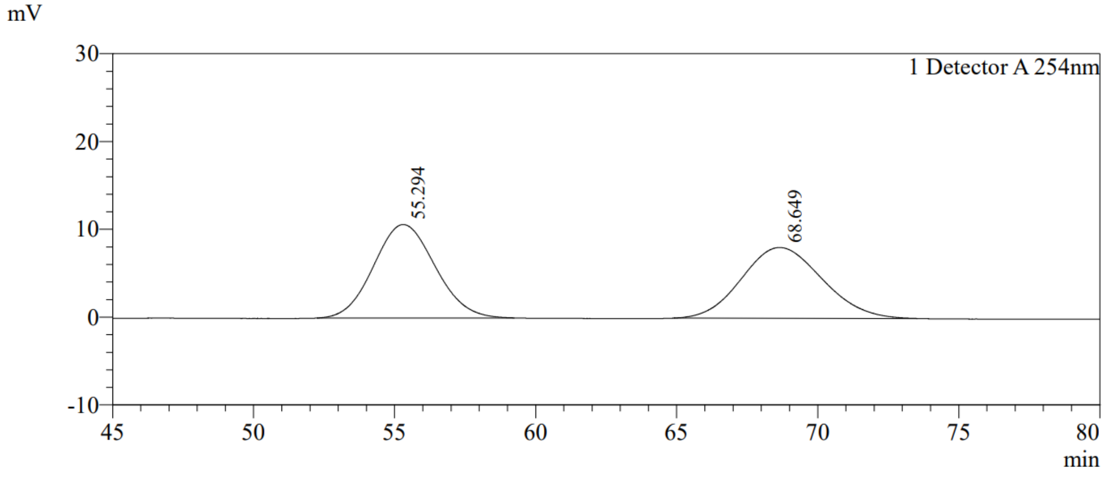

Peak Table

Detector A 254nm

Peak#	Ret. Time	Height	Height%	Area	Area%
1	55.294	10648	56.902	1614818	50.233
2	68.649	8065	43.098	1599867	49.767
Total		18713	100.000	3214685	100.000

Racemic **35**

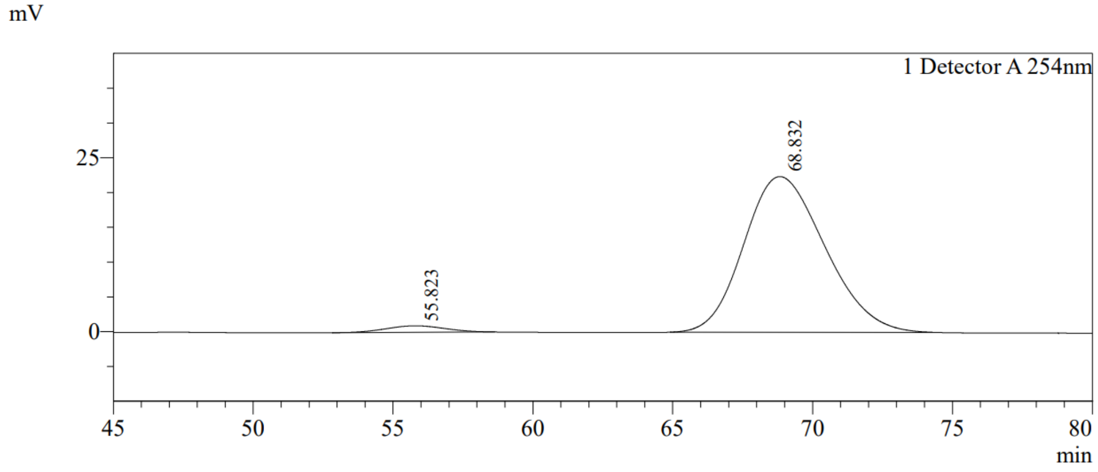

Peak Table

Detector A 254nm

Peak#	Ret. Time	Height	Height%	Area	Area%
1	55.823	932	3.994	136922	2.933
2	68.832	22399	96.006	4530755	97.067
Total		23331	100.000	4667676	100.000

Enantiomerically enriched **35**

(*S*, *E*)-4-(2-(2-(2,4-dinitrophenoxy)naphthalen-1-yl)-3-oxoprop-1-en-1-yl)phenyl 5-(2,5-dimethylphenoxy)-2,2-dimethylpentanoate (36)

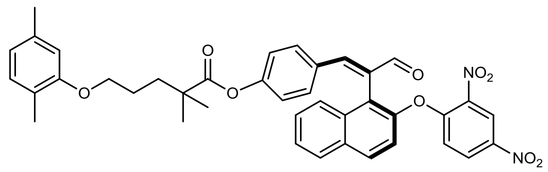

36

A yellow solid, 94% yield, 64.6 mg; m.p: 70-71 ^{o}C; $[\alpha]^{25}_{D}$ = -126.1 (c = 0.25 in $CHCl_3$); **^{1}H NMR (400 MHz, $CDCl_3$)** δ 9.79 (s, 1H), 8.70 (d, *J* = 2.7 Hz, 1H), 8.12 (dd, *J* = 9.3, 2.8 Hz, 1H), 8.05 (d, *J* = 8.9 Hz, 1H), 7.98 (d, *J* = 7.8 Hz, 1H), 7.76 (s, 1H), 7.65 (d, *J* = 8.2 Hz, 1H), 7.61 – 7.50 (m, 2H), 7.28 (d, *J* = 8.9 Hz, 1H), 7.03 (d, *J* = 8.7 Hz, 2H), 6.96 (d, *J* = 7.3 Hz, 1H), 6.88 (d, *J* = 9.3 Hz, 1H), 6.82 – 6.74 (m, 2H), 6.60 (d, *J* = 8.8 Hz, 2H), 3.95 (t, *J* = 5.4 Hz, 2H), 2.26 (s, 3H), 2.12 (s, 3H), 1.87 – 1.75 (m, 4H), 1.31 (s, 6H); **^{13}C NMR (100 MHz, $CDCl_3$)** δ 192.86, 175.97, 156.86, 155.29, 153.01, 152.58, 148.17, 141.48, 139.27, 136.59, 134.96, 132.83, 131.94, 131.66, 131.51, 131.21, 130.48, 128.94, 128.71, 128.37, 126.94, 125.06, 123.74, 123.26, 122.19, 121.58, 120.90, 119.85, 118.79, 112.10, 67.74, 42.66, 37.17, 25.30, 25.20, 25.16, 21.49, 15.88; **HRMS (ESI, m/z)** calcd for $C_{40}H_{36}N_2O_9$ $[M+Na]^+$ = 711.2319, found = 711.2320; **HPLC analysis**: 96% ee (ADH column, 25 ^{o}C, n-hexane/*i*-PrOH = 70/30, 1.0 mL/min, λ = 254 nm), Rt (major) = 9.8 min, Rt (minor) = 8.8 min.

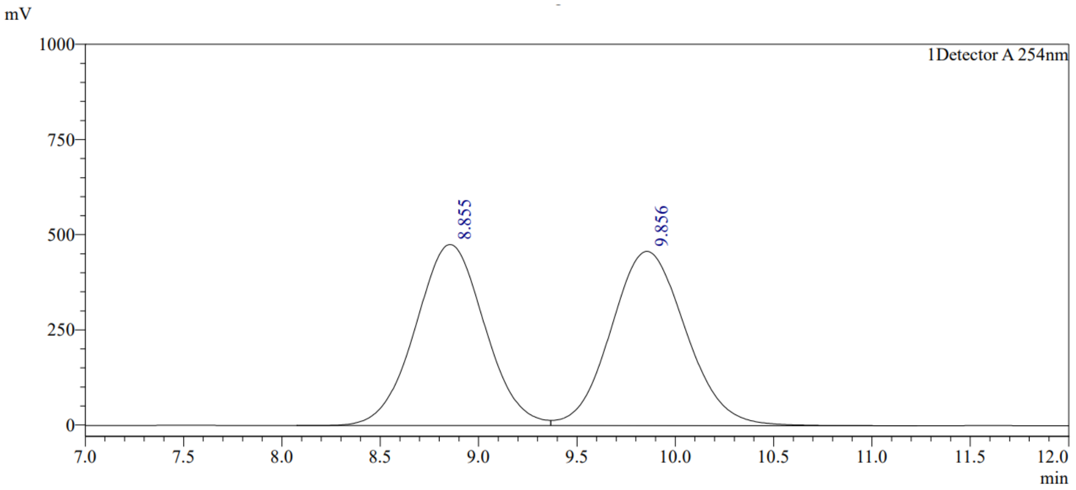

Peak Table

Detector A 254nm

Peak#	Ret. Time	Height	Height%	Area	Area%
1	8.855	475812	50.973	11695263	49.097
2	9.856	457640	49.027	12125315	50.903
Total		933452	100.000	23820578	100.000

Racemic **36**

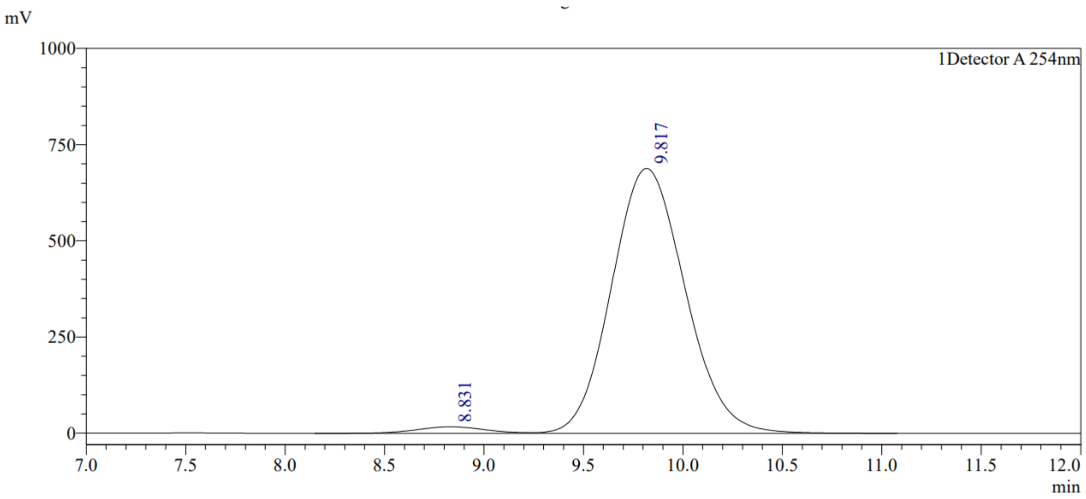

Peak Table

Detector A 254nm

Peak#	Ret. Time	Height	Height%	Area	Area%
1	8.831	17027	2.414	404027	2.232
2	9.817	688213	97.586	17697756	97.768
Total		705241	100.000	18101783	100.000

Enantiomerically enriched **36**

(*S*, *E*)-4-(2-(2-(2,4-dinitrophenoxy)naphthalen-1-yl)-3-oxoprop-1-en-1-yl)phenyl 4-([1,1'-biphenyl]-4-yl)-4-oxobutanoate (37)

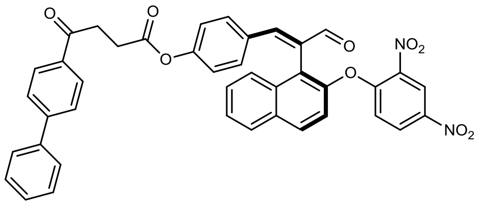

37

A yellow solid, 93% yield, 60.7 mg; m.p: 98-100 ^{o}C; $[\alpha]^{25}_{D}$ = -123.0 (c = 0.25 in $CHCl_3$); **^{1}H NMR (400 MHz, $CDCl_3$)** δ 9.78 (s, 1H), 8.70 (d, *J* = 2.7 Hz, 1H), 8.12 (dd, *J* = 9.3, 2.8 Hz, 1H), 8.07 – 8.01 (m, 3H), 7.97 (d, *J* = 7.8 Hz, 1H), 7.76 (s, 1H), 7.71 – 7.67 (m, 2H), 7.65 – 7.60 (m, 3H), 7.57 (ddd, *J* = 8.2, 6.9, 1.3 Hz, 1H), 7.54 – 7.50 (m, 1H), 7.50 – 7.44 (m, 2H), 7.43 – 7.37 (m, 1H), 7.28 (s, 1H), 7.08 – 7.03 (m, 2H), 6.93 – 6.89 (m, 2H), 6.88 (d, *J* = 9.3 Hz, 1H), 3.41 (td, *J* = 6.2, 2.4 Hz, 2H), 2.96 (t, *J* = 6.6 Hz, 2H); **^{13}C NMR (100 MHz, $CDCl_3$)** δ 197.43, 192.89, 171.18, 155.28, 152.75, 152.59, 148.18, 146.25, 141.49, 139.92, 139.29, 135.16, 134.99, 132.80, 131.94, 131.69, 131.57, 131.30, 129.11, 128.94, 128.78, 128.72, 128.45, 128.37, 127.45, 127.41, 126.94, 125.06, 123.19, 122.19, 121.60, 119.81, 118.81, 33.55, 28.63; **HRMS (ESI, m/z)** calcd for $C_{41}H_{28}N_2O_9$ $[M+Na]^+$ = 715.1693, found = 715.1695; HPLC analysis: 95% ee (IE column, 25 ^{o}C, n-hexane/*i*-PrOH =60/40, 1.0 mL/min, λ = 254 nm), Rt (major) = 106.8 min, Rt (minor) = 78.7 min.

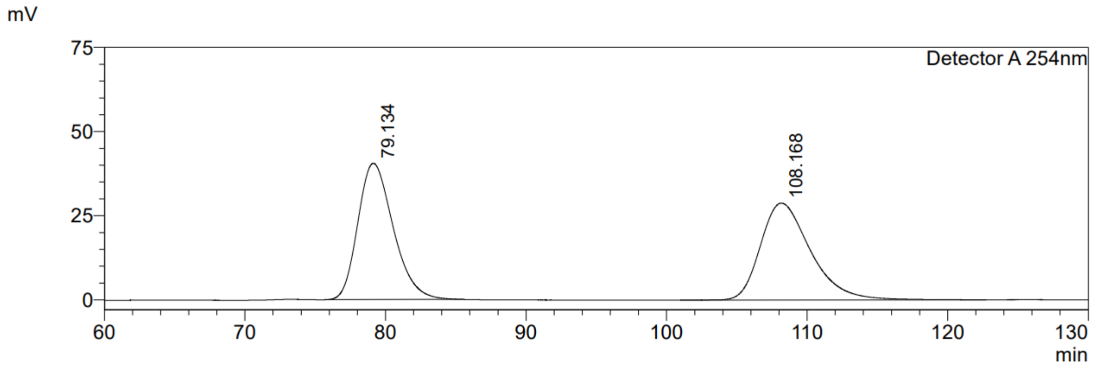

Detector A 254nm

Peak#	Ret. Time	Height	Height%	Area	Area%
1	79.134	40509	58.477	7095760	49.963
2	108.168	28764	41.523	7106300	50.037
Total		69274	100.000	14202060	100.000

Racemic **37**

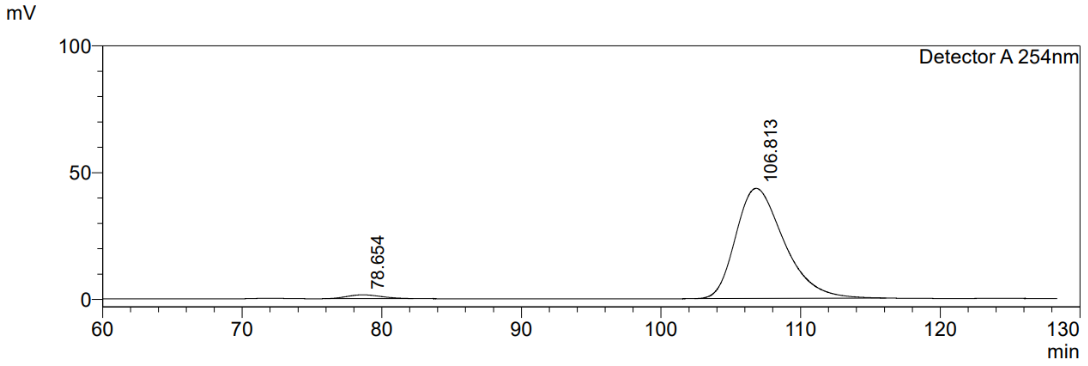

Detector A 254nm

Peak#	Ret. Time	Height	Height%	Area	Area%
1	78.654	1506	3.353	254688	2.380
2	106.813	43423	96.647	10447427	97.620
Total		44930	100.000	10702116	100.000

Enantiomerically enriched **37**

(*S*, *E*)-4-(2-(2-(2,4-dinitrophenoxy)naphthalen-1-yl)-3-oxoprop-1-en-1-yl)phenyl 2-(3-cyano-4-isobutoxyphenyl)thiazole-5-carboxylate (38)

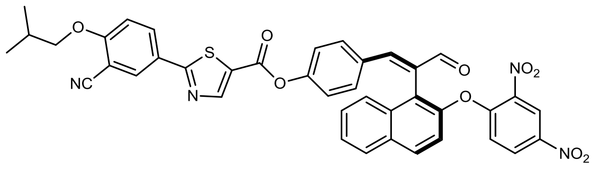

38

A yellow solid, 89% yield, 65.8 mg; m.p: 115-117 °C; $[\alpha]^{25}_{D}$ = -123.9 (c = 0.25 in $CHCl_3$); **^{1}H NMR (400 MHz, $CDCl_3$)** δ 9.80 (s, 1H), 8.72 (d, *J* = 2.7 Hz, 1H), 8.23 – 8.14 (m, 2H), 8.10 (dd, *J* = 8.9, 2.3 Hz, 1H), 8.05 (d, *J* = 8.9 Hz, 1H), 7.98 (d, *J* = 7.8 Hz, 1H), 7.80 (s, 1H), 7.65 (d, *J* = 8.2 Hz, 1H), 7.61 – 7.50 (m, 2H), 7.28 (d, *J* = 8.9 Hz, 1H), 7.12 (d, *J* = 8.8 Hz, 2H), 7.05 – 6.98 (m, 3H), 6.94 (d, *J* = 9.3 Hz, 1H), 3.90

(d, J = 6.5 Hz, 2H), 2.77 (s, 3H), 2.20 (hept, J = 6.7 Hz, 1H), 1.09 (d, J = 6.7 Hz, 6H); **^{13}C NMR (100 MHz, $CDCl_3$)** δ 192.86, 168.63, 163.70, 162.89, 159.88, 155.28, 152.41, 152.11, 148.22, 141.54, 139.30, 135.19, 132.82, 132.73, 132.39, 131.95, 131.74, 131.70, 131.65, 128.95, 128.76, 128.38, 126.96, 125.85, 125.03, 123.10, 122.19, 121.64, 120.12, 119.78, 118.85, 115.40, 112.85, 103.27, 28.29, 19.16, 17.84; HRMS (ESI, m/z) calcd for $C_{41}H_{30}N_4O_9S$ $[M+Na]^+$ = 777.1631, found = 777.1634; **HPLC analysis**: 96% ee (ADH column, 25 ^{o}C, n-hexane/*i*-PrOH = 70/30, 1.0 mL / min, λ = 254 nm), Rt (major) = 80.0 min, Rt (minor) = 111.5 min.

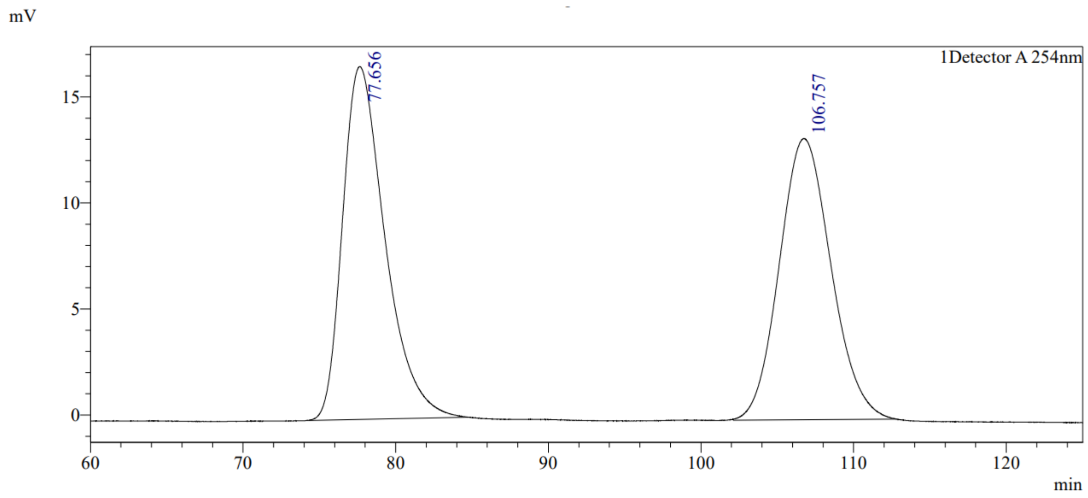

Peak Table

Detector A 254nm

Peak#	Ret. Time	Height	Height%	Area	Area%
1	77.656	16644	55.655	3217983	50.725
2	106.757	13261	44.345	3126033	49.275
Total		29905	100.000	6344015	100.000

Racemic **38**

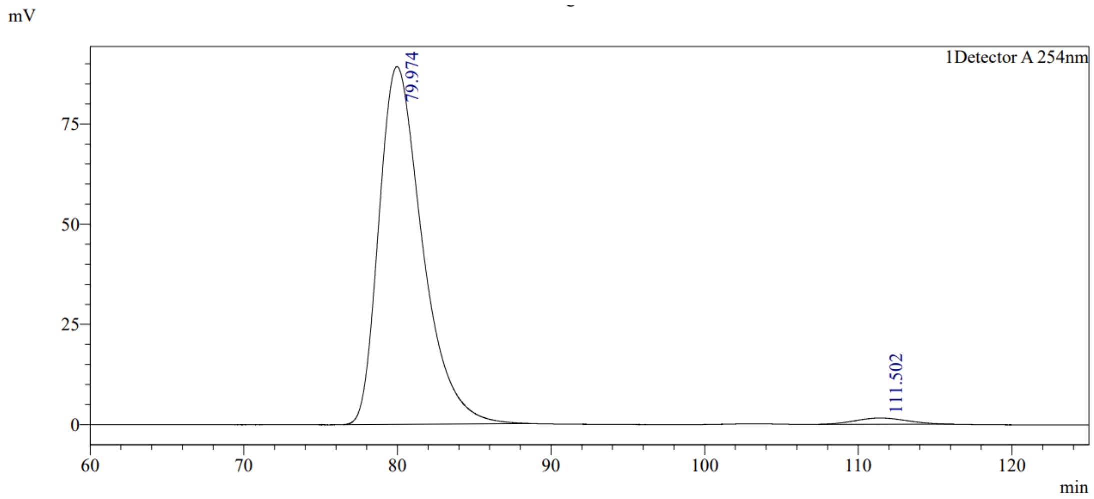

Peak Table

Detector A 254nm

Peak#	Ret. Time	Height	Height%	Area	Area%
1	79.974	89273	98.291	17543634	97.959
2	111.502	1552	1.709	365459	2.041
Total		90825	100.000	17909093	100.000

Enantiomerically enriched **38**

(*S*, *E*)-4-(2-(2-(2,4-dinitrophenoxy)naphthalen-1-yl)-3-oxoprop-1-en-1-yl)phenyl 3-(4,5-diphenyloxazol-2-yl)propanoate (39)

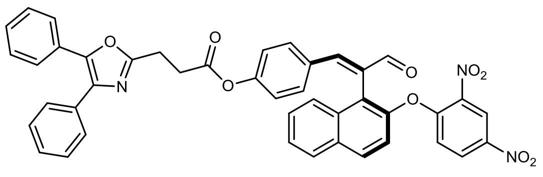

39

A yellow solid, 97% yield, 70.9 mg; m.p: 85-87 ^{o}C; $[\alpha]^{25}_{D}$ = -106.3 (c = 0.25 in $CHCl_3$); **^{1}H NMR (400 MHz, $CDCl_3$)** δ 9.78 (s, 1H), 8.70 (d, *J* = 2.7 Hz, 1H), 8.12 (dd, *J* = 9.3, 2.8 Hz, 1H), 8.04 (d, *J* = 8.9 Hz, 1H), 7.97 (d, *J* = 7.9 Hz, 1H), 7.76 (s, 1H), 7.65 – 7.57 (m, 4H), 7.56 – 7.49 (m, 3H), 7.32 (qd, *J* = 4.5, 1.6 Hz, 6H), 7.28 (s, 1H), 7.07 – 7.01 (m, 2H), 6.92 – 6.87 (m, 3H), 3.28 – 3.22 (m, 2H), 3.14 – 3.08 (m, 2H); **^{13}C NMR (100 MHz, $CDCl_3$)** δ 192.89, 170.24, 161.35, 155.26, 152.59, 152.54, 148.19, 145.77, 141.50, 139.28, 135.27, 134.99, 132.75, 132.47, 131.93, 131.69, 131.57, 131.39, 129.00, 128.93, 128.77, 128.70, 128.68, 128.66, 128.35, 128.22, 127.97, 126.94, 126.67, 125.05, 123.15, 122.13, 121.60, 119.81, 118.82, 31.34, 23.54; **HRMS (ESI, m/z)** calcd for $C_{43}H_{29}N_3O_9$ $[M+Na]^+$ = 754.1801, found = 754.1802; **HPLC analysis**: 94% ee (IE column, 25 ^{o}C, n-hexane/*i*-PrOH = 70/30, 1.0 mL/min, λ = 254 nm), Rt (major) = 77.5 min, Rt (minor) = 49.5 min.

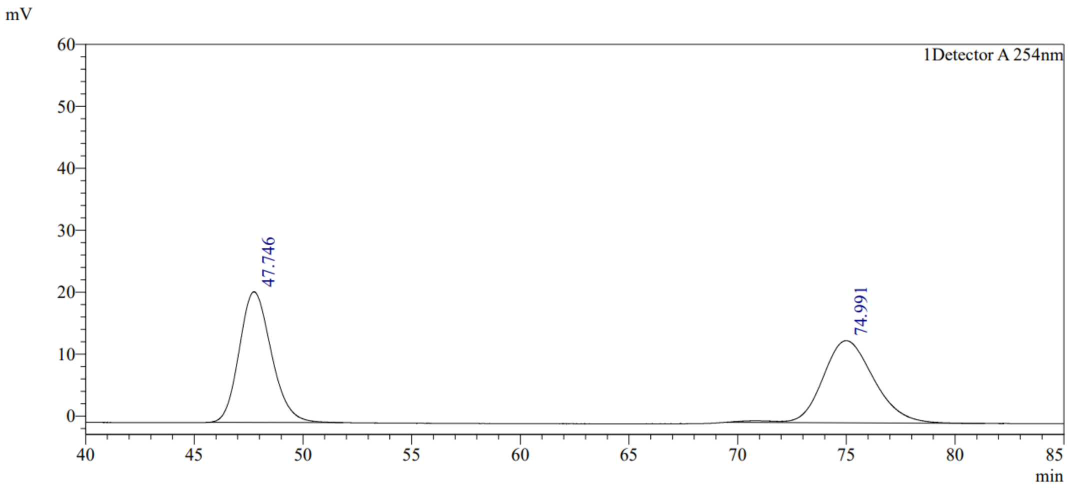

Peak Table

Detector A 254nm

Peak#	Ret. Time	Height	Height%	Area	Area%
1	47.746	21110	61.404	2171405	50.064
2	74.991	13269	38.596	2165822	49.936
Total		34378	100.000	4337227	100.000

Racemic **39**

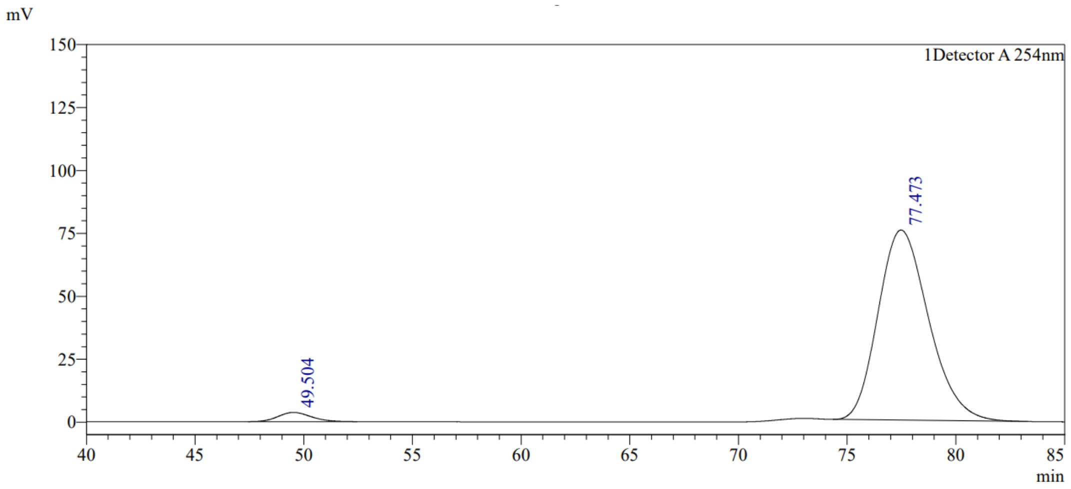

Peak Table

Detector A 254nm

Peak#	Ret. Time	Height	Height%	Area	Area%
1	49.504	3651	4.610	384987	3.053
2	77.473	75540	95.390	12225769	96.947
Total		79192	100.000	12610756	100.000

Enantiomerically enriched **39**

4-((*S*, *E*)-2-(2-(2,4-dinitrophenoxy)naphthalen-1-yl)-3-oxoprop-1-en-1-yl)phenyl (9*Z*,12*Z*)-octadeca-9,12-dienoate (40)

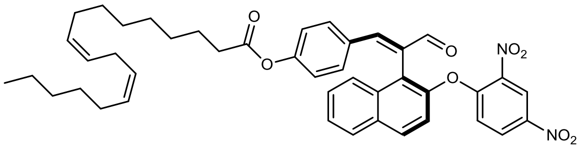

40

A yellow oil, 96% yield, 68.9 mg; **[α]^{25}$_D$** = -116.3 (c = 0.25 in $CHCl_3$); **^{1}H NMR (400 MHz, $CDCl_3$)** δ 9.78 (s, 1H), 8.70 (d, *J* = 2.7 Hz, 1H), 8.13 (dd, *J* = 9.3, 2.8 Hz, 1H), 8.04 (d, *J* = 8.9 Hz, 1H), 7.97 (d, *J* = 7.9 Hz, 1H), 7.76 (s, 1H), 7.63 (d, *J* = 8.3 Hz, 1H), 7.60 – 7.48 (m, 2H), 7.29 (s, 1H), 7.05 (d, *J* = 8.7 Hz, 2H), 6.92 – 6.83 (m, 3H), 5.43 – 5.28 (m, 4H), 2.76 (t, *J* = 6.3 Hz, 2H), 2.49 (t, *J* = 7.5 Hz, 2H), 2.04 (dd, *J* = 8.5, 5.0 Hz, 4H), 1.69 (p, *J* = 7.4 Hz, 2H), 1.54 (d, *J* = 12.4 Hz, 1H), 1.36 – 1.27 (m, 13H), 0.88 (t, *J* = 6.9 Hz, 3H); **^{13}C NMR (100 MHz, $CDCl_3$)** δ 192.89, 171.80, 155.30, 152.77, 152.62, 148.22, 141.51, 139.30, 134.92, 132.78, 131.95, 131.68, 131.57, 131.20, 130.37, 130.13, 128.93, 128.70, 128.35, 128.24, 128.04, 126.93, 125.07, 123.22, 122.18, 121.61, 119.82, 118.86, 34.45, 31.67, 29.71, 29.48, 29.26, 29.20, 29.14, 27.35, 27.31, 25.78, 24.93, 22.71, 14.21; **HRMS (ESI, m/z)** calcd for $C_{43}H_{46}N_2O_8$ $[M+Na]^+$ = 741.3152, found = 741.3155; HPLC analysis: 95% ee (IE

column, 25 °C, n-hexane/*i*-PrOH = 70/30, 1.0 mL/min, λ = 254 nm), Rt (major) = 16.4 min, Rt (minor) = 12.2 min.

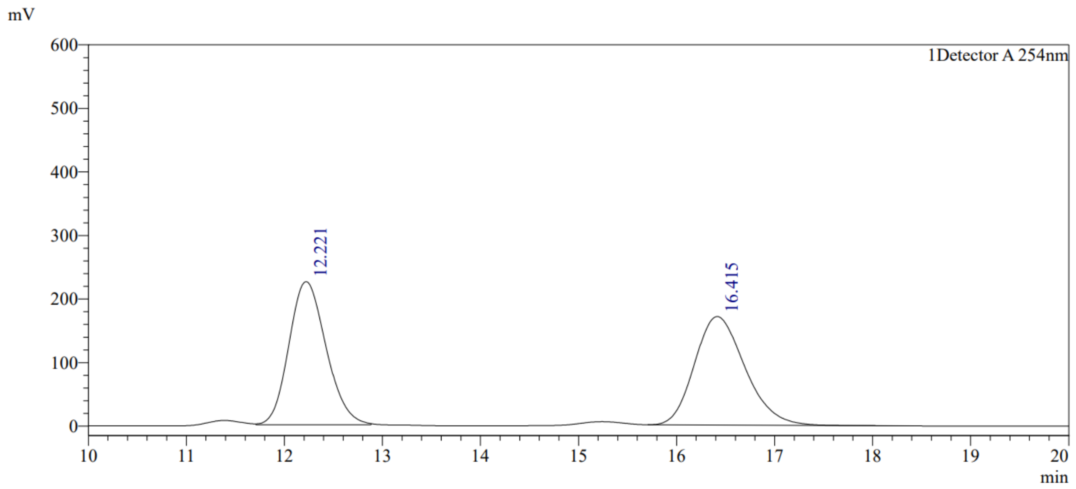

Peak Table

Detector A 254nm

Peak#	Ret. Time	Height	Height%	Area	Area%
1	12.221	225246	56.864	5945345	49.235
2	16.415	170866	43.136	6130144	50.765
Total		396112	100.000	12075488	100.000

Racemic **40**

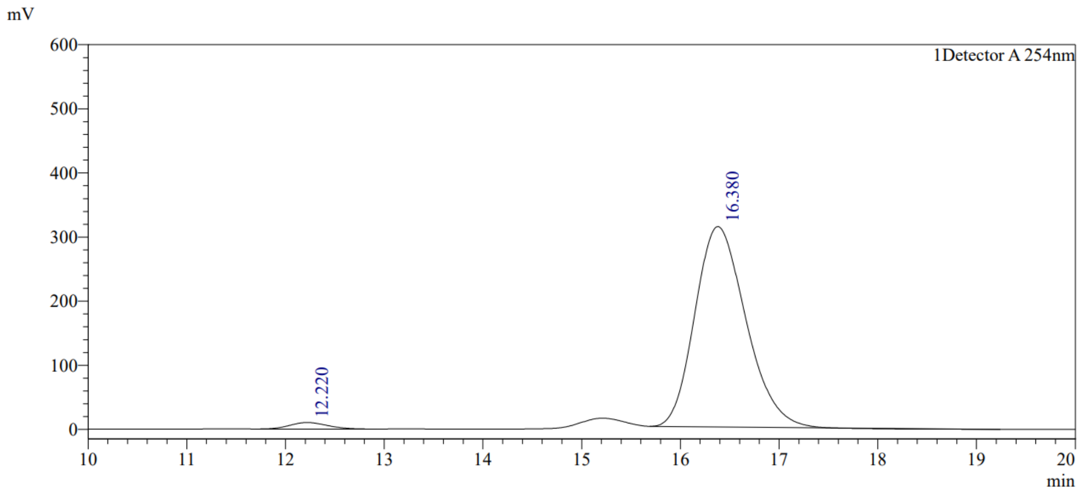

Peak Table

Detector A 254nm

Peak#	Ret. Time	Height	Height%	Area	Area%
1	12.220	10079	3.126	266748	2.329
2	16.380	312382	96.874	11185582	97.671
Total		322461	100.000	11452330	100.000

Enantiomerically enriched **40**

(*S, E*)-4-(2-(2-(2,4-dinitrophenoxy)naphthalen-1-yl)-3-oxoprop-1-en-1-yl)phenyl 2-(1-(4-chlorobenzoyl)-5-methoxy-2-methyl-1H-indol-3-yl)acetate (41)

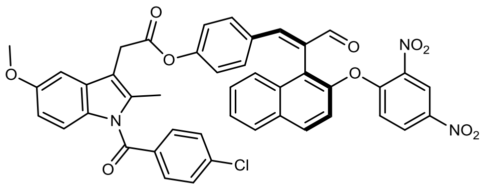

41

A yellow solid, 95% yield, 75.6 mg; m.p: 109-111 °C; **[α]$^{25}_{D}$** = -89.1 (c = 0.25 in $CHCl_3$); **^{1}H NMR (400 MHz, $CDCl_3$)** δ 9.77 (s, 1H), 8.69 (d, *J* = 2.7 Hz, 1H), 8.11 (dd, *J* = 9.3, 2.8 Hz, 1H), 8.03 (d, *J* = 8.9 Hz, 1H), 7.96 (d, *J* = 7.9 Hz, 1H), 7.75 (s, 1H), 7.67 – 7.63 (m, 2H), 7.61 (d, *J* = 8.3 Hz, 1H), 7.58 – 7.53 (m, 1H), 7.51 (dd, *J* = 8.2, 1.3 Hz, 1H), 7.48 – 7.44 (m, 2H), 7.27 (s, 1H), 7.03 (d, *J* = 8.7 Hz, 2H), 6.98 (d, *J* = 2.4 Hz, 1H), 6.89 (d, *J* = 9.4 Hz, 1H), 6.87 – 6.82 (m, 3H), 6.67 (dd, *J* = 9.0, 2.5 Hz, 1H), 3.84 (s, 2H), 3.80 (s, 3H), 2.41 (s, 3H); **^{13}C NMR (100 MHz, $CDCl_3$)** δ 192.85, 168.82, 168.40, 156.27, 155.27, 152.59, 152.43, 148.18, 141.50, 139.54, 139.26, 136.45, 135.08, 133.89, 132.73, 131.93, 131.72, 131.54, 131.46, 131.34, 130.97, 130.48, 129.29, 128.95, 128.72, 128.36, 126.94, 125.00, 123.13, 122.01, 121.60, 119.80, 118.84, 115.16, 111.93, 111.66, 101.28, 55.88, 30.62, 13.47; **HRMS (ESI, m/z)** calcd for $C_{44}H_{30}ClN_3O_{10}$ $[M+Na]^+$ = 818.1517, found = 818.1520; **HPLC analysis**: 96% ee (ADH column, 25 °C, n-hexane/*i*-PrOH = 70/30, 1.0 mL/min, λ = 254 nm), Rt (major) = 107.8 min, Rt (minor) = 122.4 min.

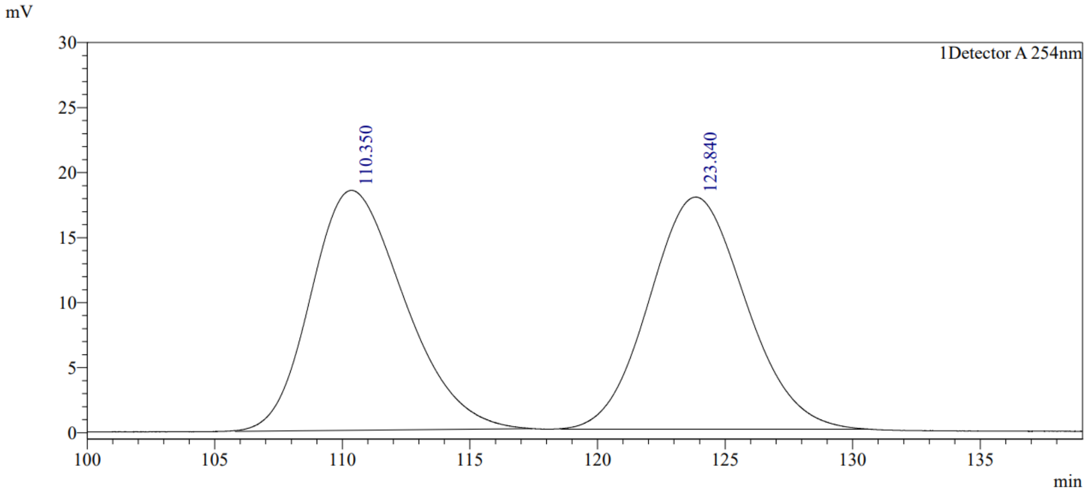

Peak Table

Detector A 254nm

Peak#	Ret. Time	Height	Height%	Area	Area%
1	110.350	18451	50.815	4710270	50.008
2	123.840	17859	49.185	4708816	49.992
Total		36310	100.000	9419086	100.000

Racemic **41**

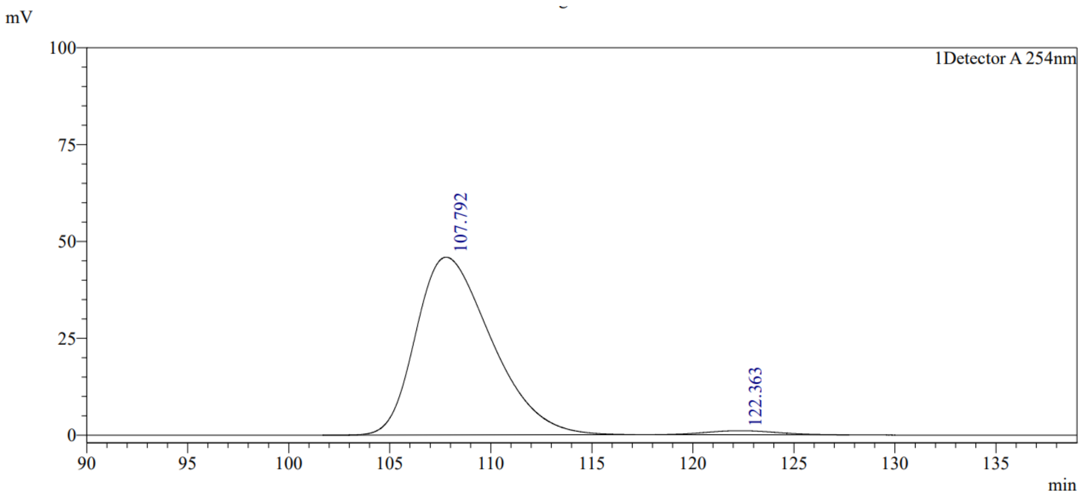

Peak Table

Detector A 254nm

Peak#	Ret. Time	Height	Height%	Area	Area%
1	107.792	45908	97.764	11941551	97.882
2	122.363	1050	2.236	258420	2.118
Total		46958	100.000	12199972	100.000

Enantiomerically enriched **41**

(*S*, *E*)-4-(2-(2-(2,4-dinitrophenoxy)naphthalen-1-yl)-3-oxoprop-1-en-1-yl)phenyl 3-(4-(4-chlorobenzoyl)phenyl)-2,2-dimethylpropanoate (42)

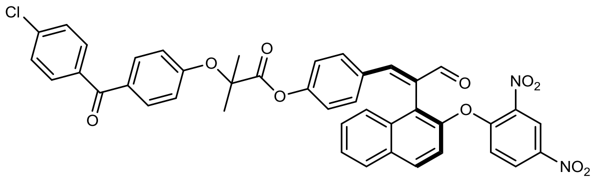

42

A yellow solid, 96% yield, 72.4 mg; m.p: 84-86 ^{o}C; **[α]^{25}$_{D}$** = -87.1 (c = 0.25 in CHCl$_3$); **^{1}H NMR (400 MHz, CDCl$_3$)** δ 9.77 (s, 1H), 8.70 (d, *J* = 2.7 Hz, 1H), 8.13 (dd, *J* = 9.3, 2.8 Hz, 1H), 8.04 (d, *J* = 8.9 Hz, 1H), 7.97 (d, *J* = 8.0 Hz, 1H), 7.76 – 7.72 (m, 3H), 7.71 – 7.67 (m, 2H), 7.62 (d, *J* = 8.3 Hz, 1H), 7.59 – 7.54 (m, 1H), 7.54 – 7.48 (m, 1H), 7.46 – 7.42 (m, 2H), 7.28 (s, 1H), 7.05 (d, *J* = 8.7 Hz, 2H), 6.94 – 6.88 (m, 3H), 6.81 – 6.76 (m, 2H), 1.77 (s, 6H); **^{13}C NMR (100 MHz, CDCl$_3$)** δ 194.28, 192.81, 172.14, 159.46, 155.27, 152.22, 148.17, 141.53, 139.27, 138.65, 136.38, 135.32, 132.73, 132.27, 131.95, 131.86, 131.81, 131.60, 131.32, 130.93, 129.00, 128.73, 128.41, 126.99, 124.97, 123.06, 121.84, 121.63, 119.84, 118.80, 117.39, 25.59, 25.43; **HRMS (ESI, m/z)** calcd for $C_{42}H_{29}ClN_2O_{10}$ [M+Na]$^+$ = 779.1409, found = 779.1408; **HPLC analysis**: 95% ee (IE column, 25 ^{o}C, n-hexane/*i*-PrOH = 70/30, 1.0 mL/min, λ = 254 nm), Rt (major) = 38.2 min, Rt (minor) = 41.5 min.

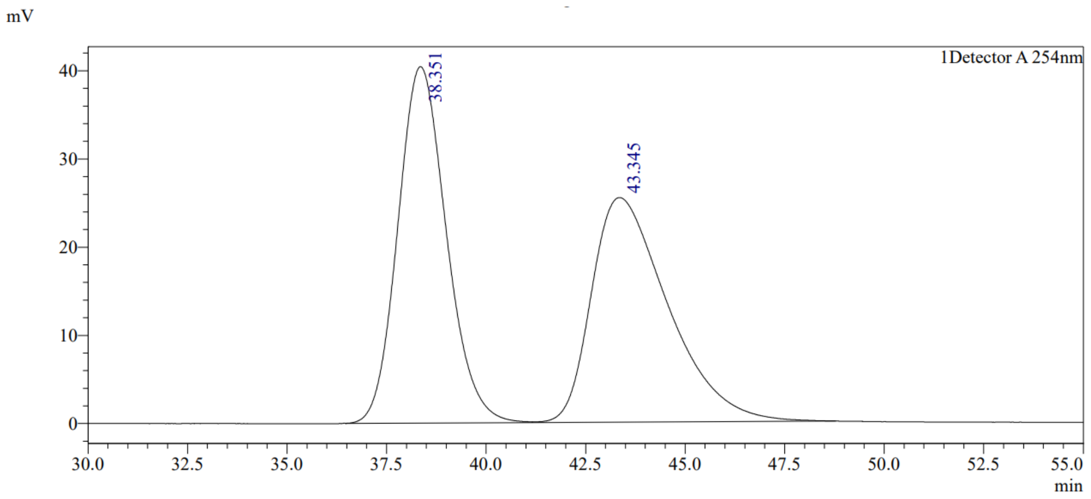

Peak Table

Detector A 254nm

Peak#	Ret. Time	Height	Height%	Area	Area%
1	38.351	40421	61.346	3431131	50.202
2	43.345	25470	38.654	3403571	49.798
Total		65891	100.000	6834702	100.000

Racemic **42**

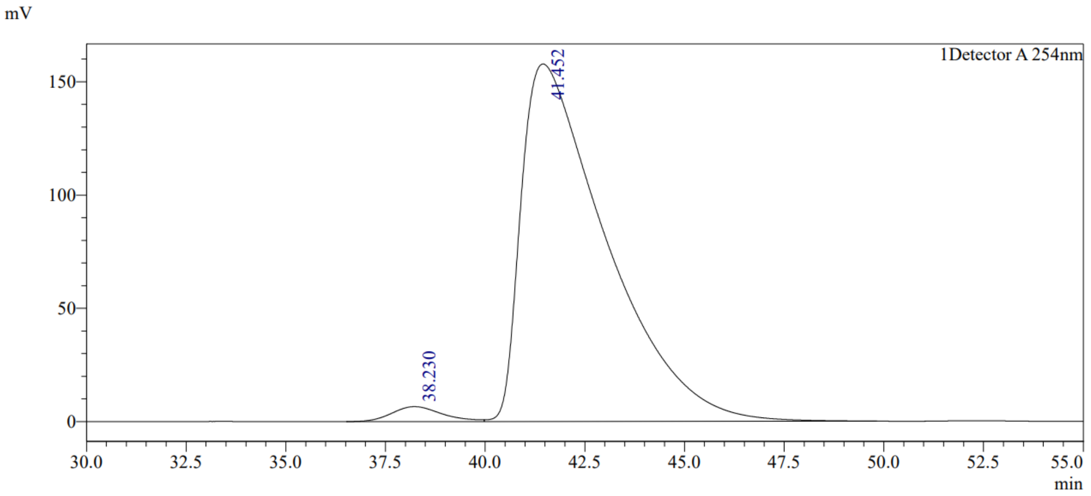

Peak Table

Detector A 254nm

Peak#	Ret. Time	Height	Height%	Area	Area%
1	38.230	6602	4.015	578142	2.401
2	41.452	157826	95.985	23497315	97.599
Total		164428	100.000	24075456	100.000

Enantiomerically enriched **42**

(*S*, *E*)-4-(2-(2-(2,4-dinitrophenoxy)naphthalen-1-yl)-3-oxoprop-1-en-1-yl)phenyl 2-(11-oxo-6,11-dihydrodibenzo[b,e]oxepin-2-yl)acetate (43)

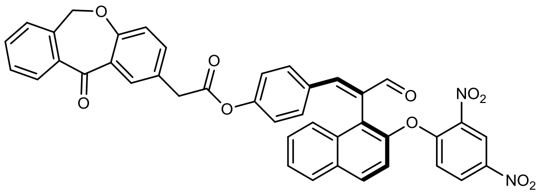

43

A yellow solid, 95% yield, 60.7 mg; m.p: 98-99 °C; **[α]^{25}$_D$** = -146.9 (c = 0.25 in $CHCl_3$); **^{1}H NMR (400 MHz, $CDCl_3$)** δ 9.77 (s, 1H), 8.69 (d, *J* = 2.7 Hz, 1H), 8.16 (d, *J* = 2.3 Hz, 1H), 8.12 (dd, *J* = 9.3, 2.8 Hz, 1H), 8.02 (d, *J* = 8.9 Hz, 1H), 7.96 (d, *J* = 7.9 Hz, 1H), 7.88 (dd, *J* = 7.7, 1.0 Hz, 1H), 7.75 (s, 1H), 7.62 (d, *J* = 8.3 Hz, 1H), 7.58 – 7.53 (m, 2H), 7.52 – 7.48 (m, 1H), 7.46 (ddd, *J* = 8.4, 4.1, 1.8 Hz, 2H), 7.36 (d, *J* = 6.9 Hz, 1H), 7.27 (s, 1H), 7.04 (dd, *J* = 8.6, 1.3 Hz, 3H), 6.92 – 6.84 (m, 3H), 5.18 (s, 2H), 3.82 (s, 2H); **^{13}C NMR (100 MHz, $CDCl_3$)** δ 192.86, 190.83, 169.41, 160.83, 155.27, 152.58, 152.47, 148.19, 141.50, 140.51, 139.29, 136.29, 135.65, 135.07, 132.99, 132.74, 132.68, 131.92, 131.70, 131.54, 131.44, 129.65, 129.43, 128.93, 128.70, 128.35, 127.98, 126.93, 125.42, 125.02, 123.13, 122.06, 121.60, 121.47, 119.78, 118.82, 40.37; **HRMS (ESI, m/z)** calcd for $C_{41}H_{26}N_2O_{10}$ $[M+Na]^+$ = 729.1485, found = 729.1485; **HPLC analysis**: 95% ee (ADH column, 25 °C, n-hexane/*i*-PrOH = 70/30, 1.0 mL/min, λ = 254 nm), Rt (major) = 96.4 min, Rt (minor) = 141.5 min.

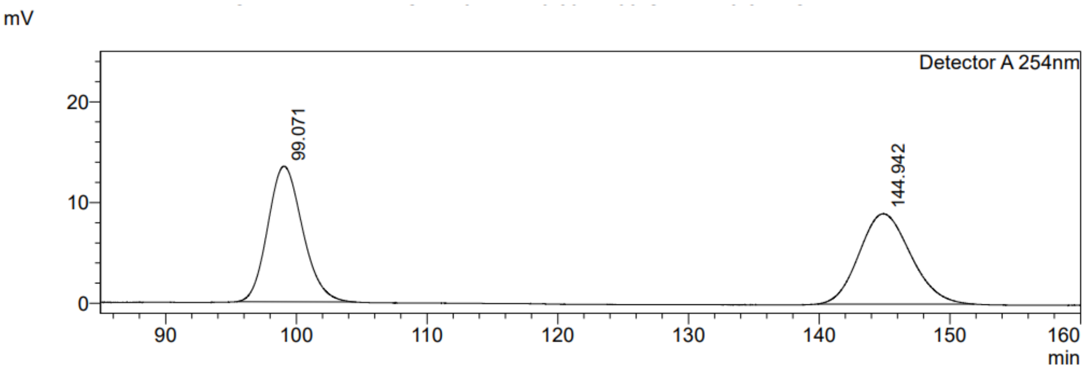

Detector A 254nm

Peak#	Ret. Time	Height	Height%	Area	Area%
1	99.071	13457	60.032	2495106	50.385
2	144.942	8959	39.968	2456946	49.615
Total		22416	100.000	4952052	100.000

Racemic **43**

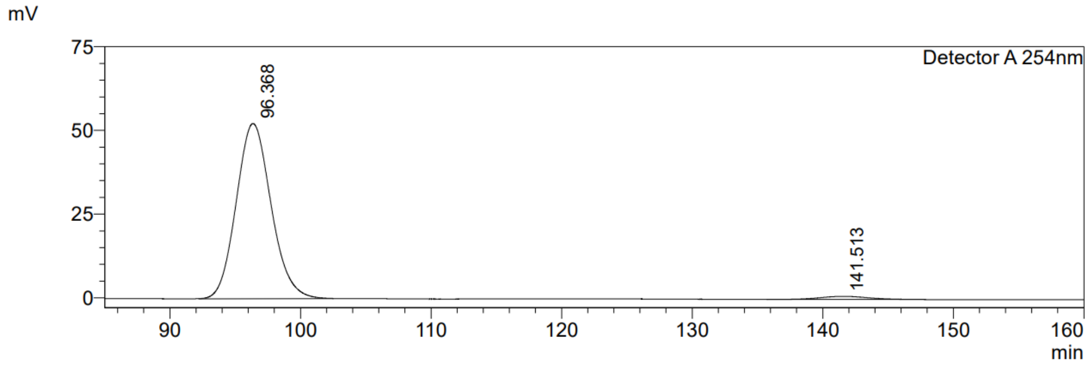

Detector A 254nm

Peak#	Ret. Time	Height	Height%	Area	Area%
1	96.368	52311	98.255	9711853	97.594
2	141.513	929	1.745	239398	2.406
Total		53240	100.000	9951251	100.000

Enantiomerically enriched **43**

6. Gram-scale preparations and transformations

6.1 Procedure for the gram scale synthesis of 17

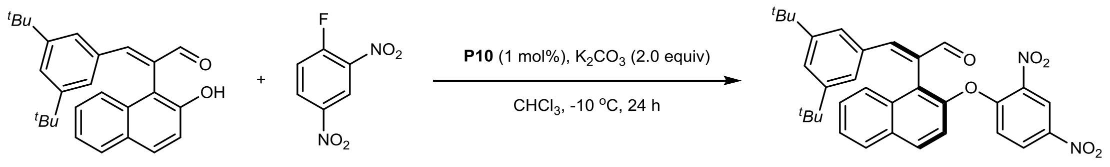

To a 100 mL round bottle flask with a magnetic stirring bar were added **1o** (1.16 g, 3.0 mmol) and **2a** (670 mg, 3.6 mmol), followed by the addition of K_2CO_3 (829.2 mg, 6.0 mmol) and catalyst **P10** (26.2 mg, 0.03 mol), followed by the addition of chloroform (30 mL). The reaction mixture was stirred at -10 ^{o}C for 24 h. Then, the reaction was quenched by 20 mL HCl (1 M) and extracted by DCM three times, and the combined organic phase was concentrated in vacuo. The crude residue was purified by column chromatography on silica gel (CH_2Cl_2/ petroleum ether = 3/1) to afford target product **17** (1.64 g, 99% yield, 96% ee).

6.2 Procedure for transformations of 17

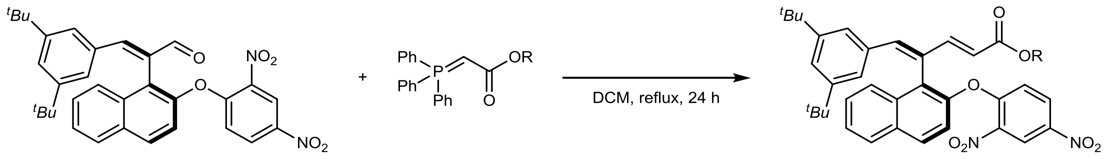

17 **S6** **44, 46, 47**

To a schlenk tube with a magnetic stirring bar were added **30** (55.3 mg, 0.10 mmol) and **S6** (0.20 mmol), followed by the addition of Dichloromethane (4.0 mL). The reaction mixture was heated to 40 °C for 24 h. Then, the solvent was removed under reduced pressure, and the residue was purified by column chromatography on silica gel (Petroleum ether/ethyl acetate = 10/1) to afford target product **44**, **46**, **47**.

ethyl (*S*, 2*E*,4*Z*)-5-(3,5-di-*tert*-butylphenyl)-4-(2-(2,4-dinitrophenoxy)naphthalen-1-yl)penta-2,4-dienoate (44)

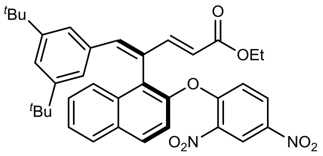

44

A yellow solid, 91% yield, 56.6 mg; m.p: 71-73 °C; **[α]$^{25}_{D}$** = +296.6 (c = 0.25 in $CHCl_3$); **^{1}H NMR (400 MHz, $CDCl_3$)** δ 8.67 (d, *J* = 2.7 Hz, 1H), 8.04 – 7.98 (m, 2H), 7.88 – 7.84 (m, 1H), 7.74 (dd, *J* = 15.5, 0.6 Hz, 1H), 7.63 – 7.54 (m, 3H), 7.27 (s, 1H), 7.22 (s, 1H), 7.17 (t, *J* = 1.8 Hz, 1H), 6.58 (d, *J* = 1.7 Hz, 2H), 6.24 (d, *J* = 9.3 Hz, 1H), 5.26 (d, *J* = 15.5 Hz, 1H), 4.19 – 4.12 (m, 2H), 1.25 (t, *J* = 7.1 Hz, 1H), 0.92 (s, 18H); **^{13}C NMR (100 MHz, $CDCl_3$)** δ 167.00, 155.54, 150.99, 147.48, 147.26, 142.55, 140.91, 139.19, 134.52, 133.74, 132.05, 131.28, 130.92, 128.78, 128.55, 127.45, 127.01, 126.65, 125.94, 123.97, 123.02, 121.62, 120.44, 120.26, 118.16, 60.58, 34.57, 31.08, 14.35; **HRMS (ESI, m/z)** calcd for $C_{37}H_{38}N_2O_7$ $[M+Na]^+$ = 645.2577, found = 645.2571; **HPLC analysis**: 94% ee (IE column, 25 °C, n-hexane/*i*-PrOH = 90/10, 1.0 mL/min, λ = 254 nm), Rt (major) = 13.6 min, Rt (minor) = 11.2 min.

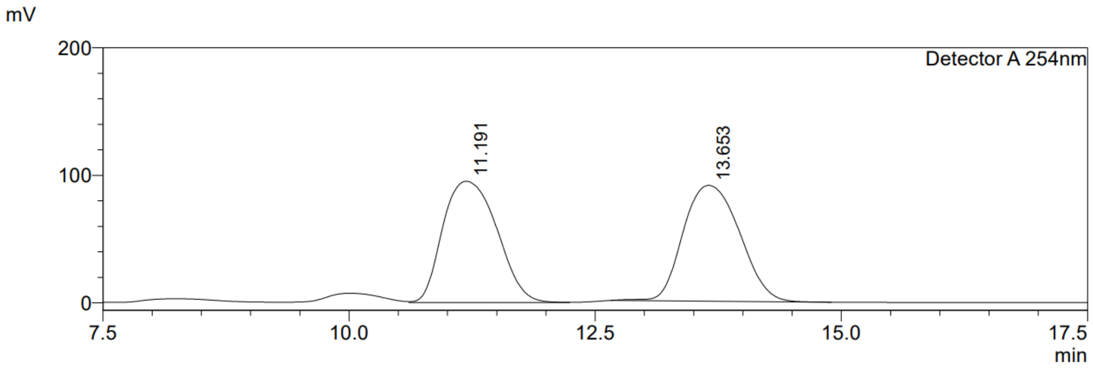

Detector A 254nm

Peak#	Ret. Time	Height	Height%	Area	Area%
1	11.191	95139	51.160	3651522	50.171
2	13.653	90824	48.840	3626620	49.829
Total		185963	100.000	7278142	100.000

Racemic **44**

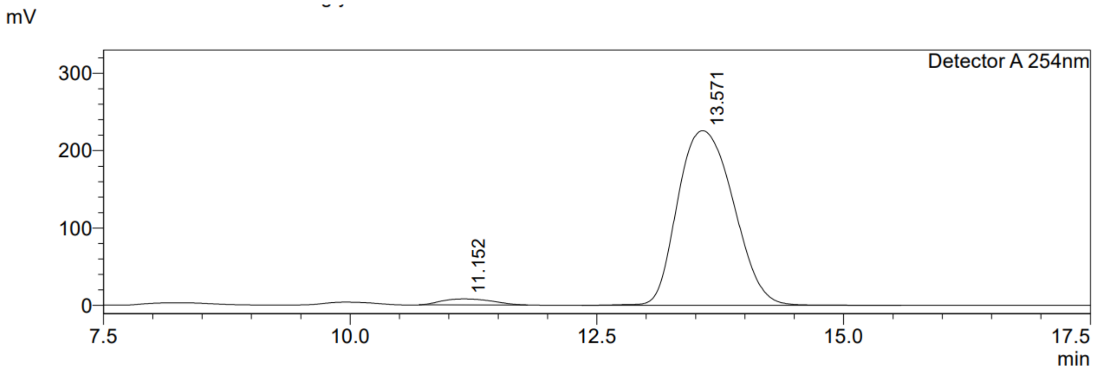

Detector A 254nm

Peak#	Ret. Time	Height	Height%	Area	Area%
1	11.152	7759	3.323	276416	3.016
2	13.571	225756	96.677	8889156	96.984
Total		233515	100.000	9165572	100.000

Enantiomerically enriched **44**

benzyl (*S*, 2*E*,4*Z*)-5-(3,5-di-*tert*-butylphenyl)-4-(2-(2,4-dinitrophenoxy)naphthalen-1-yl)penta-2,4-dienoate (46)

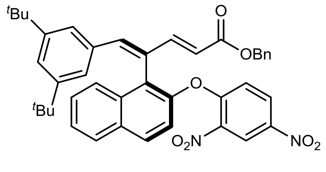

46

A yellow solid, 96% yield, 65.7 mg; m.p: 69-72 ^{o}C; **$[\alpha]^{25}_{D}$** = +183.4 (c = 0.25 in $CHCl_3$); **^{1}H NMR (400 MHz, $CDCl_3$)** δ 8.67 (d, *J* = 2.7 Hz, 1H), 8.02 – 7.97 (m, 2H), 7.86 – 7.82 (m, 1H), 7.78 (dd, J = 15.5, 0.6 Hz, 1H), 7.62 – 7.56 (m, 2H), 7.56 – 7.53 (m, 1H), 7.38 – 7.34 (m, 1H), 7.34 (s, 3H), 7.33 – 7.30 (m, 1H), 7.23 (d, *J* = 6.6 Hz,

2H), 7.17 (t, *J* = 1.8 Hz, 1H), 6.59 (d, *J* = 1.7 Hz, 2H), 6.25 (d, *J* = 9.3 Hz, 1H), 5.31 (d, *J* = 15.5 Hz, 1H), 5.13 (s, 2H), 0.92 (s, 18H); **^{13}C NMR (100 MHz, $CDCl_3$)** δ 166.83, 155.51, 151.00, 148.09, 147.25, 142.89, 140.93, 139.20, 136.00, 134.46, 133.68, 132.04, 131.22, 130.96, 128.79, 128.72, 128.57, 128.53, 128.41, 127.45, 127.02, 126.57, 125.90, 124.02, 123.11, 121.63, 120.41, 119.80, 118.12, 66.55, 34.57, 31.08; **HRMS (ESI, m/z)** calcd for $C_{42}H_{40}N_2O_7$ $[M+Na]^+$ = 707.2733, found = 707.2739; **HPLC analysis**: 92% ee (IE column, 25 ^{o}C, n-hexane/i-PrOH = 90/10, 1.0 mL/min, λ = 254 nm), Rt (major) = 18.6 min, Rt (minor) = 13.9 min.

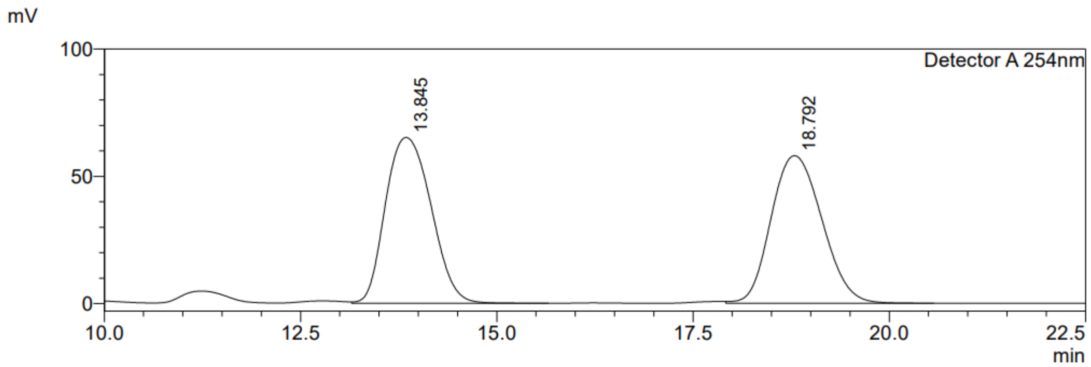

Detector A 254nm

Peak#	Ret. Time	Height	Height%	Area	Area%
1	13.845	65186	52.908	2642660	50.220
2	18.792	58021	47.092	2619511	49.780
Total		123207	100.000	5262172	100.000

Racemic **46**

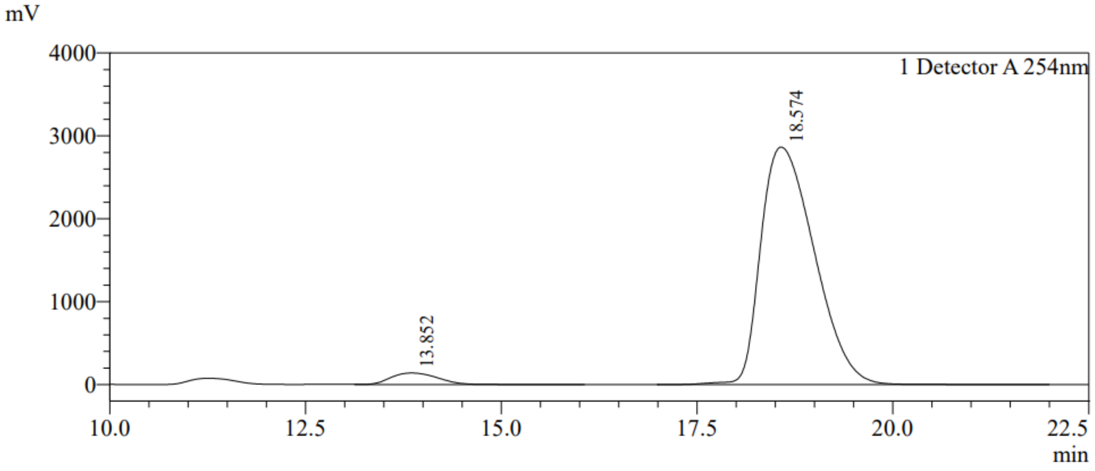

Peak Table

Detector A 254nm

Peak#	Ret. Time	Height	Height%	Area	Area%
1	13.852	139450	4.641	5861659	4.119
2	18.574	2865200	95.359	136430151	95.881
Total		3004650	100.000	142291809	100.000

Enantiomerically enriched **46**

tert-butyl(_S_,2_E_,4_Z_)-5-(3,5-di-_tert_-butylphenyl)-4-(2-(2,4-dinitrophenoxy)naphthalen-1-yl)penta-2,4-dienoate (47)

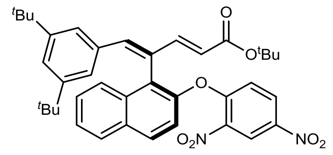

47

A yellow solid, 92% yield, 59.9 mg; m.p: 99-101 °C; **$[\alpha]^{25}_{D}$** = +270.2 (c = 0.25 in $CHCl_3$); **^{1}H NMR (400 MHz, $CDCl_3$)** δ 8.68 (dd, *J* = 2.8, 1.7 Hz, 1H), 8.04 – 7.97 (m, 2H), 7.87 (dt, *J* = 8.5, 1.8 Hz, 1H), 7.67 – 7.51 (m, 4H), 7.30 – 7.26 (m, 1H), 7.17 (dt, *J* = 3.5, 1.8 Hz, 2H), 6.58 (d, *J* = 1.7 Hz, 2H), 6.25 (dd, *J* = 9.3, 1.5 Hz, 1H), 5.18 (dd, *J* = 15.4, 1.5 Hz, 1H), 1.44 (d, *J* = 1.7 Hz, 9H), 0.92 (d, *J* = 1.6 Hz, 18H); **^{13}C NMR (100 MHz, $CDCl_3$)** δ 166.31, 155.59, 150.93, 147.28, 146.44, 141.97, 140.88, 139.20, 134.61, 133.79, 132.03, 131.33, 130.84, 128.73, 128.49, 127.41, 126.96, 126.83, 126.08, 123.92, 122.87, 122.25, 121.61, 120.51, 118.27, 34.56, 31.09, 28.25; **HRMS (ESI, m/z)** calcd for $C_{39}H_{42}N_2O_7$ $[M+Na]^+$ = 673.2890, found = 673.2889; **HPLC analysis**: 95% ee (IE column, 25 °C, n-hexane/*i*-PrOH = 95/5, 1.0 mL/min, λ = 254 nm), Rt (major) = 14.4 min, Rt (minor) = 10.9 min.

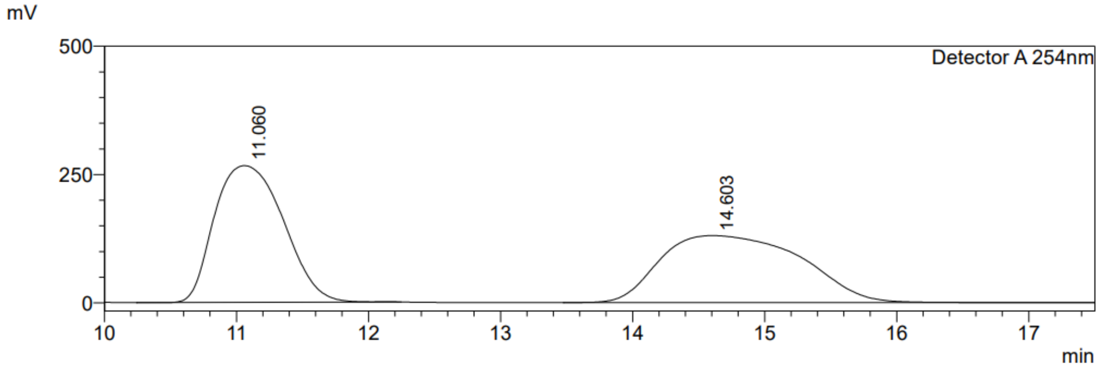

Detector A 254nm

Peak#	Ret. Time	Height	Height%	Area	Area%
1	11.060	266336	67.111	9685033	49.763
2	14.603	130525	32.889	9777282	50.237
Total		396861	100.000	19462315	100.000

Racemic **47**

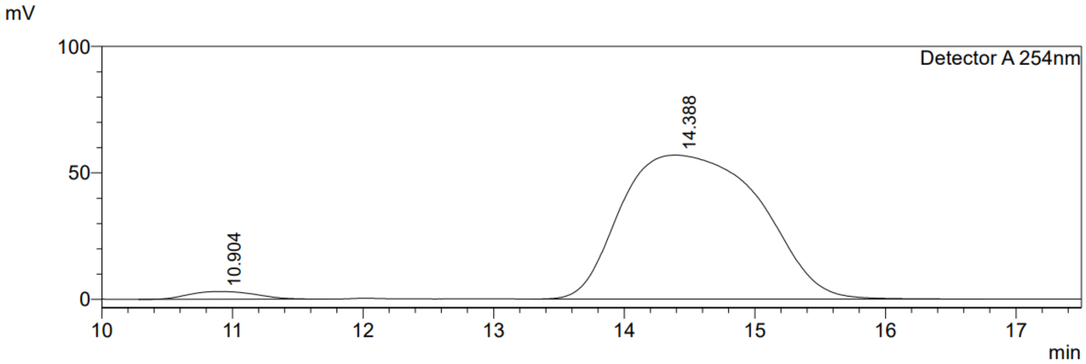

Detector A 254nm

Peak#	Ret. Time	Height	Height%	Area	Area%
1	10.904	3051	5.089	108127	2.482
2	14.388	56892	94.911	4248219	97.518
Total		59942	100.000	4356346	100.000

Enantiomerically enriched **47**

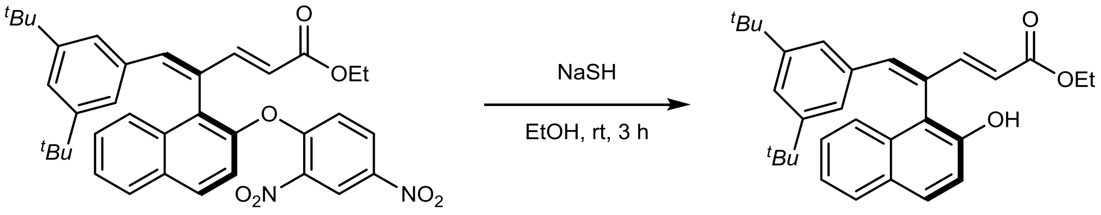

To a round bottle flask with a magnetic stirring bar were added **44** (27.4 mg, 0.10 mmol) and NaSH (22.3 mg, 0.30 mmol), followed by the addition of ethanol (2.0 mL). The reaction mixture was stirred at roomtempreture for 3 h, and TLC show that the reaction was completed. Then, the solvent was removed under reduced pressure, and the residue was purified by column chromatography on silica gel (Petroleum ether/ethyl acetate = 5/1) to afford target product **45**.

ethyl (*S*, 2*E*, 4*Z*)-5-(3,5-di-*tert*-butylphenyl)-4-(2-hydroxynaphthalen-1-yl)penta-2,4-dienoate (45)

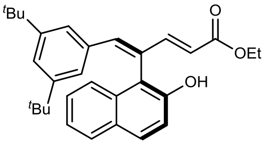

45

A yellow solid, 95% yield, 43.4 mg; m.p: 74-76 ^{o}C; **$[\alpha]^{25}_{D}$** = (c = 0.25 in $CHCl_3$); **^{1}H NMR (400 MHz, $CDCl_3$)** δ 7.91 – 7.81 (m, 3H), 7.56 (ddd, *J* = 7.9, 1.7, 0.8 Hz, 1H), 7.47 (s, 1H), 7.41 – 7.32 (m, 2H), 7.24 (d, *J* = 8.9 Hz, 1H), 7.20 (t, *J* = 1.8 Hz, 1H),

6.85 (d, J = 1.8 Hz, 2H), 5.39 (d, J = 15.4 Hz, 1H), 5.17 (s, 1H), 4.14 (m, J = 7.1, 1.1 Hz, 2H), 1.23 (t, J = 7.1 Hz, 3H), 0.99 (s, 18H); **^{13}C NMR (100 MHz, $CDCl_3$)** δ 167.33, 150.93, 149.77, 147.92, 144.45, 134.03, 132.31, 130.48, 130.30, 129.57, 128.38, 127.35, 124.49, 124.33, 123.93, 123.75, 120.16, 117.84, 115.79, 60.52, 34.63, 31.12, 14.34; **HRMS (ESI, m/z)** calcd for $C_{31}H_{36}O_3$ $[M+Na]^+$ = 479.2562, found = 479.2563; **HPLC analysis**: 91% ee (IE column, 25 °C, n-hexane/*i*-PrOH = 95/5, 0.8 mL/min, λ = 254 nm), Rt (major) = 14.7 min, Rt (minor) = 9.0 min.

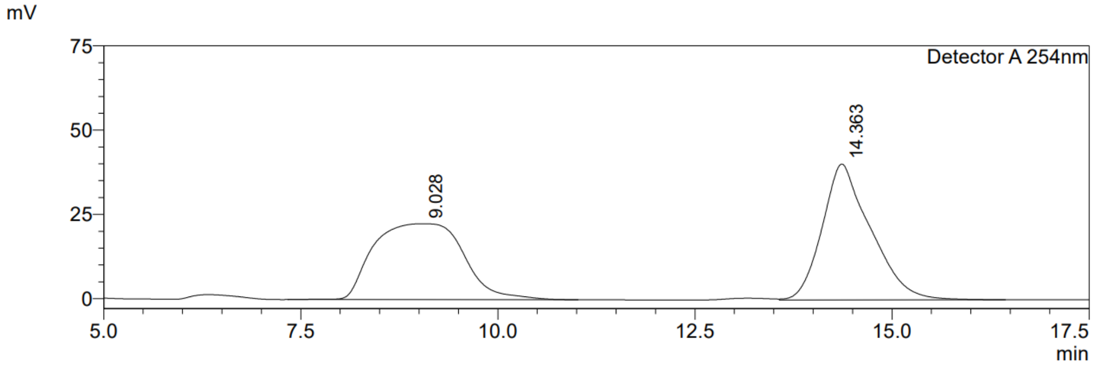

Detector A 254nm

Peak#	Ret. Time	Height	Height%	Area	Area%
1	9.028	22522	35.853	1786499	50.925
2	14.363	40296	64.147	1721606	49.075
Total		62819	100.000	3508106	100.000

Racemic **45**

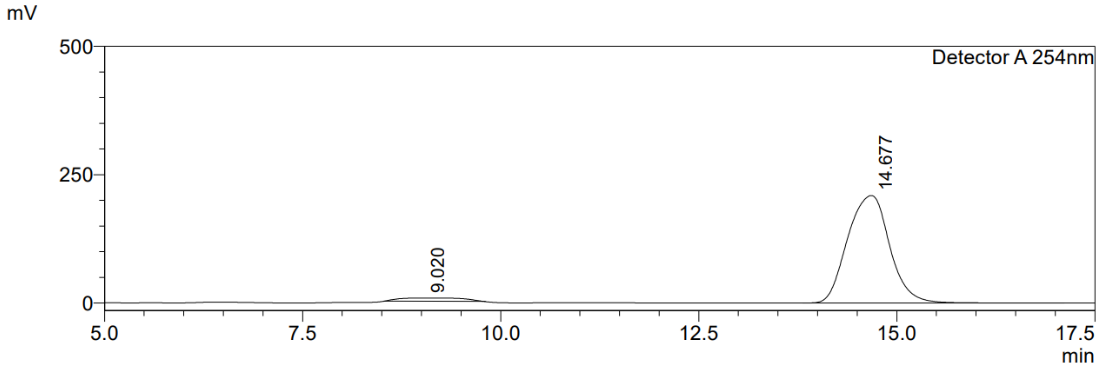

Detector A 254nm

Peak#	Ret. Time	Height	Height%	Area	Area%
1	9.020	6214	2.883	353211	4.366
2	14.677	209286	97.117	7736383	95.634
Total		215499	100.000	8089593	100.000

Enantiomerically enriched **45**

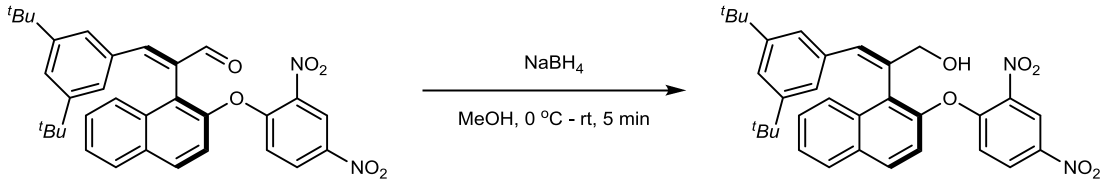

17 **48**

To a solution of **17** (55.2 mg, 0.1 mmol) in 2 mL of methanol was added sodium borohydride (7.6 mg, 0.2 mmol) in portions at 0 ℃. The reaction was continued for another 5 minutes (monitored by TLC). Water (5 mL) was added, and then the mixture was neutralized with a saturated solution of potassium bicarbonate (5 mL). The aqueous solution was extracted twice with ethyl acetate (10 mL). The combined organic phase was washed with brine and dried over sodium sulfate. The solvent was removed under reduced pressure to give a crude residue. Further purification by preparative TLC (Petroleum ether/ethyl acetate = 10: 1) gave compound **48** as a yellow solid (85% yield).

(*S*, *E*)-3-(3,5-di-*tert*-butylphenyl)-2-(2-(2,4-dinitrophenoxy)naphthalen-1-yl)prop-2-en-1-ol (48)

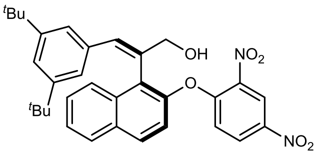

48

A yellow solid, 85% yield, 47.1 mg; m.p: 75-78 ^{o}C; **$[\alpha]^{25}_{D}$** = +278.3 (c = 0.25 in $CHCl_3$); **^{1}H NMR (400 MHz, $CDCl_3$)** δ 8.72 (d, *J* = 2.7 Hz, 1H), 8.10 – 8.04 (m, 1H), 8.02 – 7.98 (m, 1H), 7.97 (d, *J* = 8.8 Hz, 1H), 7.66 – 7.58 (m, 2H), 7.42 (dd, *J* = 9.3, 2.8 Hz, 1H), 7.21 (d, *J* = 8.9 Hz, 1H), 7.10 (t, *J* = 1.8 Hz, 1H), 6.96 (s, 1H), 6.53 (d, *J* = 1.7 Hz, 2H), 6.11 (d, *J* = 9.3 Hz, 1H), 4.63 – 4.50 (m, 2H), 0.89 (s, 18H); **^{13}C NMR (100 MHz, $CDCl_3$)** δ 155.83, 150.75, 146.75, 140.64, 134.82, 134.05, 132.15, 131.21, 130.38, 128.97, 128.92, 128.38, 127.59, 126.90, 125.93, 123.00, 121.77, 121.48, 120.30, 117.72, 68.37, 34.51, 31.10; **HRMS (ESI, m/z)** calcd for $C_{33}H_{34}N_2O_6$ $[M+Na]^+$ = 577.2315, found = 577.2309; **HPLC analysis**: 95% ee (IE column, 25 ^{o}C, n-hexane/*i*-PrOH = 95/5, 1.0 mL/min, λ = 254 nm), Rt (major) = 29.3 min, Rt (minor) = 19.0 min.

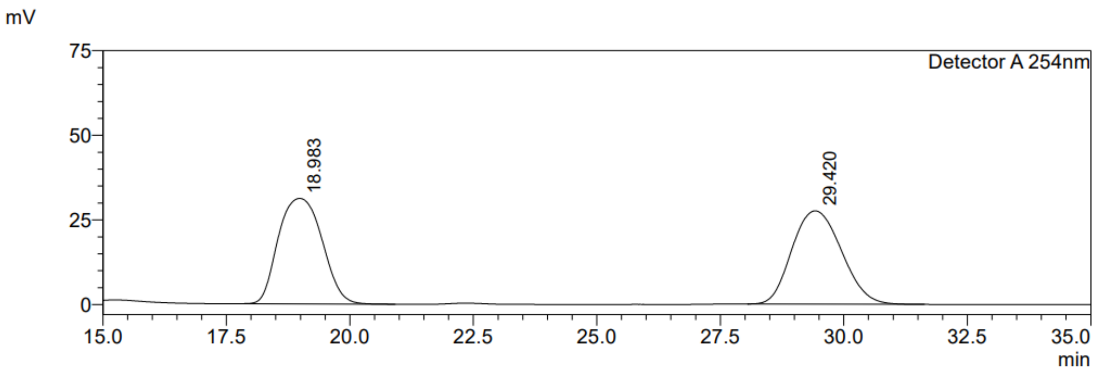

Detector A 254nm

Peak#	Ret. Time	Height	Height%	Area	Area%
1	18.983	31136	53.055	1924295	50.020
2	29.420	27550	46.945	1922794	49.980
Total		58686	100.000	3847090	100.000

Racemic **48**

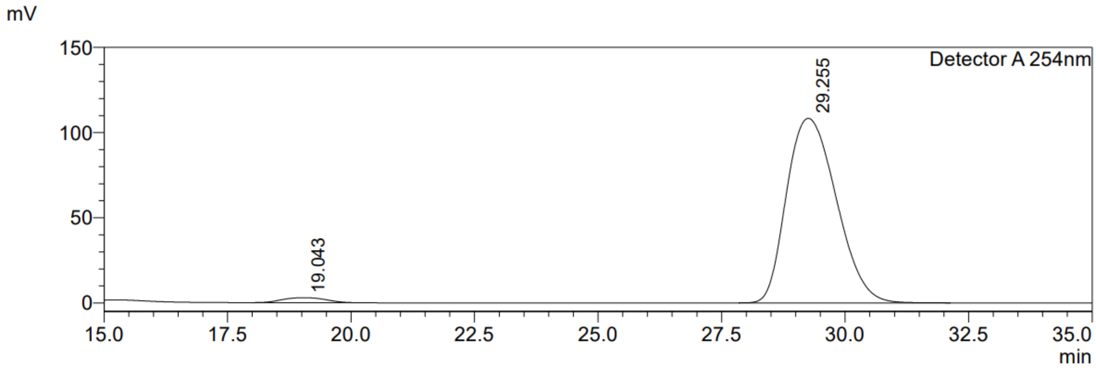

Detector A 254nm

Peak#	Ret. Time	Height	Height%	Area	Area%
1	19.043	2895	2.603	177311	2.281
2	29.255	108302	97.397	7596700	97.719
Total		111197	100.000	7774011	100.000

Enantiomerically enriched **48**

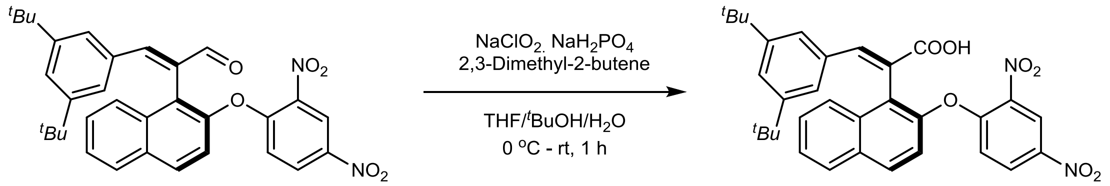

17 **49**

To a solution of **17** (55.2 mg, 0.1 mmol) in 2 mL of THF/^{t}BuOH/H_2O = 4/4/1 was added sodium dihydrogen phosphate (120 mg, 1 mmol), 2-methylbut-2-ene (126.2 mg, 1.5 mmol) and then sodium chlorite (25.3 mg, 0.28 mmol) in portions at 0 ℃. The reaction was continued for another hour (monitored by TLC). Water (5 mL) was added, the aqueous solution was extracted twice with 10 mL of ethyl acetate. The

combined organic phase was washed with brine and dried over sodium sulfate. The solvent was removed under reduced pressure to give a crude residue. Further purification by preparative TLC (Petroleum ether/ethyl acetate = 2 : 1) gave compound **49** as a yellow solid (89% yield).

(*S*, *E*)-3-(3,5-di-*tert*-butylphenyl)-2-(2-(2,4-dinitrophenoxy)naphthalen-1-yl)acrylic acid (49)

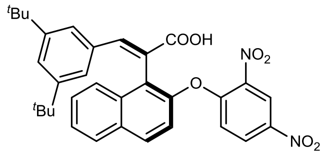

49

A yellow solid, 89% yield, 50.6 mg; m.p: 108-110 °C; **$[\alpha]^{25}_{D}$** = +92.7 (c = 0.25 in $CHCl_3$); **^{1}H NMR (400 MHz, $CDCl_3$)** δ 8.73 (d, *J* = 2.8 Hz, 1H), 8.20 (s, 1H); 8.03 – 7.92 (m, 2H), 7.90 – 7.85 (m, 1H), 7.81 (dd, *J* = 9.3, 2.8 Hz, 1H), 7.61 – 7.52 (m, 2H), 7.24 (s, 1H), 6.79 (d, *J* = 1.8 Hz, 2H), 6.63 (d, *J* = 9.3 Hz, 1H), 0.97 (s, 18H); **^{13}C NMR (100 MHz, $CDCl_3$)** δ 172.37, 155.63, 151.03, 148.15, 147.59, 141.16, 139.13, 133.66, 132.90, 131.94, 131.07, 128.70, 128.30, 127.94, 126.76, 125.53, 125.47, 125.06, 124.56, 122.68, 121.82, 120.00, 118.59, 34.61, 31.07; **HRMS (ESI, m/z)** calcd. for $C_{33}H_{32}N_2O_7$ $[M+Na]^+$ = 591.2107, found = 591.2110; **HPLC analysis**: 95% ee (ADH column, 25 °C, n-hexane/*i*-PrOH = 90/10, 1.0 mL / min, λ = 254 nm), Rt (major) = 6.7 min, Rt (minor) = 15.7 min.

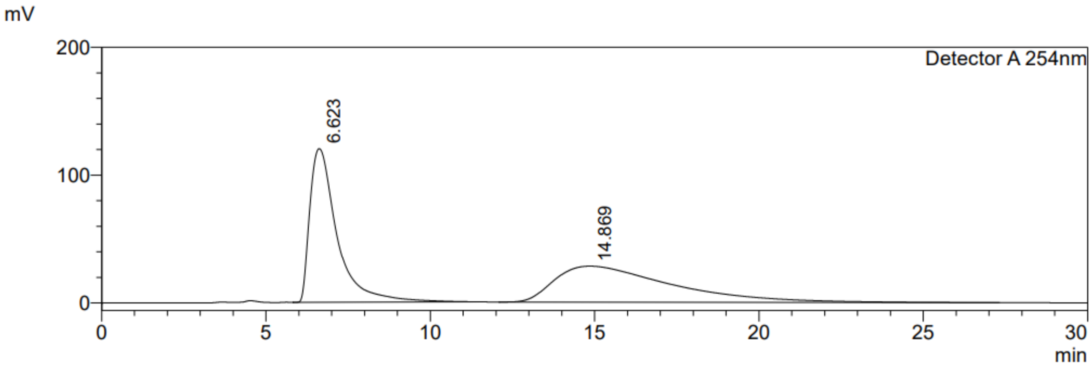

Detector A 254nm

Peak#	Ret. Time	Height	Height%	Area	Area%
1	6.623	120164	81.021	6950215	49.979
2	14.869	28149	18.979	6956191	50.021
Total		148313	100.000	13906406	100.000

Racemic **49**

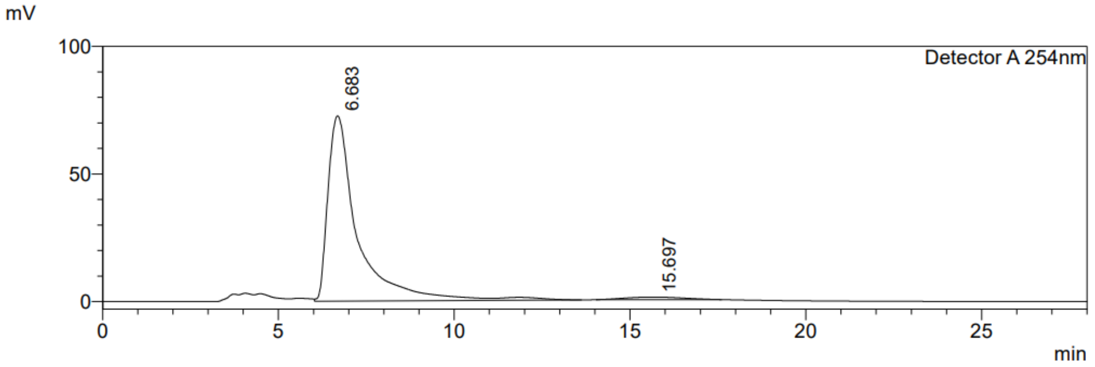

Detector A 254nm

Peak#	Ret. Time	Height	Height%	Area	Area%
1	6.683	72560	98.662	4279220	97.403
2	15.697	984	1.338	114084	2.597
Total		73544	100.000	4393304	100.000

Enantiomerically enriched **49**

7. Study on axial stability of products

Following the procedure by Curran,[3] atropisomeric compound was dissolved in DMSO or *o*-xylene under nitrogen to make a 5 mg/mL solution in 25 mL schlenk tube. The tube was sealed and placed in a pre-equilibrated oil bath with electronic temperature controller. At various time intervals, and the enantiomeric ratio was measured by analytical HPLC column. This ee was plotted against time, and the barrier to rotation was calculated from the plot. The "m" stands for the % of the minor enantiomer, and "M" denotes the % of the major enantiomer. The half-life for racemization at ambient temperature is determined by first calculating the rate of racemization (k_{rac}) from the barrier to rotation at 25 ℃, assuming that $\Delta G^{\ddagger}_{rot}$ is mostly constant over a large temperature range.

k_B = Boltzmann's constant [1.381 ×10^{-23} J K^{-1}].

T = temperature in K.

h = Planck's constant [6.626 x 10^{-34}J s].

R = gas constant [8.3145 J mol^{-1}].

ln [(M + m) / (M - m)] = ln [100 / ee].

7.1 Test experiment of axial stability for 3 (50 ℃).

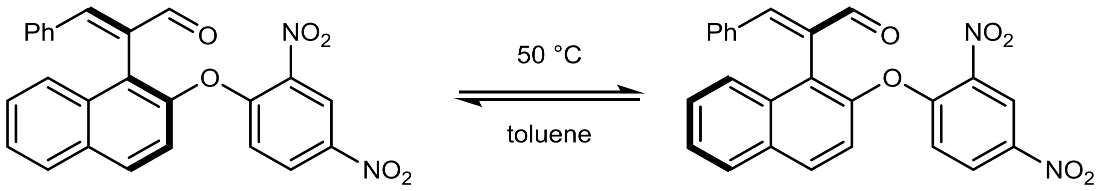

3 3

Supplementary Table 5: ee (**3**) vs time.

t/h	ee of **3** (%)
0	95.3
1	88.4
2	84.1
3	78.6
4	75.5
5	71.3
6	68.5

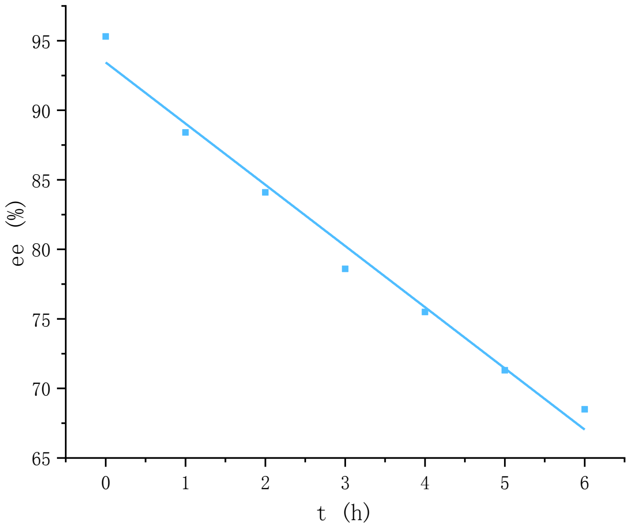

Supplementary Figure 2. ee (**3**) vs time.

7.2 Determination of $t_{1/2}^{rac}$ for 3 (50 ℃).

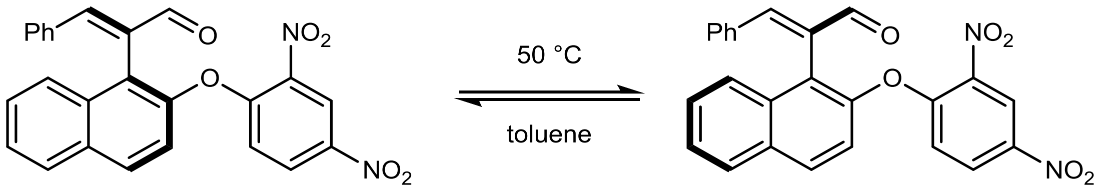

3 **3**

Supplementary Table 6: ee (**3**) vs time.

t (h)	t (10^3 s)	ee of **3** (%)	ln [100/ee (**3**)]
0	0	95.3	0.0513
1	3.6	88.4	0.1278
2	7.2	84.1	0.1744
3	10.8	78.6	0.2357
4	14.4	75.5	0.2744
5	18	71.3	0.3424
6	21.6	68.5	0.0513

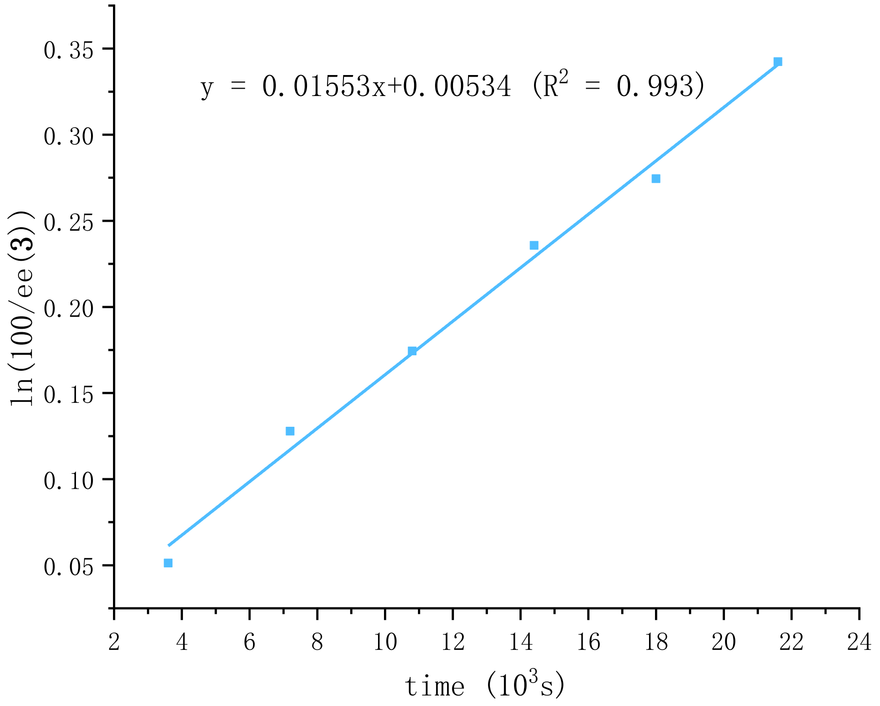

Supplementary Figure 3. ln (100/ee (**3**)) vs time.

$$k_{rac} = 2k_{rot} = \frac{2k_B T}{h} e^{\left(\frac{-\Delta G^{\ddagger}_{rot}}{RT}\right)} \qquad t_{1/2} = \frac{\ln 2}{k_{rac}}$$

$$k_{rot} = \frac{slope}{2} = \frac{1.553 \times 10^{-5} s^{-1}}{2} = 7.765 \times 10^{-6} s^{-1}$$

$$K^{\ddagger}_{rot} = \frac{k_{rot} h}{KT} = \frac{(7.765 \times 10^{-6} s^{-1})(6.626 \times 10^{-34} J \cdot s)}{(1.381 \times 10^{-23}\, J/K)(323.15K)} = 1.1529 \times 10^{-18}$$

$$\Delta G^{\ddagger}_{rot} = -RT \ln K^{\ddagger}_{rot} = -(0.00831 kJ/mol)(323.15K)\ln(1.1529 \times 10^{-18})$$
$$= 110.9174 kJ/mol = 26.5353\ kcal/mol$$

$$t_{1/2} for\ racemization\ at\ 50\ °C = 13.4\ h$$

8. Determination of absolute configuration of substrates and products

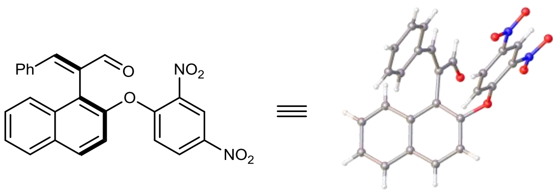

3
CCDC：2162443

Supplementary Table 7. Crystal data and structure refinement for **3**

Identification code	**3**
Empirical formula	$C_{25}H_{16}N_2O_6$
Formula weight	440.40
Temperature/K	243.0
Crystal system	monoclinic
Space group	$P2_1$
a/Å	7.9010(5)
b/Å	8.2619(5)
c/Å	16.3464(11)
α/°	90
β/°	101.325(4)
γ/°	90
Volume/Å^3	1046.27(12)
Z	2
ρ_{calc}g/cm^3	1.398
μ/mm^{-1}	0.846

F(000)	456.0
Crystal size/mm^3	0.42 × 0.34 × 0.09
Radiation	CuKα (λ = 1.54178)
2Θ range for data collection/°	5.514 to 131.028
Index ranges	$-8 \le h \le 9$, $-9 \le k \le 9$, $-19 \le l \le 19$
Reflections collected	10834
Independent reflections	3535 [R_{int} = 0.0473, R_{sigma} = 0.0433]
Data/restraints/parameters	3535/1/298
Goodness-of-fit on F^2	1.048
Final R indexes [I>=2σ (I)]	R_1 = 0.0345, wR_2 = 0.0938
Final R indexes [all data]	R_1 = 0.0367, wR_2 = 0.0959
Largest diff. peak/hole / e $Å^{-3}$	0.16/-0.16
Flack parameter	-0.09(12)

9. Mechanism studies

A). Model reaction catalyzed by different phosphonium salts

We also prepared the methylated of phosphonium salt catalysts **P10-1**, **P10-2** and **P10-3** When H-Blocked phosphonium salts **P10-1**, **P10-2** and **P10-3** was used, the enantioselectivity the model reaction decreased dramatically. These preliminary results indicated the importance of both hydrogen-bonding and ion-pair interactions in this phase-transfer system (Supplementary Figure 4).

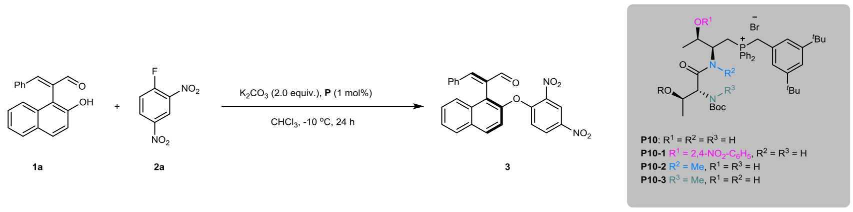

cat.	sol.	t (h)	results (3)
P10	$CHCl_3$	24 h	99% y, 96% ee
P10-1	$CHCl_3$	24 h	92% y, -29% ee
P10-2	$CHCl_3$	24 h	99% y, 26% ee
P10-3	$CHCl_3$	24 h	99% y, 64% ee

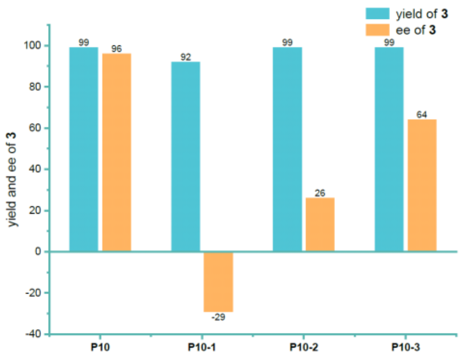

Supplementary Figure 4. Model reaction catalyzed by different phosphonium salts

B). Non-linear effect

Reactions were performed with **1a** (0.1 mmol), **2a** (0.12 mmol), **P10** with different ee value (1 mol%) and K_2CO_3 (0.20 mmol) in $CHCl_3$ (1 mL) at -10 °C. The ee value was determined by HPLC analysis on a chiral stationary phase. The catalytic results were shown in below. The linear relationship between the enantiopurities of **P10** and **1a** indicated that a monomeric phosphonium salt molecule might participate in the stereodeterming step.

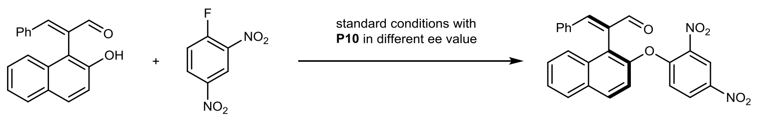

1a (0.1 mmol) **2a** (0.12 mmol) **3**

Entry	ee of **P10** (%)	ee of **3** (%)
1	+100	+96
2	+60	+61
3	+20	+23
4	-20	-19
5	-60	-57
6	-100	-95

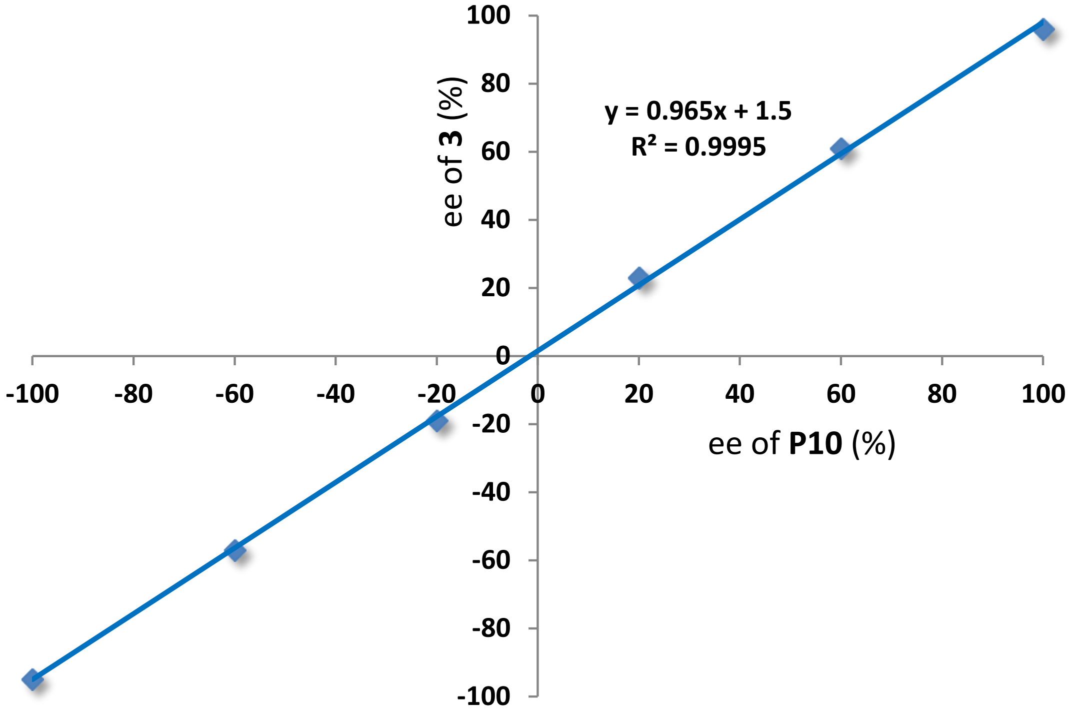

Supplementary Figure 5. Non-linear effect.

C). ^{1}H NMR experiments

(1). Hydrogen-bonding titration of **P10** with **1a**

P10 (18.2 mg, 0.02 mmol) was dissolved in $CDCl_3$ (0.5 mL) and then **1a** was stepwise added to the solution of phosphonium salt **P10** in $CDCl_3$. A ^{1}H-NMR spectrum was recorded after each addition. ^{1}H-NMR spectra were recorded on a Bruker spectrometer and calibrated to the residual $CDCl_3$ solvent peak.

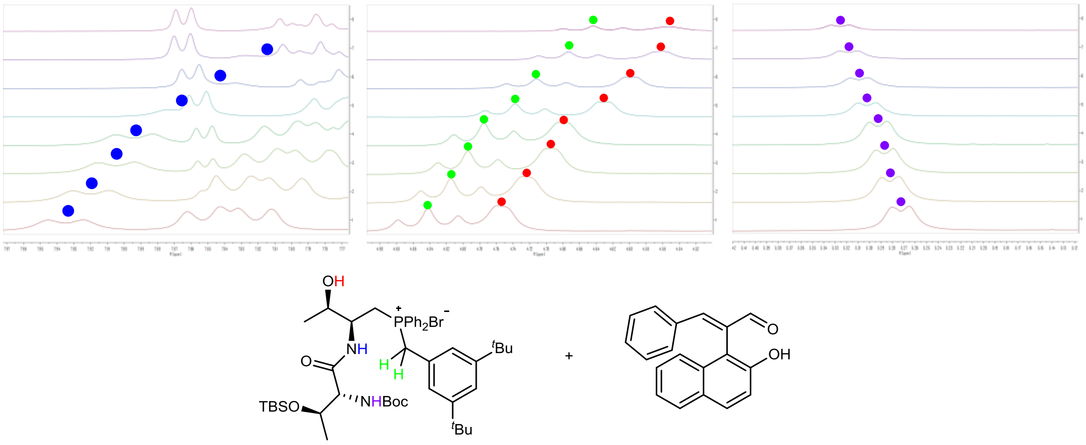

Supplementary Figure 6. Hydrogen-bonding titration with **1a**

Supplementary Table 8 Chemical shift of H in **P10**

Entry	equivalent of **1a**	Chemical shift of OH [ppm]	Chemical shift of NH [ppm]	Chemical shift of NH [ppm]	Chemical shift of CH [ppm]
1	0.000	4.7591	7.9453	5.2801	4.842
2	0.111	4.7273	7.9307	5.2891	4.8147
3	0.250	4.6981	7.9153	5.2941	4.7941
4	0.429	4.6831	7.9047	5.3001	4.7749
5	0.667	4.6360	7.8740	5.3097	4.7374
6	1.000	4.6040	7.8545	5.3165	4.7115
7	1.500	4.5653	7.8275	5.3258	4.6732
8	2.333	4.5570	-	5.3336	4.6438

(2). Job plot analysis

General procedure for Job plot: ^{1}H NMR titration was conducted in chloroform-d. The total concentration of **P10** and **1a** was 0.08 mmol/mL. The proportion of the concentration of **P10** vs the total concentration varied from 0.3 to 0.9. The chemical shifts of the oxhydryl O-H on the **P10** were recorded. A 1:1 binding pattern could be observed in the Job plot (Supplementary Figure 6).

Supplementary Table 9 Job plot analysis

Entry	c (**P10**)	c (**1a**)	c (**P10**)/0.08	△δ	Δδ*10^4*c(**P10**)/0.08
1	0.08	0	1	0	0
2	0.072	0.008	0.9	0.0318	286.2
3	0.064	0.016	0.8	0.061	488
4	0.056	0.024	0.7	0.076	532
5	0.048	0.032	0.6	0.1231	738.6
6	0.04	0.04	0.5	0.1551	775.5
7	0.032	0.048	0.4	0.1938	775.2
8	0.024	0.056	0.3	0.2021	606.3

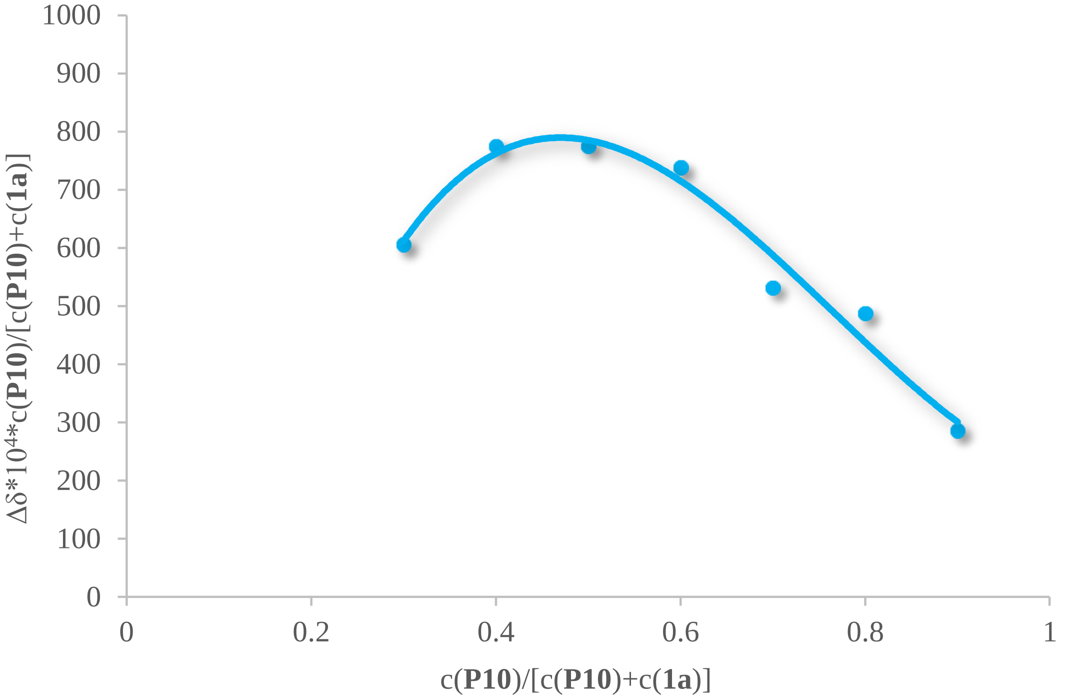

Supplementary Figure 6. Job plot analysis

D). ^{19}F NMR experiments

P10 (18.2 mg, 0.02 mmol) was dissolved in $CDCl_3$ (0.5 mL) and then **2a** was stepwise added to the solution of phosphonium salt **P10** in $CDCl_3$. A ^{19}F NMR spectrum was recorded after each addition.

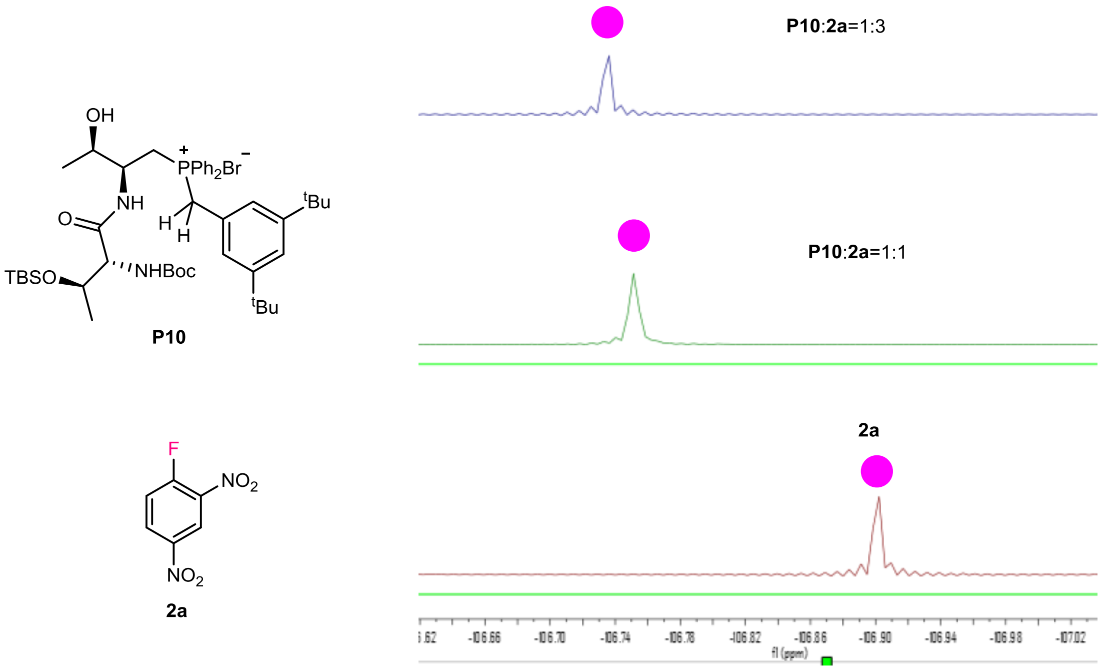

Supplementary Figure 7. Hydrogen-bonding titration with **2a**

E). Kinetic experiments

Monitored the reaction of **1a** and **2a** under standard conditions, measured the yield and changes in enantiomeric excess (ee) of **1a** and **3** during the reaction.

Supplementary Table 10. Kinetic experimental data

t(h)	yield(%) of **3**	ee(%) of **3**	ee(%) of **1a**
1	23.5	86	0
2	31.5	88	0
4	61.5	92	0
6	80	94	0
12	99	96	

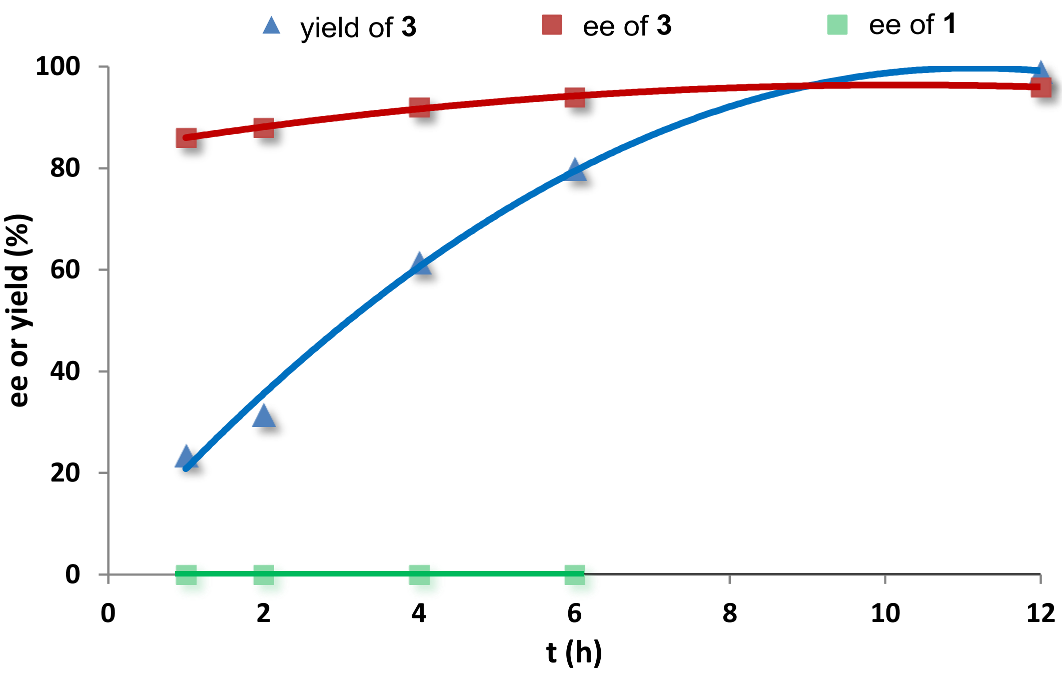

Supplementary Figure 8. Kinetic experiment curve

10. DFT studies

Computational details: All calculations were performed using Gaussian 09 program package[4]. Geometries were optimized in chloroform solvent and characterized by frequency analysis at 263 K, using M062X functional[5] with Grimme's D3 dispersion correction[6,7] and 6-31G(d, p) basis set. The self-consistent reaction field (SCRF) and SMD solvation model[8] were adopted to evaluate the effect of solvent in the structural optimization. Single-point energies were obtained at the M062X-D3/6-311+G(d, p)(SMD, chloroform) level of theory. The intrinsic reaction coordinate (IRC) path was traced to check the energy profile connecting each transition state to two associated minima of the proposed mechanism[9]. Non-covalent interaction (NCI) analysis by using independent gradient model based on Hirshfeld partition (IGMH)[10] were performed by Multiwfn 3.8 (dev)[11], and the results were visualized by VMD[12] software. The H-bond binding energy (E_{HB}) was evaluated using the following equation[13]:

$$E_{HB}\text{ (kcal/mol)} = -223.08 \times \rho_{BCP} + 0.7423$$

where the electron density (ρ, a.u.) at the (3, -1) bond critical point (BCP) was obtained by Atoms-in-Molecules (AIM) analysis[14]. The optimized geometries of all stationary points were visualized using CYLView software[15].

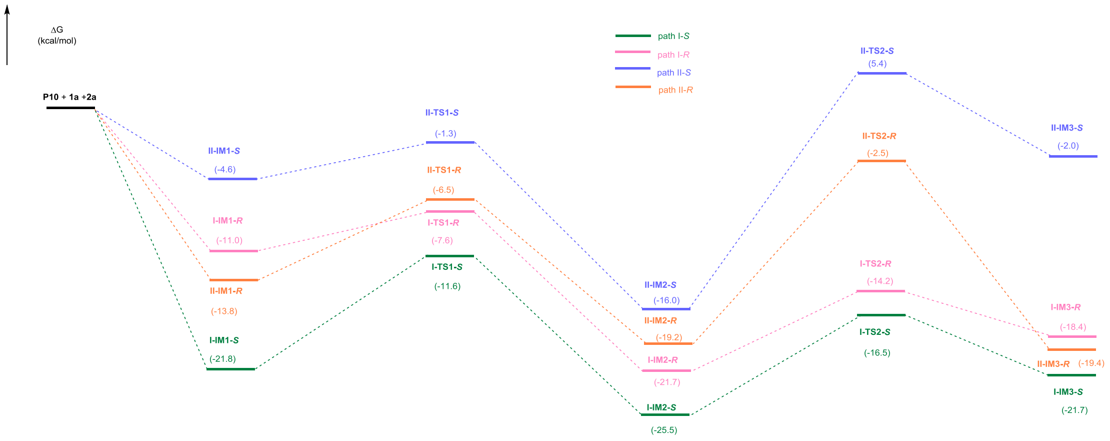

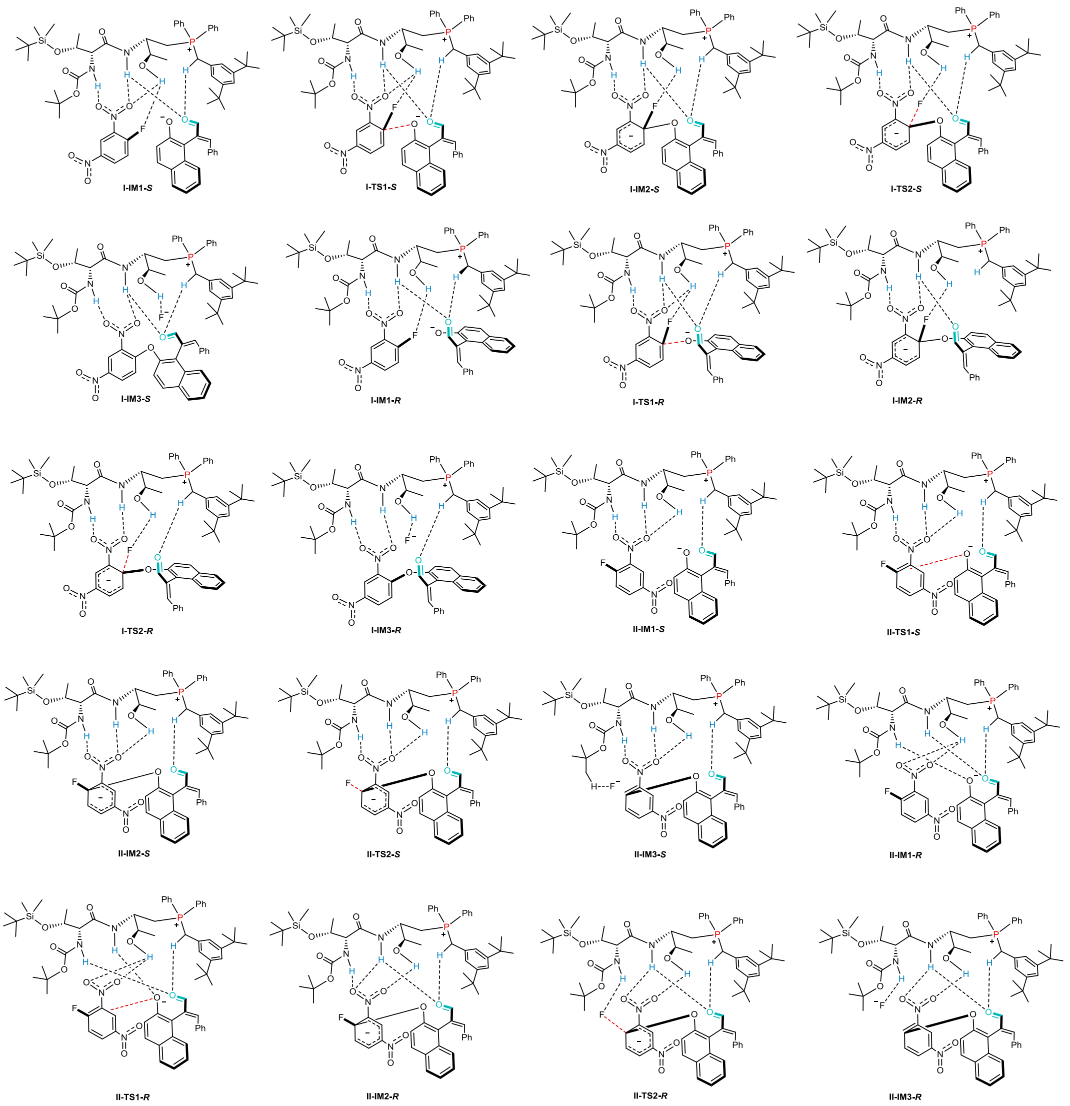

Supplementary Figure 9. Energy profiles for S_NAr reaction between **1a** and **2a** catalyzed by phosphonium salt catalyst **P10** along four pathways (I-*S*~II-*R*). The relative free energies are given in kcal/mol.

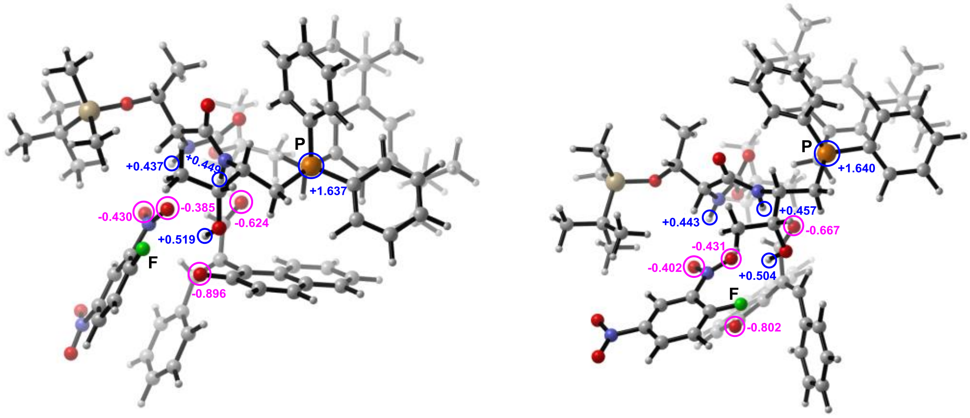

I-IM1-*S* I-IM1-*R*

Supplementary Figure 10. Charge population in I-IM1-*S* and I-IM1-*R*, obtained by natural bond orbital (NBO) analysis.

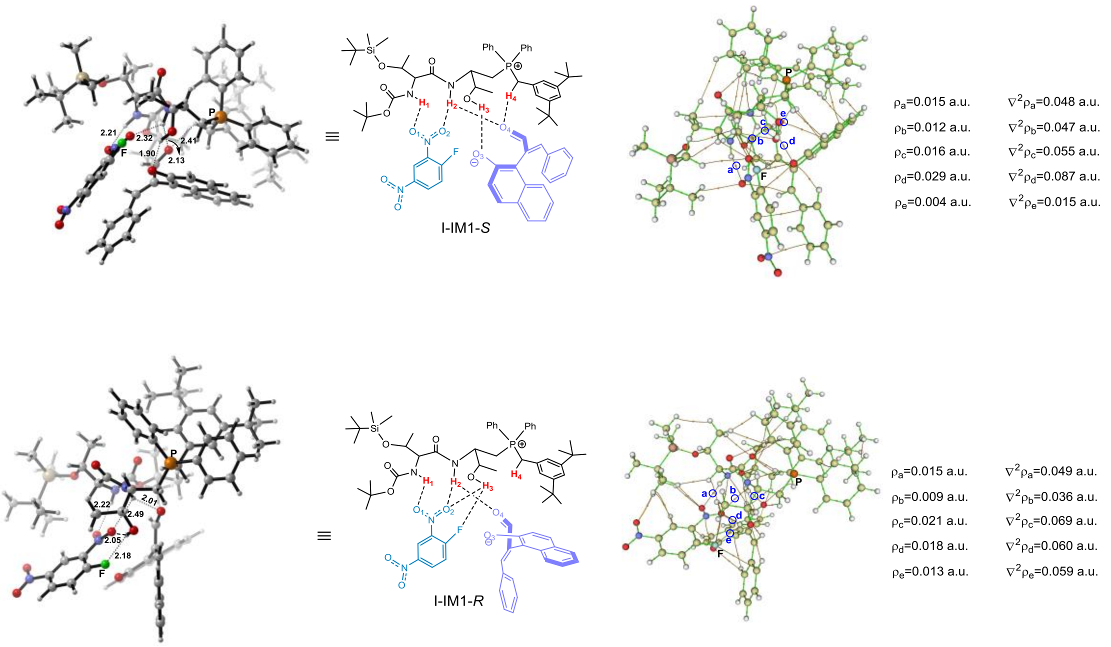

Supplementary Figure 11. Optimized structures of I-IM1-*S* and I-IM1-*R*. The distances are in Å. Laplacian ($\nabla^2\rho$) and electron density (ρ) values of selected bond critical points (BCPs) in I-IM1-*S* and I-IM1-*R*, obtained by AIM analysis.

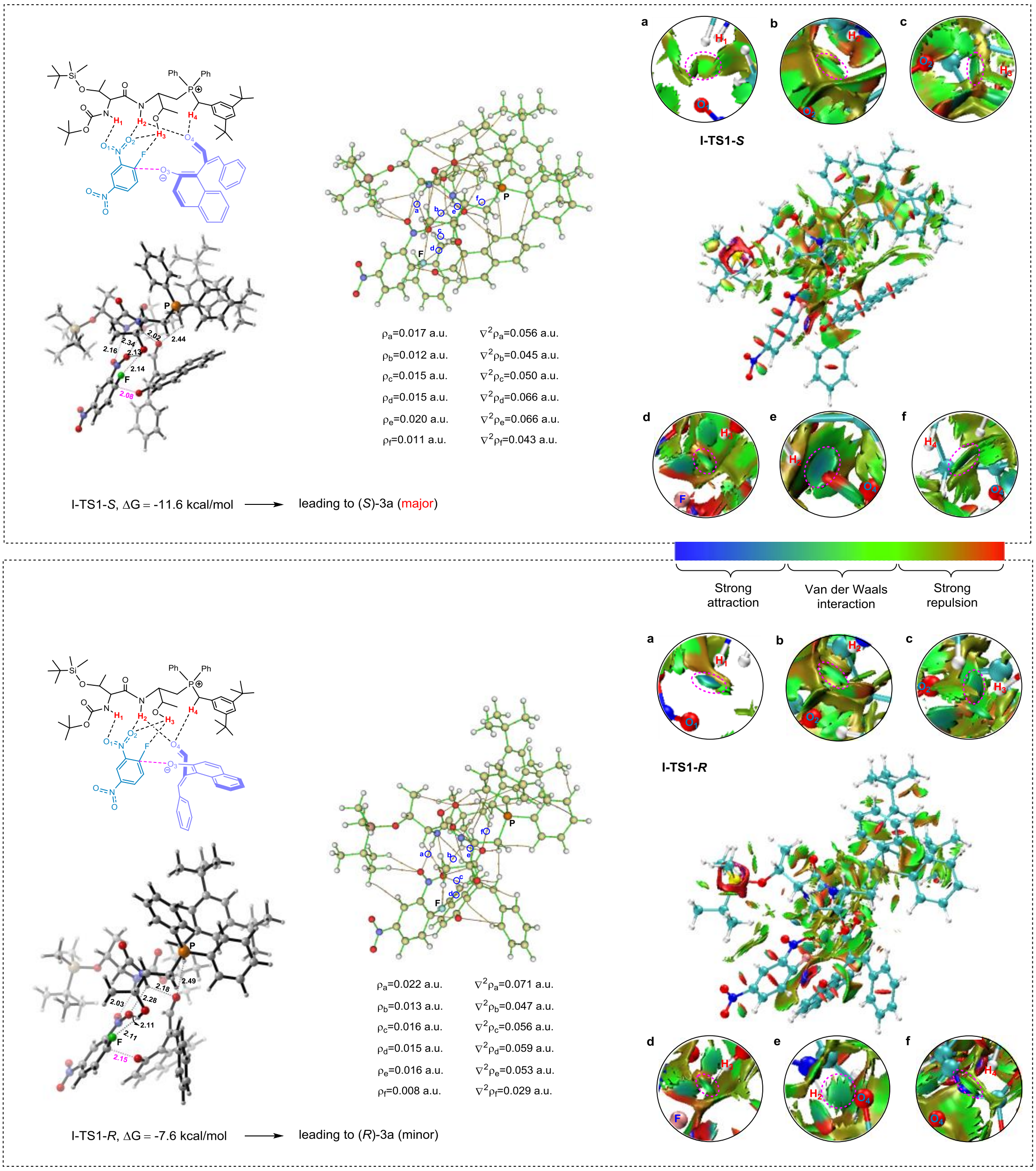

Supplementary Figure 12. Optimized structures of I-TS1-*S* and I-TS1-*R*. The distances are in Å. Laplacian ($\nabla^2\rho$) and electron density (ρ) values of selected bond critical points (BCPs) in I-TS1-*S* and I-TS1-*R*, obtained by AIM analysis. Non-covalent interaction (NCI) plots for I-TS1-*S* and I-TS1-*R*, obtained by Multiwfn and VMD softwares.

Supplementary Table 11. Electron density at the (3, -1) bond critical points (ρ_{BCP}, in a.u.) and H-bond binding energy (E_{HB}, kcal/mol) for transition states **I-TS1-*S***, **I-TS1-*R***, **II-TS1-*S*** and **II-TS1-*R***.

Structures	Bonds	Bond length (Å)	ρ_{BCP}	E_{HB} (kcal/mol)
**I-TS1-*S***	H1···O1	2.16	0.017	-3.05
	H2···O2	2.34	0.012	-1.93

	H3···O2	2.13	0.015	-2.60
	H2···O4	2.02	0.020	-3.72
	H4···O4	2.44	0.011	-1.71
	H3···F	2.14	0.015	-2.60
**I-TS1-*R***	H1···O1	2.03	0.022	-4.17
	H2···O2	2.28	0.013	-2.16
	H2···O4	2.18	0.016	-2.83
	H3···O2	2.11	0.016	-2.83
	H4···O4	2.49	0.008	-1.04
	H3···F	2.11	0.015	-2.60
**II-TS1-*S***	H1···O1	2.23	0.015	-2.60
	H2···O2	2.10	0.018	-3.27
	H3···O2	2.25	0.012	-1.93
	H4···O4	2.29	0.014	-2.38
**II-TS1-*R***	H1···O3	2.07	0.020	-3.72
	H2···O4	2.07	0.019	-3.50
	H3···O2	2.02	0.022	-4.17

Supplementary Table 12. The corrected electronic energies (*E*z), enthalpies (*H*), and Gibbs free energies (*G*) for all stationary points (in Hartree), obtained at M062X-D3/6-31G(d,p)(SMD, chloroform) theoretical level.

Structures	[a]*ZPE*	[b]H_c	[c]G_c	E_z	*H*	*G*	[d]*G*
**I-IM1-*S***	1.48406	1.55762	1.37827	-4532.69746	-4532.62390	-4532.80325	-4533.84848
**I-TS1-*S***	1.48354	1.55653	1.37760	-4532.68217	-4532.60918	-4532.78811	-4533.83230
**I-IM2-*S***	1.48550	1.55808	1.38179	-4532.70896	-4532.63639	-4532.81267	-4533.85451
**I-TS2-*S***	1.48209	1.55510	1.37600	-4532.68213	-4532.60911	-4532.78822	-4533.84006
**I-IM3-*S***	1.48181	1.55516	1.37474	-4532.68477	-4532.61143	-4532.79185	-4533.84836
**I-IM1-*R***	1.48544	1.55874	1.38043	-4532.67904	-4532.60574	-4532.78405	-4533.83138
**I-TS1-*R***	1.48232	1.55602	1.37263	-4532.67080	-4532.59710	-4532.78049	-4533.82593
**I-IM2-*R***	1.48502	1.55703	1.38115	-4532.70317	-4532.63116	-4532.80704	-4533.84838
**I-TS2-*R***	1.48176	1.55501	1.37394	-4532.67764	-4532.60439	-4532.78546	-4533.83649
**I-IM3-*R***	1.48304	1.55630	1.37715	-4532.68254	-4532.60927	-4532.78843	-4533.84303
**II-IM1-*S***	1.48555	1.55832	1.38407	-4532.67342	-4532.60064	-4532.77490	-4533.82115
**II-TS1-*S***	1.48351	1.55632	1.38118	-4532.67009	-4532.59728	-4532.77241	-4533.81586
**II-IM2-*S***	1.48658	1.55886	1.38478	-4532.69737	-4532.62510	-4532.79917	-4533.83936
**II-TS2-*S***	1.48581	1.55778	1.38561	-4532.64543	-4532.57346	-4532.74563	-4533.80516
**II-IM3-*S***	1.48668	1.55917	1.38590	-4532.65858	-4532.58609	-4532.75936	-4533.81698
**II-IM1-*R***	1.48494	1.55788	1.38025	-4532.68671	-4532.61378	-4532.79141	-4533.83575
**II-TS1-*R***	1.48519	1.55705	1.38402	-4532.67945	-4532.60759	-4532.78063	-4533.82409
**II-IM2-*R***	1.48759	1.55978	1.38193	-4532.69633	-4532.62413	-4532.80199	-4533.84445
**II-TS2-*R***	1.48571	1.55788	1.38169	-4532.66784	-4532.59567	-4532.77187	-4533.81784

**II-IM3-*R***	1.48332	1.55598	1.37968	-4532.69296	-4532.62030	-4532.79660	-4533.84464

[a] Zero-point energy;

[b] Thermal correction to enthalpy;

[c] Thermal correction to Gibbs free energy;

[d] Gibbs free energy at the M062X-D3/6-311+G(d,p)(SMD, chloroform)//M062X-D3/6-31G(d,p)(SMD, chloroform) theoretical level.

Cartesian coordinates of DFT-computed structures

I-IM1-*S*

P	2.86872400	-0.16223900	1.79256200
N	-0.09009600	1.21909800	1.55632100
C	-0.37039300	2.54665100	1.46419100
O	0.02763100	3.37828200	2.27241800
C	-1.36398000	2.93391700	0.36936200
N	-1.18675700	2.24796000	-0.88659900
C	1.26510400	-0.56429700	2.56637500
C	0.36990700	0.65215800	2.81522100
C	3.42529400	1.46202400	2.35309100
C	-0.01518700	2.18628900	-1.56508600
C	4.24565900	1.58030500	3.48185500
C	2.94615300	2.60731700	1.70545000
O	-0.23742900	1.69435400	-2.79408800
O	1.06791200	2.54126900	-1.11691300
C	3.67714900	-2.64797600	2.74781200
C	4.09090000	-1.39472700	2.28934400
C	5.45648100	-1.11668100	2.13376000
C	4.62781300	-3.61518800	3.06601300
C	3.31423000	3.86464300	2.17642200
C	4.15029000	3.98227700	3.28362900
C	5.98369200	-3.33417300	2.92703100
C	6.39763900	-2.08568700	2.46132800
C	4.61039700	2.84196300	3.93950900
C	-0.76723200	0.24513600	3.79630600
C	-1.87313200	1.28888800	3.87795900
C	-1.56667000	4.45057100	0.23767500
C	-0.46679000	5.17671200	-0.51024000
C	2.72696700	-0.19003500	-0.02588300
H	-2.31028100	2.54810800	0.76441500
H	-2.01110700	1.87701500	-1.34077500
H	0.75734800	-1.30048800	1.92744500
H	1.48307600	-1.05154500	3.52251600
H	4.60443500	0.69462300	3.99773000
H	2.28129600	2.53137700	0.84696000
H	2.62051900	-2.87943800	2.84876200
H	4.30324200	-4.58658200	3.42521800
H	2.93842700	4.75138900	1.67673600
H	4.43767700	4.96542300	3.64288800
H	6.72189600	-4.08847700	3.18089000
H	5.25333700	2.93193600	4.80887300
H	-1.46588200	2.27699100	4.11002300
H	-2.58110500	1.00087500	4.65943200
H	-2.41992800	1.34738100	2.93184000
H	-1.62684200	4.84767800	1.25990900
H	0.50157400	5.00083100	-0.03649000
H	-0.67730900	6.24961500	-0.51359200
H	-0.42348600	4.83269000	-1.54786000
H	2.24677300	-1.14830000	-0.25871000
H	2.02434100	0.60380400	-0.30839900
H	-0.51265200	0.59865100	0.87033900
H	0.93526500	1.44752100	3.30981000
H	5.78194400	-0.14823800	1.76230600
H	7.45472400	-1.86498500	2.35429200
C	4.07180200	-0.04926300	-0.68909000
C	4.60150200	1.22117300	-0.92132000
C	4.79841700	-1.18434800	-1.04865300
C	5.85885000	1.37808400	-1.50551900
H	4.00198500	2.08685800	-0.66257800
C	6.06201400	-1.06422500	-1.63557300
H	4.36022200	-2.16034600	-0.85671700
C	6.56889700	0.22216400	-1.84666600
H	7.55019800	0.32942700	-2.30273200
C	6.46222200	2.75709100	-1.78492100
C	7.80621300	2.88429800	-1.04963600
H	7.67028400	2.77645100	0.03178200
H	8.52114300	2.12387500	-1.37845600
H	8.25106800	3.86691100	-1.24088100
C	6.68995400	2.91108200	-3.29717800
H	5.74528500	2.81233600	-3.84226400

H	7.11254200	3.89755200	-3.51774800
H	7.38245900	2.15519800	-3.68056800
C	5.54361900	3.88876300	-1.31332100
H	6.01724800	4.85436700	-1.51667000
H	4.58018400	3.87321200	-1.83443800
H	5.35061600	3.83010300	-0.23632600
C	6.90313100	-2.28013700	-2.03624100
C	6.18053100	-3.59466800	-1.73303500
H	5.95963400	-3.69647900	-0.66481800
H	5.23766600	-3.66785500	-2.28505200
H	6.81108300	-4.43948100	-2.02834200
C	7.19931800	-2.22334700	-3.54353400
H	7.76227500	-1.32481100	-3.81379100
H	7.79252700	-3.09350500	-3.84528500
H	6.27007700	-2.22610000	-4.12231100
C	8.22518300	-2.25888100	-1.25230400
H	8.84025300	-3.12412100	-1.52283500
H	8.80506100	-1.35461400	-1.46112100
H	8.03409000	-2.29816500	-0.17453100
O	-2.79523400	4.65179500	-0.45630800
O	-1.26226100	-1.04974000	3.57864400
H	-1.83278000	-1.14462000	2.78687000
H	-0.27193300	0.19227100	4.77636300
Si	-4.24139000	4.87320000	0.36670500
C	-4.38873500	3.65517100	1.79760600
H	-4.45105800	2.61106700	1.46905000
H	-3.53558200	3.74074600	2.48137400
H	-5.28888000	3.87025300	2.38434100
C	-4.29317400	6.61403600	1.06364000
H	-4.22680300	7.36810400	0.27323200
H	-5.21369700	6.79367300	1.62920900
H	-3.45378200	6.77899900	1.74877900
C	-5.58242300	4.57535900	-0.93142600
C	-6.96790700	4.77273000	-0.30349900
H	-7.10162100	5.79056000	0.08081300
H	-7.75428800	4.59798600	-1.05008000
H	-7.14184200	4.07446800	0.52380600
C	-5.46033300	3.14052900	-1.46055000
H	-4.46808600	2.95071000	-1.88441100
H	-5.63467700	2.39939100	-0.67168700
H	-6.20285300	2.95865700	-2.24945800
C	-5.39844800	5.55885500	-2.09460500
H	-5.49269000	6.60009800	-1.76577200
H	-4.41723800	5.44179400	-2.56696400

H	-6.16337000	5.38677300	-2.86409200
C	0.82443200	1.61742400	-3.78856000
C	1.97325400	0.74484500	-3.30001400
H	2.63730200	0.51458800	-4.13930400
H	2.54952100	1.25917000	-2.53314600
H	1.59395600	-0.19526500	-2.88649400
C	0.12465800	0.96875000	-4.97619800
H	-0.19813100	-0.04581100	-4.72249300
H	-0.75212700	1.55204900	-5.27092800
H	0.80925400	0.91070300	-5.82652200
C	1.28830000	3.02793000	-4.13518000
H	1.73619100	3.51251800	-3.26537300
H	2.03694700	2.98178600	-4.93204100
H	0.44545100	3.63122200	-4.48655900
N	-3.67700200	0.03016900	0.05950300
O	-3.68473600	0.45331200	-1.09000600
O	-2.81029200	0.29473300	0.86912500
C	-4.76197600	-0.84668600	0.46228000
C	-4.92032700	-1.25847000	1.79181400
C	-5.62625100	-1.29638300	-0.52506300
C	-5.90943400	-2.17712600	2.12066500
C	-6.61339800	-2.19528100	-0.16629900
C	-6.75600300	-2.66286000	1.13731600
H	-7.52927900	-3.38494700	1.37056000
N	-7.52535500	-2.67703100	-1.20323300
O	-8.32393600	-3.54403100	-0.89713800
O	-7.43477100	-2.18411000	-2.31344400
H	-5.99526100	-2.49167000	3.15375600
H	-5.51296900	-0.96001700	-1.54787200
F	-4.19542400	-0.74868000	2.76733900
O	-2.53734000	-2.45384200	1.60352400
O	-0.03652200	-0.50661700	-0.88273200
C	-1.43709500	-3.02957800	1.30454900
C	-0.90181500	-3.04322300	-0.00594100
C	-1.60378600	-2.28446800	-1.06543000
C	-2.76958500	-2.55013500	-1.70174300
H	-3.05987400	-1.83081800	-2.47192000
C	-3.73282600	-3.64271800	-1.53986200
C	-3.79683200	-4.46873000	-0.40645500
H	-3.11196100	-4.31061300	0.41263700
C	-4.78243600	-5.44533000	-0.30485700
H	-4.82421300	-6.06737200	0.58410600
C	-5.72261000	-5.61490300	-1.31992800
H	-6.49776000	-6.36946300	-1.22701800

C	-0.67509200	-3.73390000	2.32385100
H	-1.10351600	-3.74605500	3.32163700
C	0.51992100	-4.32898700	2.05847700
H	1.06159200	-4.85461500	2.84437200
C	1.09842800	-4.27750700	0.75352700
C	0.37362500	-3.61368600	-0.28356500
C	-5.67326500	-4.79850000	-2.44950400
H	-6.41557700	-4.90613400	-3.23457700
C	-4.69420300	-3.81719900	-2.55094000
H	-4.67395000	-3.16480400	-3.42092900
C	-0.97820900	-1.00925900	-1.47113100
H	-1.42016400	-0.49110600	-2.34570400
C	0.98053000	-3.56804400	-1.57427400
H	0.44756900	-3.09105400	-2.39236000
C	2.21311500	-4.13240800	-1.81038300
H	2.64520200	-4.07710500	-2.80625700
C	2.91525900	-4.79748100	-0.77885600
H	3.87624000	-5.26055000	-0.97913300
C	2.35482700	-4.86197500	0.47676300
H	2.87449400	-5.37392100	1.28421800

I-TS1-*S*

P	-3.14595800	-0.17969900	-1.75831700
N	-0.12588600	0.95949500	-1.66928200
C	0.28315400	2.25690700	-1.70680600
O	-0.03232800	3.03550400	-2.59895800
C	1.30530700	2.66742200	-0.64678900
N	1.09919400	2.10107100	0.66249200
C	-1.63119200	-0.77036800	-2.57066100
C	-0.61067300	0.32357200	-2.88567000
C	-3.59910100	1.44833300	-2.39030400
C	-0.05610600	2.16176500	1.36326900
C	-4.42465000	1.55619900	-3.51662000
C	-3.04463400	2.59374200	-1.80560200
O	0.17288800	1.76982600	2.62697100
O	-1.13644700	2.51827800	0.90858900
C	-4.15349600	-2.66539300	-2.47540600
C	-4.47173300	-1.35072200	-2.12368500
C	-5.80998900	-0.97348200	-1.94729900
C	-5.17251200	-3.59683900	-2.66349900
C	-3.34210900	3.84444200	-2.34044700
C	-4.17637800	3.95401100	-3.44995600
C	-6.50237900	-3.21747000	-2.50583500
C	-6.82051400	-1.90666200	-2.14863900
C	-4.71335700	2.81142600	-4.04053400
C	0.51847500	-0.31912000	-3.72783900
C	1.69513300	0.61510500	-3.96717700
C	1.57850400	4.18053400	-0.62714700
C	0.43343400	5.02075600	-0.09492800
C	-2.91037800	-0.13739000	0.04874400
H	2.23345400	2.19903200	-1.00403700
H	1.92890100	1.79840700	1.15541000
H	-1.18167400	-1.53874300	-1.92800500
H	-1.93666800	-1.25412900	-3.50452500
H	-4.84812700	0.66815200	-3.97659400
H	-2.38082900	2.52214300	-0.94609000
H	-3.11974500	-2.97779300	-2.58645000
H	-4.92076900	-4.61753000	-2.93371300
H	-2.91506900	4.73324600	-1.88776800
H	-4.40434600	4.93260300	-3.86075500
H	-7.29416900	-3.94425000	-2.65766900
H	-5.35846100	2.89508700	-4.90886900
H	1.36582800	1.56219600	-4.40284600
H	2.40769300	0.13740500	-4.64410600
H	2.21354400	0.82615400	-3.02583000
H	1.78914500	4.47578800	-1.66452600
H	-0.47822600	4.83517300	-0.66727900
H	0.69600800	6.07969000	-0.17022400
H	0.24867600	4.78715900	0.95713800
H	-2.41683300	-1.08623800	0.29178700
H	-2.20038700	0.66702400	0.27554100
H	0.24095500	0.36759800	-0.92547600
H	-1.06973700	1.11575200	-3.48480100
H	-6.06175700	0.04161200	-1.65072400
H	-7.85677200	-1.61008000	-2.02303900
C	-4.23146300	0.01138700	0.75489400
C	-4.79563000	1.27525100	0.93307100
C	-4.91370600	-1.12469100	1.18664700
C	-6.04515800	1.41996300	1.53910100
H	-4.23295600	2.14417300	0.60764200
C	-6.16633700	-1.01900400	1.79555800
H	-4.45203900	-2.09383500	1.02211600
C	-6.70949200	0.26064100	1.95508900
H	-7.68444700	0.36025700	2.42638100
C	-6.69657700	2.79003400	1.74857300
C	-8.03949000	2.82985900	1.00131400
H	-7.89358800	2.66017400	-0.07082800
H	-8.73170100	2.06861800	1.37356500

H	-8.51561600	3.80788600	1.13106300	C	5.25424800	5.91600600	1.85253500
C	-6.94066800	3.01496300	3.24947500	H	5.61390800	6.74746800	1.23573800
H	-5.99858000	2.98161800	3.80639200	H	4.21577400	6.12946600	2.13154600
H	-7.40090700	3.99522700	3.41420900	H	5.85003100	5.90774300	2.77535900
H	-7.60892200	2.25631500	3.66842500	C	-0.87937000	1.77043100	3.63422800
C	-5.81614700	3.92810900	1.22309300	C	-2.02130700	0.85322600	3.21732200
H	-6.32074900	4.88585700	1.38382400	H	-2.64894000	0.62901700	4.08546600
H	-4.85231700	3.96781400	1.74221800	H	-2.64043700	1.32554700	2.45638100
H	-5.62317600	3.82801900	0.14924900	H	-1.62414900	-0.08670100	2.81892200
C	-6.94704300	-2.24400200	2.28178200	C	-0.16515200	1.21687600	4.86061500
C	-6.20468000	-3.55024100	1.98412100	H	0.16276800	0.18817500	4.68074100
H	-6.02779800	-3.67899400	0.91016600	H	0.71017700	1.82566800	5.10352700
H	-5.24079500	-3.59106800	2.50230400	H	-0.84220000	1.21898000	5.71870200
H	-6.80342200	-4.39921800	2.32862600	C	-1.35034200	3.19969600	3.87585200
C	-7.16035700	-2.13916600	3.80057500	H	-1.82104400	3.60863500	2.97973800
H	-7.73328200	-1.24606800	4.06804900	H	-2.08137000	3.20917100	4.68996300
H	-7.71058000	-3.01313000	4.16624200	H	-0.50670500	3.83616800	4.15978700
H	-6.20088100	-2.09387700	4.32596000	N	3.55885800	-0.11648800	-0.31850200
C	-8.31219800	-2.29399000	1.57754500	O	3.67366300	0.62872000	0.65185500
H	-8.88185000	-3.16428700	1.92091100	O	2.48748300	-0.29344800	-0.88536100
H	-8.90827900	-1.39998800	1.78433300	C	4.72298200	-0.79541400	-0.79951400
H	-8.18487000	-2.37530800	0.49279900	C	4.60905800	-1.77295800	-1.81977600
O	2.71265800	4.38883700	0.20374700	C	5.91229900	-0.62505200	-0.10315000
O	0.92560100	-1.55412200	-3.17974000	C	5.77390500	-2.46029100	-2.22259000
H	1.51687400	-1.39880700	-2.42689700	C	7.01173600	-1.36477100	-0.48501500
H	0.06190500	-0.56778000	-4.69520100	C	6.95296000	-2.28482200	-1.54671600
Si	4.26395200	4.57740100	-0.40539400	H	7.84417000	-2.83246100	-1.82841200
C	4.69160700	3.16627800	-1.57137200	N	8.25742100	-1.18087300	0.23228400
H	4.75119300	2.21062900	-1.03869600	O	9.21458000	-1.86395200	-0.09961200
H	3.94955900	3.06784100	-2.37271700	O	8.29154700	-0.35291800	1.13072000
H	5.66057000	3.34141900	-2.05155700	H	5.68886600	-3.14961400	-3.05397900
C	4.35949200	6.19882600	-1.34595000	H	5.96490500	0.06734800	0.72743500
H	3.96832800	7.02734800	-0.74686700	F	3.61664300	-1.73250700	-2.70535400
H	5.39194800	6.44032000	-1.62275600	O	3.62583500	-3.13173300	-0.59654700
H	3.77781800	6.15399400	-2.27326300	O	-0.04668600	-0.72495000	0.75335200
C	5.37950400	4.57217100	1.12274600	C	2.39434500	-3.48071000	-0.76399600
C	6.83742500	4.35131300	0.69825900	C	1.38667100	-3.18376100	0.18786800
H	7.18156500	5.11221600	-0.01302400	C	1.71685400	-2.26476800	1.29708000
H	7.50075200	4.39748100	1.57235100	C	2.76690700	-2.30681300	2.15833600
H	6.97835900	3.36863300	0.23343500	H	2.88075600	-1.43384600	2.80453100
C	4.94959200	3.43863700	2.06330500	C	3.77997000	-3.33279200	2.39916900
H	3.91845800	3.57111700	2.40727200	C	3.68407200	-4.65088100	1.92534800
H	5.01381300	2.46006500	1.57381300	H	2.82617800	-4.94348600	1.33103200
H	5.60179100	3.41043000	2.94707200	C	4.67237500	-5.57778300	2.22542600

H	4.58202100	-6.59529700	1.85792500
C	5.78436300	-5.20659100	2.98352300
H	6.56071600	-5.93351900	3.20212400
C	2.00325000	-4.23090000	-1.93367200
H	2.77689100	-4.42176900	-2.67211000
C	0.73257900	-4.68672600	-2.11275500
H	0.47000900	-5.25210000	-3.00429600
C	-0.27453600	-4.45762900	-1.13125900
C	0.06458100	-3.70016600	0.03133800
C	5.89389100	-3.90230700	3.45847900
H	6.75455700	-3.60598100	4.04982900
C	4.89204800	-2.97929300	3.17842800
H	4.97078000	-1.96255300	3.55587100
C	0.91757100	-1.03141300	1.43645700
H	1.24833700	-0.34821400	2.24287500
C	-0.94855600	-3.53974000	1.02260100
H	-0.71012100	-3.01107800	1.94038800
C	-2.20802800	-4.06558700	0.85677900
H	-2.95002000	-3.93951800	1.64092600
C	-2.54165500	-4.79204200	-0.30994100
H	-3.53889000	-5.20495500	-0.42762000
C	-1.58230900	-4.98358100	-1.27674000
H	-1.81033600	-5.55648800	-2.17366300

I-IM2-*S*

P	-3.08532800	-0.05119600	-1.78509400
N	-0.07556700	1.04946900	-1.57566800
C	0.34325600	2.34701900	-1.57323400
O	0.02997800	3.15535800	-2.43860300
C	1.36339900	2.70737900	-0.49567600
N	1.12594000	2.10919400	0.79393200
C	-1.56140000	-0.63551800	-2.58440400
C	-0.51585700	0.45240900	-2.82823000
C	-3.51576000	1.59334500	-2.38612100
C	-0.04140500	2.16001600	1.47091100
C	-4.30607300	1.73516700	-3.53395400
C	-2.97304600	2.71971500	-1.75563300
O	0.14204900	1.67795300	2.71261000
O	-1.10031900	2.58093700	1.01868100
C	-4.08227500	-2.52533200	-2.54680800
C	-4.40683000	-1.21058900	-2.19983200
C	-5.74803500	-0.82967100	-2.06196900
C	-5.09763500	-3.45309200	-2.76823400
C	-3.24928300	3.98584800	-2.26528800
C	-4.04961100	4.12905000	-3.39542500
C	-6.43065000	-3.06993400	-2.64771900
C	-6.75496900	-1.75946000	-2.29576300
C	-4.57389500	3.00528500	-4.03199100
C	0.64487000	-0.18376400	-3.62999500
C	1.84227100	0.74112300	-3.78874500
C	1.66649500	4.21358500	-0.44224100
C	0.55683700	5.05843100	0.15416500
C	-2.89202700	-0.05553900	0.02825500
H	2.28569200	2.22413500	-0.84816400
H	1.93380500	1.69903500	1.24691000
H	-1.13447400	-1.43127800	-1.96114200
H	-1.85336600	-1.07926600	-3.54264700
H	-4.71848300	0.86211200	-4.03122700
H	-2.33283100	2.62283400	-0.88060200
H	-3.04610700	-2.83822900	-2.63342200
H	-4.84199900	-4.47299900	-3.03775700
H	-2.82891300	4.85945500	-1.77821500
H	-4.26035900	5.11928200	-3.78704500
H	-7.22016500	-3.79343900	-2.82498700
H	-5.19111700	3.11578300	-4.91750000
H	1.54766000	1.71253200	-4.19500800
H	2.57525700	0.28045800	-4.45566400
H	2.32563000	0.89861500	-2.81771000
H	1.83960900	4.53289300	-1.47957400
H	-0.38017600	4.90393700	-0.38550300
H	0.83566800	6.11438700	0.09738600
H	0.40801000	4.79970500	1.20621600
H	-2.40508300	-1.01048100	0.25703600
H	-2.18602100	0.74096300	0.29325900
H	0.34087000	0.43404000	-0.87787600
H	-0.94040700	1.26531700	-3.42460200
H	-6.00504400	0.18467100	-1.76730100
H	-7.79365500	-1.46041900	-2.19935500
C	-4.23057100	0.07195800	0.70524700
C	-4.81108600	1.32786000	0.88657900
C	-4.91406100	-1.07608400	1.10248400
C	-6.07815900	1.45296600	1.45954500
H	-4.24931600	2.20646800	0.58707600
C	-6.18431600	-0.99019200	1.67735800
H	-4.44014300	-2.03948900	0.93734200
C	-6.74339800	0.28215600	1.84030000
H	-7.73163500	0.36697700	2.28633800
C	-6.74676500	2.81445900	1.67052300

C	-8.07708400	2.84952800	0.90068500
H	-7.91191300	2.69117700	-0.17040400
H	-8.76920300	2.07932500	1.25416200
H	-8.56334200	3.82236600	1.03127400
C	-7.01855900	3.02095800	3.16934300
H	-6.08543200	2.99348300	3.74135800
H	-7.49319400	3.99396100	3.33607200
H	-7.68413000	2.24973600	3.56928200
C	-5.86850300	3.96659900	1.17249600
H	-6.38735300	4.91711700	1.33070700
H	-4.91621400	4.01328300	1.71191900
H	-5.65218400	3.87778800	0.10210000
C	-6.96934600	-2.22854400	2.12105700
C	-6.20527500	-3.52439100	1.83255300
H	-5.99307900	-3.63781600	0.76322300
H	-5.25837200	-3.56242000	2.38147900
H	-6.80686300	-4.38294000	2.14697000
C	-7.23491000	-2.14500300	3.63291500
H	-7.83180400	-1.26491300	3.89076000
H	-7.78206400	-3.03197100	3.97069600
H	-6.29437300	-2.08931700	4.19045400
C	-8.30835700	-2.28070400	1.36846300
H	-8.88538100	-3.15703700	1.68292500
H	-8.91575400	-1.39146900	1.56142400
H	-8.14149100	-2.35056300	0.28829300
O	2.83613400	4.38794900	0.34536100
O	1.01886100	-1.42283300	-3.06842000
H	1.68592200	-1.27010000	-2.37997900
H	0.23296600	-0.41260700	-4.62255900
Si	4.36038400	4.53581600	-0.34275900
C	4.69382200	3.12260400	-1.53618200
H	4.71632900	2.15658400	-1.01960000
H	3.92838900	3.07568700	-2.32063300
H	5.65835600	3.26207800	-2.03683200
C	4.44600600	6.15864100	-1.28553400
H	4.09304700	6.99431200	-0.67269400
H	5.47208800	6.38096300	-1.59989500
H	3.83134800	6.12597200	-2.19179600
C	5.55324900	4.51306100	1.12477400
C	6.98525200	4.27783200	0.62511200
H	7.29884500	5.03373500	-0.10572900
H	7.69325800	4.32204000	1.46379000
H	7.09639500	3.29222800	0.15864400
C	5.16109400	3.38096200	2.08242500
H	4.15429000	3.52846100	2.48706000
H	5.17483200	2.40563800	1.58336500
H	5.86460500	3.33602600	2.92537800
C	5.48283300	5.85564900	1.86407200
H	5.82030600	6.68546700	1.23261400
H	4.46324200	6.08046100	2.19856200
H	6.12624000	5.83621700	2.75430500
C	-0.92302400	1.69034800	3.70459200
C	-2.12419600	0.88702200	3.22219900
H	-2.78011800	0.66017300	4.06870000
H	-2.69459700	1.44667900	2.48278800
H	-1.79221300	-0.05572400	2.77379100
C	-0.26931400	1.01256700	4.90228100
H	-0.01687500	-0.02557200	4.66379000
H	0.64566300	1.53901000	5.18732100
H	-0.95524900	1.01505200	5.75335800
C	-1.29371000	3.13241000	4.03009600
H	-1.71499600	3.63056600	3.15467100
H	-2.03870500	3.14588000	4.83138500
H	-0.41196600	3.68546500	4.36772200
N	3.53678100	-0.16871300	-0.16260200
O	3.67465200	0.72626200	0.69005200
O	2.42251900	-0.38891900	-0.67595500
C	4.62596300	-0.93049300	-0.55935100
C	4.35867600	-2.19374900	-1.31071600
C	5.89574900	-0.54723100	-0.13342400
C	5.62236900	-2.90870200	-1.65415500
C	6.98976100	-1.29716700	-0.48458500
C	6.83988500	-2.49695900	-1.26250600
H	7.72743500	-3.06153000	-1.52217900
N	8.28779200	-0.87364400	-0.07506900
O	9.25202700	-1.56892100	-0.38960800
O	8.40030500	0.16420100	0.57563900
H	5.48708900	-3.82131600	-2.22578600
H	6.00944600	0.35154600	0.46099500
F	3.65468300	-1.95768800	-2.48937500
O	3.56046600	-3.06698600	-0.50391100
O	-0.10178600	-0.75162600	0.84059000
C	2.27308600	-3.43477200	-0.78349700
C	1.30574100	-3.19561100	0.17934800
C	1.62729400	-2.33233200	1.34348200
C	2.63819900	-2.49720300	2.22438800
H	2.81125900	-1.67798800	2.92484300
C	3.56577000	-3.62442600	2.35520900

C	3.20008300	-4.94431300	2.05779700
H	2.18429400	-5.16357600	1.74380200
C	4.12773100	-5.97137000	2.18293500
H	3.83418700	-6.99172400	1.95668700
C	5.43077500	-5.69560800	2.59728000
H	6.15493400	-6.49950500	2.68653200
C	1.97778800	-4.16734100	-1.95923700
H	2.76497600	-4.32007200	-2.68856800
C	0.71804000	-4.65935700	-2.16161400
H	0.48645900	-5.21817300	-3.06401600
C	-0.30139900	-4.47174000	-1.19094300
C	-0.00554100	-3.73732400	-0.00633400
C	5.79982300	-4.38818300	2.90549700
H	6.81156300	-4.16771200	3.23093200
C	4.86777200	-3.36165500	2.79936600
H	5.15031700	-2.34039800	3.04196300
C	0.87414800	-1.07112800	1.49605300
H	1.25390700	-0.39746200	2.28760000
C	-1.02843900	-3.60096200	0.97456700
H	-0.81147600	-3.07249000	1.89735000
C	-2.27477300	-4.14187900	0.77700700
H	-3.03636300	-4.03700700	1.54445000
C	-2.57143800	-4.85506200	-0.40981400
H	-3.56059900	-5.28057100	-0.54938200
C	-1.60220700	-5.01756000	-1.36734100
H	-1.81000700	-5.57595400	-2.27710800

I-TS2-*S*

P	-2.98760200	-0.21541700	-1.83821600
N	0.00816500	0.87470800	-1.43238200
C	0.40482900	2.17549100	-1.43944400
O	0.12893400	2.96295300	-2.33696800
C	1.35242200	2.58968200	-0.31301600
N	1.09351900	1.96941600	0.96382900
C	-1.40893700	-0.84356200	-2.47890000
C	-0.33056900	0.22267500	-2.69064900
C	-3.34301900	1.41213300	-2.52844000
C	-0.10078600	1.97949200	1.59781100
C	-4.02762100	1.52557600	-3.74534100
C	-2.84086000	2.55155200	-1.89005500
O	0.05507100	1.50701500	2.84811500
O	-1.15605600	2.36033000	1.10539500
C	-3.96170900	-2.70662200	-2.56095300
C	-4.29166100	-1.36925000	-2.31941600
C	-5.63098300	-0.95852700	-2.34295500
C	-4.96889500	-3.62679000	-2.84070600
C	-3.05187300	3.80389100	-2.46168400
C	-3.74945600	3.91938000	-3.66046900
C	-6.29821200	-3.21486800	-2.88434400
C	-6.62878300	-1.88242900	-2.63618900
C	-4.23264600	2.78155700	-4.30506500
C	0.90571600	-0.48736200	-3.28444800
C	2.09814500	0.45006500	-3.43786800
C	1.56485900	4.11107000	-0.24892100
C	0.42366900	4.89123900	0.37396900
C	-2.94431600	-0.15268600	-0.01665600
H	2.31810400	2.17015500	-0.62833800
H	1.90339200	1.64171400	1.47408800
H	-1.03261000	-1.59926100	-1.77804200
H	-1.62330800	-1.33623400	-3.43363300
H	-4.40262900	0.64075100	-4.25131900
H	-2.27191500	2.47088700	-0.96588100
H	-2.92865400	-3.04099400	-2.52600200
H	-4.71028000	-4.66268500	-3.03456700
H	-2.65567300	4.68724000	-1.97176200
H	-3.90829500	4.89835200	-4.10171000
H	-7.08072200	-3.93242100	-3.11048700
H	-4.76592200	2.87051900	-5.24576100
H	1.84315000	1.33929000	-4.02231100
H	2.92032500	-0.08430700	-3.91785400
H	2.45459600	0.75609100	-2.44886400
H	1.69099800	4.44300200	-1.28834100
H	-0.51517600	4.69200700	-0.14812000
H	0.64809800	5.96022500	0.31686100
H	0.30776600	4.62208900	1.42775500
H	-2.59316600	-1.14699100	0.28201100
H	-2.16336900	0.56421300	0.26428600
H	0.42691600	0.26615400	-0.73019400
H	-0.68348200	1.00214600	-3.37160100
H	-5.89241000	0.07526400	-2.13239400
H	-7.66480800	-1.56151600	-2.66984300
C	-4.29290000	0.17645200	0.56554000
C	-4.64454100	1.50650000	0.79858700
C	-5.19881000	-0.84348300	0.85686700
C	-5.89765600	1.83583900	1.31713700
H	-3.90853700	2.27724500	0.59839700
C	-6.46250100	-0.55195200	1.37509700
H	-4.90295100	-1.86959000	0.66010800

C	-6.78577400	0.79124800	1.59506300
H	-7.76338100	1.03138100	2.00694000
C	-6.30550200	3.28345500	1.60450900
C	-7.59494100	3.61721400	0.83702700
H	-7.44551200	3.50986300	-0.24240000
H	-8.42425800	2.96673200	1.13065500
H	-7.89436500	4.65139200	1.03870000
C	-6.55227700	3.44261700	3.11340500
H	-5.64909700	3.19732400	3.68244200
H	-6.83295800	4.47579100	3.34570400
H	-7.35828400	2.78840600	3.46032900
C	-5.22153800	4.27892800	1.17904400
H	-5.56123800	5.29920400	1.38260200
H	-4.28732100	4.12343800	1.72891300
H	-5.00364100	4.20519600	0.10780600
C	-7.48835100	-1.64293100	1.69750200
C	-6.96563200	-3.04220600	1.35802500
H	-6.72909300	-3.13559700	0.29191200
H	-6.06857100	-3.29277300	1.93399900
H	-7.73224600	-3.78611400	1.59685000
C	-7.82560200	-1.59549000	3.19629800
H	-8.24393700	-0.62595900	3.48384200
H	-8.56365800	-2.36666700	3.44301400
H	-6.93102400	-1.77115300	3.80270300
C	-8.76551300	-1.39625000	0.87879700
H	-9.50872800	-2.17177400	1.09362700
H	-9.21621300	-0.42641000	1.10972300
H	-8.54610200	-1.41985100	-0.19400600
O	2.74550100	4.35233300	0.50961000
O	1.19080900	-1.56668900	-2.45132300
H	2.20524200	-1.71453000	-2.39776600
H	0.60327600	-0.82681900	-4.29566300
Si	4.18242100	4.70716500	-0.28477300
C	4.56316000	3.36128000	-1.53846500
H	4.68467300	2.38590900	-1.05581600
H	3.75702800	3.27265500	-2.27745700
H	5.48215100	3.58445900	-2.09141900
C	3.98574400	6.34318000	-1.18941200
H	3.62181200	7.12856200	-0.51896300
H	4.93529300	6.68030600	-1.61993200
H	3.27067900	6.25277200	-2.01455500
C	5.49577900	4.82180900	1.07133200
C	6.89289200	4.80183600	0.43568500
H	7.02797600	5.60707800	-0.29686500

H	7.66330900	4.93146300	1.20772200
H	7.09472800	3.85061000	-0.07042700
C	5.36523100	3.63457500	2.03379700
H	4.38434800	3.61552900	2.52007800
H	5.49573900	2.67651000	1.51943200
H	6.13267900	3.70009100	2.81764700
C	5.30804900	6.12899600	1.85272900
H	5.46431700	7.00827600	1.21773200
H	4.30423900	6.20129500	2.28727600
H	6.03123500	6.18590800	2.67786600
C	-1.03635900	1.52309500	3.81207100
C	-2.19679800	0.66568400	3.32462000
H	-2.89065800	0.48170200	4.15091200
H	-2.73703000	1.16565800	2.52267500
H	-1.82848600	-0.29756400	2.95592200
C	-0.39769000	0.90977500	5.05194100
H	-0.10439200	-0.12729800	4.85974600
H	0.48991000	1.47646500	5.34611300
H	-1.11041500	0.91860100	5.88064700
C	-1.45813400	2.96355400	4.07833200
H	-1.86850500	3.42093700	3.17635100
H	-2.22419000	2.98068200	4.85939700
H	-0.60196800	3.55312700	4.42040000
N	3.70443800	-0.10333200	0.20129000
O	3.95411600	0.92514400	0.83207500
O	2.57364400	-0.46235500	-0.07112100
C	4.81265700	-0.90063200	-0.26695300
C	4.60257400	-2.26501800	-0.57983600
C	6.01913500	-0.26470500	-0.49530600
C	5.71463400	-3.00361100	-1.03927400
C	7.05427700	-1.01233400	-1.02751800
C	6.91321300	-2.38092300	-1.29621600
H	7.76320300	-2.93882700	-1.67080600
N	8.32343600	-0.36042700	-1.28465400
O	9.23334100	-1.03627900	-1.74126600
O	8.42285300	0.83187900	-1.03382300
H	5.59554000	-4.07147100	-1.18108700
H	6.14666400	0.78672300	-0.26931700
F	3.60093800	-1.91329400	-2.35087600
O	3.60501000	-2.91144700	0.08035000
O	-0.23566200	-0.94561300	0.89645200
C	2.46589700	-3.46678500	-0.45123800
C	1.32537100	-3.34985000	0.31564100
C	1.36851300	-2.58438200	1.58783900

C	2.12207900	-2.87459300	2.67021800
H	2.10086800	-2.14229000	3.47966200
C	2.99926200	-4.02107700	2.92648000
C	2.86618500	-5.26304200	2.28562300
H	2.06907000	-5.41988300	1.56775100
C	3.73905300	-6.30187200	2.58389400
H	3.62134400	-7.25978700	2.08710300
C	4.75929800	-6.12119500	3.51799000
H	5.44086600	-6.93568600	3.74284000
C	2.49773800	-4.22824300	-1.64144400
H	3.41306300	-4.26118300	-2.21706200
C	1.35708700	-4.84545900	-2.07111900
H	1.36511800	-5.42769800	-2.98825400
C	0.14386900	-4.73856900	-1.33999400
C	0.12475400	-3.98240000	-0.13388500
C	4.89337800	-4.89766600	4.16975500
H	5.67831400	-4.75304900	4.90510400
C	4.01224700	-3.86123500	3.88325700
H	4.10961400	-2.90740700	4.39511000
C	0.59389700	-1.33434200	1.69758200
H	0.80849400	-0.73578200	2.60335700
C	-1.09218300	-3.91309500	0.60144900
H	-1.11947800	-3.35333400	1.53109400
C	-2.22405100	-4.55174000	0.15642600
H	-3.14192000	-4.49252900	0.73442700
C	-2.20013600	-5.30614400	-1.04161300
H	-3.09746600	-5.82078700	-1.37152900
C	-1.04093300	-5.39210200	-1.77147600
H	-1.00656200	-5.97184500	-2.69029600

I-IM3-*S*

P	-2.89984700	0.24035600	-1.96250200
N	0.10869000	1.10772800	-1.45174100
C	0.54510700	2.39216700	-1.32850000
O	0.26933300	3.27719700	-2.13005300
C	1.53627900	2.66720200	-0.19584400
N	1.25144100	1.98691500	1.04452100
C	-1.38479200	-0.38361500	-2.73944500
C	-0.22174200	0.60874200	-2.78290300
C	-3.20833300	1.96179800	-2.40580700
C	0.05519500	2.02731900	1.67279600
C	-3.88675200	2.25728300	-3.59555500
C	-2.71676200	2.99091700	-1.59619400
O	0.16670800	1.49718700	2.90449000
O	-0.97187300	2.48672300	1.18873100
C	-4.06338100	-2.02798200	-3.08064200
C	-4.29065300	-0.75385100	-2.55276700
C	-5.60063600	-0.28085400	-2.39693300
C	-5.14389200	-2.82270000	-3.45905200
C	-2.93814900	4.31526100	-1.96719400
C	-3.62666600	4.61024500	-3.14029000
C	-6.44446500	-2.34763300	-3.31430400
C	-6.67230500	-1.07702000	-2.78488800
C	-4.09307600	3.58264000	-3.95926300
C	0.99712600	-0.14070000	-3.36913600
C	2.22747500	0.76082500	-3.46156600
C	1.83582500	4.16660600	-0.03515200
C	0.72935600	4.97591600	0.61262500
C	-2.79460400	0.04123000	-0.15230200
H	2.47312600	2.21524800	-0.54927000
H	2.04068000	1.59285700	1.53803400
H	-1.06745100	-1.27994400	-2.19005000
H	-1.64473300	-0.67903900	-3.76178900
H	-4.26063000	1.45753500	-4.22873400
H	-2.15569800	2.77155800	-0.69047000
H	-3.05326800	-2.40958400	-3.19441100
H	-4.96538700	-3.80874300	-3.87583400
H	-2.56330700	5.11535400	-1.33731000
H	-3.79545700	5.64471500	-3.42275200
H	-7.28442200	-2.96588100	-3.61526600
H	-4.62254300	3.81232400	-4.87801600
H	2.02967100	1.66905500	-4.04037900
H	3.04695000	0.19852700	-3.91158700
H	2.55779900	1.03914500	-2.45470900
H	2.00431900	4.55171700	-1.04984500
H	-0.20779200	4.85329800	0.06568000
H	1.01202800	6.03273800	0.61481600
H	0.58249700	4.65876800	1.64911500
H	-2.36188700	-0.95642100	-0.01621700
H	-2.05758100	0.76323200	0.21919100
H	0.53290700	0.40728300	-0.84163500
H	-0.48326400	1.47521200	-3.39574700
H	-5.78140900	0.70303500	-1.97146300
H	-7.68544100	-0.70497400	-2.67216200
C	-4.14089600	0.15730300	0.50963800
C	-4.65394900	1.40706500	0.85805600
C	-4.89186100	-0.99342000	0.74469300
C	-5.91385400	1.52262200	1.44937500

H	-4.04285600	2.28578700	0.67884300	C	5.75487000	4.57917500	1.42092100
C	-6.15949000	-0.91649700	1.32542000	C	7.16826600	4.62038500	0.82458500
H	-4.47022200	-1.95014600	0.45099600	H	7.34053000	5.52609400	0.23017000
C	-6.64492600	0.34986500	1.66780100	H	7.92010700	4.60911400	1.62570900
H	-7.62873800	0.42701700	2.12509000	H	7.36213700	3.75431000	0.18116600
C	-6.49939600	2.87478200	1.86543300	C	5.57200100	3.26947100	2.19732000
C	-7.82889600	3.10215600	1.12802600	H	4.56491200	3.19189900	2.62032900
H	-7.67469800	3.11026000	0.04378900	H	5.73073700	2.39466900	1.55817600
H	-8.55961600	2.32126300	1.36001300	H	6.29484200	3.21431400	3.02334100
H	-8.26224400	4.06506700	1.41926100	C	5.56971300	5.76463500	2.37780600
C	-6.74650800	2.86957200	3.38265600	H	5.74754200	6.72342800	1.87771700
H	-5.80896600	2.70854200	3.92576200	H	4.55934600	5.78859800	2.80220400
H	-7.16650800	3.82978200	3.70156300	H	6.27982900	5.69214000	3.21299100
H	-7.44710800	2.08153000	3.67530800	C	-0.94044400	1.55537200	3.84996600
C	-5.55633600	4.03550500	1.53420400	C	-2.14458700	0.78763600	3.32084500
H	-6.01607300	4.97882500	1.84519100	H	-2.87737800	0.65568200	4.12345400
H	-4.59988500	3.94159100	2.05974500	H	-2.61929500	1.32631300	2.50283300
H	-5.35151500	4.10033600	0.46000200	H	-1.84194400	-0.20153200	2.96182400
C	-7.02120400	-2.15598300	1.58166700	C	-0.36786100	0.87280800	5.08558300
C	-6.32233000	-3.44150700	1.12980500	H	-0.13583800	-0.17609300	4.87518600
H	-6.09908000	-3.42195000	0.05686500	H	0.54651700	1.37546900	5.41239300
H	-5.38740800	-3.60495300	1.67685600	H	-1.09717200	0.90694000	5.89920300
H	-6.97422900	-4.29954300	1.32118400	C	-1.27856200	3.01154400	4.15154000
C	-7.32388800	-2.26761800	3.08427200	H	-1.65569500	3.51700100	3.26093500
H	-7.86133300	-1.38900300	3.45378000	H	-2.04689900	3.05318400	4.92933300
H	-7.94511500	-3.14821800	3.28067300	H	-0.39136000	3.53976000	4.51449000
H	-6.39894900	-2.36598600	3.66190000	N	3.74691500	-0.23798800	0.09450000
C	-8.33893900	-2.02521200	0.80132100	O	4.10229600	0.82366700	0.60406000
H	-8.96904900	-2.90457800	0.97416000	O	2.59089500	-0.53915100	-0.10264100
H	-8.90620900	-1.14025000	1.10565400	C	4.79650500	-1.17055400	-0.29318700
H	-8.14249300	-1.94919700	-0.27340800	C	4.54566300	-2.54996300	-0.32081000
O	3.01178900	4.28937300	0.75810800	C	6.01722600	-0.64262600	-0.65895700
O	1.21682300	-1.24979700	-2.56994300	C	5.56644200	-3.41167300	-0.71207200
H	2.28279800	-1.47573100	-2.53307200	C	6.99619500	-1.53076900	-1.07996500
H	0.70900200	-0.42048000	-4.40599200	C	6.79312000	-2.90496000	-1.11611200
Si	4.47468100	4.67671200	0.03188500	H	7.59289400	-3.56022100	-1.43885200
C	4.83941300	3.46722800	-1.35703700	N	8.29173600	-0.99158900	-1.49032100
H	4.96088500	2.44715000	-0.97795600	O	9.13921700	-1.77861100	-1.87545500
H	4.02460100	3.45598500	-2.09174200	O	8.45460800	0.21464400	-1.42405200
H	5.75259000	3.74533800	-1.89438000	H	5.38145800	-4.47955300	-0.68547200
C	4.35525400	6.41474600	-0.67226700	H	6.19754100	0.42490700	-0.63666400
H	3.99103200	7.12251400	0.07937800	F	3.53351300	-1.69140300	-2.51577400
H	5.32808700	6.77123100	-1.02885800	O	3.41836600	-3.04547800	0.24365700
H	3.66653200	6.45097400	-1.52347800	O	-0.17890800	-0.77080400	0.89461200

C	2.28659600	-3.42025600	-0.45467900
C	1.09724100	-3.28863600	0.22702500
C	1.09372600	-2.67247100	1.57835200
C	1.67297900	-3.18639700	2.68356500
H	1.67248400	-2.55446000	3.57331800
C	2.33992800	-4.48177100	2.85560400
C	2.02900200	-5.61135100	2.08216300
H	1.25207100	-5.55232300	1.32775700
C	2.69448200	-6.81212900	2.29611800
H	2.43870800	-7.68015700	1.69668000
C	3.67996200	-6.90738500	3.27836700
H	4.19911700	-7.84727800	3.43824300
C	2.36840400	-4.01351500	-1.73281700
H	3.31949500	-4.03061700	-2.24852000
C	1.22403400	-4.46693100	-2.32652500
H	1.26635300	-4.91820000	-3.31382700
C	-0.03597800	-4.34813000	-1.68289800
C	-0.10472400	-3.74124300	-0.39645900
C	3.98657200	-5.79704300	4.06208100
H	4.74556900	-5.86760500	4.83483400
C	3.31112200	-4.59845700	3.86033200
H	3.54340200	-3.73332100	4.47600800
C	0.47400300	-1.34375700	1.74482500
H	0.63051600	-0.87175400	2.73430100
C	-1.37309600	-3.64523700	0.24148300
H	-1.43946100	-3.19754900	1.22900000
C	-2.50754400	-4.12249600	-0.36999800
H	-3.46586300	-4.05452200	0.13798300
C	-2.43613900	-4.72685600	-1.64839600
H	-3.34056400	-5.10735900	-2.11335600
C	-1.22612500	-4.83198600	-2.28780800
H	-1.15461300	-5.29619200	-3.26820700

I-IM1-*R*

O	2.03281200	3.96869400	0.35791000
Si	3.19907800	5.17068600	0.26814900
C	2.80805300	6.37395900	-1.11999900
H	2.92497600	5.93497300	-2.11600400
H	1.78648100	6.75953100	-1.03902700
H	3.48497500	7.23333400	-1.05706000
C	3.15832900	6.04823400	1.91983300
H	3.20683100	5.33186700	2.74579600
H	3.98960200	6.75237800	2.03160500
H	2.22514000	6.61222500	2.02335300
C	4.87918600	4.34514900	-0.02958400
C	5.94397300	5.40875000	-0.32793500
H	6.03537700	6.13910200	0.48510900
H	6.92662400	4.93362600	-0.45277800
H	5.72278100	5.95771900	-1.25055600
C	4.77906100	3.38768400	-1.22345900
H	4.08613100	2.56376200	-1.00989700
H	4.43880500	3.89150800	-2.13651700
H	5.76334800	2.94738900	-1.43725000
C	5.28781800	3.54550600	1.21501100
H	5.41034600	4.19159300	2.09180500
H	4.54363600	2.77793600	1.46195700
H	6.24913300	3.04198500	1.04339600
P	-3.25279200	-0.80117100	-2.04868100
N	-0.21531500	0.27886400	-1.75804700
C	0.19187900	1.57082600	-1.85114800
O	0.00582100	2.26745800	-2.84241300
C	1.03697300	2.09906000	-0.69159800
N	0.79833500	1.47228500	0.58879300
C	-1.67698800	-1.50072700	-2.63897500
C	-0.59215700	-0.46459200	-2.94987600
C	-3.56525700	0.77169400	-2.87054400
C	-0.38171500	1.51084400	1.25663200
C	-4.21591100	0.79078300	-4.11087800
C	-3.06123200	1.95426500	-2.31568000
O	-0.17432300	1.18949100	2.53875600
O	-1.46244800	1.78289400	0.74713900
C	-4.26635300	-3.33660600	-2.49349700
C	-4.57216600	-1.97249300	-2.43067700
C	-5.90165400	-1.54372000	-2.53045200
C	-5.28675200	-4.26631600	-2.67136300
C	-3.23714200	3.15556600	-2.99735600
C	-3.90174800	3.17838000	-4.22041300
C	-6.60663500	-3.83857900	-2.79052200
C	-6.91342700	-2.48006600	-2.72057400
C	-4.38569100	1.99734200	-4.78036800
C	0.61132500	-1.20825700	-3.57397700
C	1.77046500	-0.28343300	-3.91234400
C	1.09468500	3.63637900	-0.65151000
C	-0.23150800	4.32532800	-0.37027500
C	-3.18815200	-0.61421700	-0.23989400
H	2.05255900	1.78219200	-0.96405700
H	1.62036900	1.26513000	1.14343400
H	-1.31237600	-2.19764000	-1.87571200

H	-1.90422500	-2.06803700	-3.54787300	C	-7.85188400	-1.47072400	1.59961800
H	-4.59321900	-0.12815300	-4.54978000	C	-7.51361200	-2.93640400	1.31209900
H	-2.51973200	1.94212700	-1.37176100	H	-7.30225500	-3.10192400	0.24970200
H	-3.24040100	-3.68135200	-2.39917500	H	-6.64720400	-3.27178600	1.89151800
H	-5.04755600	-5.32336600	-2.71928900	H	-8.36438000	-3.56785100	1.58660000
H	-2.84617200	4.07392900	-2.57156300	C	-8.16781700	-1.33008600	3.09745200
H	-4.03641300	4.11975000	-4.74365200	H	-8.46110800	-0.30788700	3.35481200
H	-7.39953600	-4.56550000	-2.93477900	H	-8.99327900	-1.99552400	3.37276700
H	-4.89577100	2.01420800	-5.73776300	H	-7.29678500	-1.59508400	3.70538200
H	1.44619900	0.53182400	-4.56387800	C	-9.09478900	-1.09020200	0.77849700
H	2.55707600	-0.85277700	-4.41436100	H	-9.93027700	-1.75343900	1.02765800
H	2.19251000	0.15301600	-3.00059100	H	-9.41131700	-0.06170300	0.97680200
H	1.45556700	3.94938900	-1.64005000	H	-8.89282400	-1.18158000	-0.29423500
H	-0.99529600	3.99091600	-1.07768600	O	1.01888000	-2.27675000	-2.73904900
H	-0.10682400	5.40759000	-0.47359300	H	0.24319700	-1.67120400	-4.49892200
H	-0.56996700	4.11182500	0.64562000	C	-1.25920700	1.04806700	3.50511900
H	-2.92485000	-1.61216700	0.12804500	C	-2.06750100	-0.19788900	3.17383700
H	-2.33992900	0.04327800	-0.02471400	H	-2.86176600	-0.32492100	3.91604700
H	0.04240500	-0.24823200	-0.92651500	H	-2.53201900	-0.10216900	2.19138300
H	-0.96054900	0.26889700	-3.67339900	H	-1.42822100	-1.08577400	3.19340300
H	-6.14702600	-0.48751500	-2.46181500	C	-0.50247100	0.87598000	4.81550100
H	-7.94214800	-2.14689900	-2.81102200	H	0.20580700	0.04617200	4.73639900
C	-4.47545700	-0.10483000	0.35628100	H	0.05039600	1.78721600	5.06164700
C	-4.63657000	1.26177000	0.59066100	H	-1.20468400	0.66038100	5.62573600
C	-5.50176700	-0.99362000	0.68064500	C	-2.13096700	2.29953900	3.54175400
C	-5.82529100	1.75992400	1.12686600	H	-2.80006700	2.34238900	2.68135600
H	-3.80737300	1.92402700	0.37464800	H	-2.73086800	2.28325500	4.45688500
C	-6.69957200	-0.53104400	1.23077600	H	-1.50655400	3.19853700	3.55783900
H	-5.34924200	-2.05315900	0.49800000	H	1.65234500	-1.94242400	-2.08489100
C	-6.83672600	0.84596800	1.43819400	N	3.53953900	-0.30695000	-0.22355200
H	-7.76395900	1.22038000	1.86530100	O	3.61547900	0.35270700	0.79869900
C	-6.03677700	3.25428600	1.38812800	O	2.47970700	-0.67944100	-0.70449200
C	-7.30990600	3.72649500	0.66713100	C	4.76831000	-0.56608700	-0.95773800
H	-7.22841500	3.56377900	-0.41271800	C	4.83650200	-1.54837400	-1.95011500
H	-8.20076400	3.20021600	1.02302200	C	5.87184400	0.21449800	-0.66257100
H	-7.46437000	4.79679900	0.84121900	C	6.00933900	-1.74783000	-2.66203500
C	-6.19194300	3.48338700	2.90002300	C	7.02560500	0.00897600	-1.40192200
H	-5.29195900	3.16246200	3.43518700	C	7.11940900	-0.96014300	-2.39358200
H	-6.35428200	4.54660500	3.10817700	H	8.04547100	-1.09017600	-2.93989600
H	-7.04300800	2.92535200	3.30343400	N	8.18458500	0.86189200	-1.13244200
C	-4.86137100	4.09717100	0.88144500	O	9.21619600	0.62327800	-1.73343900
H	-5.06816800	5.15676400	1.06175900	O	8.05010500	1.76748100	-0.32713200
H	-3.92575700	3.85009900	1.39474100	H	6.03176200	-2.52911900	-3.41285300
H	-4.70605600	3.96161300	-0.19482300	H	5.82157000	0.96187000	0.12024300

F	3.79554500	-2.31409500	-2.23271200
O	4.34935500	-2.60733900	0.66474000
O	-0.42147100	-1.68078700	0.40148000
C	3.92285500	-2.48544300	1.84974400
C	2.56026000	-2.69657000	2.22889900
C	1.54657800	-2.85221300	1.17237400
C	1.53198000	-3.66481200	0.08669100
H	0.74900000	-3.44864400	-0.64049900
C	2.39797300	-4.77857100	-0.28521900
C	2.27732100	-5.29036600	-1.58787800
H	1.57948100	-4.81332800	-2.27251900
C	3.04989400	-6.36684100	-2.00993700
H	2.94813100	-6.74267600	-3.02370500
C	3.94964200	-6.96334600	-1.12886300
H	4.55214500	-7.80751000	-1.45083900
C	4.82609900	-2.06375200	2.91332100
H	5.85450800	-1.87289300	2.61623900
C	4.42444200	-1.90021700	4.20158200
H	5.12966700	-1.57297700	4.96344600
C	3.07251600	-2.15250900	4.59181000
C	2.14448200	-2.56283800	3.58615000
C	4.07571200	-6.46848600	0.16985300
H	4.77741700	-6.92791700	0.85956800
C	3.31942500	-5.38074100	0.58685700
H	3.42641500	-4.99790200	1.59509800
C	0.51740000	-1.78520500	1.18361000
H	0.66772800	-1.01070400	1.95748100
C	0.81377300	-2.85262700	4.01055600
H	0.09991600	-3.23695400	3.28579400
C	0.42585300	-2.70196100	5.32397900
H	-0.59736000	-2.93254800	5.60931700
C	1.34491500	-2.26680800	6.30189800
H	1.03192600	-2.15231900	7.33473800
C	2.64692900	-2.00801500	5.92905500
H	3.37637200	-1.68600300	6.66950400

I-TS1-*R*

O	2.10681600	4.25919200	0.24038100
Si	3.17675300	5.49093300	-0.13551900
C	2.89752200	6.05941600	-1.90358600
H	2.91980600	5.23010400	-2.61797600
H	1.93446800	6.56987100	-2.01343900
H	3.67664000	6.77200200	-2.19692600
C	2.91905900	6.93635700	1.03161100
H	3.10715400	6.65570500	2.07285600
H	3.58051300	7.77281100	0.78115800
H	1.88824800	7.30127000	0.96788000
C	4.89892200	4.73246400	0.08306700
C	5.97755300	5.81601800	-0.04173200
H	5.87490100	6.58082700	0.73654700
H	6.97635400	5.37144600	0.06383300
H	5.94462800	6.31970100	-1.01558300
C	5.12148200	3.66232000	-0.99339700
H	4.36020400	2.87443900	-0.92668200
H	5.09188100	4.08315000	-2.00527400
H	6.10485700	3.18901700	-0.86038700
C	4.99556900	4.07082200	1.46453600
H	4.86780200	4.79837300	2.27467300
H	4.23654100	3.29118200	1.58588700
H	5.98368800	3.60647400	1.59358700
P	-3.35160000	-0.78874100	-1.70573600
N	-0.31721600	0.55045300	-1.49363900
C	-0.22210200	1.90268000	-1.47325700
O	-0.82472400	2.63690200	-2.25057300
C	0.81678100	2.43856200	-0.48457000
N	0.79141400	1.76933700	0.79327300
C	-1.70630100	-1.32733400	-2.24366900
C	-0.77553900	-0.18042200	-2.66034800
C	-4.15002700	0.12047100	-3.04836500
C	-0.31480100	1.64802000	1.56717500
C	-4.98706500	-0.53793200	-3.95622800
C	-3.85365000	1.47930400	-3.21980800
O	0.02045000	1.06960100	2.73247700
O	-1.43516100	2.00781100	1.24098700
C	-3.70124200	-3.45538500	-0.98932900
C	-4.33350800	-2.24682800	-1.29797200
C	-5.72806400	-2.13798800	-1.20539900
C	-4.46366100	-4.55412600	-0.60065100
C	-4.41698200	2.17657600	-4.28450900
C	-5.26712800	1.52519900	-5.17555900
C	-5.84991600	-4.44964300	-0.52390100
C	-6.48131300	-3.24332700	-0.82683700
C	-5.54544000	0.16948000	-5.01607800
C	0.41371800	-0.78094100	-3.44340400
C	1.43372000	0.27488800	-3.83522900
C	0.88365300	3.96918600	-0.41959400
C	-0.27146700	4.64673200	0.29536400
C	-3.19447400	0.26893800	-0.23065200

H	1.77470100	2.13934700	-0.93381800
H	1.68334700	1.50793400	1.19307400
H	-1.24454600	-1.86358200	-1.40810100
H	-1.85859200	-2.02779700	-3.07264500
H	-5.20778300	-1.59422900	-3.83636900
H	-3.16562400	1.99057100	-2.55036600
H	-2.62138700	-3.55152700	-1.04397200
H	-3.96996700	-5.49023500	-0.36235000
H	-4.18686400	3.22869000	-4.41568300
H	-5.70967900	2.07370700	-6.00103900
H	-6.44134700	-5.30992400	-0.22690800
H	-6.19958900	-0.33998600	-5.71590700
H	0.95148100	1.09057400	-4.38199300
H	2.20486800	-0.16767000	-4.46981600
H	1.91872600	0.69096000	-2.94663800
H	0.91773100	4.31639700	-1.46087400
H	-1.22539600	4.35987800	-0.15223200
H	-0.15739400	5.73337200	0.22275100
H	-0.27849200	4.36996100	1.35276000
H	-2.46919900	-0.23910500	0.41706900
H	-2.72106900	1.20406200	-0.54094400
H	0.23175000	0.03643700	-0.81090400
H	-1.28871000	0.53594500	-3.30983800
H	-6.22250400	-1.19492300	-1.42438700
H	-7.56158000	-3.16078000	-0.76674900
C	-4.52307500	0.48820500	0.44222700
C	-5.33695300	1.55544400	0.06207100
C	-4.96033400	-0.41062100	1.41642900
C	-6.59474300	1.73599700	0.64404800
H	-4.96985600	2.24742500	-0.68900100
C	-6.20533600	-0.25517900	2.02739600
H	-4.31020000	-1.23806900	1.68498100
C	-7.00123000	0.82143700	1.62089000
H	-7.97501400	0.95516900	2.08623000
C	-7.53035500	2.88132500	0.24660400
C	-8.83621500	2.29230000	-0.31243900
H	-8.63718300	1.67762300	-1.19697000
H	-9.34622400	1.66745900	0.42730900
H	-9.52018700	3.09745200	-0.60232700
C	-7.84577700	3.73665600	1.48451000
H	-6.92935300	4.15931900	1.90866700
H	-8.51090600	4.56335500	1.21236900
H	-8.34109000	3.15085500	2.26473000
C	-6.91147800	3.78567300	-0.82342600
H	-7.61426700	4.58587200	-1.07582700
H	-5.98587200	4.25270400	-0.47128800
H	-6.69053800	3.23284300	-1.74300700
C	-6.71277100	-1.21117800	3.11049500
C	-5.72968600	-2.35623200	3.37121600
H	-5.55809600	-2.95198600	2.46767100
H	-4.76246900	-1.98775400	3.72886600
H	-6.13762000	-3.02139400	4.13867300
C	-6.91290600	-0.42770500	4.41780300
H	-7.64620900	0.37550500	4.29553800
H	-7.27219800	-1.09589000	5.20817900
H	-5.97102900	0.02182800	4.74960800
C	-8.05291500	-1.82020900	2.66902200
H	-8.42851600	-2.50350000	3.43835200
H	-8.81415900	-1.05248500	2.50205400
H	-7.93023300	-2.38617100	1.73948200
O	0.97632800	-1.85257200	-2.71291700
H	-0.01286200	-1.21843100	-4.35683200
C	-0.94875200	0.93379500	3.80961200
C	-2.13534300	0.08558100	3.36550900
H	-2.73675100	-0.17823500	4.24190100
H	-2.76118800	0.63166400	2.65976100
H	-1.77662800	-0.83508000	2.89327700
C	-0.14784700	0.21214200	4.88495400
H	0.19104700	-0.75955500	4.51317500
H	0.73237500	0.79664600	5.16703500
H	-0.76627200	0.05462600	5.77295700
C	-1.38074300	2.31746100	4.28177700
H	-1.93563000	2.83964600	3.50002700
H	-2.02490900	2.21904700	5.16070800
H	-0.50544000	2.91222800	4.56014400
H	1.76477400	-1.56736000	-2.22414200
N	3.53669400	0.18025700	-0.26778800
O	3.56195800	0.93558500	0.69850300
O	2.49320500	-0.25321700	-0.74709900
C	4.78272300	-0.18664300	-0.87401400
C	4.82716500	-1.14680100	-1.91036100
C	5.94881100	0.33235600	-0.31960300
C	6.09030100	-1.52534900	-2.41711600
C	7.15855400	-0.05651600	-0.84932000
C	7.24207600	-1.00180200	-1.88918200
H	8.21293300	-1.28433000	-2.27863700
N	8.37151700	0.53396600	-0.31734600
O	9.44030600	0.12138900	-0.74106200

O	8.26492600	1.41724500	0.52050800
H	6.10700200	-2.23092700	-3.23913900
H	5.89142600	1.03384700	0.50376300
F	3.79515600	-1.32909800	-2.73127800
O	4.05783700	-2.85966400	-0.86859200
O	-0.27458200	-1.35836800	0.79035100
C	4.17213800	-2.78833000	0.41094000
C	3.05782700	-2.79665000	1.28820200
C	1.69756400	-2.74457600	0.72257600
C	1.15350000	-3.50620900	-0.25475200
H	0.17741000	-3.17013200	-0.59736200
C	1.62250200	-4.70885400	-0.93866300
C	0.91088800	-5.10428900	-2.08155100
H	0.09351500	-4.47715600	-2.42767700
C	1.25427600	-6.25493100	-2.78133000
H	0.69676500	-6.53691000	-3.66929600
C	2.31322000	-7.04308600	-2.33727800
H	2.58542300	-7.94541500	-2.87646100
C	5.48084700	-2.65313200	1.01339500
H	6.33024900	-2.64656600	0.33485000
C	5.65987200	-2.53626800	2.35876700
H	6.65830800	-2.42113200	2.77532500
C	4.55111500	-2.58256700	3.25243200
C	3.24272300	-2.73779600	2.70515900
C	3.02502500	-6.66729300	-1.19703100
H	3.85057700	-7.28016700	-0.84816400
C	2.69281000	-5.50815300	-0.50688400
H	3.25167100	-5.22355800	0.37742100
C	0.87265000	-1.58337800	1.15571800
H	1.37034700	-0.87597800	1.84478900
C	2.15979800	-2.85202700	3.62465200
H	1.15787300	-3.03838700	3.24559800
C	2.35944100	-2.76612800	4.98299700
H	1.51198500	-2.85817500	5.65671400
C	3.65517300	-2.57693800	5.51185600
H	3.80157500	-2.51086100	6.58515800
C	4.72637700	-2.49812600	4.65333400
H	5.73508500	-2.37281600	5.04023100

I-IM2-*R*

O	1.98565100	4.32337400	1.10469400
Si	3.05486500	5.58122300	0.81930200
C	2.28595100	6.82689400	-0.36060900
H	2.22396800	6.46060800	-1.39049300
H	1.27561100	7.10723400	-0.04324800
H	2.88762100	7.74255700	-0.37246600
C	3.37718100	6.38514600	2.48035300
H	3.64973700	5.63636400	3.23083200
H	4.18350000	7.12482700	2.43174300
H	2.47595200	6.89752300	2.83434700
C	4.66033800	4.88244700	0.08602300
C	5.50970500	6.03257400	-0.47291900
H	5.72759300	6.79325500	0.28727500
H	6.47367300	5.65074300	-0.83532400
H	5.01608100	6.53203000	-1.31443000
C	4.33431000	3.89461400	-1.04146900
H	3.81706500	3.01497700	-0.64367300
H	3.70466300	4.34302900	-1.82119800
H	5.25813500	3.54691300	-1.52557900
C	5.45327000	4.14683700	1.17485000
H	5.75999200	4.82305800	1.98108500
H	4.86486400	3.33422000	1.61774300
H	6.36733500	3.70567100	0.75228200
P	-3.28194100	-0.41759000	-1.83414700
N	-0.25885300	0.84771000	-1.27203100
C	-0.24216800	2.17461600	-0.99145100
O	-0.90763500	3.00501800	-1.60387100
C	0.78018400	2.57007300	0.07614900
N	0.77003300	1.70191800	1.22886800
C	-1.62167300	-0.85610600	-2.41161200
C	-0.66981200	0.33672800	-2.56705200
C	-4.06085300	0.69536900	-3.02794200
C	-0.34050200	1.38274800	1.93060000
C	-4.89728200	0.19070400	-4.03001000
C	-3.75369000	2.06226600	-2.98745300
O	-0.01109700	0.55353700	2.94017700
O	-1.46678400	1.78067300	1.67447800
C	-3.63117500	-3.16991100	-1.58884500
C	-4.26282400	-1.92697500	-1.69698300
C	-5.65854300	-1.83716700	-1.60106200
C	-4.39464000	-4.31867600	-1.39561100
C	-4.30519300	2.91733800	-3.93750700
C	-5.15458600	2.41735300	-4.92235700
C	-5.78171400	-4.22978800	-1.31507000
C	-6.41293200	-2.99012800	-1.41944600
C	-5.44465900	1.05579300	-4.97254600
C	0.56462800	-0.13355100	-3.36523200
C	1.60385700	0.96507200	-3.51796100

C	0.78737400	4.07167500	0.38477600
C	-0.40147600	4.57243800	1.18466600
C	-3.18887500	0.37785800	-0.19299100
H	1.74917200	2.37245300	-0.40190100
H	1.66949800	1.34436800	1.52957300
H	-1.19004200	-1.55059700	-1.68345700
H	-1.75048800	-1.37672800	-3.36750300
H	-5.12744700	-0.86935300	-4.07368300
H	-3.06561900	2.46099700	-2.24555200
H	-2.55057500	-3.25343300	-1.64671200
H	-3.90193800	-5.28133100	-1.30961900
H	-4.06544600	3.97498800	-3.90618500
H	-5.58544900	3.09053700	-5.65668000
H	-6.37325800	-5.12798000	-1.16707100
H	-6.09960800	0.66409900	-5.74367000
H	1.14863600	1.87266900	-3.92557400
H	2.40155700	0.63978400	-4.18913900
H	2.05334800	1.20294200	-2.54796700
H	0.80646500	4.57987600	-0.58921800
H	-1.33793900	4.32830500	0.67881900
H	-0.32788300	5.65848600	1.30349500
H	-0.40666800	4.11617800	2.17773300
H	-2.45113800	-0.20221400	0.37444800
H	-2.76623900	1.37813400	-0.32204600
H	0.38445500	0.26495500	-0.74290700
H	-1.14750200	1.16076300	-3.10626000
H	-6.15346800	-0.87059800	-1.65842200
H	-7.49365100	-2.91795800	-1.35348200
C	-4.54758600	0.39656700	0.45517000
C	-5.45551600	1.41293700	0.15784600
C	-4.93192200	-0.66274900	1.27753600
C	-6.75639000	1.37841100	0.66656400
H	-5.13325100	2.22766200	-0.48222400
C	-6.21693700	-0.72191100	1.81871300
H	-4.20979800	-1.44920800	1.47398000
C	-7.10807800	0.30638900	1.49454000
H	-8.11474100	0.27312000	1.90396300
C	-7.80033800	2.44534900	0.32223900
C	-8.96948700	1.77533900	-0.41870300
H	-8.62110500	1.30009300	-1.34234800
H	-9.45214400	1.00913800	0.19618500
H	-9.72615300	2.52217800	-0.68270300
C	-8.31986400	3.09636300	1.61368300
H	-7.50386500	3.57542400	2.16398400

H	-9.06695800	3.86114500	1.37526900
H	-8.79074500	2.36397700	2.27616300
C	-7.22568500	3.54364000	-0.57762000
H	-8.00441100	4.28166900	-0.79380100
H	-6.39356700	4.06745600	-0.09605300
H	-6.87287600	3.14172100	-1.53372200
C	-6.66082000	-1.85561400	2.74837200
C	-5.59744000	-2.95197500	2.86278800
H	-5.37105800	-3.39358400	1.88550900
H	-4.66484300	-2.57253000	3.29311400
H	-5.96389900	-3.75030200	3.51570900
C	-6.92142900	-1.27146200	4.14630100
H	-7.70110600	-0.50362400	4.11822500
H	-7.24581900	-2.05948300	4.83485200
H	-6.01202600	-0.81466400	4.55112100
C	-7.94983700	-2.49834700	2.21336400
H	-8.26959100	-3.30722200	2.87913300
H	-8.77147200	-1.77956800	2.14562100
H	-7.78523900	-2.92243900	1.21714900
O	1.08535600	-1.29611000	-2.74760300
H	0.20095200	-0.42771700	-4.36002000
C	-0.98617800	0.18995200	3.95629300
C	-2.18190100	-0.52584700	3.33622900
H	-2.75648800	-1.01628400	4.12916800
H	-2.83261600	0.17232800	2.81051300
H	-1.83220500	-1.28794500	2.63056900
C	-0.19871600	-0.76523000	4.84370500
H	0.09651300	-1.65393500	4.27675300
H	0.70674500	-0.28203400	5.22093400
H	-0.81040200	-1.07920200	5.69388900
C	-1.40014400	1.43864900	4.72621500
H	-1.93435200	2.13489200	4.07673800
H	-2.05861800	1.16024100	5.55452900
H	-0.51807600	1.93774100	5.13896000
H	1.91018400	-1.09012000	-2.27659000
N	3.55954800	0.34828500	-0.01745300
O	3.57068900	1.17219300	0.91022500
O	2.48737900	-0.13707600	-0.43232700
C	4.74697300	-0.04549800	-0.62182900
C	4.69335500	-1.19304300	-1.56997600
C	5.92489200	0.61597500	-0.28255400
C	6.03927500	-1.53018200	-2.11836700
C	7.10972300	0.23479900	-0.86117600
C	7.15801800	-0.85635900	-1.79759000

H	8.11504900	-1.12916300	-2.22665400
N	8.30815100	0.93458400	-0.52964600
O	9.35713700	0.56934800	-1.05624400
O	8.25379900	1.87117000	0.26529600
H	6.04969200	-2.36661200	-2.80967900
H	5.88897300	1.43438100	0.42749100
F	3.86673800	-0.90073300	-2.66603000
O	4.03192100	-2.34874800	-1.04918600
O	-0.38975900	-1.60695300	0.49577900
C	4.13827400	-2.63750900	0.28554400
C	2.98458400	-2.96053400	0.97873500
C	1.65519200	-2.84798100	0.33142100
C	1.26582400	-3.48322000	-0.78923000
H	0.30985700	-3.17642400	-1.20897400
C	2.01060400	-4.49706100	-1.54615500
C	1.93291200	-4.47738700	-2.94494500
H	1.34680400	-3.69787400	-3.42429400
C	2.63732400	-5.40745300	-3.70185200
H	2.58458400	-5.37306200	-4.78572100
C	3.40885900	-6.38101000	-3.07018100
H	3.95384600	-7.11177000	-3.65987000
C	5.40449400	-2.64607900	0.92486600
H	6.28768300	-2.39671600	0.34739100
C	5.50342000	-2.96221700	2.25191800
H	6.47223900	-2.95615400	2.74323300
C	4.35274200	-3.31206000	3.00500200
C	3.08468500	-3.32968200	2.35888900
C	3.47630700	-6.41833600	-1.67784800
H	4.06705100	-7.18237000	-1.18206000
C	2.78615300	-5.48048800	-0.91828400
H	2.83267900	-5.51646200	0.16614800
C	0.73734600	-1.83044000	0.91017100
H	1.13250900	-1.25566900	1.76761000
C	1.94765600	-3.72421600	3.11770100
H	0.97686100	-3.78508800	2.63297000
C	2.06472200	-4.04922300	4.44726100
H	1.18412900	-4.34583300	5.00904300
C	3.32392100	-4.00905400	5.09019200
H	3.40109800	-4.26742100	6.14144200
C	4.44213700	-3.65506400	4.37912100
H	5.41844100	-3.63087000	4.85600800

I-TS2-*R*

O	2.06226400	4.23628300	1.59770400
Si	3.12956200	5.49780400	1.31356900
C	2.32779000	6.78894300	0.20815200
H	2.23344600	6.46507200	-0.83334900
H	1.32890400	7.05893200	0.56792800
H	2.93457100	7.70113000	0.21610500
C	3.51734400	6.23894700	2.98943400
H	3.80353600	5.46111100	3.70445500
H	4.33295000	6.96810500	2.93774600
H	2.63565600	6.75060300	3.39021400
C	4.70439400	4.82303100	0.49673100
C	5.53892100	5.99152600	-0.04726300
H	5.78669300	6.72217600	0.73286100
H	6.48707600	5.61728000	-0.45689300
H	5.01897600	6.52301000	-0.85254100
C	4.33954200	3.88477100	-0.66101700
H	3.84975600	2.97938400	-0.28582700
H	3.67023900	4.35859200	-1.39093300
H	5.24610200	3.57372000	-1.19972200
C	5.53571900	4.04472000	1.52574200
H	5.86986700	4.68893200	2.34728300
H	4.96753500	3.21183400	1.95704400
H	6.43444600	3.62704500	1.05017500
P	-3.04214200	-0.08060700	-2.09695100
N	-0.05480100	0.95543100	-1.14939600
C	-0.11459200	2.22147100	-0.66852600
O	-0.82936500	3.09185800	-1.15839800
C	0.87440300	2.52712700	0.46142000
N	0.81531100	1.58428000	1.55294100
C	-1.37407700	-0.47355000	-2.68212200
C	-0.35590400	0.66485600	-2.54620500
C	-3.74617100	1.17721500	-3.19035300
C	-0.33439900	1.17608500	2.13566600
C	-4.40025400	0.79657700	-4.36865600
C	-3.54236800	2.53282500	-2.90518800
O	-0.05357500	0.28843400	3.10990100
O	-1.45032700	1.54927600	1.81002200
C	-3.46104500	-2.82688200	-1.97418600
C	-4.05526800	-1.57495000	-2.16554200
C	-5.44833300	-1.46402600	-2.26128700
C	-4.26121800	-3.96510600	-1.90596400
C	-4.02105400	3.49987200	-3.78566300
C	-4.69124500	3.12089800	-4.94597900
C	-5.64486400	-3.85648700	-2.02194500
C	-6.23780200	-2.60637100	-2.19654300

C	-4.87393100	1.77110400	-5.24071200	H	-9.55476900	2.86265400	-0.41374100
C	0.95571100	0.20415900	-3.21759800	C	-8.03148800	3.03781700	1.87526300
C	2.03758900	1.27663200	-3.15422900	H	-7.17400500	3.40471700	2.44853000
C	0.85697400	4.00232800	0.88086600	H	-8.76014500	3.85120900	1.78790400
C	-0.32786500	4.40327500	1.74073500	H	-8.49839700	2.22847400	2.44489700
C	-3.02161100	0.49951900	-0.36771300	C	-7.01083100	3.76540700	-0.27656300
H	1.86179500	2.36857700	0.00956900	H	-7.77032200	4.54921800	-0.35899400
H	1.69378200	1.21819000	1.89560000	H	-6.14673600	4.19128400	0.24375300
H	-1.00399100	-1.32980000	-2.11250000	H	-6.70185300	3.49286700	-1.29165000
H	-1.48283900	-0.76497000	-3.73364200	C	-6.56326600	-2.09743000	2.15728900
H	-4.54647900	-0.25401100	-4.60313400	C	-5.50682200	-3.20601500	2.13525400
H	-2.98504400	2.83934200	-2.02430500	H	-5.26054100	-3.50387800	1.10977800
H	-2.38645400	-2.92181100	-1.85241800	H	-4.58182700	-2.89660200	2.63356200
H	-3.79916800	-4.93580600	-1.75950500	H	-5.89010700	-4.08684100	2.66015500
H	-3.86205900	4.54978400	-3.56294800	C	-6.86468700	-1.73362900	3.62019300
H	-5.06615100	3.87791700	-5.62752400	H	-7.64852700	-0.97302700	3.69138500
H	-6.26414900	-4.74668200	-1.97082000	H	-7.20121800	-2.61964000	4.16987200
H	-5.38766100	1.47481100	-6.14933900	H	-5.96942200	-1.34184900	4.11456300
H	1.71133700	2.20124900	-3.64131600	C	-7.83838000	-2.63852300	1.49141200
H	2.94487700	0.90423000	-3.63210600	H	-8.18137900	-3.53880000	2.01279800
H	2.29425800	1.49403400	-2.11145800	H	-8.65304100	-1.90851400	1.51413700
H	0.84216800	4.58434100	-0.04995500	H	-7.64451900	-2.90046500	0.44577100
H	-1.26696000	4.18943500	1.22698300	O	1.33271400	-0.96931400	-2.55532100
H	-0.27049400	5.47371800	1.96221300	H	0.71904600	0.01032200	-4.28021400
H	-0.30742600	3.85588100	2.68751000	C	-1.09413900	-0.18033000	4.01389400
H	-2.31473700	-0.16357600	0.14326400	C	-2.13669900	-0.98994300	3.25085900
H	-2.58981800	1.50370700	-0.34726500	H	-2.81868300	-1.46019700	3.96709100
H	0.64601100	0.33144900	-0.75269200	H	-2.71609400	-0.35006200	2.58446300
H	-0.72728900	1.58566500	-3.00736300	H	-1.65519400	-1.78015900	2.66429100
H	-5.91736100	-0.49001000	-2.37200500	C	-0.31866800	-1.06899800	4.97768100
H	-7.31650700	-2.51804600	-2.27598700	H	0.17989500	-1.87726200	4.43419500
C	-4.39736600	0.43930900	0.24051900	H	0.44097600	-0.48746200	5.50771000
C	-5.28271000	1.50861100	0.10837900	H	-0.99956600	-1.50860300	5.71188600
C	-4.80502200	-0.72841900	0.88705200	C	-1.71741900	1.00095600	4.75001300
C	-6.58117000	1.42676500	0.61905500	H	-2.28779800	1.63426900	4.06914900
H	-4.94186300	2.40535000	-0.39899000	H	-2.39050600	0.62783800	5.52771900
C	-6.08919900	-0.84130800	1.42063500	H	-0.93685900	1.60003500	5.22937100
H	-4.09850800	-1.55057600	0.95433400	H	2.37288600	-1.03569500	-2.51374800
C	-6.95490900	0.24741100	1.27183400	N	3.61268000	0.14892600	0.40963300
H	-7.95929600	0.17409600	1.68214500	O	3.70740900	1.05399300	1.23713700
C	-7.59355500	2.56751300	0.47889100	O	2.55027600	-0.37043700	0.10411600
C	-8.82064700	2.05823800	-0.29538600	C	4.80553500	-0.29585000	-0.25705200
H	-8.53306000	1.70867500	-1.29288900	C	4.82309600	-1.53773800	-0.93914200
H	-9.31195000	1.23020500	0.22453100	C	5.86574900	0.59822900	-0.32417500

C	6.02486500	-1.89218300	-1.60361700
C	6.98045200	0.23730600	-1.05042300
C	7.07225500	-1.01380500	-1.68899300
H	7.97900800	-1.27510200	-2.22119300
N	8.07960200	1.17519500	-1.15198200
O	9.07798800	0.82447300	-1.76268200
O	7.95694200	2.27335900	-0.62663700
H	6.06272800	-2.87347700	-2.06202100
H	5.80333400	1.55692900	0.17685500
F	3.74122200	-1.05557300	-2.57992400
O	3.96509900	-2.55130400	-0.64550000
O	-0.81023000	-1.96728300	0.00451500
C	3.73171100	-2.89258700	0.66777400
C	2.44960300	-3.27226700	1.01614600
C	1.31446700	-3.07822200	0.08337200
C	1.23837300	-3.51334000	-1.18766700
H	0.41670600	-3.12484400	-1.78486300
C	2.20269200	-4.37527900	-1.88141700
C	2.59478000	-4.01842400	-3.17764700
H	2.18480400	-3.11309400	-3.61536900
C	3.54364900	-4.77679900	-3.85351700
H	3.85720800	-4.48389400	-4.85077500
C	4.09699700	-5.90554000	-3.24953300
H	4.83484200	-6.49953000	-3.78041400
C	4.79664400	-2.90824100	1.59998900
H	5.78868400	-2.60915900	1.27779300
C	4.56265500	-3.29464400	2.89249100
H	5.37298000	-3.29683000	3.61598900
C	3.27274300	-3.71932000	3.30290800
C	2.21345800	-3.73013500	2.35148000
C	3.69996500	-6.27241000	-1.96433700
H	4.12383300	-7.15500100	-1.49518000
C	2.76047300	-5.50844100	-1.27915800
H	2.44733600	-5.79266700	-0.27828000
C	0.26375000	-2.14222200	0.55709300
H	0.51003600	-1.57802700	1.47738400
C	0.93954800	-4.21274000	2.76434300
H	0.13200200	-4.27662600	2.04020100
C	0.72849200	-4.62824200	4.05613500
H	-0.24953000	-4.99598200	4.35124500
C	1.77575400	-4.58470800	5.00707200
H	1.59271300	-4.90955300	6.02630000
C	3.02014300	-4.14521000	4.63336000
H	3.83789000	-4.11955100	5.34866700

I-IM3-*R*

O	2.27790800	4.22456600	2.01722100
Si	3.35801100	5.47324400	1.71665400
C	2.44639600	6.94748100	0.99066800
H	2.12216200	6.78771800	-0.04287200
H	1.56108000	7.19488900	1.58566100
H	3.10018000	7.82659400	0.99780600
C	4.08129400	5.91640500	3.38401300
H	4.46093300	5.02788600	3.89828800
H	4.90630200	6.63079400	3.28966500
H	3.31662200	6.37074100	4.02255100
C	4.72584500	4.90328500	0.52529500
C	5.45275500	6.12963600	-0.04424100
H	5.86282800	6.77134100	0.74554800
H	6.29332500	5.80973600	-0.67506900
H	4.78958000	6.74336200	-0.66425800
C	4.12820200	4.09361700	-0.63331700
H	3.71222400	3.14810000	-0.26897800
H	3.33159000	4.63233800	-1.16258700
H	4.90675600	3.84493800	-1.36875500
C	5.73316300	4.01954500	1.27375400
H	6.25786200	4.57522300	2.05918100
H	5.24398000	3.15648200	1.74191900
H	6.49242400	3.63798400	0.57683500
P	-2.73652400	0.02491800	-2.00861400
N	0.21659400	0.98185100	-0.82205400
C	0.15726300	2.24583600	-0.33295400
O	-0.51041800	3.13548700	-0.85258600
C	1.09176600	2.52851900	0.85103300
N	0.94069400	1.59527700	1.94333800
C	-1.03592600	-0.38733900	-2.47289300
C	0.00059700	0.71681100	-2.24348700
C	-3.33884700	1.31380000	-3.12978900
C	-0.26447600	1.17381600	2.39456900
C	-3.98783700	0.97202700	-4.32196600
C	-3.06468100	2.65641400	-2.83872700
O	-0.09305900	0.20065100	3.30875800
O	-1.33609400	1.61292800	2.00945800
C	-3.19460400	-2.72001300	-2.09176100
C	-3.76723500	-1.44822800	-2.19263800
C	-5.15548400	-1.31142400	-2.32762700
C	-4.00823400	-3.84935500	-2.14640000
C	-3.46988100	3.64935000	-3.72658700

C	-4.13842000	3.30975700	-4.90034700
C	-5.38515200	-3.71298600	-2.29997100
C	-5.95832300	-2.44439700	-2.38919000
C	-4.38915300	1.97265000	-5.20129800
C	1.34003600	0.21549600	-2.82077500
C	2.43052000	1.28046100	-2.73937500
C	1.09134400	4.00611300	1.26139600
C	-0.11636100	4.42500900	2.07934700
C	-2.84571700	0.59743900	-0.27896900
H	2.09612200	2.34162400	0.45442300
H	1.77089500	1.14210400	2.29953500
H	-0.71745100	-1.26635300	-1.90823500
H	-1.07381200	-0.64935100	-3.53714700
H	-4.18572400	-0.06796600	-4.56356900
H	-2.50861400	2.92991100	-1.94574800
H	-2.12603700	-2.83798000	-1.94600500
H	-3.56121400	-4.83467400	-2.06628200
H	-3.25681000	4.68859100	-3.49875000
H	-4.45752300	4.08773900	-5.58675900
H	-6.01531700	-4.59586700	-2.34362500
H	-4.89965200	1.70571700	-6.12076600
H	2.16759100	2.20118100	-3.27253300
H	3.34544900	0.86524000	-3.17425100
H	2.63705500	1.52659000	-1.69041600
H	1.11440000	4.58286100	0.32716500
H	-1.03854500	4.21543900	1.53443500
H	-0.05720400	5.49529000	2.29750100
H	-0.13229800	3.88026000	3.02783700
H	-2.20987400	-0.09081900	0.28779900
H	-2.38695400	1.58818300	-0.21689100
H	0.89141600	0.34254900	-0.40367200
H	-0.30650500	1.65579800	-2.71489500
H	-5.60942100	-0.32490800	-2.37573500
H	-7.03221700	-2.33487400	-2.50126900
C	-4.27158000	0.57831600	0.20323000
C	-5.11243700	1.66935000	-0.01380900
C	-4.76887000	-0.57512300	0.81114900
C	-6.45504200	1.62349800	0.37172100
H	-4.70247200	2.55536700	-0.48742600
C	-6.10014500	-0.65266400	1.22121100
H	-4.09309900	-1.41478100	0.94473600
C	-6.91942900	0.45800600	0.99042100
H	-7.95861700	0.41391800	1.30787100
C	-7.41719200	2.79156600	0.13272900
C	-8.59622700	2.30800900	-0.72808500
H	-8.24300800	1.93185200	-1.69436000
H	-9.15489800	1.50601300	-0.23649300
H	-9.29080200	3.13402600	-0.91592500
C	-7.94603500	3.29929100	1.48407600
H	-7.12350200	3.64915600	2.11622100
H	-8.63974700	4.13323600	1.33056500
H	-8.47935500	2.51332000	2.02751400
C	-6.73846400	3.95628000	-0.59440700
H	-7.46599100	4.75771100	-0.75626200
H	-5.90937100	4.37006500	-0.01134900
H	-6.35231600	3.65021600	-1.57295400
C	-6.67510300	-1.89384400	1.90965400
C	-5.64986900	-3.02798600	1.99774200
H	-5.31015100	-3.33954300	1.00370100
H	-4.77165900	-2.73859800	2.58453300
H	-6.10607000	-3.89525500	2.48545500
C	-7.11425800	-1.51486400	3.33318300
H	-7.88224000	-0.73519900	3.31970100
H	-7.52746300	-2.38887500	3.84864400
H	-6.26448400	-1.14220400	3.91495700
C	-7.88914800	-2.40835200	1.11988100
H	-8.31232300	-3.29100300	1.61189200
H	-8.67982500	-1.65555800	1.04728800
H	-7.59492500	-2.69185300	0.10368000
O	1.70094800	-0.92485200	-2.10805100
H	1.16905000	-0.02051500	-3.88605200
C	-1.21032400	-0.24801400	4.13082300
C	-2.31670600	-0.84835900	3.26935000
H	-3.02841000	-1.36865700	3.91920800
H	-2.84654200	-0.07468200	2.71437700
H	-1.90196700	-1.57300300	2.55955200
C	-0.56986100	-1.31776900	5.00452900
H	-0.18166000	-2.13284300	4.38580300
H	0.25887000	-0.89679200	5.58066600
H	-1.30906900	-1.72683300	5.69865700
C	-1.71519300	0.91726900	4.97373000
H	-2.14797600	1.69612800	4.34289700
H	-2.48517100	0.56200500	5.66497900
H	-0.89521400	1.34383300	5.55960500
H	2.27649200	-1.50650100	-2.85970100
N	3.63562200	0.06954800	0.73373200
O	3.97721800	0.95184100	1.51052700
O	2.55962900	-0.50080900	0.79646600

C	4.52230800	-0.27325900	-0.36327700
C	4.38128000	-1.47975300	-1.07459100
C	5.40975700	0.71654200	-0.77174000
C	5.07742900	-1.62244600	-2.28227400
C	6.10837700	0.51153500	-1.94346500
C	5.93003600	-0.63215500	-2.72447000
H	6.46800800	-0.73648000	-3.65878900
N	7.03029700	1.55035700	-2.39707200
O	7.59789900	1.37893600	-3.46080800
O	7.18349200	2.53119300	-1.68672000
H	4.87261900	-2.50613200	-2.87101900
H	5.51551200	1.63143200	-0.20064800
F	2.85880900	-1.98509800	-3.84207800
O	3.64484000	-2.53835500	-0.69473000
O	-0.92237300	-2.04429600	0.17067400
C	3.58072400	-2.95455300	0.62059600
C	2.36120600	-3.39107100	1.09205000
C	1.15352600	-3.22656900	0.24675500
C	0.99401200	-3.80496300	-0.95692800
H	0.15987000	-3.46498800	-1.56965500
C	1.87129200	-4.84412400	-1.52000000
C	2.34896600	-4.72046400	-2.83098700
H	2.13986100	-3.81810200	-3.40193000
C	3.18692800	-5.70184000	-3.35307300
H	3.57467400	-5.59109100	-4.36142300
C	3.53511500	-6.81640300	-2.59125500
H	4.18237100	-7.58161700	-3.00929900
C	4.74930700	-2.97114200	1.41451700
H	5.69019900	-2.63355000	0.99097700
C	4.67608000	-3.42227800	2.70620600
H	5.56639500	-3.43949500	3.32800600
C	3.45015900	-3.88978800	3.24745400
C	2.28373100	-3.88190200	2.42959200
C	3.05506800	-6.94428300	-1.28909700
H	3.32135000	-7.81023700	-0.69092400
C	2.23321300	-5.95871300	-0.75226300
H	1.85294600	-6.05519300	0.26134500
C	0.15865300	-2.22958800	0.70639500
H	0.43976100	-1.64583500	1.60415000
C	1.06696500	-4.37877400	2.97299000
H	0.17625200	-4.40073300	2.35044600
C	1.01422300	-4.84202800	4.26457600
H	0.07806400	-5.21948500	4.66435000
C	2.17127800	-4.83559500	5.08025100
H	2.11272500	-5.20388800	6.09932700
C	3.36130500	-4.37293300	4.57901400
H	4.25831800	-4.37073100	5.19231500

II-IM1-*S*

P	2.84973600	-2.72206200	-0.70553800
N	-0.67504400	-3.24653900	-1.27577900
C	-1.92387100	-3.77049500	-1.16530900
O	-2.23564300	-4.87428700	-1.58950700
C	-2.94911900	-2.79089300	-0.60150200
N	-2.44557200	-2.05982900	0.54307500
C	1.59520900	-4.05839100	-0.78008600
C	0.43512600	-4.02163700	-1.78882600
C	3.83473500	-3.08276400	0.77584500
C	-1.91036600	-2.69869500	1.62371100
C	5.17851500	-2.68820100	0.80998600
C	3.23857700	-3.62028000	1.92082400
O	-1.92941800	-1.89354100	2.69174700
O	-1.47121200	-3.83800700	1.59508100
C	4.78533000	-4.01475600	-2.17200700
C	3.98384200	-2.86691800	-2.10590800
C	4.08024100	-1.88585100	-3.09563100
C	5.67959400	-4.17480400	-3.22328900
C	3.98734600	-3.78387700	3.08338300
C	5.32624600	-3.40292700	3.11039000
C	5.78518700	-3.18879400	-4.20329000
C	4.98774200	-2.04912700	-4.13952800
C	5.91871400	-2.85151100	1.97514000
C	0.82029000	-3.52650500	-3.18939800
C	-0.27225900	-3.79476700	-4.21000300
C	-4.33673500	-3.40925800	-0.37705400
C	0.24141100	-2.64047300	3.60461200
C	-1.20249900	-0.89989100	4.66656500
C	-1.19604100	-2.22090300	3.90809900
C	-1.96372100	-3.30371200	4.65495600
C	-4.41134800	-4.42219000	0.75092100
C	2.18207700	-1.02923800	-0.50438900
H	-3.07668100	-2.03707500	-1.38762900
H	-2.82321800	-1.13109900	0.68768300
H	2.20222200	-4.95643200	-0.94683500
H	1.15798900	-4.14290500	0.21908900
H	5.64487100	-2.24526700	-0.06542100
H	2.19371700	-3.90913300	1.93120100
H	4.72496900	-4.77525500	-1.39705400

H	6.29847800	-5.06453100	-3.27098900
H	3.51888000	-4.20741900	3.96607600
H	5.90722000	-3.52934400	4.01880200
H	6.49163400	-3.31082400	-5.01809900
H	6.95859000	-2.54207800	1.99700700
H	-0.46824500	-4.86573000	-4.30657900
H	0.02648400	-3.40126100	-5.18448900
H	-1.20458700	-3.30540900	-3.90667200
H	-4.61763600	-3.90144300	-1.31956200
H	0.28034700	-3.64749700	3.18866000
H	0.81897500	-2.61829100	4.53443300
H	0.68722900	-1.93092700	2.90078600
H	-0.66799000	-0.13944900	4.08905500
H	-0.71440600	-1.02189800	5.63769200
H	-2.22736800	-0.55421900	4.83291200
H	-2.98761900	-2.97211700	4.85305900
H	-1.47698700	-3.51231700	5.61251100
H	-1.99531800	-4.22456400	4.06807600
H	-3.68571700	-5.22386600	0.60069600
H	-5.41896100	-4.84580000	0.78776400
H	-4.21568700	-3.93553100	1.71128000
H	1.89080300	-0.67983400	-1.49603800
H	1.27554500	-1.14401300	0.10477900
H	-0.52193800	-2.33714600	-0.85191000
H	0.06892800	-5.04947500	-1.86988800
H	3.44003000	-1.01173600	-3.06695400
H	5.06718000	-1.28367400	-4.90445500
C	3.15105200	-0.10226500	0.18667600
C	3.17248700	-0.05299900	1.58137900
C	4.03389700	0.68359700	-0.55213300
C	4.07454400	0.76908000	2.25584800
H	2.46659700	-0.66649700	2.12936200
C	4.96010500	1.51105700	0.08848000
H	3.98700700	0.64575000	-1.63501900
C	4.95929700	1.53534800	1.48683400
H	5.67079100	2.18119600	1.99732100
C	4.12705000	0.83208700	3.78616200
C	3.07723100	-0.08273400	4.42574200
H	2.06126400	0.19067200	4.11832500
H	3.24857100	-1.13209100	4.15952400
H	3.13205000	0.00129500	5.51577100
C	5.51827500	0.38542100	4.26304400
H	6.30659200	1.03640800	3.87230400
H	5.56935900	0.41536100	5.35702100
H	5.72902700	-0.63816400	3.93598900
C	3.87222100	2.27093300	4.26110300
H	3.93215000	2.32087800	5.35399300
H	4.61251800	2.96851800	3.85579500
H	2.87749000	2.61689800	3.96122400
C	5.95439400	2.37807900	-0.68964900
C	5.84138200	2.16572000	-2.20273200
H	6.03264500	1.12357100	-2.48298300
H	4.85213600	2.45460600	-2.57263900
H	6.58159800	2.78940900	-2.71365900
C	5.67038500	3.85848500	-0.39338700
H	5.75770400	4.07804200	0.67633300
H	6.38570200	4.49419000	-0.92761000
H	4.65936400	4.12610100	-0.71545800
C	7.38647300	2.02840400	-0.25435900
H	8.10749900	2.64016900	-0.80745700
H	7.54332800	2.21245100	0.81268800
H	7.60960100	0.97433400	-0.45235200
O	-5.21468800	-2.33422400	-0.07577200
O	1.12313200	-2.13834800	-3.12248900
H	0.29352700	-1.66708800	-2.94136100
H	1.75132600	-4.01805400	-3.49771500
Si	-6.25525900	-1.63778100	-1.19608800
C	-5.37064000	-1.33778200	-2.82820500
H	-4.57722300	-0.58956400	-2.73728100
H	-4.93260400	-2.26119900	-3.22529400
H	-6.08460500	-0.97639600	-3.57736300
C	-7.68811600	-2.81497900	-1.49535700
H	-8.26081600	-2.99288000	-0.57932600
H	-8.37622300	-2.42978000	-2.25529100
H	-7.32326000	-3.78615300	-1.84913500
C	-6.80455100	-0.02542600	-0.38335200
C	-7.91436800	0.63479800	-1.21140100
H	-8.81542700	0.01157100	-1.26050000
H	-8.20278400	1.59443800	-0.76144800
H	-7.59008500	0.84278200	-2.23842000
C	-5.60362100	0.92549600	-0.27896800
H	-4.75271500	0.45200900	0.22288500
H	-5.24835400	1.26430900	-1.25663500
H	-5.86481100	1.81903400	0.30554800
C	-7.33182200	-0.33087800	1.02499100
H	-8.19412600	-1.00836900	1.00323200
H	-6.55643100	-0.79121900	1.64683500
H	-7.65014800	0.59768400	1.51842300

N	-1.62593100	0.48131200	-1.75970100
O	-0.66265200	-0.26369300	-1.56699400
O	-2.75753700	0.23192800	-1.41308500
C	-1.36383700	1.73242200	-2.44043600
C	-2.38662700	2.42746800	-3.08986400
C	-0.08475600	2.26247000	-2.35233700
C	-2.11430000	3.63914700	-3.71394000
C	0.15044800	3.49232700	-2.93980800
C	-0.84592600	4.18778600	-3.62209600
H	-0.62452900	5.15301400	-4.06157300
N	1.47272600	4.09259400	-2.78714300
O	1.63799100	5.21767300	-3.22587000
O	2.33925100	3.42957700	-2.24064700
H	-2.91925500	4.14741500	-4.23103400
H	0.70323400	1.73554600	-1.82937000
F	-3.59783700	1.91116200	-3.20931200
O	-3.13374900	3.07726600	-0.46998800
O	0.06006600	-0.13997800	1.53463100
C	-2.09567200	3.74055200	-0.20668800
C	-1.00980800	3.24639600	0.60092900
C	-1.14424900	1.91850100	1.22050800
C	-2.19485800	1.38250500	1.90220000
H	-2.06566800	0.33200000	2.16148800
C	-3.43479800	1.94780600	2.41956600
C	-3.77512800	3.31063000	2.39712500
H	-3.09139600	4.02759600	1.96162400
C	-4.96823500	3.74718300	2.96133000
H	-5.21329200	4.80484200	2.93967100
C	-5.85328200	2.84074400	3.54497500
H	-6.78937700	3.18990100	3.97060000
C	-1.93573100	5.08408800	-0.75486000
H	-2.79678300	5.48900600	-1.28105800
C	-0.76984300	5.77303800	-0.67214200
H	-0.66717500	6.75374100	-1.13479900
C	0.36071300	5.22812700	0.00929000
C	0.21786800	3.97689700	0.69066000
C	-5.52752500	1.48535500	3.58155600
H	-6.20726600	0.77032800	4.03547500
C	-4.32947100	1.04928500	3.02986400
H	-4.07115300	-0.00841500	3.05319500
C	-0.04603300	0.94658700	0.98666800
H	0.71106600	1.25653200	0.24038000
C	1.34939200	3.53748300	1.44570000
H	1.28259700	2.63763600	2.05338700
C	2.53863200	4.22910200	1.43379100
H	3.38219000	3.84200900	1.99790600
C	2.67546600	5.43555600	0.71408000
H	3.61915800	5.97192400	0.71757900
C	1.58836700	5.92518800	0.02982100
H	1.65400300	6.86458400	-0.51491400

II-TS1-*S*

P	2.90948100	-2.89534400	-0.20135200
N	-0.62697100	-3.56604600	-0.30909700
C	-1.88833500	-4.02913900	-0.10524100
O	-2.20483100	-5.20400600	-0.22407600
C	-2.91438600	-2.92161200	0.13426200
N	-2.46108300	-1.94305300	1.09481400
C	1.70167200	-4.22680500	0.16179400
C	0.45321300	-4.44195800	-0.70966000
C	4.03313800	-2.85288000	1.22219000
C	-1.87223800	-2.26854600	2.27379900
C	5.34991300	-2.41542100	1.02954100
C	3.55663000	-3.07259900	2.51824400
O	-1.71390900	-1.16181300	3.01936800
O	-1.52920400	-3.39623600	2.58975100
C	4.76515100	-4.45361400	-1.50823300
C	3.91260100	-3.34929500	-1.63608200
C	3.84715000	-2.64875100	-2.84309500
C	5.54686400	-4.85237800	-2.58652400
C	4.39432500	-2.86702100	3.61142000
C	5.70251200	-2.43162100	3.41662400
C	5.48649900	-4.14796100	-3.78810000
C	4.64014500	-3.04983500	-3.91487900
C	6.17838700	-2.20554000	2.12590600
C	0.70563400	-4.30819100	-2.21877700
C	-0.45075800	-4.83674100	-3.04947900
C	-4.32981100	-3.44873700	0.40294500
C	0.52455800	-1.66245500	3.85986700
C	-0.82354500	0.31821400	4.57863400
C	-0.87314100	-1.15860100	4.20793100
C	-1.53694200	-1.98449000	5.30216500
C	-4.51033500	-4.05548500	1.78092500
C	2.21107200	-1.20777300	-0.34401900
H	-2.96361600	-2.37855800	-0.81827000
H	-2.71673400	-0.97902900	0.92271300
H	2.31938600	-5.13255100	0.14797400
H	1.36777500	-4.07968000	1.19310400

H	5.72406800	-2.21976200	0.02875200	C	5.76142300	1.60742400	3.69070200
H	2.53570900	-3.39176800	2.69690200	H	6.49617400	2.15017300	3.08843200
H	4.82643300	-4.99308800	-0.56625200	H	5.81967100	1.99323400	4.71433600
H	6.20613000	-5.70813900	-2.48673900	H	6.04888500	0.55032000	3.70505700
H	4.01761700	-3.04158500	4.61415900	C	3.95869800	3.26367900	3.12071000
H	6.35108000	-2.26356600	4.27084600	H	3.96693600	3.67577200	4.13641400
H	6.10076100	-4.45660500	-4.62792600	H	4.65399100	3.84900700	2.51043600
H	7.19400200	-1.85618500	1.97089700	H	2.95689000	3.39504800	2.69945900
H	-0.63397100	-5.89473100	-2.84631400	C	6.03207200	1.90258200	-1.63476700
H	-0.22893200	-4.71199900	-4.11194900	C	5.84176000	1.28931500	-3.02554900
H	-1.36890900	-4.28386400	-2.82067400	H	6.00214400	0.20496000	-3.01617100
H	-4.52921600	-4.21739900	-0.35799800	H	4.83930100	1.49440200	-3.41414700
H	0.51434700	-2.73521600	3.65973700	H	6.56638900	1.72716700	-3.71938500
H	1.20238900	-1.46308500	4.69596400	C	5.80177600	3.41928400	-1.74262700
H	0.88814200	-1.12166300	2.97900300	H	5.93683500	3.91763500	-0.77750400
H	-0.37003700	0.89165200	3.76377200	H	6.51481800	3.85714000	-2.45028900
H	-0.22898300	0.45483200	5.48652500	H	4.78798200	3.62659200	-2.09838000
H	-1.83169300	0.70386800	4.75906400	C	7.47461000	1.63117300	-1.17642200
H	-2.54937900	-1.61322700	5.48887200	H	8.18549600	2.03562300	-1.90517900
H	-0.96297900	-1.89616100	6.22958000	H	7.68675700	2.09638000	-0.20893800
H	-1.59068800	-3.03662800	5.01702600	H	7.65882500	0.55523400	-1.08025300
H	-3.79253500	-4.86269500	1.94122600	O	-5.22205200	-2.34971100	0.26859600
H	-5.52628900	-4.44825400	1.87633800	O	0.96513300	-2.94335400	-2.52405200
H	-4.36634500	-3.28969900	2.54937900	H	0.13596000	-2.45046300	-2.41018100
H	1.81976700	-1.10159800	-1.35693800	H	1.62595500	-4.84875300	-2.47270300
H	1.35932300	-1.16411200	0.34595100	Si	-6.08328000	-2.06595600	-1.14661800
H	-0.47599200	-2.56700400	-0.21661200	C	-4.93067200	-2.01915700	-2.63335200
H	0.10834300	-5.45881700	-0.50069200	H	-4.22701800	-1.18247800	-2.58247600
H	3.16545300	-1.81479400	-2.96054600	H	-4.35721800	-2.94983900	-2.72482500
H	4.58980300	-2.50313900	-4.85062200	H	-5.51213200	-1.91373200	-3.55642100
C	3.22853000	-0.15309500	0.01196300	C	-7.31076800	-3.46875300	-1.39628500
C	3.32031900	0.27994000	1.33480600	H	-8.07733300	-3.48006900	-0.61470400
C	4.09679100	0.35886800	-0.94983900	H	-7.81502000	-3.38715300	-2.36509700
C	4.26035600	1.23561900	1.71088900	H	-6.80301900	-4.43960900	-1.37490300
H	2.62628200	-0.12778100	2.05909000	C	-6.94798600	-0.40773800	-0.88140500
C	5.06044800	1.31227600	-0.60787800	C	-7.91004600	-0.13651900	-2.04554100
H	3.99982600	0.02506600	-1.97669100	H	-8.69463500	-0.89939400	-2.11615200
C	5.12032000	1.73208700	0.72356800	H	-8.40635900	0.83380500	-1.90836500
H	5.85241100	2.48735100	1.00024400	H	-7.38611200	-0.09871600	-3.00840300
C	4.33684800	1.77406900	3.14143100	C	-5.90279900	0.71432600	-0.80665500
C	3.36840200	1.04046000	4.07553400	H	-5.17448000	0.53979900	-0.00862700
H	2.32706300	1.16361200	3.75572900	H	-5.34296700	0.81894300	-1.74090600
H	3.59219300	-0.03197700	4.11613300	H	-6.39089900	1.67723800	-0.60385100
H	3.45719300	1.44494800	5.08891100	C	-7.73741200	-0.45453800	0.43288200

H	-8.51641900	-1.22637700	0.41448900
H	-7.07836600	-0.65717700	1.28274300
H	-8.23135400	0.51044200	0.61313300
N	-1.59958700	0.07510100	-1.48956100
O	-0.71422300	-0.74240600	-1.21957300
O	-2.74475500	-0.01470000	-1.08859200
C	-1.24002400	1.18480700	-2.32437700
C	-2.23506500	1.92571200	-2.99718000
C	0.09421000	1.56224000	-2.37823000
C	-1.83596300	2.95379600	-3.87050300
C	0.43541000	2.65017600	-3.15877500
C	-0.52182000	3.35058200	-3.91219600
H	-0.20830300	4.18279200	-4.53112700
N	1.81987600	3.06716900	-3.19247500
O	2.11467200	4.04418000	-3.86203400
O	2.62907400	2.41124100	-2.54766800
H	-2.60419000	3.45311000	-4.44871600
H	0.84612100	1.02097100	-1.81506500
F	-3.44605400	1.41480700	-3.16338600
O	-2.98168400	3.02932300	-1.28884000
O	0.31841600	0.63139100	1.31073000
C	-2.14518100	3.92716600	-0.91237500
C	-1.24912400	3.74727400	0.17475300
C	-1.19873600	2.44119500	0.86289300
C	-2.19251400	1.73239100	1.45409100
H	-1.86807400	0.76348900	1.83176100
C	-3.58813600	2.03140900	1.75900000
C	-4.26567100	3.21153600	1.40873100
H	-3.75339900	3.98421700	0.85325200
C	-5.59106000	3.39751200	1.78559800
H	-6.10006000	4.31566100	1.50814200
C	-6.27253500	2.41671900	2.50558400
H	-7.31082200	2.56820800	2.78594100
C	-2.08447000	5.19037900	-1.61877100
H	-2.79383900	5.32270600	-2.43094600
C	-1.20276300	6.17301800	-1.28472600
H	-1.17821500	7.10838000	-1.83988800
C	-0.30804200	6.00933000	-0.18946900
C	-0.35290800	4.79227200	0.55864400
C	-5.61276200	1.24299700	2.86578100
H	-6.13031800	0.47406500	3.43124800
C	-4.28669200	1.05623700	2.49714400
H	-3.76867200	0.14231500	2.77972700
C	0.11812100	1.74372700	0.84707100
H	0.94568100	2.29260800	0.35257600
C	0.49442100	4.70237000	1.70297800
H	0.43809200	3.82188800	2.33842300
C	1.35083300	5.72262300	2.04522900
H	1.97777500	5.62351700	2.92712900
C	1.41085400	6.90622200	1.27625300
H	2.08953800	7.70515800	1.55706200
C	0.58419900	7.04025200	0.18681500
H	0.59592100	7.95153100	-0.40678600

II-IM2-*S*

P	3.13310300	-2.72629800	0.10880700
N	-0.34826400	-3.61928800	0.06201600
C	-1.59286400	-4.14652100	0.21550400
O	-1.82941400	-5.34634100	0.18263000
C	-2.70047500	-3.09374500	0.29456000
N	-2.39230800	-2.00811400	1.19606400
C	2.02224700	-4.12496400	0.53127100
C	0.77816500	-4.44839700	-0.31332800
C	4.20137400	-2.48812500	1.55538800
C	-1.87500500	-2.18701400	2.43376500
C	5.51808600	-2.04852000	1.37461500
C	3.66110800	-2.55630000	2.84417100
O	-1.80198100	-1.00092000	3.07229900
O	-1.53669600	-3.26269600	2.90096500
C	5.12421800	-4.22609800	-1.06243000
C	4.19660900	-3.19754100	-1.27557700
C	4.09812900	-2.58902200	-2.52997500
C	5.95112800	-4.63766100	-2.10150100
C	4.44205900	-2.21103800	3.94390700
C	5.75555400	-1.78479900	3.76177400
C	5.86005100	-4.02173600	-3.34898400
C	4.93707800	-3.00121800	-3.56154400
C	6.29063100	-1.70063100	2.47792800
C	1.01746500	-4.38602300	-1.83053800
C	-0.12196000	-4.99850000	-2.62712800
C	-4.09408300	-3.69953700	0.50911800
C	0.45885000	-1.31999000	3.93855700
C	-0.96109100	0.67369100	4.45090800
C	-0.95631900	-0.83617500	4.24340000
C	-1.57532500	-1.55869100	5.43303300
C	-4.31820500	-4.27775300	1.89347700
C	2.30323200	-1.11932800	-0.15594000
H	-2.71253500	-2.62589600	-0.69918400

H	-2.51826800	-1.06934200	0.83702400
H	2.70091300	-4.98636700	0.53044800
H	1.69712400	-3.97096500	1.56400700
H	5.93759700	-1.96319800	0.37664600
H	2.63352400	-2.86420200	3.00976100
H	5.21101200	-4.69424000	-0.08479000
H	6.66982000	-5.43317100	-1.93561700
H	4.01937800	-2.27407100	4.94151000
H	6.36097400	-1.51307200	4.62079700
H	6.50959900	-4.34021100	-4.15813100
H	7.30955000	-1.35749800	2.33163000
H	-0.29061200	-6.04092800	-2.34603900
H	0.10777500	-4.94994100	-3.69416300
H	-1.05098100	-4.44443700	-2.45105100
H	-4.20566400	-4.50030100	-0.23698800
H	0.49137300	-2.40763600	3.85240400
H	1.13214500	-1.00792800	4.74339800
H	0.79551400	-0.86524000	3.00010100
H	-0.53796000	1.16770800	3.57053800
H	-0.36404800	0.93768900	5.32860600
H	-1.98224800	1.03859600	4.60042300
H	-2.60053000	-1.21025100	5.59138900
H	-0.99586100	-1.34425400	6.33630500
H	-1.58871600	-2.63657600	5.26693000
H	-3.56942800	-5.03960500	2.11749400
H	-5.31615400	-4.72254400	1.94302500
H	-4.25920800	-3.48585100	2.64558100
H	1.92215500	-1.12882100	-1.17680900
H	1.43667400	-1.10961800	0.51546100
H	-0.29444700	-2.60906400	-0.03303700
H	0.49472500	-5.47142200	-0.05001000
H	3.35980700	-1.81758000	-2.71468100
H	4.86096000	-2.52366500	-4.53266500
C	3.20712500	0.05076900	0.13767700
C	3.14790100	0.65499900	1.39373200
C	4.11568600	0.51136500	-0.81240100
C	3.97457900	1.73181500	1.70822800
H	2.42829800	0.27898900	2.11110400
C	4.97305900	1.57870600	-0.52758500
H	4.14195300	0.04311800	-1.78795000
C	4.87763400	2.17438000	0.73295900
H	5.51897300	3.01998900	0.96616600
C	3.90403300	2.44010400	3.06319900
C	2.84327300	1.81352400	3.97402000

H	1.84715900	1.85351900	3.51820900
H	3.07616200	0.76576100	4.19565300
H	2.80193000	2.35821900	4.92274800
C	5.26725400	2.34442300	3.76515600
H	6.06142000	2.80307700	3.16810400
H	5.23277600	2.85948700	4.73167800
H	5.53747000	1.29773500	3.94176100
C	3.54355800	3.91675300	2.83666700
H	3.45226700	4.43737700	3.79665300
H	4.30685500	4.43249000	2.24609300
H	2.59192900	4.00529500	2.30259100
C	5.95085800	2.12011300	-1.57666700
C	6.18226100	1.11360500	-2.71172700
H	6.54603200	0.15211800	-2.33017600
H	5.26870200	0.94162000	-3.28844600
H	6.93568500	1.50916300	-3.40076100
C	5.35693600	3.40415800	-2.18077300
H	5.14865600	4.14759000	-1.40409200
H	6.05929700	3.84520200	-2.89732700
H	4.42417100	3.18437900	-2.71089100
C	7.30898900	2.43797900	-0.93236700
H	8.01537400	2.76771200	-1.70122300
H	7.24003800	3.23840500	-0.19004100
H	7.72904700	1.55394600	-0.44025500
O	-5.04427100	-2.66550600	0.29567700
O	1.23816400	-3.03825500	-2.22061300
H	0.39411900	-2.55602600	-2.17916700
H	1.95338000	-4.91504900	-2.05316600
Si	-5.85606400	-2.44183100	-1.15632800
C	-4.65743100	-2.38787100	-2.60207000
H	-3.95908300	-1.54901400	-2.51732700
H	-4.07838100	-3.31606200	-2.67979400
H	-5.20619800	-2.27547200	-3.54436600
C	-7.04983700	-3.87197800	-1.41152500
H	-7.82662400	-3.89201400	-0.64013200
H	-7.54285800	-3.80909200	-2.38766500
H	-6.52178200	-4.83149200	-1.37348800
C	-6.75431400	-0.79493200	-0.93178300
C	-7.73901300	-0.56079800	-2.08342400
H	-8.52232800	-1.32752100	-2.11399500
H	-8.23550100	0.41246700	-1.96764300
H	-7.23405800	-0.55349200	-3.05698000
C	-5.71426900	0.33350200	-0.89957600
H	-4.94341600	0.14584200	-0.14596700

H	-5.20225000	0.45287400	-1.85972700
H	-6.19509900	1.29067500	-0.65098500
C	-7.51617900	-0.81565700	0.39959600
H	-8.28285900	-1.59983500	0.42048300
H	-6.83604900	-0.98528400	1.24048400
H	-8.02122600	0.14692800	0.56268100
N	-1.46528700	-0.17144900	-1.53264200
O	-0.63560100	-1.07360600	-1.27386000
O	-2.60666000	-0.19993300	-1.06453800
C	-1.09494600	0.87245900	-2.37406600
C	-2.16212600	1.78788800	-2.88986200
C	0.24446300	0.98829900	-2.73330400
C	-1.64901400	2.61842900	-4.02464200
C	0.63234700	1.92437600	-3.65936400
C	-0.34843500	2.71751400	-4.34979400
H	-0.02187200	3.36319300	-5.15688700
N	2.01164800	2.05465500	-3.97635100
O	2.35535500	2.95573400	-4.73747000
O	2.81638600	1.26331100	-3.47690600
H	-2.41222600	3.16756000	-4.56629600
H	0.98000000	0.34383900	-2.27133400
F	-3.23542100	1.04490600	-3.35203400
O	-2.79121700	2.58101200	-1.87332700
O	0.05153600	0.53058100	1.14765600
C	-2.10808700	3.67298200	-1.39087900
C	-1.46603700	3.60256600	-0.17145200
C	-1.41716000	2.33065400	0.59296300
C	-2.42137100	1.75044900	1.28421400
H	-2.13858400	0.82205700	1.78038400
C	-3.80650200	2.14859300	1.53121900
C	-4.41839000	3.31248700	1.03716700
H	-3.85906200	4.00705900	0.42288400
C	-5.74703400	3.58745400	1.33709100
H	-6.20646200	4.49008000	0.94650900
C	-6.49075000	2.71127000	2.12758600
H	-7.53018600	2.93055200	2.35258300
C	-2.19121400	4.89550600	-2.10599300
H	-2.74940000	4.91416800	-3.03464200
C	-1.59841000	6.02554000	-1.61435300
H	-1.66060000	6.96211700	-2.16136800
C	-0.90940200	6.00014700	-0.37387800
C	-0.85454600	4.78325300	0.36397200
C	-5.89618600	1.55449300	2.62766800
H	-6.46918600	0.86706300	3.24206000
C	-4.56638000	1.27863000	2.33280000
H	-4.09939100	0.37010700	2.70886300
C	-0.11365400	1.61907500	0.62251000
H	0.73347200	2.14059800	0.13311000
C	-0.20853100	4.79065300	1.63037500
H	-0.18698200	3.87922400	2.22072100
C	0.36691800	5.93697600	2.12144400
H	0.85695600	5.92051000	3.09033700
C	0.32339700	7.14012600	1.37853400
H	0.78113100	8.03861200	1.77986900
C	-0.30618400	7.16823700	0.16057000
H	-0.35688200	8.08758000	-0.41665900

II-TS2-*S*

P	-3.71765100	-2.06582900	-0.21923800
N	-0.63488600	-3.65416600	-0.37205600
C	0.50100500	-4.34158900	-0.69025500
O	0.54454800	-5.55550300	-0.81634700
C	1.74244900	-3.44555800	-0.74304000
N	1.54123700	-2.26070700	-1.54687700
C	-3.07839800	-3.71864800	-0.68835000
C	-1.85807800	-4.31912700	0.02784700
C	-4.87652000	-1.60386000	-1.53701000
C	0.96310500	-2.28085000	-2.76944900
C	-6.01541600	-0.85476600	-1.22125100
C	-4.57700300	-1.88586100	-2.87584300
O	1.04262500	-1.05587200	-3.33229900
O	0.45343900	-3.26347900	-3.28436700
C	-5.79098900	-2.96994100	1.34554800
C	-4.66356700	-2.13923200	1.31277800
C	-4.30027200	-1.39719300	2.43867700
C	-6.55184900	-3.05099500	2.50612200
C	-5.42581700	-1.44234600	-3.88503200
C	-6.57051500	-0.71341400	-3.56610700
C	-6.19397800	-2.30175700	3.62662400
C	-5.07263000	-1.47669200	3.59343900
C	-6.86023700	-0.41541800	-2.23668000
C	-1.99784100	-4.33830200	1.56008800
C	-0.98191700	-5.25072000	2.22370400
C	3.04644800	-4.19600100	-1.03909600
C	-1.27461200	-0.92561000	-4.06581500
C	0.45056500	0.79629300	-4.61545200
C	0.18468000	-0.69490500	-4.44776500
C	0.59347800	-1.46614400	-5.69715500

C	3.22028900	-4.58960400	-2.49268200	H	-3.53985300	4.41518100	-0.45285700
C	-2.38203000	-0.82073700	-0.19179300	C	-3.91546800	3.23554600	-2.85094100
H	1.84890700	-3.06795400	0.27933700	C	-3.98558600	2.23771300	-4.00977300
H	1.81729000	-1.36950600	-1.15286300	H	-3.01216600	1.76843300	-4.19363200
H	-3.94375400	-4.38288300	-0.57773600	H	-4.71780600	1.44575400	-3.81993000
H	-2.84290500	-3.66178200	-1.75466100	H	-4.28435400	2.75923400	-4.92455500
H	-6.24314600	-0.61015800	-0.18819000	C	-5.29927500	3.87992800	-2.67255400
H	-3.68517400	-2.43970500	-3.15021500	H	-5.29177000	4.63549200	-1.88107200
H	-6.08100800	-3.54314200	0.46796100	H	-5.61194800	4.36890500	-3.60156300
H	-7.42376900	-3.69586200	2.53397100	H	-6.04894000	3.12442200	-2.41392300
H	-5.19047900	-1.66771400	-4.92013100	C	-2.88719500	4.31943800	-3.21509700
H	-7.23181800	-0.37035900	-4.35554500	H	-3.17285300	4.80994300	-4.15196700
H	-6.79077200	-2.36400300	4.53105300	H	-2.81361800	5.08988400	-2.44181100
H	-7.74407500	0.16151300	-1.98536900	H	-1.89298800	3.87921600	-3.34954900
H	-1.08221300	-6.28009400	1.87094800	C	-2.60208100	3.67702800	2.05144200
H	-1.11776800	-5.23082100	3.30753400	C	-3.26650700	3.06584900	3.29534200
H	0.03623200	-4.91303300	1.99836000	H	-4.35766200	3.12209600	3.21875100
H	3.03337600	-5.10531500	-0.41974100	H	-3.00622600	2.01252400	3.42981500
H	-1.46898800	-1.98599300	-3.89227300	H	-2.95947900	3.60887900	4.19557600
H	-1.93190900	-0.57584600	-4.86850400	C	-1.07475600	3.71543200	2.23742900
H	-1.49965900	-0.35551500	-3.15773200	H	-0.59420200	4.21300900	1.38665400
H	0.17477300	1.33267300	-3.70103000	H	-0.81044700	4.26833200	3.14621900
H	-0.14000300	1.19094500	-5.44733400	H	-0.65541900	2.70742700	2.31852900
H	1.50906600	0.97903200	-4.82466800	C	-3.10520500	5.11440200	1.89540600
H	1.66325800	-1.33115500	-5.88380000	H	-2.91188700	5.66909900	2.81905200
H	0.04328800	-1.08309900	-6.56203600	H	-2.59276100	5.63948800	1.08316400
H	0.38651400	-2.53067700	-5.58463000	H	-4.18275500	5.14553700	1.70315100
H	2.38142200	-5.20512000	-2.82453900	O	4.11539100	-3.33974800	-0.66246400
H	4.15221400	-5.14891400	-2.61215400	O	-1.88183800	-3.00918600	2.05430600
H	3.27603800	-3.69368600	-3.11879500	H	-0.93773500	-2.80545700	2.14077300
H	-1.78000500	-1.02445800	0.69851200	H	-3.01525200	-4.66564300	1.80986700
H	-1.76331400	-1.06612100	-1.06409100	Si	4.82204300	-3.40531100	0.86885600
H	-0.49796000	-2.68180200	-0.12060500	C	3.55593300	-3.42833900	2.25198800
H	-1.77418200	-5.34899100	-0.33214700	H	3.21769200	-2.41073500	2.51218200
H	-3.41256600	-0.77488100	2.43532500	H	2.70153500	-4.08388300	2.04593100
H	-4.78133800	-0.90095900	4.46530100	H	4.04499900	-3.83236300	3.14828300
C	-2.80130300	0.62964100	-0.24628300	C	5.84406200	-4.98642300	0.93421900
C	-3.23777900	1.20106500	-1.44930000	H	6.51380300	-5.06389200	0.07146000
C	-2.62988700	1.43860300	0.86901700	H	6.45444600	-5.03224000	1.84324900
C	-3.49680300	2.56472300	-1.53987300	H	5.19864300	-5.87237700	0.93425700
H	-3.34330800	0.56481800	-2.32175600	C	5.90950400	-1.86450700	1.00493200
C	-2.89899300	2.81565100	0.82097900	C	6.31881800	-1.67231000	2.47465000
H	-2.23027400	1.00088500	1.78204800	H	6.80480300	-2.55896500	2.90346700
C	-3.33559300	3.35137600	-0.38668600	H	7.03272400	-0.84006200	2.55811700

H	5.43786800	-1.41237300	3.07429500
C	5.09829900	-0.63028000	0.58495900
H	4.63635900	-0.76465700	-0.39889900
H	4.31837500	-0.41958200	1.32654100
H	5.75964800	0.24860400	0.52336400
C	7.14605200	-2.01869300	0.11282000
H	7.77721700	-2.86065900	0.42299100
H	6.86826000	-2.17626100	-0.93685900
H	7.76163100	-1.10879500	0.15389100
N	1.22758700	-0.65756800	1.45762600
O	0.47779400	-1.64123500	1.40602600
O	2.10857500	-0.47112600	0.65991500
C	0.97574000	0.30188600	2.51339400
C	1.87622300	1.36240200	2.78881800
C	-0.14284700	0.07406500	3.28962400
C	1.62065600	2.13133000	3.93487900
C	-0.38274400	0.90566900	4.37354300
C	0.51132700	1.90748800	4.73113500
H	0.31825200	2.51869300	5.60497900
N	-1.60311600	0.71682700	5.13421200
O	-1.77661100	1.39771500	6.12954500
O	-2.41147900	-0.10667700	4.72074800
H	2.30876100	2.92112500	4.20102800
H	-0.81315100	-0.74775000	3.07458700
F	3.18034700	-0.49748600	3.18313300
O	2.82587400	1.75250200	1.89473700
O	0.03828000	0.50465400	-0.98145800
C	2.71531100	3.05340200	1.40764700
C	2.16969200	3.25343300	0.15658000
C	1.78353400	2.09851400	-0.69652900
C	2.58625700	1.44234200	-1.56119500
H	2.09478600	0.62574800	-2.09163700
C	3.99458800	1.61462400	-1.91372300
C	4.83556900	2.63157700	-1.43217800
H	4.45206500	3.39531600	-0.76633200
C	6.17501800	2.66391500	-1.80078100
H	6.81550100	3.45279800	-1.41925000
C	6.70147400	1.68761500	-2.64642700
H	7.75165800	1.71508200	-2.92030900
C	3.25597200	4.12333200	2.15966200
H	3.78856000	3.90384700	3.07809600
C	3.13013800	5.40819100	1.70386900
H	3.53806600	6.23513700	2.27803700
C	2.47103800	5.68211400	0.47686300
C	2.01130500	4.59484300	-0.31960200
C	5.87746400	0.67718600	-3.13690200
H	6.27940400	-0.08814800	-3.79301100
C	4.53621700	0.64717100	-2.77759300
H	3.89086400	-0.14996400	-3.14119100
C	0.38955100	1.61119900	-0.60874600
H	-0.34756300	2.31329700	-0.17226700
C	1.39006200	4.87831600	-1.56653400
H	1.05541700	4.05389200	-2.18864700
C	1.21034700	6.17458700	-1.98029400
H	0.72345200	6.37432900	-2.92984900
C	1.65726100	7.25443200	-1.18160600
H	1.51031600	8.27386400	-1.52384600
C	2.28111100	7.01090400	0.01518000
H	2.63714700	7.83061200	0.63331300

II-IM3-*S*

P	3.95354400	-1.17303800	-0.37463100
N	1.40336600	-3.52352500	-0.82490900
C	0.55735400	-4.57006000	-0.63839700
O	0.83894500	-5.72951600	-0.90102300
C	-0.82973900	-4.12428500	-0.15655200
N	-0.74210600	-3.18475600	0.94719200
C	3.75599500	-2.99351800	-0.40951500
C	2.73911600	-3.65829400	-1.35377600
C	5.06869300	-0.82279400	1.01624000
C	0.14201600	-3.33692500	1.96172300
C	5.71874500	0.41994900	1.04541100
C	5.26226200	-1.73205800	2.06035100
O	0.08171300	-2.27445900	2.79585100
O	0.90156400	-4.28343200	2.08830500
C	6.18606400	-0.88696400	-1.94948700
C	4.82707700	-0.56178300	-1.83579600
C	4.20853100	0.22053800	-2.81378000
C	6.91565400	-0.43276300	-3.04130300
C	6.09737600	-1.40053300	3.12556300
C	6.74489600	-0.16918600	3.14881000
C	6.30040600	0.36094400	-4.00864800
C	4.95234900	0.68866100	-3.89374500
C	6.55576600	0.74020500	2.10781000
C	2.79258200	-3.15379500	-2.80525800
C	2.07807400	-4.09432300	-3.75930700
C	-1.78601200	-5.30146700	0.08431000
C	2.33682800	-2.27697200	3.73705700

C	0.43174600	-0.95691300	4.68697400	C	2.78765000	3.48541100	-0.08017900
C	0.84166000	-2.27348500	4.04010300	H	2.30969400	1.98509800	-1.55579700
C	0.42492100	-3.44976000	4.91580500	C	3.02136800	3.69484600	1.27764200
C	-1.49487300	-6.08101800	1.35226500	H	3.21246600	4.70241400	1.63021300
C	2.35750400	-0.30881400	-0.07787100	C	3.25270700	2.94951100	3.69523700
H	-1.24722900	-3.54305200	-0.98891000	C	3.40161000	1.66823800	4.52134400
H	-1.26889800	-2.31960200	0.88555600	H	2.48207000	1.07575000	4.52551600
H	4.76437200	-3.37316000	-0.61129200	H	4.21530900	1.03776100	4.14334800
H	3.48557000	-3.28738600	0.61019600	H	3.62948900	1.92748700	5.56002900
H	5.56963100	1.13752700	0.24243600	C	4.54199000	3.76945700	3.86059000
H	4.77528600	-2.70062600	2.06169000	H	4.48833600	4.73392700	3.34822200
H	6.67926900	-1.47965600	-1.18360300	H	4.72406200	3.96952600	4.92187700
H	7.96594300	-0.68991500	-3.12970700	H	5.40316300	3.22009600	3.46487800
H	6.24279300	-2.11354200	3.93020700	C	2.05834300	3.75242600	4.23461400
H	7.39744100	0.08451300	3.97823200	H	2.19976700	3.97798000	5.29745500
H	6.87511400	0.72345100	-4.85516700	H	1.93808400	4.69847800	3.69749200
H	7.06232500	1.69965600	2.12077800	H	1.12774900	3.18562600	4.12320100
H	2.54251300	-5.08375600	-3.75910200	C	2.79179600	4.62347400	-1.10612500
H	2.10911700	-3.68961400	-4.77367700	C	3.63320500	4.22263000	-2.33031900
H	1.02870500	-4.21037500	-3.46403200	H	4.66311100	3.98661600	-2.03966300
H	-1.67600800	-5.97160100	-0.78046600	H	3.22645900	3.35135100	-2.85297300
H	2.65277700	-3.23881900	3.33083900	H	3.65956200	5.04829800	-3.04895300
H	2.90274800	-2.07336700	4.65175300	C	1.34218800	4.88977800	-1.54117800
H	2.57087100	-1.48523500	3.01638200	H	0.74995500	5.24984200	-0.69463300
H	0.65268800	-0.11787700	4.01875100	H	1.31256800	5.63451900	-2.34458200
H	0.97872300	-0.81391100	5.62309200	H	0.87150100	3.97160200	-1.90496000
H	-0.64002300	-0.95047300	4.90423300	C	3.37217000	5.91884000	-0.52980900
H	-0.66117600	-3.44983700	5.05213600	H	3.39662200	6.68420000	-1.31186800
H	0.89067200	-3.34893400	5.90064700	H	2.76104400	6.30762300	0.29048500
H	0.72972500	-4.40058100	4.47769100	H	4.39483700	5.77714700	-0.16385100
H	-0.46685200	-6.44755000	1.34880300	O	-3.10446000	-4.78969900	0.16553000
H	-2.18558400	-6.92520200	1.42643000	O	2.22632700	-1.84936600	-2.86044000
H	-1.64218400	-5.44006000	2.22715500	H	1.26006600	-1.94134600	-2.87865500
H	1.80210700	-0.38067600	-1.01577800	H	3.84317000	-3.03857300	-3.10169900
H	1.83606200	-0.91433500	0.67710900	Si	-4.08611900	-4.47790800	-1.16997500
H	1.00315700	-2.60003300	-0.71038100	C	-3.07980800	-4.09142300	-2.70568300
H	2.96774200	-4.72823000	-1.33820700	H	-2.77758600	-3.03093500	-2.72009600
H	3.15166500	0.44850100	-2.76195900	H	-2.20092800	-4.73836900	-2.81766300
H	4.46463900	1.30281600	-4.64343200	H	-3.71272600	-4.26265200	-3.58582300
C	2.53234500	1.11464900	0.39246500	C	-5.13479200	-6.01097200	-1.46122200
C	2.76402000	1.36136700	1.74985400	H	-5.65774700	-6.31878400	-0.55000400
C	2.52406600	2.17528600	-0.50688000	H	-5.88510000	-5.84435200	-2.24233200
C	3.00874100	2.64968900	2.21398700	H	-4.50843000	-6.85053100	-1.78406900
H	2.77439700	0.52022600	2.43536100	C	-5.13848700	-2.98499000	-0.67917600

C	-5.76077500	-2.36469900	-1.93932600
H	-6.33187900	-3.08822900	-2.53662000
H	-6.45422000	-1.55914100	-1.65621500
H	-4.97047100	-1.92235400	-2.55795200
C	-4.20593100	-1.93927500	-0.05373100
H	-3.65404400	-2.35392400	0.79608800
H	-3.50874600	-1.56358500	-0.81288900
H	-4.78758600	-1.07989200	0.31523300
C	-6.22796100	-3.39718800	0.31565600
H	-6.94415800	-4.10234800	-0.12422800
H	-5.80260100	-3.86649500	1.21188800
H	-6.79342800	-2.51425200	0.64702000
N	-1.00737100	-0.47708500	-1.24767200
O	-0.27008800	-1.37282500	-1.65312800
O	-1.45656600	-0.44611100	-0.12080800
C	-1.15260800	0.70609000	-2.07899300
C	-2.25055900	1.57872200	-1.92919600
C	-0.08684100	1.01688900	-2.89233400
C	-2.24940500	2.76935300	-2.66591400
C	-0.10577400	2.22782300	-3.57498300
C	-1.17688300	3.10392500	-3.48086500
H	-1.16224100	4.04162100	-4.02327000
N	1.07737500	2.61480900	-4.32272300
O	1.10603100	3.72083800	-4.83169400
O	2.00405800	1.81441800	-4.38048900
H	-3.09558500	3.44132900	-2.59394000
H	0.75190100	0.33891900	-2.99391100
F	-2.71970400	-1.01797800	-2.62361600
O	-3.23529100	1.23050800	-1.09271500
O	-0.62674000	4.29591900	1.14288600
C	-4.20519800	2.17586800	-0.79978000
C	-3.97094400	3.09457000	0.19800500
C	-2.67054900	3.07304100	0.91967300
C	-2.27178600	2.10031300	1.76471800
H	-1.23405000	2.16160100	2.09651500
C	-3.04659900	0.97416000	2.29623300
C	-4.42989300	1.03615700	2.51918100
H	-4.97702600	1.94226900	2.27957500
C	-5.09915000	-0.04583900	3.08273100
H	-6.16841800	0.01790200	3.25883300
C	-4.40399400	-1.20591700	3.42066000
H	-4.93221000	-2.05229100	3.84918900
C	-5.40675700	2.12293600	-1.53603900
H	-5.51619600	1.35151900	-2.29105100
C	-6.38187900	3.04973200	-1.28129400
H	-7.31374900	3.03377400	-1.83878000
C	-6.18941900	4.05222500	-0.29239600
C	-4.97990500	4.07348500	0.46148000
C	-3.02842700	-1.27757100	3.20398400
H	-2.47449600	-2.18104600	3.44521500
C	-2.35572200	-0.19131700	2.65821000
H	-1.28519500	-0.25264000	2.48741800
C	-1.69453200	4.14298200	0.58441400
H	-2.00650600	4.82492900	-0.23206700
C	-4.81681500	5.06689800	1.46515100
H	-3.90904900	5.07423400	2.06182800
C	-5.79470400	6.00477700	1.68602900
H	-5.65545400	6.76006400	2.45290600
C	-6.98788500	5.99398200	0.92461700
H	-7.75090400	6.74330500	1.10900100
C	-7.18068000	5.03591800	-0.03776200
H	-8.09647200	5.01235600	-0.62206500

II-IM1-*R*

P	-2.81169800	-0.55498200	-1.84826700
N	-0.65382200	1.99486400	-1.83654000
C	-0.66678300	3.20355700	-1.22018800
O	-1.48653000	4.07753700	-1.47586900
C	0.52554500	3.42890600	-0.28129100
N	0.70128300	2.38737100	0.70495600
C	-1.72994300	0.20001000	-3.10660600
C	-1.48224400	1.71145200	-2.99376100
C	-4.51001700	-0.54241200	-2.47511300
C	-0.26250500	2.04844200	1.59282900
C	-5.42550900	-1.47742900	-1.97606800
C	-4.93638600	0.45841600	-3.35234000
O	0.27440100	1.25844600	2.54609900
O	-1.43646200	2.38416500	1.52653400
C	-2.52676200	-3.15151700	-2.72472000
C	-2.34639900	-2.29063300	-1.63500500
C	-1.78518300	-2.77309300	-0.44996600
C	-2.14511800	-4.48516300	-2.62346900
C	-6.27710500	0.52547700	-3.72497000
C	-7.18804100	-0.39952500	-3.22014800
C	-1.59552700	-4.96661300	-1.43580100
C	-1.41754700	-4.11207600	-0.35048700
C	-6.76227500	-1.40077200	-2.34757500
C	-0.80243200	2.16198900	-4.31161700

C	-0.36040100	3.61663400	-4.30359400	H	-7.05778100	2.85304400	2.63142800
C	0.57655400	4.86226300	0.28016400	H	-8.75581600	3.00386800	2.14087600
C	-0.50117900	5.22721400	1.28482100	H	-8.18637400	1.51957300	2.92327100
C	-2.81909900	0.34760500	-0.25212600	C	-7.26261500	2.89442700	-0.11746200
H	1.40259500	3.33606400	-0.93450400	H	-8.13822900	3.54678700	-0.19312000
H	1.61616300	1.93746400	0.81884500	H	-6.42578000	3.50206000	0.24129100
H	-0.77513300	-0.32844200	-3.06008100	H	-7.02311200	2.53520200	-1.12467400
H	-2.18575000	-0.03371700	-4.07482200	C	-5.70948300	-2.56124600	2.76289500
H	-5.09659700	-2.25357100	-1.29074000	C	-4.47699300	-3.47143000	2.77735700
H	-4.23462800	1.18684400	-3.74833300	H	-4.21560100	-3.81720300	1.77082000
H	-2.96773200	-2.78501500	-3.64840800	H	-3.60382000	-2.96661800	3.20517500
H	-2.28155000	-5.14913900	-3.47056500	H	-4.68332300	-4.35392700	3.39067100
H	-6.60739000	1.30117200	-4.40783200	C	-6.01609100	-2.13408600	4.20718700
H	-8.23309900	-0.34222700	-3.50865800	H	-6.91715700	-1.51529800	4.26145000
H	-1.30008300	-6.00820500	-1.35790200	H	-6.17403000	-3.01663600	4.83668800
H	-7.47181200	-2.12101200	-1.95318100	H	-5.18476300	-1.55798900	4.62664500
H	-1.18400500	4.29003600	-4.05687900	C	-6.89519000	-3.36419800	2.20399400
H	0.03904700	3.87364400	-5.28784300	H	-7.05669600	-4.26638900	2.80383600
H	0.43695900	3.76543800	-3.56678600	H	-7.82223700	-2.78323900	2.21751300
H	0.49266600	5.53145800	-0.58977000	H	-6.70401300	-3.67257600	1.17021200
H	-1.49466200	5.03556500	0.87853900	O	1.83180300	5.01937200	0.92240700
H	-0.40422900	6.28796600	1.53483300	O	0.27889900	1.30741000	-4.62666000
H	-0.37093400	4.64737900	2.20207800	H	0.92410600	1.35983900	-3.90273200
H	-1.91794500	0.06137800	0.29938500	H	-1.53995100	2.01323900	-5.11114100
H	-2.70664200	1.40673600	-0.49992800	Si	3.22699600	5.59731600	0.20427100
H	0.02682900	1.29868900	-1.54363100	C	3.38676100	4.98479600	-1.56692700
H	-2.41653600	2.27387300	-2.87516900	H	3.49744100	3.89964100	-1.64722000
H	-1.61614200	-2.11191800	0.39412800	H	2.51467800	5.28216700	-2.16162100
H	-0.98417200	-4.48044100	0.57387100	H	4.26286000	5.44471100	-2.03865400
C	-4.10323700	0.10280700	0.50152200	C	3.16270000	7.47377200	0.15083400
C	-5.15744300	1.00458900	0.35473700	H	2.99009900	7.89972200	1.14406200
C	-4.27489000	-1.04487900	1.27501100	H	4.09323900	7.89865600	-0.24240900
C	-6.39024700	0.77015400	0.96718500	H	2.34969600	7.81127300	-0.50227600
H	-5.00045800	1.89070800	-0.25274900	C	4.62573100	4.96556700	1.32007100
C	-5.49175800	-1.31115800	1.90552600	C	5.91777000	4.83532200	0.50216400
H	-3.44538000	-1.73252600	1.38543600	H	6.20634800	5.78170000	0.02726900
C	-6.53082700	-0.39179800	1.73334800	H	6.75098700	4.53023100	1.15037800
H	-7.48403500	-0.58327700	2.22015000	H	5.81692600	4.08073300	-0.28592200
C	-7.57295700	1.73398500	0.83180600	C	4.24872100	3.59217000	1.89580300
C	-8.79110800	0.97638000	0.27994000	H	3.36334800	3.65464300	2.53658800
H	-8.56475000	0.54610100	-0.70135400	H	4.03333900	2.85046300	1.11874600
H	-9.10290500	0.16327900	0.94245600	H	5.08224300	3.20695900	2.50215800
H	-9.63987000	1.65982800	0.16801900	C	4.85228100	5.94638400	2.47829900
C	-7.91222700	2.30896200	2.21624800	H	5.18771700	6.92779500	2.12436500

H	3.93991900	6.09513500	3.06786500
H	5.62289200	5.55880000	3.15891100
C	-0.51133800	0.77718800	3.67018300
C	-1.65872400	-0.10067700	3.18419500
H	-2.14428400	-0.57694400	4.04222800
H	-2.40105200	0.48741200	2.64475100
H	-1.27295500	-0.89445500	2.53090000
C	0.50319300	-0.04812400	4.45062200
H	0.90494000	-0.84684600	3.81843100
H	1.33651500	0.57960100	4.77771200
H	0.03019400	-0.49544100	5.32928000
C	-1.00837000	1.96029200	4.49368700
H	-1.75009400	2.53942300	3.94114500
H	-1.46364800	1.59616500	5.41958800
H	-0.16876300	2.61152800	4.75582600
N	2.72965100	0.19614200	-2.50414000
O	2.15911900	-0.60784500	-3.21517800
O	2.30635800	1.32372800	-2.28258900
C	3.99449300	-0.20758200	-1.90928500
C	4.74871600	0.65571400	-1.10637700
C	4.43670600	-1.49742600	-2.16183000
C	5.91595600	0.20829000	-0.49890200
C	5.61821800	-1.90615500	-1.57260800
C	6.35347200	-1.08656800	-0.72051900
H	7.26095700	-1.45990100	-0.26154000
N	6.11000600	-3.25043100	-1.87159500
O	7.00973600	-3.68958500	-1.17600800
O	5.59709700	-3.84635900	-2.79977700
H	6.46350700	0.89614000	0.13538100
H	3.86444200	-2.16136000	-2.79734800
F	4.42178300	1.92609200	-0.95394100
O	2.81797700	0.50690300	0.68093700
O	0.24167300	-0.61617700	-1.06793800
C	3.03409900	-0.37319400	1.59322500
C	2.57416700	-1.71152200	1.45189200
C	1.79560300	-1.92760200	0.22030700
C	1.78753000	-2.90786800	-0.71559100
H	1.03973500	-2.77506500	-1.49840500
C	2.61282100	-4.10526700	-0.88200500
C	3.78147100	-4.37946000	-0.14954400
H	4.12573600	-3.67258000	0.59770800
C	4.50668600	-5.54227000	-0.38828100
H	5.41031400	-5.73496500	0.18177900
C	4.09262800	-6.44688100	-1.36468000
H	4.66509200	-7.35075000	-1.54876300
C	3.77962100	-0.04763400	2.78719600
H	4.15097800	0.96963400	2.87796900
C	4.00689000	-0.97321600	3.76085700
H	4.57791900	-0.70764500	4.64835000
C	3.48858100	-2.30023400	3.66430600
C	2.74270100	-2.66302600	2.50108200
C	2.94810100	-6.17885400	-2.11315500
H	2.62480500	-6.87049800	-2.88517900
C	2.22089700	-5.01936900	-1.87475000
H	1.32953100	-4.80499000	-2.45982600
C	0.81181100	-0.83416200	-0.00532700
H	0.53726100	-0.27548200	0.90135500
C	2.17261000	-3.96701800	2.46990700
H	1.57641600	-4.25619900	1.61053900
C	2.35764300	-4.85507100	3.50381900
H	1.90666400	-5.84188600	3.44795900
C	3.12482600	-4.50005100	4.63509500
H	3.27105600	-5.21279400	5.44017700
C	3.67141400	-3.23938100	4.70573400
H	4.25105900	-2.93827800	5.57564400

II-TS1-*R*

P	-2.80282600	-0.64974500	-1.86505200
N	-0.68972700	1.93162500	-2.00712800
C	-0.74415900	3.15319100	-1.41785500
O	-1.61946700	3.97533300	-1.66455500
C	0.45393600	3.46836100	-0.51447100
N	0.74641000	2.42314000	0.44311800
C	-1.78313600	0.07810100	-3.18825200
C	-1.54622800	1.59424600	-3.12848500
C	-4.53038100	-0.65631500	-2.40752700
C	-0.14395900	2.06741700	1.40276500
C	-5.42268800	-1.57203900	-1.83544900
C	-4.99649600	0.31465400	-3.29847200
O	0.48056300	1.36368100	2.36695700
O	-1.33858800	2.32248800	1.37113100
C	-2.54043900	-3.26558800	-2.68989600
C	-2.31016300	-2.37524600	-1.63374600
C	-1.68847000	-2.82452900	-0.46595600
C	-2.15097300	-4.59544000	-2.57193400
C	-6.35304000	0.37285300	-3.60976600
C	-7.24032300	-0.53160500	-3.03204600
C	-1.54499300	-5.04467700	-1.39938300

C	-1.31476600	-4.16052700	-0.34809100	H	-8.92650000	0.17428300	1.21188000
C	-6.77510700	-1.50508800	-2.14798900	H	-9.48861800	1.67610900	0.46437200
C	-0.94461700	2.01958900	-4.49558100	C	-7.67689600	2.30956400	2.44420000
C	-0.41935700	3.44652900	-4.50461800	H	-6.80188500	2.84295000	2.82936600
C	0.41228800	4.89400500	0.07597100	H	-8.51550400	3.01332300	2.40244000
C	-0.73324200	5.21965200	1.01999100	H	-7.93357100	1.52062300	3.15817500
C	-2.73101500	0.28923600	-0.29367200	C	-7.11414800	2.89896800	0.08899300
H	1.31010500	3.45734500	-1.19695500	H	-7.98504300	3.56085500	0.05283200
H	1.70533100	2.09269100	0.55406700	H	-6.25575700	3.49531500	0.41424300
H	-0.82422300	-0.44405000	-3.16860500	H	-6.92098300	2.54197600	-0.92860100
H	-2.27622900	-0.18764000	-4.12979400	C	-5.46346300	-2.54828000	2.93201300
H	-5.06477600	-2.32629500	-1.14037400	C	-4.24617800	-3.47709300	2.87674200
H	-4.31470100	1.02632600	-3.75467700	H	-4.05805700	-3.83792500	1.85901400
H	-3.02868900	-2.92564900	-3.59972900	H	-3.33899200	-2.98070200	3.23837000
H	-2.32760200	-5.28206300	-3.39304000	H	-4.42374100	-4.34919900	3.51340900
H	-6.71348800	1.12523200	-4.30323800	C	-5.66525400	-2.09430700	4.38659100
H	-8.29755600	-0.48148500	-3.27346100	H	-6.54987300	-1.45816500	4.48951000
H	-1.24769900	-6.08452900	-1.30602800	H	-5.79615600	-2.96399500	5.03973600
H	-7.46630000	-2.21087100	-1.69871500	H	-4.79773800	-1.52707700	4.73979800
H	-1.18204800	4.16099900	-4.18692200	C	-6.69599100	-3.34160900	2.46868000
H	-0.08327900	3.69773500	-5.51356000	H	-6.82875200	-4.23000700	3.09555900
H	0.43980100	3.53585200	-3.82966800	H	-7.61193800	-2.74698600	2.53451700
H	0.35938700	5.57317600	-0.78913900	H	-6.58127300	-3.67199500	1.43044700
H	-1.69487700	4.97268700	0.57057100	O	1.61758900	5.08654900	0.79762000
H	-0.70047900	6.28853700	1.25207600	O	0.04448400	1.10945700	-4.92675400
H	-0.62128200	4.66513600	1.95427500	H	0.77630900	1.09023400	-4.28797900
H	-1.79712600	0.02653600	0.21326400	H	-1.75874300	1.93005200	-5.22758700
H	-2.64302400	1.34074500	-0.57780900	Si	3.06918200	5.65600700	0.19403400
H	0.07266500	1.30515600	-1.76481500	C	3.42216600	4.97172900	-1.52013000
H	-2.48158500	2.14739400	-2.98510500	H	3.54498200	3.88481700	-1.53988100
H	-1.48334500	-2.14164500	0.35236600	H	2.61849400	5.23647100	-2.21826000
H	-0.84027500	-4.50514000	0.56543800	H	4.34275200	5.41987200	-1.91182500
C	-3.96878700	0.06420700	0.53784800	C	2.98591100	7.52633500	0.05062000
C	-5.02074600	0.97518300	0.44020800	H	2.68336900	7.99372500	0.99255500
C	-4.10505500	-1.06930400	1.33833600	H	3.95122000	7.95128300	-0.24700300
C	-6.21707200	0.76312000	1.12789200	H	2.25556800	7.81628200	-0.71367900
H	-4.88999500	1.85121800	-0.18725900	C	4.34637600	5.09370400	1.47785000
C	-5.28557800	-1.31418200	2.04261000	C	5.73489800	5.01900800	0.82759400
H	-3.27771700	-1.76504100	1.40883300	H	6.04029300	5.97656300	0.38700400
C	-6.32416500	-0.38679500	1.91759400	H	6.49211900	4.75079000	1.57739700
H	-7.24932900	-0.56197800	2.46127800	H	5.76744600	4.25831600	0.03944300
C	-7.39604200	1.73722300	1.04546300	C	3.96745300	3.70453000	2.01251200
C	-8.63918300	0.98860000	0.53964600	H	2.99273900	3.71119100	2.51137700
H	-8.45448900	0.56080900	-0.45144100	H	3.92570600	2.95110500	1.21806300

H	4.72035700	3.37417800	2.74354200
C	4.38516800	6.08647300	2.64786900
H	4.71511600	7.08197400	2.32990600
H	3.40295100	6.19322400	3.12309000
H	5.08716600	5.73671200	3.41737100
C	-0.24277400	0.89125600	3.53941800
C	-1.30853200	-0.11868000	3.13014200
H	-1.74914100	-0.56713500	4.02657700
H	-2.10181400	0.36002400	2.55616000
H	-0.85083800	-0.92437700	2.54063200
C	0.84828900	0.21197000	4.35383400
H	1.28616600	-0.61376500	3.78497100
H	1.64195900	0.92381700	4.59695500
H	0.42982100	-0.18375800	5.28345500
C	-0.83043900	2.07450600	4.30029200
H	-1.64394400	2.54168900	3.74414500
H	-1.21446800	1.72977300	5.26495500
H	-0.05076000	2.81967300	4.48826600
N	2.72357600	0.50832600	-2.56051900
O	2.21669800	-0.08914400	-3.50654100
O	2.28191400	1.57274900	-2.14175600
C	3.87019400	-0.06111100	-1.92633200
C	4.49678800	0.61123700	-0.84042300
C	4.26323600	-1.33886700	-2.30462800
C	5.62987100	0.00038500	-0.24843400
C	5.33104800	-1.92242900	-1.65638100
C	6.01414900	-1.26090100	-0.61634000
H	6.86131200	-1.74384600	-0.14431700
N	5.76900400	-3.23851900	-2.06924100
O	6.72866300	-3.72772800	-1.48931300
O	5.16470500	-3.79501000	-2.97238000
H	6.15257000	0.55437800	0.52291200
H	3.73926700	-1.85718300	-3.09773400
F	4.45881100	1.94529300	-0.78152500
O	3.05121900	0.51729800	0.55936900
O	0.35689400	-0.67257100	-1.22987000
C	3.21426900	-0.38258600	1.49825200
C	2.65733700	-1.67134200	1.38553200
C	1.87597500	-1.91778100	0.15443200
C	1.86669900	-2.95338200	-0.71791000
H	1.13884000	-2.84971700	-1.52399300
C	2.65703200	-4.18309900	-0.78777500
C	3.84028600	-4.41027000	-0.06584400
H	4.22542400	-3.63989100	0.59435900
C	4.54117300	-5.60022400	-0.22213000
H	5.46598400	-5.75141200	0.32533100
C	4.07317200	-6.58799500	-1.08789700
H	4.62525900	-7.51539000	-1.20482300
C	4.00845800	-0.07677100	2.65294200
H	4.45598100	0.91209200	2.70088800
C	4.17655100	-0.98236000	3.66037100
H	4.78144000	-0.73522500	4.52968000
C	3.54268200	-2.25668800	3.61333800
C	2.75736500	-2.59701500	2.47149900
C	2.90255400	-6.37502800	-1.81271900
H	2.53811900	-7.13358700	-2.49857400
C	2.20813700	-5.18043800	-1.66844800
H	1.30290500	-5.00134400	-2.24377600
C	0.92225200	-0.82144100	-0.15450000
H	0.69470800	-0.16289100	0.69523400
C	2.08346500	-3.84965200	2.48223800
H	1.46465000	-4.11902700	1.63273400
C	2.19944900	-4.71259600	3.54517200
H	1.66990700	-5.66055900	3.52677900
C	2.99846700	-4.37954500	4.66269700
H	3.08640800	-5.07299800	5.49281300
C	3.65082400	-3.17120200	4.69033600
H	4.25751600	-2.88992700	5.54770200

II-IM2-*R*

P	-2.78516700	-0.82249500	-1.74592500
N	-0.70886000	1.80142700	-2.03027800
C	-0.90248400	3.04395400	-1.52322300
O	-1.86783700	3.74520600	-1.80595100
C	0.26600700	3.53470700	-0.65921700
N	0.63220900	2.60936300	0.39186200
C	-1.64730500	-0.22333500	-3.03670600
C	-1.49549900	1.30075100	-3.14008600
C	-4.43860800	-0.95495400	-2.47028300
C	-0.21854600	2.22833100	1.37481000
C	-5.37539700	-1.80985300	-1.87670200
C	-4.80922000	-0.14909500	-3.55073100
O	0.48194900	1.63111200	2.36163100
O	-1.42904400	2.37901800	1.34908600
C	-2.42600000	-3.51265200	-2.22425000
C	-2.27995800	-2.49574400	-1.27263900
C	-1.68102000	-2.77273500	-0.04073500
C	-1.96978400	-4.79637300	-1.94126500

C	-6.11448700	-0.19934900	-4.03458100
C	-7.04522700	-1.04984500	-3.44340300
C	-1.37683800	-5.07161300	-0.71003300
C	-1.23827300	-4.06209000	0.24076700
C	-6.67509600	-1.85631700	-2.36711900
C	-0.86447700	1.62682900	-4.51906500
C	-0.38641400	3.06753300	-4.61687600
C	0.14181100	4.99876400	-0.19132300
C	-1.02860700	5.31778500	0.72339500
C	-2.89061400	0.30561700	-0.30729800
H	1.12107500	3.50986500	-1.34225500
H	1.61715600	2.40876500	0.52224300
H	-0.67301400	-0.67689600	-2.83664100
H	-2.01107900	-0.64041800	-3.98157600
H	-5.09256000	-2.42907800	-1.02957200
H	-4.09223700	0.51684200	-4.02202600
H	-2.89767600	-3.30640600	-3.18181200
H	-2.07993100	-5.58047400	-2.68298100
H	-6.39955100	0.42427500	-4.87519700
H	-8.06161500	-1.08751100	-3.82259600
H	-1.02097200	-6.07368300	-0.49195000
H	-7.39943200	-2.52075700	-1.90731900
H	-1.18182200	3.77396900	-4.36476100
H	-0.03666700	3.26563800	-5.63273200
H	0.45266300	3.23178600	-3.93120000
H	0.05833000	5.60588900	-1.10581800
H	-1.97031800	4.99372300	0.28044100
H	-1.05560600	6.39747600	0.89669400
H	-0.89733800	4.82100100	1.68739800
H	-1.97018000	0.18518900	0.26983700
H	-2.86385400	1.31840000	-0.72216600
H	0.16000600	1.32769700	-1.79088300
H	-2.46275300	1.81362800	-3.08678100
H	-1.53907800	-1.98584100	0.69214900
H	-0.78107900	-4.27127800	1.20282900
C	-4.15016100	0.08165600	0.48982400
C	-5.28615700	0.83839700	0.19924900
C	-4.21039000	-0.90774500	1.47019400
C	-6.48625900	0.61880700	0.87730300
H	-5.21556900	1.59846200	-0.57248400
C	-5.39335400	-1.15723300	2.16978200
H	-3.31953700	-1.48392900	1.68772300
C	-6.51290600	-0.38136300	1.85606800
H	-7.43954800	-0.55875200	2.39648600
C	-7.75843500	1.41759300	0.57745500
C	-8.87002900	0.44984100	0.13876200
H	-8.57332300	-0.09809800	-0.76231700
H	-9.10167900	-0.28146000	0.91892400
H	-9.78762300	1.00503500	-0.08422700
C	-8.20221300	2.16403600	1.84564700
H	-7.42585900	2.85866500	2.18204800
H	-9.11273100	2.73922800	1.64480300
H	-8.41619300	1.47312700	2.66687500
C	-7.54390700	2.44108100	-0.54122400
H	-8.47891700	2.97855500	-0.72729700
H	-6.78128700	3.17979200	-0.27437200
H	-7.24367000	1.95638600	-1.47666500
C	-5.48383800	-2.23070900	3.25852100
C	-4.19510700	-3.05317800	3.36131900
H	-3.96139300	-3.55542100	2.41566900
H	-3.33627600	-2.43394900	3.64218100
H	-4.31189300	-3.82342000	4.12996300
C	-5.74091300	-1.54730800	4.61132900
H	-6.67788000	-0.98172000	4.60164500
H	-5.80532300	-2.29543000	5.40901300
H	-4.93016400	-0.85284300	4.85575600
C	-6.64164800	-3.19205200	2.94396700
H	-6.70809600	-3.96606300	3.71591700
H	-7.60461900	-2.67407700	2.90951700
H	-6.48870400	-3.68591300	1.97827400
O	1.32309900	5.32199700	0.52298300
O	0.16180200	0.71863200	-4.85422400
H	0.84833100	0.71932700	-4.16364400
H	-1.65469600	1.45807300	-5.26300500
Si	2.85815300	5.68336600	-0.03768400
C	3.29528200	4.73790300	-1.60148400
H	3.34742900	3.65436000	-1.45109900
H	2.58108400	4.94176200	-2.40834400
H	4.27792400	5.06869100	-1.95846100
C	2.95413300	7.52231800	-0.40412600
H	2.61585900	8.11863700	0.44886600
H	3.97730600	7.82975700	-0.64780500
H	2.32314900	7.77978500	-1.26219500
C	3.99124400	5.20081100	1.40499900
C	5.44364900	5.10926300	0.91749700
H	5.78837200	6.04407200	0.45763400
H	6.11466200	4.89473000	1.76083800
H	5.56844400	4.30387500	0.18517100

C	3.56651300	3.83509300	1.96268900
H	2.52442000	3.84074000	2.29990500
H	3.68486700	3.04243000	1.21456300
H	4.19812800	3.56822900	2.82267200
C	3.88393600	6.25638800	2.51320400
H	4.23595500	7.23793200	2.17608000
H	2.85157200	6.37100600	2.86408700
H	4.49655100	5.96571900	3.37777000
C	-0.17785200	1.08928600	3.54080800
C	-1.18186200	0.00880800	3.14901600
H	-1.57339000	-0.46085500	4.05726000
H	-2.01552200	0.43438500	2.58843300
H	-0.68851000	-0.77097300	2.55360300
C	0.98320800	0.48448100	4.31895900
H	1.45948100	-0.31030800	3.73483400
H	1.73357600	1.24922900	4.53949500
H	0.62405000	0.05984900	5.26030900
C	-0.83270700	2.22334400	4.32005700
H	-1.67558500	2.64275400	3.76838800
H	-1.19283400	1.84782700	5.28232500
H	-0.10013900	3.01405100	4.50954600
N	2.68508600	0.36107400	-2.15107000
O	2.07791400	-0.26913400	-3.03624000
O	2.20976200	1.40628000	-1.67709300
C	3.90305900	-0.10274900	-1.66808100
C	4.45811800	0.55115500	-0.44833900
C	4.45795800	-1.24323200	-2.23963100
C	5.77075700	-0.03531400	-0.05459600
C	5.63462400	-1.75357200	-1.74680100
C	6.30770800	-1.11539400	-0.65081900
H	7.26266100	-1.50972100	-0.32355500
N	6.15877800	-2.95719500	-2.29831500
O	7.17169600	-3.43582200	-1.78794900
O	5.58222900	-3.48105400	-3.24793100
H	6.28091200	0.47517800	0.75549200
H	3.94884300	-1.72758300	-3.06403400
F	4.64398100	1.91684900	-0.65010200
O	3.49185200	0.55285000	0.63064400
O	0.12076600	-0.61029500	-0.66359200
C	3.58239400	-0.42537800	1.59785100
C	2.89065700	-1.61584600	1.45845500
C	1.98624400	-1.77704800	0.29055900
C	1.91215800	-2.74721400	-0.64765500
H	1.09845500	-2.61296400	-1.36262000
C	2.74268700	-3.92440900	-0.89058600
C	3.97333000	-4.16558200	-0.25525000
H	4.36402600	-3.45151600	0.46216600
C	4.71261600	-5.29854800	-0.56864100
H	5.67180400	-5.46194400	-0.08829900
C	4.23765900	-6.21162300	-1.51168500
H	4.82339100	-7.09242600	-1.75533400
C	4.33594000	-0.12913000	2.75875500
H	4.84423800	0.82836300	2.80950700
C	4.39335000	-1.02979500	3.78550400
H	4.96672900	-0.80941000	4.68148900
C	3.69326100	-2.26326500	3.70347900
C	2.92442100	-2.55946400	2.54093000
C	3.02241600	-5.98310400	-2.15162500
H	2.65507200	-6.68337500	-2.89490100
C	2.28637600	-4.84334500	-1.84833500
H	1.34411400	-4.65021800	-2.35544100
C	0.99173200	-0.68208700	0.18798300
H	1.06769200	0.07813500	0.97733200
C	2.20294900	-3.78341600	2.50578900
H	1.60976200	-4.02253900	1.62979000
C	2.25162400	-4.66174900	3.56005200
H	1.69500300	-5.59256200	3.51141000
C	3.02091500	-4.36722800	4.71001700
H	3.05099000	-5.07183300	5.53492000
C	3.72204400	-3.19084400	4.77756100
H	4.31203200	-2.94489700	5.65626400

II-TS2-*R*

P	3.51360000	-0.19943700	2.30838600
N	0.63412900	1.37973200	1.74721100
C	0.51730200	2.62382400	1.19027600
O	1.16772200	3.58316600	1.58845800
C	-0.57438000	2.78008500	0.13401100
N	-0.69947000	1.67483200	-0.78578900
C	1.96738700	-0.06757200	3.24462400
C	1.17466700	1.23726500	3.08927200
C	4.71636900	1.01479500	2.88838100
C	0.33038600	1.19156000	-1.49952400
C	5.89879300	1.24507000	2.16971000
C	4.47492400	1.70962800	4.07912100
O	-0.08616500	0.22373400	-2.35281100
O	1.49929100	1.54507700	-1.37519100
C	5.44208700	-2.08797200	3.07758900

C	4.15983300	-1.86823200	2.57118600	H	4.60482800	-2.18797400	-0.10207000
C	3.34444700	-2.94937600	2.20723300	C	6.43709800	-0.19922600	-2.14346200
C	5.91023100	-3.39284600	3.22019800	H	7.27602000	-0.24384500	-2.83425100
C	5.39704200	2.64636700	4.53483500	C	6.25736200	2.26546800	-2.71881300
C	6.56024700	2.88914300	3.81011200	C	7.72369000	2.55625800	-2.35925600
C	5.10457300	-4.46795000	2.85480900	H	7.83014800	2.74907000	-1.28615600
C	3.82280800	-4.24673100	2.34954000	H	8.37830000	1.71859100	-2.61916400
C	6.81293100	2.18509000	2.63417700	H	8.07789500	3.43990500	-2.90109500
C	0.06340300	1.23853700	4.17007000	C	6.14371400	2.00000000	-4.22871100
C	-0.93920900	2.36422300	3.97011700	H	5.10689100	1.78495700	-4.50777000
C	-0.55707100	4.16874400	-0.53414800	H	6.47435300	2.87880600	-4.79279600
C	0.62928300	4.43368800	-1.44467600	H	6.76038800	1.15022600	-4.53731500
C	3.12786500	0.00925100	0.54454200	C	5.41813600	3.50082300	-2.37801100
H	-1.51886100	2.72808200	0.69404700	H	5.78334800	4.36030400	-2.94873400
H	-1.68263600	1.42600000	-0.96242200	H	4.36208600	3.35553300	-2.62925700
H	1.34296600	-0.90727900	2.92107600	H	5.48413700	3.75387200	-1.31456400
H	2.21256400	-0.25093200	4.29646700	C	6.80418700	-2.67690200	-1.73711400
H	6.11280100	0.69100900	1.26140000	C	6.20017800	-3.84882300	-0.95759900
H	3.57483100	1.52762800	4.65782000	H	6.22893500	-3.67245700	0.12341600
H	6.07289200	-1.25110900	3.36031900	H	5.16010700	-4.03606800	-1.24604600
H	6.90605300	-3.56483000	3.61522400	H	6.77290900	-4.75858900	-1.16329200
H	5.20134500	3.18656200	5.45499400	C	6.76840800	-3.01711100	-3.23551000
H	7.27497400	3.62439100	4.16575600	H	7.23762000	-2.23498300	-3.83987000
H	5.47493000	-5.48246200	2.96273100	H	7.30562900	-3.95311000	-3.42341600
H	7.72476700	2.36643700	2.07450000	H	5.73743500	-3.13967000	-3.58357800
H	-0.43953100	3.33454400	3.89923900	C	8.26352400	-2.49868100	-1.28748800
H	-1.64590000	2.38516400	4.80325300	H	8.82654800	-3.42322500	-1.45598800
H	-1.50653900	2.20477300	3.04539100	H	8.76146900	-1.69570200	-1.83951700
H	-0.53984200	4.90185200	0.28647900	H	8.31343900	-2.25704400	-0.22024700
H	1.57151100	4.27420900	-0.91703600	O	-1.73592800	4.30146300	-1.30830000
H	0.58207500	5.46753400	-1.79999300	O	-0.57464800	-0.02084300	4.23940800
H	0.59353600	3.76952500	-2.31216100	H	-1.33716400	-0.02250300	3.64005400
H	2.35637000	-0.74770800	0.34124400	H	0.57450300	1.37331000	5.13360000
H	2.63758800	0.97934500	0.44809700	Si	-3.16609400	4.92695700	-0.69100900
H	-0.03096300	0.67679000	1.44316500	C	-3.46251400	4.43550900	1.10024800
H	1.81014800	2.11250000	3.25223700	H	-3.67256000	3.36493500	1.15191300
H	2.34692700	-2.77943800	1.80529900	H	-2.59053000	4.66835800	1.72426900
H	3.19567000	-5.08557800	2.06642400	H	-4.30922300	4.99521300	1.51465700
C	4.30392300	-0.08700500	-0.38915800	C	-2.93269700	6.80376800	-0.67331600
C	4.71560300	1.06501400	-1.05896500	H	-2.72884000	7.20770400	-1.67029900
C	4.95887200	-1.29850000	-0.61165900	H	-3.81084400	7.32352700	-0.27378400
C	5.78704700	1.02545000	-1.95314200	H	-2.08254900	7.06699400	-0.03213900
H	4.16783500	1.98408400	-0.88104100	C	-4.58044800	4.43123400	-1.87910200
C	6.04367800	-1.37111700	-1.48816800	C	-5.67766700	3.70016100	-1.09104700

H	-6.12569300	4.33826100	-0.31855900
H	-6.48849200	3.39072800	-1.76770500
H	-5.24964700	2.81137700	-0.61974400
C	-4.04283800	3.48257500	-2.96063500
H	-3.25411300	3.95081900	-3.55992600
H	-3.63729100	2.58512000	-2.48644300
H	-4.85840900	3.19801900	-3.64301700
C	-5.18501200	5.67026200	-2.55423200
H	-5.59984900	6.37960900	-1.82846400
H	-4.44924400	6.20930000	-3.16198200
H	-6.00454500	5.36992900	-3.22251100
C	0.81590100	-0.28166000	-3.37972800
C	1.99199900	-1.02866200	-2.76016500
H	2.55402300	-1.53993600	-3.54880400
H	2.66118500	-0.34226700	-2.24522600
H	1.63729200	-1.78975800	-2.05488900
C	-0.06363200	-1.24876900	-4.15921300
H	-0.43932700	-2.03696900	-3.49773400
H	-0.91878900	-0.72716700	-4.59700900
H	0.51403500	-1.71393600	-4.96297700
C	1.28149100	0.86447500	-4.27263000
H	1.95407500	1.53299900	-3.73259200
H	1.80959400	0.45890600	-5.14103000
H	0.41877900	1.43585000	-4.62957600
N	-3.07312600	-0.01703300	1.80771400
O	-3.22249200	-0.20974000	3.01434400
O	-1.99068100	0.24987500	1.32008700
C	-4.22368600	-0.11711600	0.95054000
C	-4.09596200	-0.10500800	-0.46312700
C	-5.45352500	-0.20064200	1.58365800
C	-5.29632900	-0.15080200	-1.20261000
C	-6.59088600	-0.28611700	0.80658300
C	-6.52548200	-0.23475100	-0.58750500
H	-7.43673900	-0.28458700	-1.17150600
N	-7.86828600	-0.49690200	1.46073900
O	-8.84952000	-0.67129900	0.75385700
O	-7.89691400	-0.50899600	2.68137800
H	-5.23852600	-0.12265300	-2.28235500
H	-5.51351400	-0.22274000	2.66372000
F	-3.33679600	1.89353200	-0.45496600
O	-2.91591500	-0.40475500	-1.09340400
O	0.12613900	-1.93222400	1.24648200
C	-2.90708600	-1.58450200	-1.82771300
C	-2.45462300	-2.72463800	-1.20470900
C	-1.91403800	-2.62502900	0.17710400
C	-2.53339700	-2.91945200	1.33893300
H	-1.93392600	-2.73873000	2.23327700
C	-3.93173200	-3.27188500	1.60231400
C	-4.89437100	-3.51905400	0.60759600
H	-4.60408900	-3.59077900	-0.43396300
C	-6.23539800	-3.65601400	0.94833400
H	-6.97083500	-3.82735900	0.16812300
C	-6.64245800	-3.55584200	2.27912100
H	-7.69396400	-3.64472200	2.53519100
C	-3.30157100	-1.56935000	-3.18451400
H	-3.58964300	-0.62688100	-3.63845000
C	-3.27334500	-2.73404400	-3.90333300
H	-3.56968500	-2.73998400	-4.94842900
C	-2.83683800	-3.94781500	-3.30736800
C	-2.40661800	-3.94607100	-1.94880200
C	-5.69515000	-3.33624600	3.27710500
H	-6.00282400	-3.25803100	4.31495100
C	-4.35366500	-3.19816100	2.93978400
H	-3.61782600	-2.99755100	3.71370700
C	-0.57822100	-1.97249200	0.25220100
H	-0.26323000	-1.47819800	-0.68614600
C	-1.96160000	-5.16527800	-1.37073300
H	-1.64250500	-5.17101900	-0.33331000
C	-1.94246700	-6.32235500	-2.10854100
H	-1.60141500	-7.24711000	-1.65369300
C	-2.36446200	-6.32306900	-3.45959200
H	-2.34267700	-7.24640000	-4.02948200
C	-2.80135700	-5.16168000	-4.04325100
H	-3.12828600	-5.14937400	-5.07940100

II-IM3-*R*

P	-3.08829700	-1.23257500	2.02742900
N	-0.28217200	-2.61872800	1.58473600
C	0.04286800	-3.74604400	0.87514400
O	-0.43087800	-4.84445000	1.13030400
C	1.14653800	-3.52998400	-0.15587000
N	1.01916500	-2.31774800	-0.92812600
C	-1.72931300	-1.49646500	3.20565600
C	-0.87606500	-2.72957000	2.90729500
C	-3.93666200	-2.79636900	1.73427900
C	-0.11650100	-2.02362000	-1.55483900
C	-3.48664900	-3.63496200	0.70600100
C	-4.96185200	-3.21007800	2.59371500

O	-0.01055700	-0.85113000	-2.23921200
O	-1.16834600	-2.67650700	-1.50707900
C	-5.54660200	0.04970200	2.32861200
C	-4.21309900	-0.01885900	2.75100300
C	-3.69225200	0.96441200	3.59819700
C	-6.35503300	1.09307600	2.76432700
C	-5.55667800	-4.45276400	2.40444500
C	-5.12329600	-5.28280800	1.37154600
C	-5.83812700	2.06795200	3.61729400
C	-4.51095500	2.00344700	4.03367700
C	-4.08950600	-4.87807800	0.53098700
C	0.19251600	-2.85635500	4.01541000
C	1.12767900	-4.03717500	3.80574100
C	1.46501300	-4.78760500	-0.98755500
C	0.34414600	-5.27150300	-1.89192400
C	-2.48506300	-0.53449400	0.45141400
H	2.03967300	-3.35581600	0.46205500
H	-1.09691800	-0.60076900	3.19842900
H	-2.18416100	-1.58172100	4.19877000
H	-2.67143400	-3.34065300	0.04706100
H	-5.29900100	-2.56293300	3.39857500
H	-5.94657800	-0.69666500	1.64854700
H	-7.38571000	1.14890100	2.42926500
H	-6.35766600	-4.77203000	3.06294800
H	-5.59055400	-6.25173900	1.22624100
H	-6.47159500	2.88316600	3.95310600
H	-3.74077600	-5.52937000	-0.26363900
H	0.57557200	-4.97437300	3.70351600
H	1.81470800	-4.11327800	4.65219600
H	1.72528400	-3.89329400	2.89780400
H	1.71306200	-5.58382900	-0.26784900
H	-0.57344800	-5.43827100	-1.32543300
H	0.65163000	-6.20519400	-2.37237500
H	0.15206700	-4.52804800	-2.66920900
H	-1.63485500	0.09514200	0.73090800
H	-2.12272700	-1.35562000	-0.17647100
H	0.27469700	-1.78880300	1.38959700
H	-1.49015100	-3.63510300	2.90393300
H	-2.65213000	0.93833900	3.90727500
H	-4.10657200	2.76377800	4.69352100
C	-3.57706800	0.26399200	-0.20987800
C	-4.58695700	-0.37846800	-0.92496400
C	-3.60190700	1.64724400	-0.05264900
C	-5.62969200	0.35443300	-1.49615000
H	-4.54087500	-1.45821800	-1.03189800
C	-4.61138300	2.41532100	-0.63524600
H	-2.82697600	2.10856200	0.54858800
C	-5.61358800	1.74640200	-1.34552400
H	-6.40981500	2.32965700	-1.80206800
C	-6.76920800	-0.31438800	-2.27100400
C	-8.11116100	0.04003200	-1.60990700
H	-8.14119900	-0.30969700	-0.57216000
H	-8.29018300	1.11952800	-1.60776900
H	-8.93554300	-0.43524000	-2.15248800
C	-6.76727600	0.19652900	-3.72078400
H	-5.81824400	-0.03620700	-4.21453600
H	-7.57471700	-0.27726700	-4.28974400
H	-6.91491800	1.27987700	-3.76597000
C	-6.62860900	-1.83964300	-2.29257900
H	-7.46998000	-2.27732000	-2.83881100
H	-5.70698000	-2.15380800	-2.79358800
H	-6.63001100	-2.26251400	-1.28144000
C	-4.63146300	3.94262500	-0.52581600
C	-3.48176800	4.46370500	0.34273300
H	-3.53730500	4.07474500	1.36543700
H	-2.50799200	4.18858700	-0.07769900
H	-3.52438500	5.55678600	0.39274000
C	-4.48685200	4.54710100	-1.93218900
H	-5.30010900	4.23010000	-2.59246200
H	-4.50301700	5.64137600	-1.87761400
H	-3.53950400	4.24316500	-2.39017700
C	-5.95993200	4.39960100	0.09734700
H	-5.97775700	5.49091800	0.19075900
H	-6.81888100	4.10267300	-0.51226400
H	-6.08883100	3.96738100	1.09544100
O	2.57232900	-4.49636600	-1.82056300
O	0.91917200	-1.64672300	4.13715200
H	1.57451500	-1.63074000	3.42142600
H	-0.34461300	-2.98409300	4.96402200
Si	4.19901400	-4.48147600	-1.42477300
C	4.52387300	-3.85491300	0.31437900
H	4.19059800	-2.81626500	0.38890800
H	4.00890800	-4.46221400	1.06888700
H	5.59579500	-3.90348600	0.53977400
C	4.80289000	-6.26808800	-1.48000700
H	4.65481600	-6.73176600	-2.46038100
H	5.86751000	-6.34017000	-1.22974300
H	4.25665800	-6.86846500	-0.74266100

C	5.04845500	-3.41878900	-2.76818900
C	5.95457700	-2.37552400	-2.09724000
H	6.73033400	-2.83854100	-1.47429800
H	6.46848700	-1.77504600	-2.86219200
H	5.35198700	-1.70560400	-1.47625600
C	3.99354000	-2.67602000	-3.60298700
H	3.31413900	-3.37211600	-4.10593200
H	3.40416900	-2.01044600	-2.96555400
H	4.49309300	-2.07370000	-4.37669200
C	5.89117300	-4.30024800	-3.70092400
H	6.68386100	-4.83615500	-3.16552100
H	5.27996400	-5.04663200	-4.22153400
H	6.37501500	-3.68041400	-4.46895700
C	-0.70772700	-0.70122000	-3.50365200
C	-2.19021900	-0.43592700	-3.27760000
H	-2.68203900	-0.24330900	-4.23715100
H	-2.66517700	-1.29284900	-2.79865700
H	-2.32500500	0.44496000	-2.64152400
C	-0.04271500	0.52359600	-4.11835500
H	-0.15988100	1.38701300	-3.45577100
H	1.02532900	0.34207000	-4.26780100
H	-0.50118200	0.76059200	-5.08296600
C	-0.47709400	-1.93313100	-4.37430500
H	-1.01322600	-2.79831300	-3.97837200
H	-0.83144200	-1.74099600	-5.39138100
H	0.59178500	-2.16679200	-4.41707700
N	2.59857000	-0.41722700	1.36064800
O	2.87931300	-1.42266900	2.00322500
O	1.45699900	-0.07488300	1.12194100
C	3.69558300	0.46281900	0.98249200
C	3.60069700	1.33398600	-0.11714200
C	4.80049500	0.46003500	1.81501600
C	4.66591300	2.20588000	-0.36614100
C	5.81413500	1.36741800	1.55422400
C	5.76651200	2.23747400	0.47110500
H	6.57740500	2.93372500	0.29698100
N	6.92990400	1.45639100	2.48734100
O	7.75192500	2.33961500	2.30730700
O	6.97429600	0.65597000	3.40637600
H	4.61193200	2.87670800	-1.21559100
H	4.85198500	-0.21200100	2.66204500
F	3.22628500	-1.34395500	-0.85594500
O	2.51049700	1.29623300	-0.89887500
O	-0.76126400	2.00755500	1.77494000
C	2.12648800	2.45954100	-1.54958100
C	1.37158000	3.37883300	-0.85904700
C	0.99253200	3.09182100	0.55256700
C	1.68690400	3.38161500	1.67008900
H	1.26797900	2.97394300	2.59083600
C	2.96291300	4.08789100	1.81777700
C	3.42527200	5.06039600	0.91775300
H	2.80036800	5.37157500	0.08719100
C	4.67454900	5.64265300	1.09881500
H	5.02105200	6.39823700	0.40070900
C	5.48298100	5.26112100	2.17086900
H	6.46297400	5.71005300	2.30040500
C	2.45737000	2.60525200	-2.91361200
H	3.06206800	1.83827700	-3.38674900
C	1.97950100	3.68945400	-3.60027800
H	2.21029800	3.81674500	-4.65398700
C	1.15607300	4.65281500	-2.95645600
C	0.84796300	4.50092500	-1.57346400
C	5.02566200	4.31109000	3.08186200
H	5.64673000	4.01710300	3.92274400
C	3.76861000	3.74005100	2.91245400
H	3.40709400	2.99942900	3.62146200
C	-0.22232000	2.24920100	0.71120000
H	-0.61971600	1.82778400	-0.23504500
C	0.01932300	5.46870800	-0.94389000
H	-0.20279800	5.36421400	0.11440600
C	-0.49542000	6.51989000	-1.66153200
H	-1.13473100	7.24738800	-1.17086100
C	-0.19627800	6.66653400	-3.03699500
H	-0.61095400	7.50307500	-3.59020700
C	0.61574900	5.75719700	-3.66603100
H	0.85397800	5.86222500	-4.72091800
H	1.99760100	-1.74904400	-1.011220

11. NMR spectra

NMR of **1b** ($CDCl_3$)

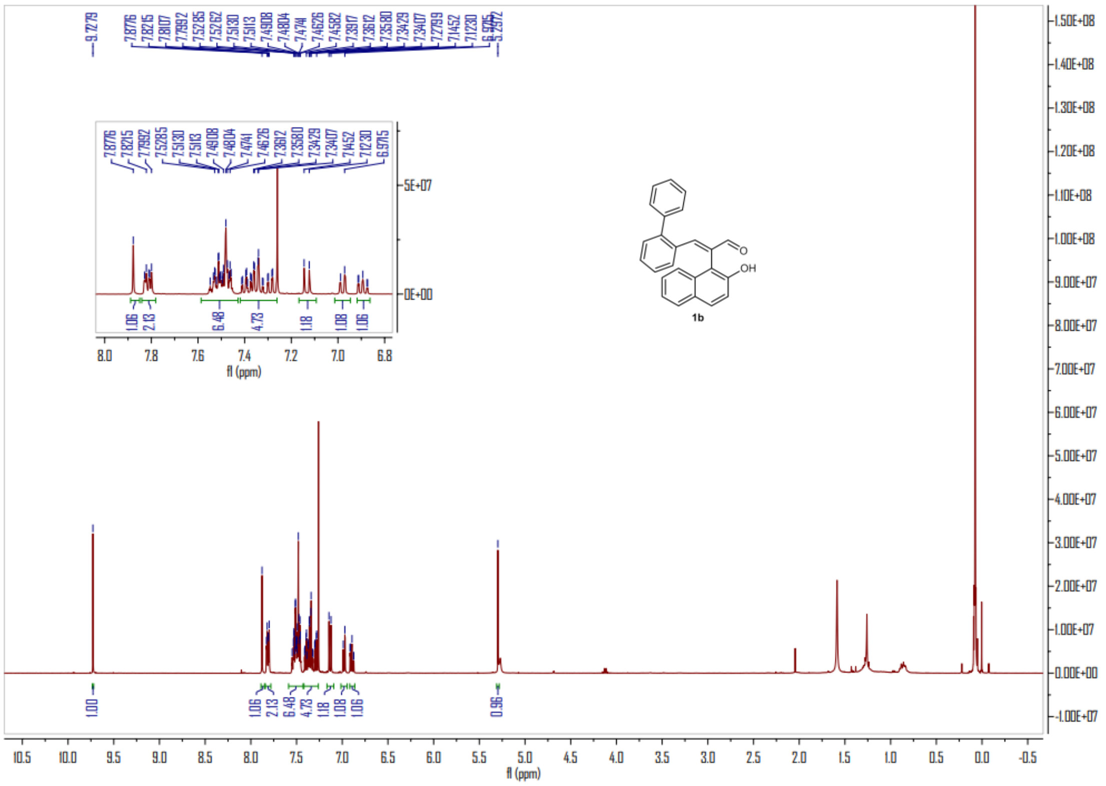

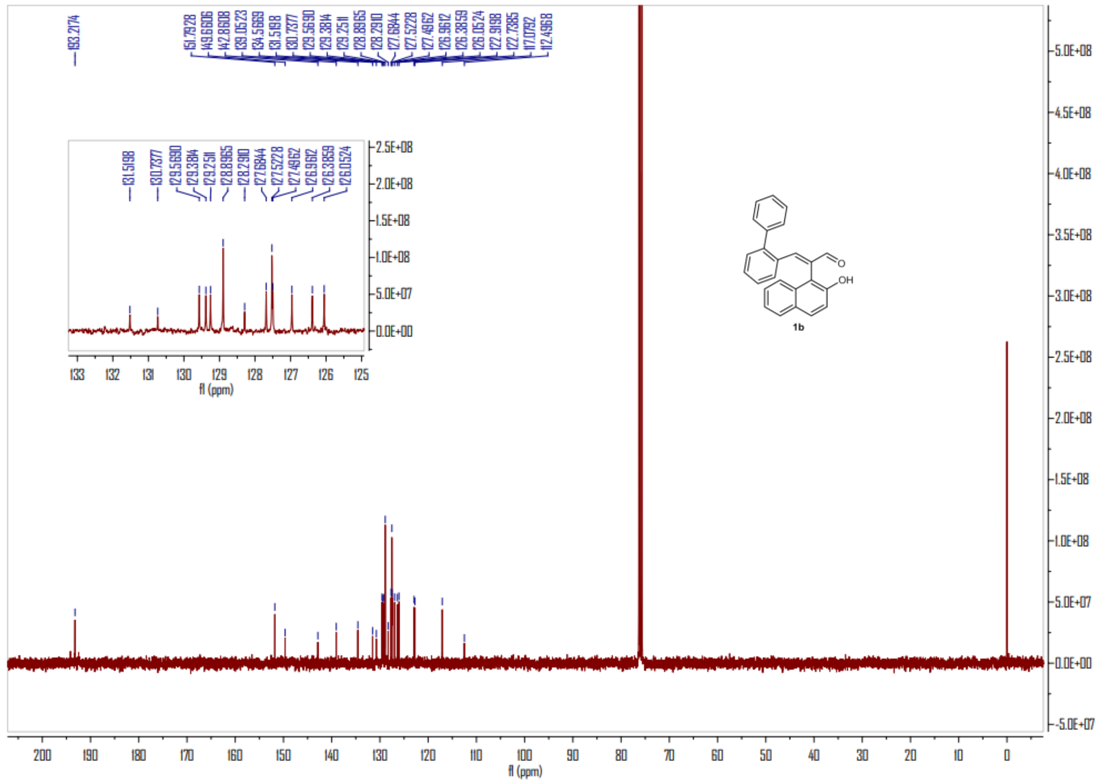

NMR of **1c** ($CDCl_3$)

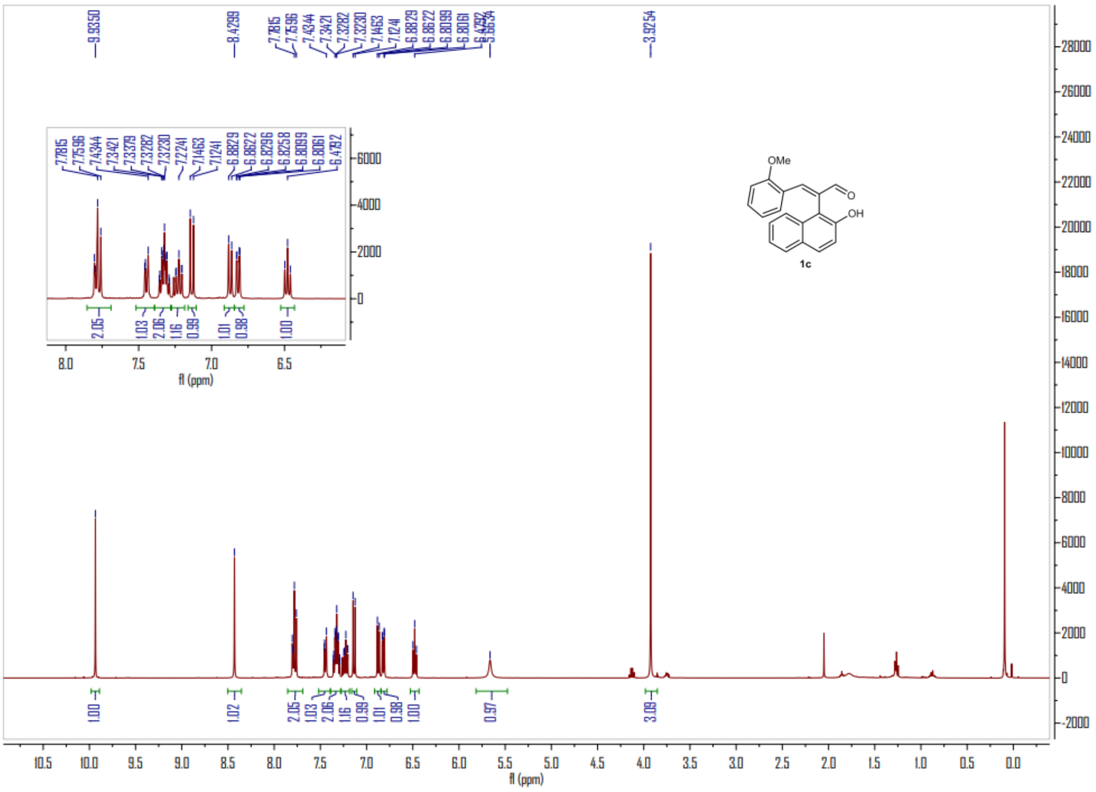

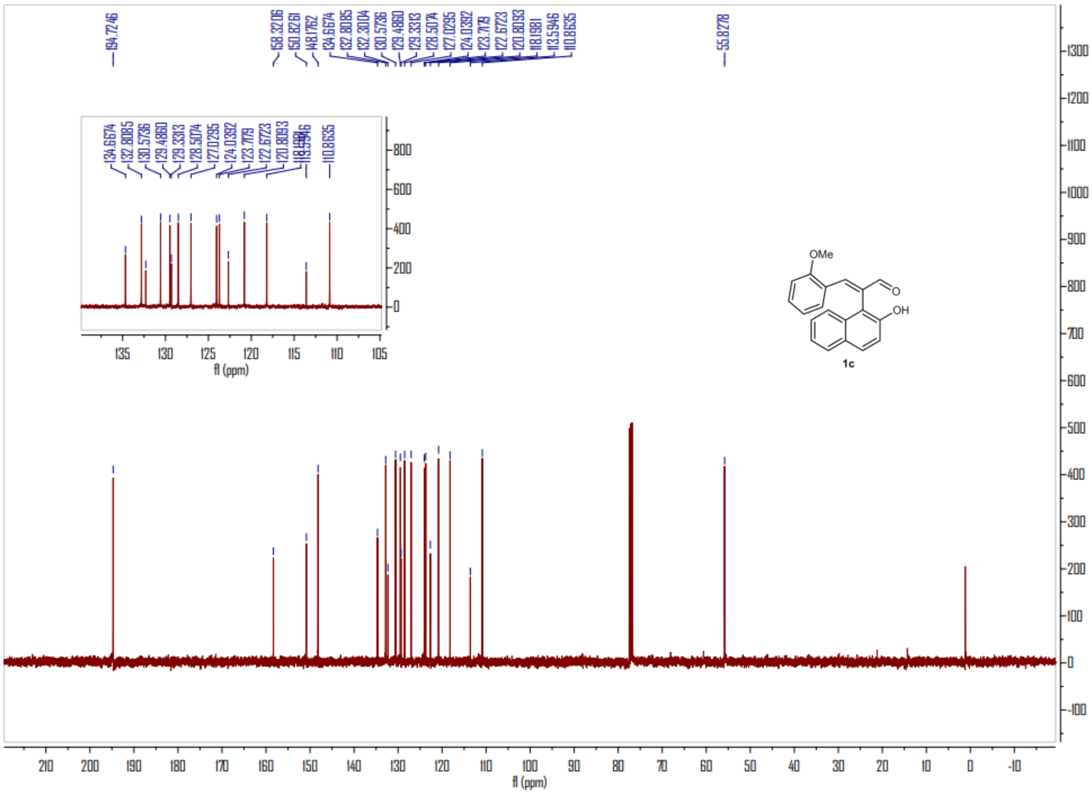

NMR of **1d** ($CDCl_3$)

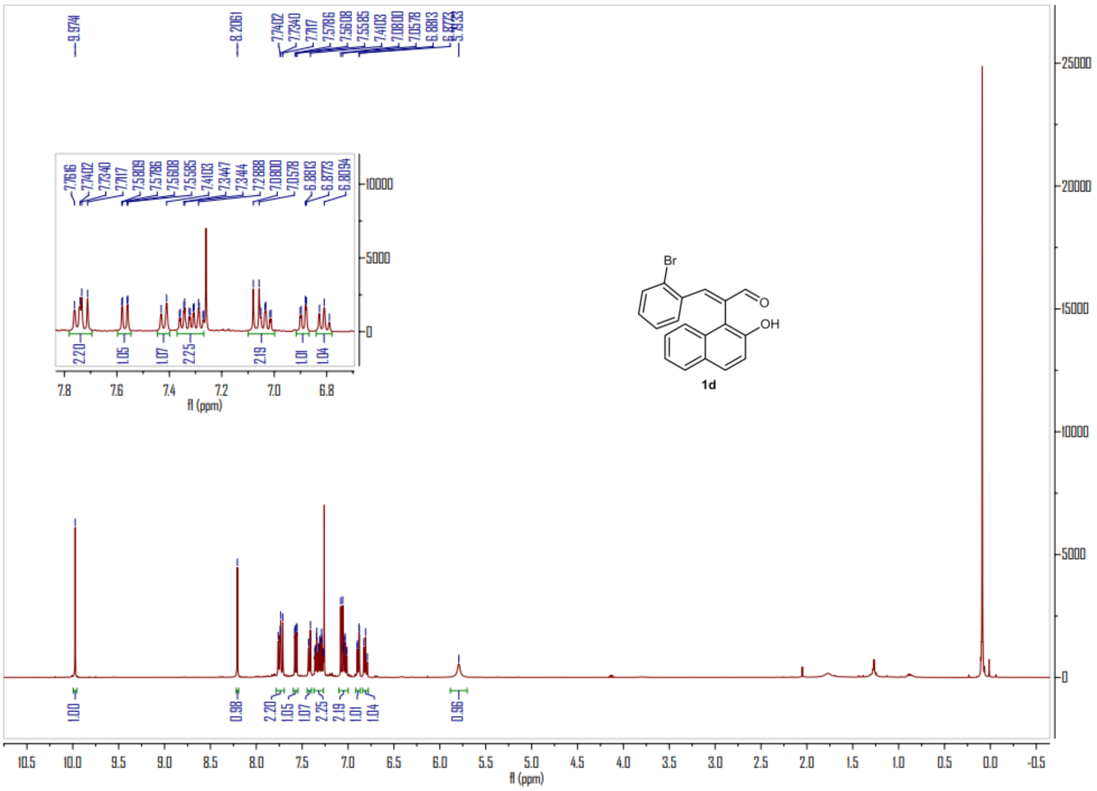

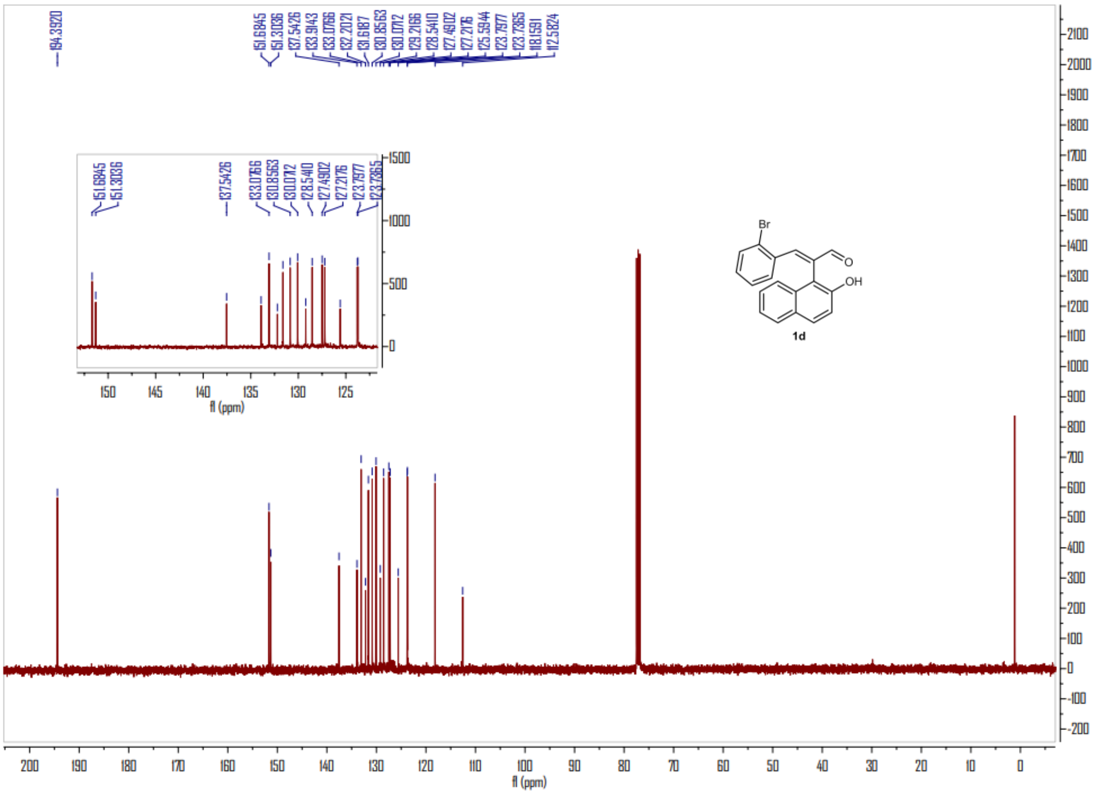

NMR of **1f** $CDCl_3$)

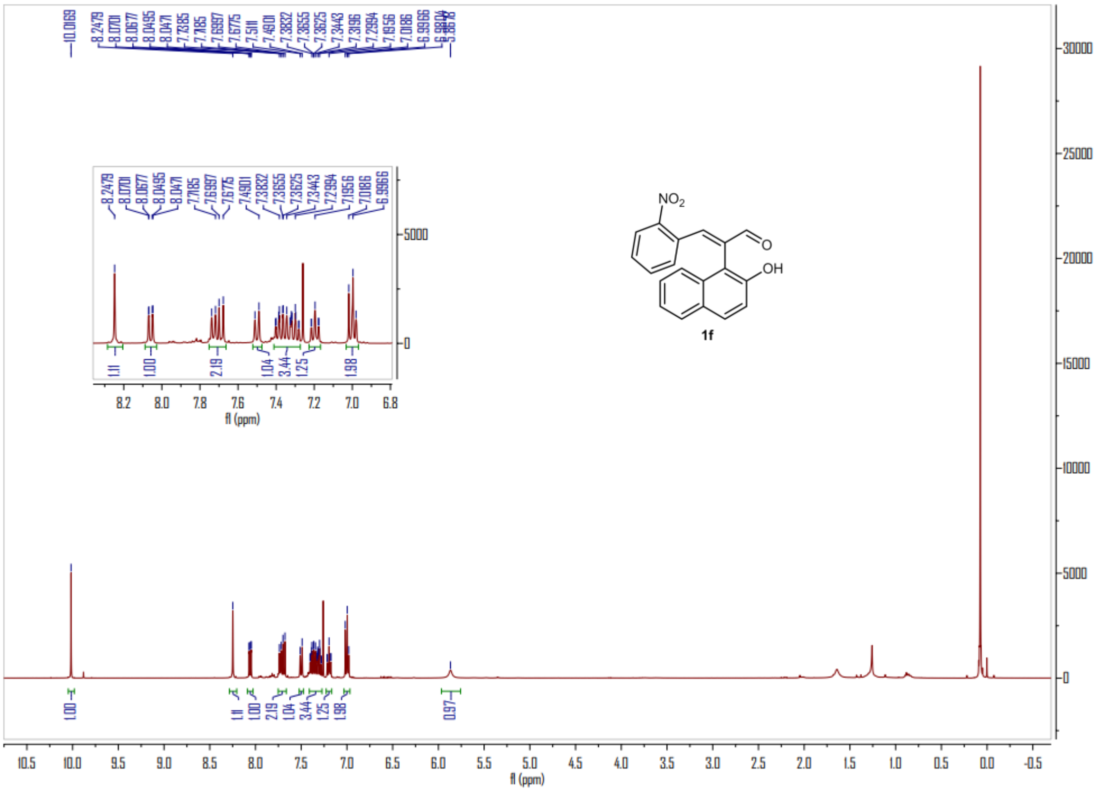

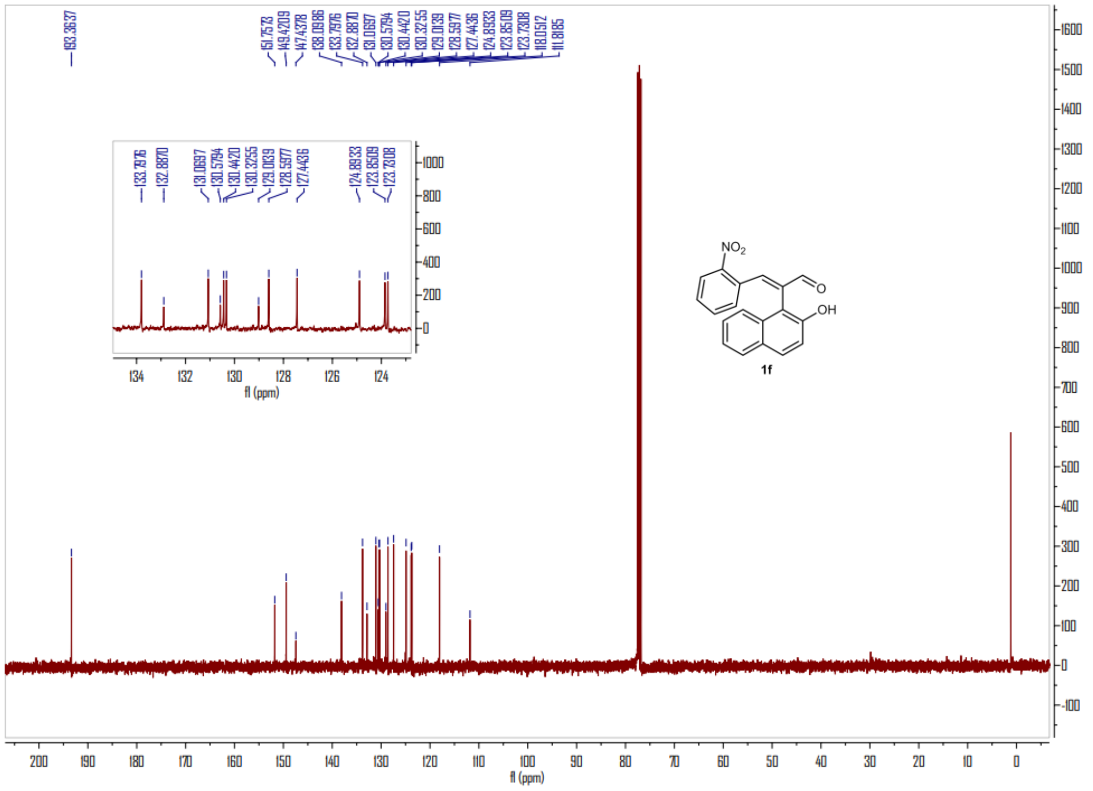

NMR of **1g** ($CDCl_3$)

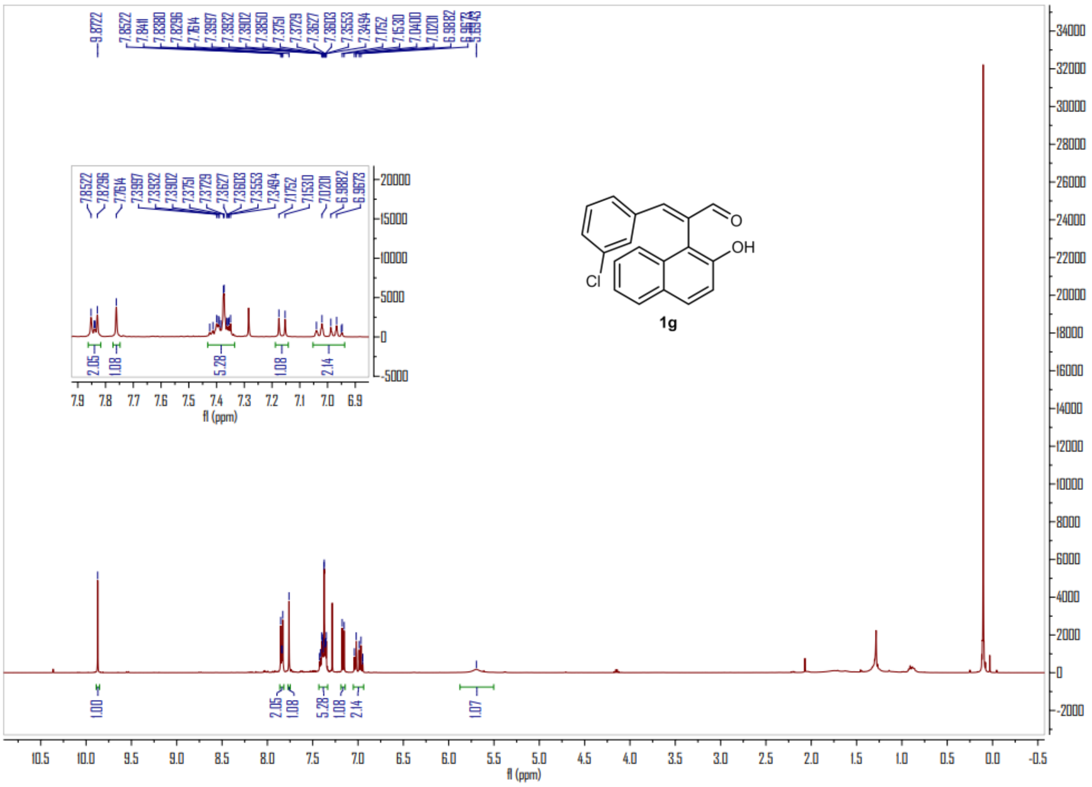

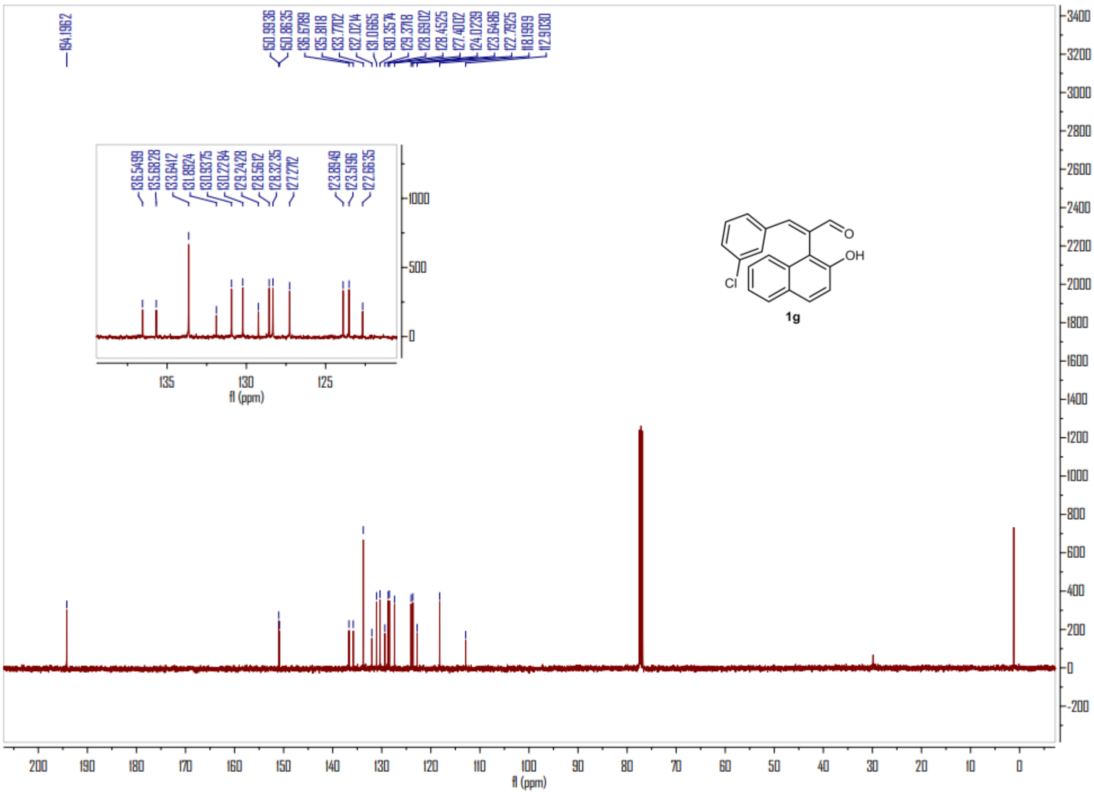

NMR of **1n** ($CDCl_3$)

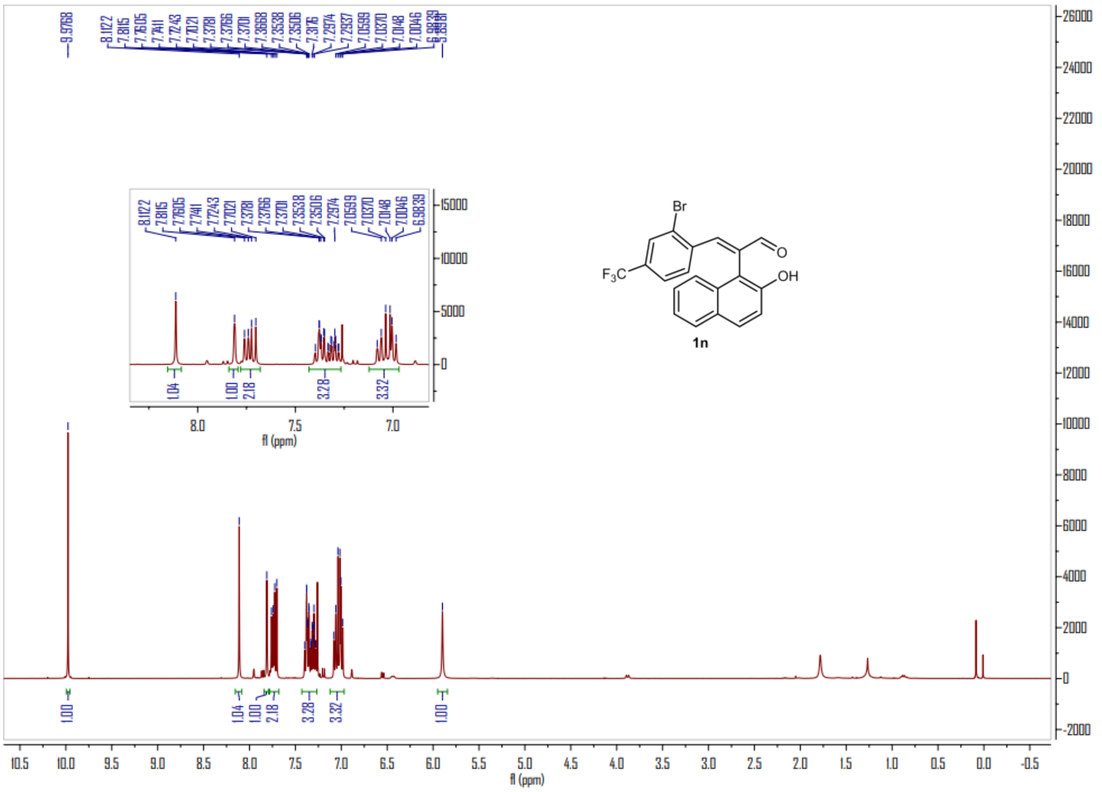

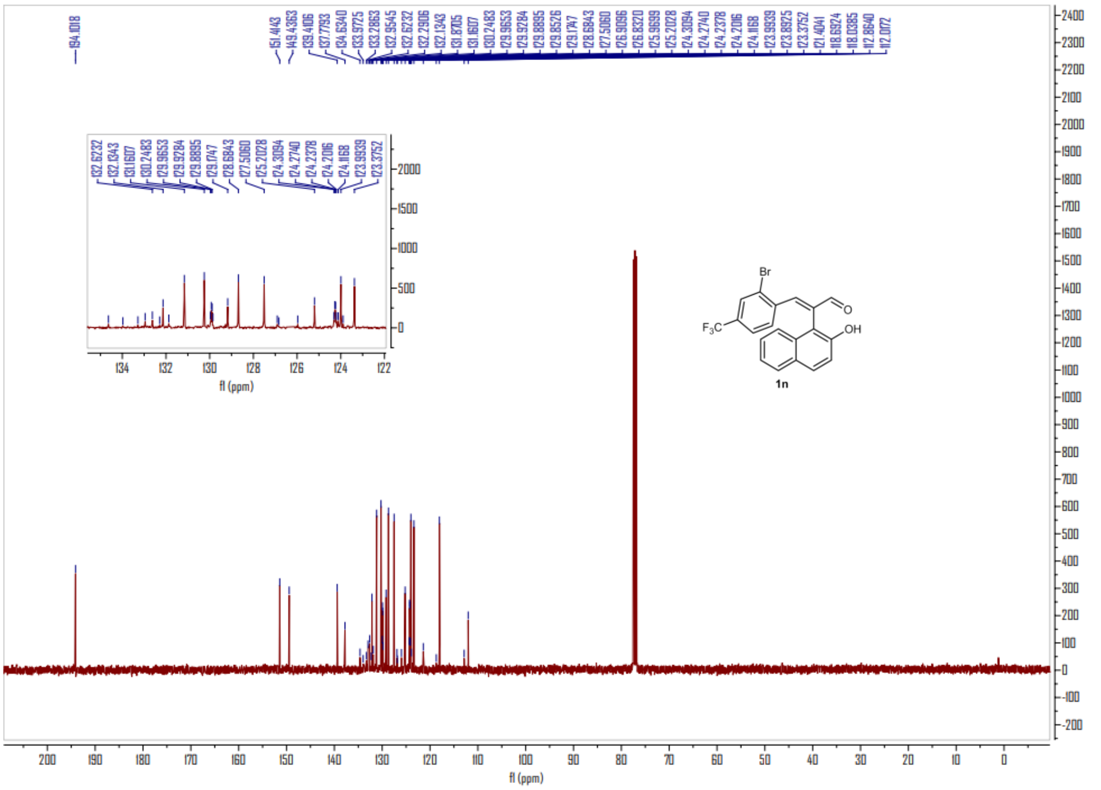

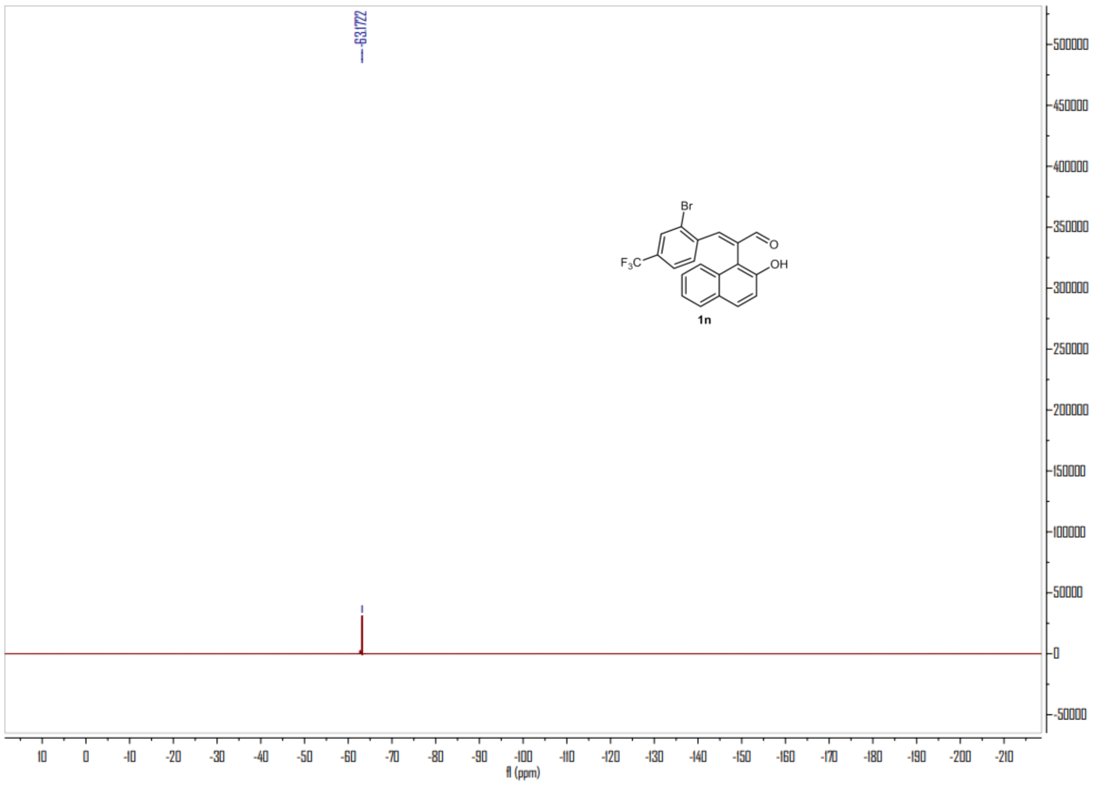

NMR of **1o** ($CDCl_3$)

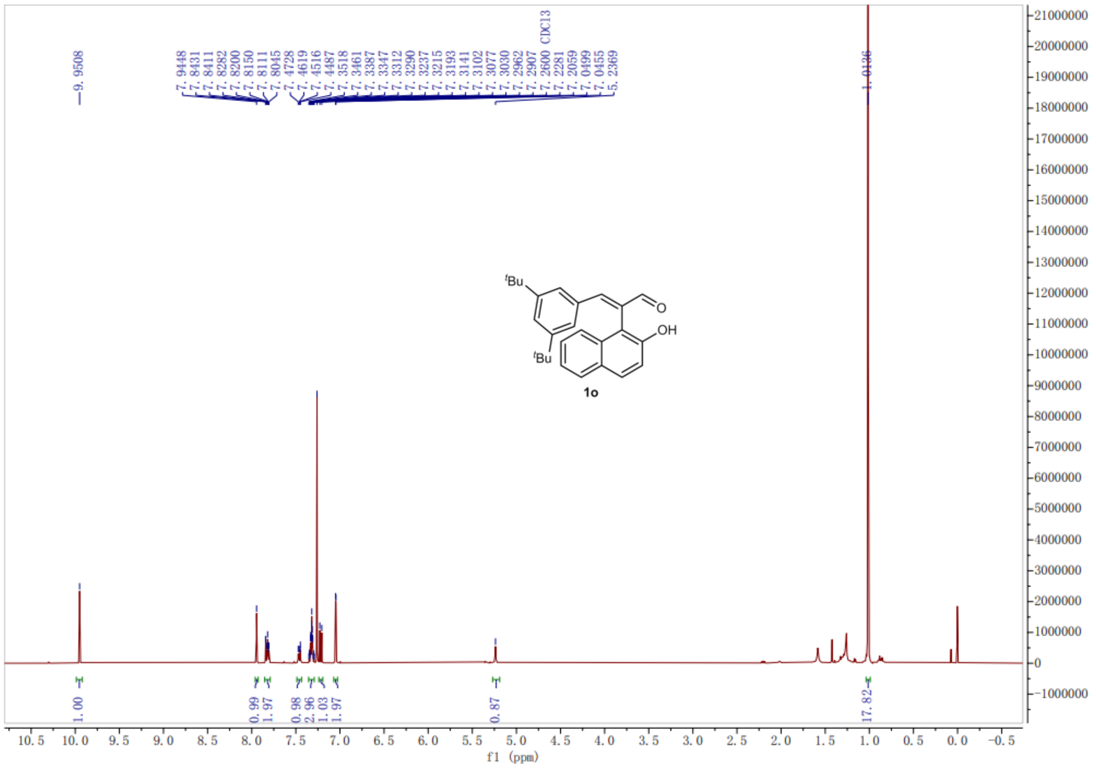

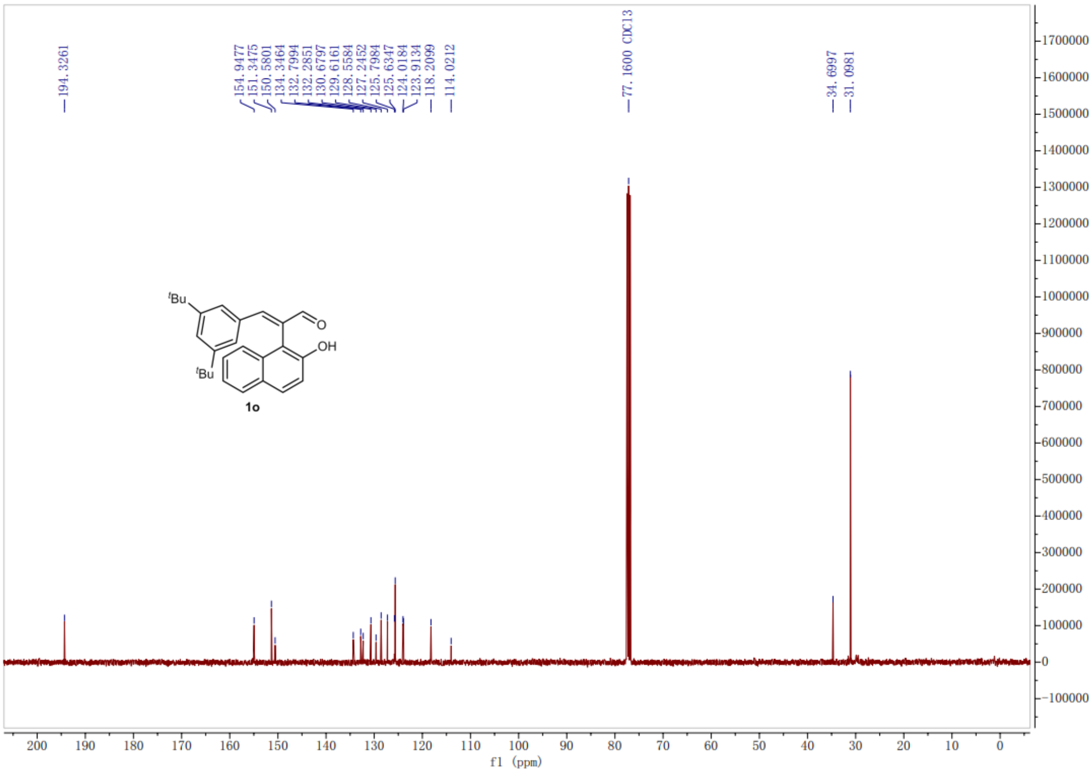

NMR of **1q** ($CDCl_3$)

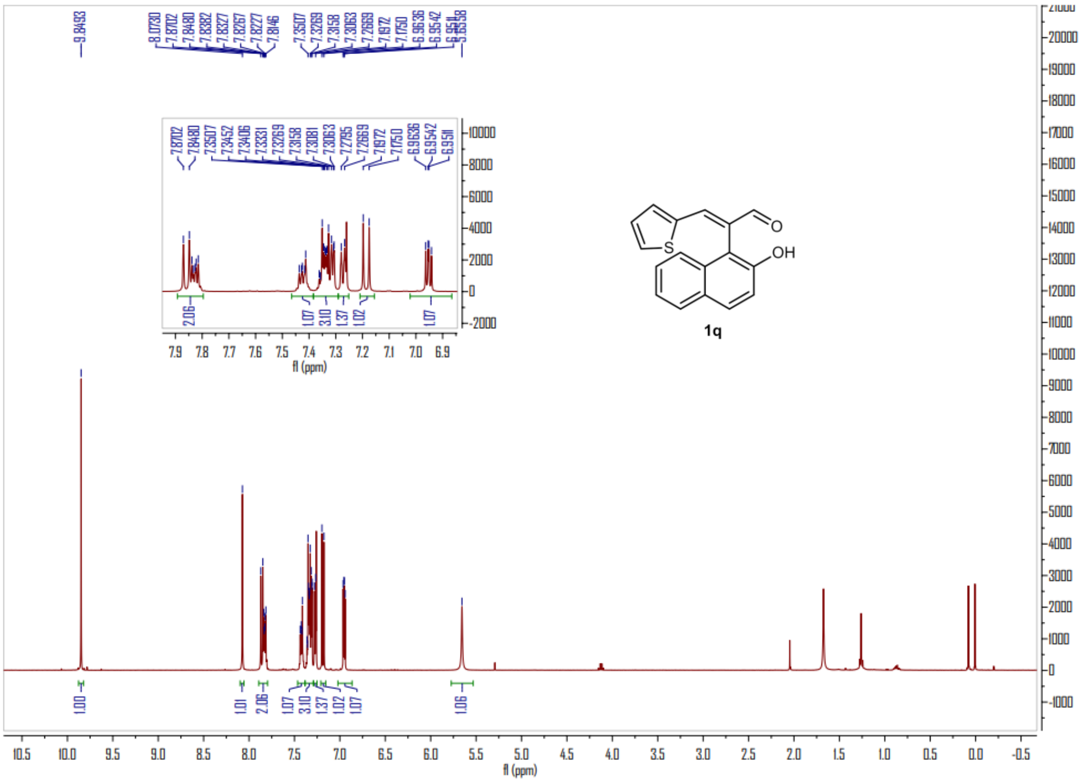

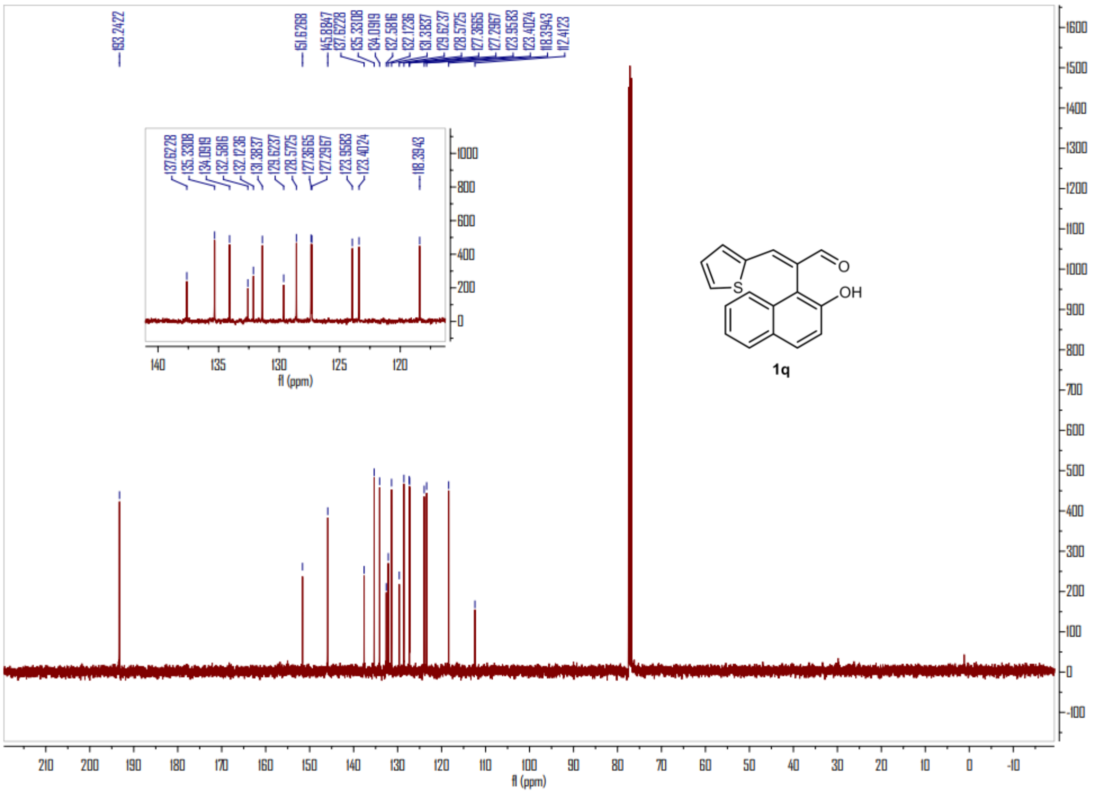

NMR of **1r** ($CDCl_3$)

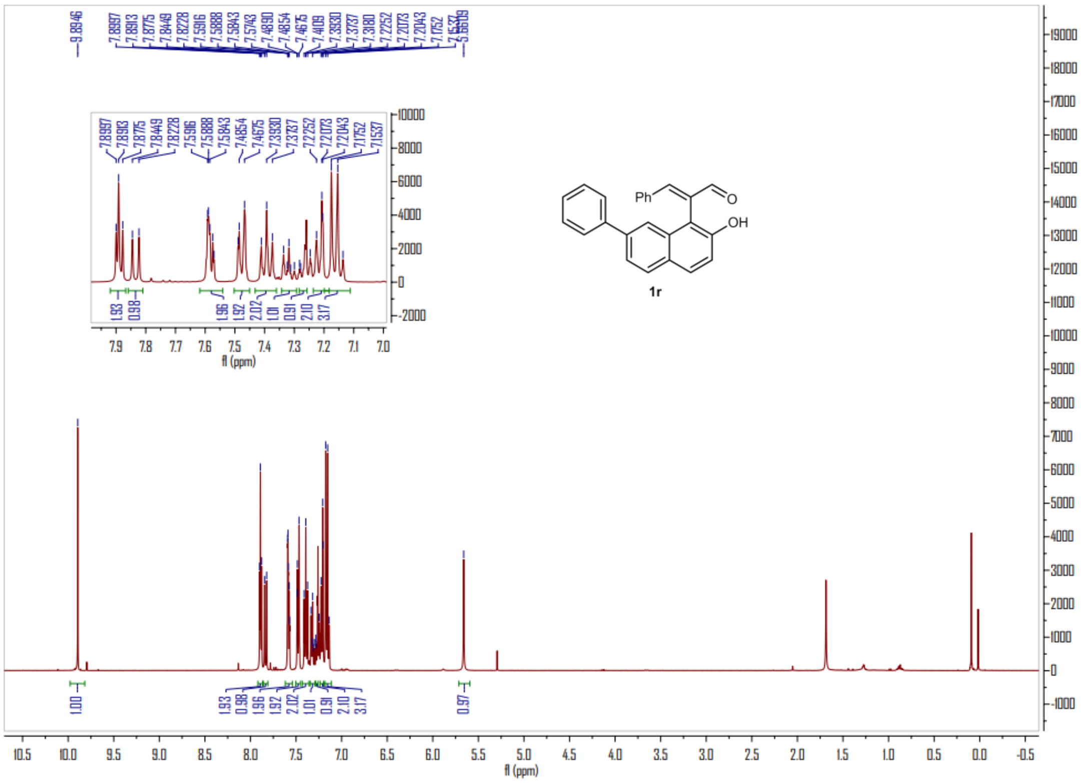

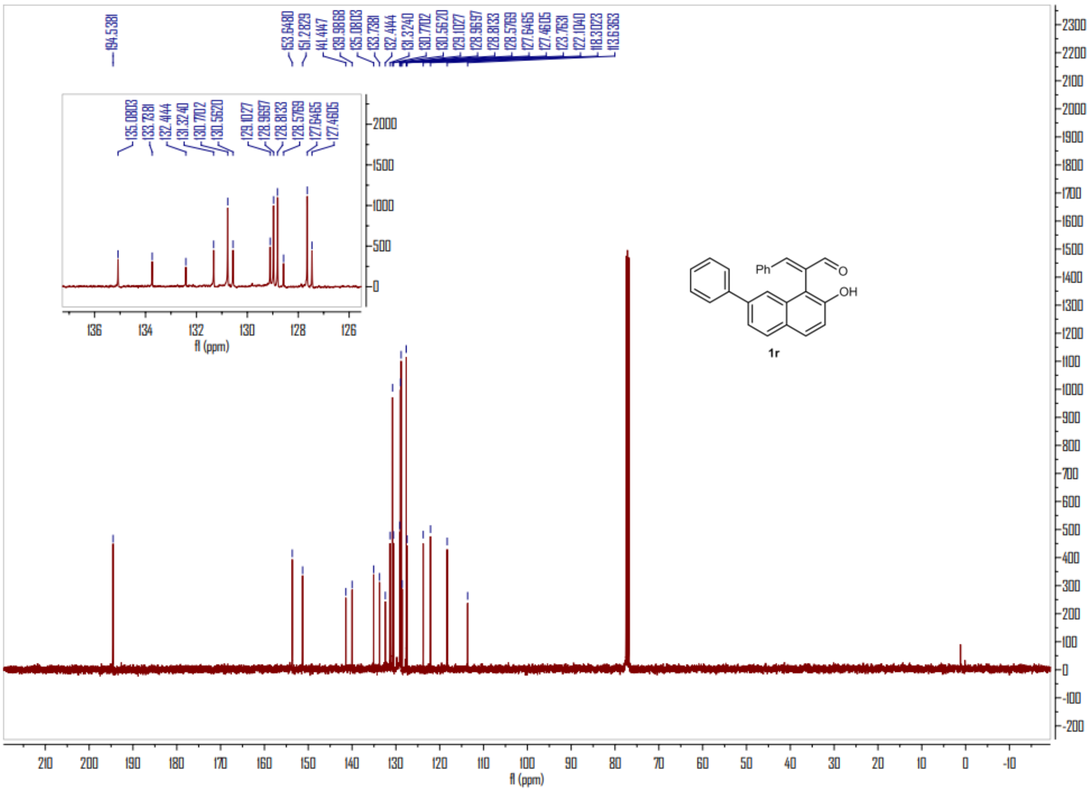

NMR of **1t** ($CDCl_3$)

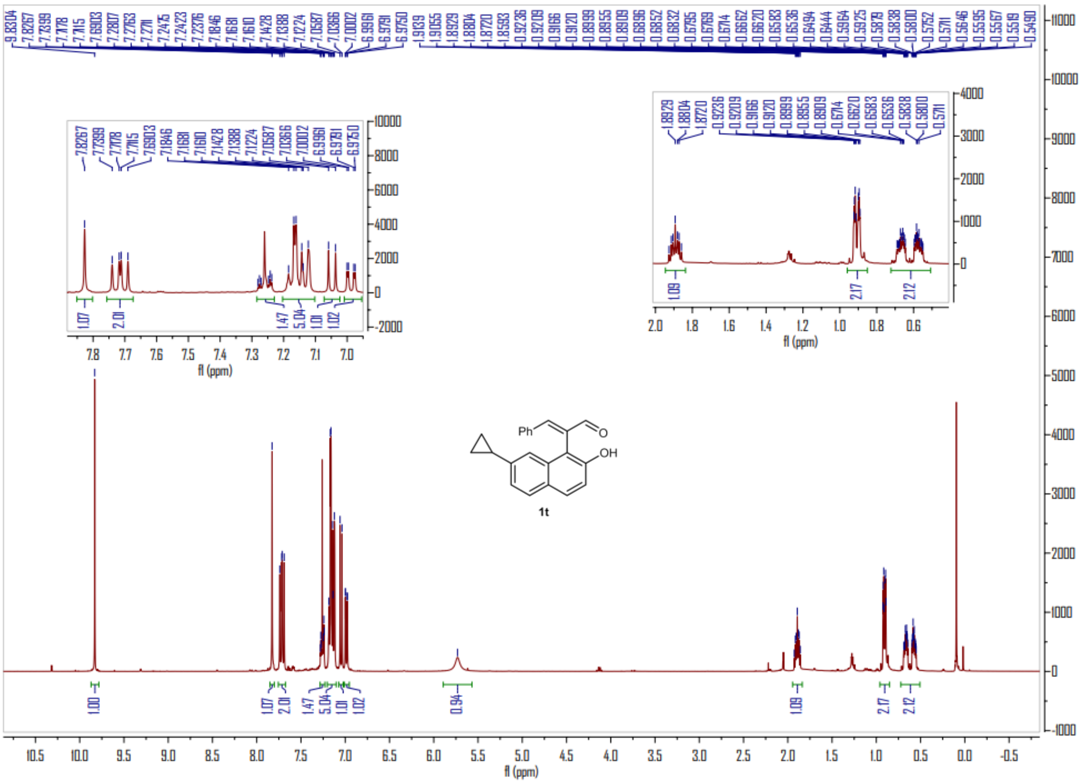

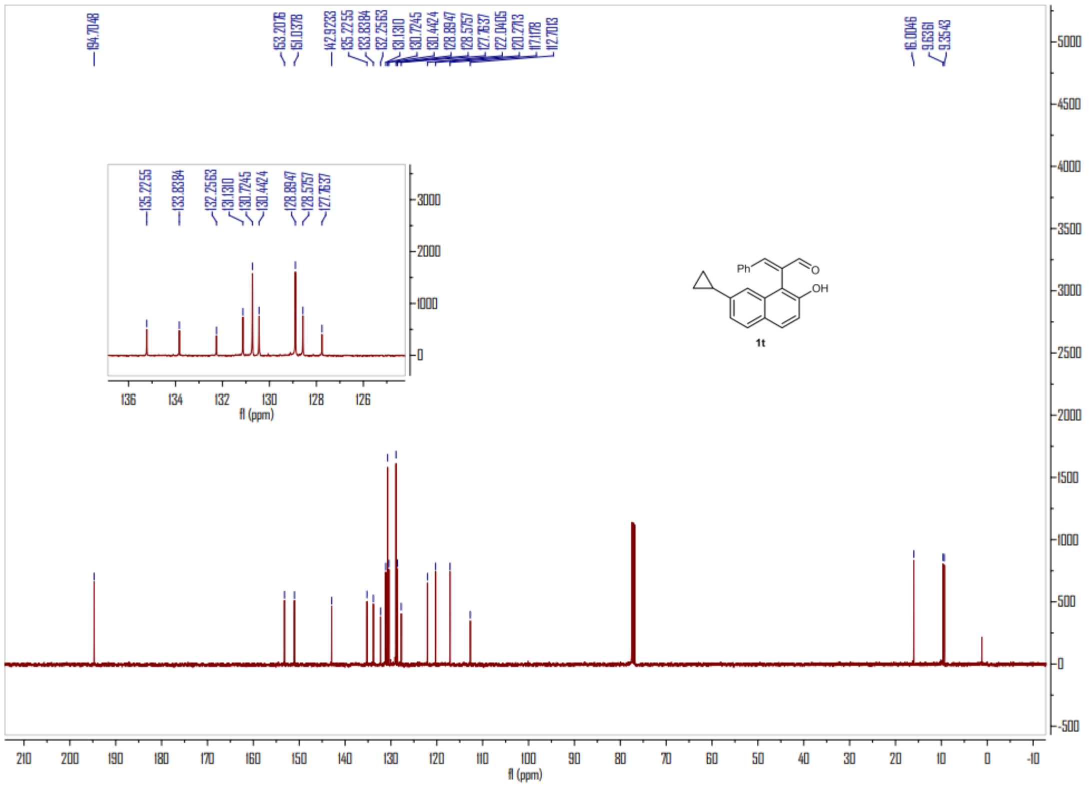

NMR of **1u** ($CDCl_3$)

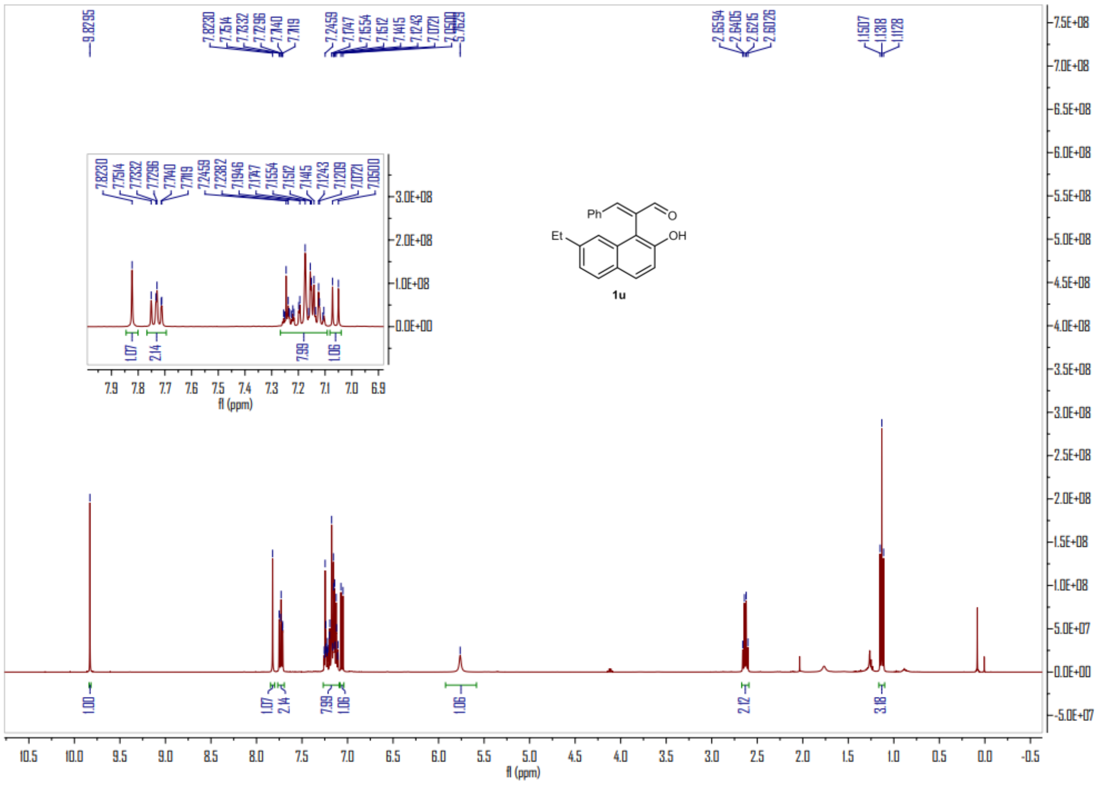

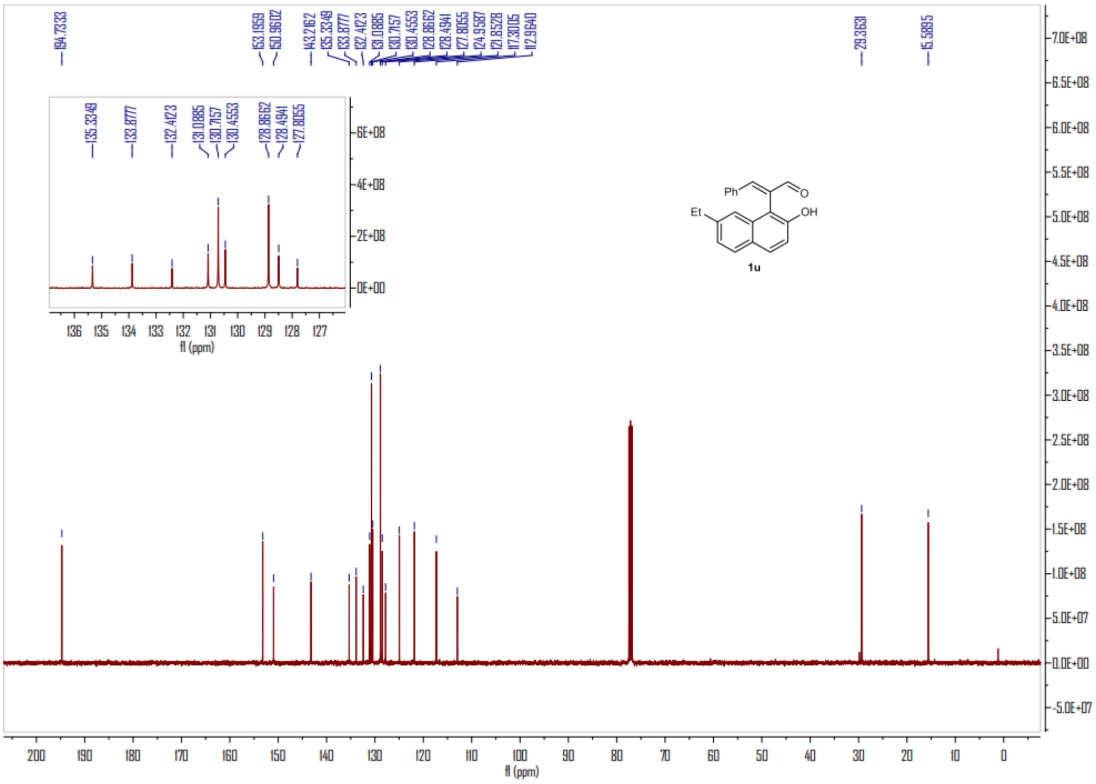

NMR of **1v** ($CDCl_3$)

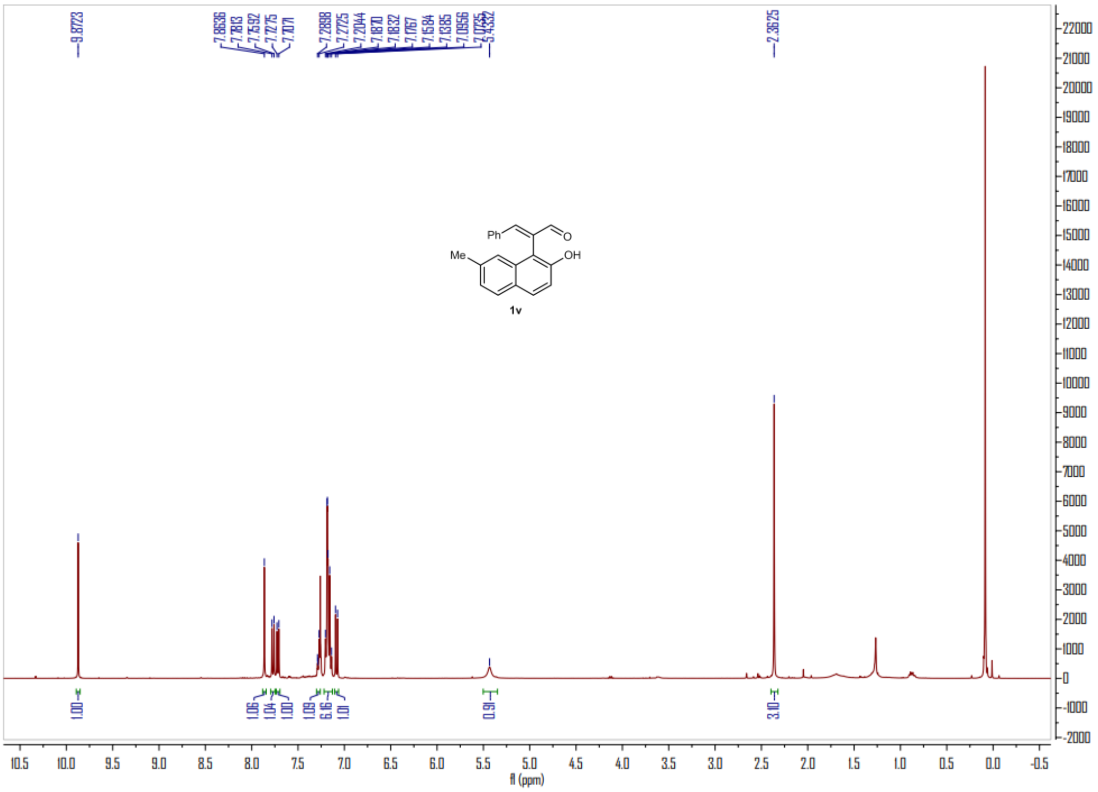

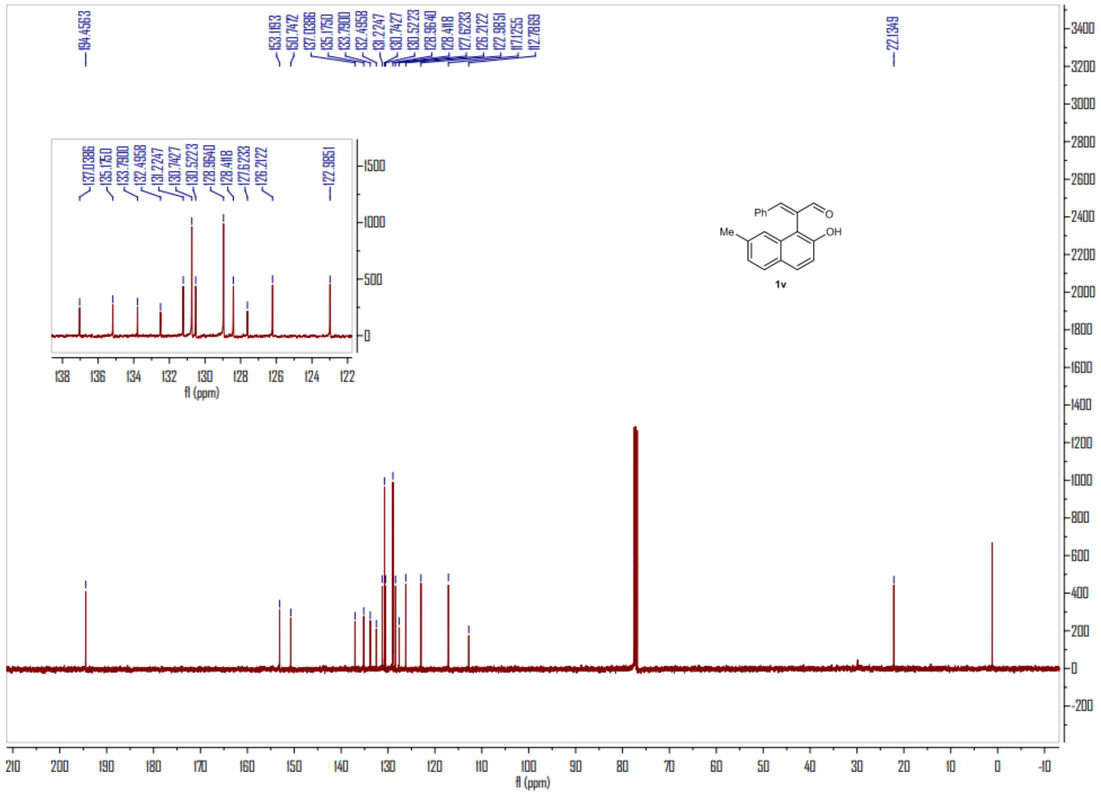

NMR of **1x** ($CDCl_3$)

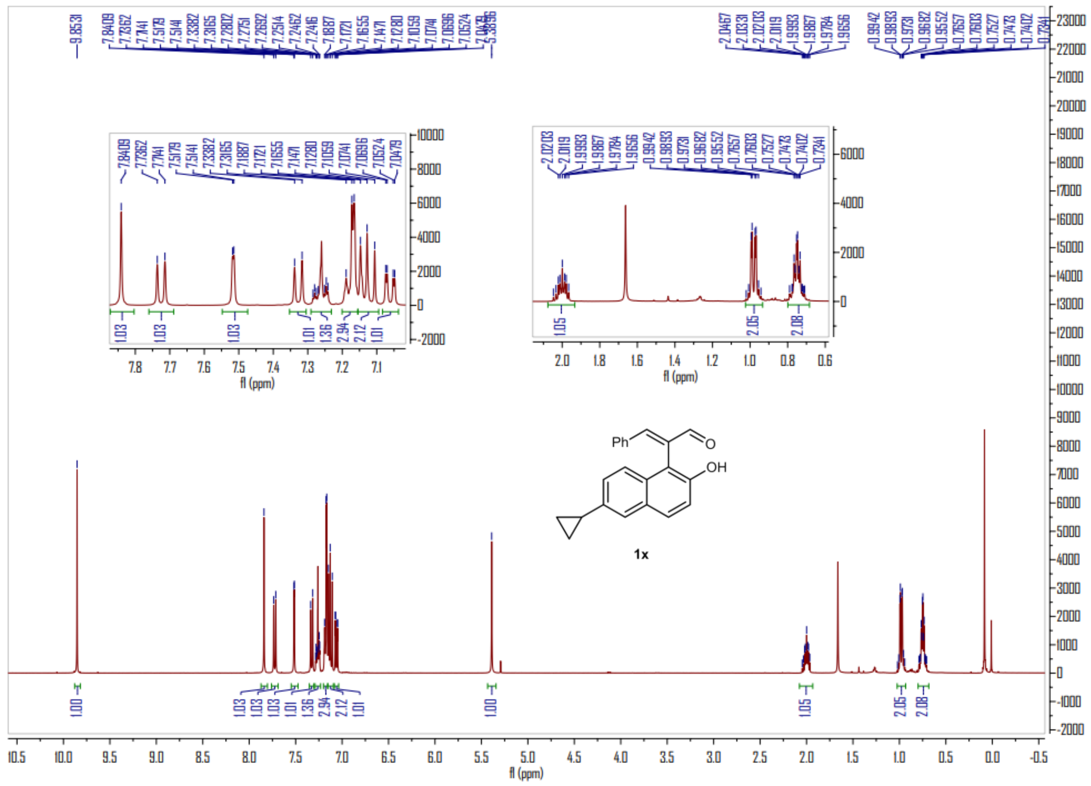

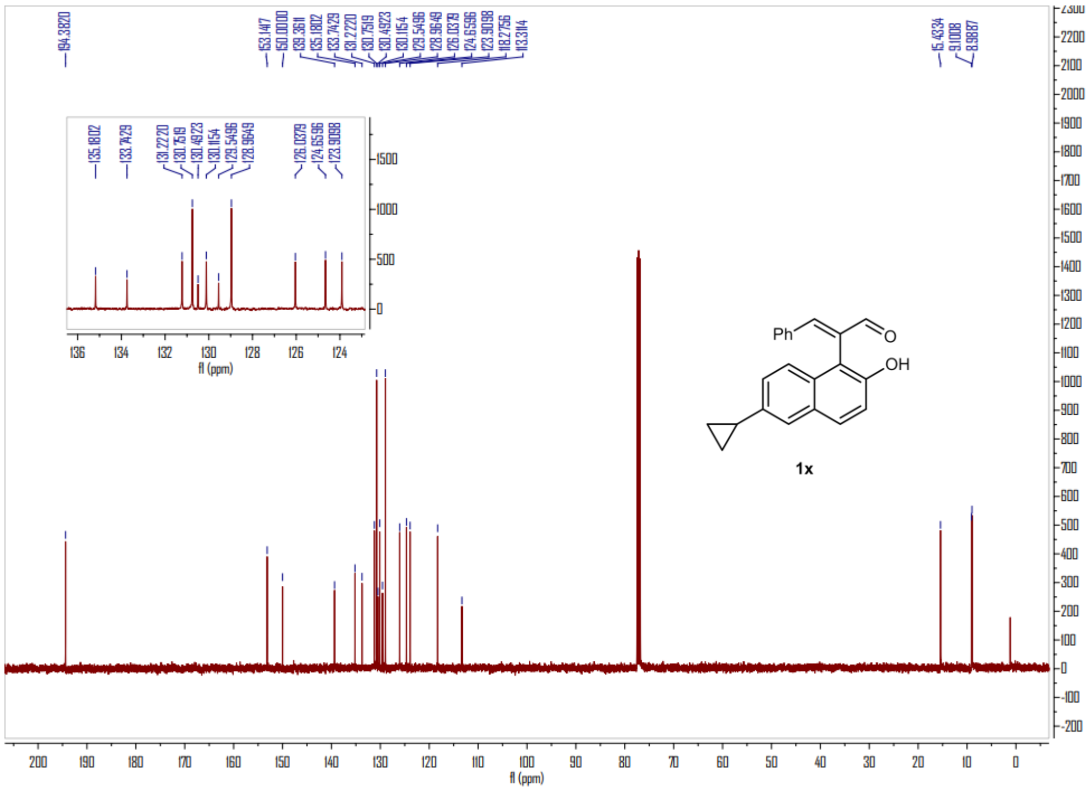

NMR of **1y** (DMSO-d_6)

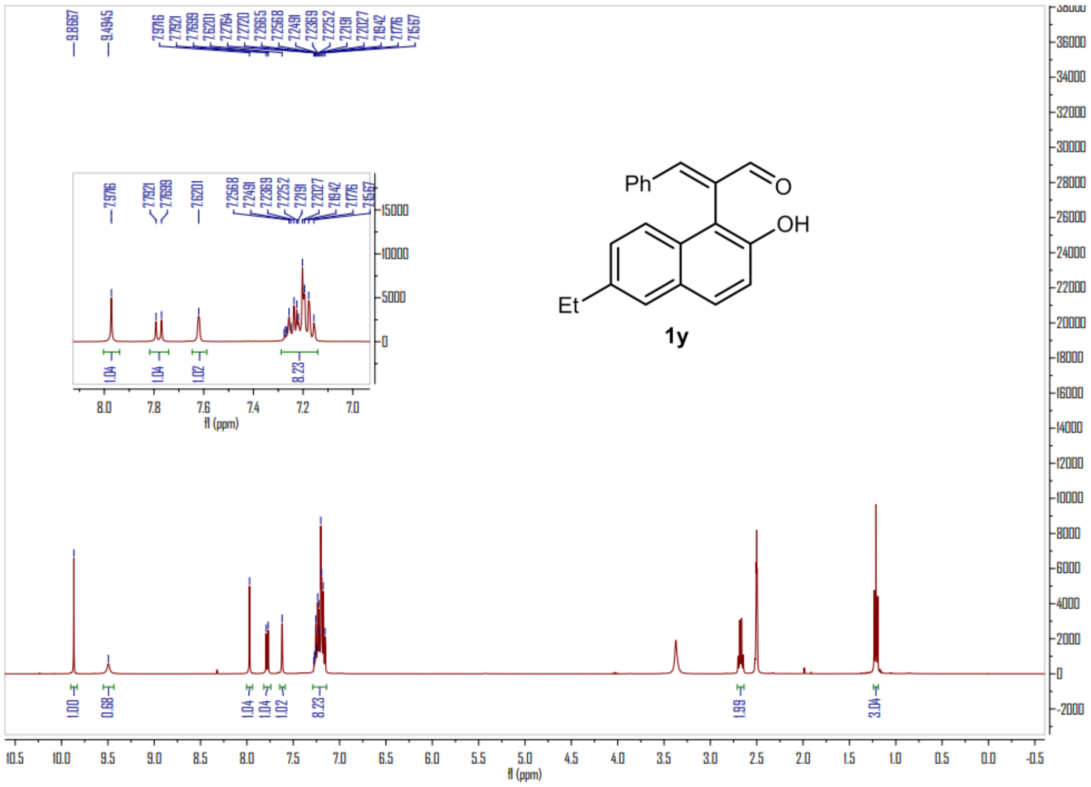

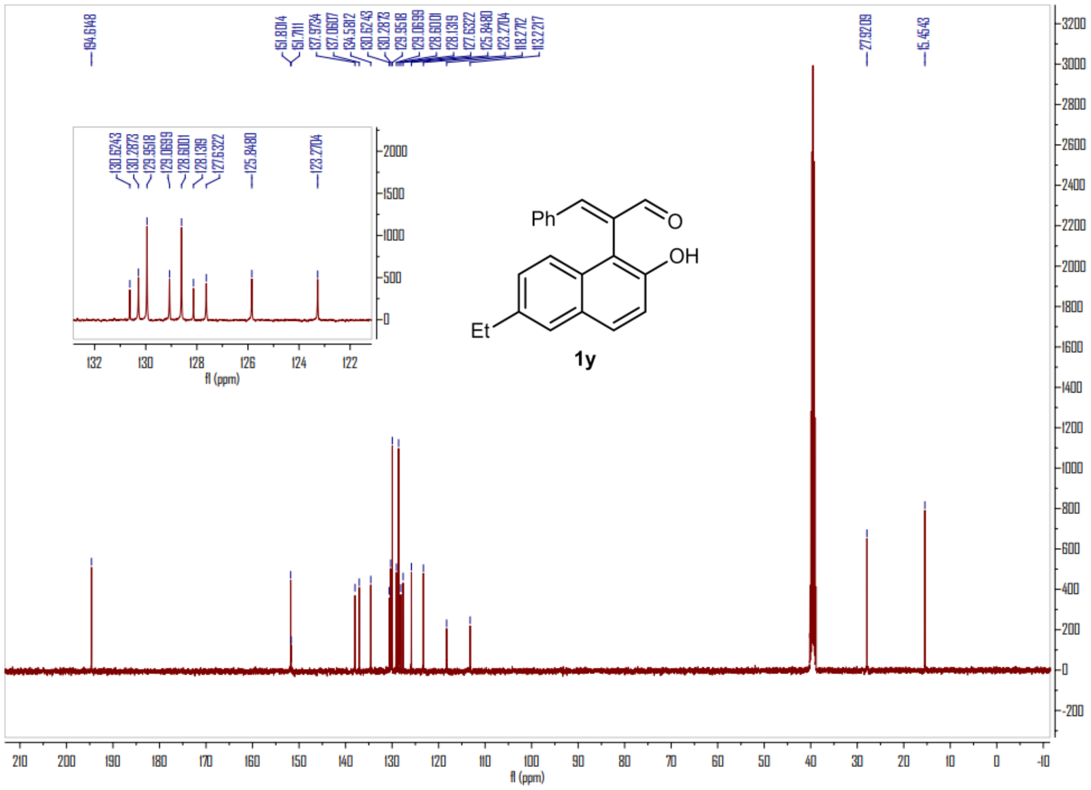

NMR of **1z** ($CDCl_3$)

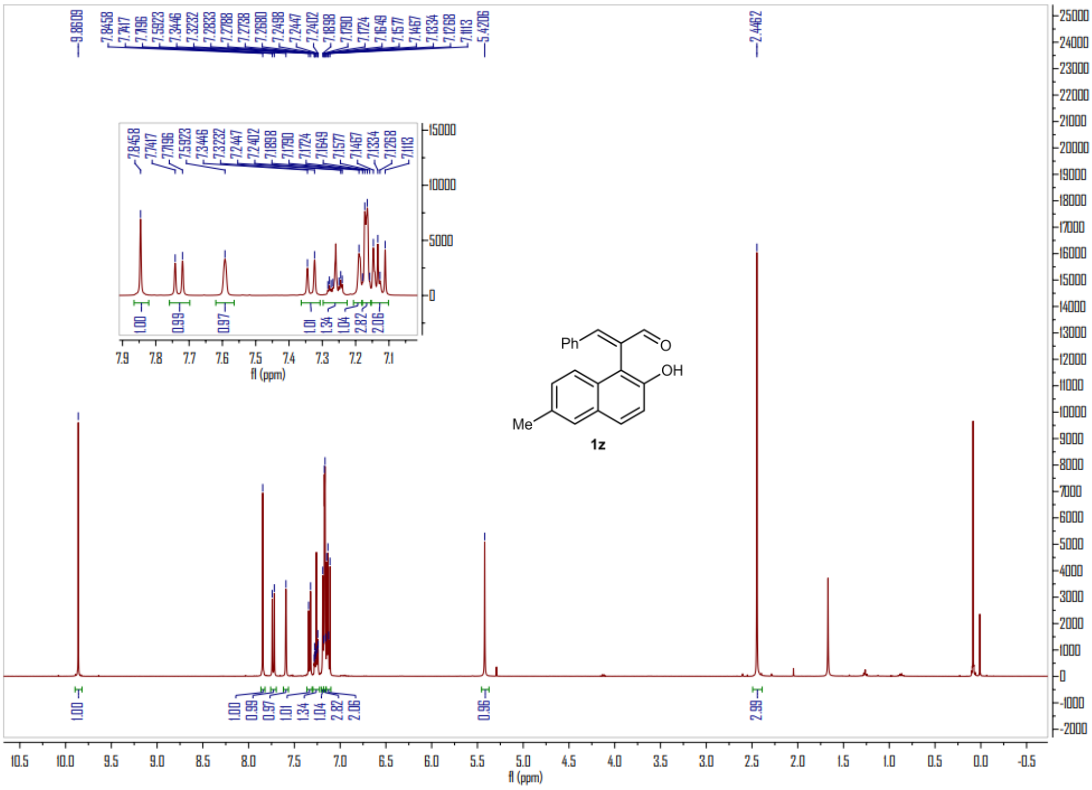

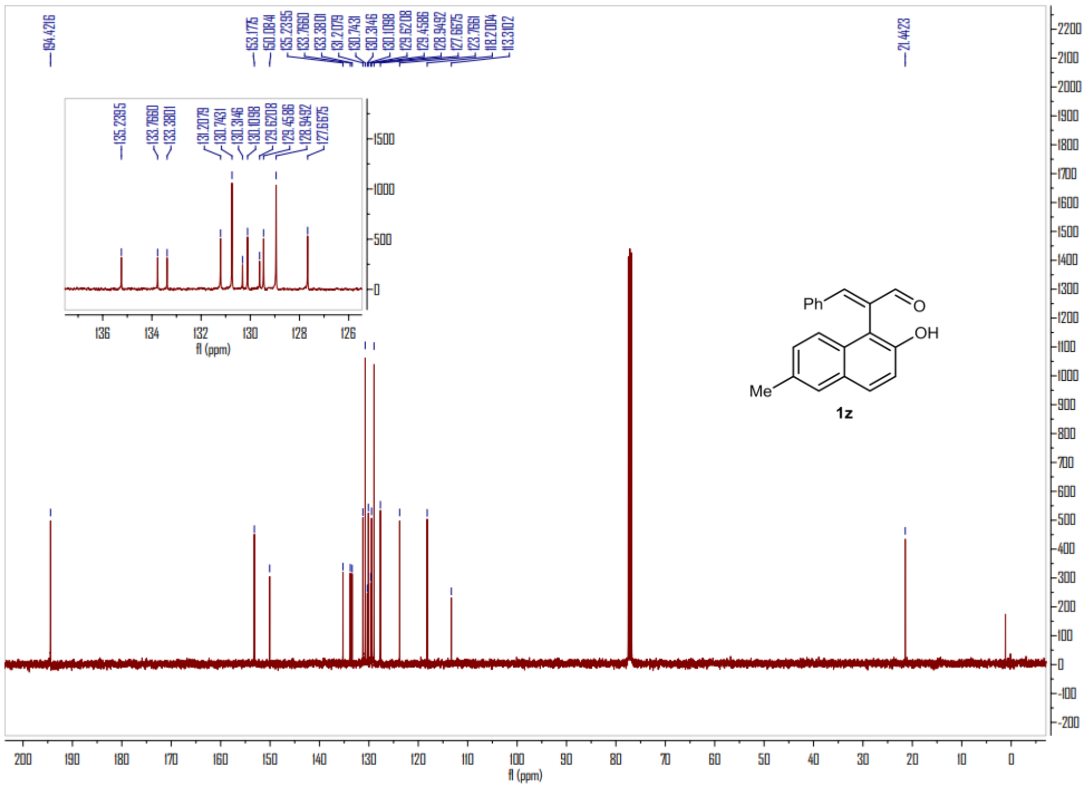

NMR of **1ab** ($CDCl_3$)

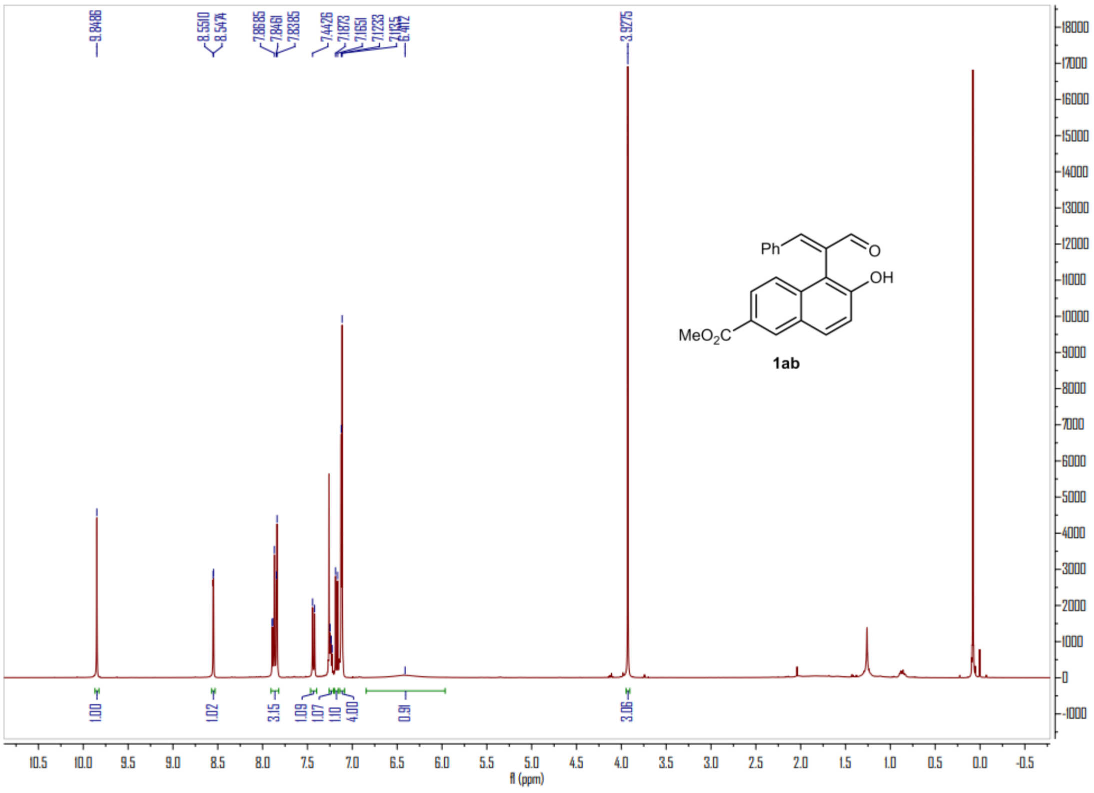

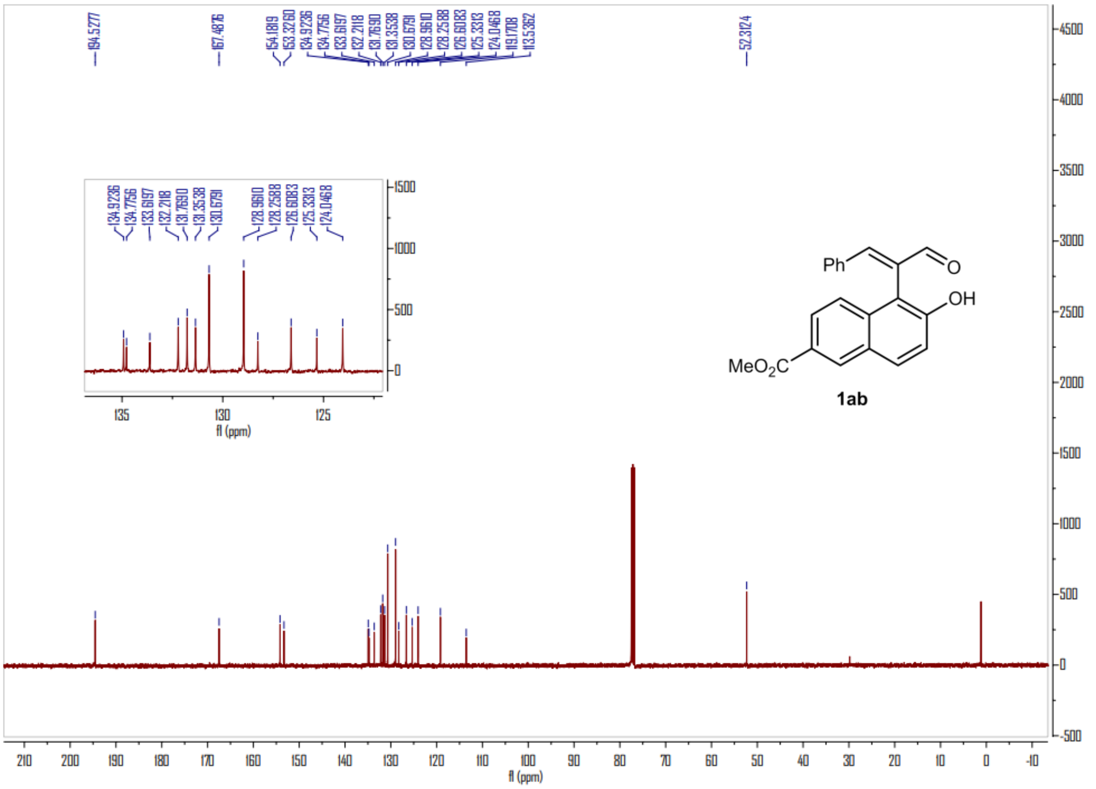

NMR of **1ac** (MeOH-d_4)

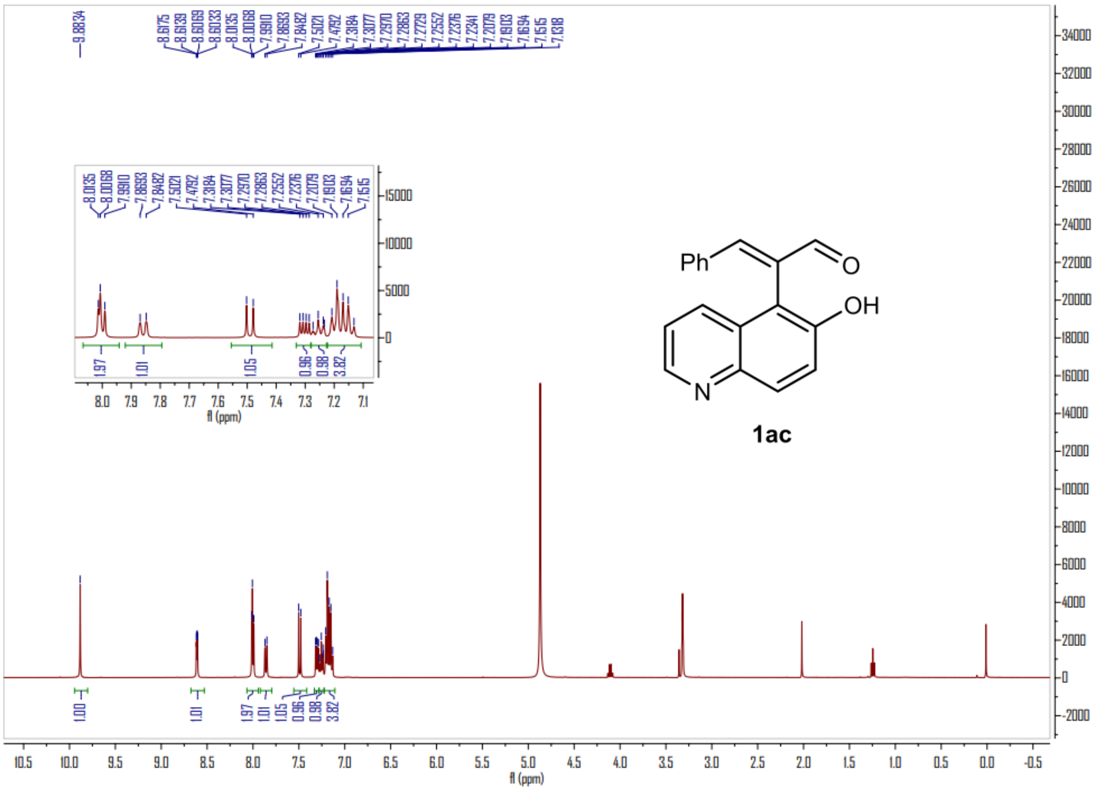

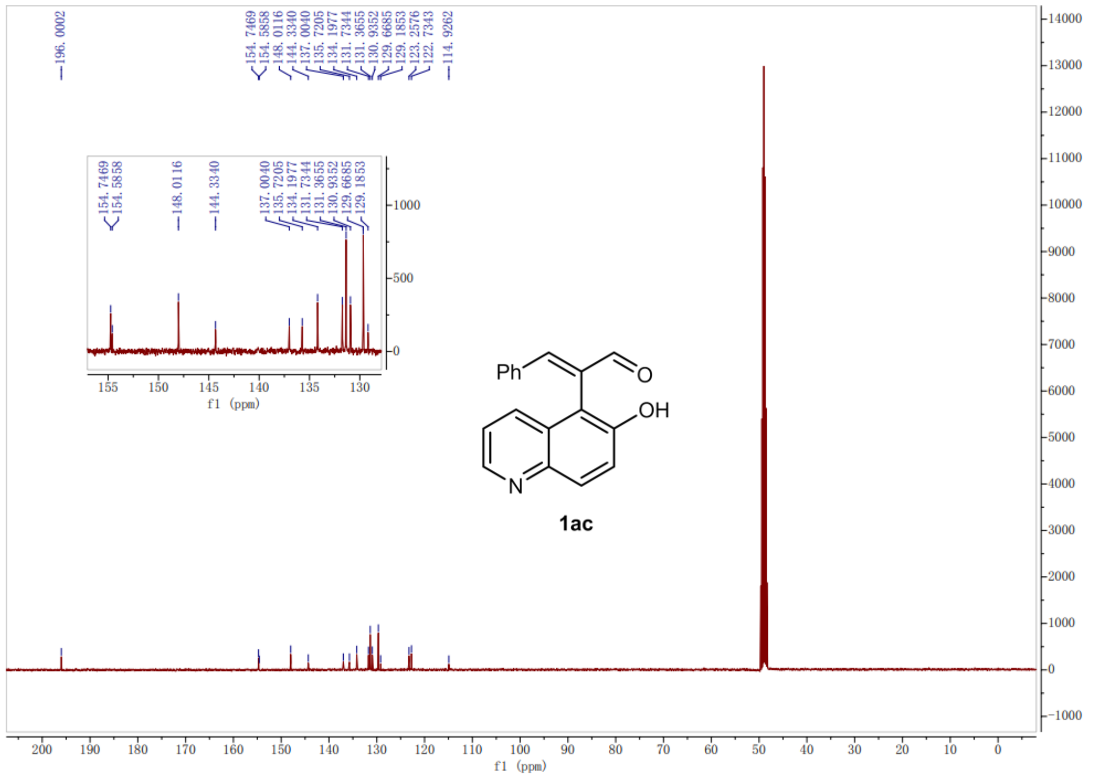

NMR of **1ad** ($CDCl_3$)

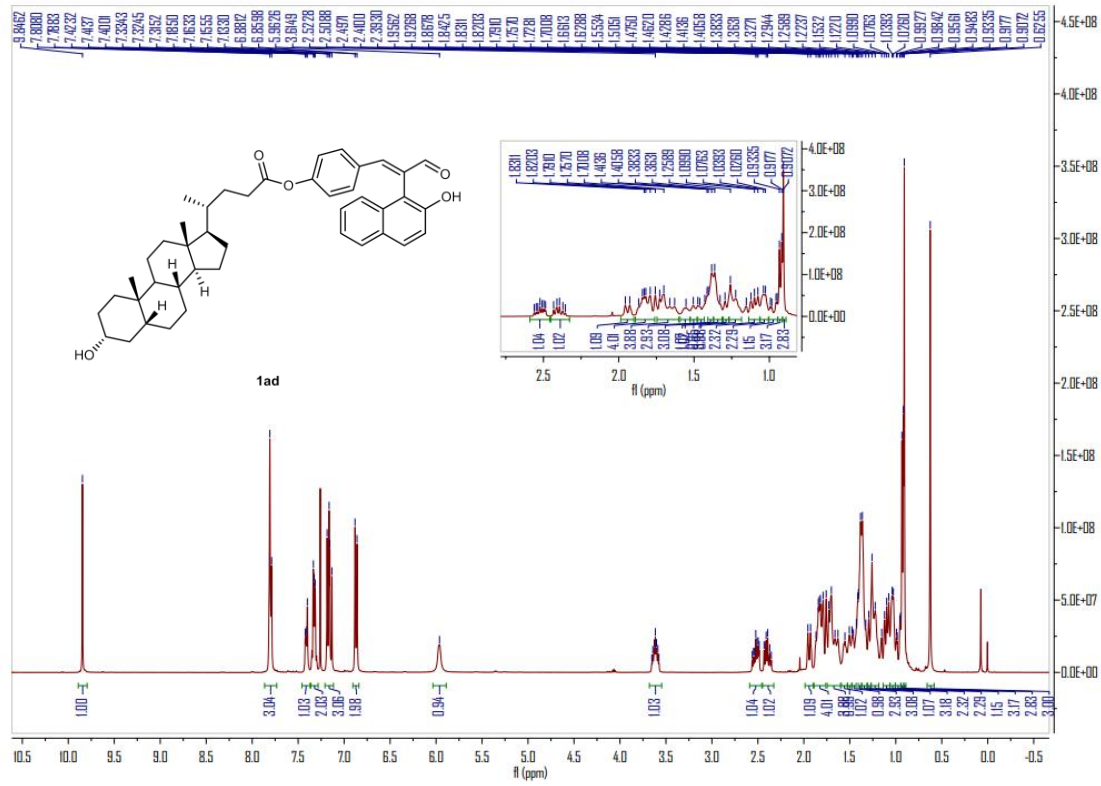

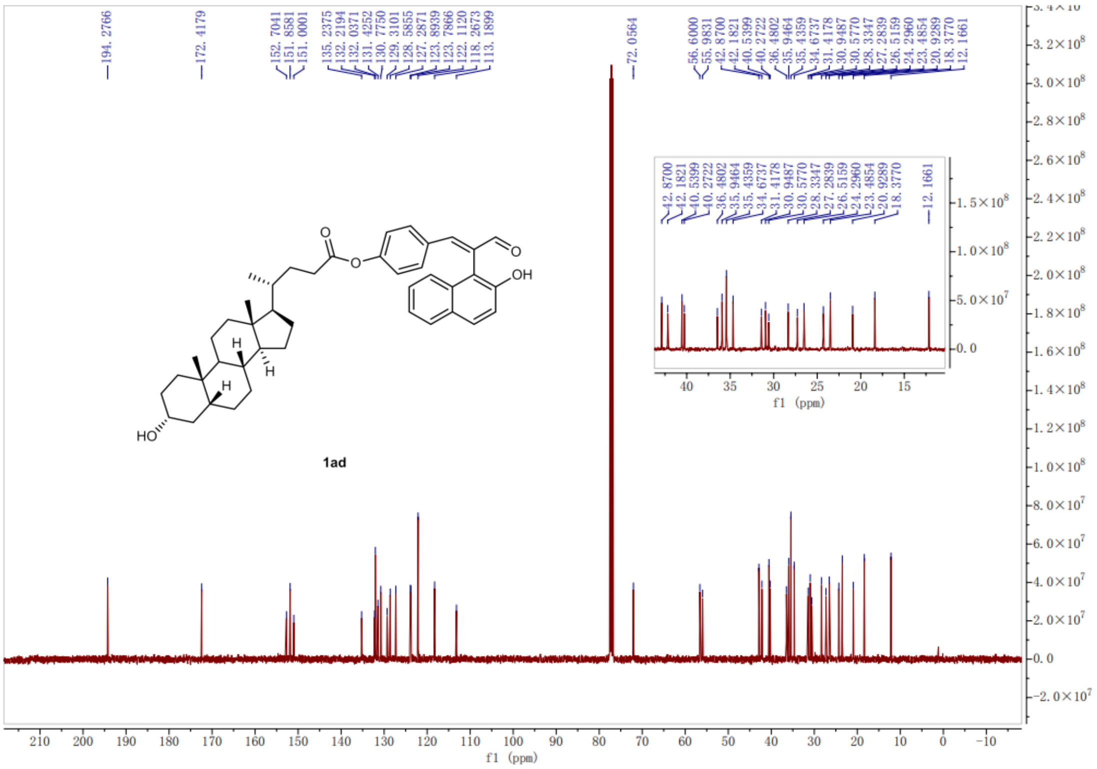

NMR of **1ae** ($CDCl_3$)

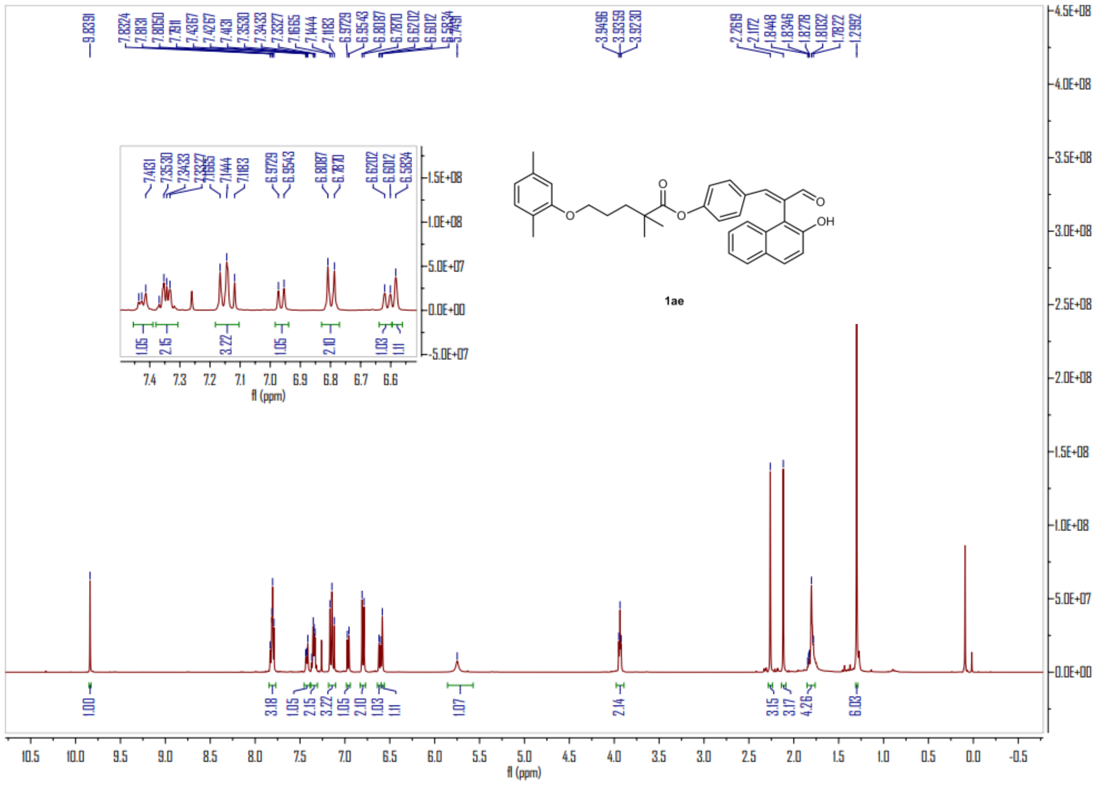

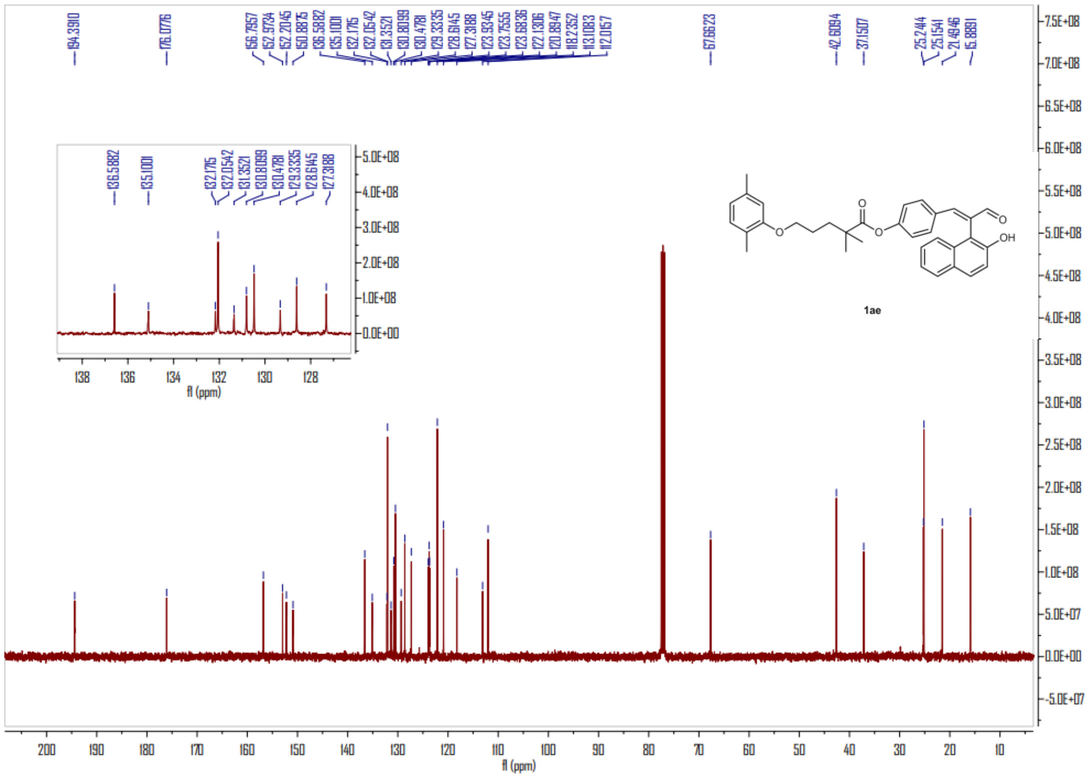

NMR of **1af** ($CDCl_3$)

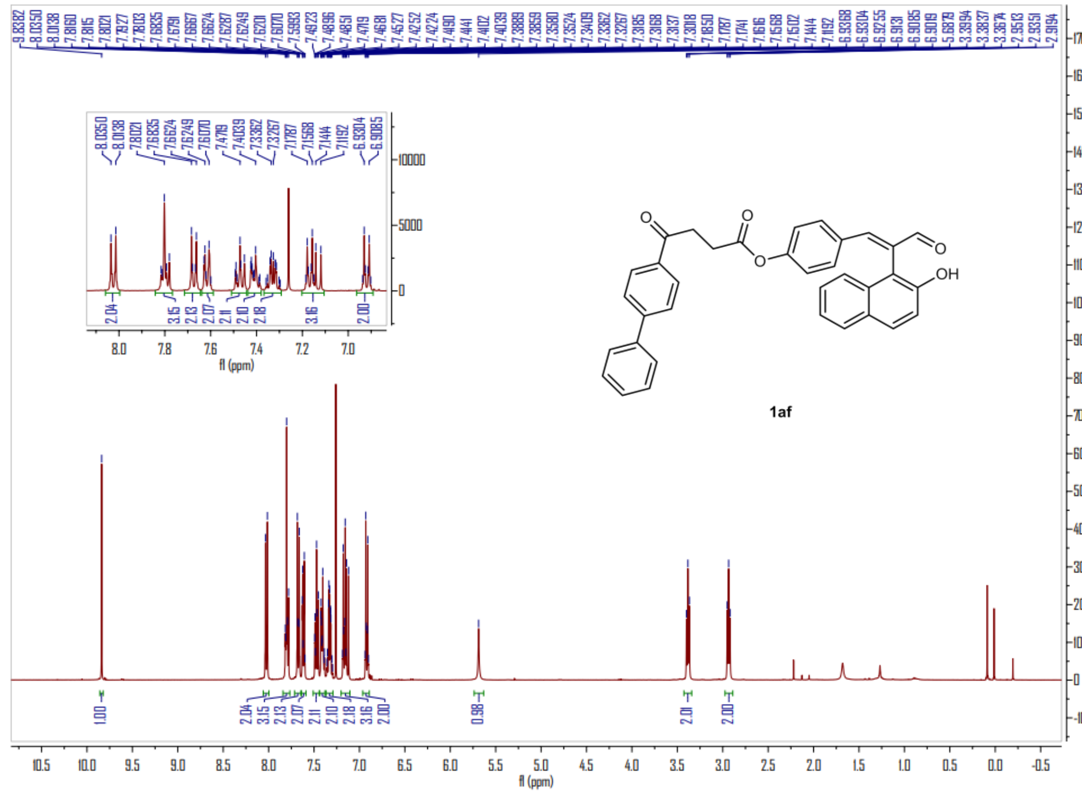

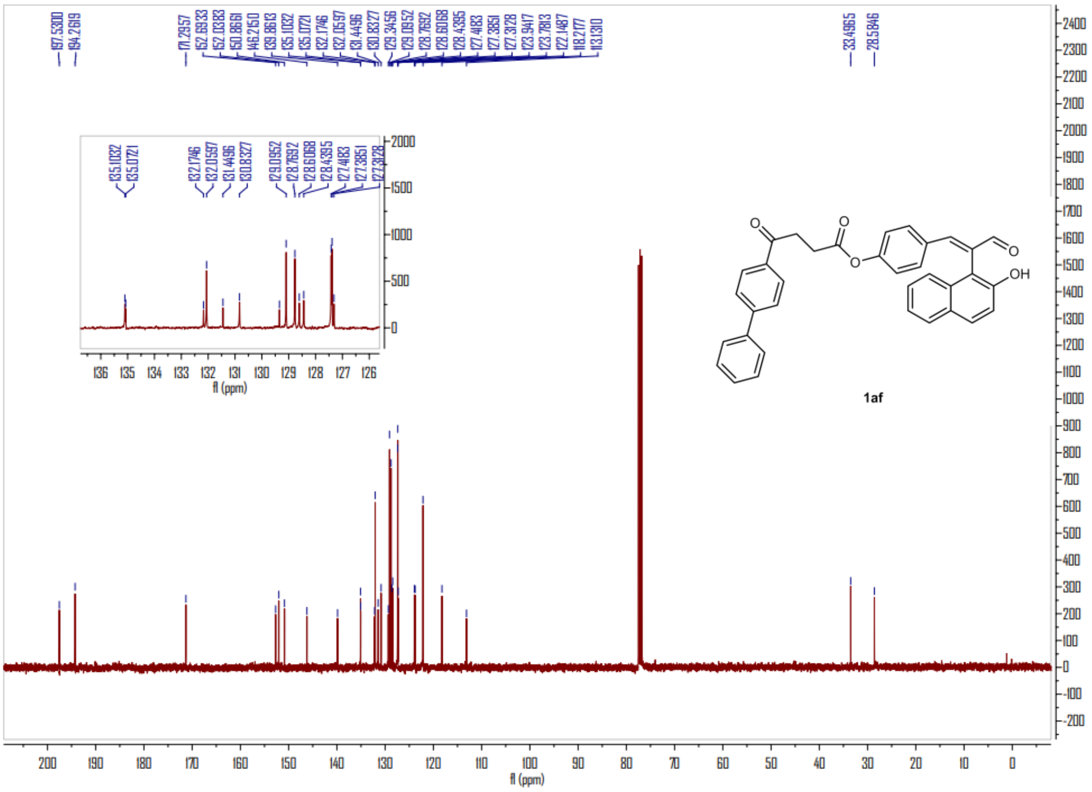

NMR of **1ag** ($CDCl_3$)

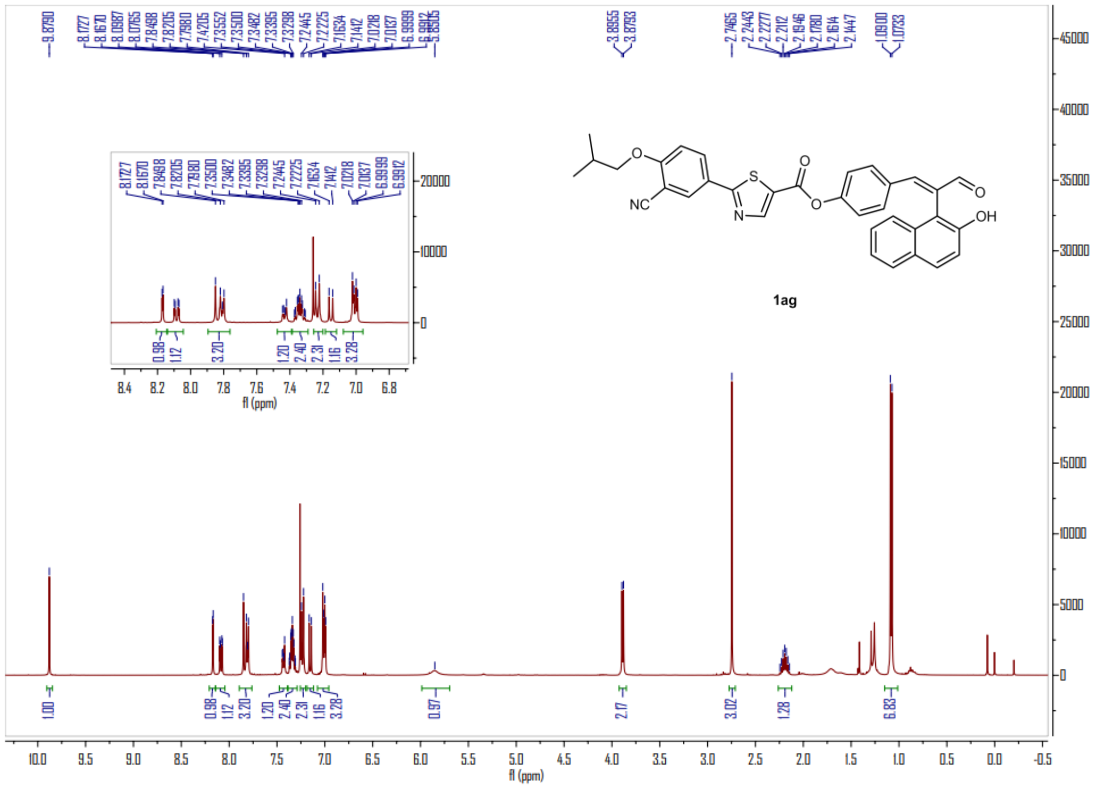

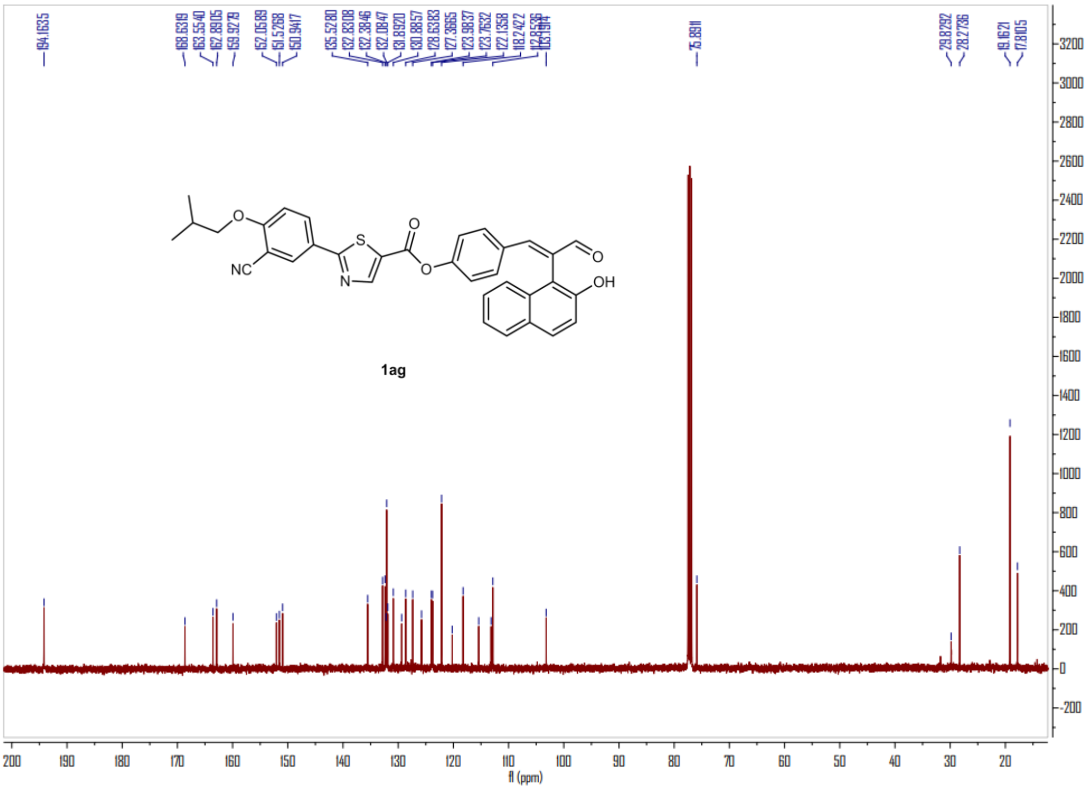

NMR of **1ah** ($CDCl_3$)

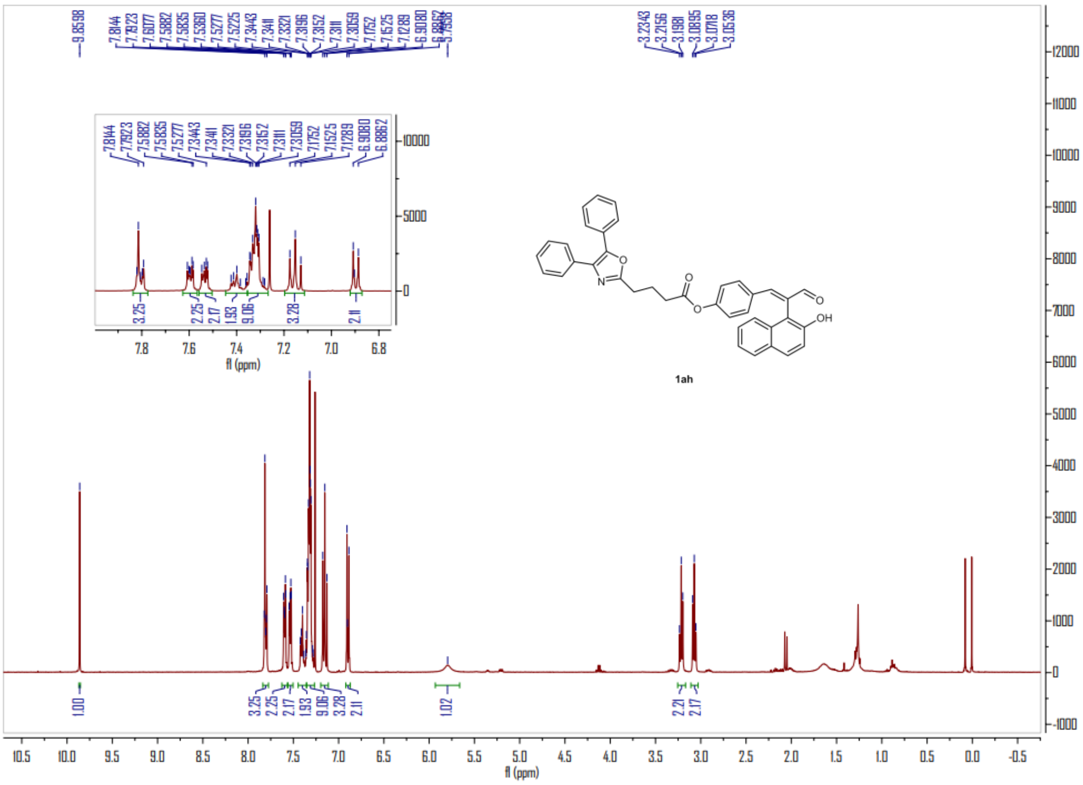

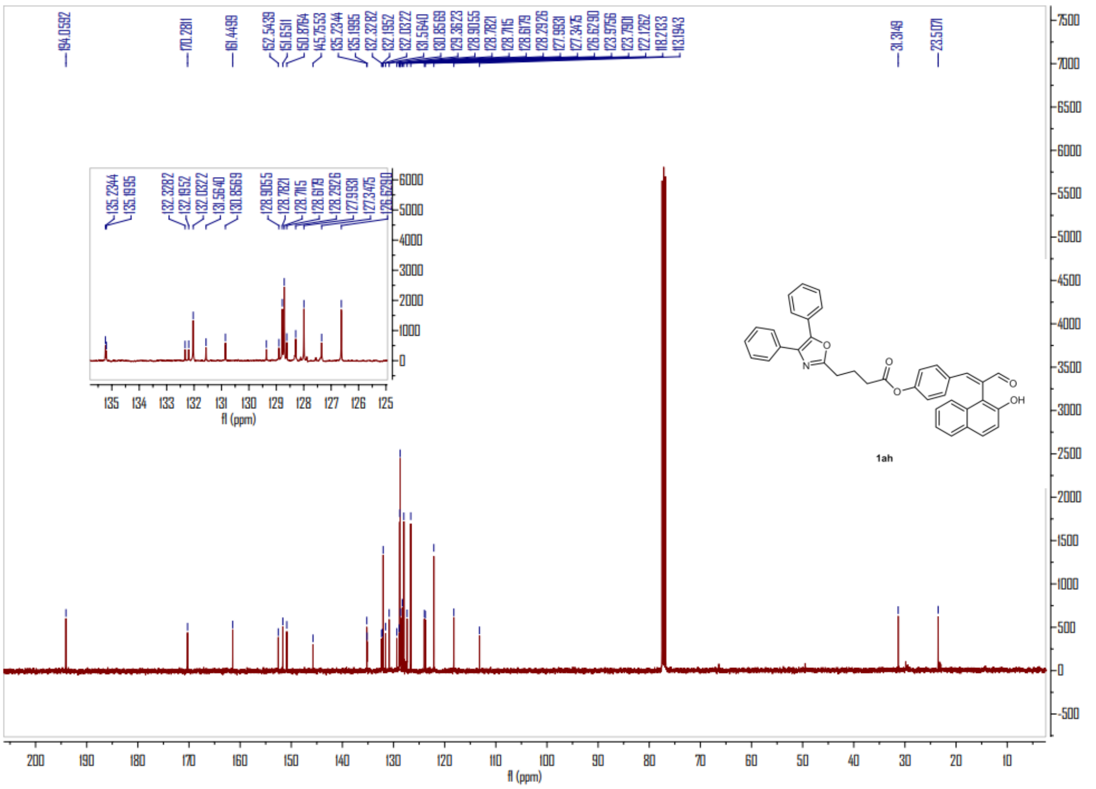

1ah

NMR of **1ai** ($CDCl_3$)

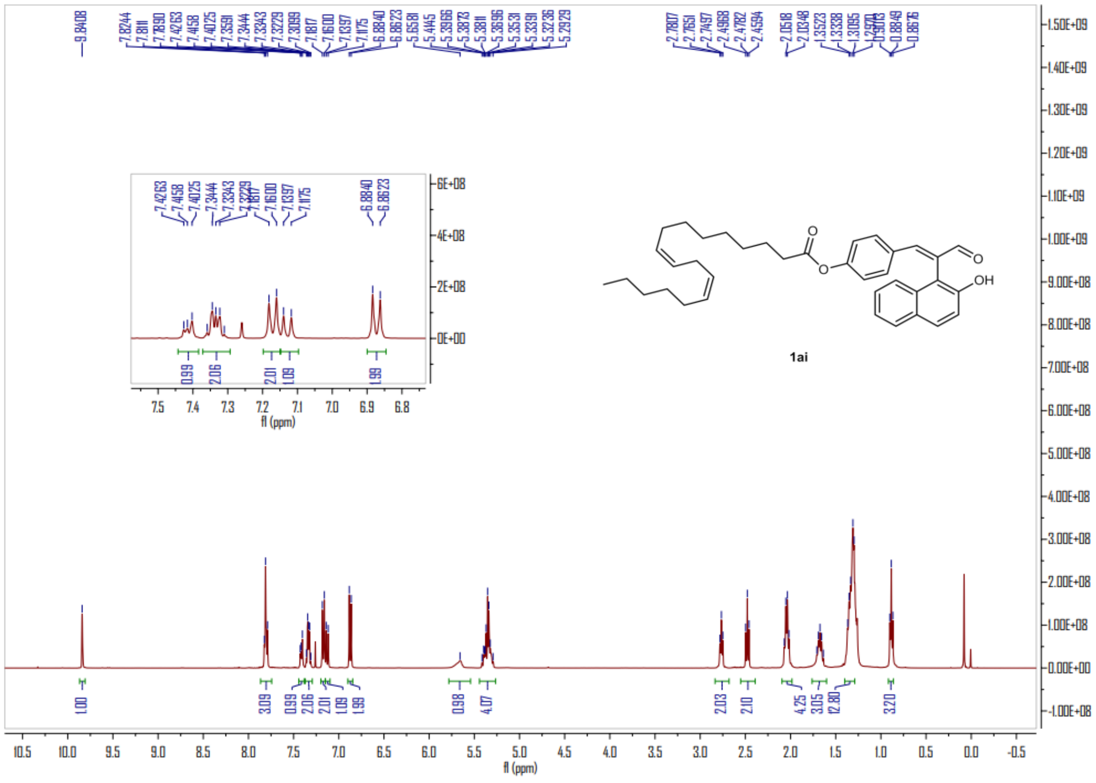

1ai

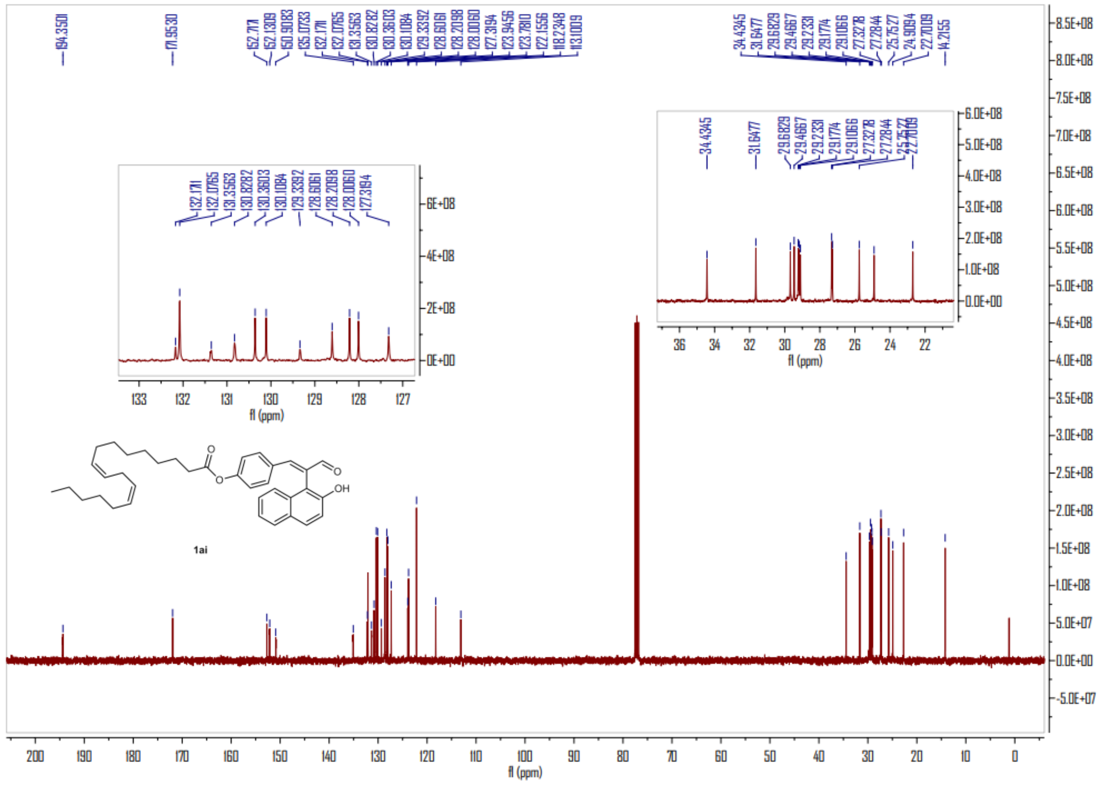

NMR of **1aj** ($CDCl_3$)

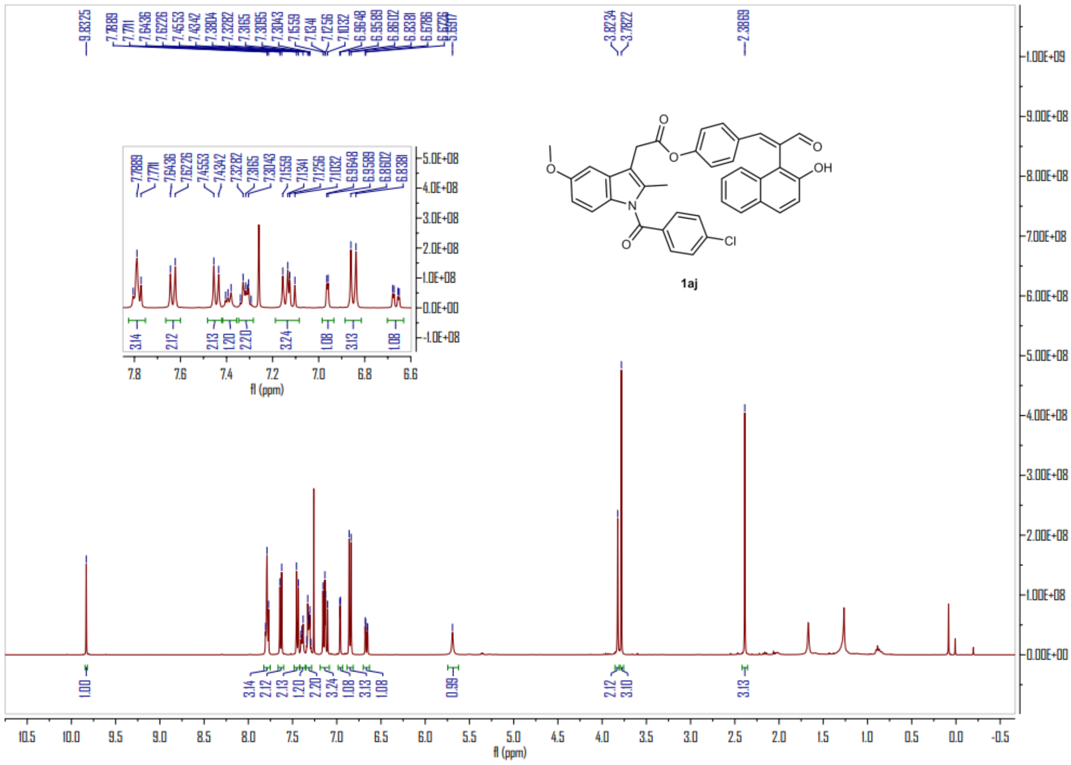

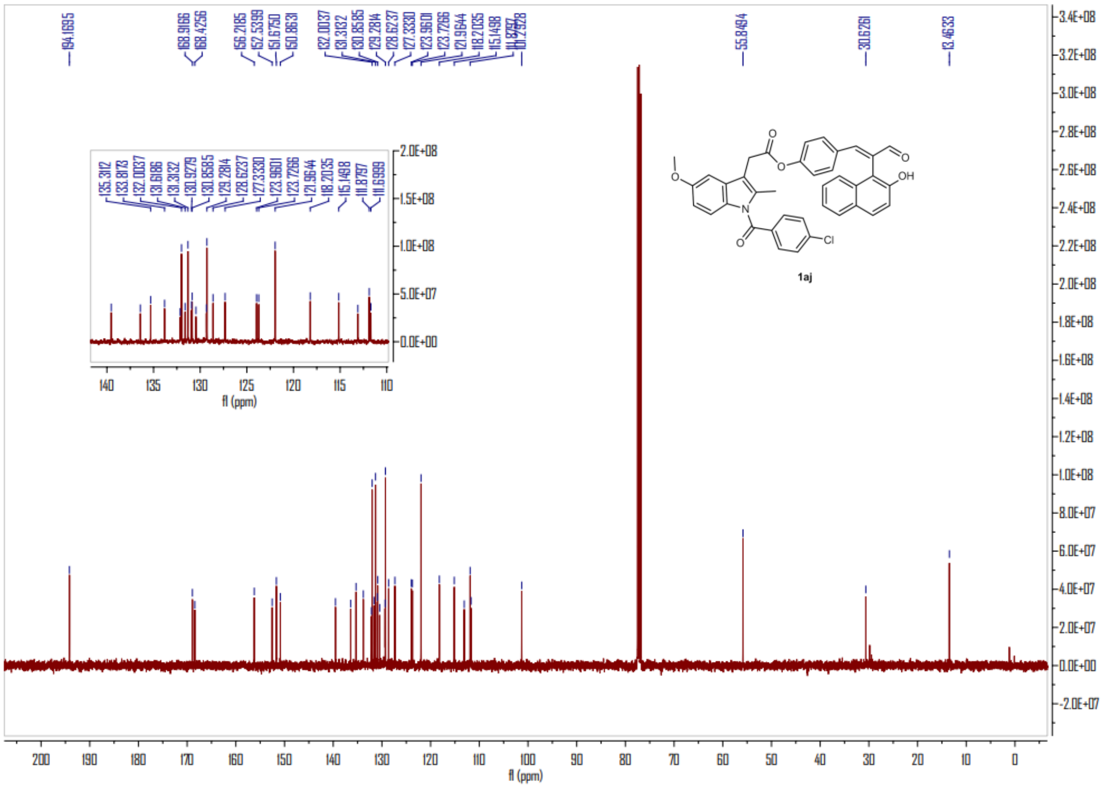

NMR of **1ak** ($CDCl_3$)

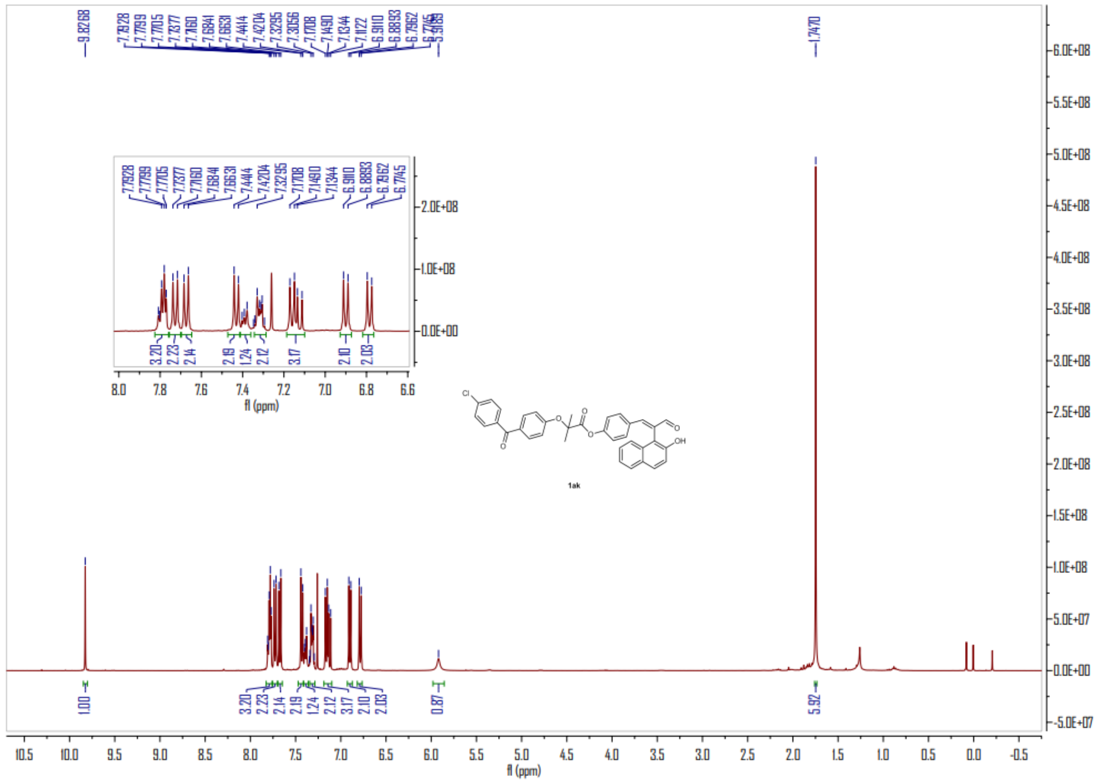

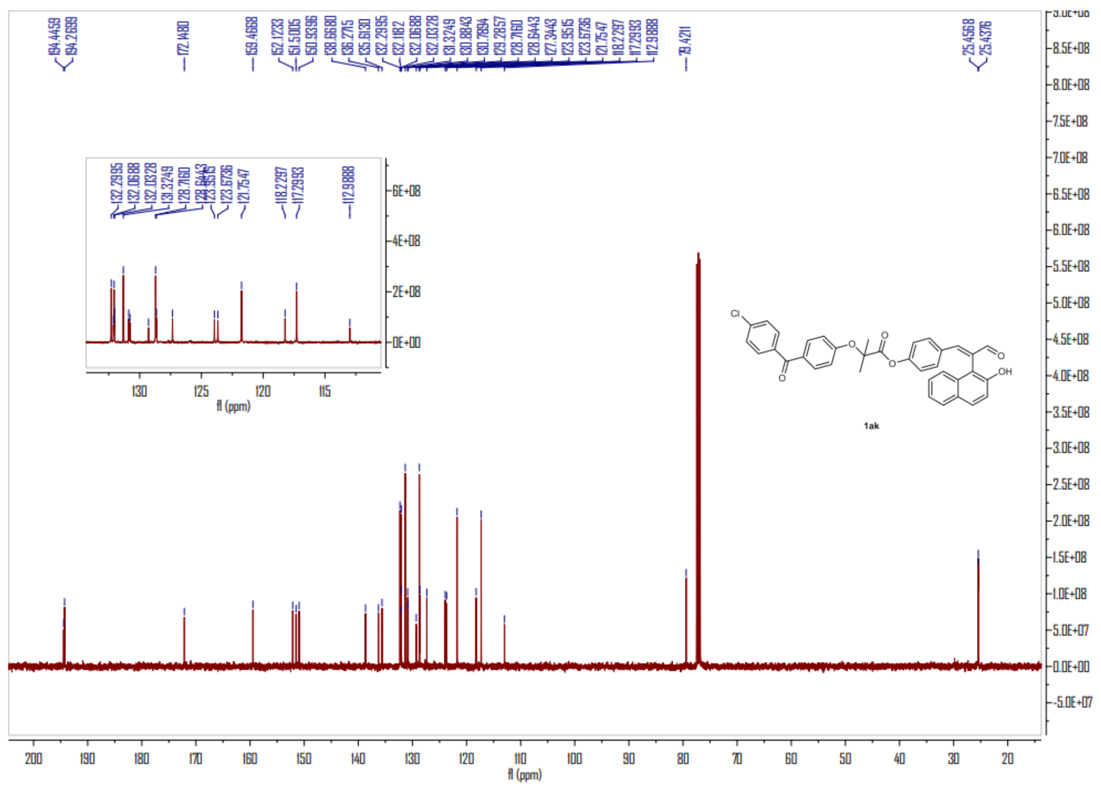

NMR of **1al** ($CDCl_3$)

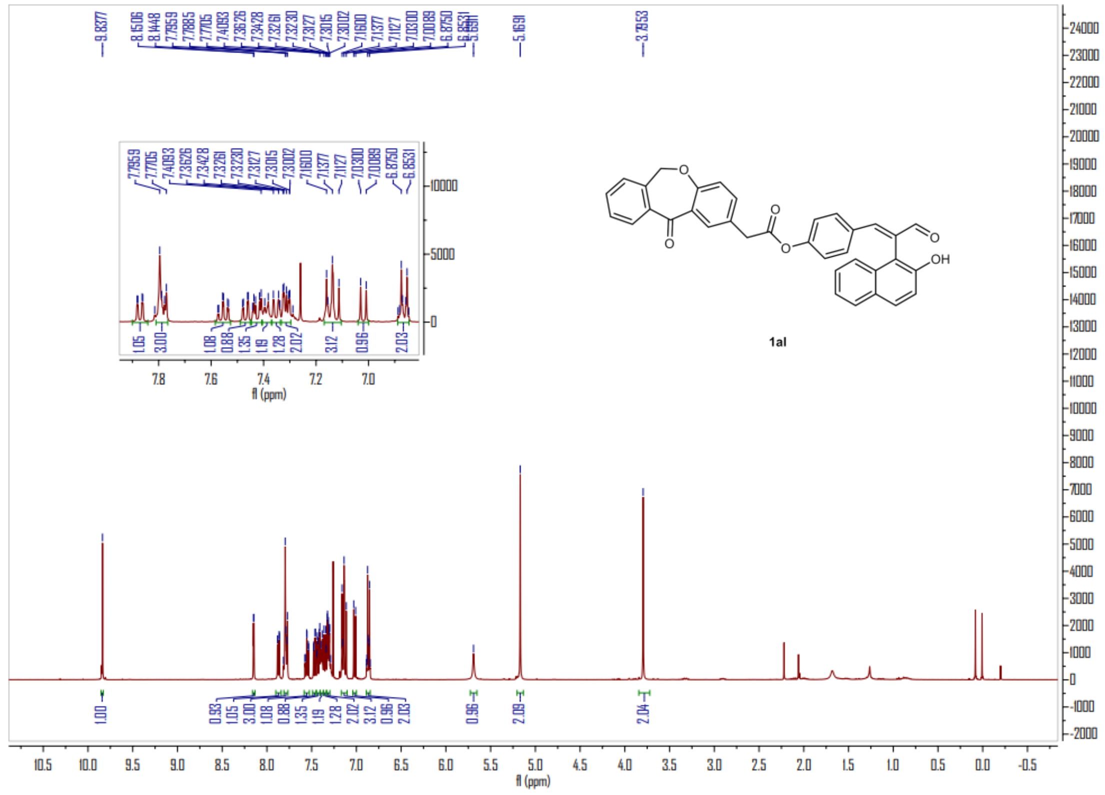

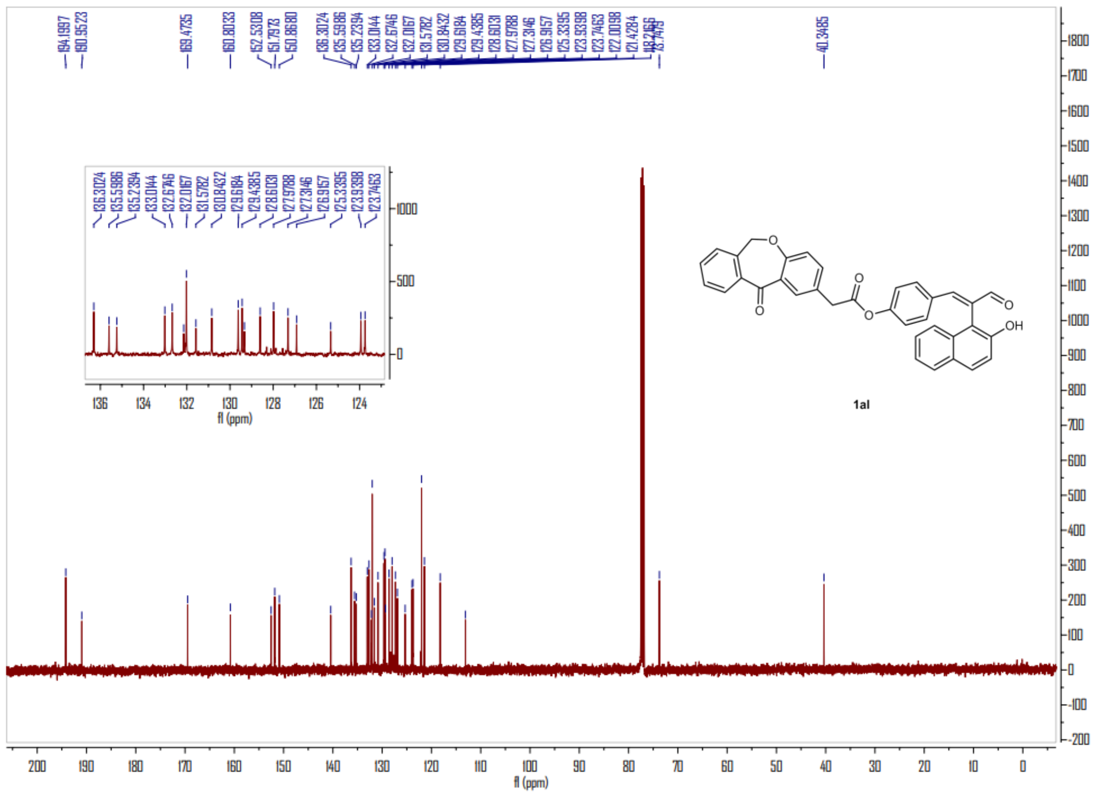

NMR of **P6** ($CDCl_3$)

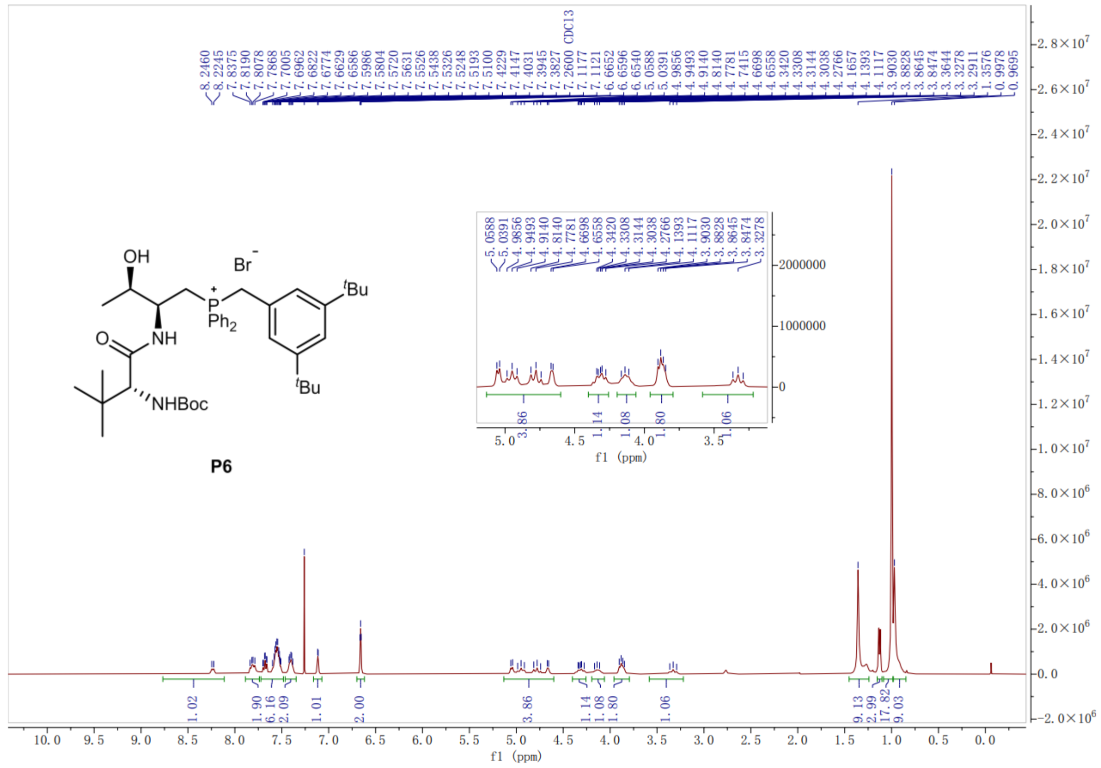

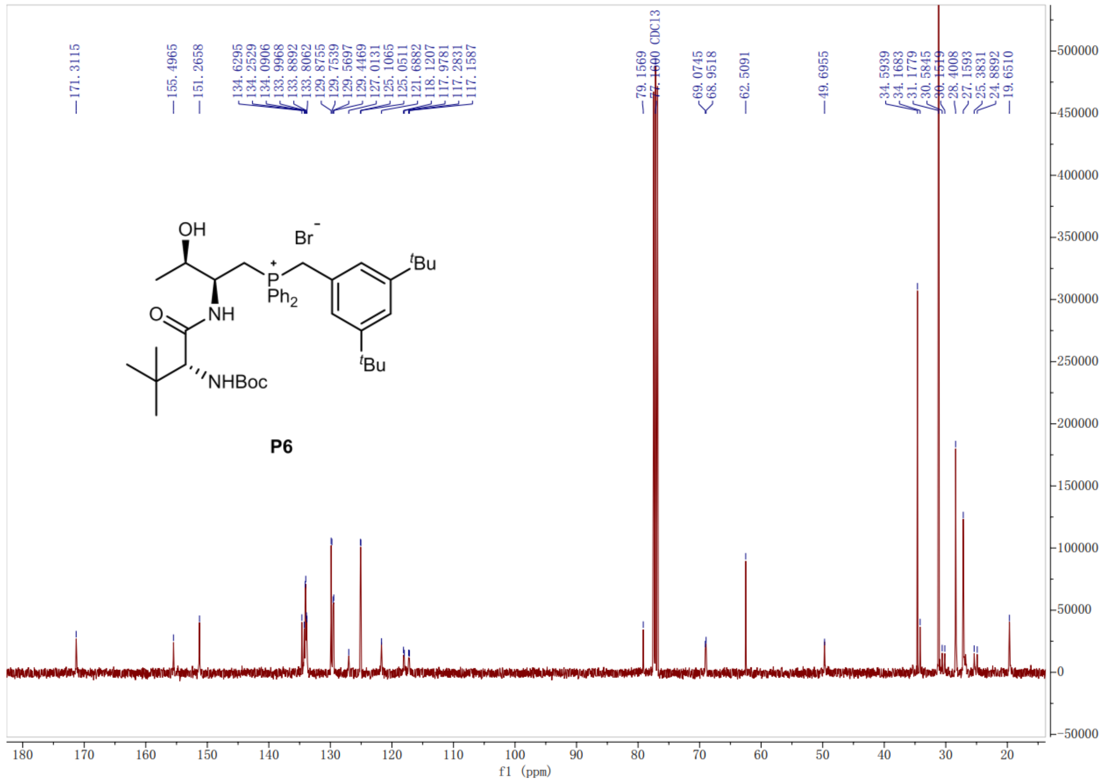

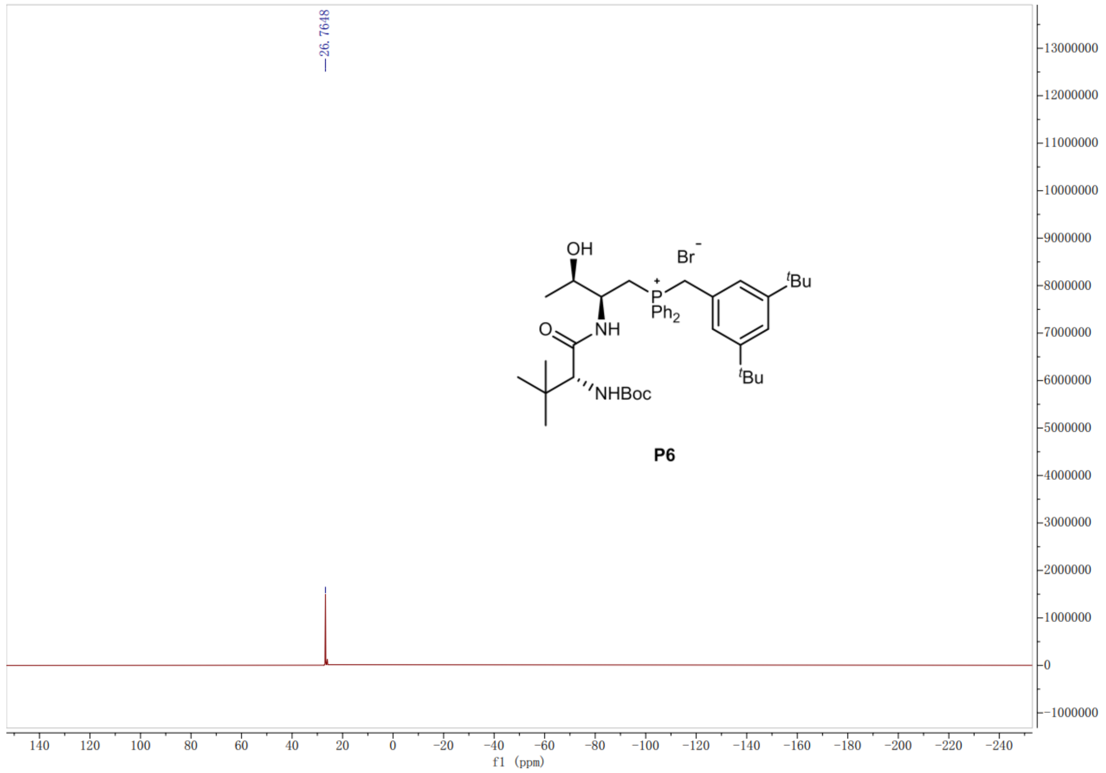

NMR of **P7** ($CDCl_3$)

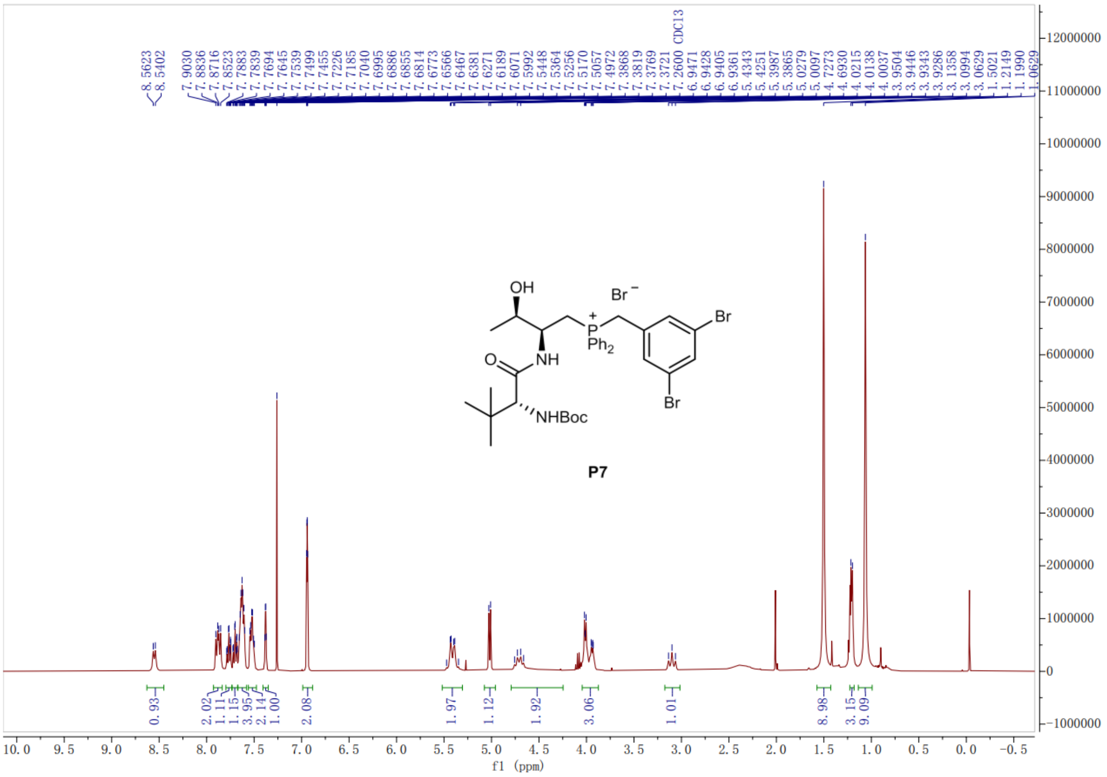

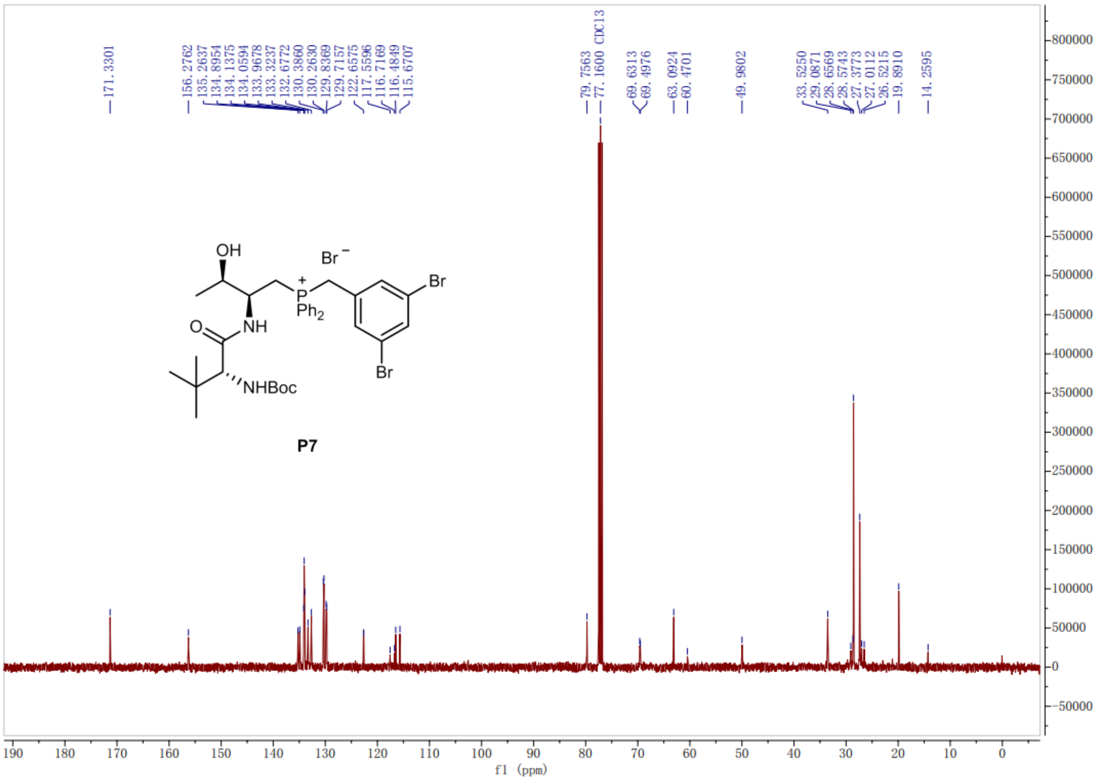

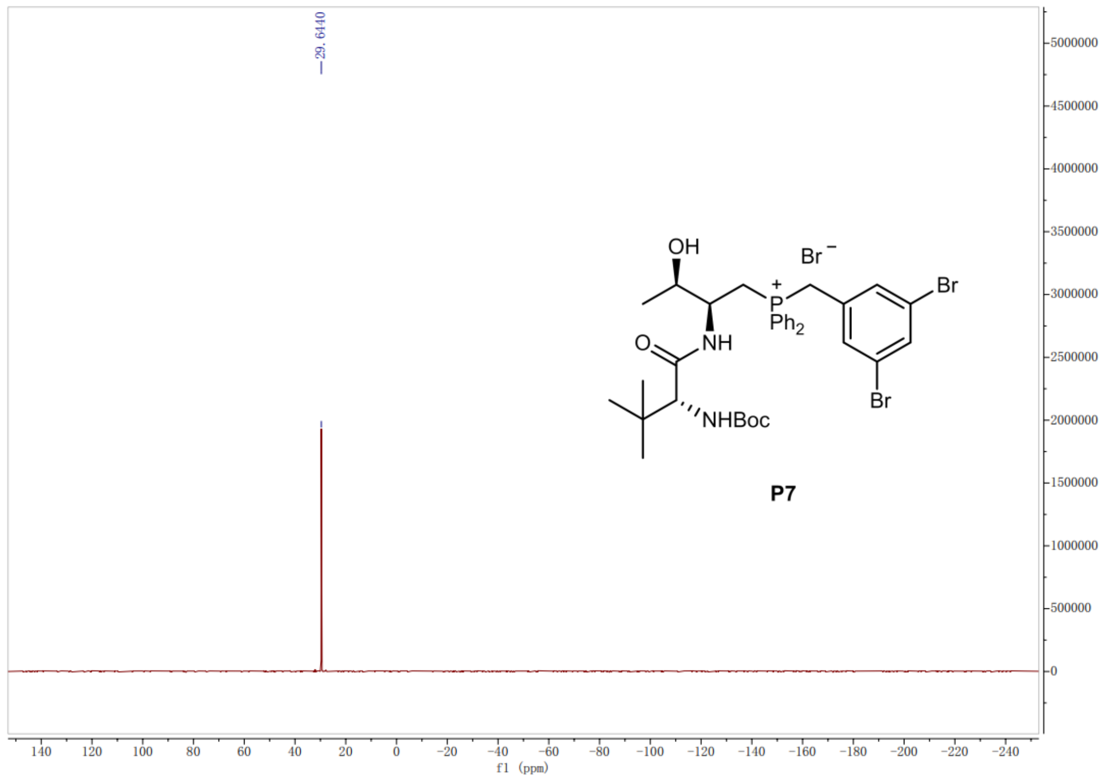

NMR of **P8** ($CDCl_3$)

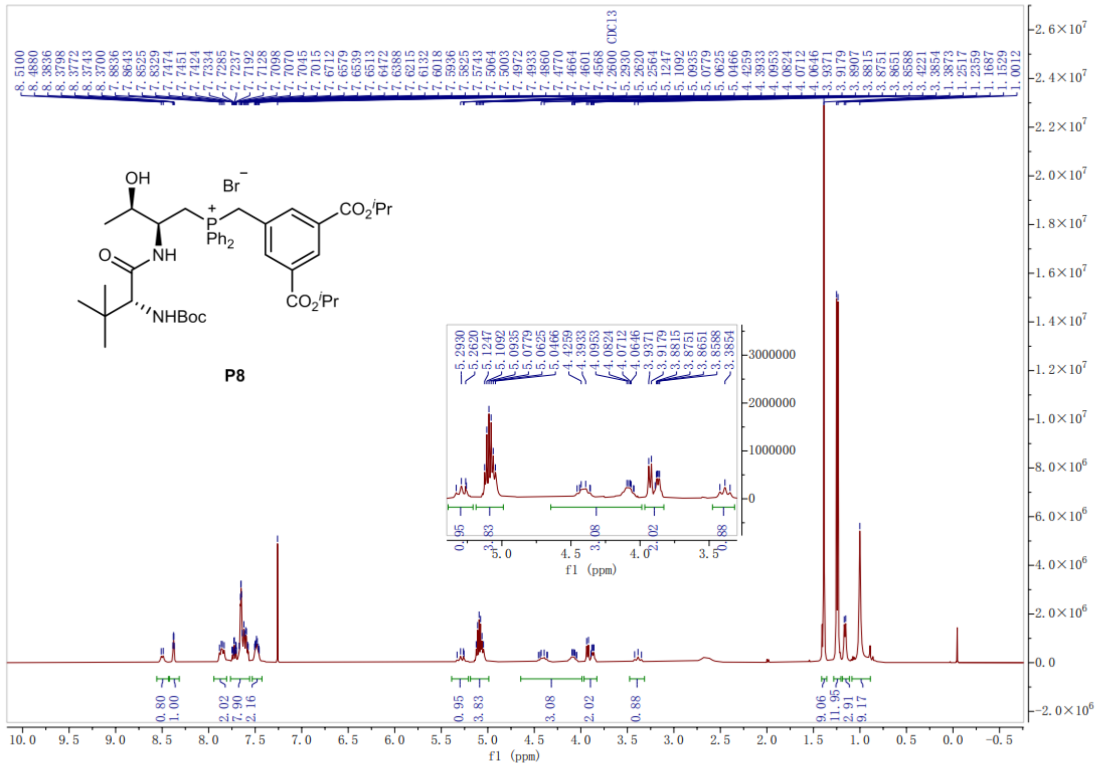

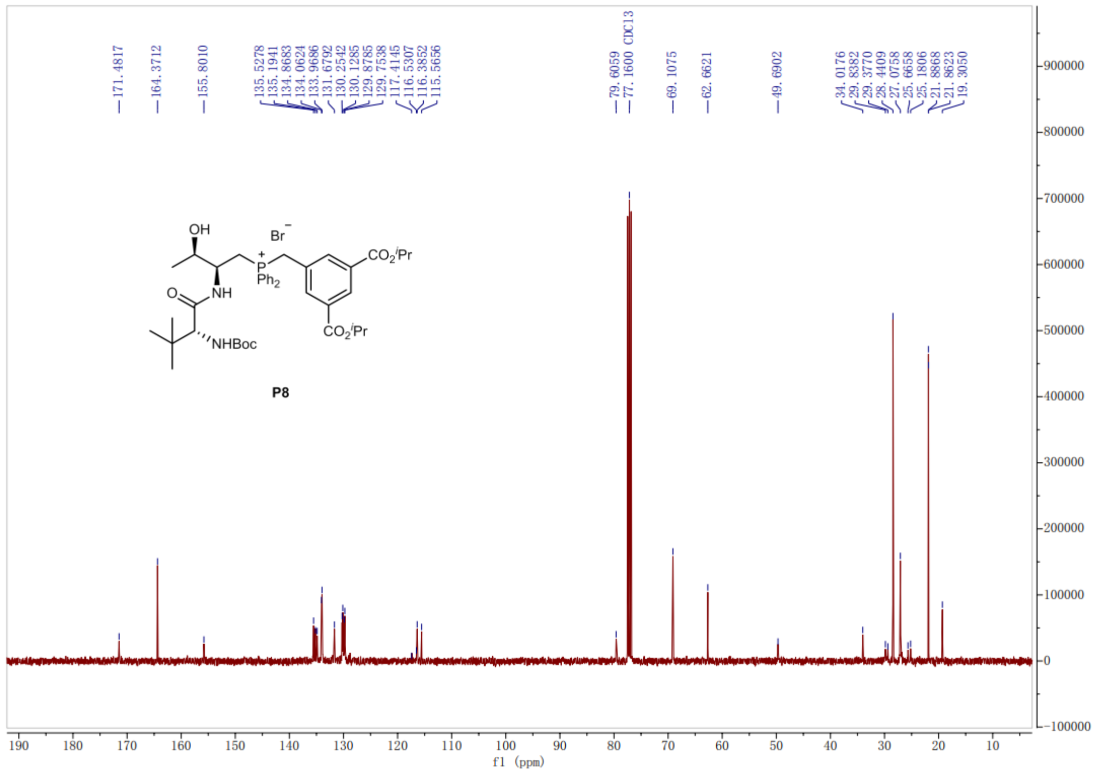
171.4817
164.3712
155.8010
135.5278
135.1941
134.8683
134.0624
133.9686
131.6792
130.2542
130.1285
129.8785
129.7538
117.4145
116.5307
116.3852
115.5656
79.6059
77.1600 CDCl3
69.1075
62.6621
49.6902
34.0176
29.8382
29.3770
28.4409
27.0758
25.6658
25.1806
21.8868
21.8623
19.3050
OH
Br
NH
Ph2
CO2iPr
NHBoc
P8
f1 (ppm)

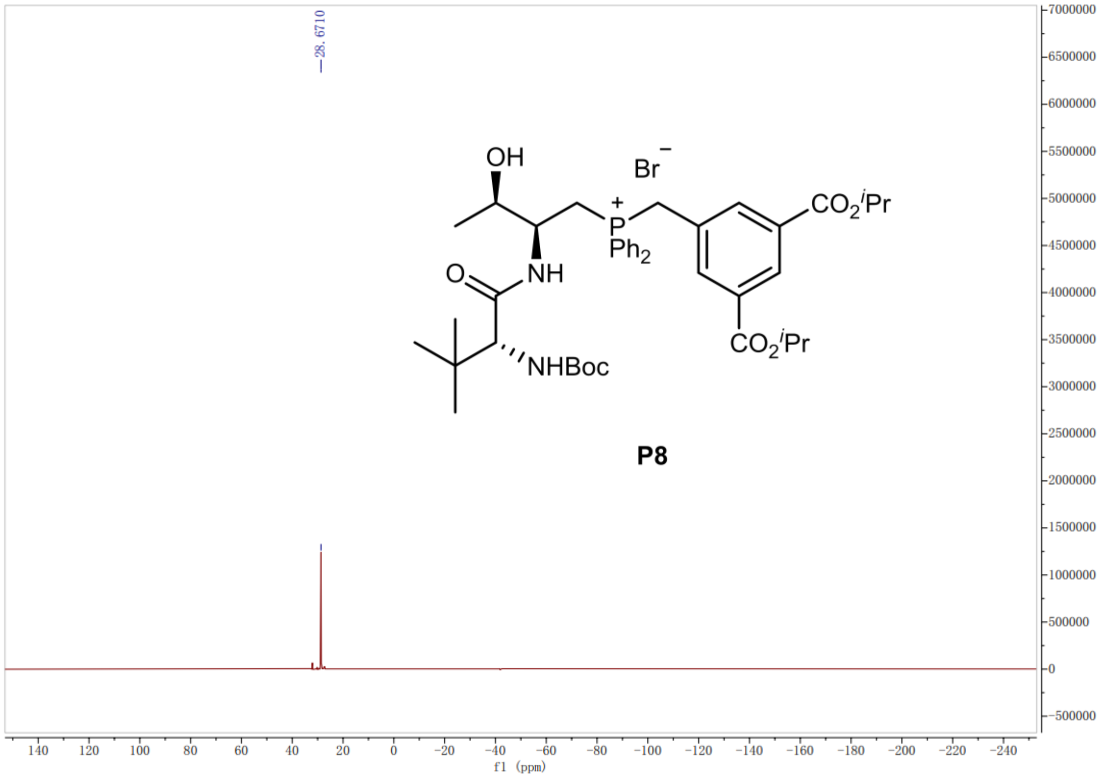
28.6710
OH
Br
NH
Ph2
CO2iPr
NHBoc
P8
f1 (ppm)

NMR of **P9** ($CDCl_3$)

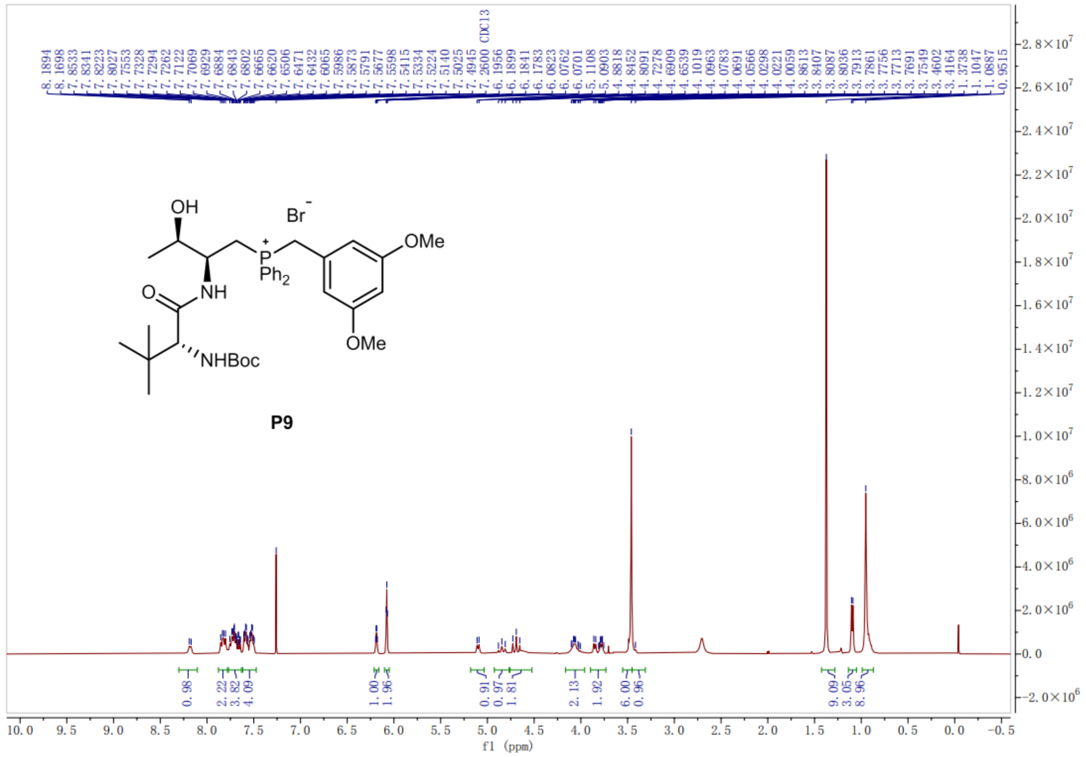

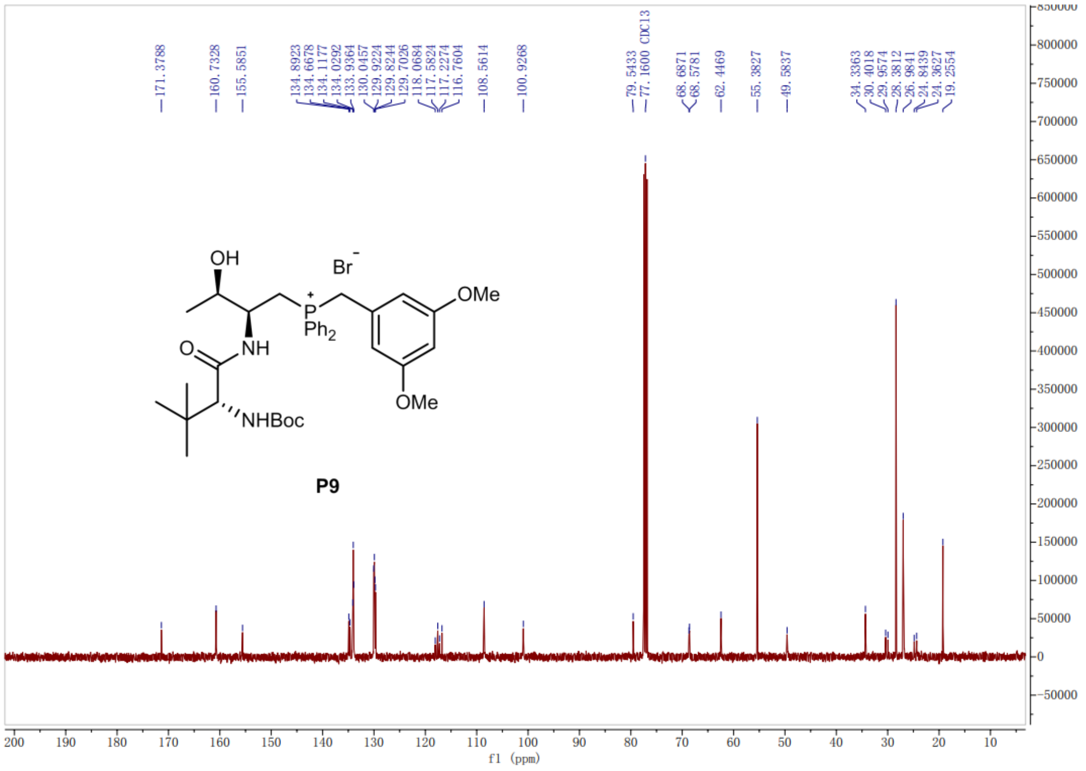

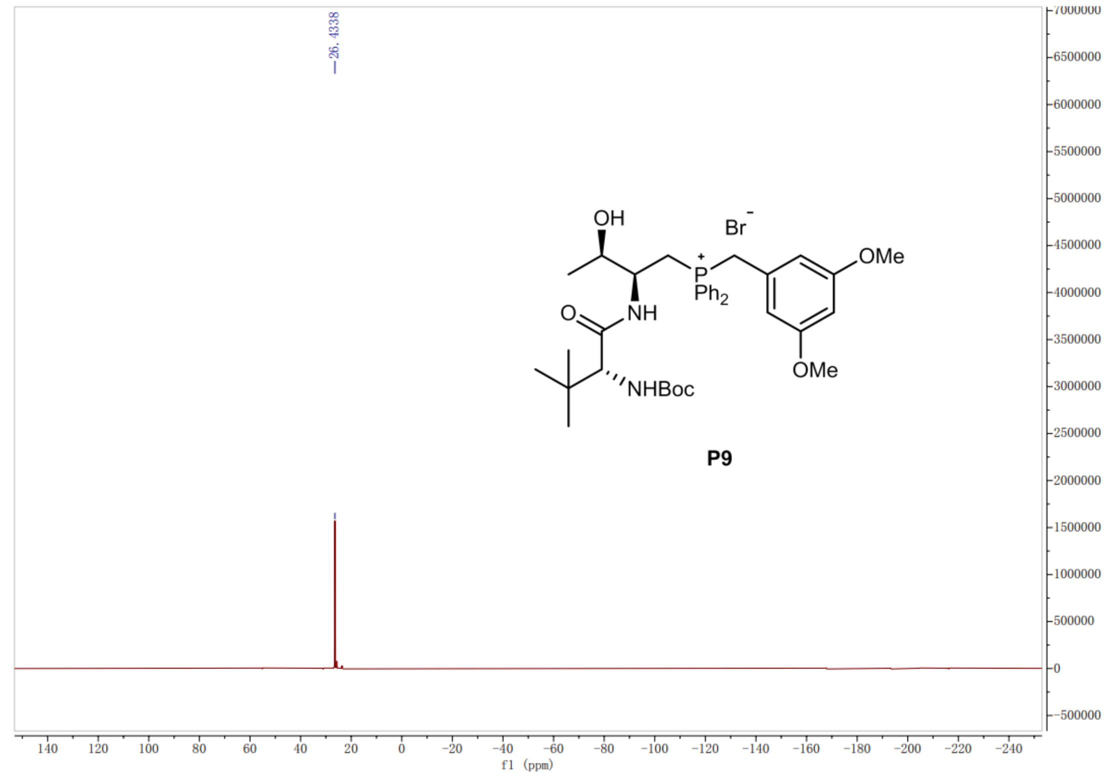

NMR of **P10** ($CDCl_3$)

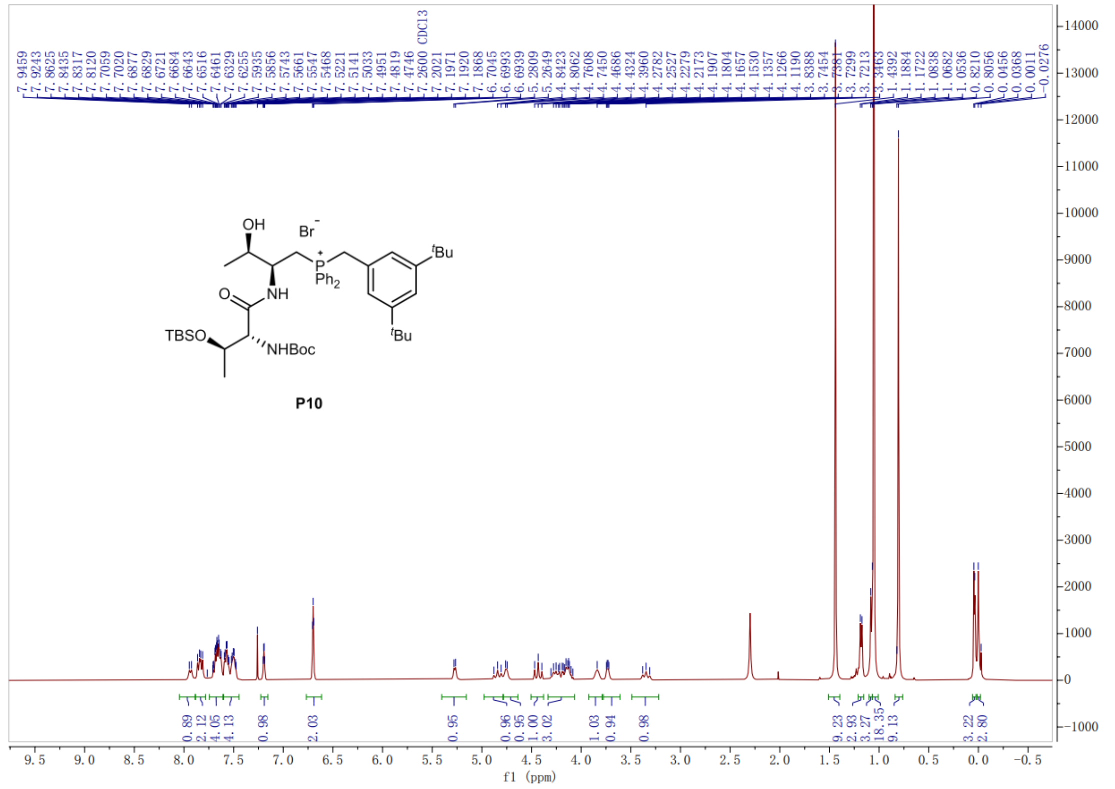

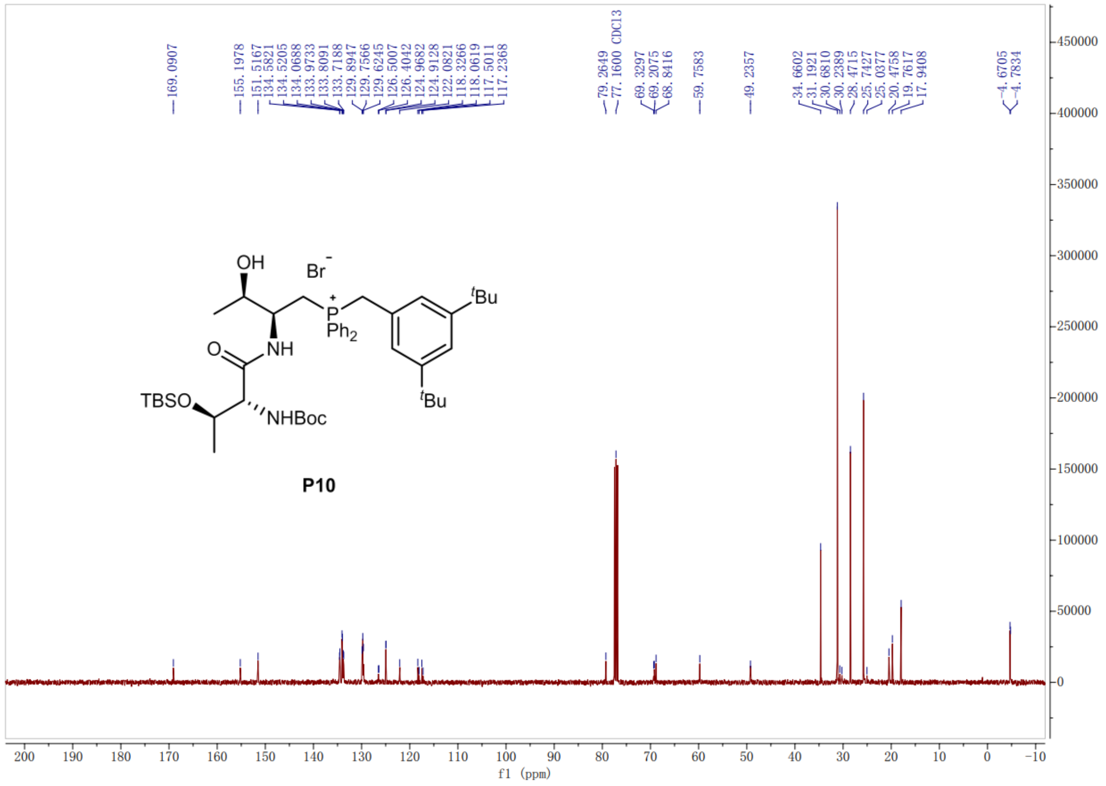
169.0907
155.1978
151.5167
134.5821
134.5205
134.0688
133.9733
133.8091
133.7188
129.8947
129.7566
129.6245
126.5007
126.4042
124.9682
124.9128
122.0821
118.3266
118.0619
117.5011
117.2368
79.2649
77.1600 CDCl3
69.3297
69.2075
68.8416
59.7583
49.2357
34.6602
31.1921
30.6810
30.2389
28.4715
25.7427
25.0377
20.4758
19.7617
17.9408
-4.6705
-4.7834
OH
Br-
+P
Ph2
tBu
tBu
O
NH
TBSO
NHBoc
P10
f1 (ppm)

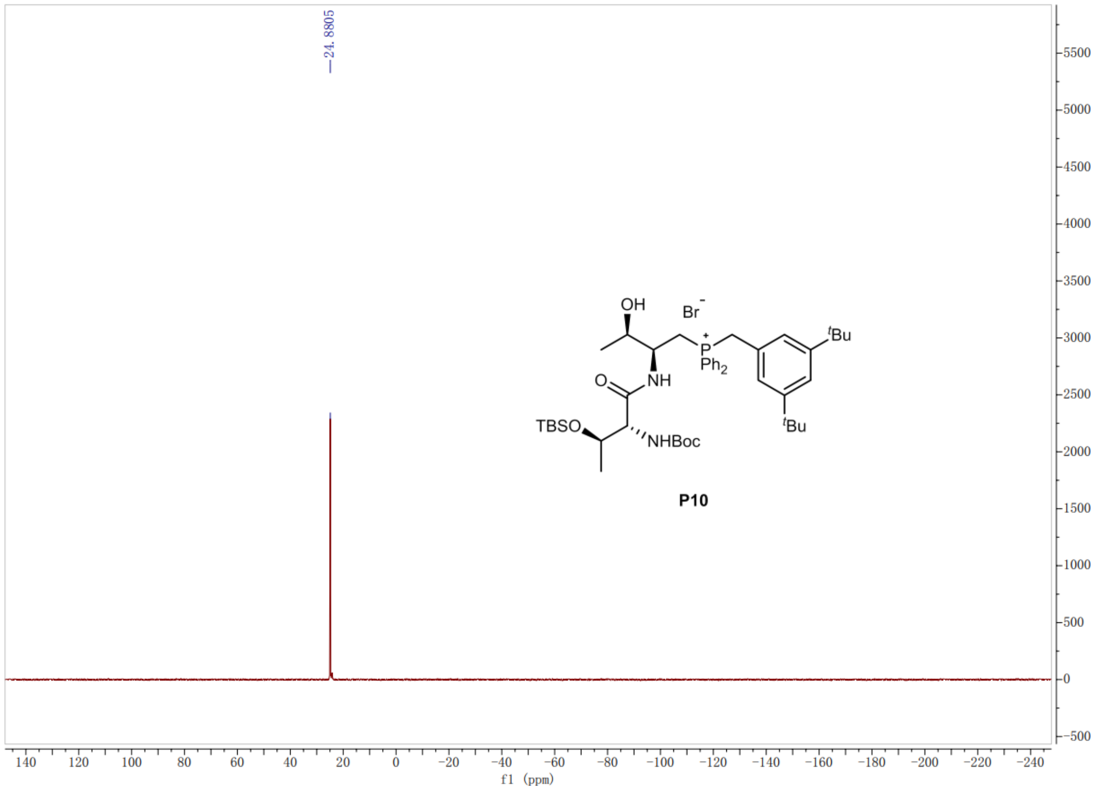
24.8805
OH
Br-
+P
Ph2
tBu
tBu
O
NH
TBSO
NHBoc
P10
f1 (ppm)

NMR of **P10-1** ($CDCl_3$)

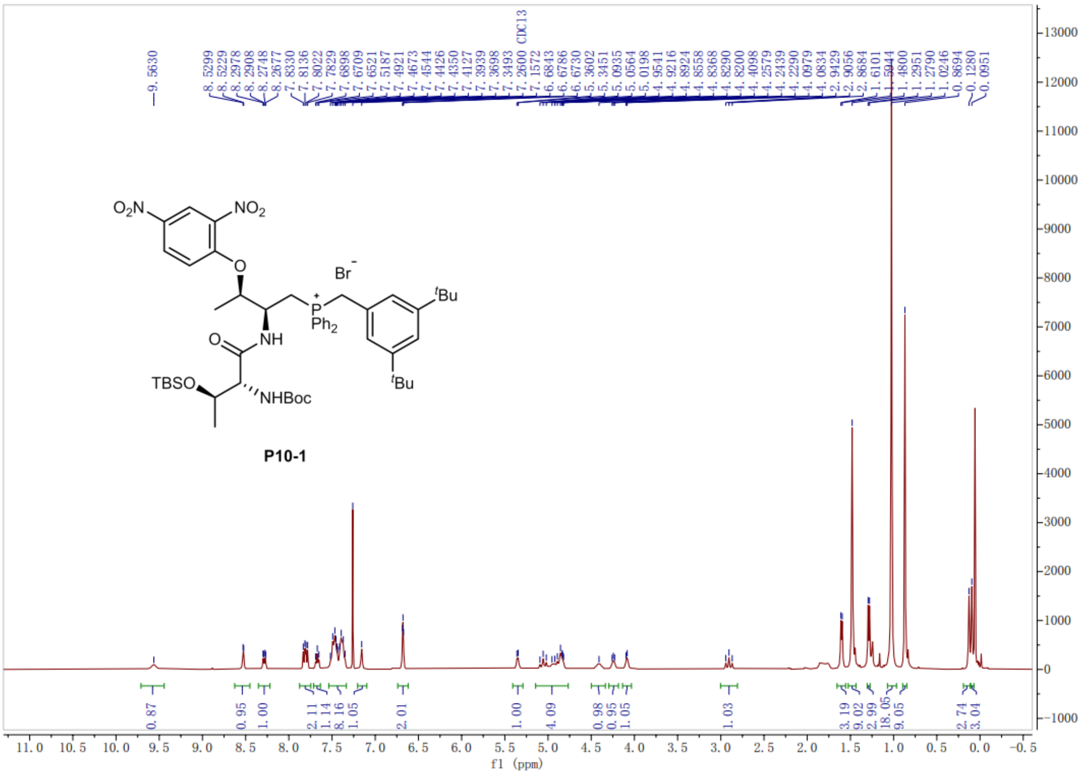

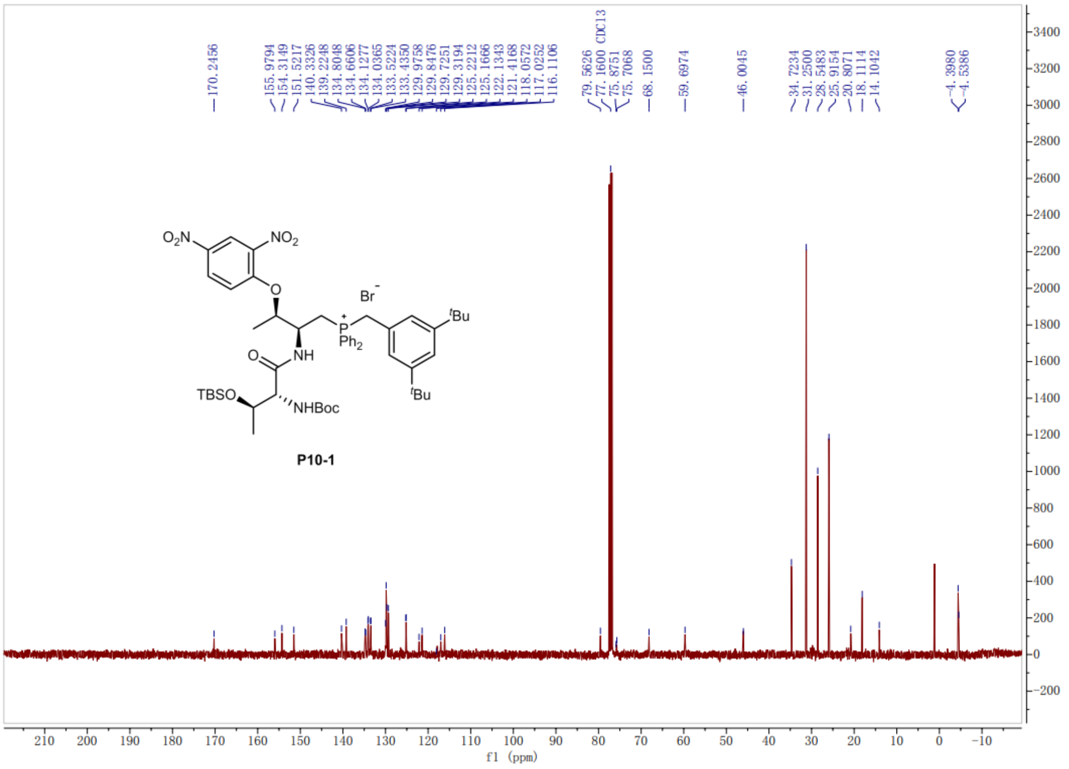

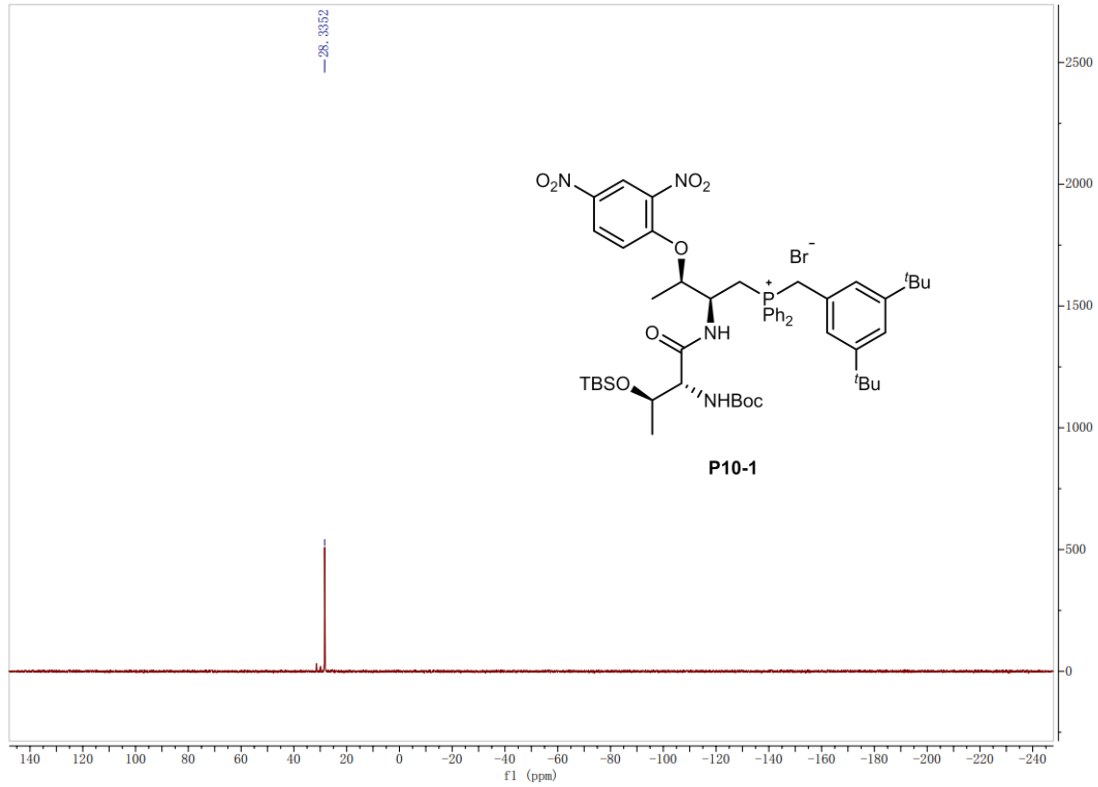

NMR of **P10-2** ($CDCl_3$)

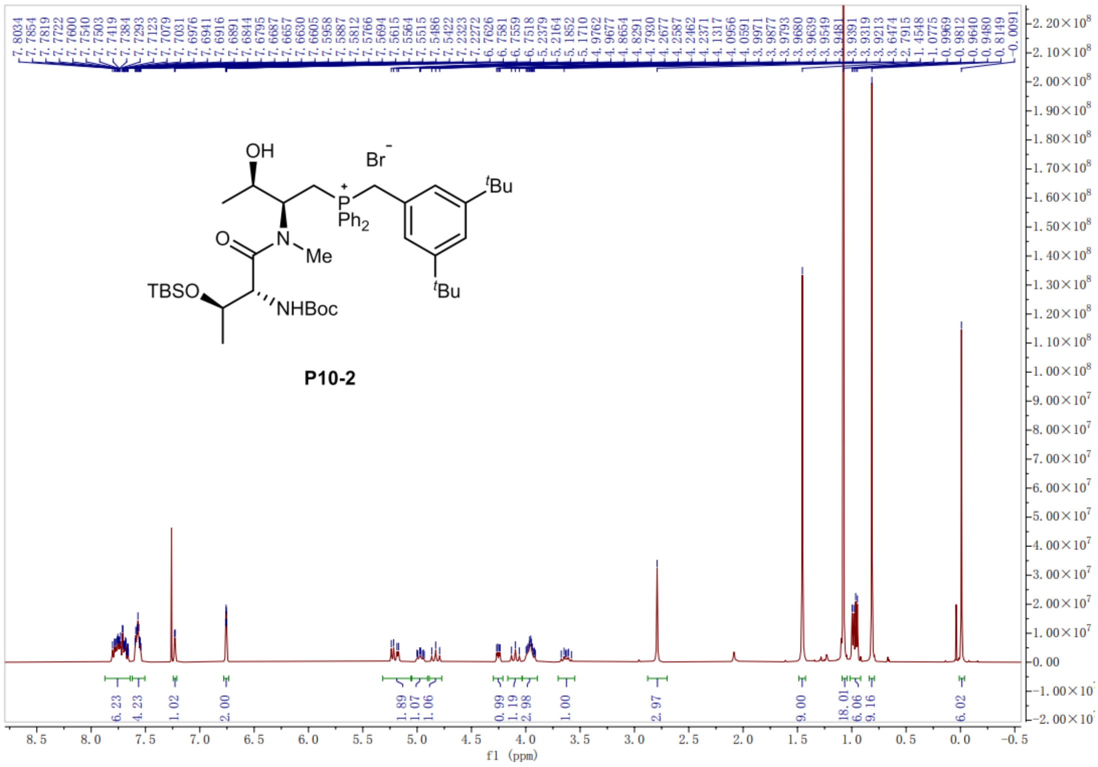

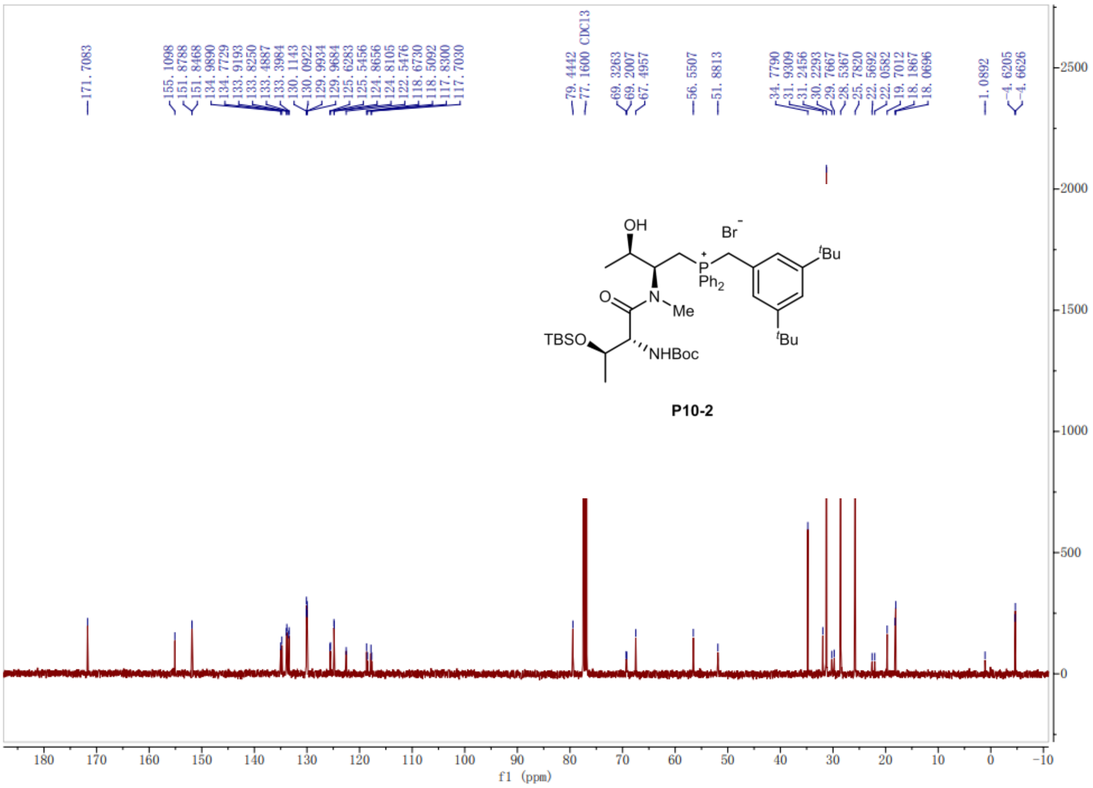
171.7083
155.1098
151.8788
151.8468
134.9890
134.7729
133.9193
133.8250
133.4887
133.3984
130.1143
130.0922
129.9934
129.9684
125.6283
125.5456
124.8656
124.8105
122.5476
118.6730
118.5092
117.8300
117.7030
79.4442
77.1600 CDCl3
69.3263
69.2007
67.4957
56.5507
51.8813
34.7790
31.9309
31.2456
30.2293
29.7667
28.5367
25.7820
22.5692
22.0582
19.7012
18.1867
18.0696
1.0892
-4.6205
-4.6626
OH
Br⁻
P
Ph2
tBu
tBu
O
N
Me
TBSO
NHBoc
P10-2
f1 (ppm)

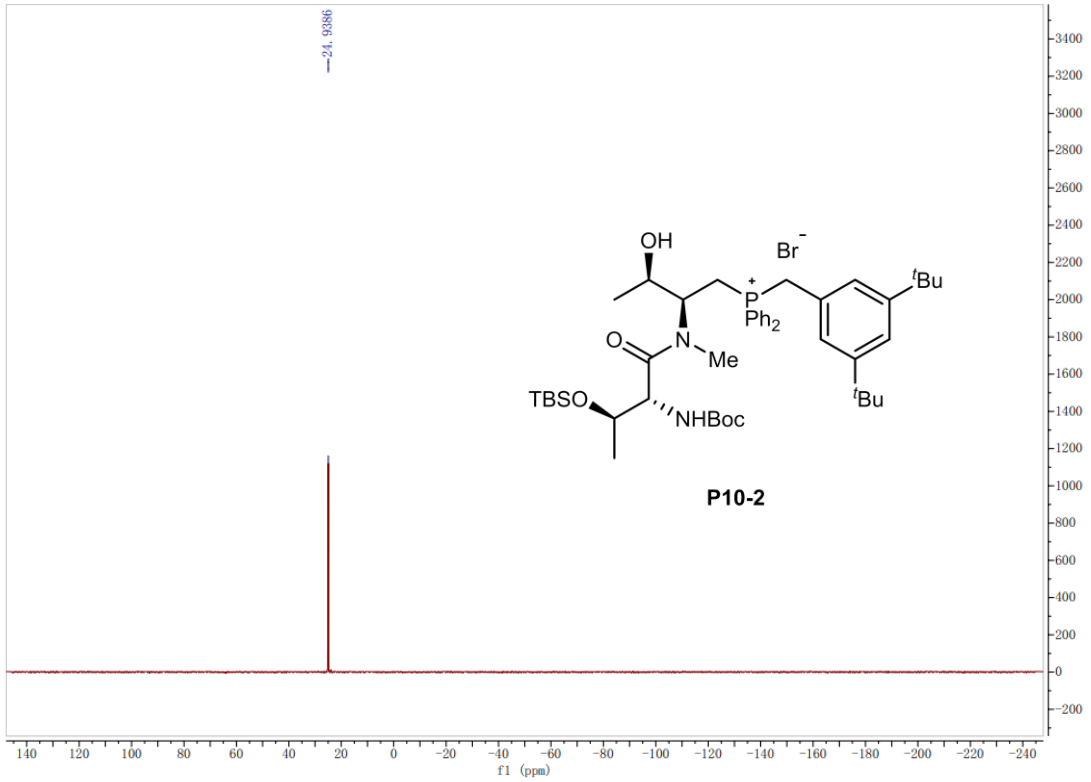
24.9386
OH
Br⁻
P
Ph2
tBu
tBu
O
N
Me
TBSO
NHBoc
P10-2
f1 (ppm)

NMR of **P10-3** ($CDCl_3$)

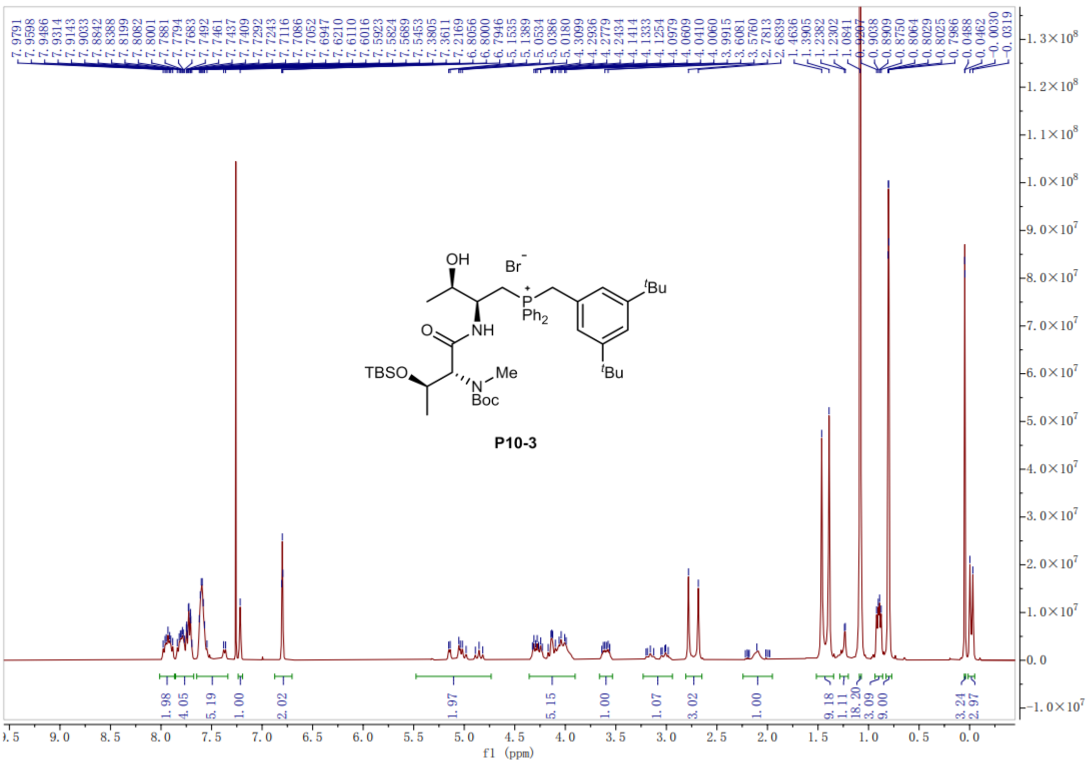

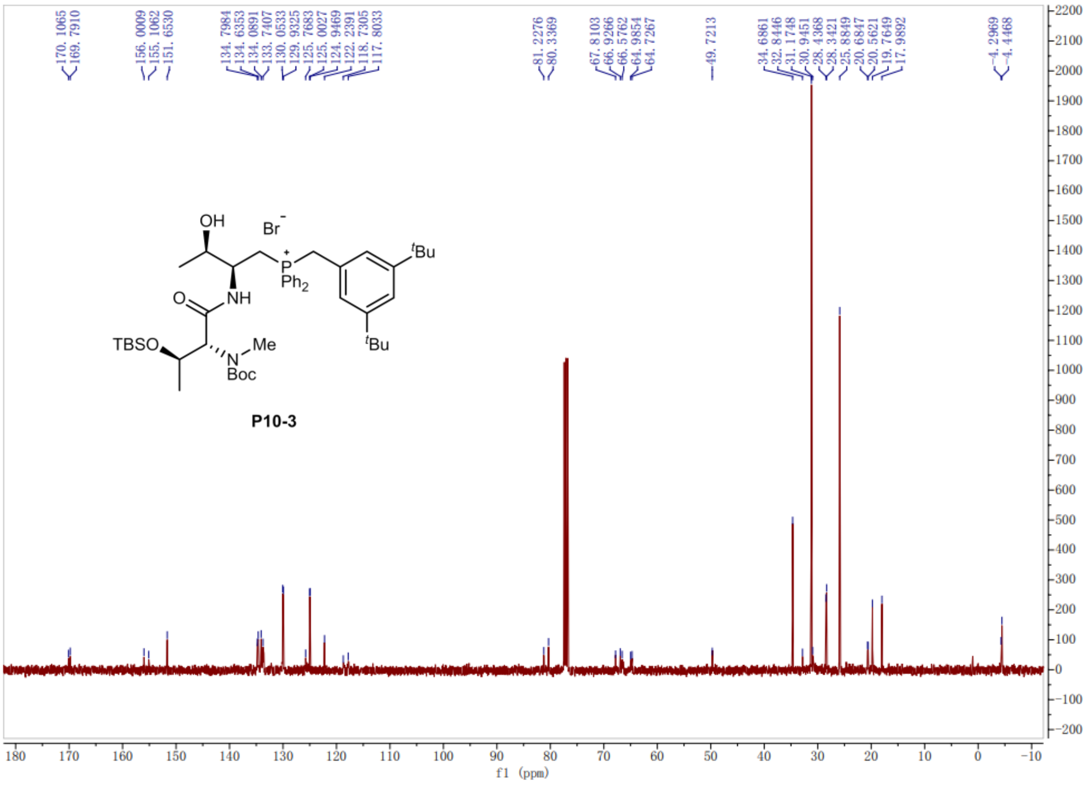

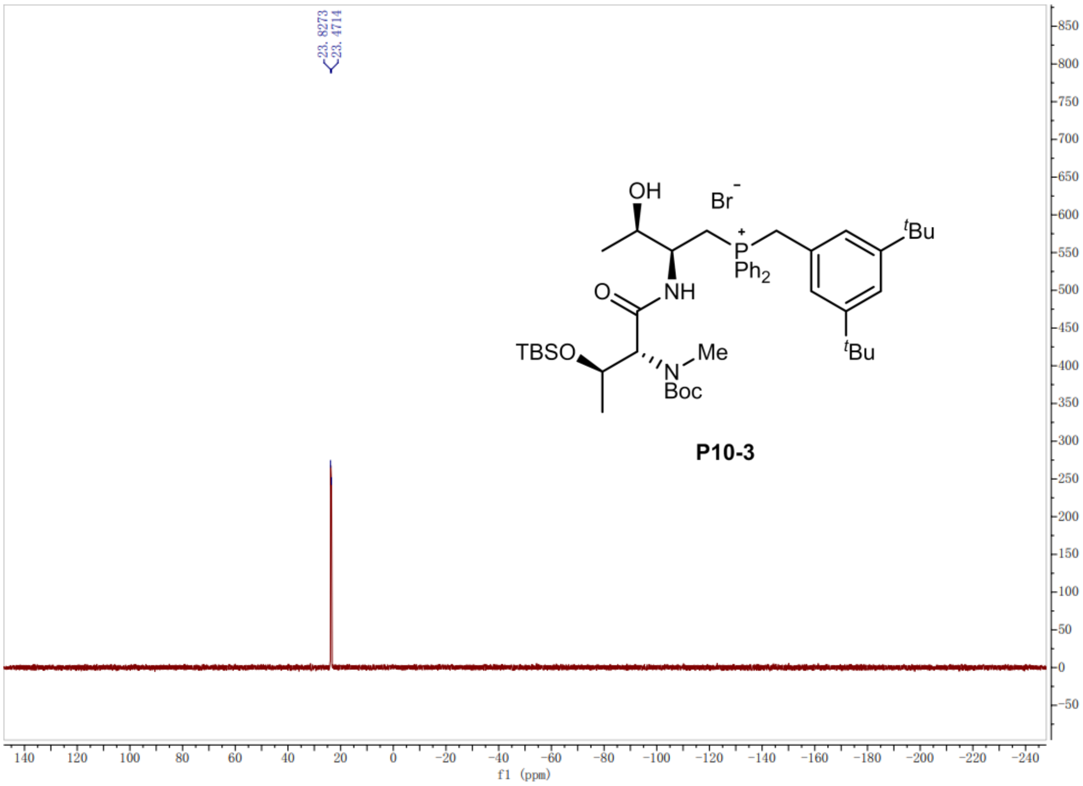

NMR of **3** ($CDCl_3$)

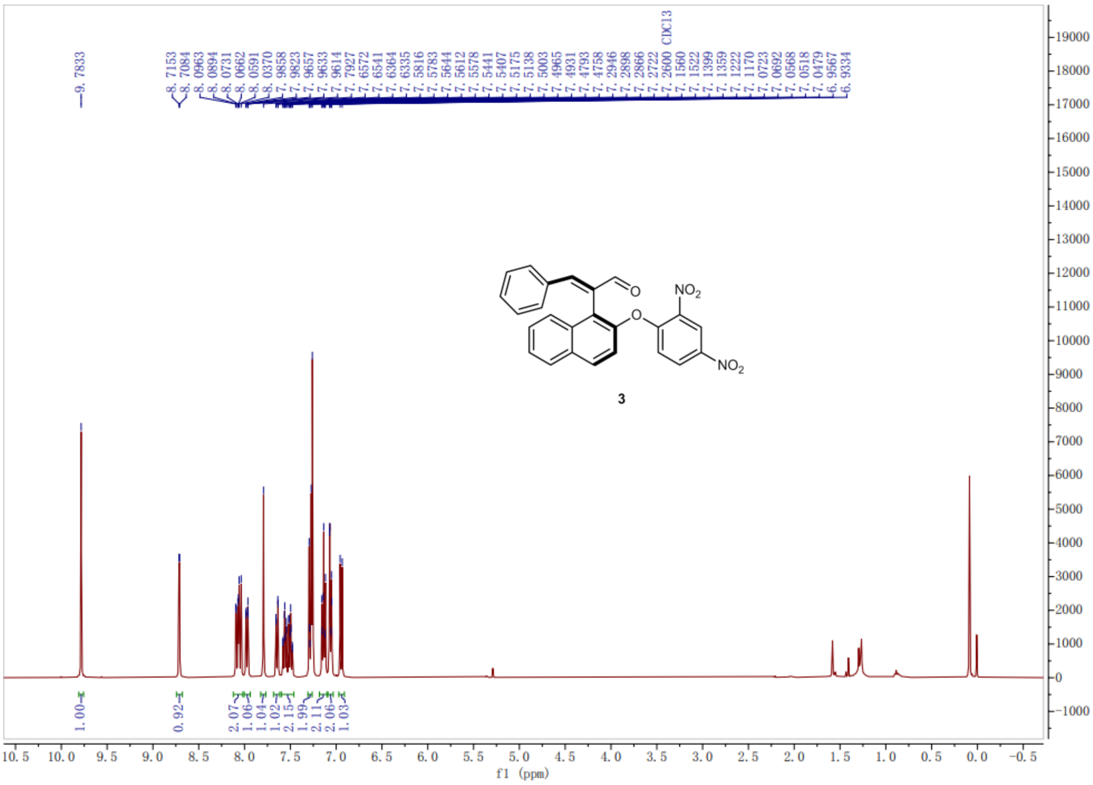

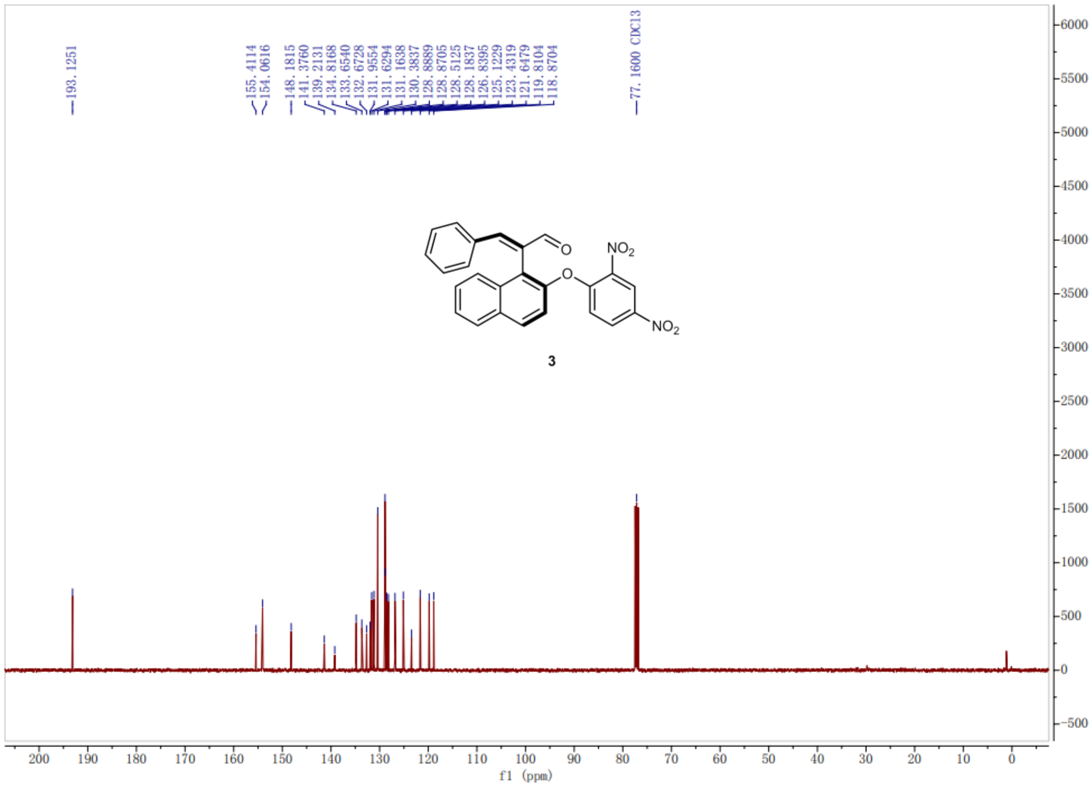

3

NMR of **4** ($CDCl_3$)

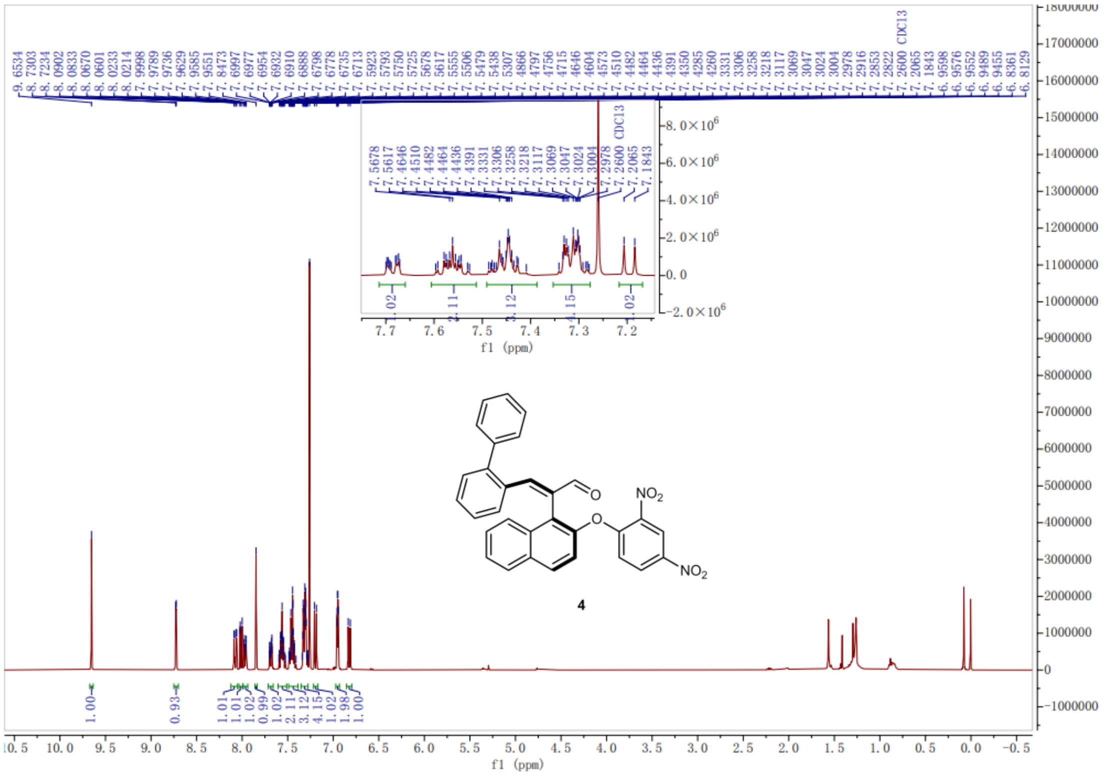

4

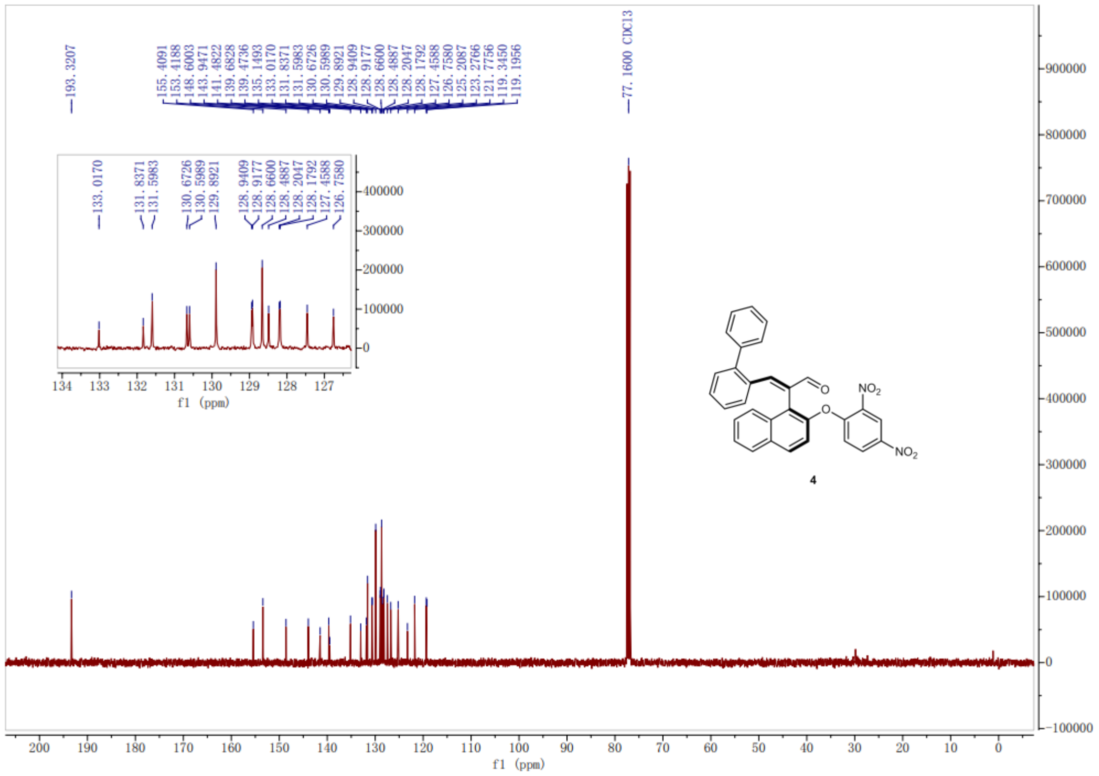

NMR of **5** ($CDCl_3$)

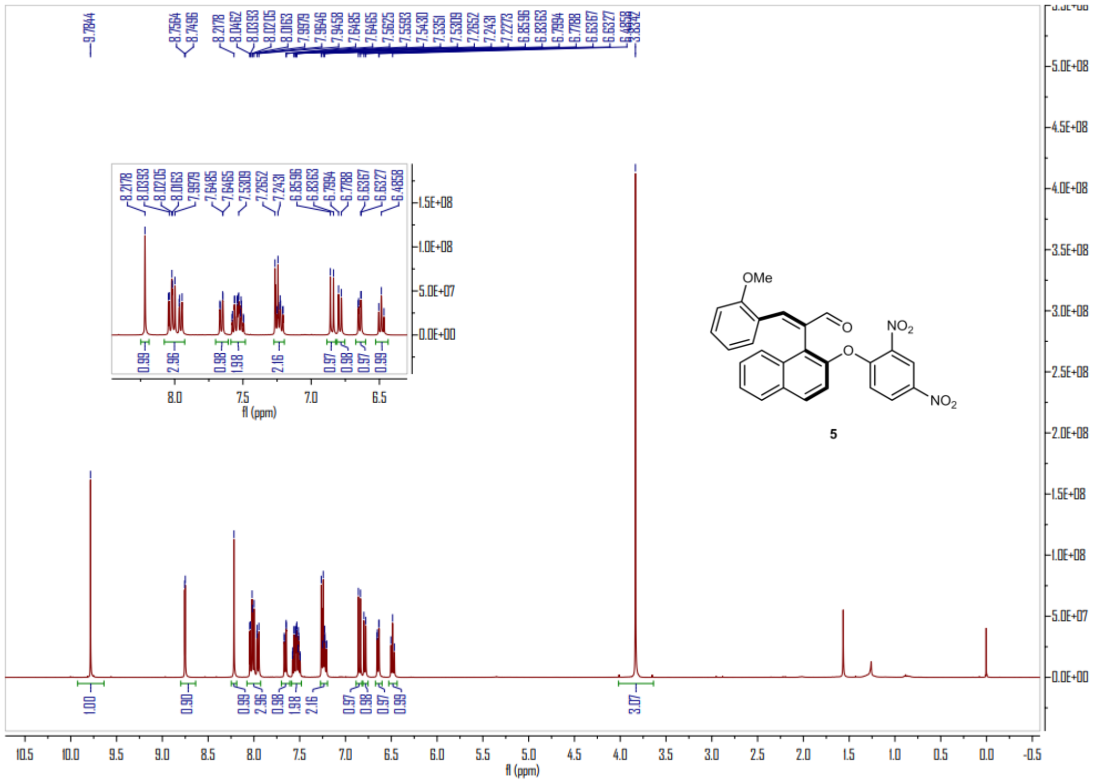

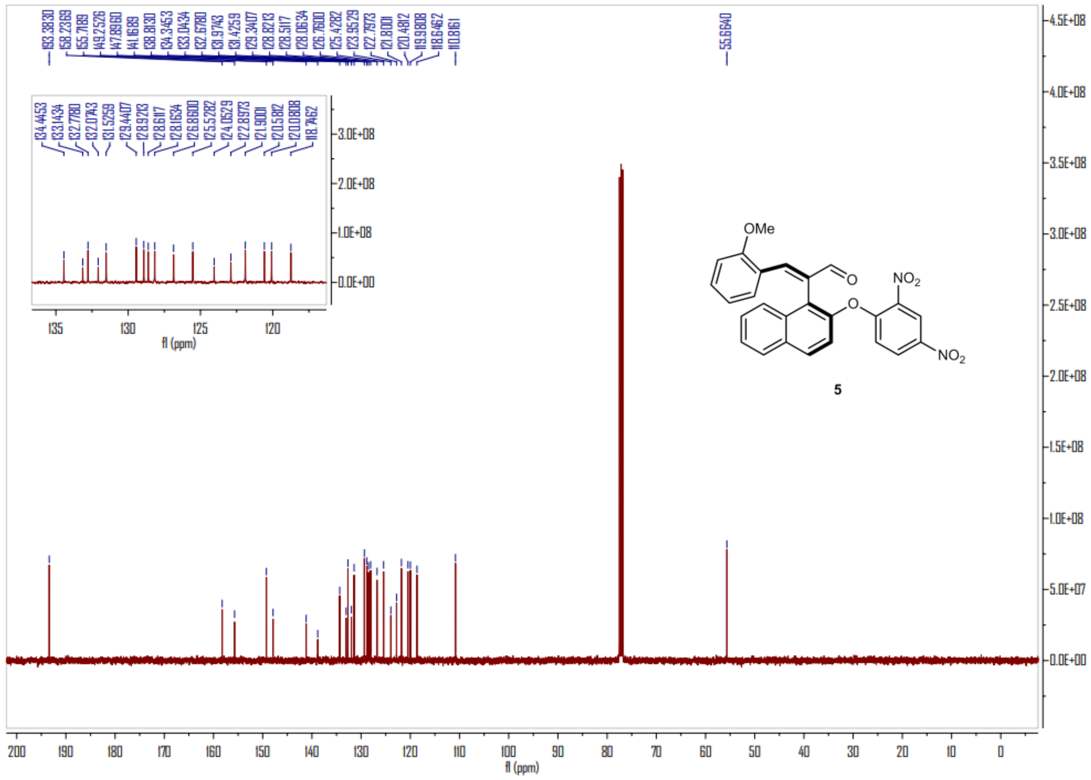

NMR of **6** ($CDCl_3$)

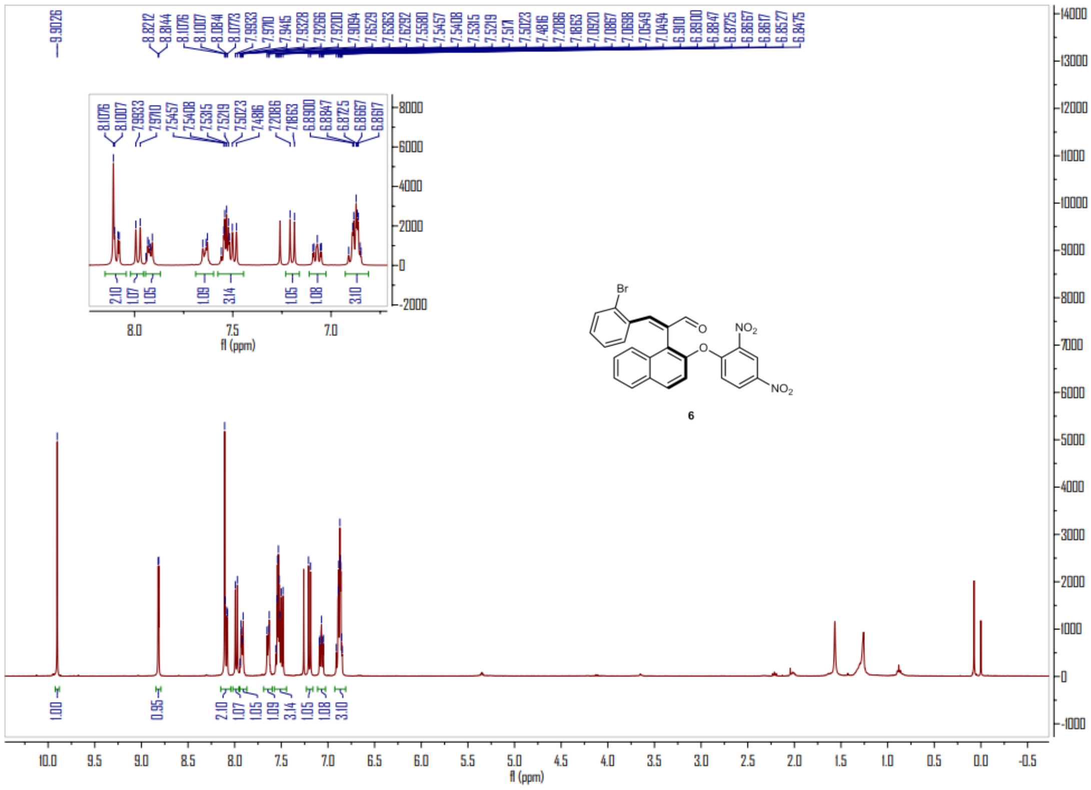

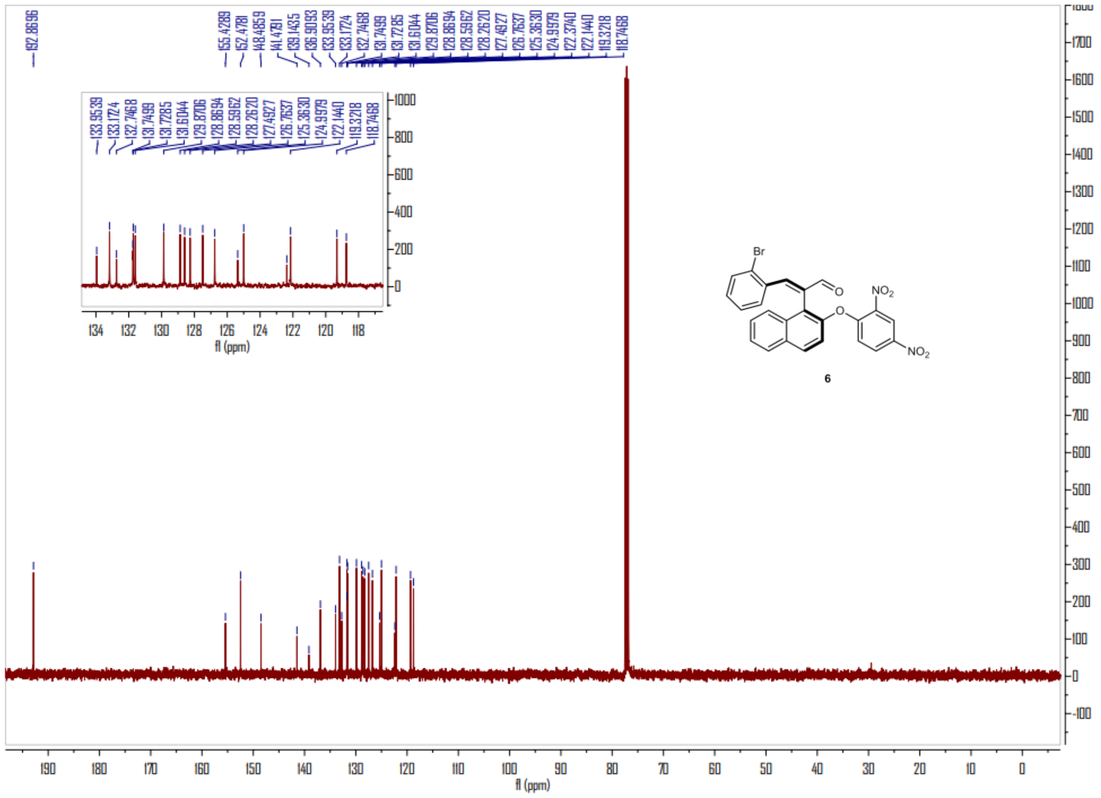

NMR of **7** ($CDCl_3$)

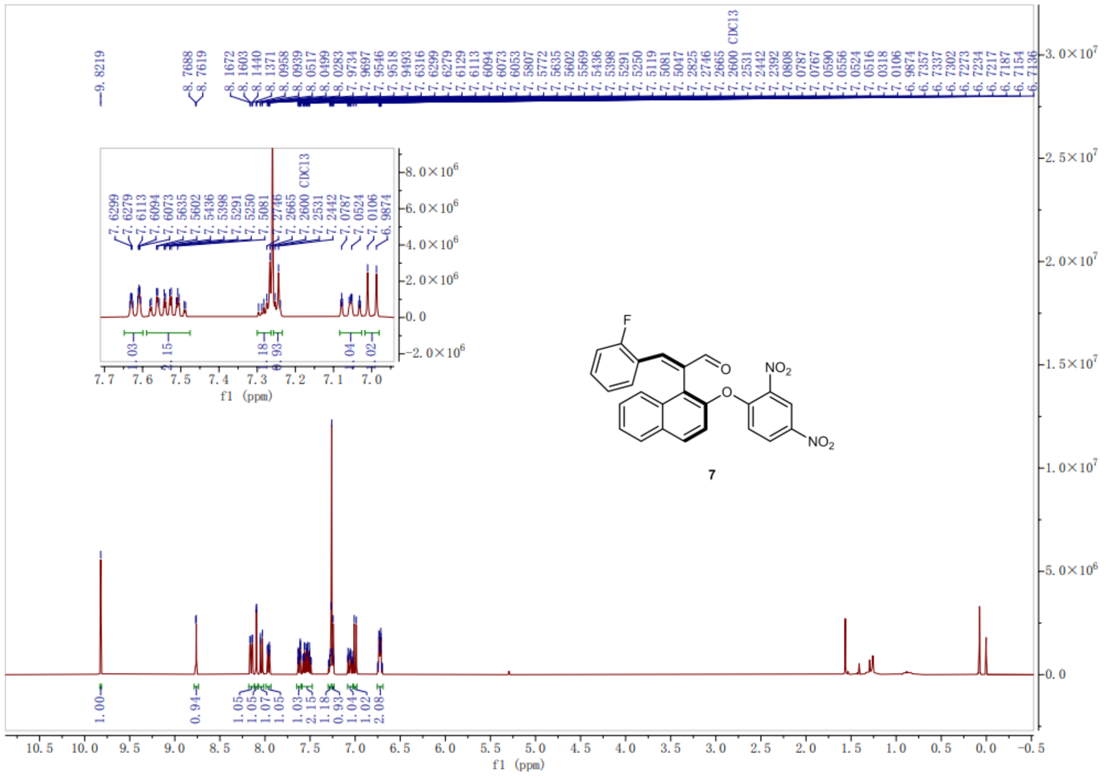

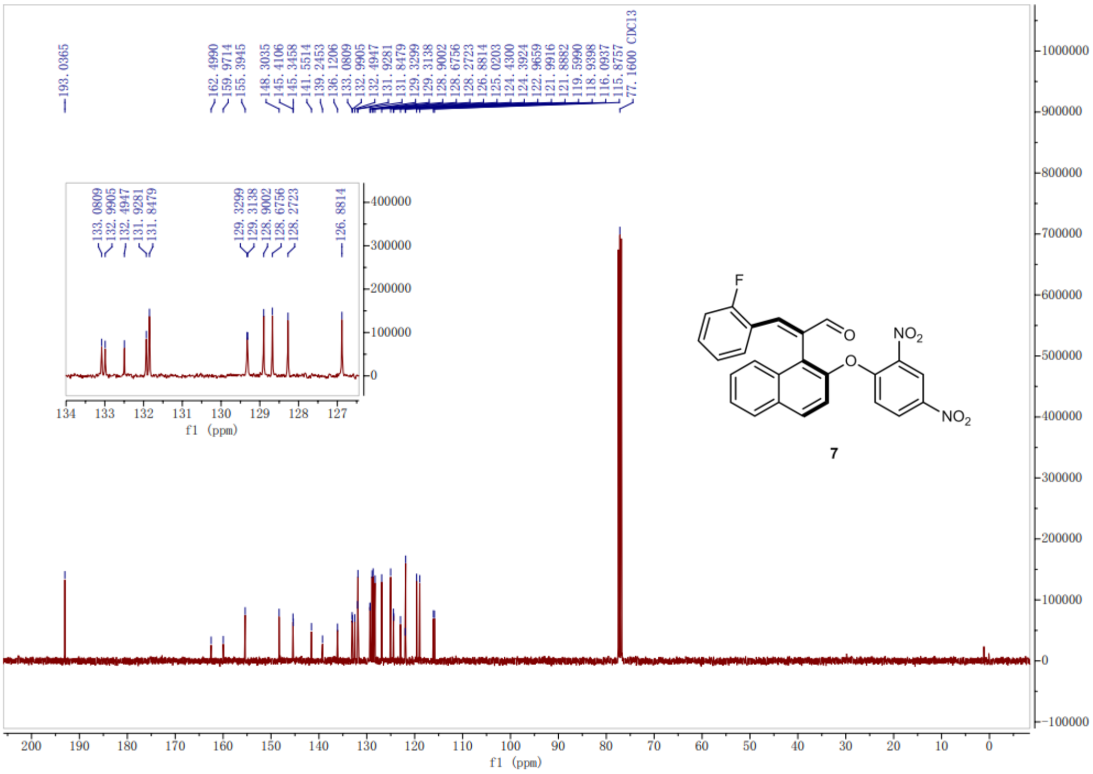
193.0365
162.4990
159.9714
155.3945
148.3035
145.4106
145.3458
141.5514
139.2453
136.1206
133.0809
132.9905
132.4947
131.9281
131.8479
129.3299
129.3138
128.9002
128.6756
128.2723
126.8814
125.0203
124.4300
124.3924
122.9659
121.9916
121.8882
119.5990
118.9398
116.0937
115.8757
77.1600 CDCl3
F
NO2
O
NO2
7
f1 (ppm)

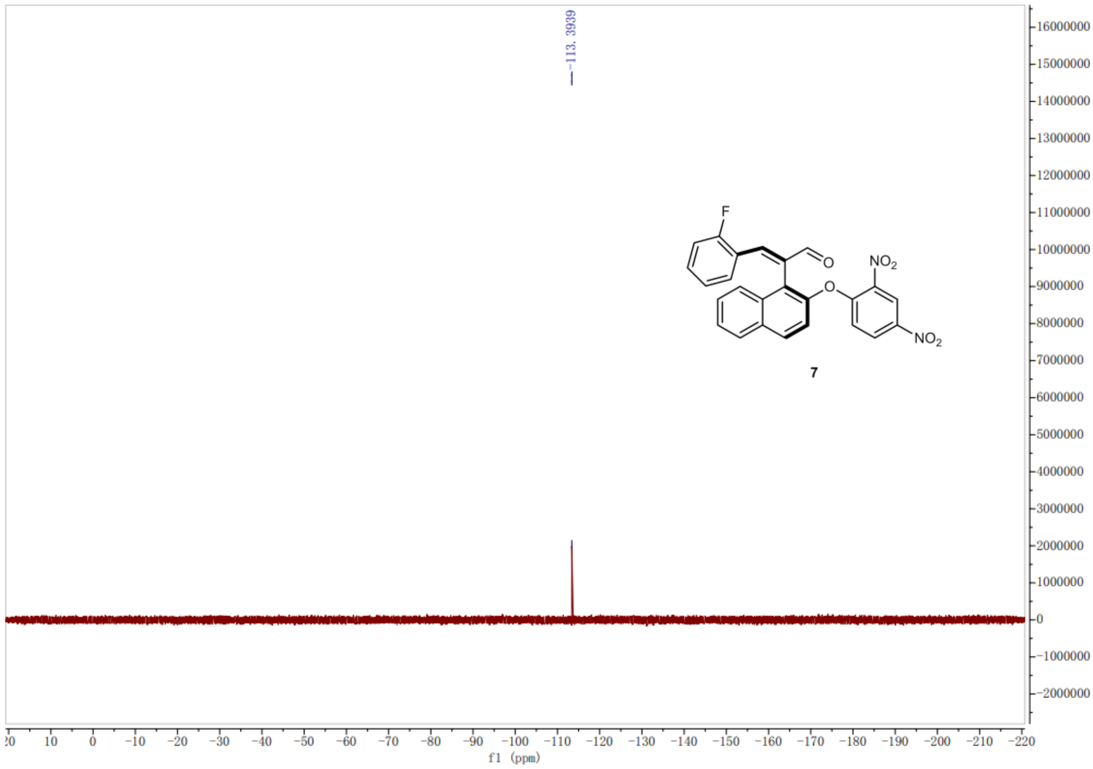
-113.3939
F
NO2
O
NO2
7
f1 (ppm)

NMR of **8** ($CDCl_3$)

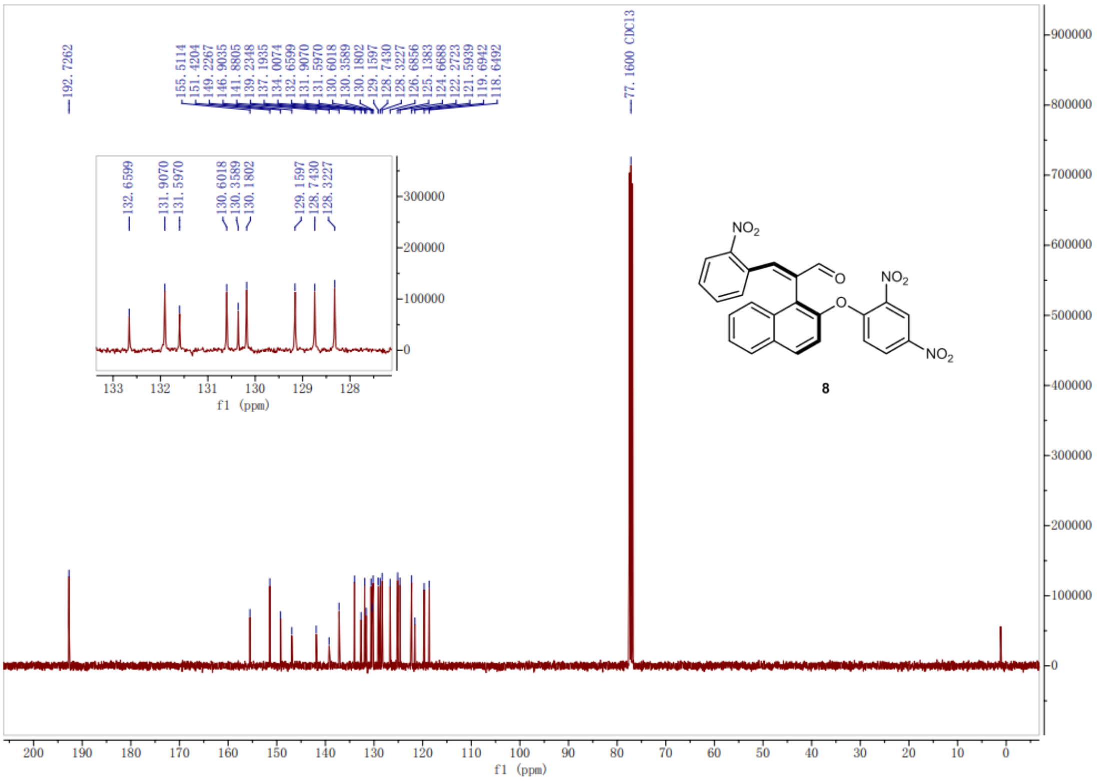

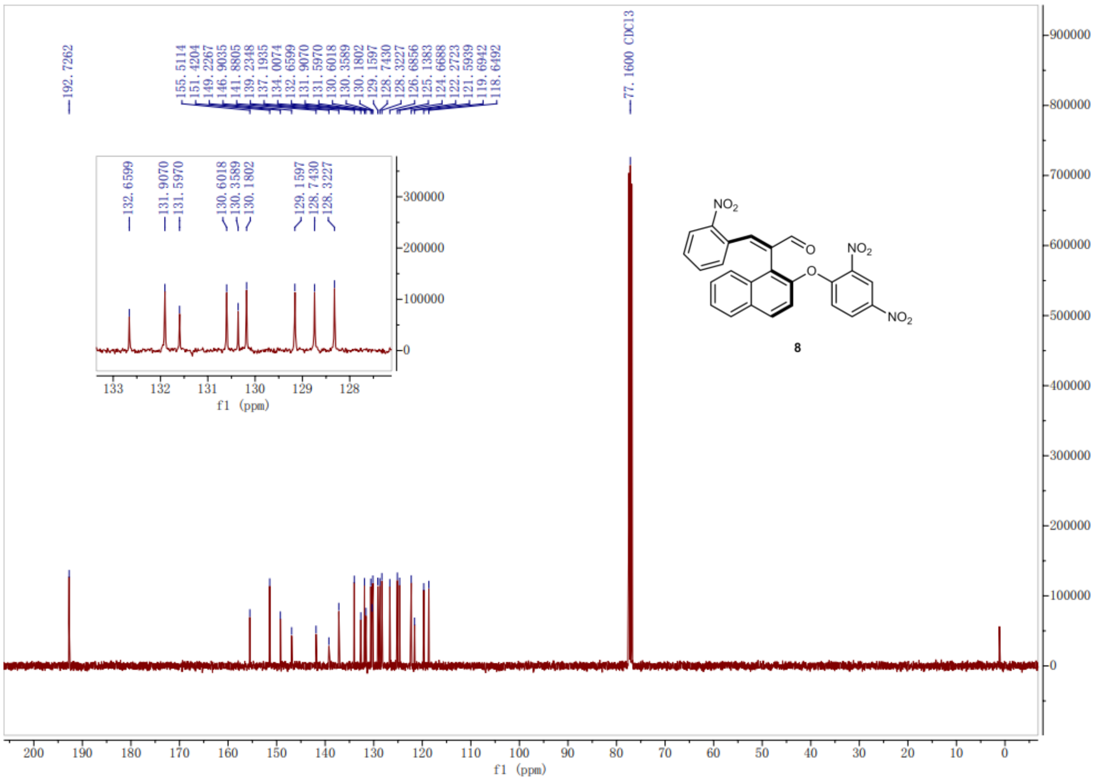

NMR of **9** ($CDCl_3$)

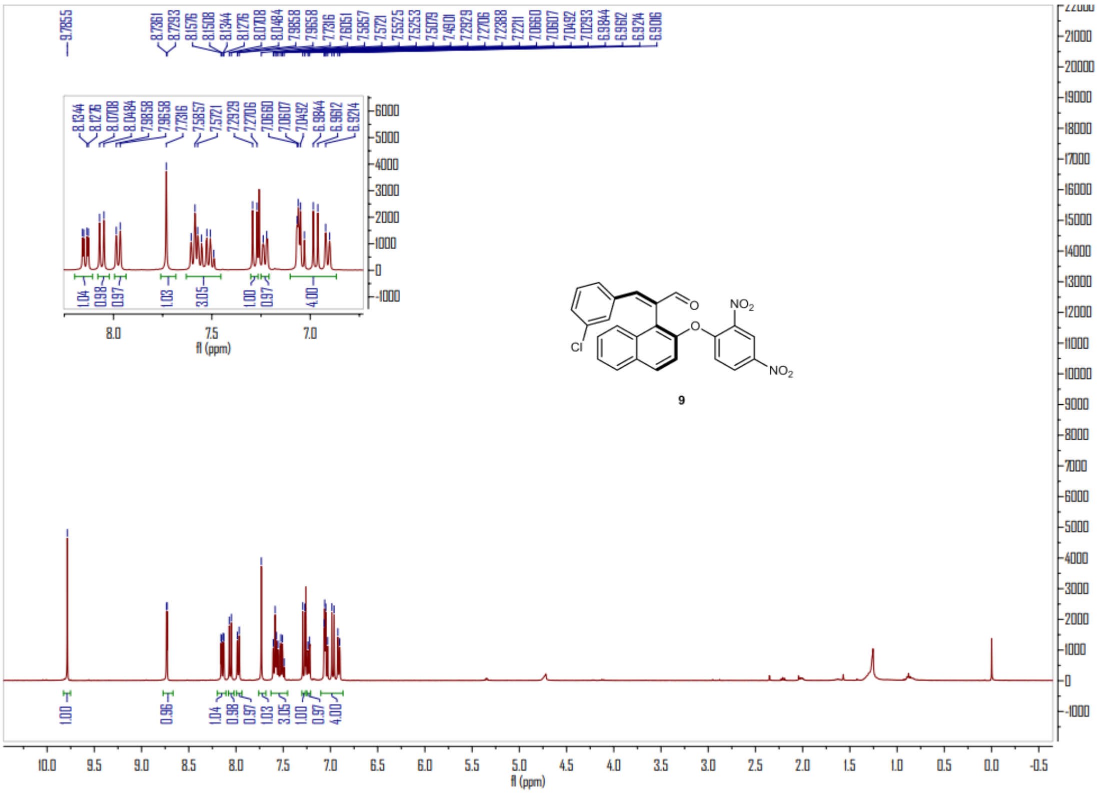

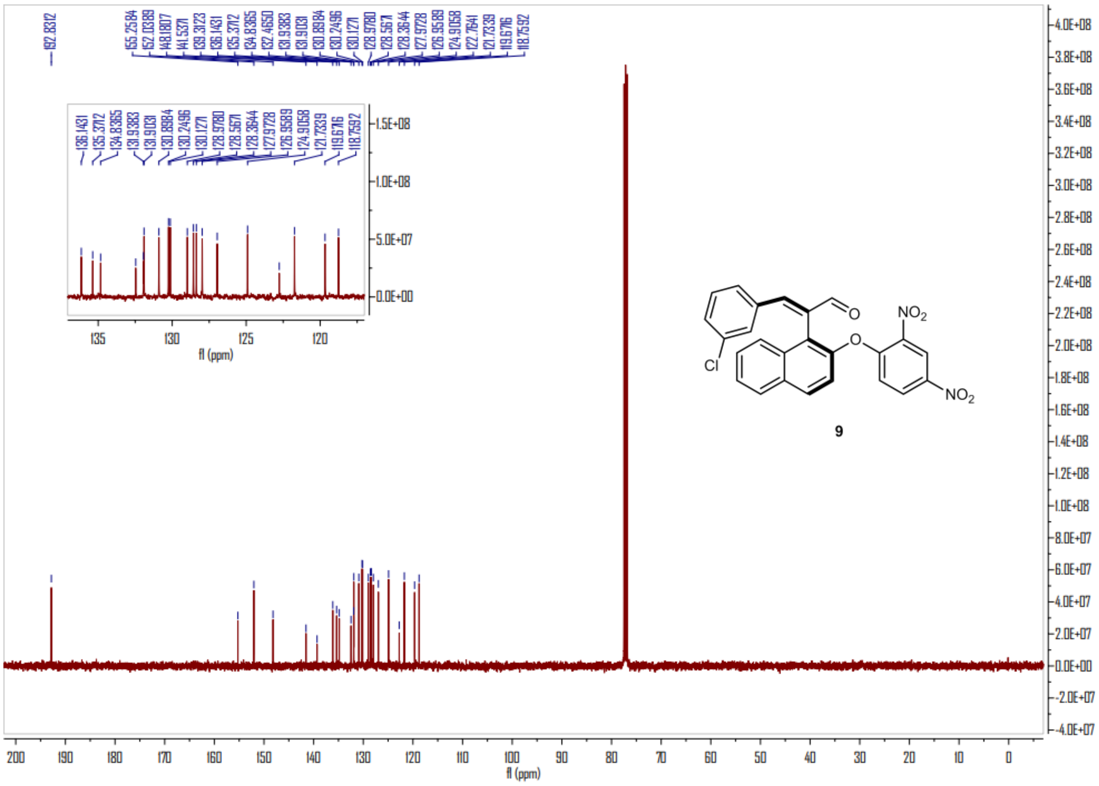

NMR of **10** ($CDCl_3$)

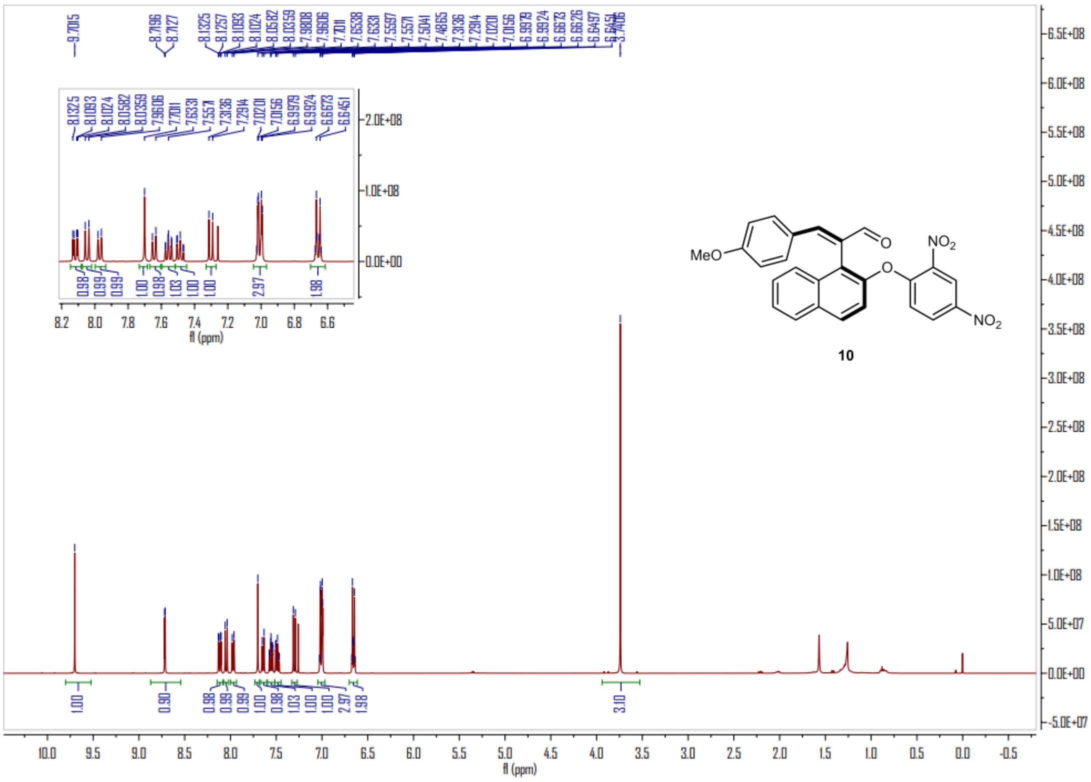

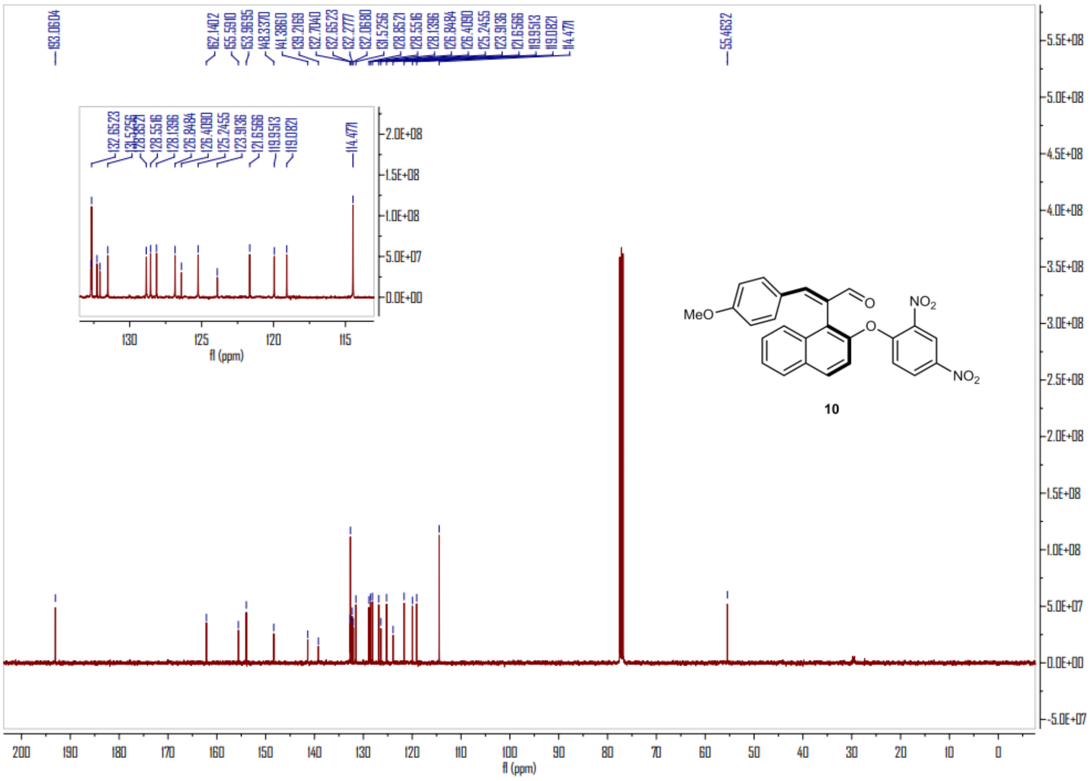

NMR of **11** ($CDCl_3$)

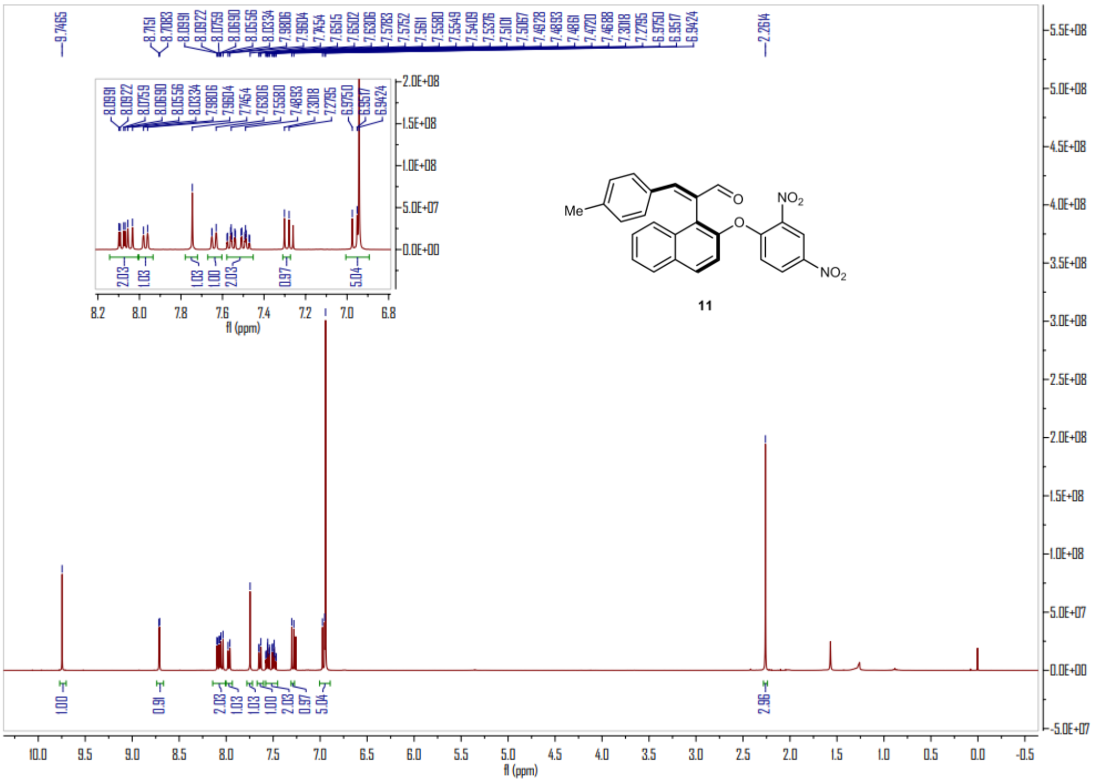

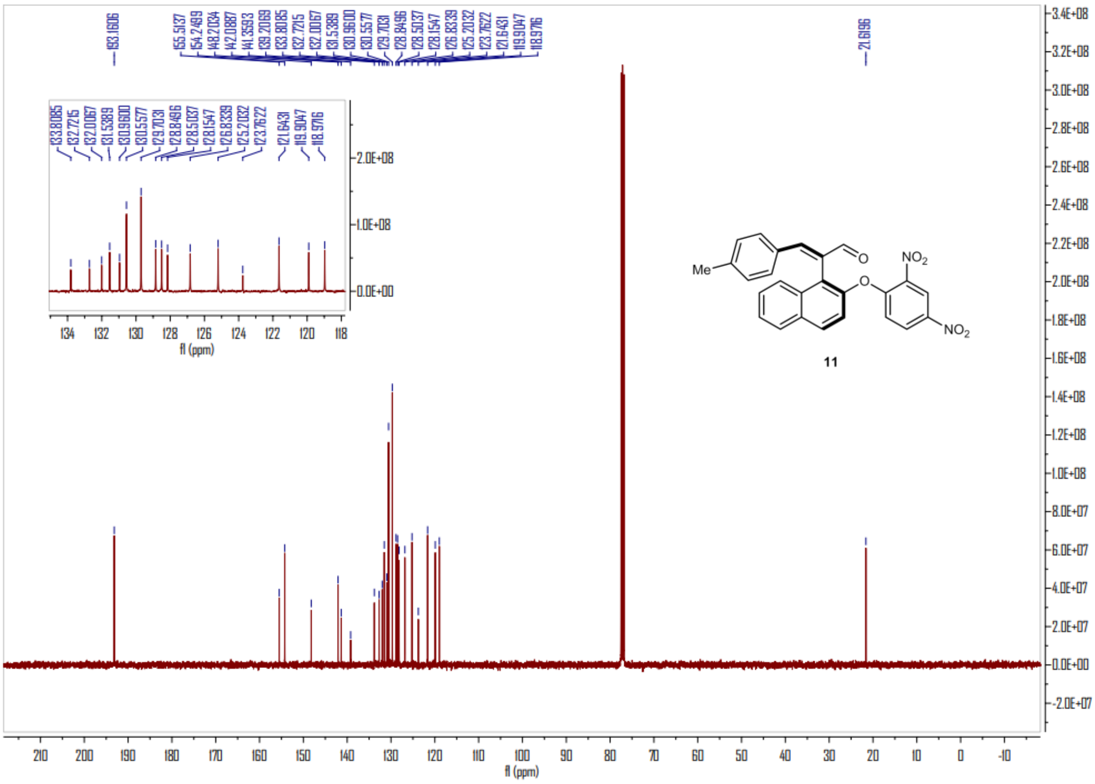

NMR of **12** ($CDCl_3$)

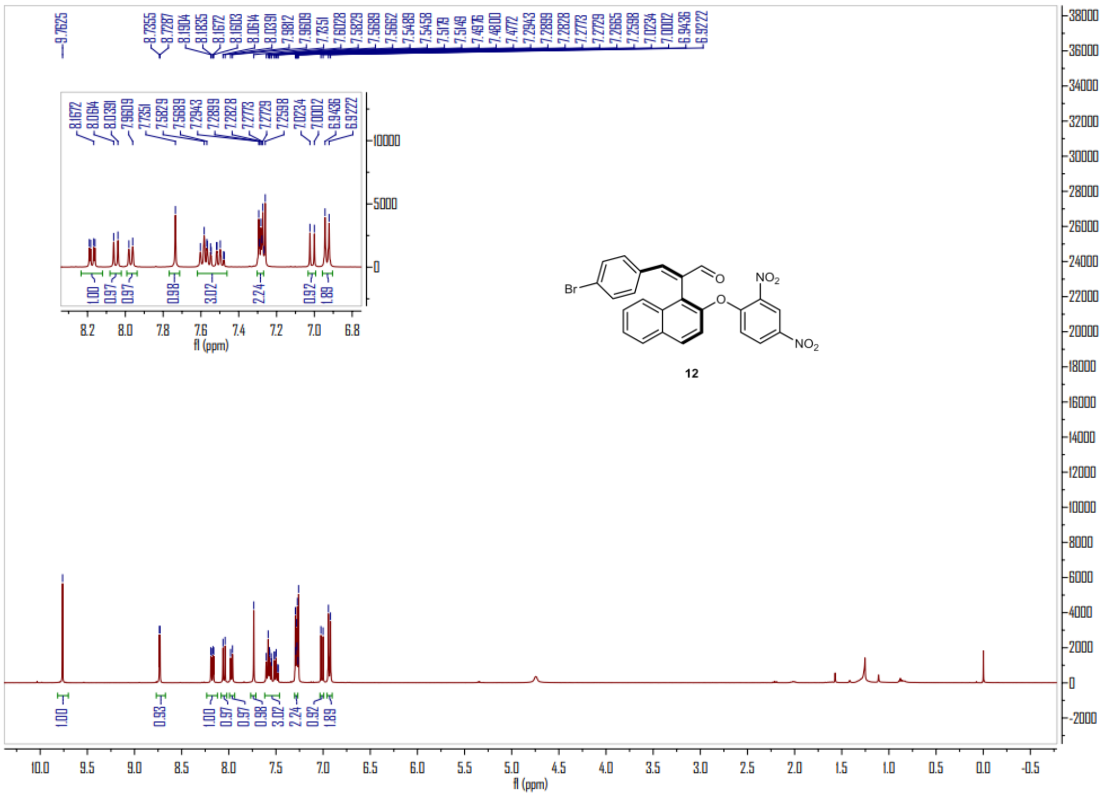

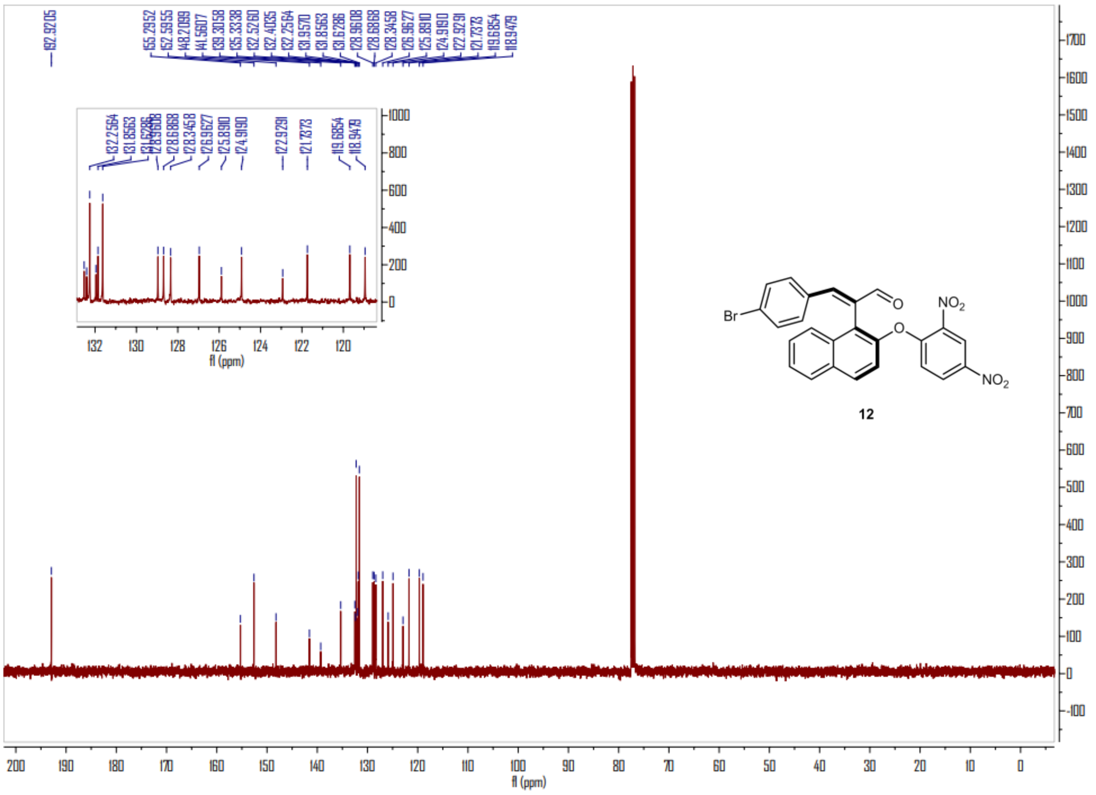

NMR of **13** ($CDCl_3$)

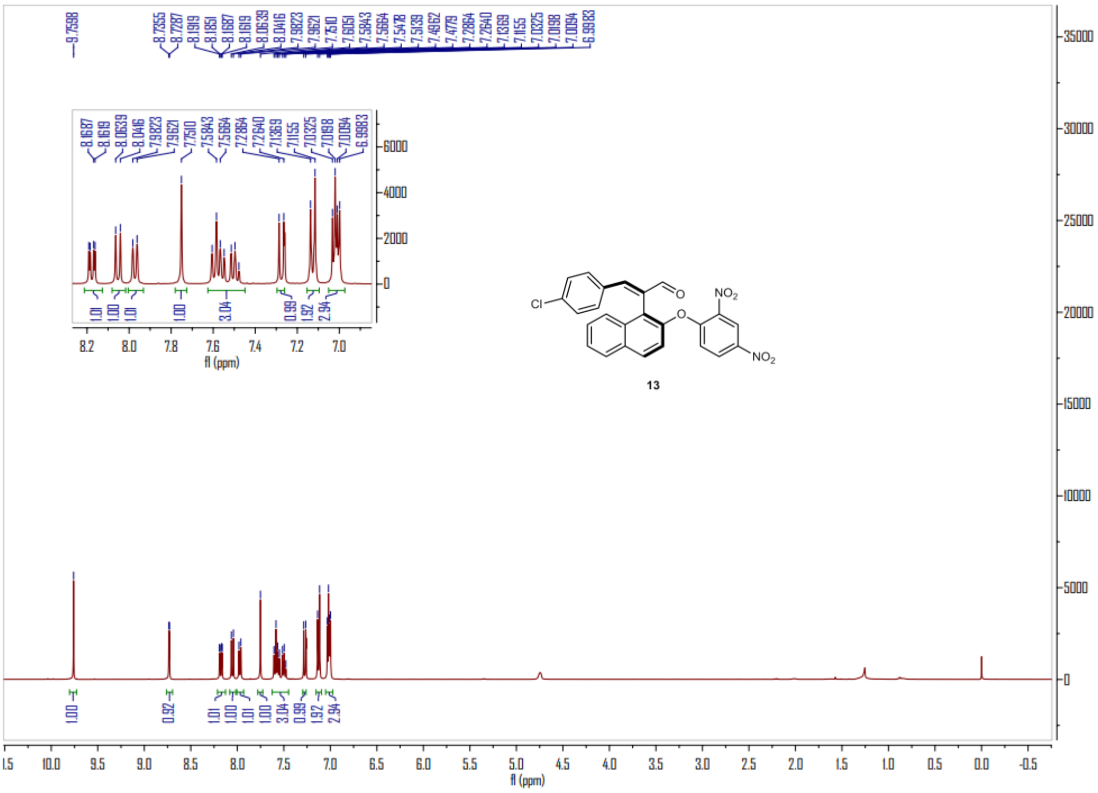

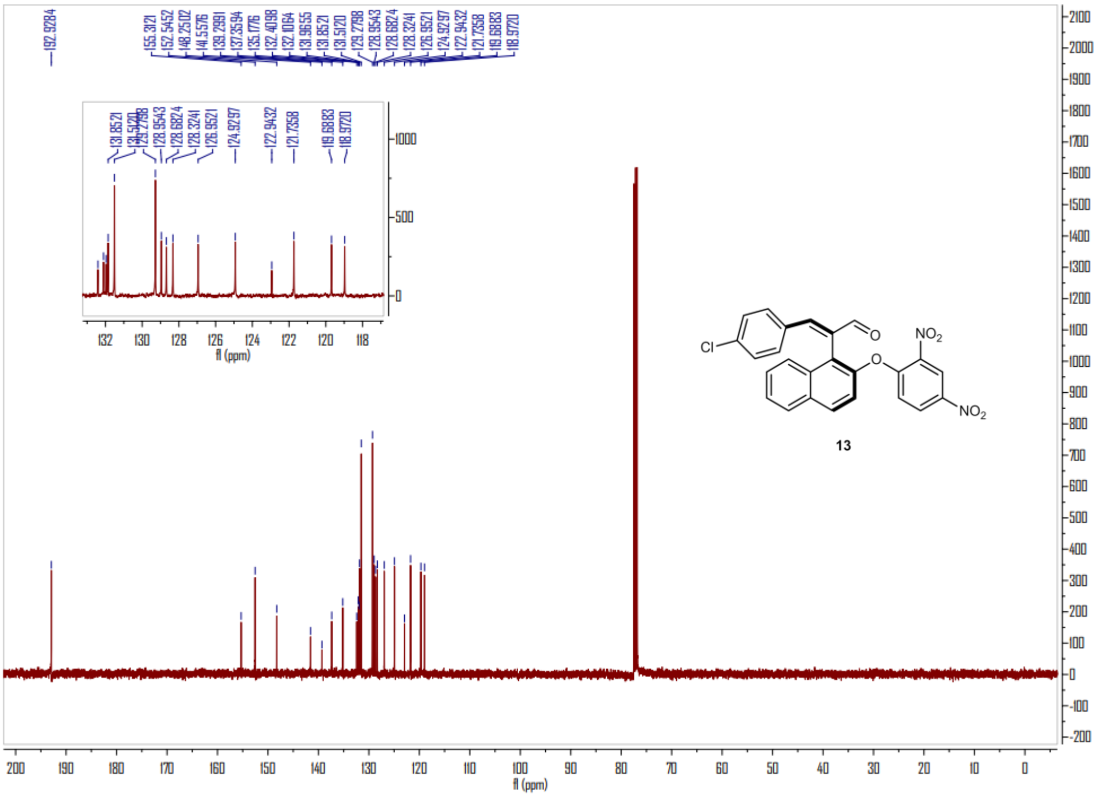

NMR of **14** ($CDCl_3$)

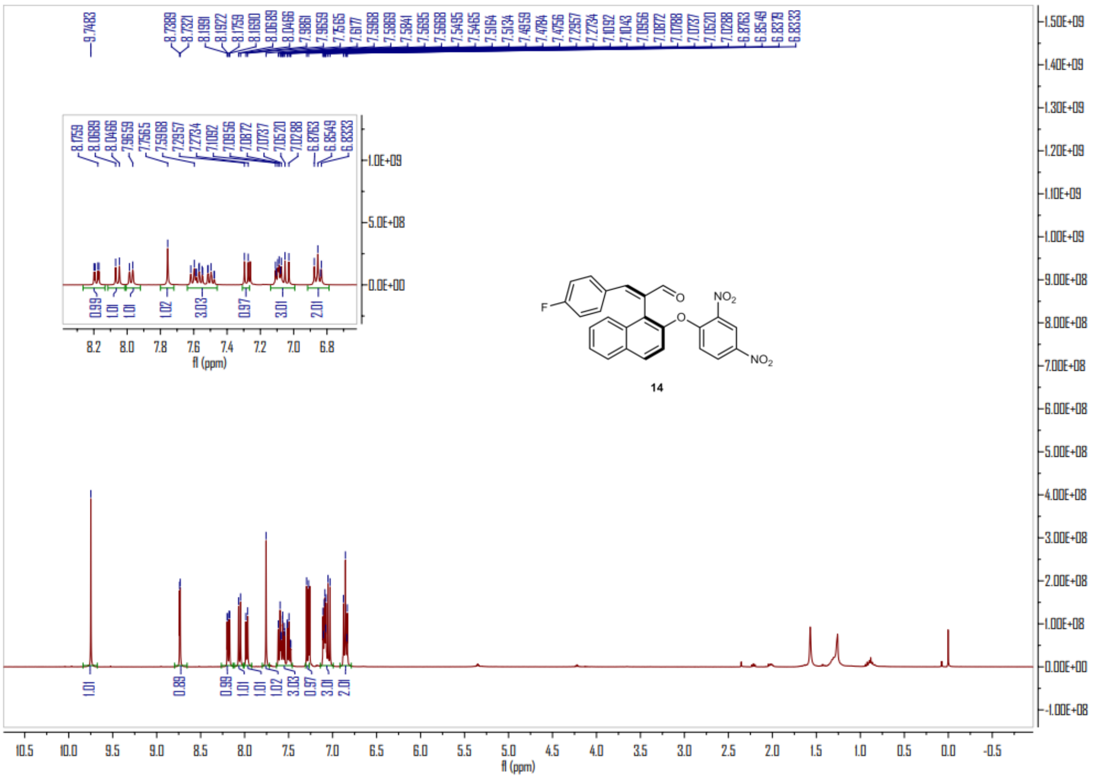

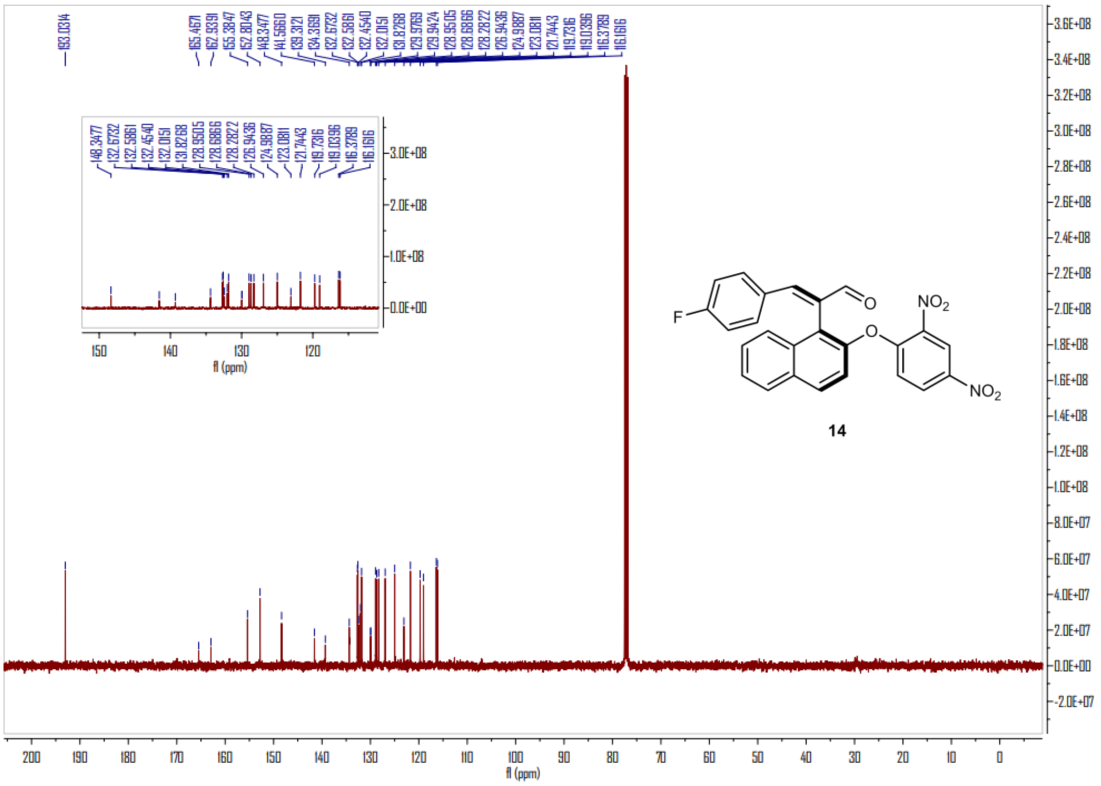

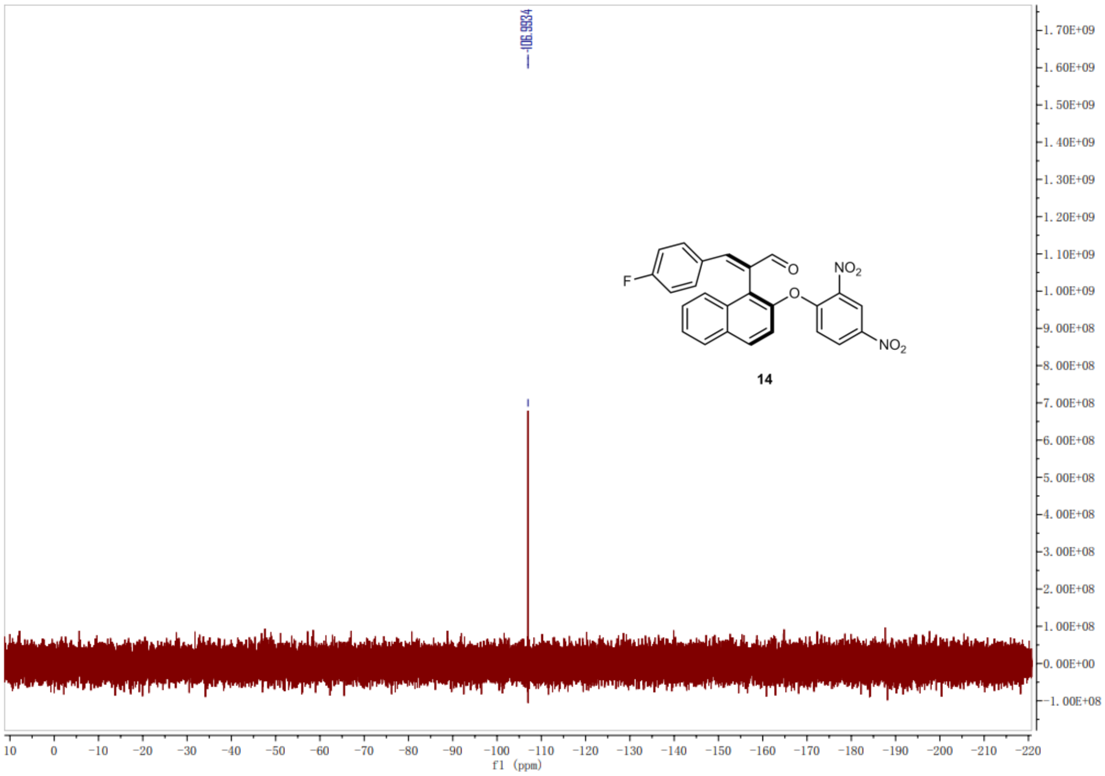

NMR of **15** ($CDCl_3$)

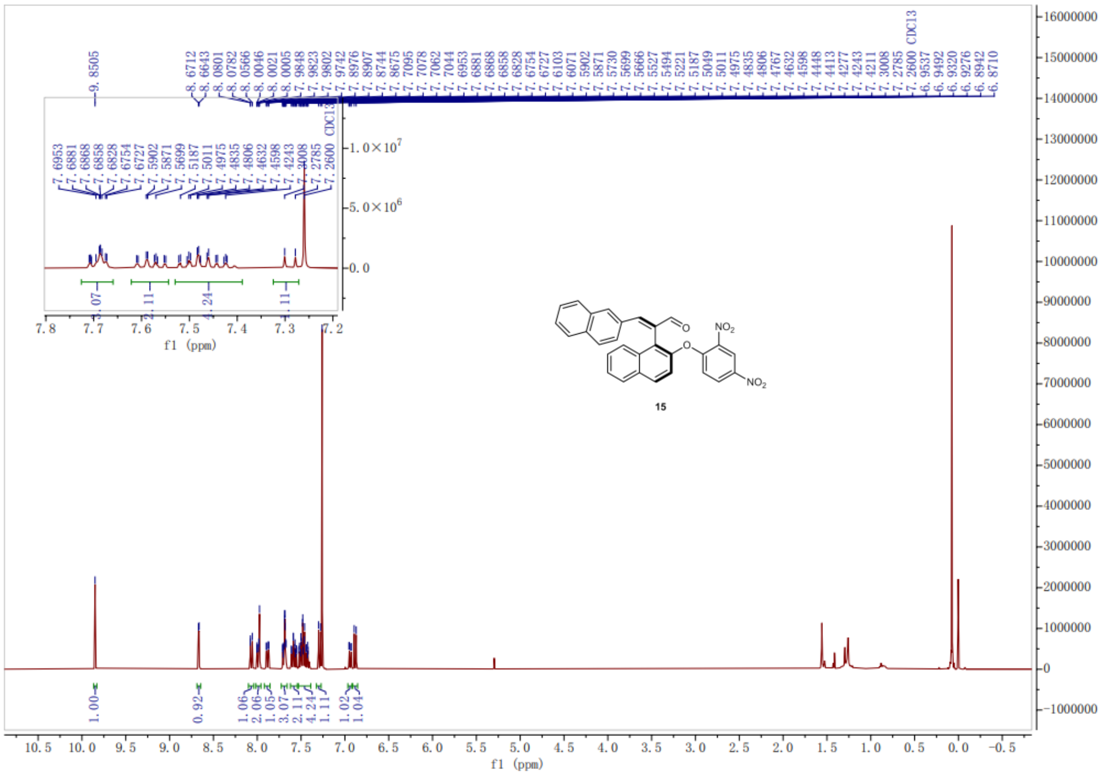

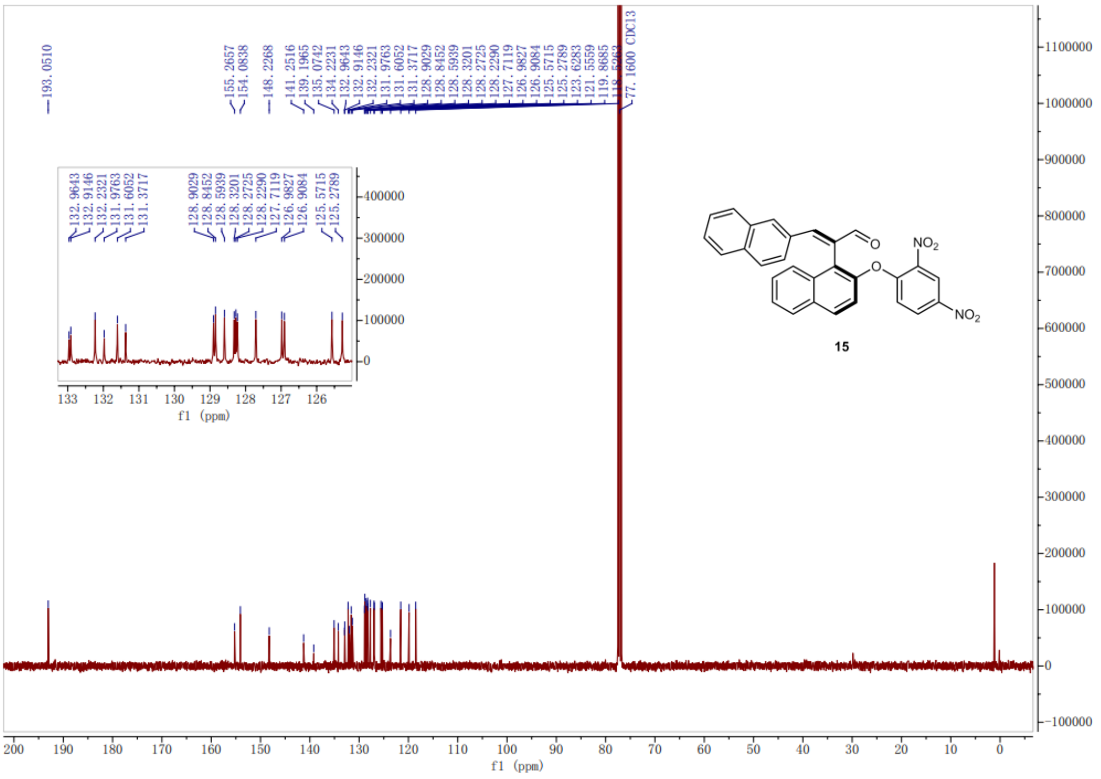

NMR of **16** ($CDCl_3$)

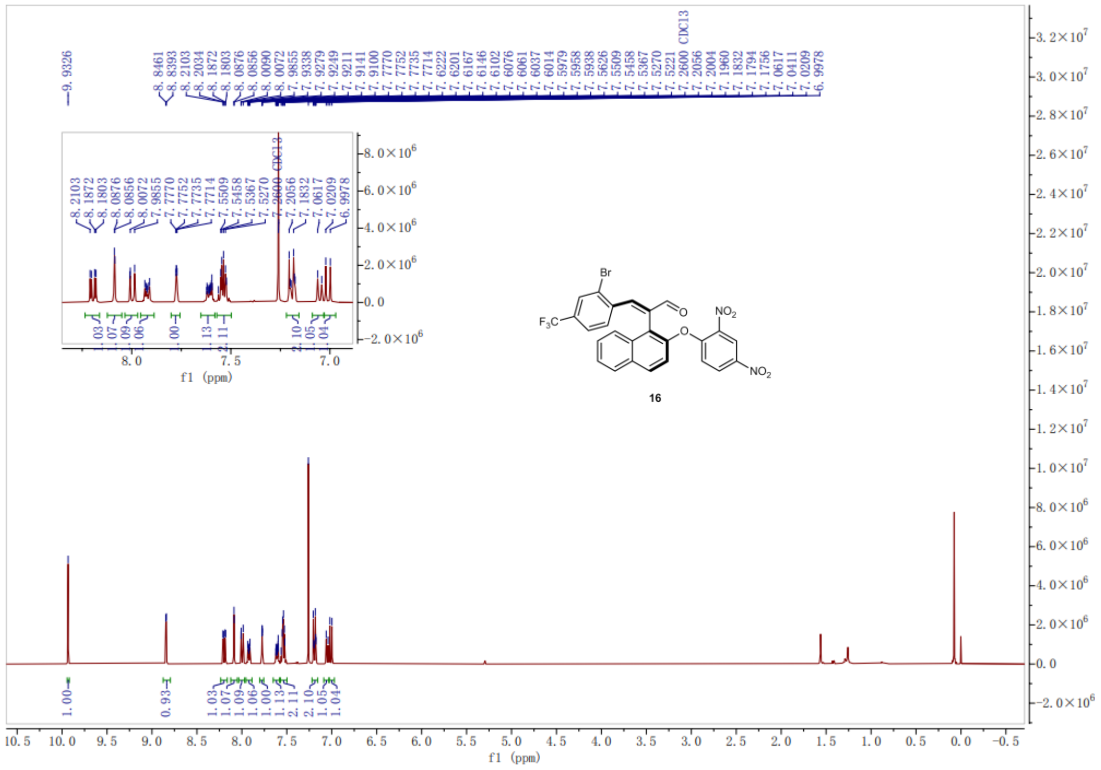

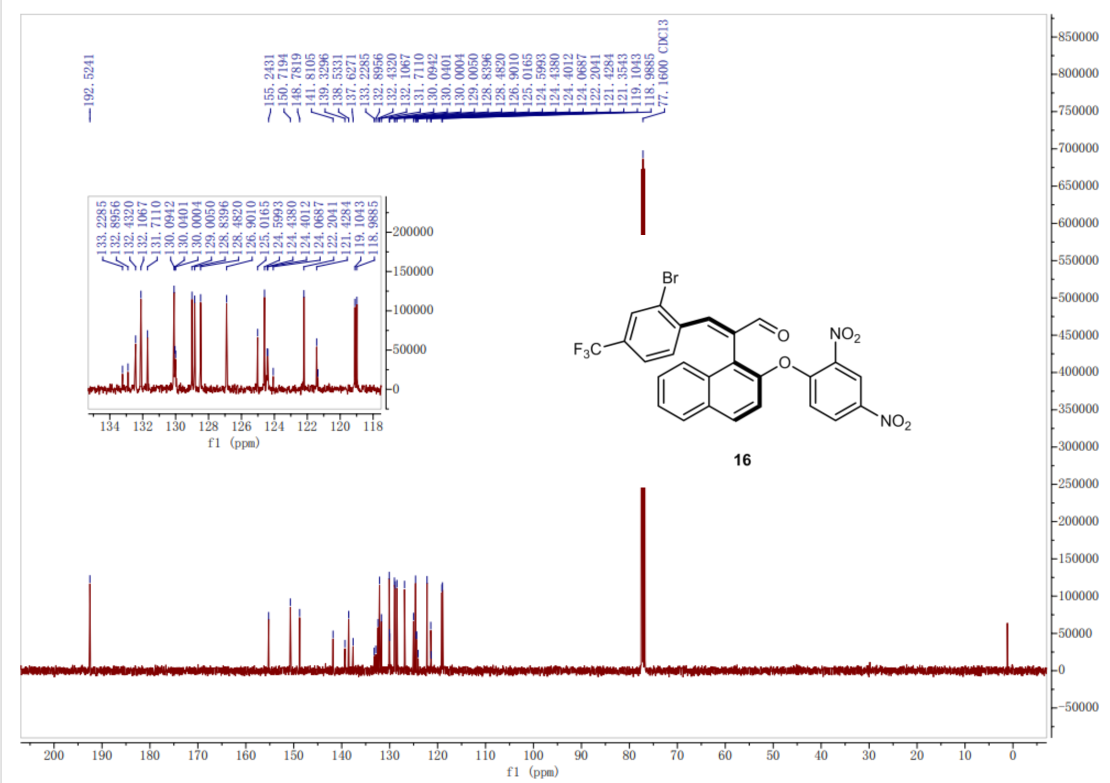
192.5241
155.2431
150.7194
148.7819
141.8105
139.3296
138.5331
137.6271
133.2285
132.8956
132.4320
132.1067
131.7110
130.0942
130.0401
130.0004
129.0050
128.8396
128.4820
126.9010
125.0165
124.5993
124.4380
124.4012
124.0687
122.2041
121.4284
121.3543
119.1043
118.9885
77.1600 CDCl3
Br
F_3C
O
NO_2
O
NO_2
16
f1 (ppm)

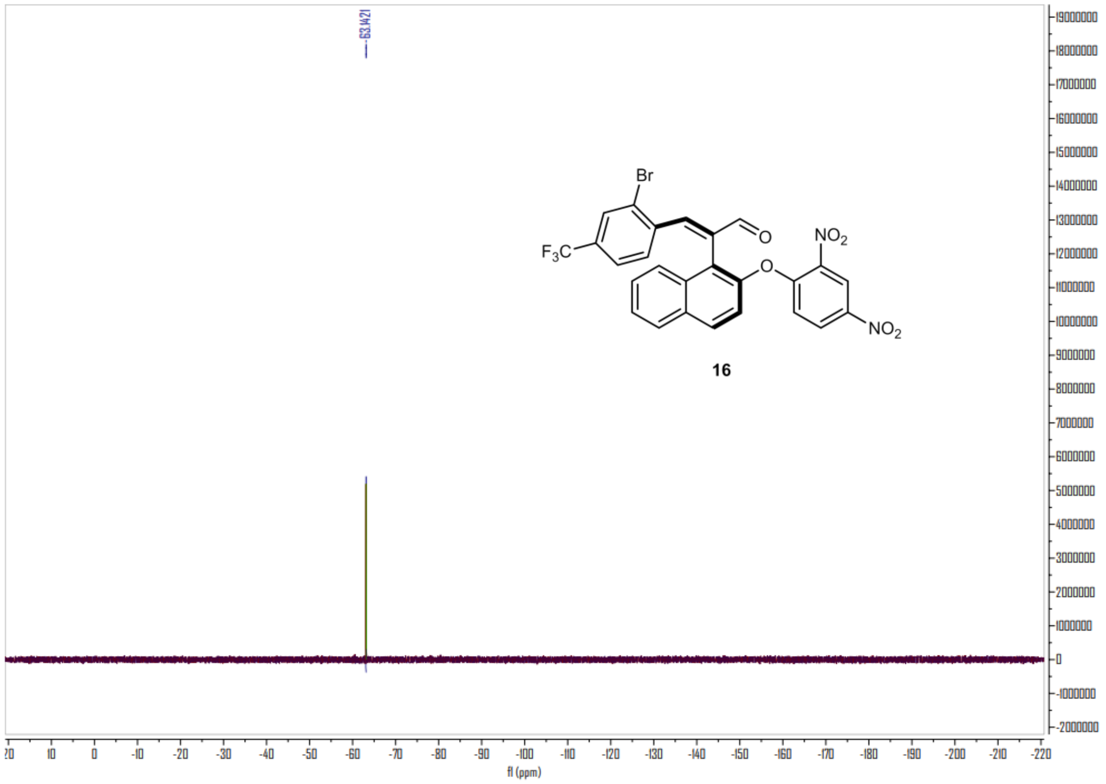
-63.1421
Br
F_3C
O
NO_2
O
NO_2
16
f1 (ppm)

NMR of **17** ($CDCl_3$)

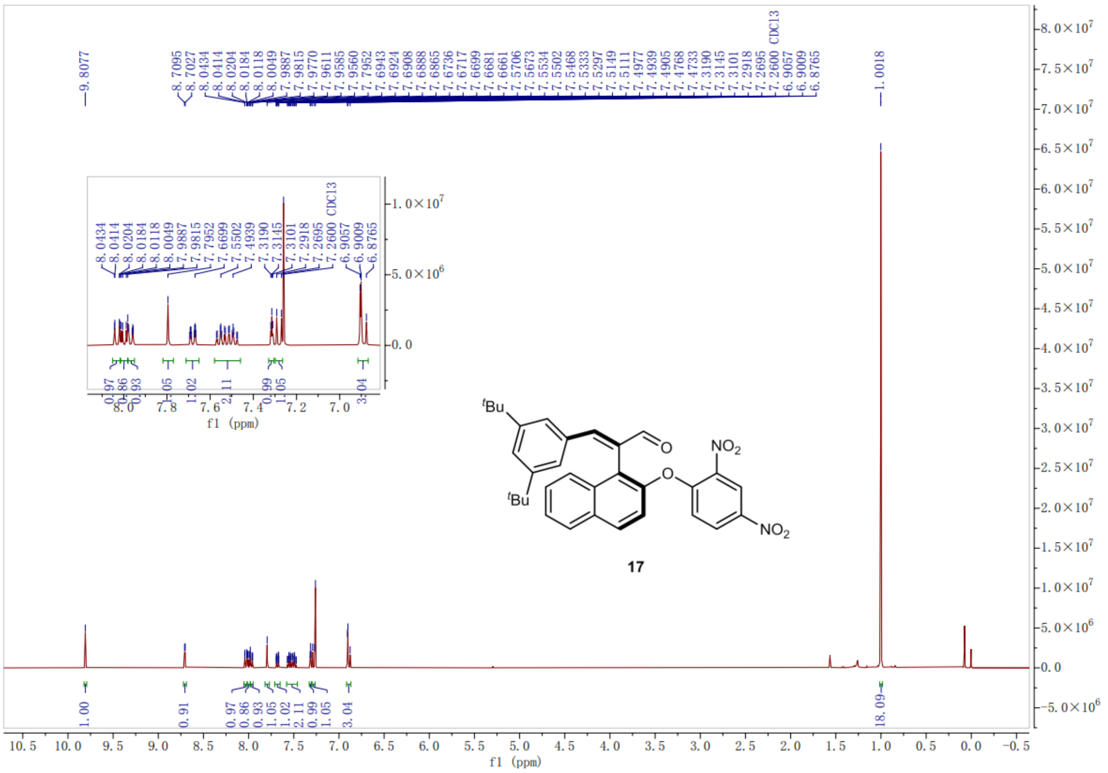

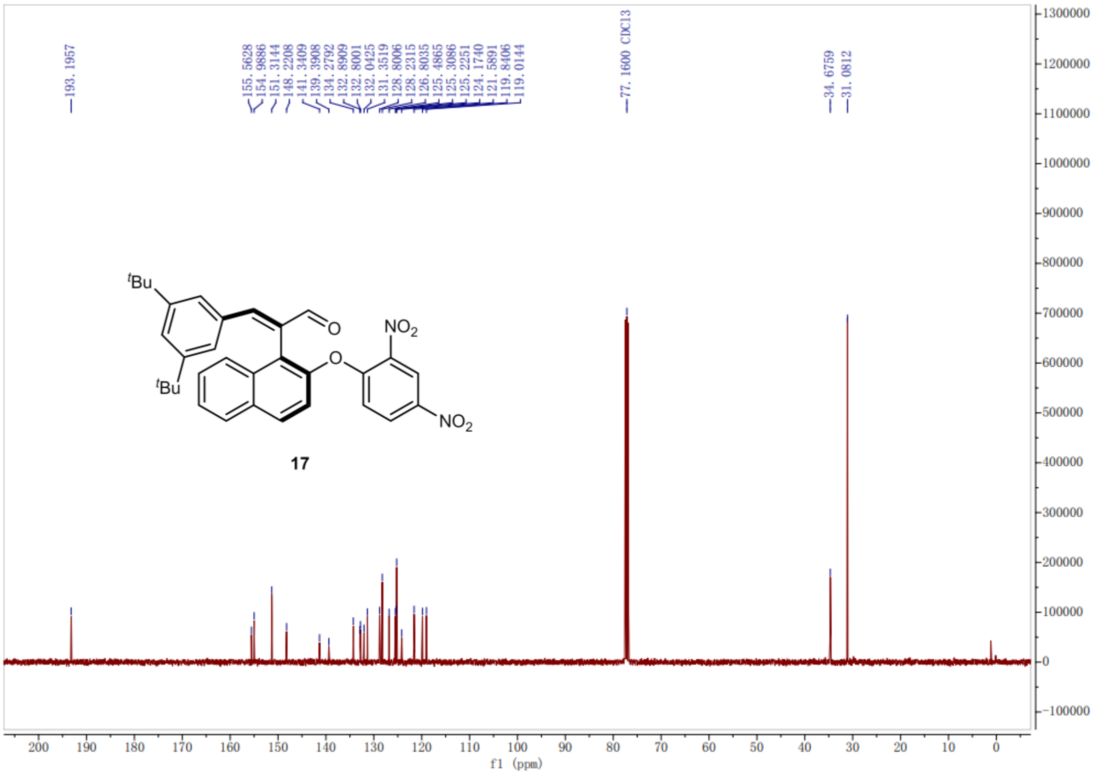

NMR of **18** ($CDCl_3$)

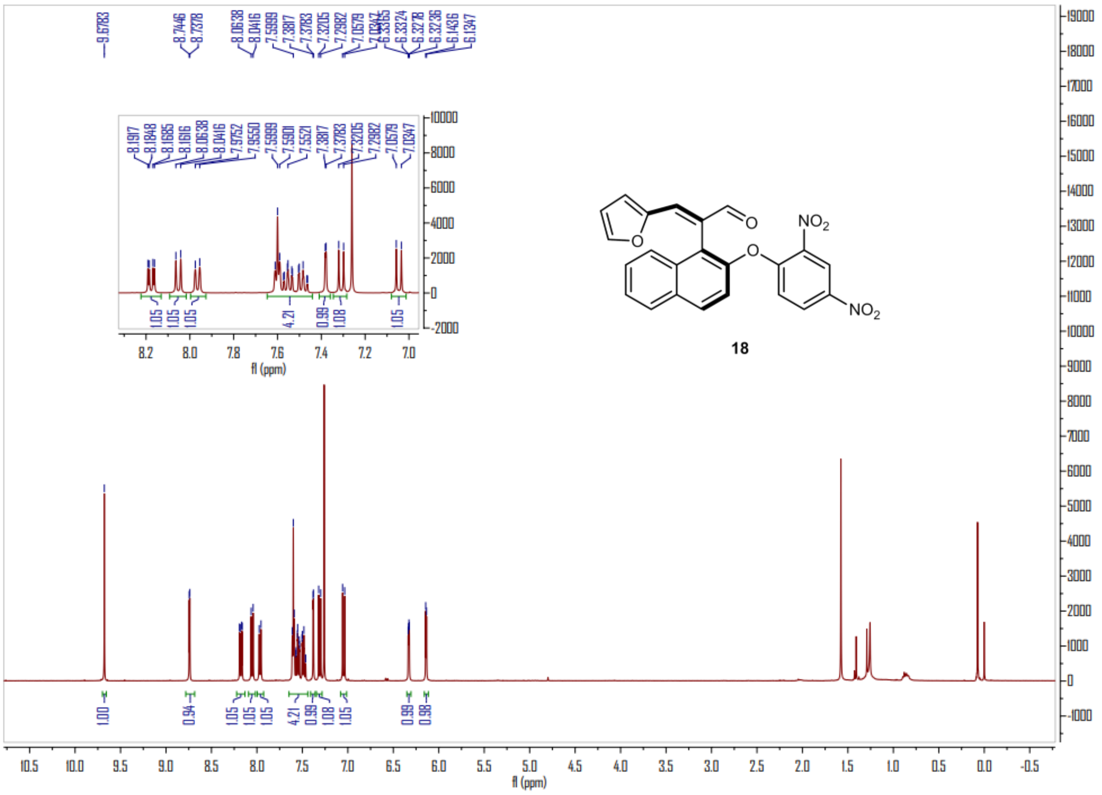

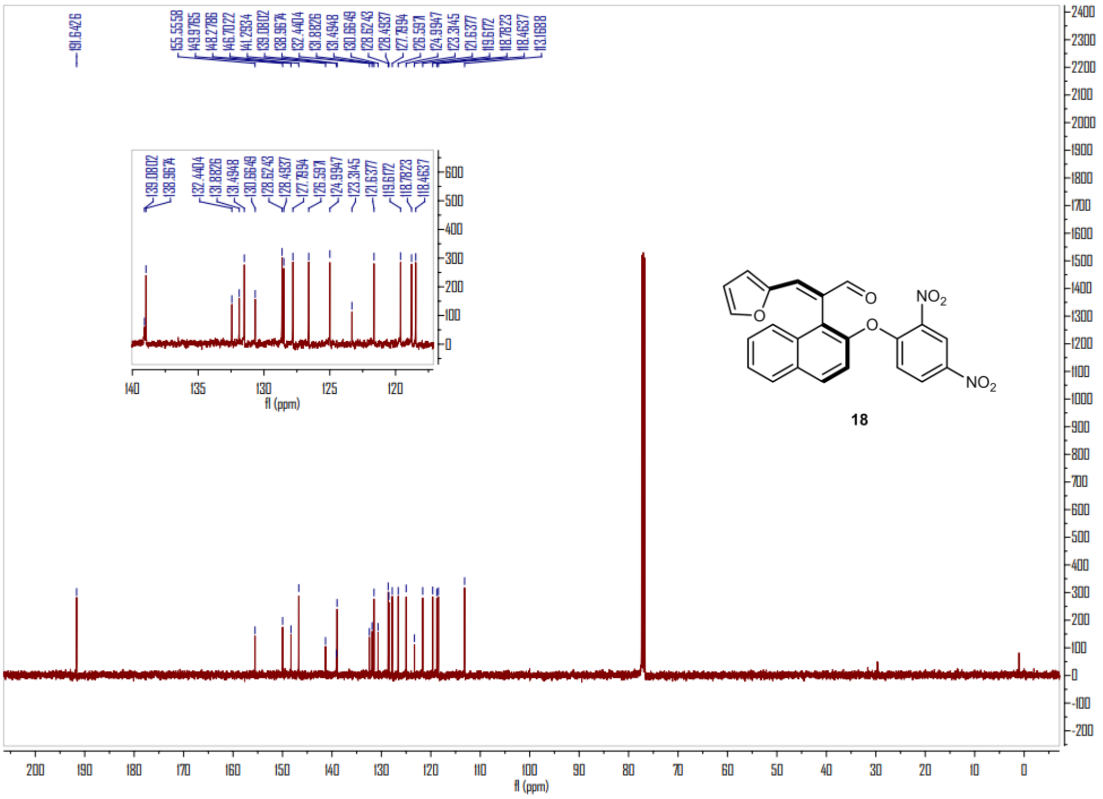

NMR of **19** ($CDCl_3$)

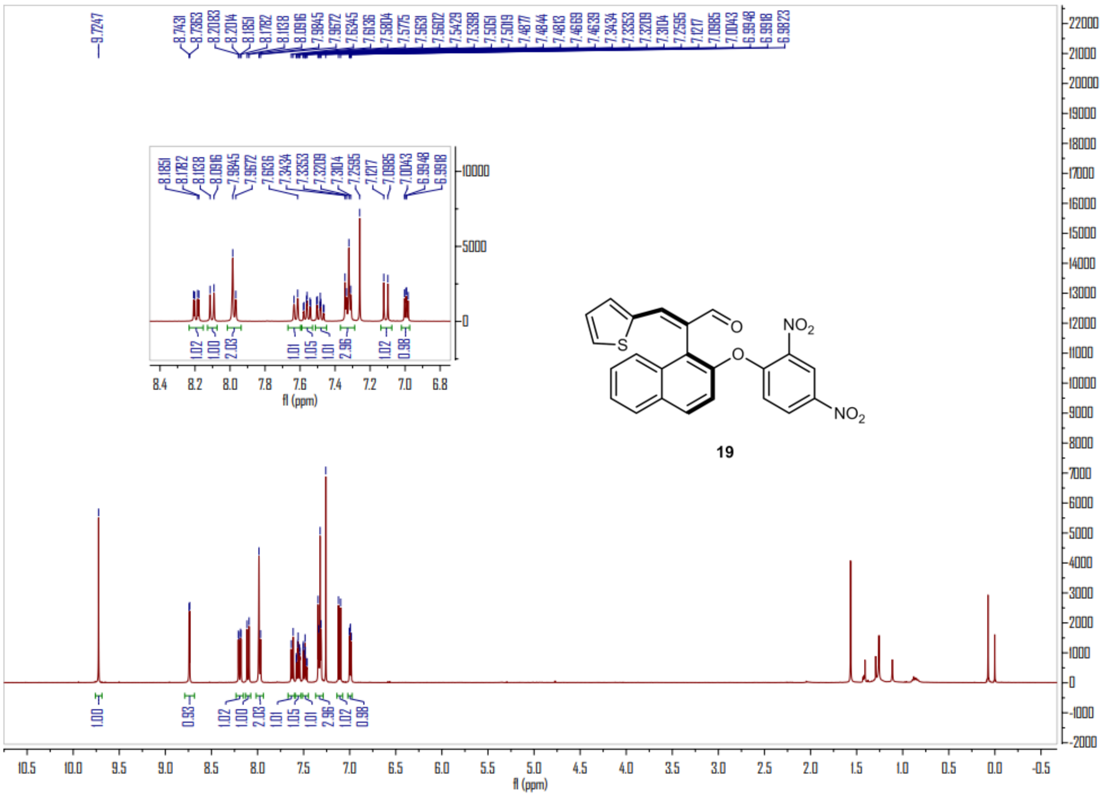

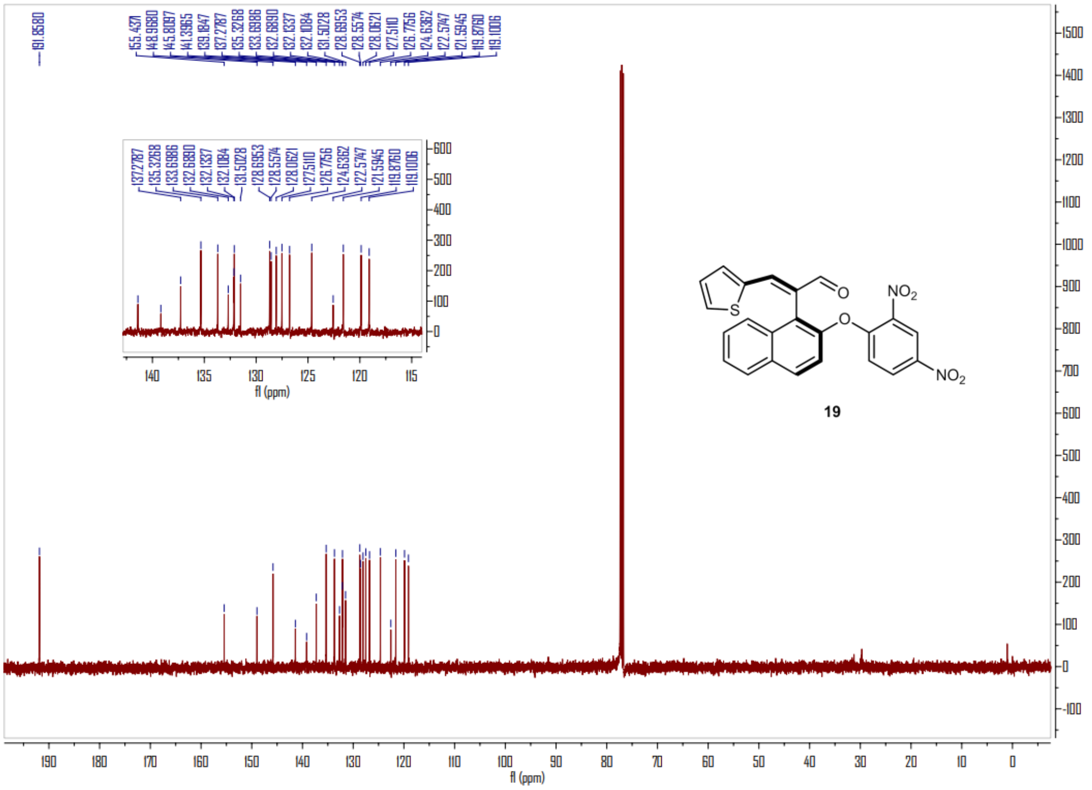

NMR of **20** ($CDCl_3$)

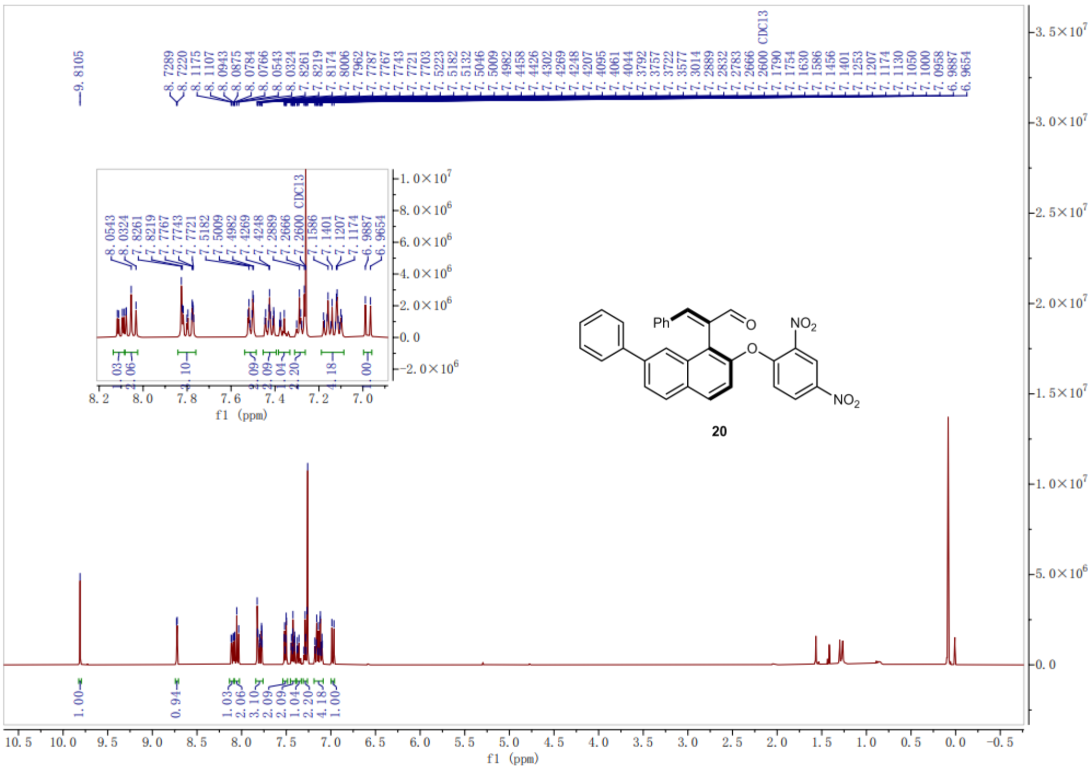

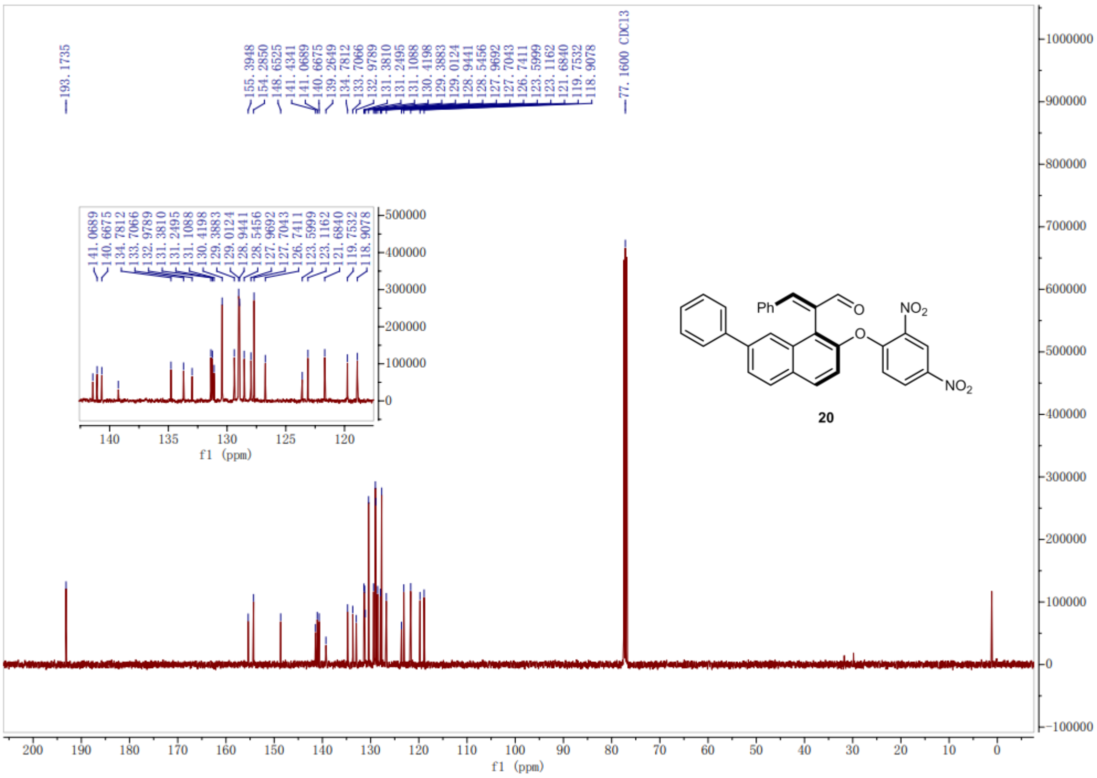

NMR of **21** ($CDCl_3$)

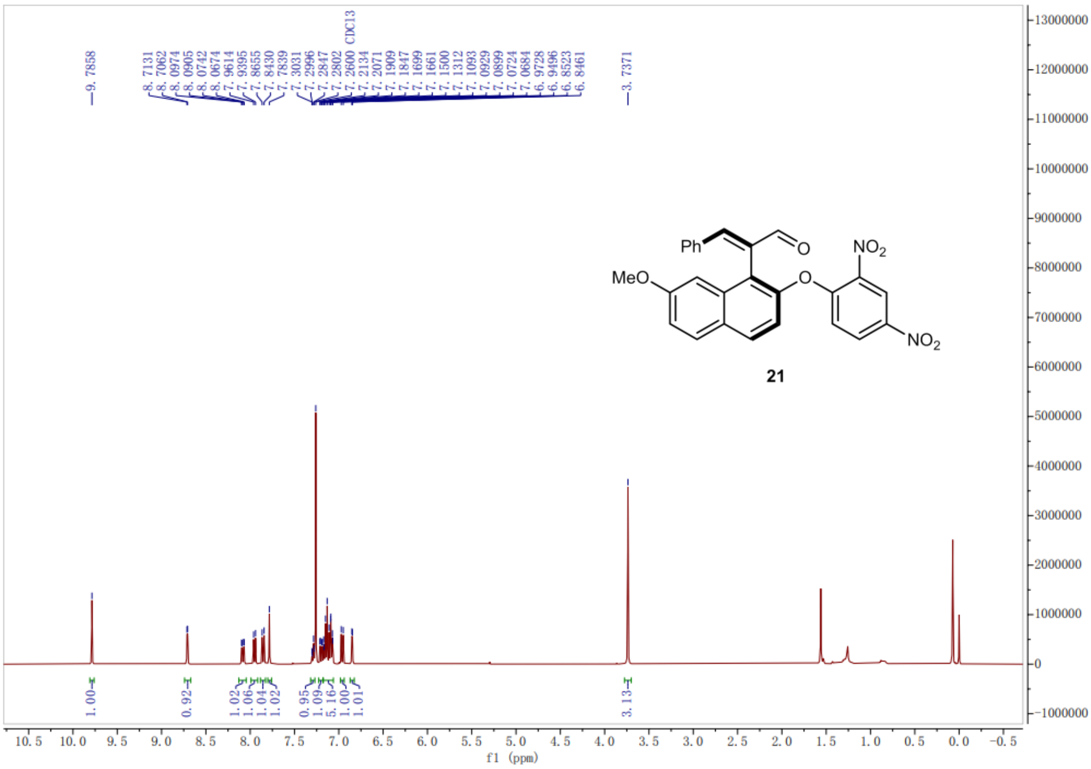

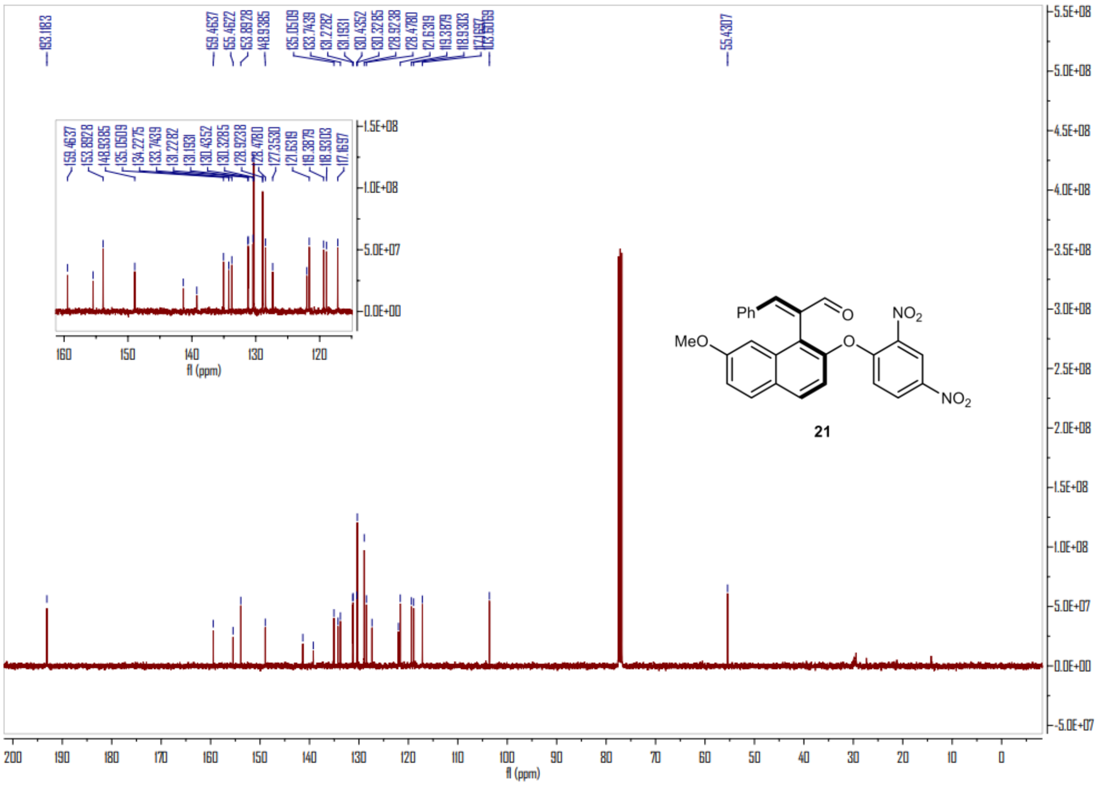

NMR of **22** ($CDCl_3$)

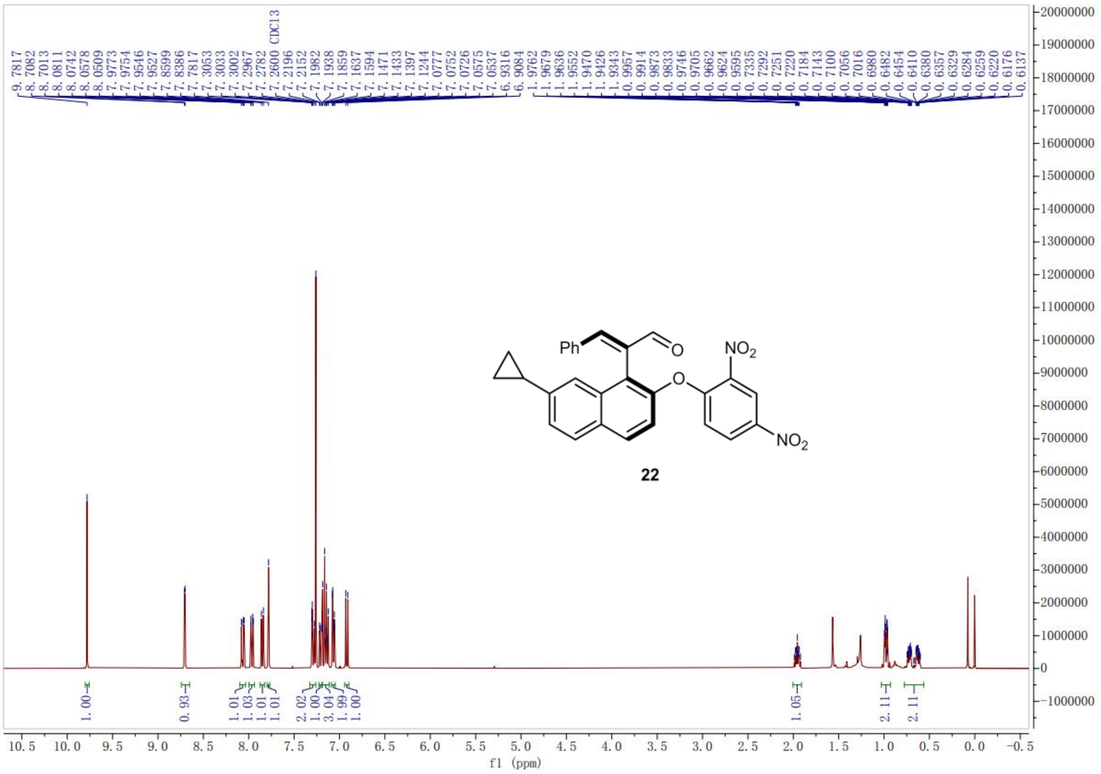

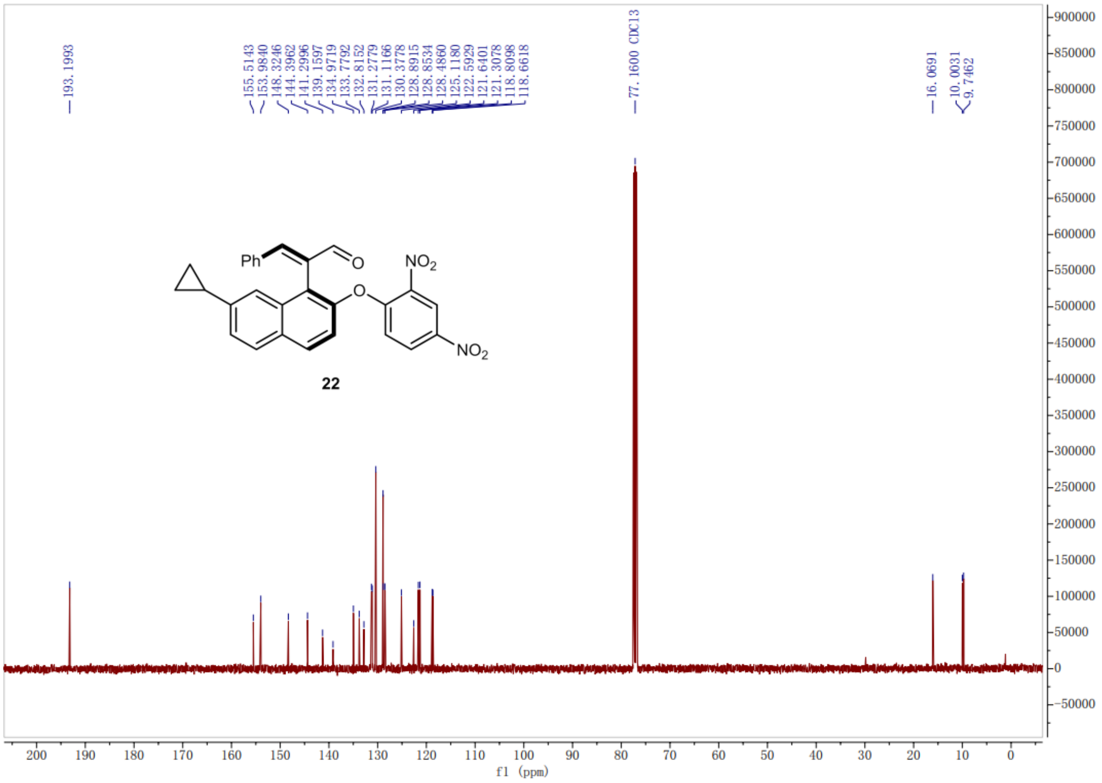

NMR of **23** ($CDCl_3$)

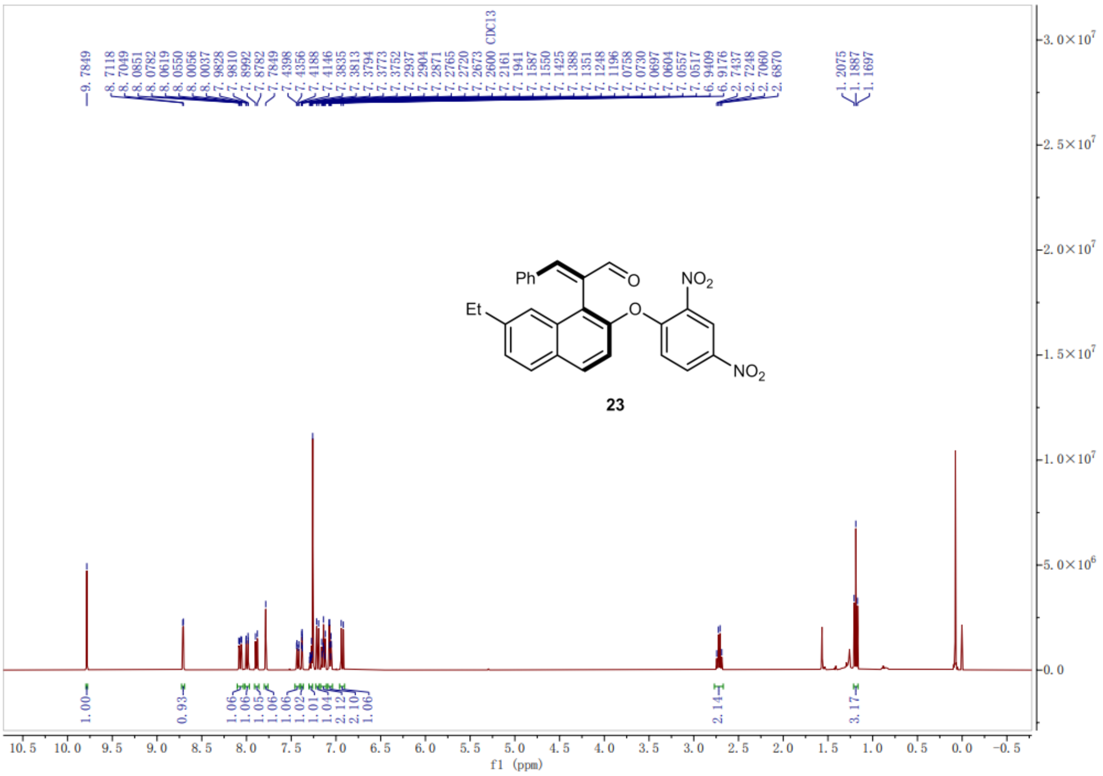

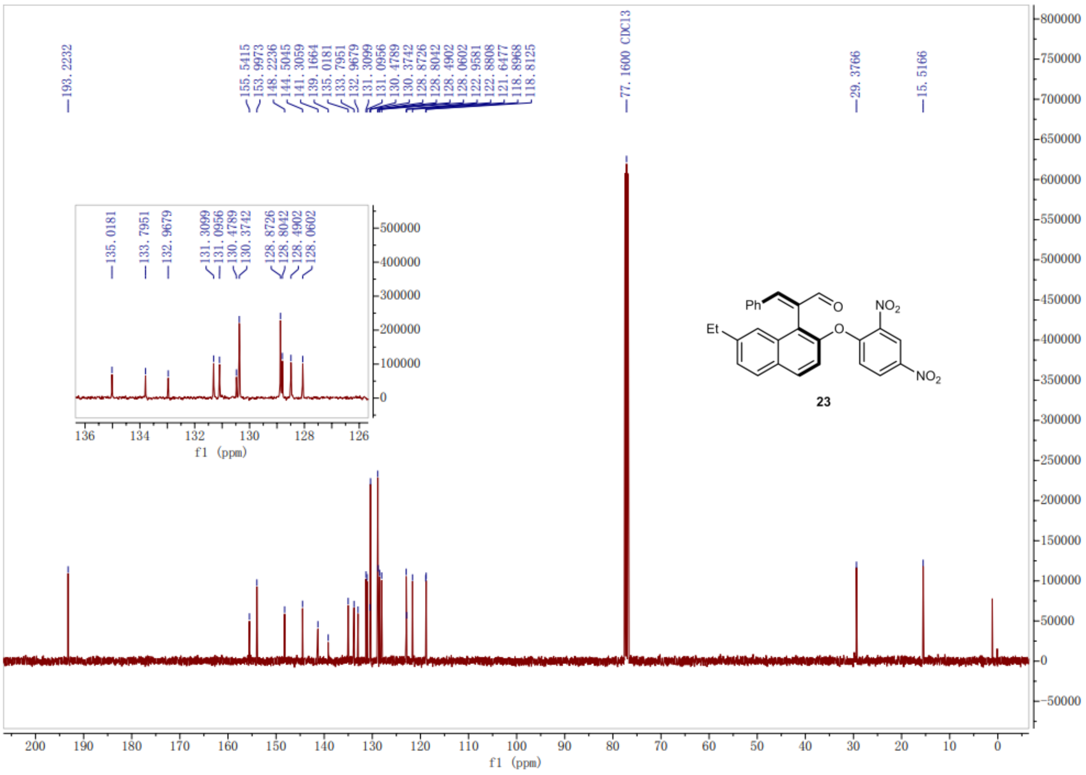

NMR of **24** ($CDCl_3$)

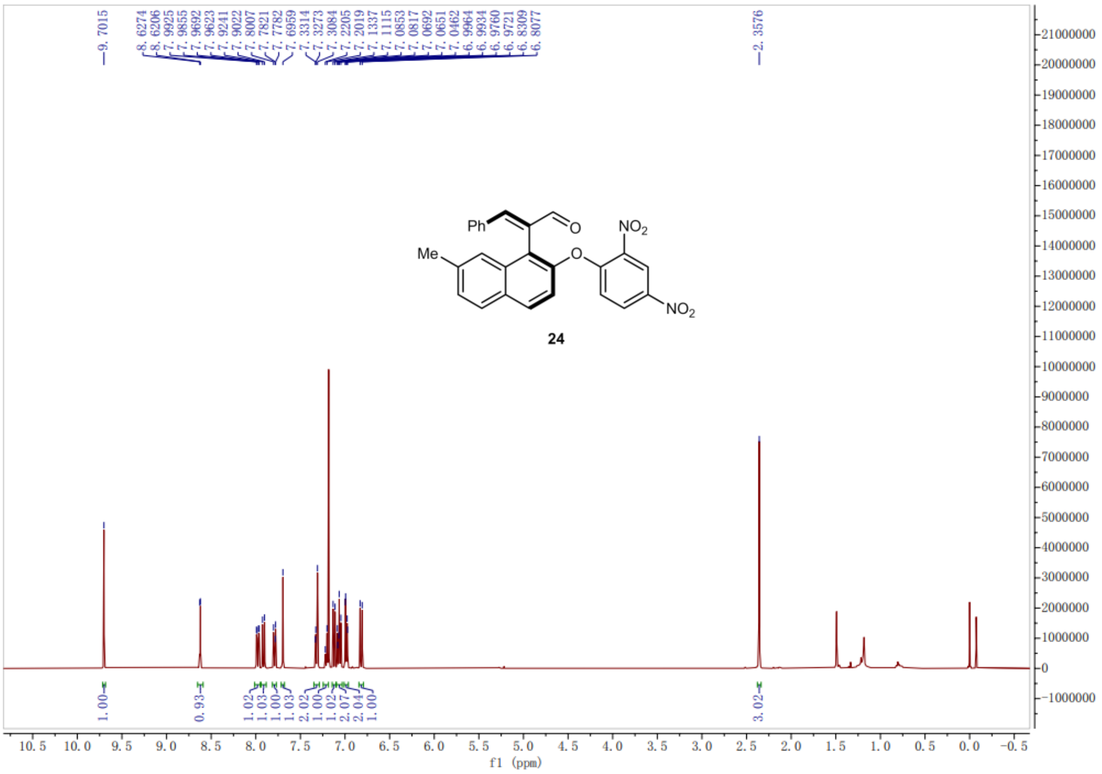

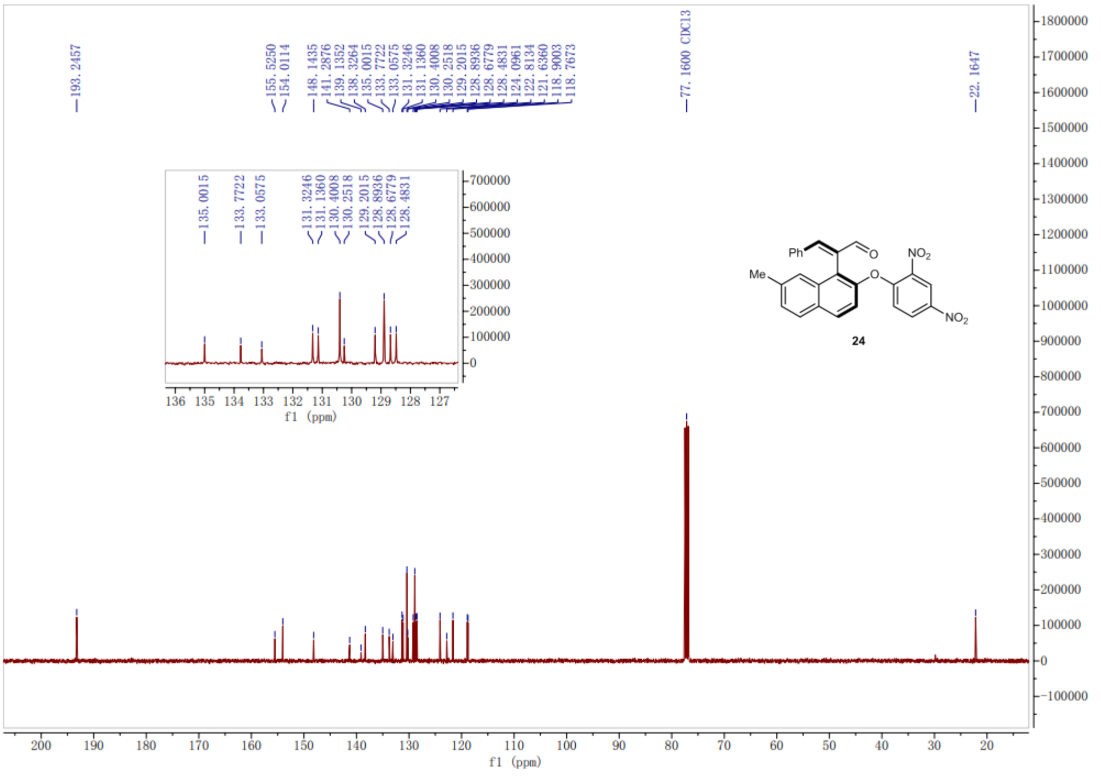

NMR of **25** ($CDCl_3$)

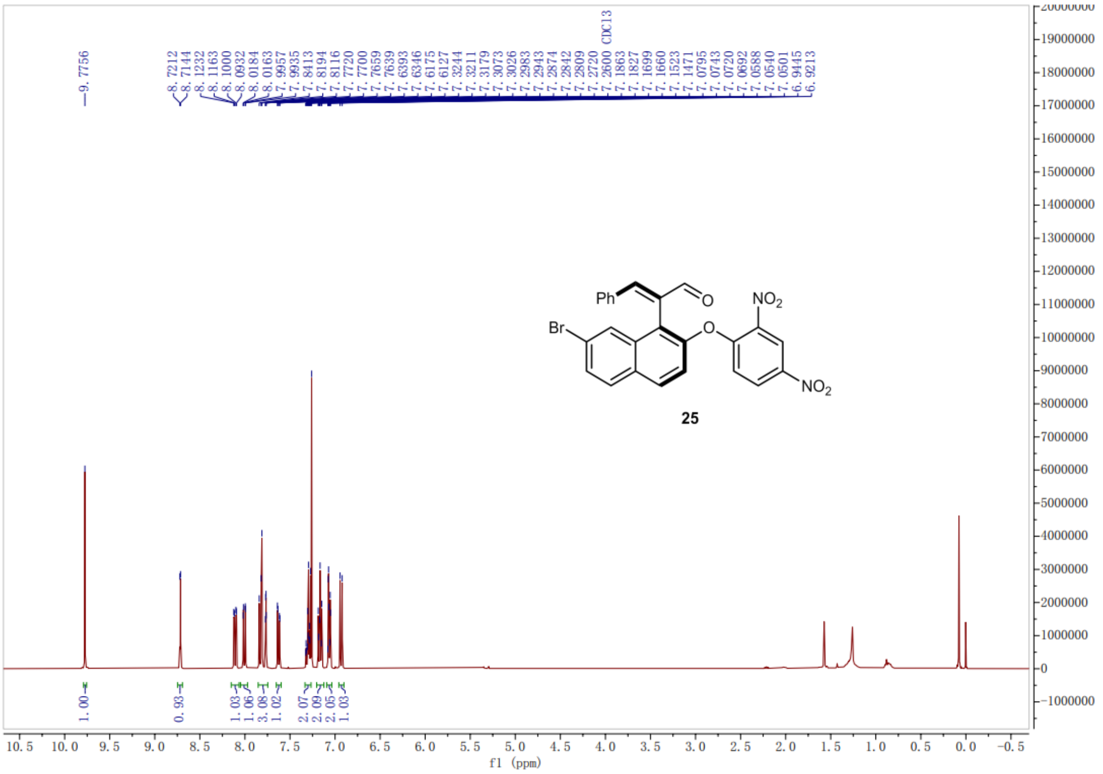

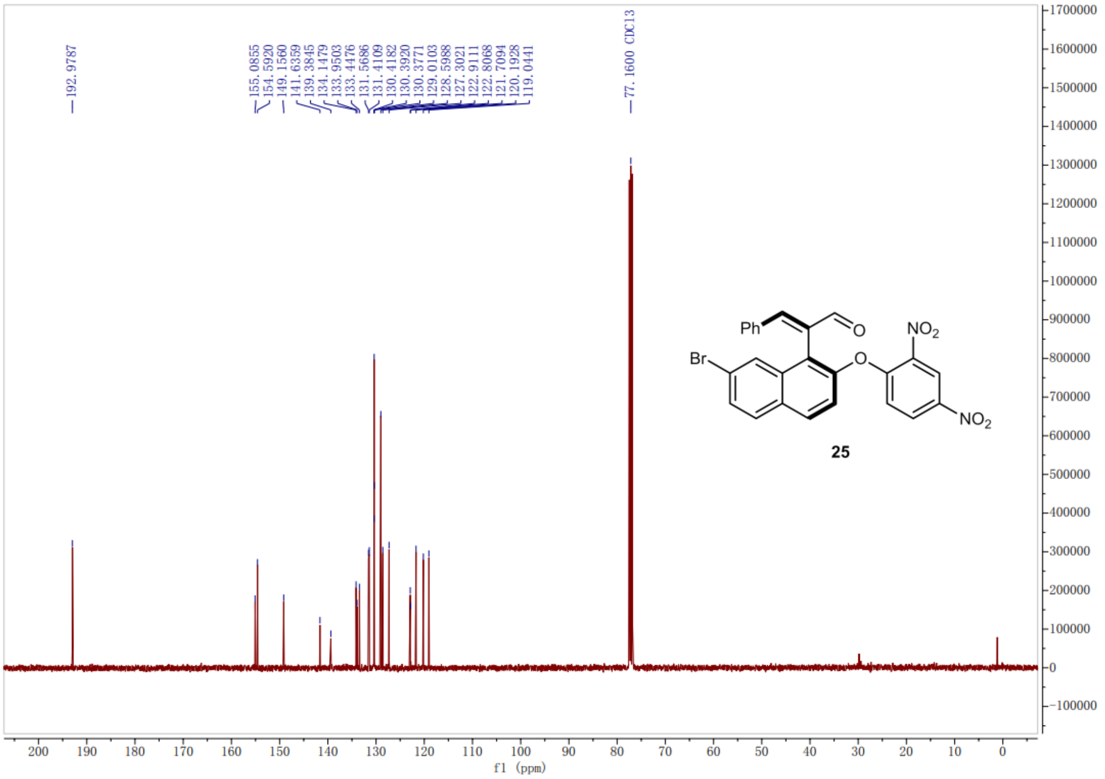

NMR of **26** ($CDCl_3$)

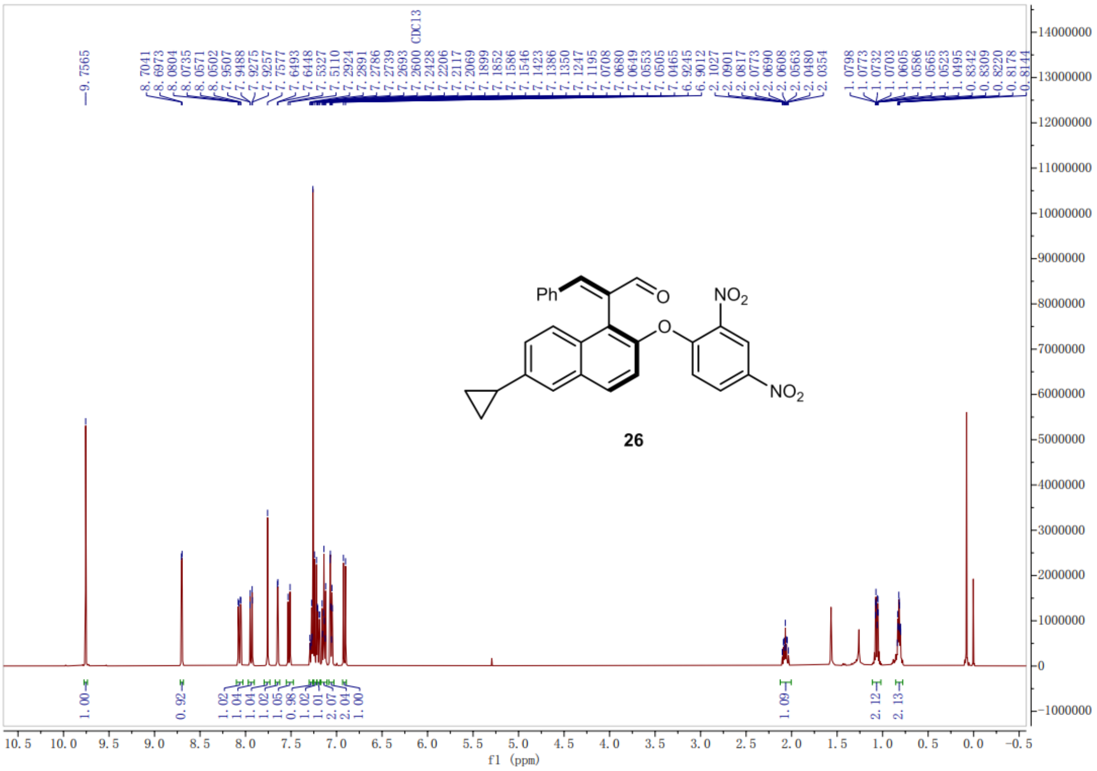

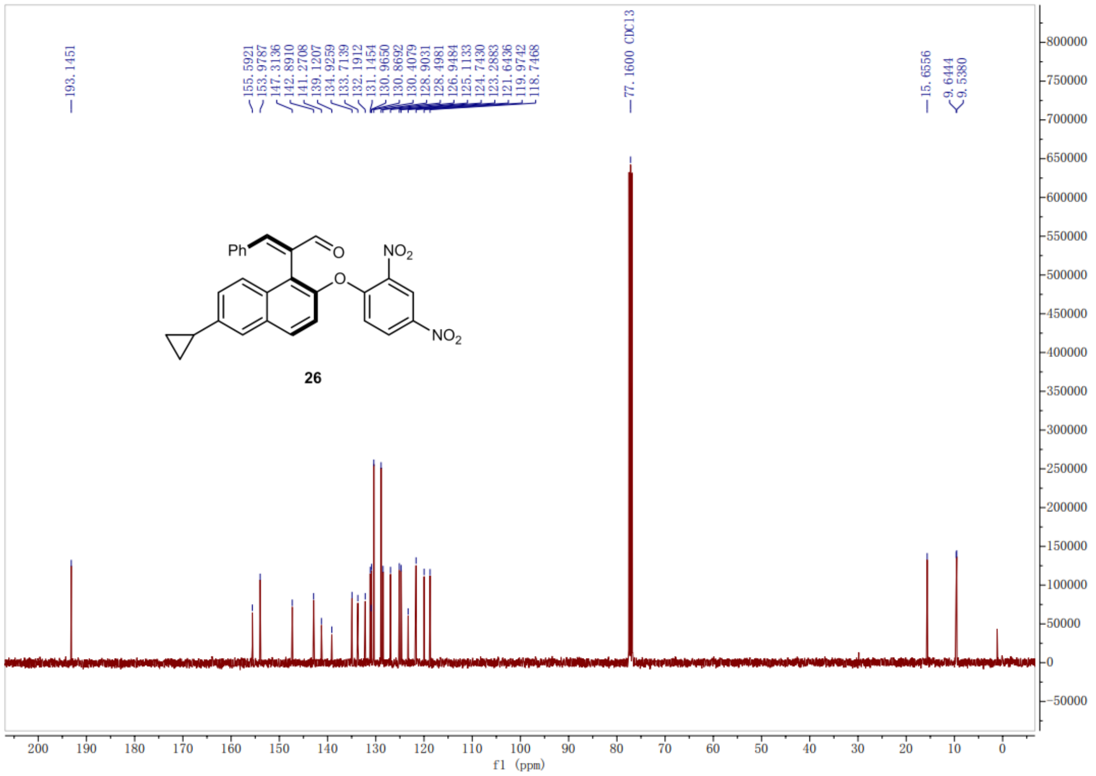

NMR of **27** ($CDCl_3$)

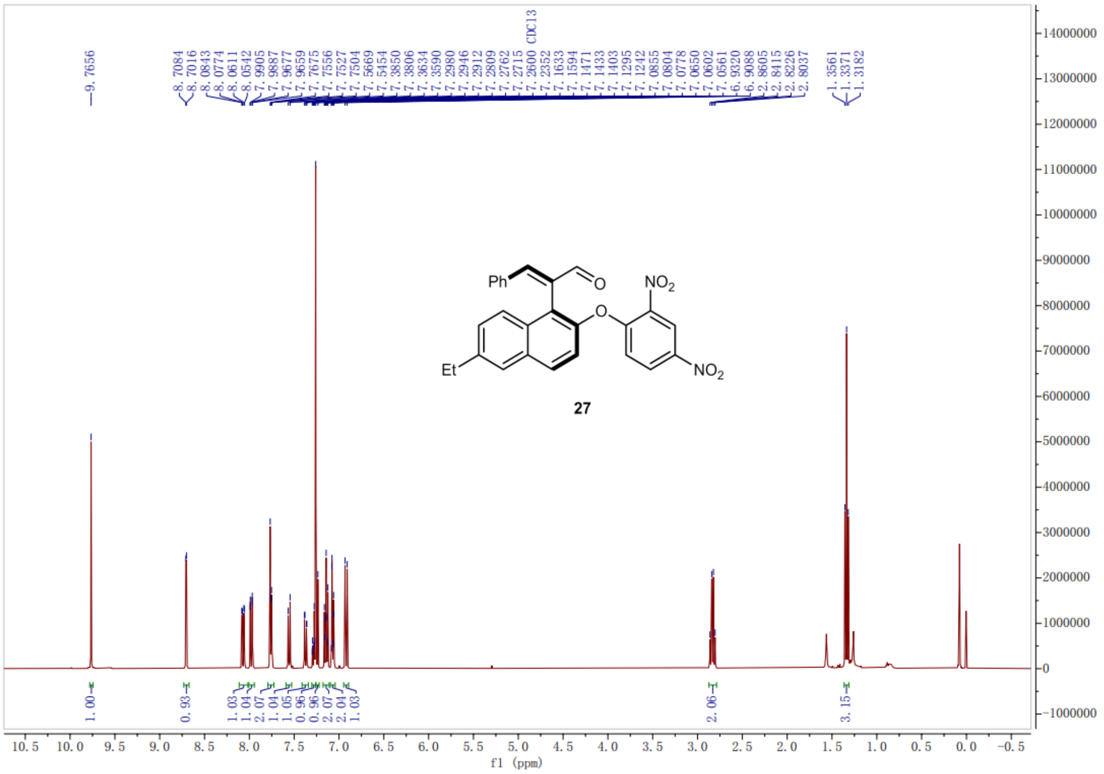

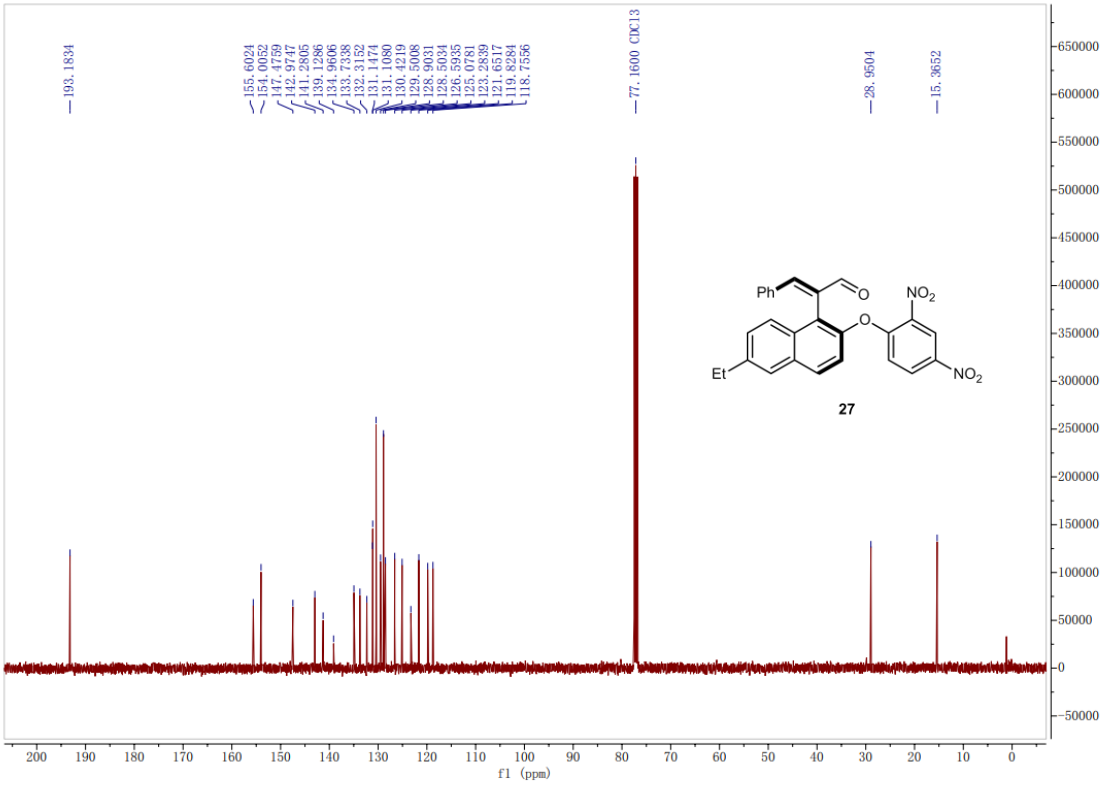

NMR of **28** (DMSO-d_6)

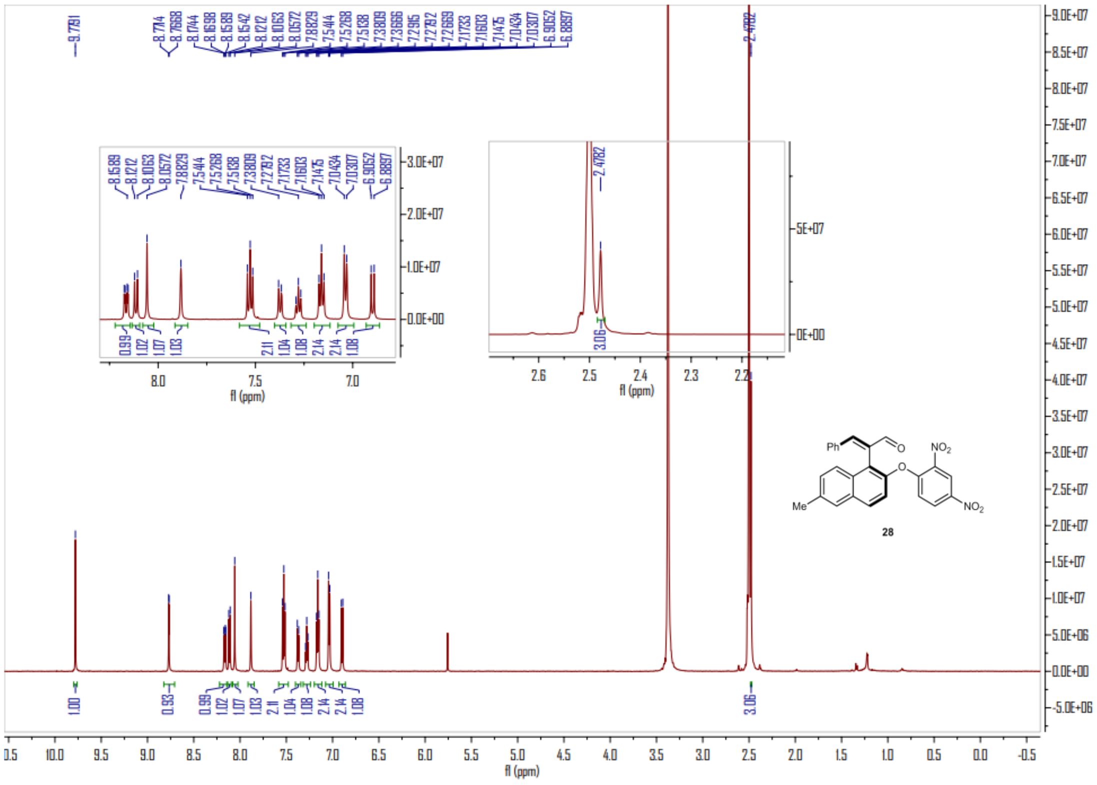

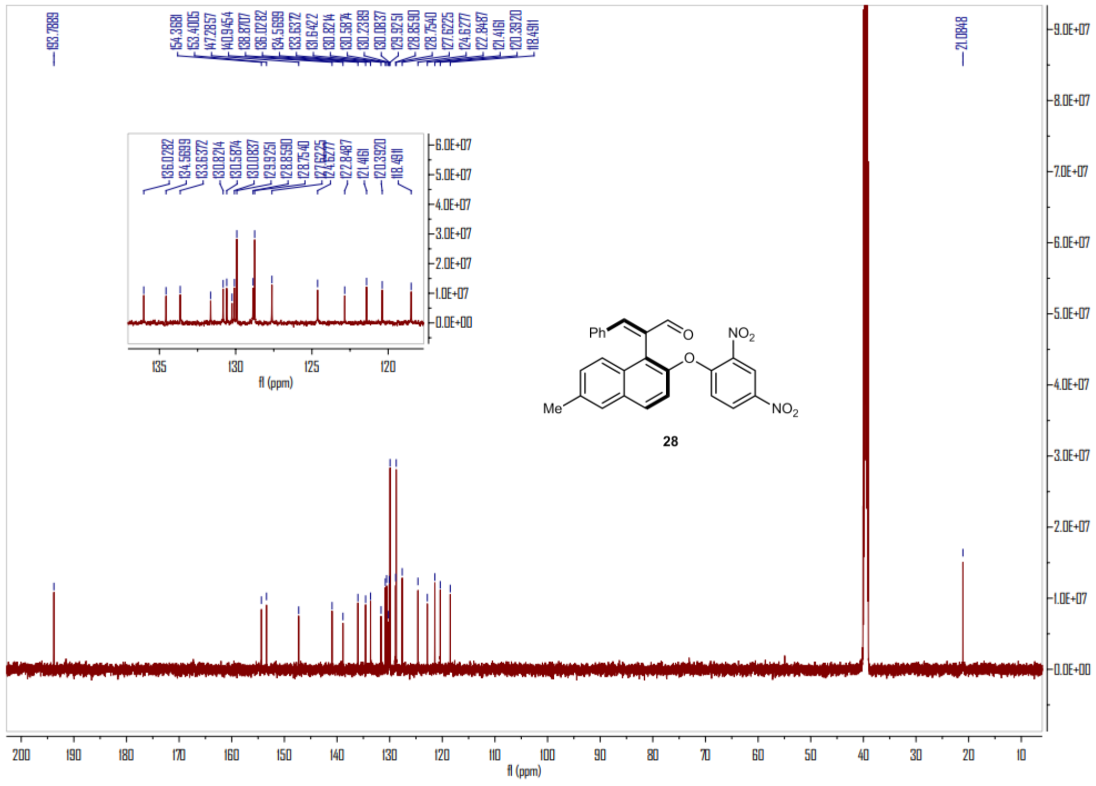

NMR of **29** (DMSO-d_6)

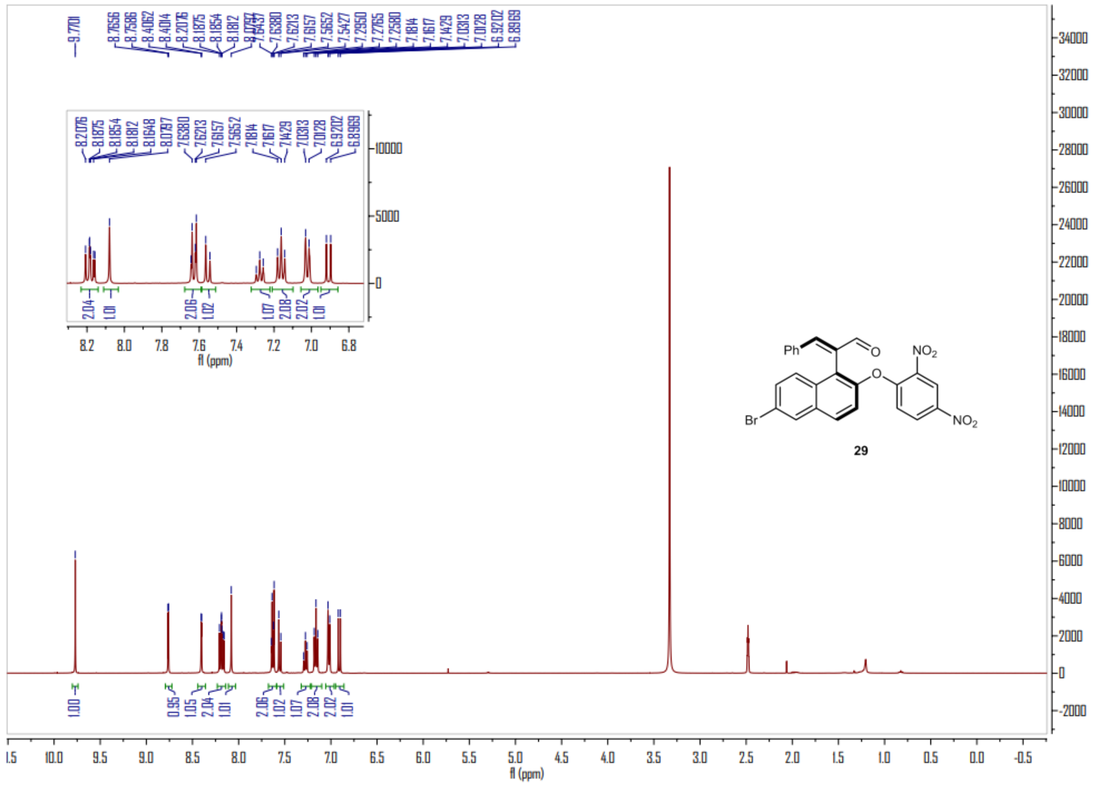

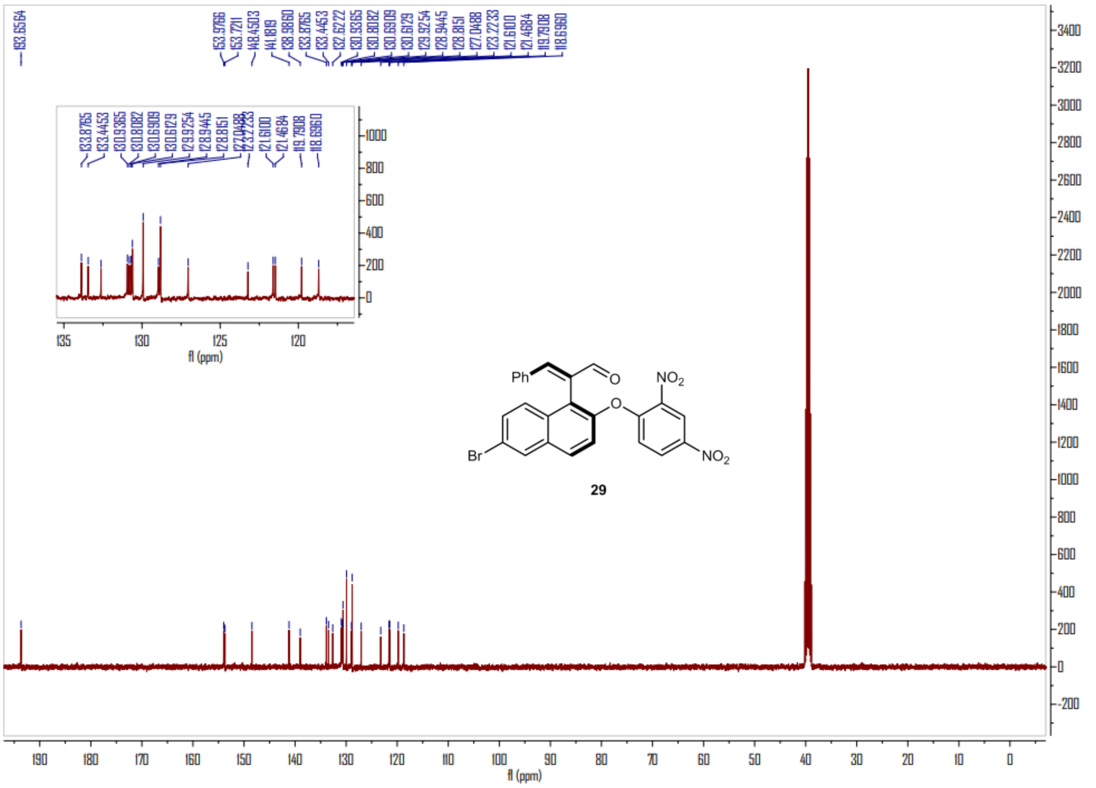

NMR of **30** ($CDCl_3$)

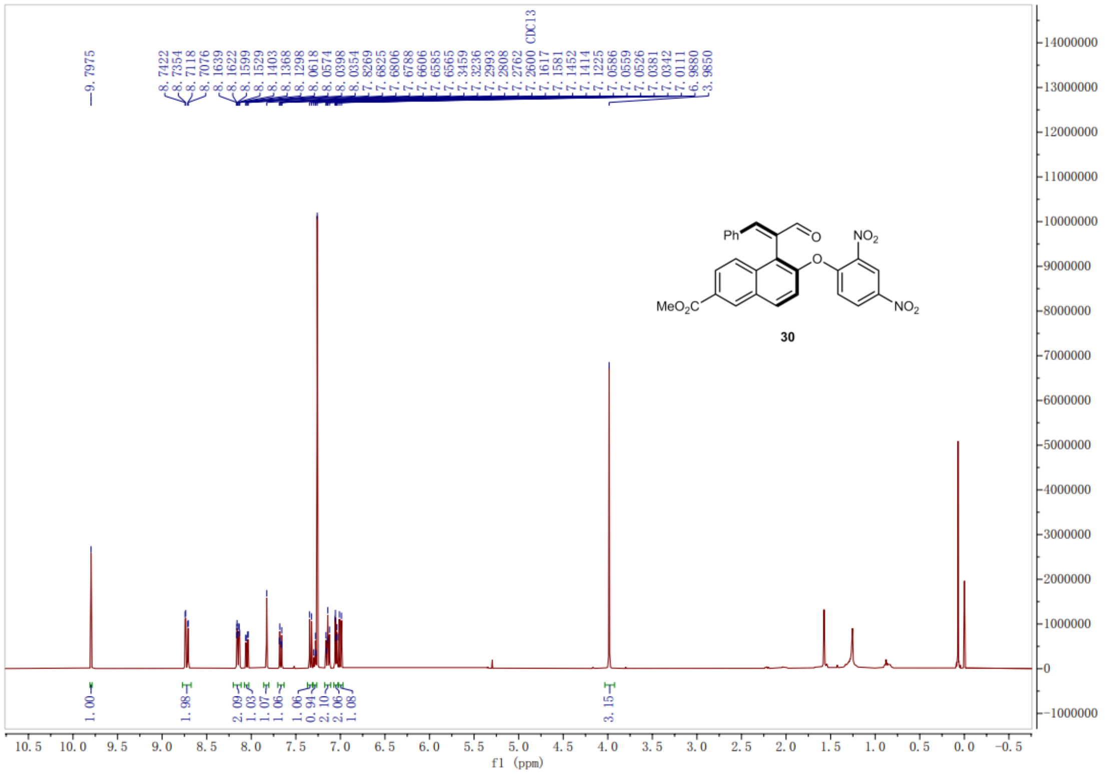

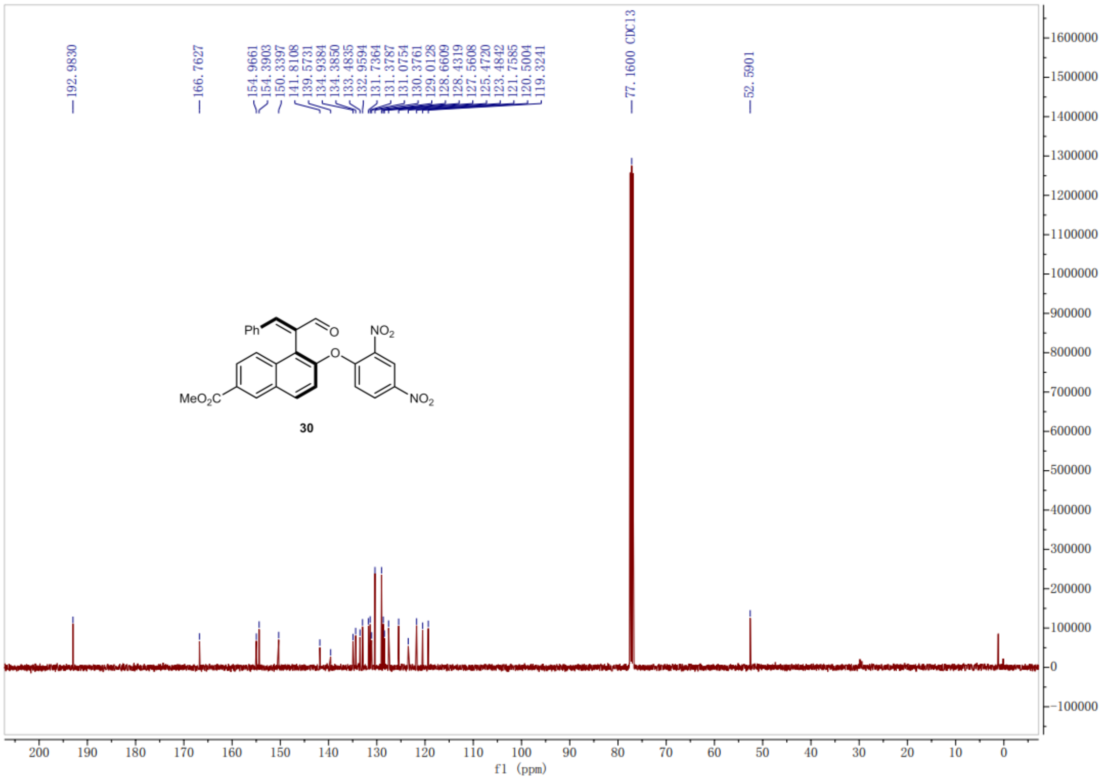

NMR of **31** ($CDCl_3$)

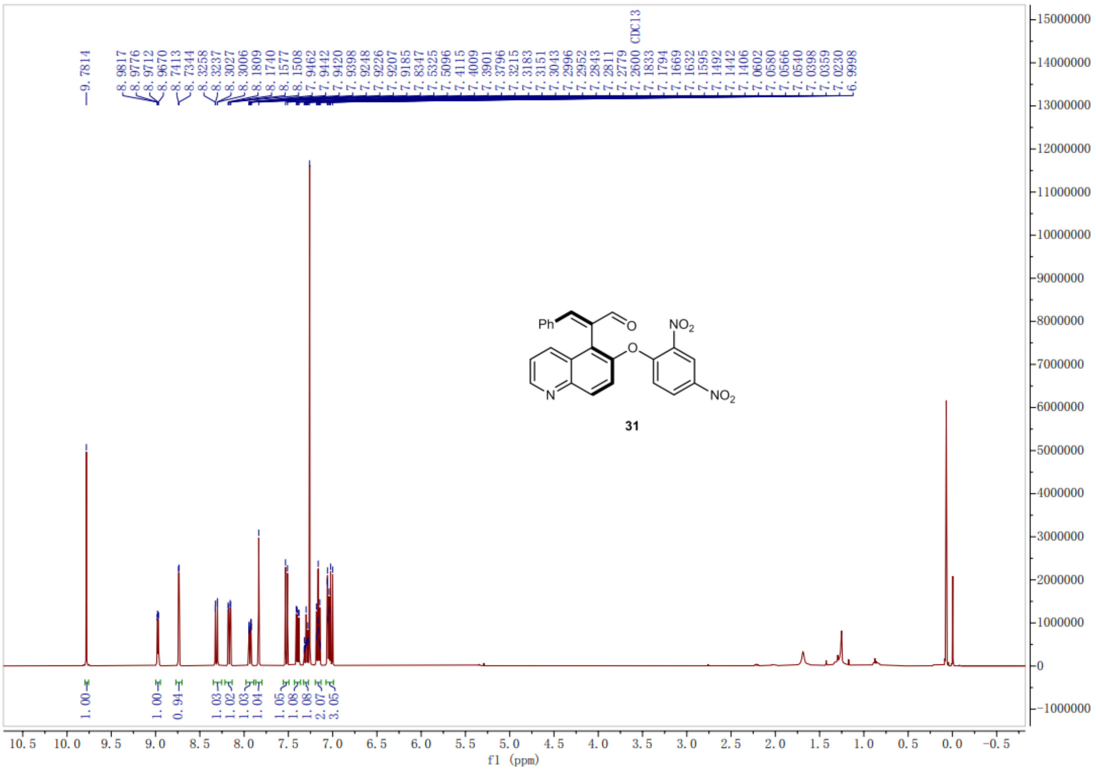

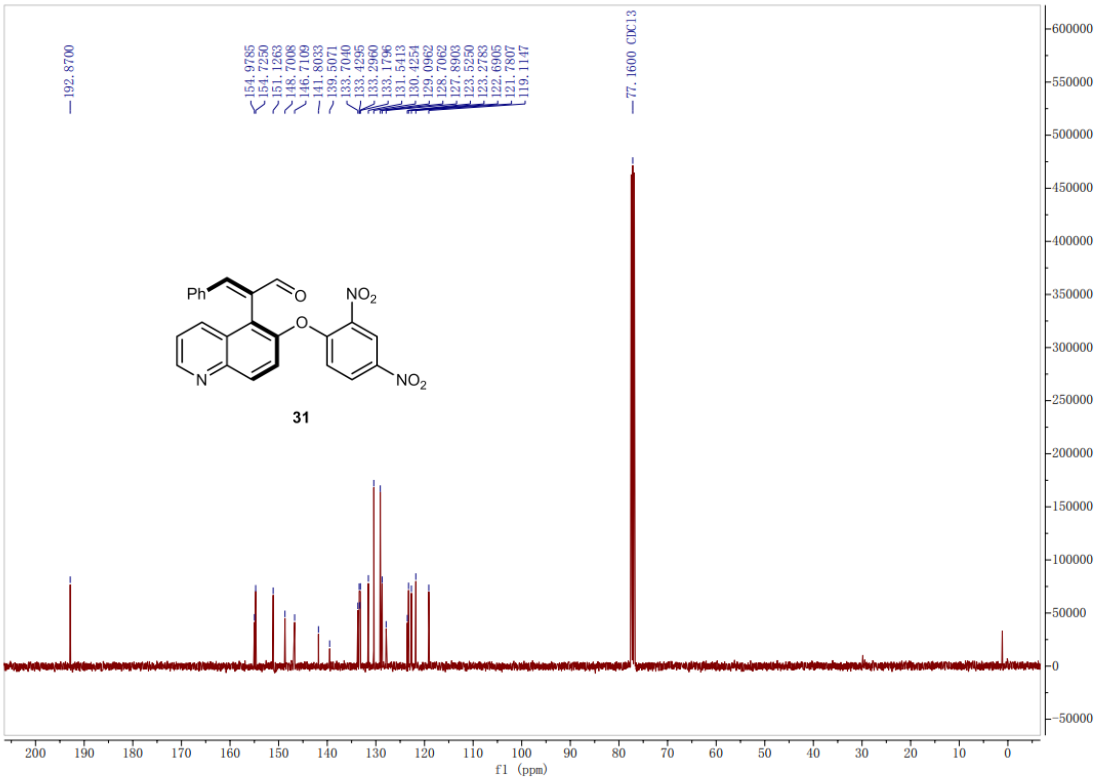

NMR of **32** ($CDCl_3$)

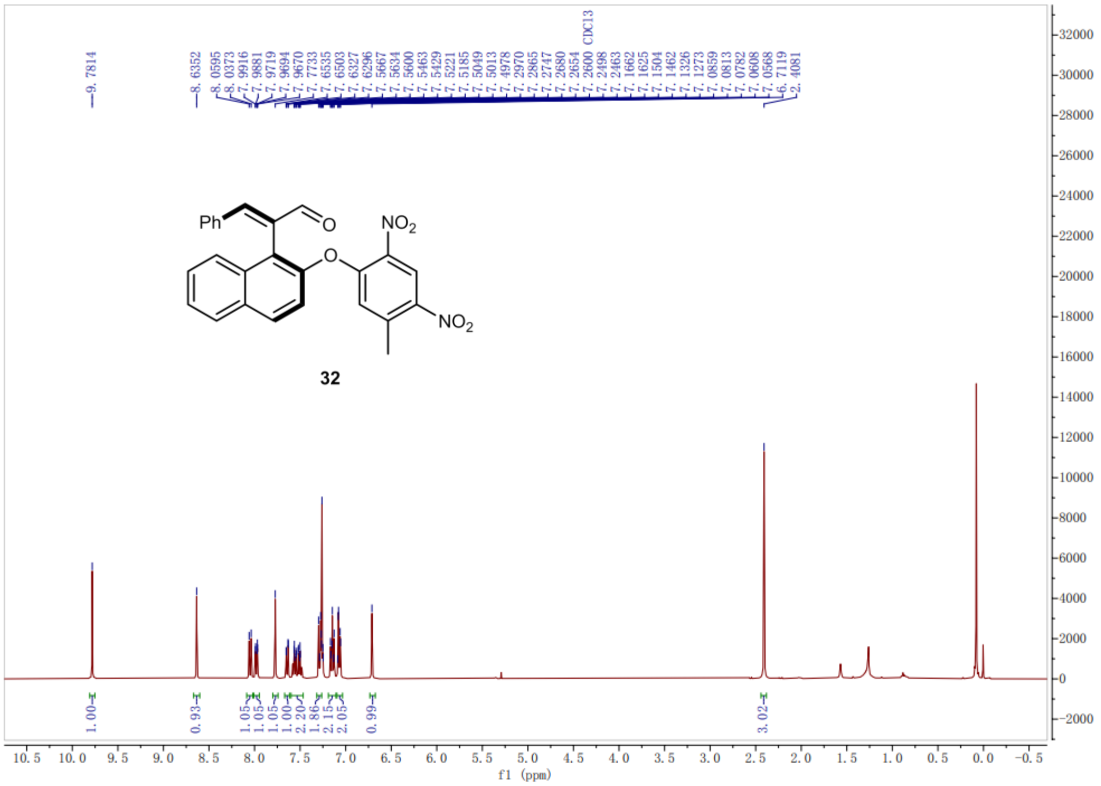

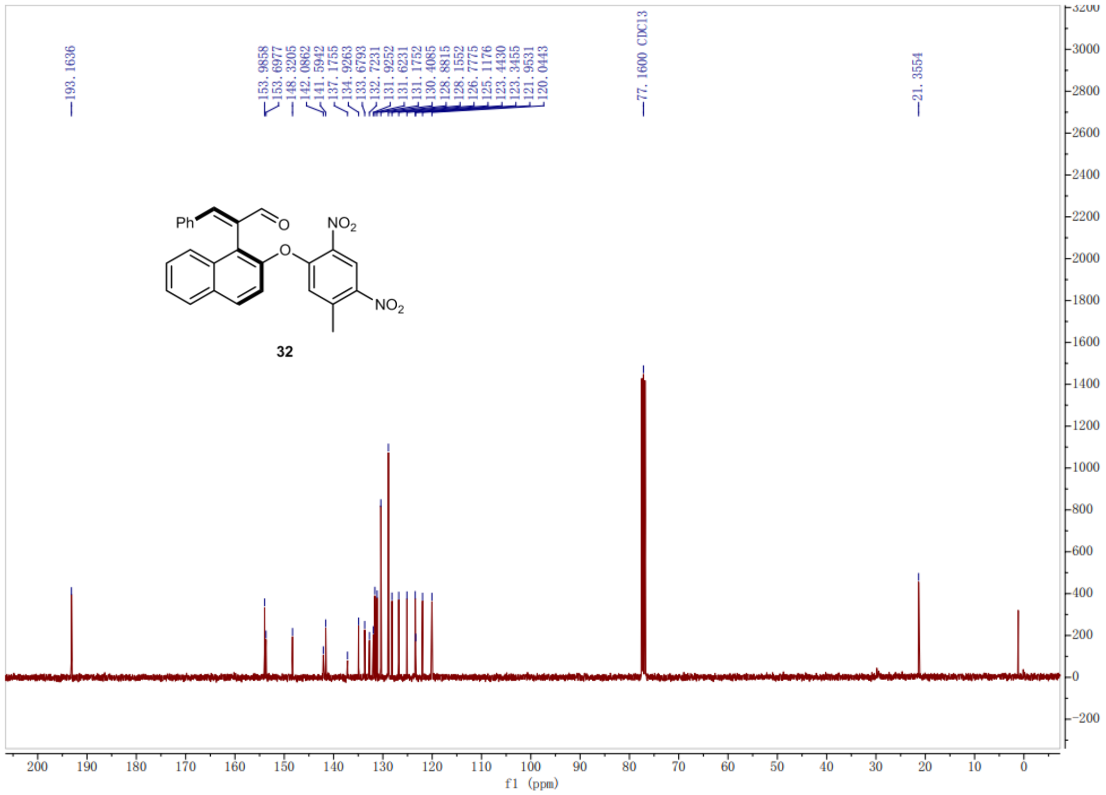

NMR of **33** ($CDCl_3$)

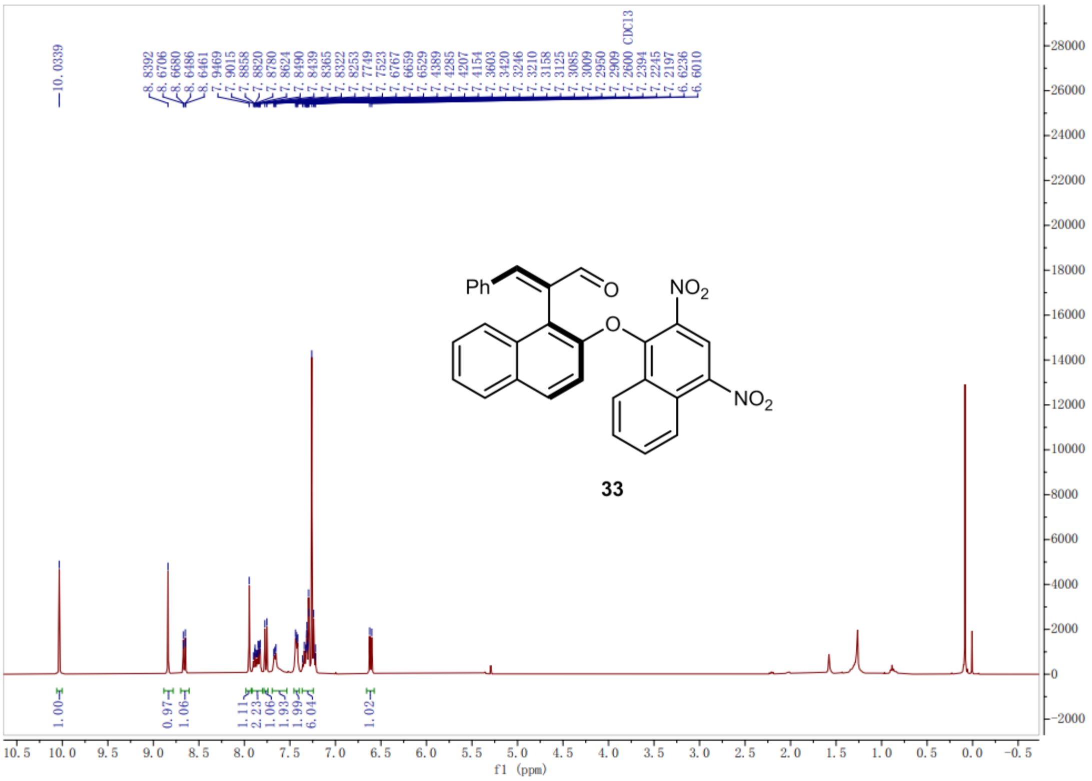

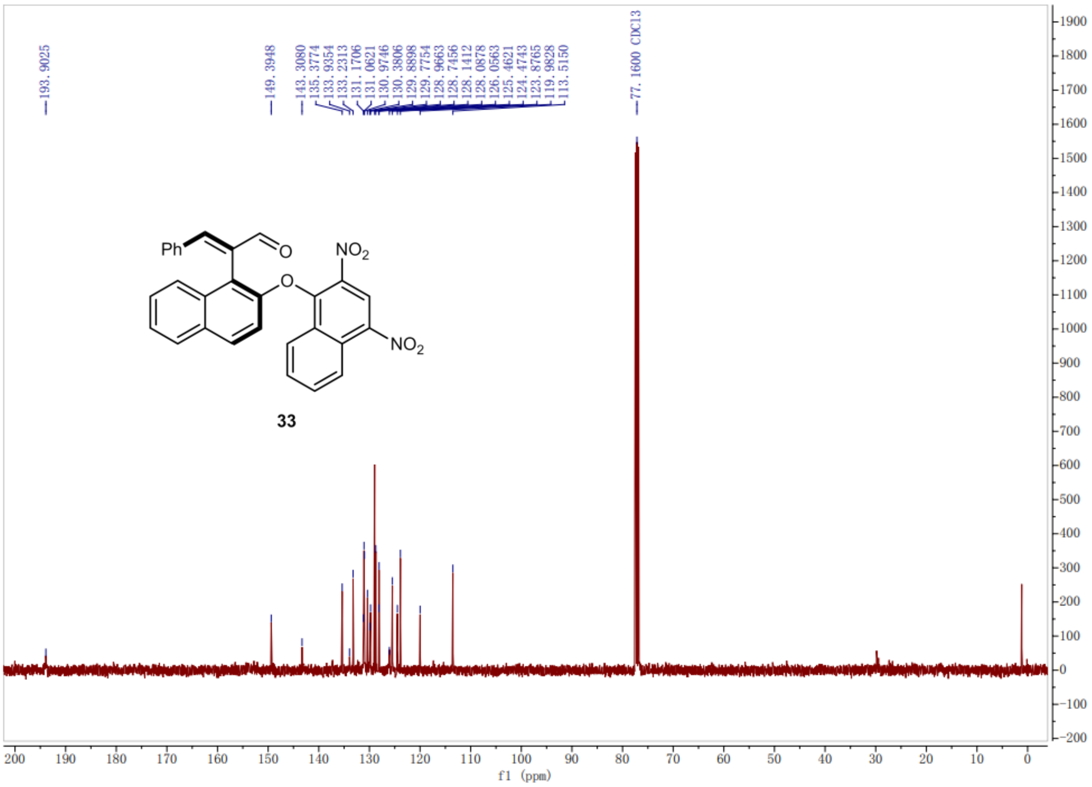

NMR of **34** ($CDCl_3$)

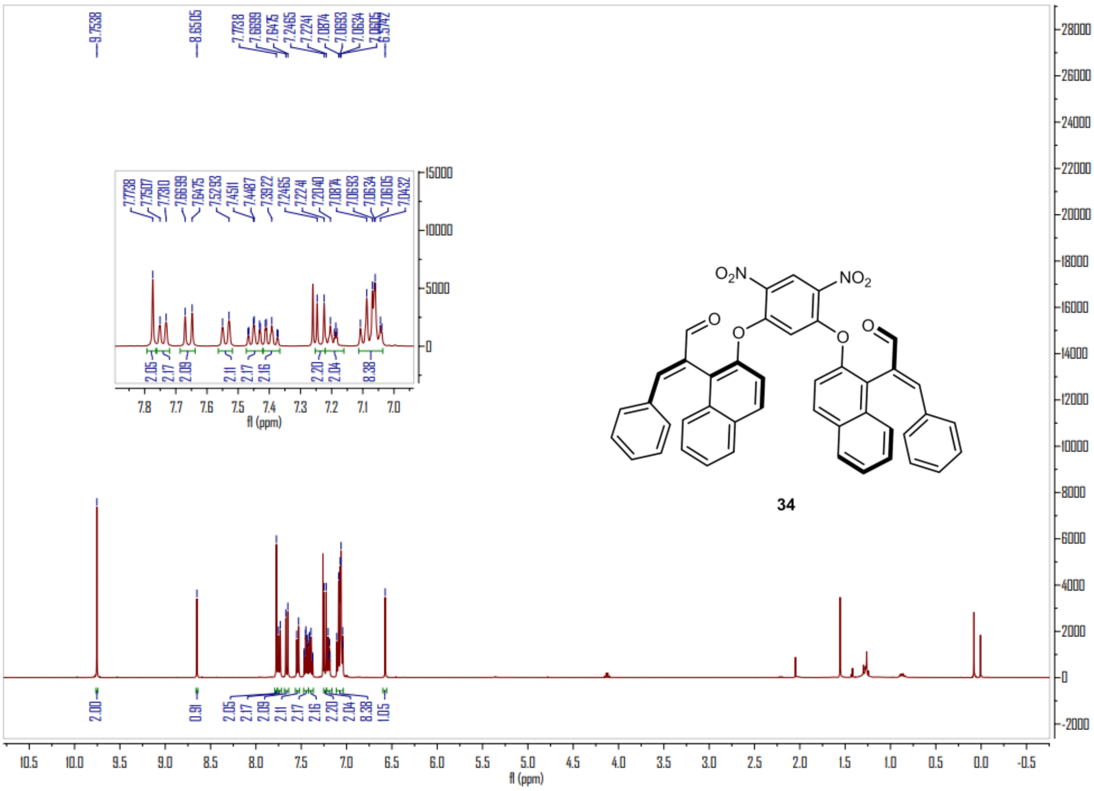

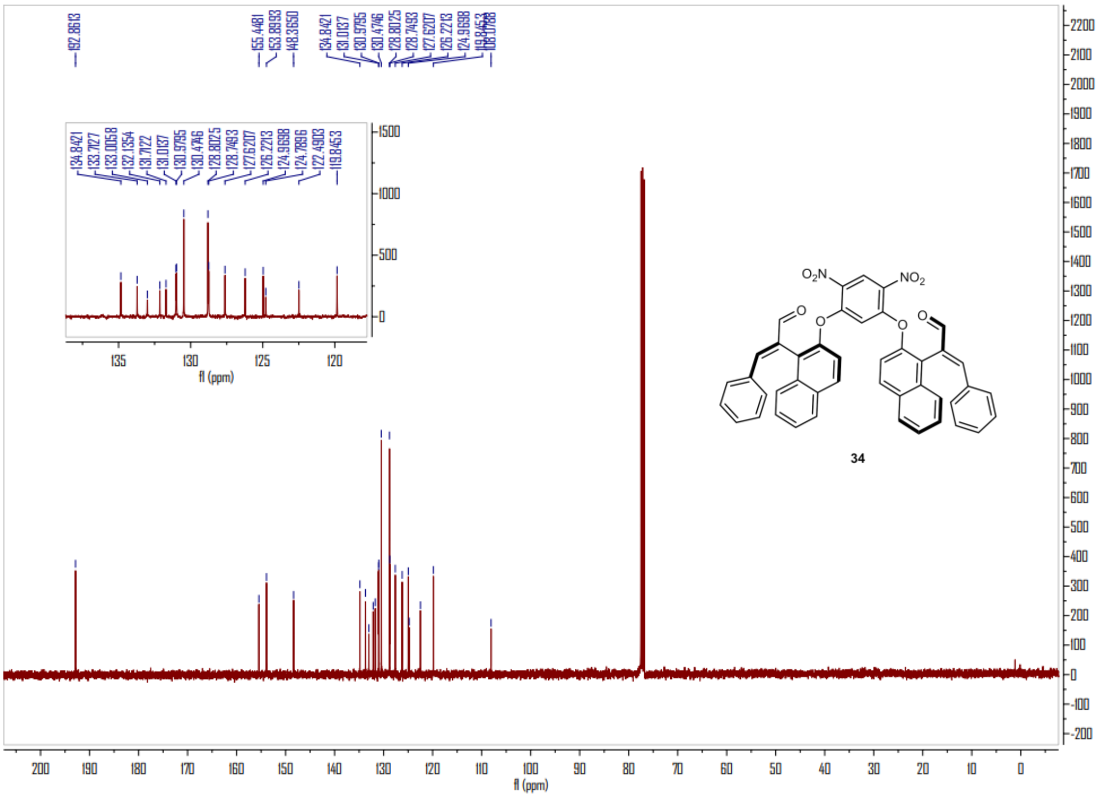

NMR of **35** ($CDCl_3$)

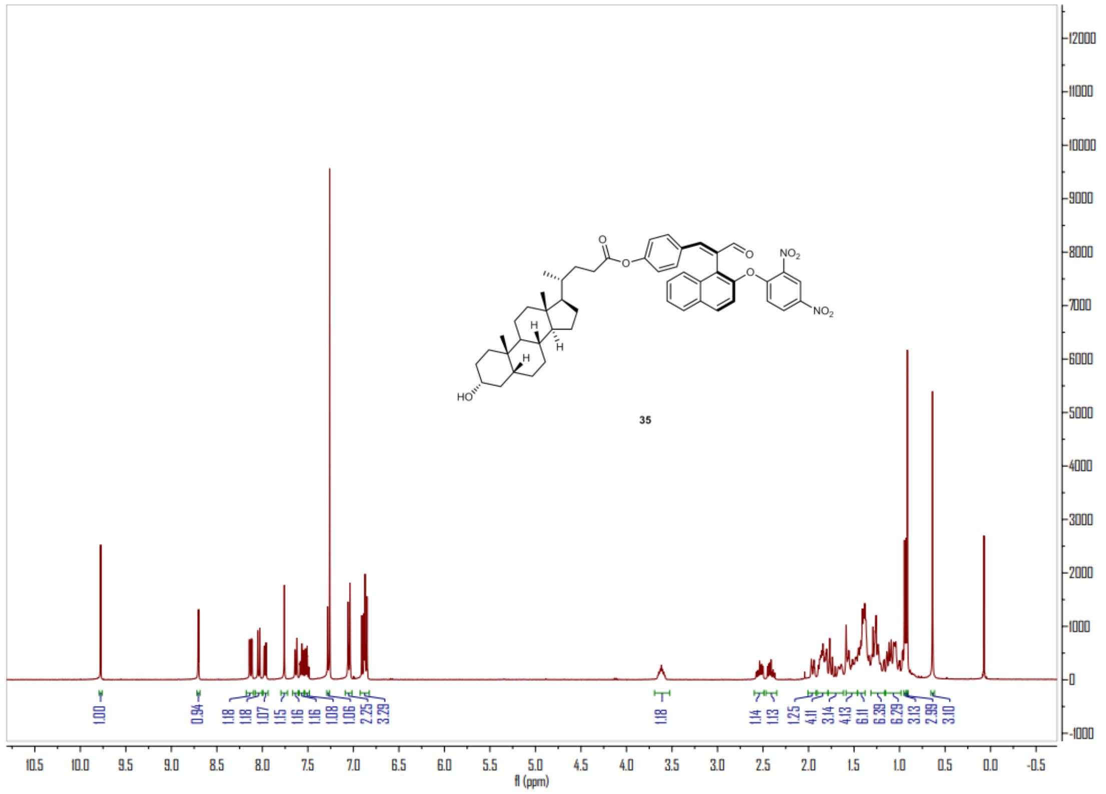

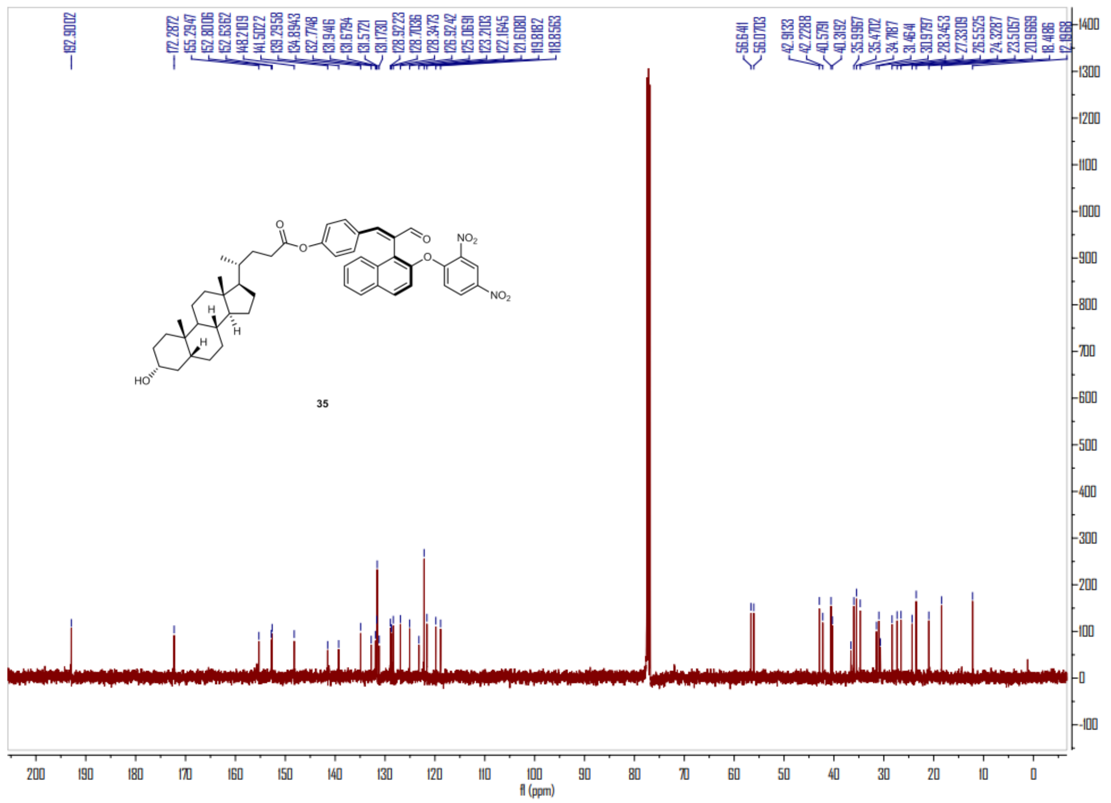

NMR of **36** ($CDCl_3$)

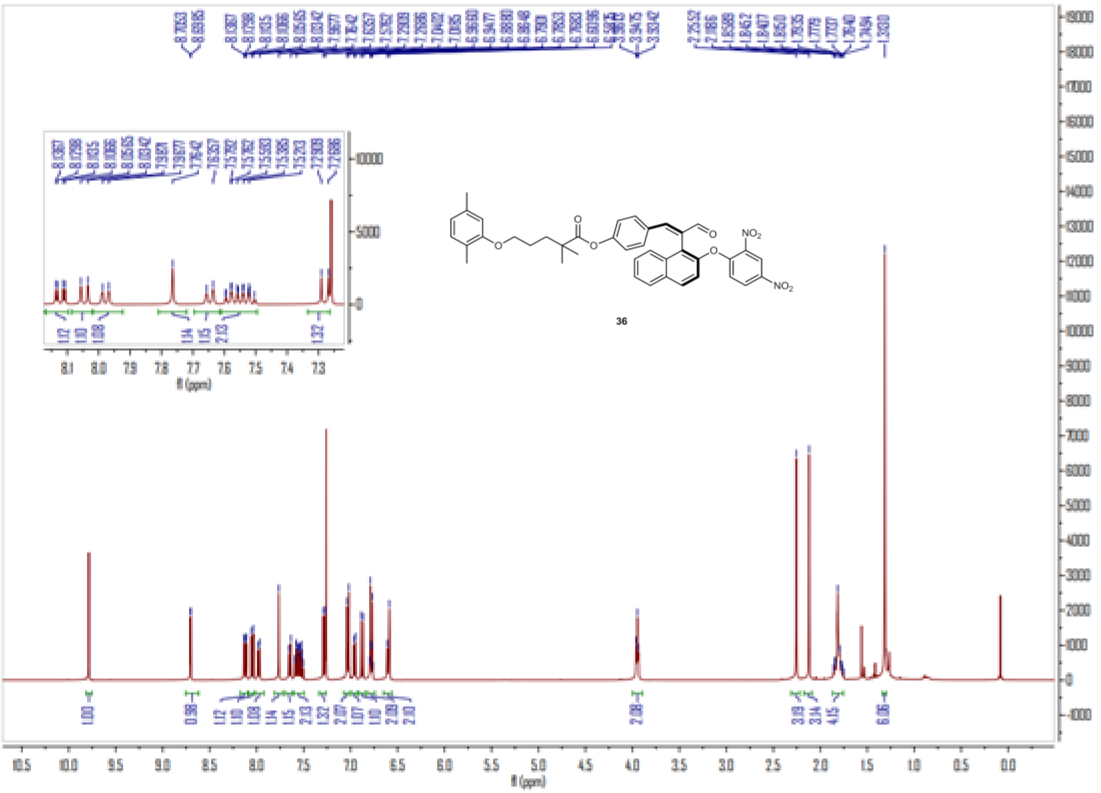

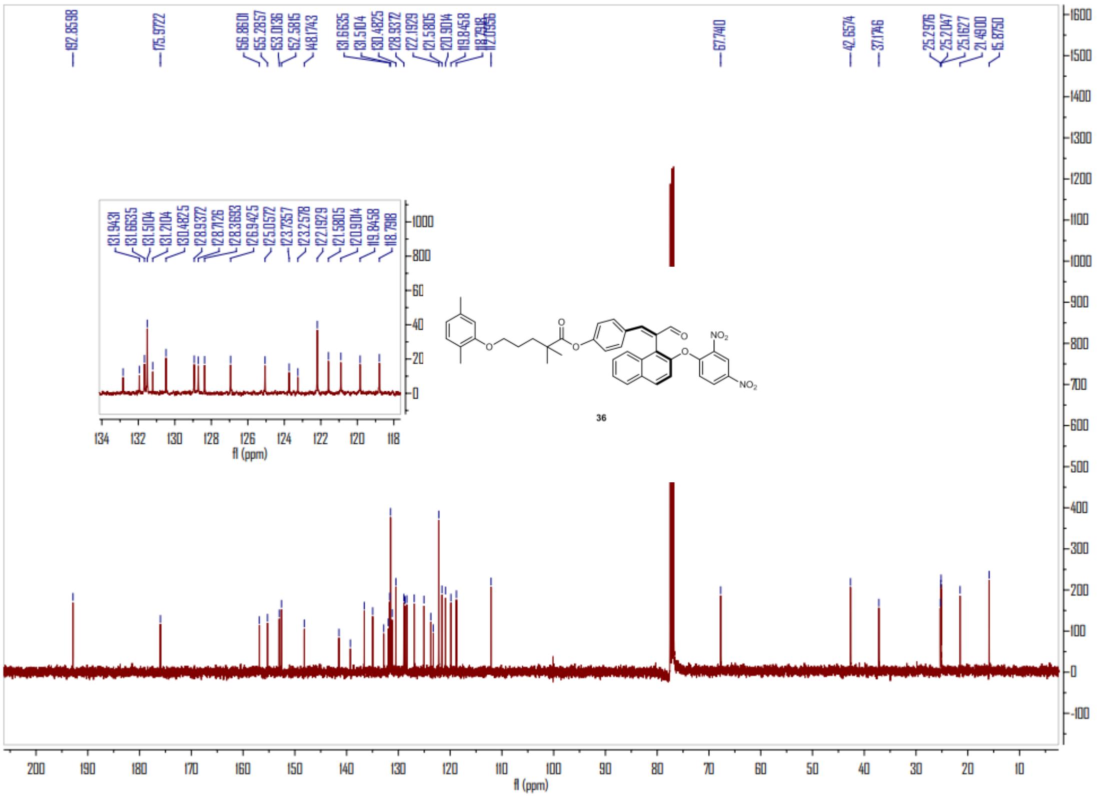

NMR of **37** ($CDCl_3$)

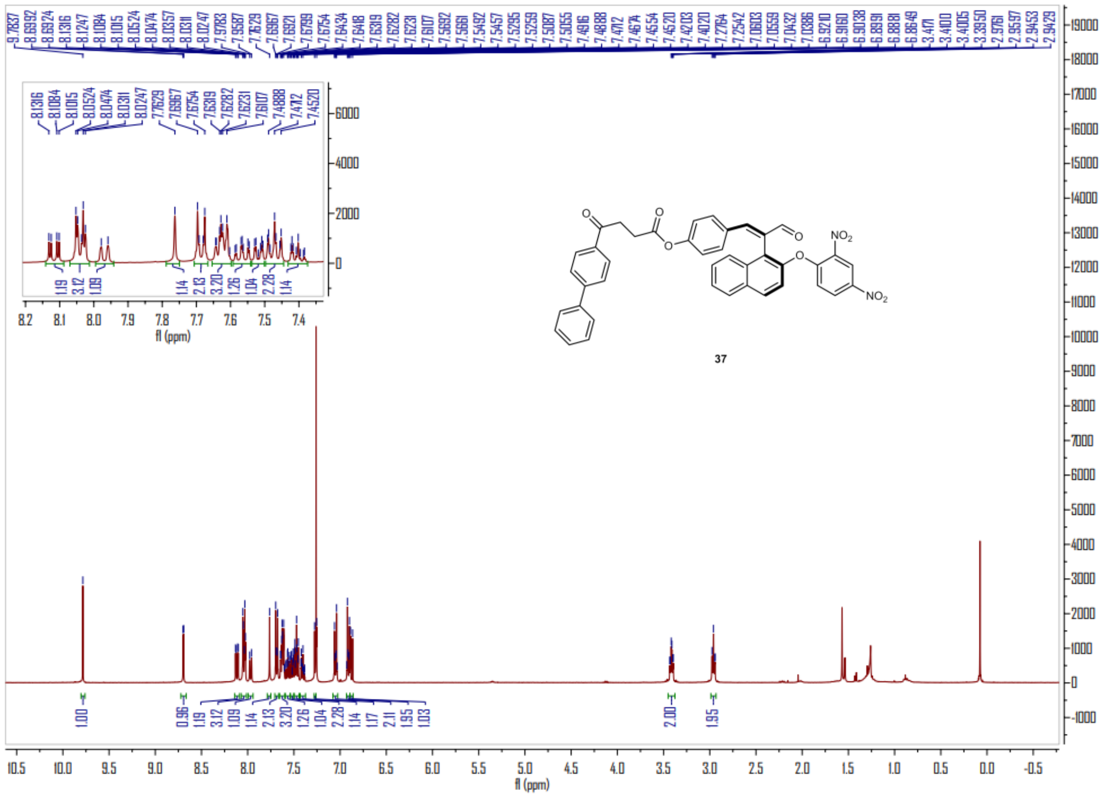

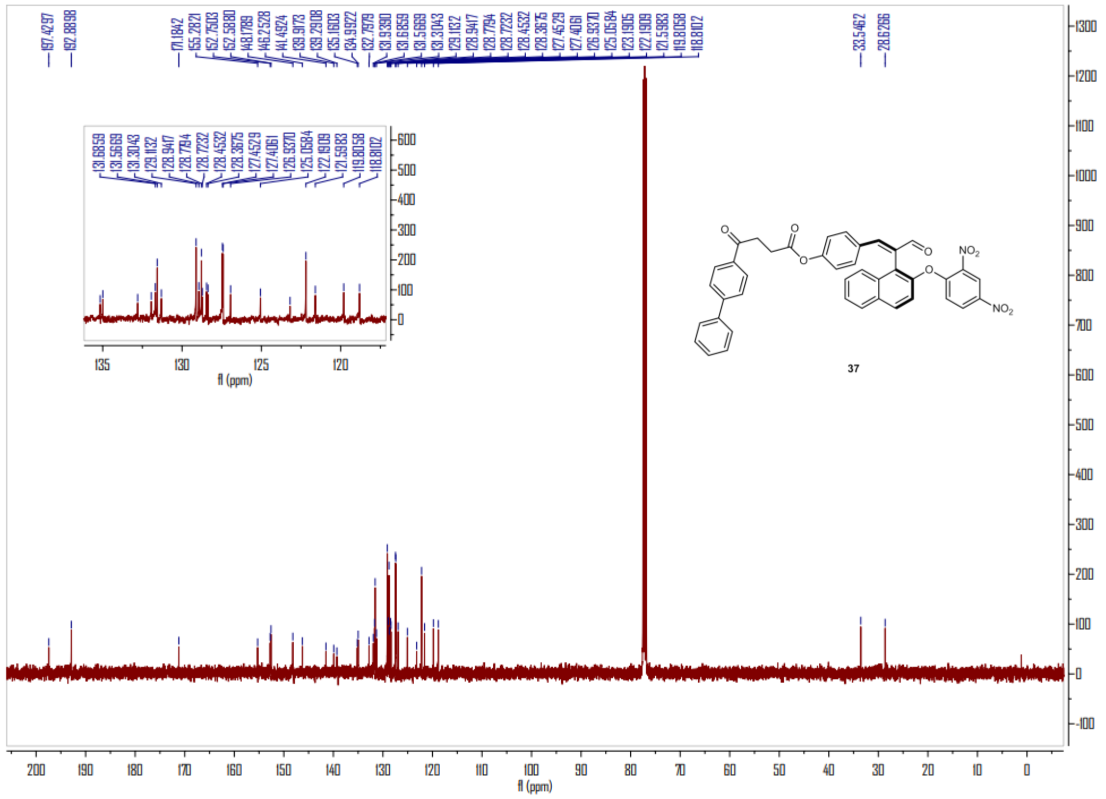

NMR of **38** ($CDCl_3$)

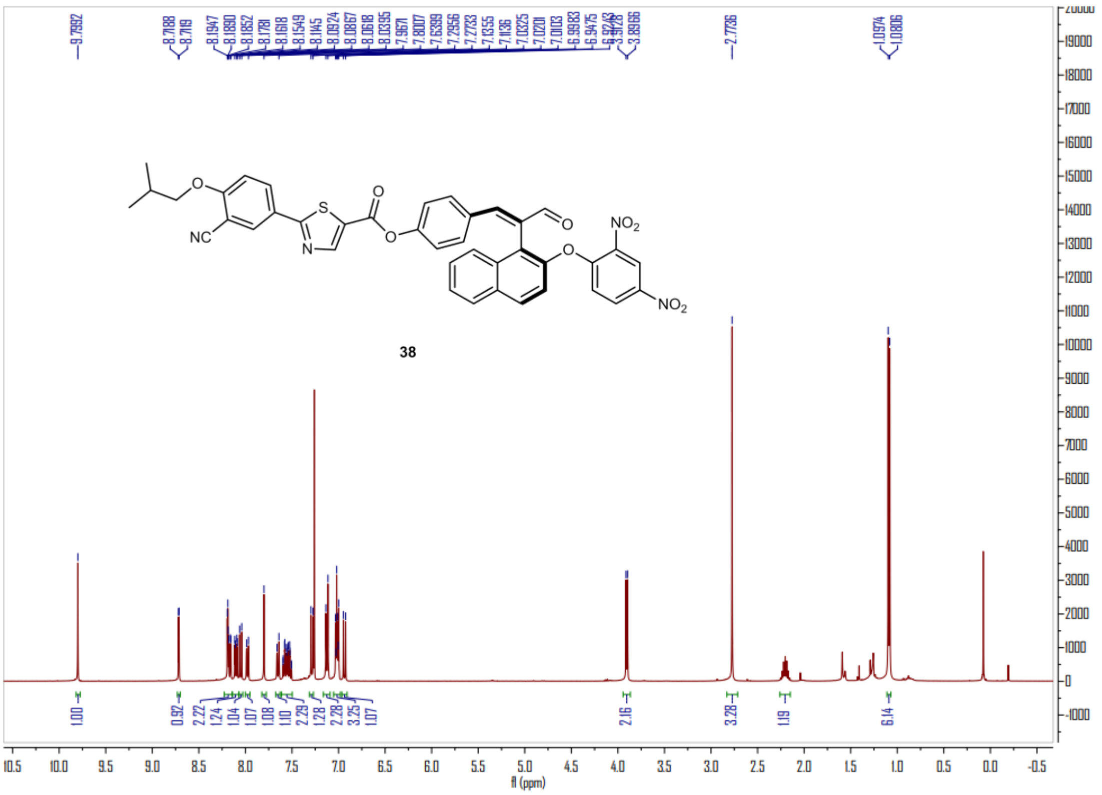

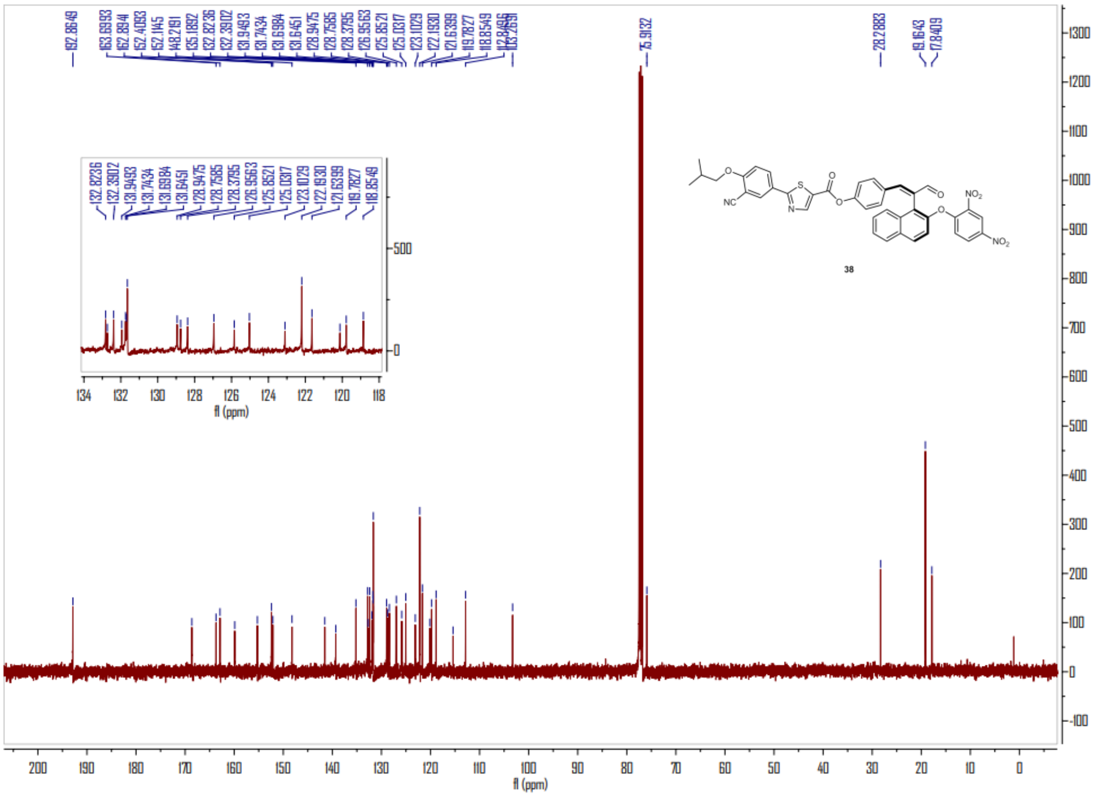

NMR of **39** ($CDCl_3$)

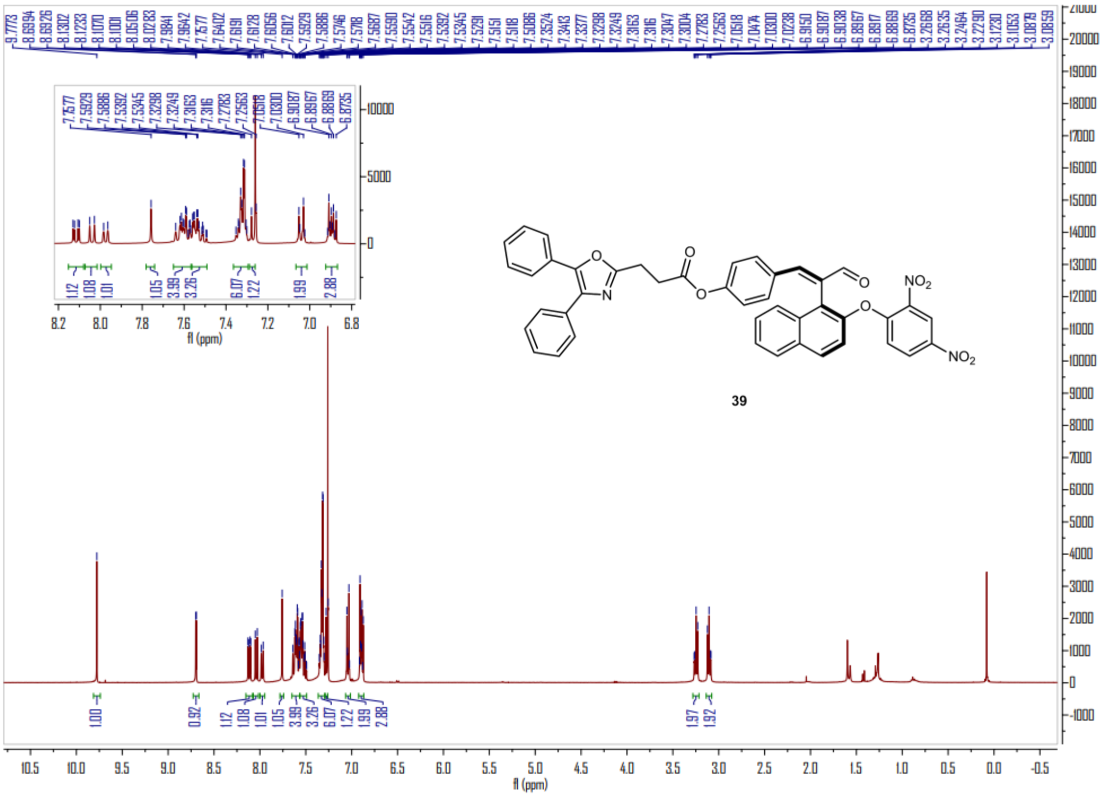

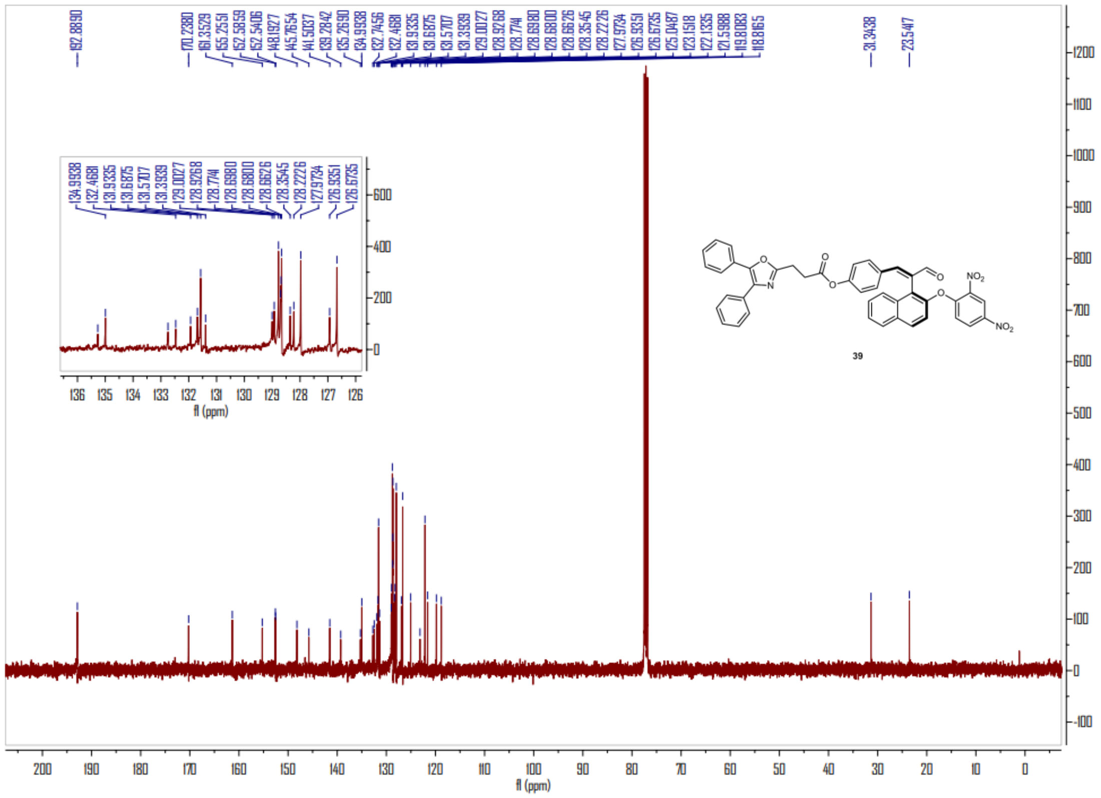

NMR of **40** ($CDCl_3$)

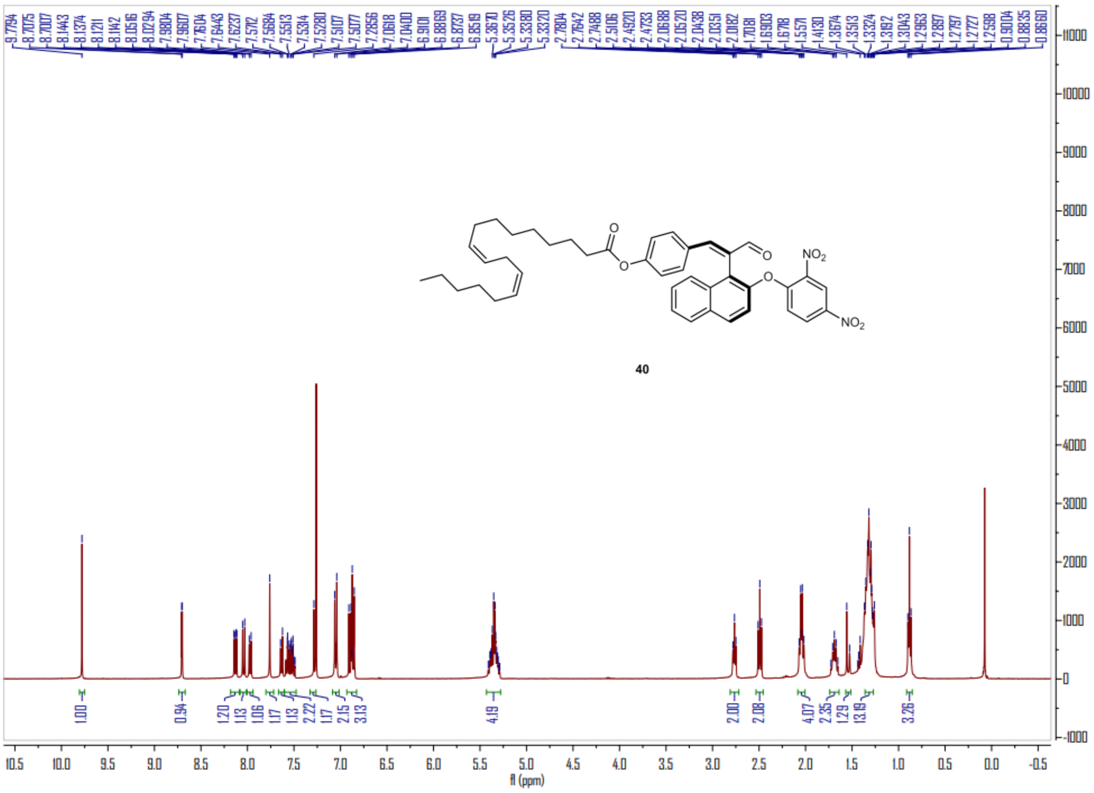

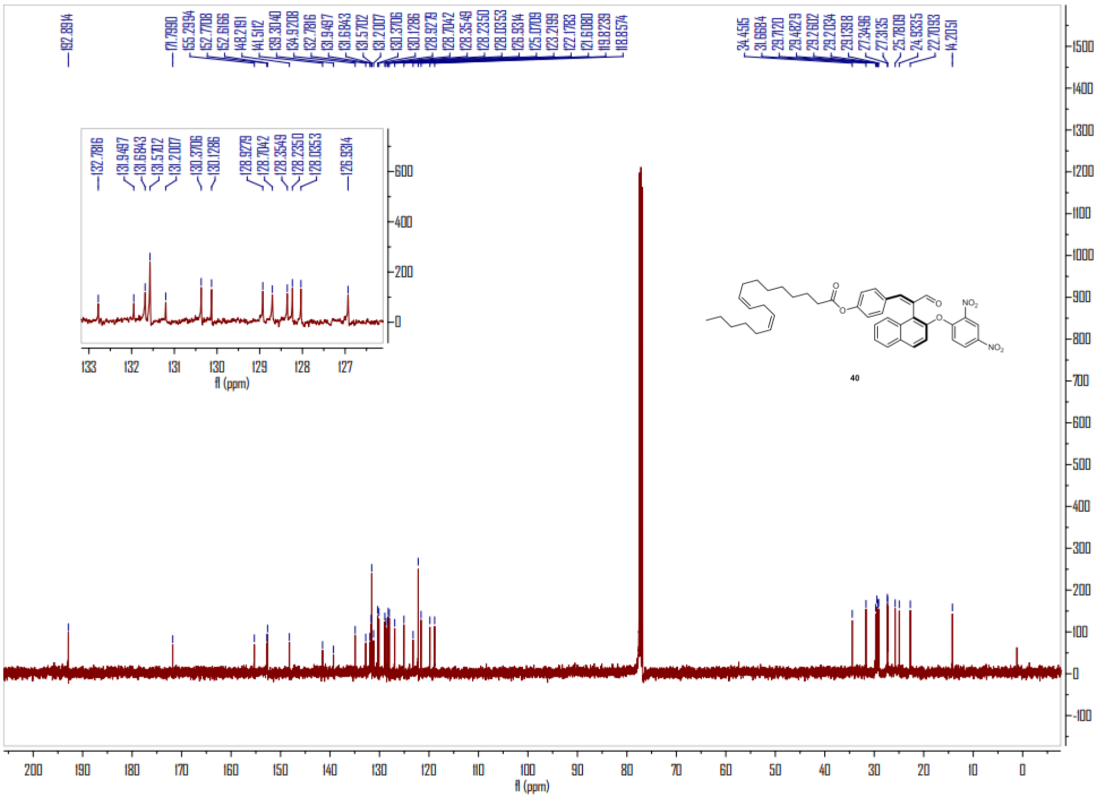

NMR of **41** ($CDCl_3$)

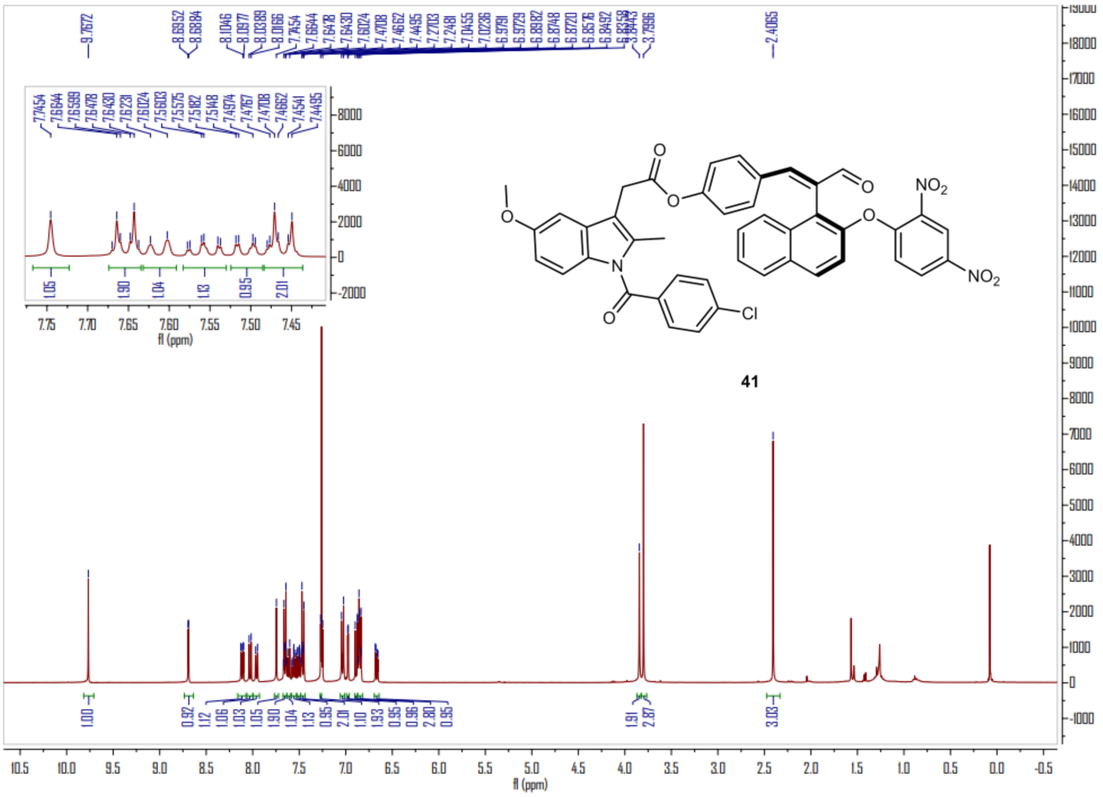

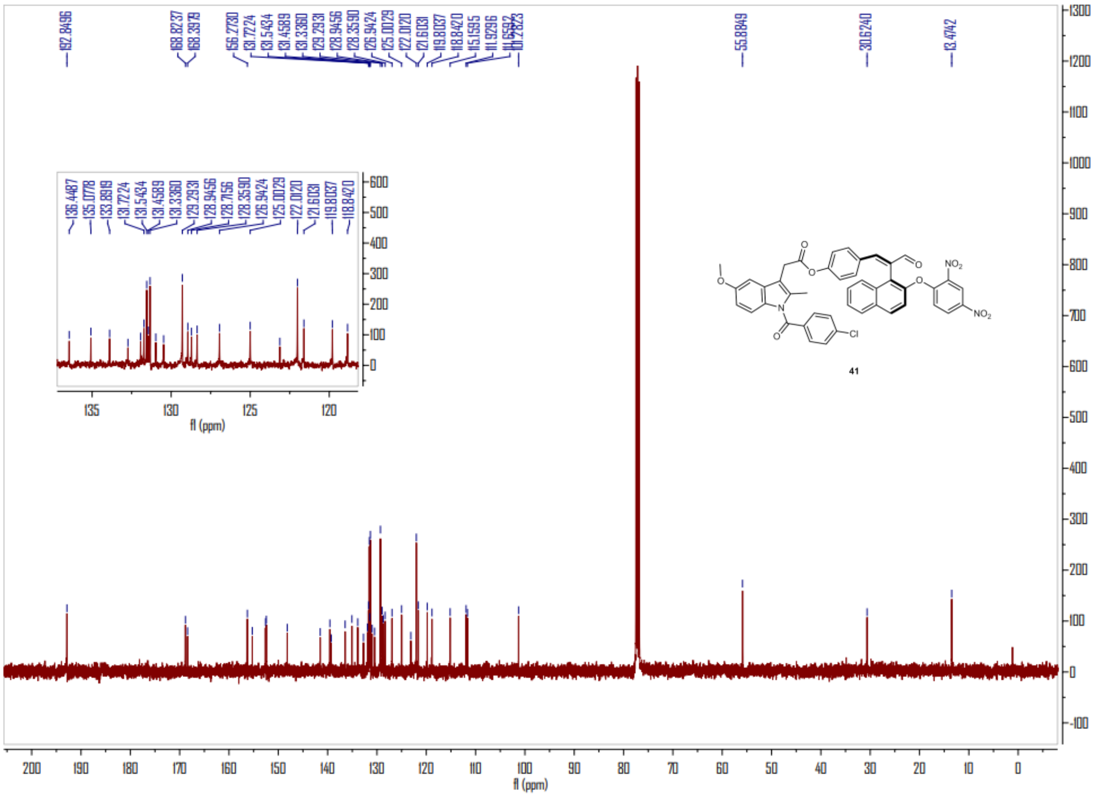

NMR of **42** ($CDCl_3$)

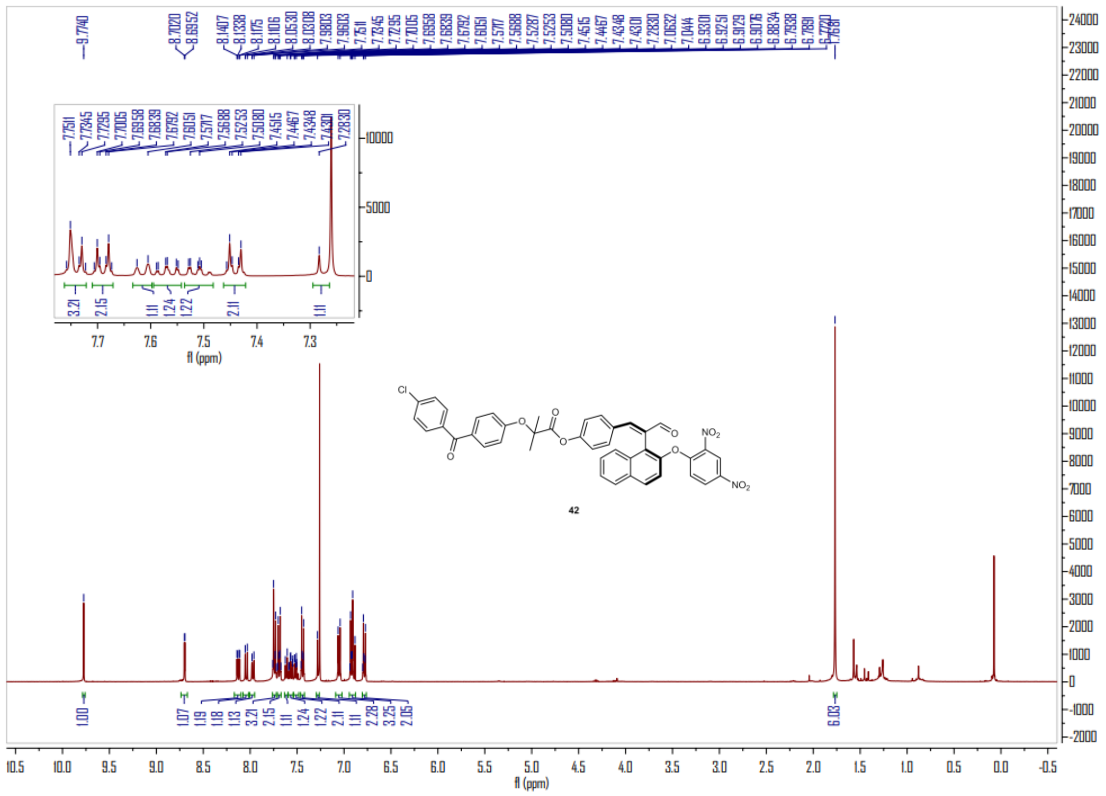

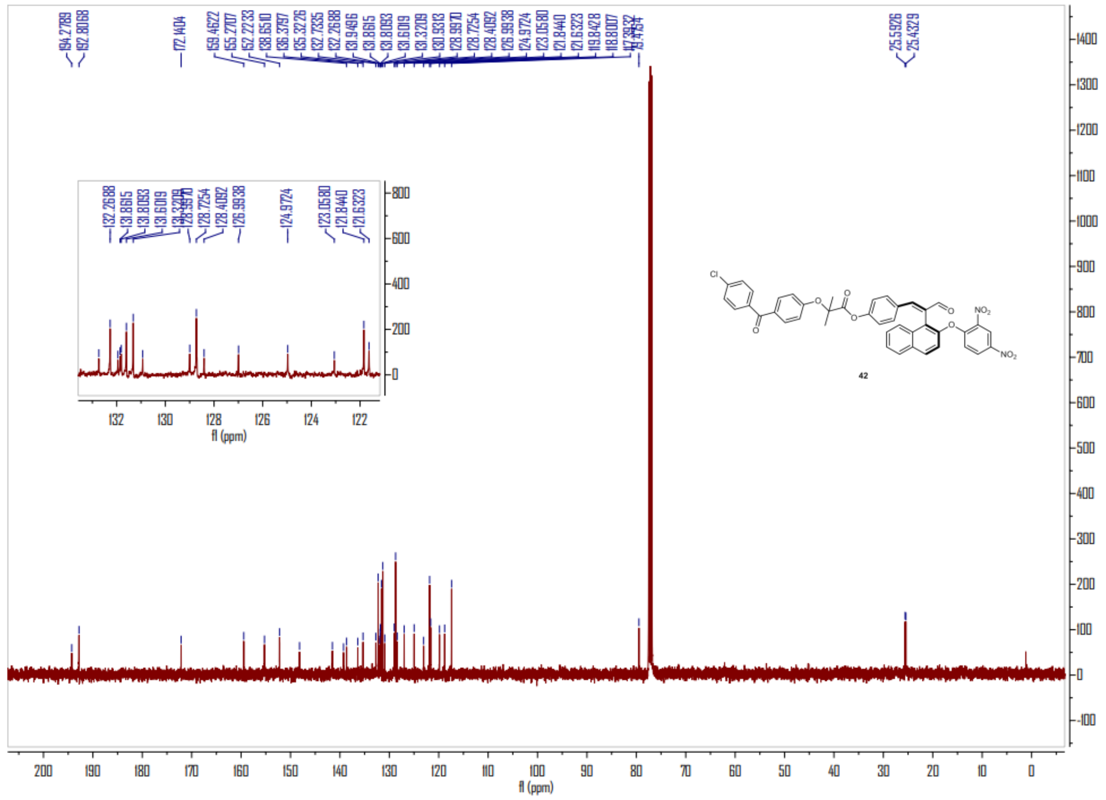

NMR of **43** ($CDCl_3$)

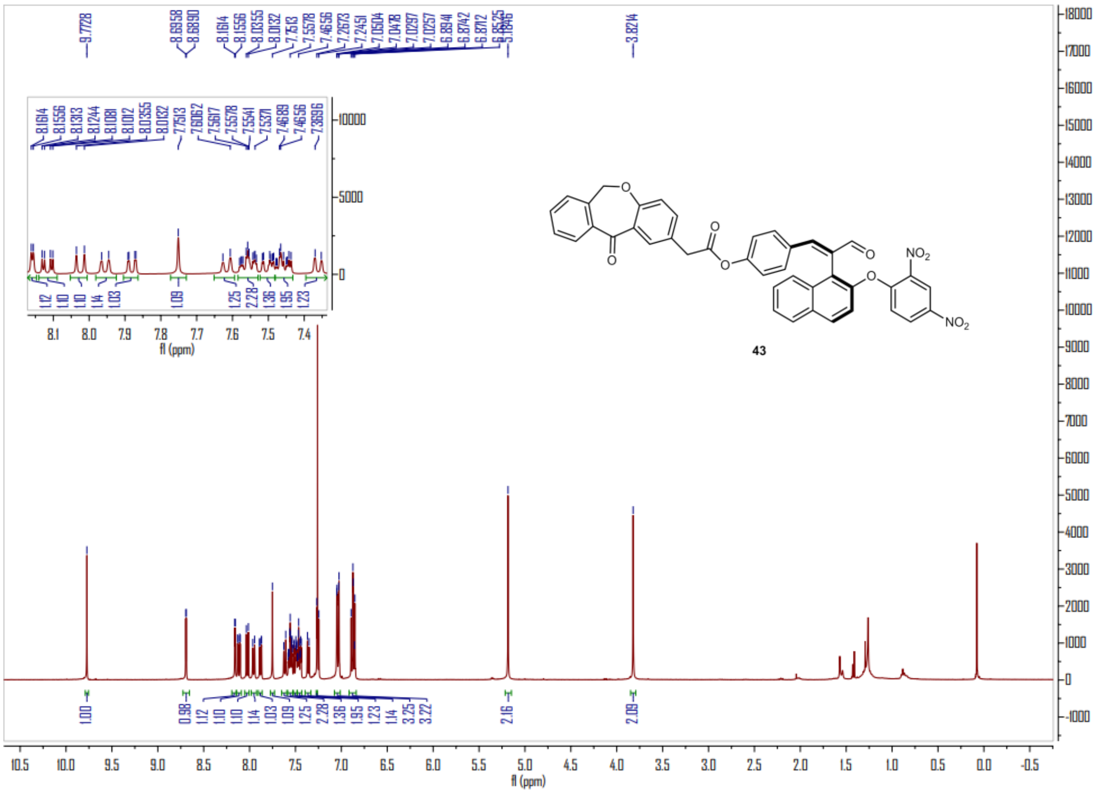

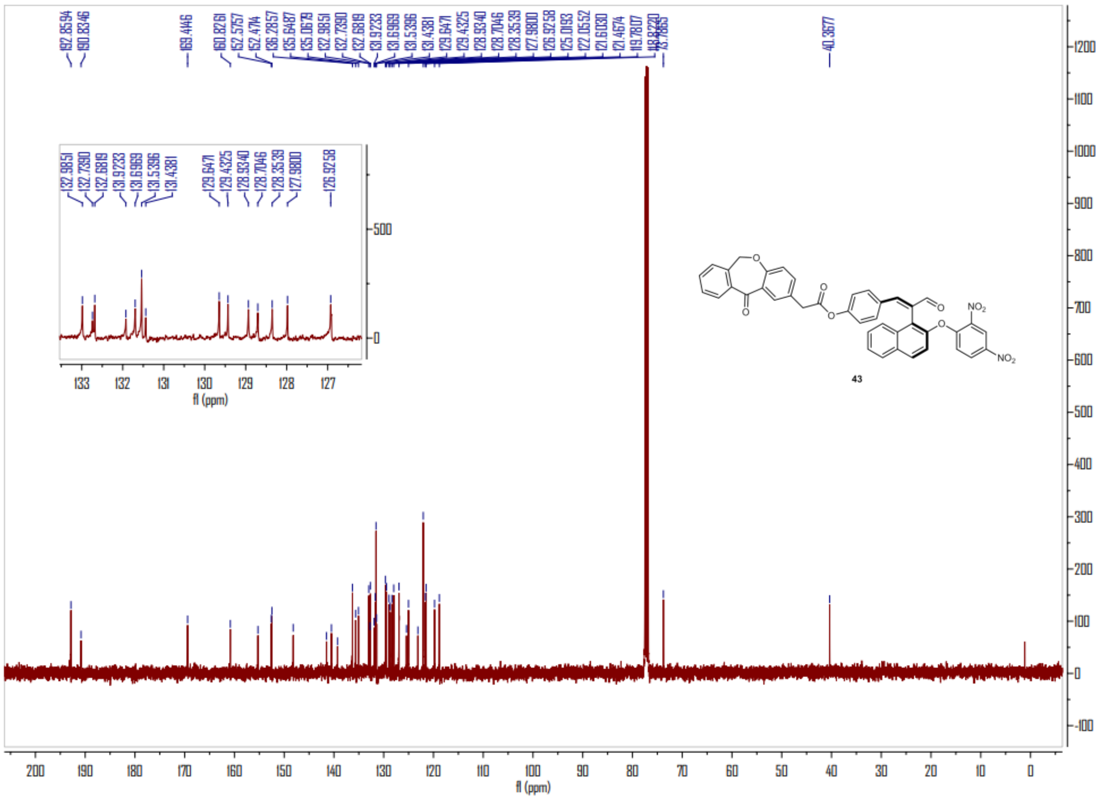

NMR of **44** ($CDCl_3$)

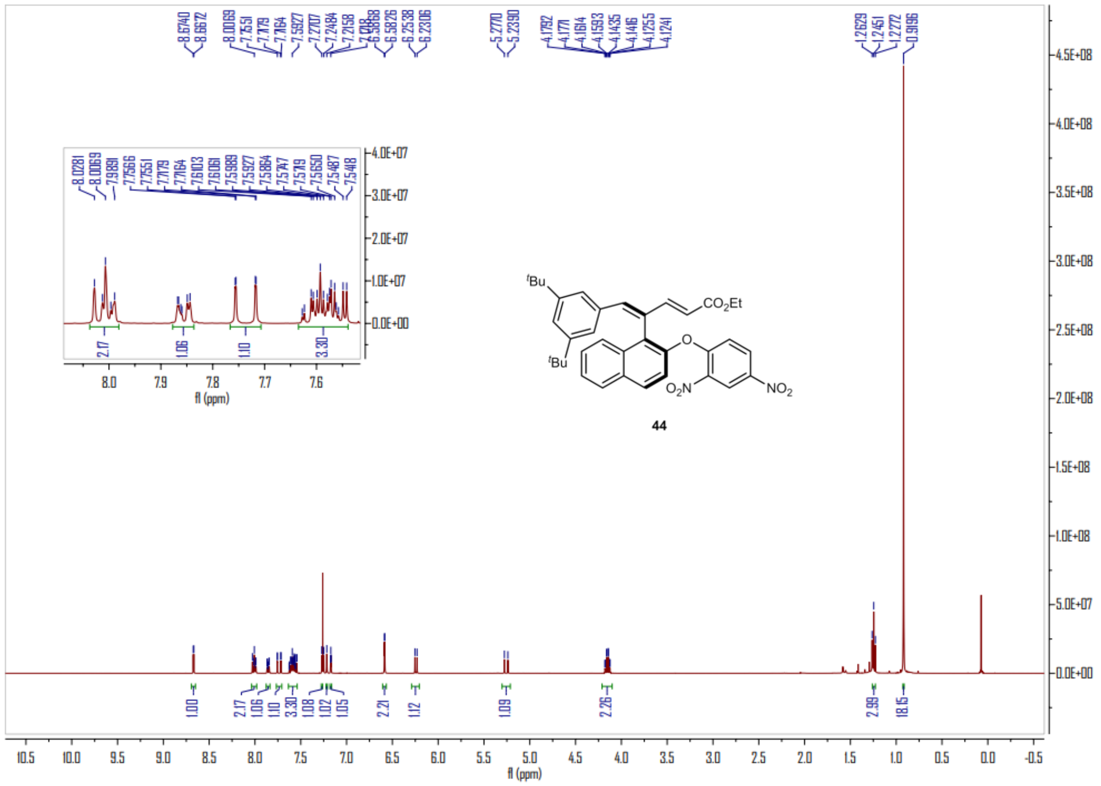

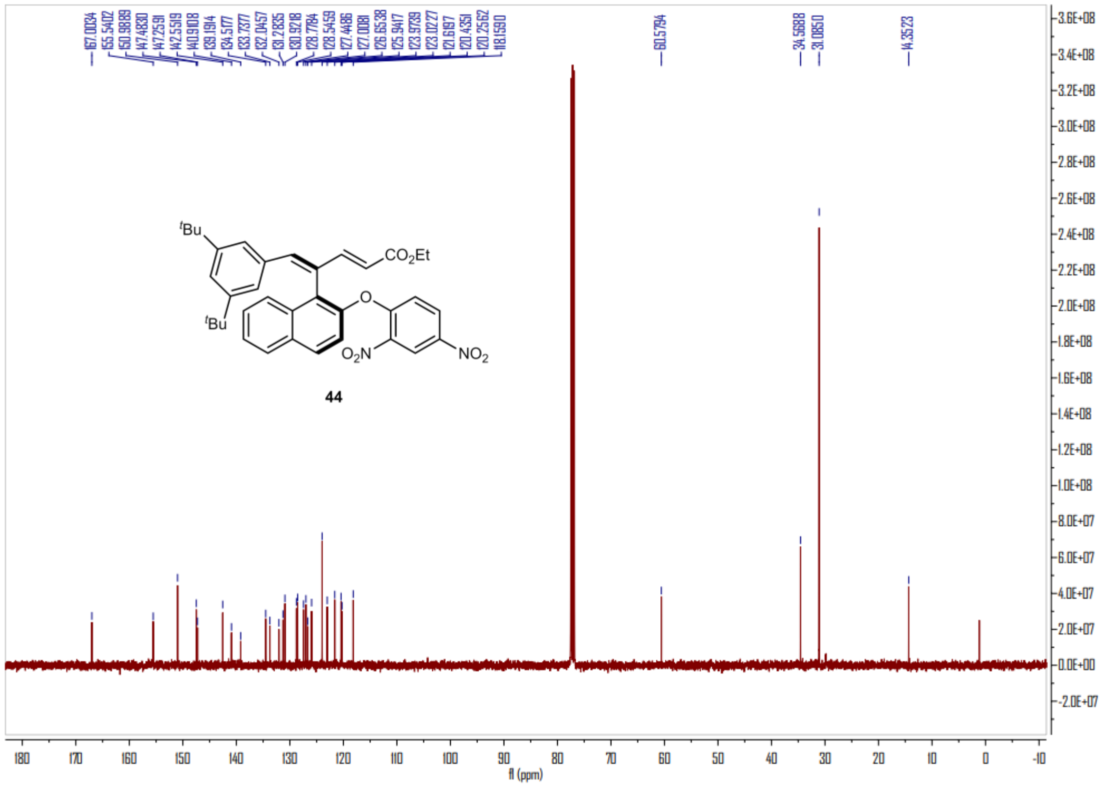

NMR of **45** ($CDCl_3$)

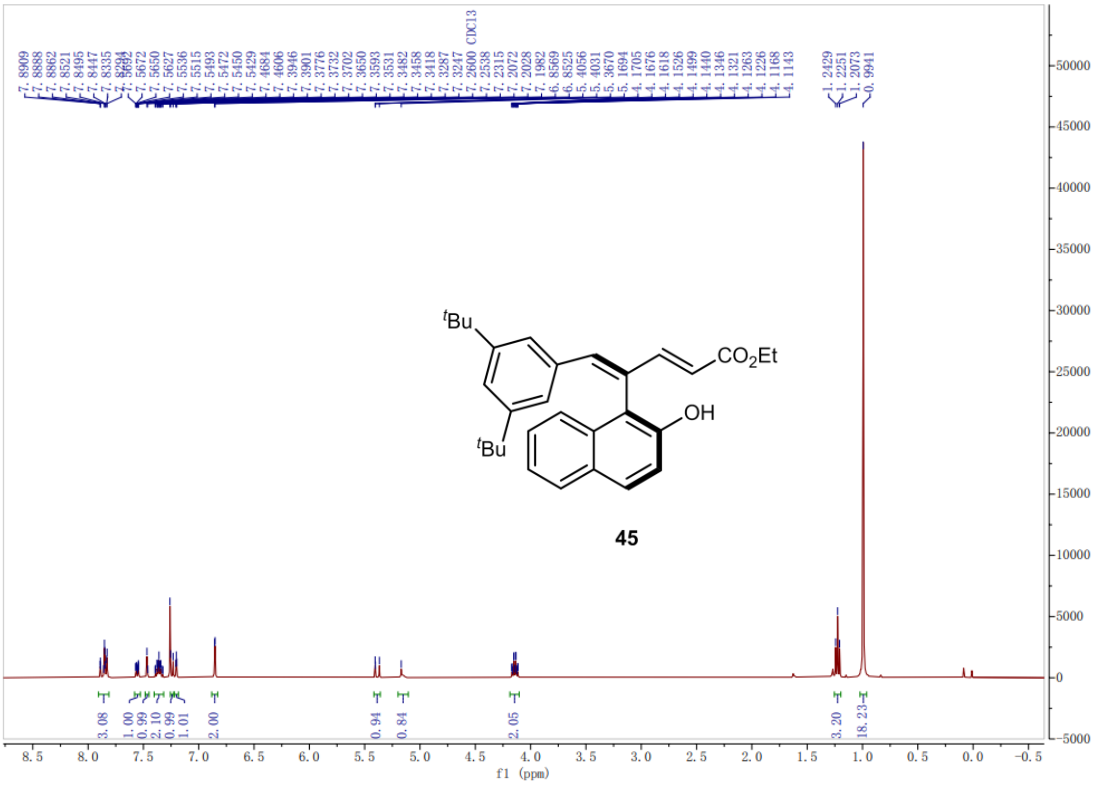

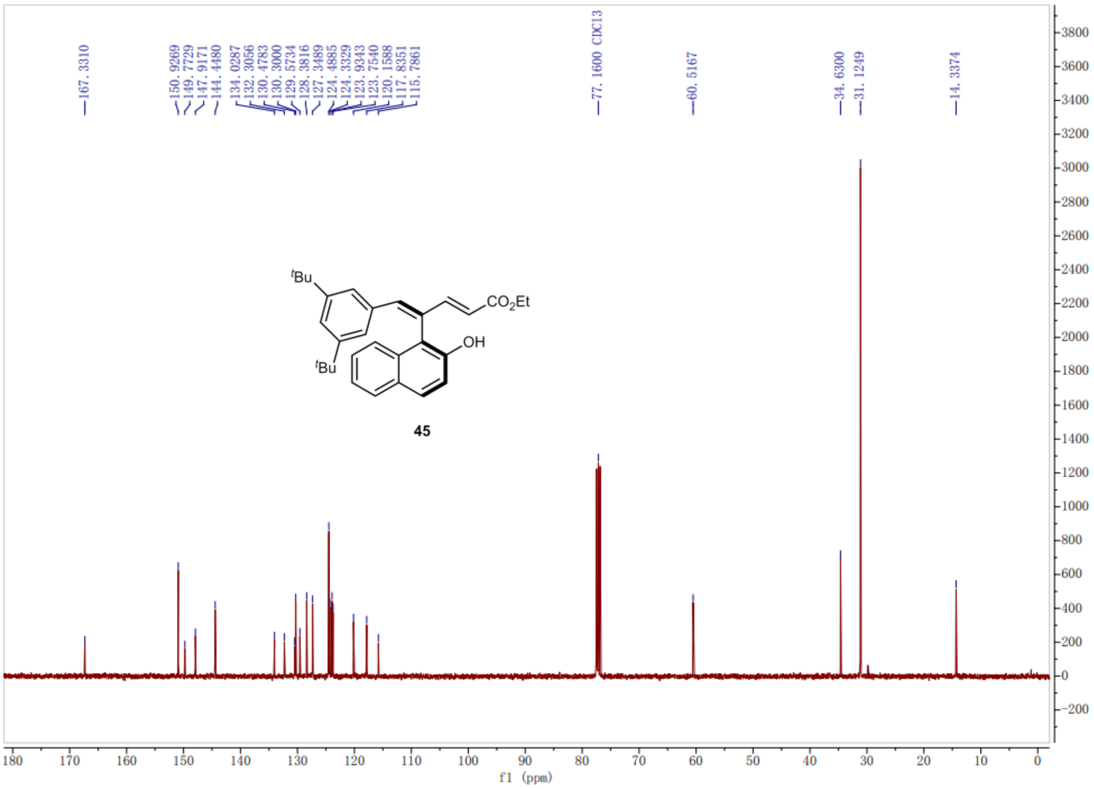

NMR of **46** ($CDCl_3$)

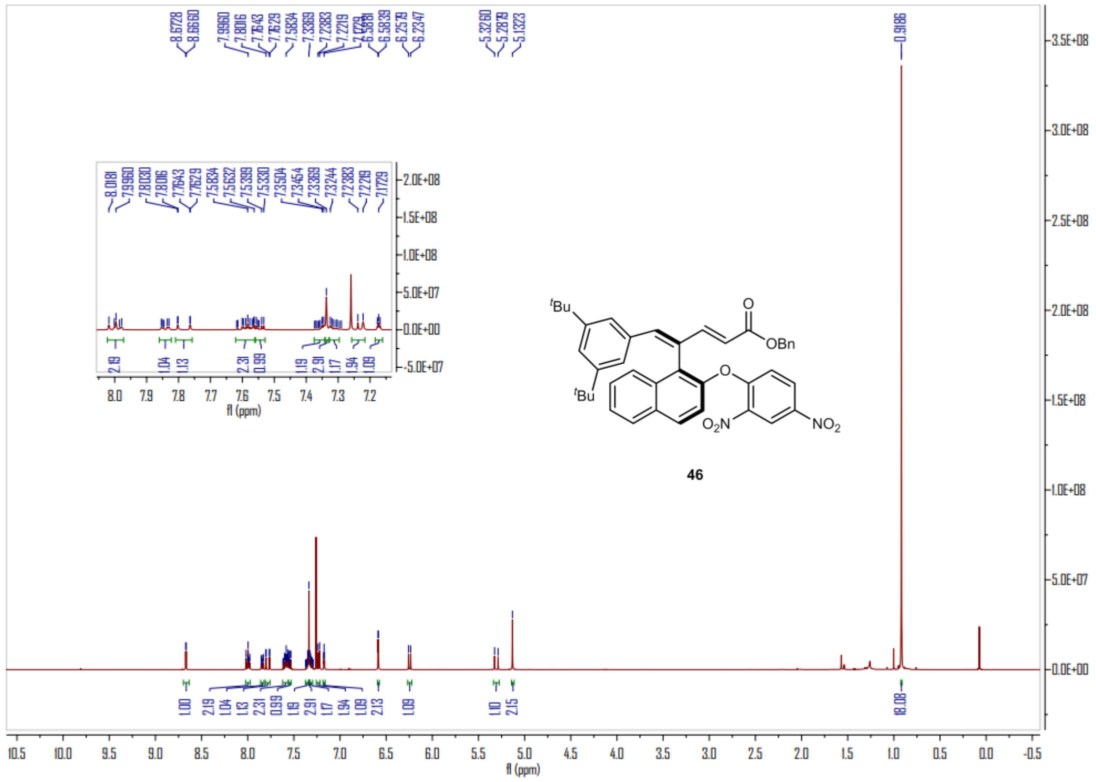

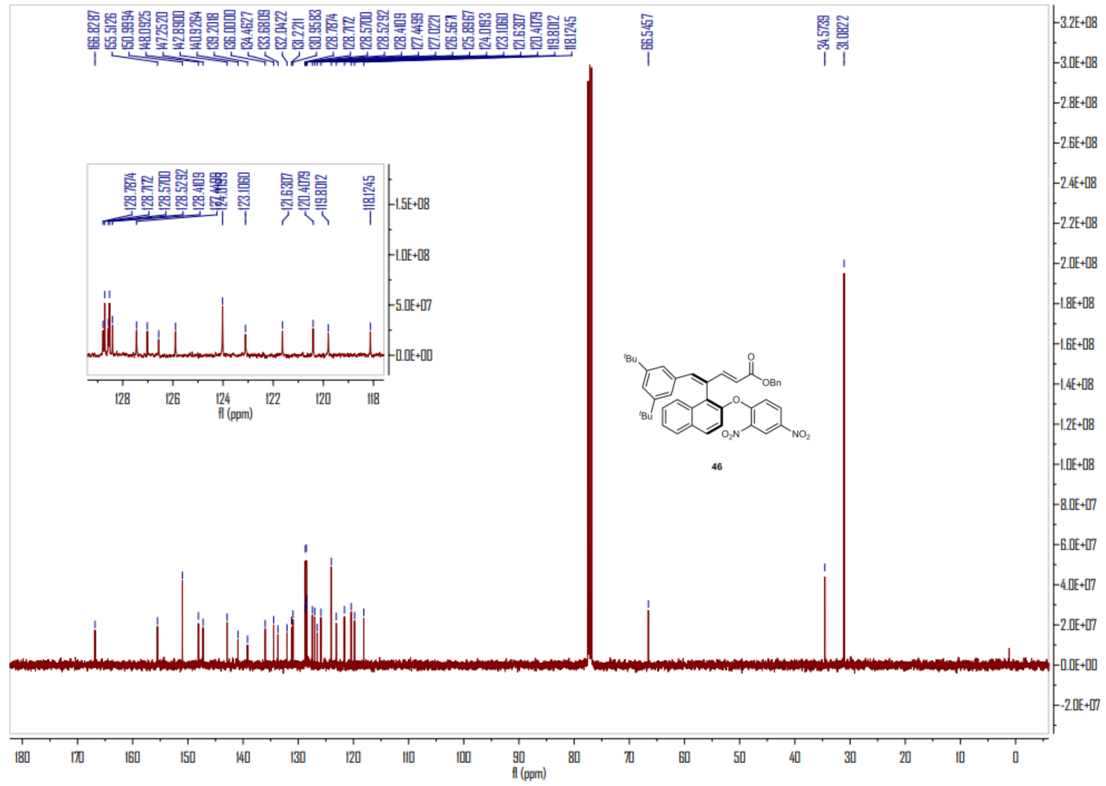

NMR of **47** ($CDCl_3$)

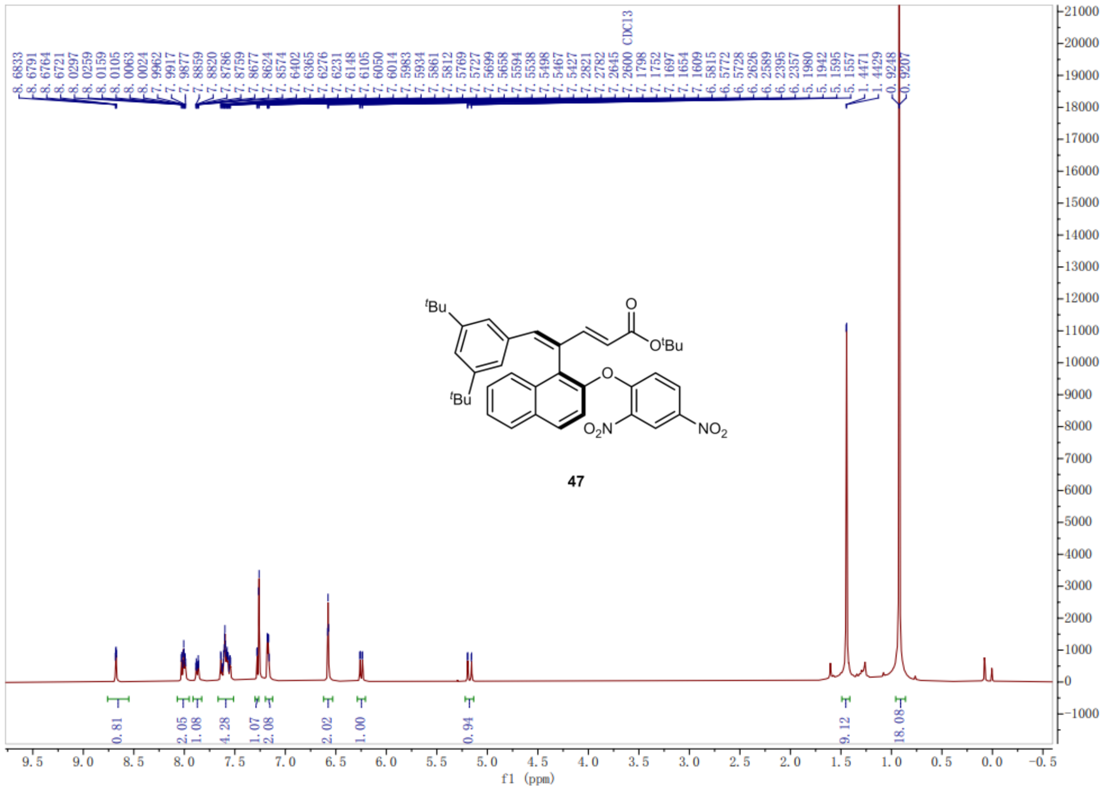

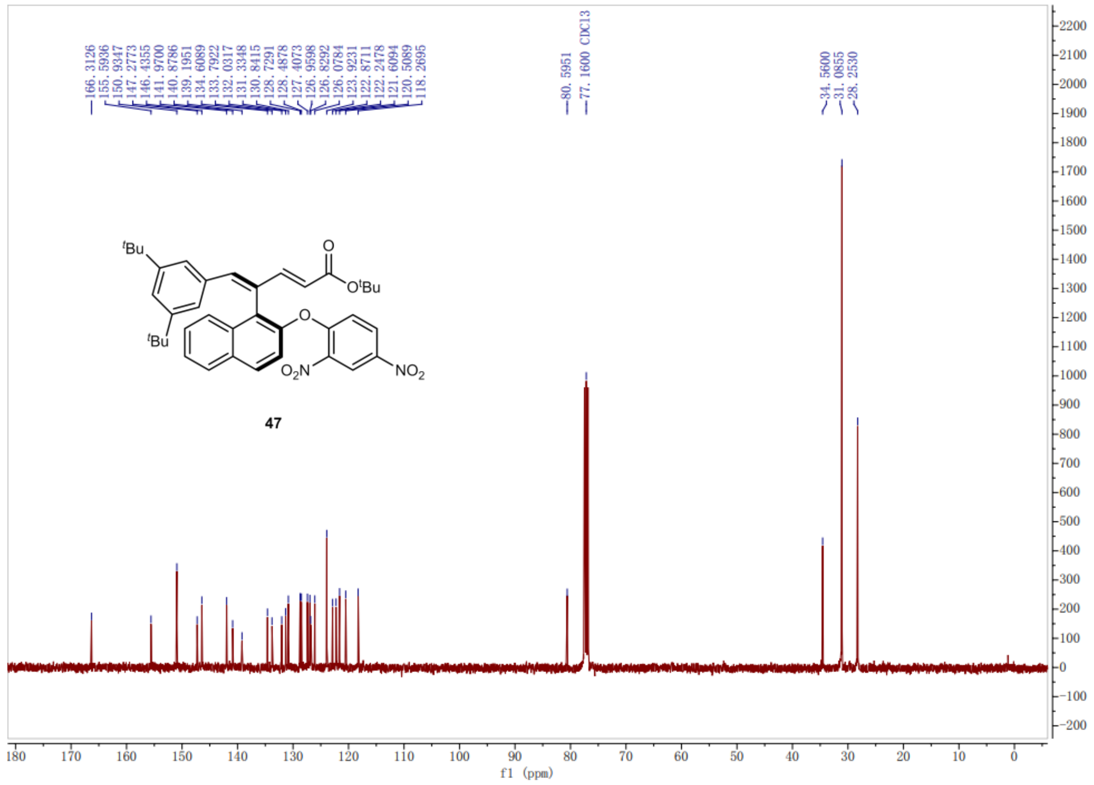

NMR of **48** ($CDCl_3$)

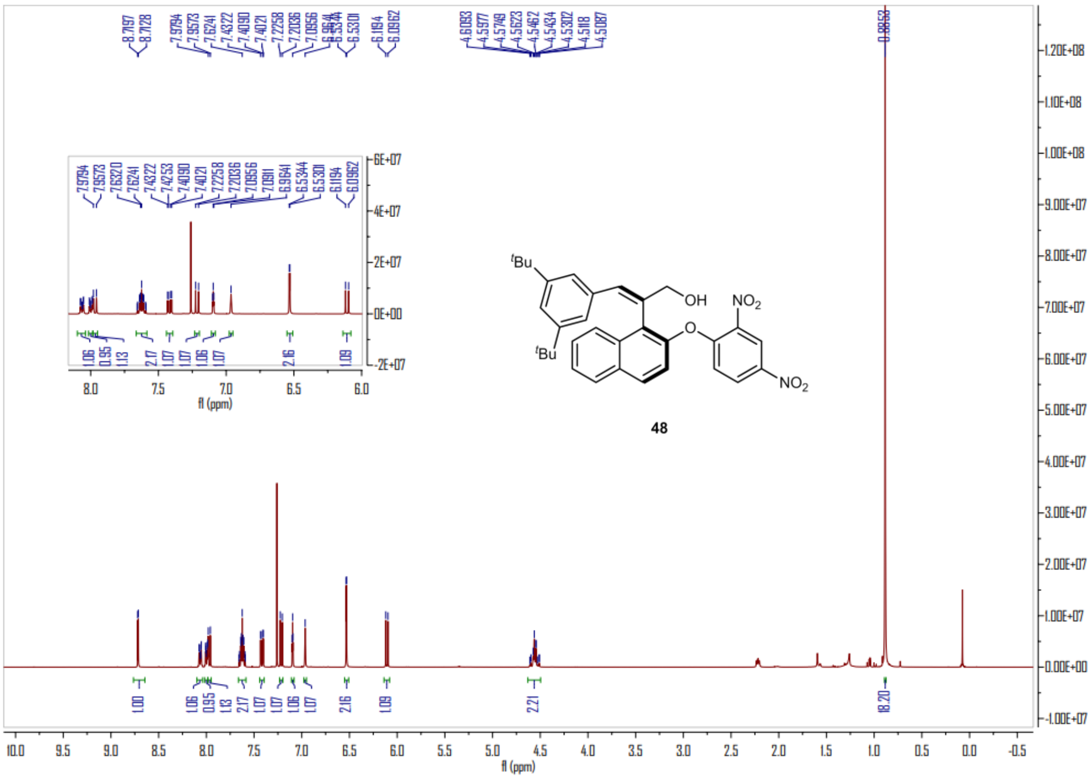

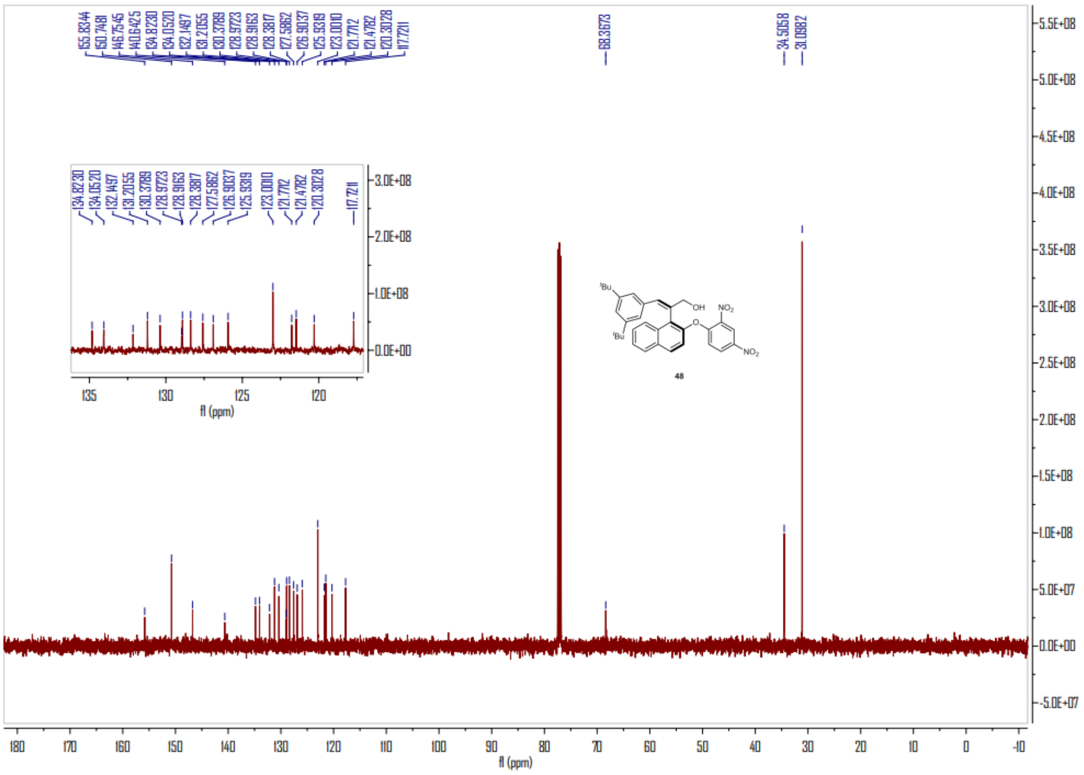

NMR of **49** ($CDCl_3$)

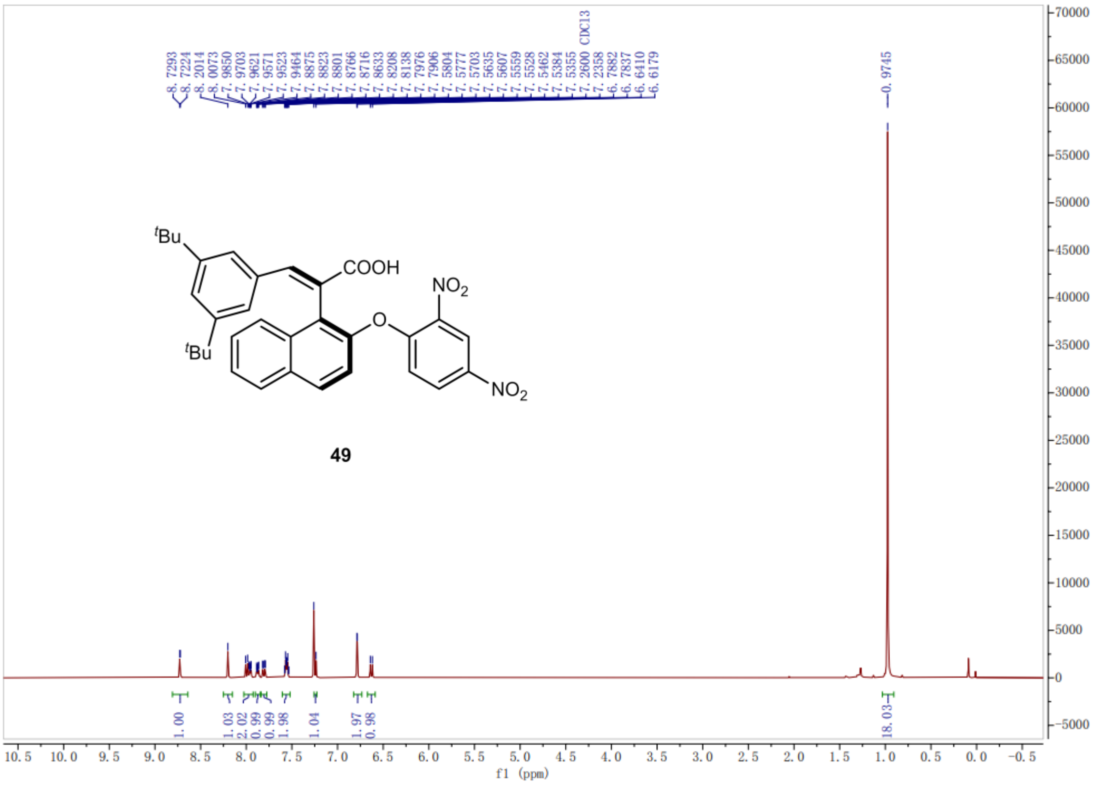

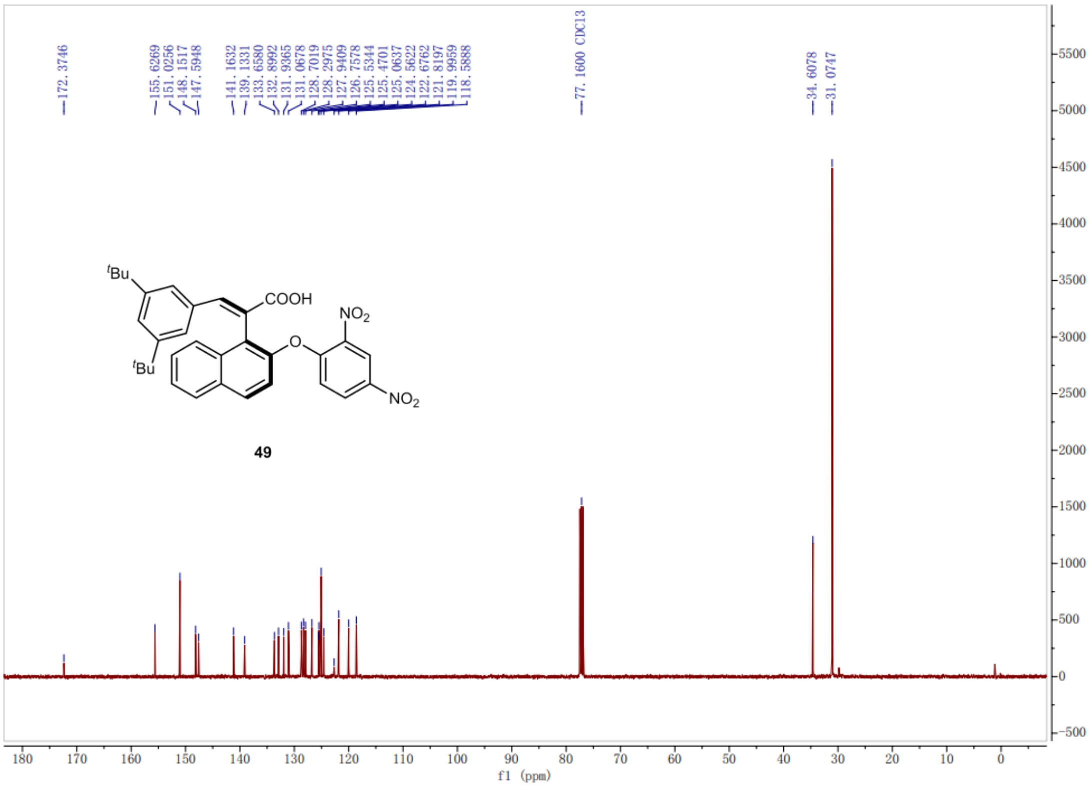

12. Supplementary References

[1] Y-D, S. et al. Construction of axially chiral styrene-type allylamines via chiral phosphoric acid-catalyzed asymmetric reductive amination. *Org. Chem. Front*, **9**, 764–770 (2022).

[2] Zeng, G. et al. Enantioselective Construction of Quinoxaline-Based Heterobiaryls and P,N-Ligands via Chirality Transfer Strategy. *Org. Lett.* **23**,1181–1187 (2021).

[3] David, B, G. & Dennis, P, C. Asymmetric Radical and Anionic Cyclizations of Axially Chiral Carbamates *Org. Lett.* **11**, 249–251 (2009).

[4] M. J. Frisch, G. W. Trucks, H. B. Schlegel, G. E. Scuseria, M. A. Robb, J. R. Cheeseman, G. Scalmani, V. Barone, B Mennucci, G. A. Petersson, H. Nakatsuji, M. Li, X. Caricato, H. P. Hratchian, A. F. Izmaylov, J. Bloino, G. Zheng, J. L. Sonnenberg, M. Hada, M. Ehara, K. Toyota, R. Fukuda, J. Hasegawa, M. Ishida, T. Nakajima, Y. Honda, O. Kitao, H. Nakai, T. Vreven, J. A. Montgomery, J. J. E. Peralta, F. Ogliaro, M. Bearpark, J. J. Heyd, E. Brothers, K. N. Kudin, V. N. Taroverov, T. Keith, R. Kobayashi, J. Normand, K. Raghavachari, A. Rendell, J. C. Burant, S. S. Iyengar, J. Tomasi, M. N. C. Rega, J. M. Millam, M. Klene, J. E. Knox, J. B. Cross, V. Bakken, C. Adamo, J. Jaramillo, R. Gomperts, R. E. Stratmann, O. Yazyev, A. J. Austin, R. Cammi, C. Pomelli, J. W. Ochterski, R. L. Martin, K. Morokuma, V. G. Zakrzewski, G. A. Voth, P. Salvador, J. J. Dannenberg, S. Dapprich, A. D. Daniels, O. Farkas, J. B. Foresman, J. V. Ortiz, J. Cioslowski, D. J. Fox, Gaussian 09, Revision D.01, Gaussian, Inc. Wallingford, CT, 2013.

[5] Yan, Z. & Donald, G, T. The M06 suite of density functionals for main group thermochemistry, thermochemical kinetics, noncovalent interactions, excited states, and transition elements: two new functionals and systematic testing of four M06-class functionals and 12 other functionals. *Theor. Chem. Acc.* **120**, 215–241 (2008).

[6] Stefan. G., Jens, A., Stephan, E. & Helge, K. A consistent and accurate ab initio parametrization of density functional dispersion correction (DFT-D) for the 94 elements H-Pu *J. Chem. Phys.* **132**, 154104–154119 (2010).

[7] Stefan. G., Stephan, E. & Lars. G. Effect of the damping function in dispersion corrected density functional theory *J. Comput. Chem.* **32**, 1456–1465 (2011).

[8] Jacopo. T., Benedetta, M. & Roberto, C. Quantum Mechanical Continuum Solvation Models *Chem. Rev.* **105**, 2999–3094 (2005).

[9] Carlos, G. & H, Bermhard, S. An improved algorithm for reaction path following *J. Chem. Phys.* **90**, 2154 (1989).

[10] Q-X, C. & Tian, L. Independent gradient model based on Hirshfeld partition: A new method for visual study of interactions in chemical systems *J. Comput. Chem.* **43**, 539–555 (2022).

[11]F-W, C. & Tian, Lu. Multiwfn: A multifunctional wavefunction analyzer *J. Comput. Chem.* **33**, 580–592 (2012).

[12] William, H. Andrew, D. & Klaus. S. VMD: Visual molecular dynamics *J. Mol. Graphics*, **14**, 33–38 (1996). (http://www.ks.uiuc.edu/Research/vmd/)

[13] Holger, K., Hamidreza, E., Tian, L. & Saeedreza, E. Exploring Nature and Predicting Strength of Hydrogen Bonds: A Correlation Analysis Between Atoms-in-Molecules Descriptors, Binding Energies, and Energy Components of Symmetry-Adapted Perturbation Theory *J. Comput. Chem.* **40**, 2868–2881 (2019).

[14] R, F, W, Bader. & P, M, Beddall. Virial Field Relationship for Molecular Charge Distributions and the Spatial Partitioning of Molecular Properties *J. Chem. Phys.* **56**, 3320–3329 (1972).

[15] CYLview, 1.0b; Legault, C. Y., Université de Sherbrooke, 2009 (http://www.cylview.org)
